# Hydro- and deutero-deamination of primary amines using O-diphenylphosphinylhydroxylamine

**DOI:** 10.1038/s41467-024-54599-y

**Published:** 2024-11-24

**Authors:** Panpan Ma, Ting Guo, Hongjian Lu

**Affiliations:** 1 State Key Laboratory of Coordination Chemistry, School of Chemistry and Chemical Engineering, Nanjing University, Nanjing, China; 2 The Key Laboratory of Functional Molecular Solids, Ministry of Education, Anhui Normal University, Wuhu, Anhui China

**Keywords:** Synthetic chemistry methodology, Synthetic chemistry methodology

## Abstract

While selective defunctionalizations are valuable in organic synthesis, hydrodeamination of primary amines poses challenges. Deuterodeamination, analogous to hydrodeamination, presents even greater difficulties due to its frequently slower deuteration rate, interference by hydrogenation and constraints in deuterated sources. This study introduces a reliable, robust, and scalable hydro- and deuterodeamination method capable of handling various primary amines. Defined by its mild reaction conditions, rapid completion, simplified purification facilitated by water-soluble byproducts, the method leverages deuterium oxide as a deuterium source and employs commercialized O-diphenylphosphinylhydroxylamine for deamination. Applied to a diverse range of biologically active molecules, it has consistently achieved high yields and efficient deuterium incorporation. By synergizing with site-selective C–H functionalization of primary aliphatic amines, our method reveals synthetic strategies utilizing nitrogen atom as a traceless directing group, encompassing deaminative alkylation, 1,1-deuteroalkylation, 1,1-dialkylation, 1,1,1-deuterodialkylation, C–H arylation, and 1,3-deuteroarylation. Emphasizing this innovation, the processes of deaminative degree-controlled deuteration have been developed.

## Abstract (web summary)

Deuterodeamination, analogous to hydrodeamination, presents greater difficulties due to its frequently slower deuteration rate, interference by hydrogenation and constraints in deuterated sources. Here, the authors report a reliable, robust and scalable hydro- and deuterodeamination method capable of handling various primary amines.

## Introduction

Deuterium represents a naturally stable variant of hydrogen, distinguished merely by the inclusion of an extra neutron. The deuterium plays a significant role in advancing NMR spectroscopy techniques, mass spectrometry and drug developments^1–3^. Therefore, developing deuteration methods advances the discovery of functional molecules across diverse scientific fields. Given the widely presence of d-alkyl groups in current d-drugs^3^ and the prevalence of alkyl moieties in top-selling drugs^4^, the synthesis of d-labelled functionalized alkanes is particularly valuable. Despite the notable efficacy of hydrogen isotope exchange in the direct replacement of hydrogen with deuterium, the targeted incorporation of deuterium at inert aliphatic sites—specifically those devoid of acidity or proximity to radical-stabilizing moieties—presents a substantial obstacle in the absence of directing functionalities. Furthermore, the precise manipulation of the degree of deuteration, a controlled introduction of a defined quantity of deuterium at a particular site, remains unattainable^5–8^. d-Labelling approaches via functional-group transformations provide the ability to selectively incorporate deuterium atom at specific positions within a molecule^9–11^. While several functional-group transformations, such as reductive deuteration of double bonds^12,13^, dehalogenative^14–18^, decarboxylative^19–21^, and dehydroxylative^22,23^ deuterations, have been developed for this purpose, the diversity of organic compounds necessitates ongoing efforts to explore other functional-group transformations using abundant feedstock chemicals, aiming to fulfil the requirements of green and efficient synthesis.

**Fig. 1:**
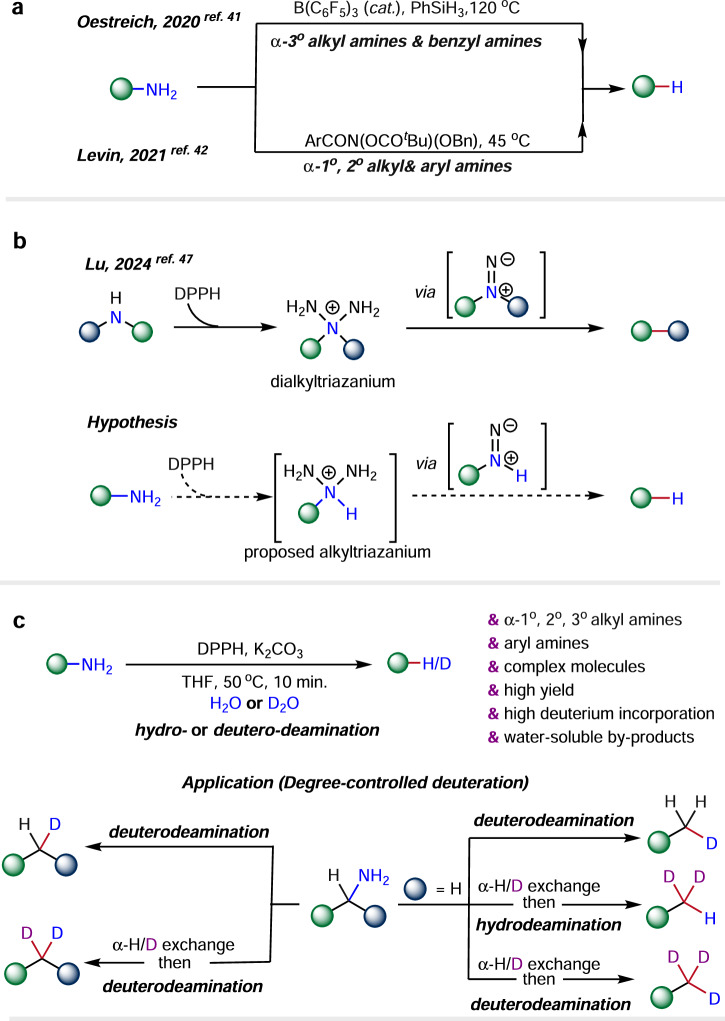
Strategies to access hydro- and deuterondeamination. **a**. Development of direct hydrodeaminaton of primary amines. **b**. N-Deletion of alkylamines by DPPH. **c**. DPPH promoted hydro- and deuterodeamination of primary amines (This work).

Amines are among the most prevalent functional groups found in natural products, pharmaceuticals, and synthetic intermediates. Its synthesis and derivatization can be accomplished through a diverse array of dependable methods^24^. Consequently, the establishment of an efficient deuterodeamination protocol holds the potential to substantially enhance the accessibility and chemical diversity of deuterated compounds. However, the development of deuterodeamination protocols is impeded by the low nucleofugality and high nucleophilicity of amines, alongside the robustness of the C–N bond. Furthermore, the inherent challenges of deuteration, including the potential for unintended hydrogenation reactions and the constraints and expenses linked to employing deuterated materials, further complicate the development of such protocols. The hydrodeamination process, like deuterodeamination in certain aspects, exhibits accelerated hydrogenation kinetics and leverages the abundant accessibility of hydrogen sources; nevertheless, its advancement remains constrained to a restricted scope of research and development^25–27^. The hydrodeamination method often necessitates the utilization of harsh conditions, which can have adverse effects on both the overall yield and the tolerance of functional groups^28–35^. Consequently, the two-step indirect deamination methods involving the pre-activation of the primary amines have been developed, in which the conversion of aliphatic primary amines to reactive isonitriles^36,37^ or Katritzky-type pyridinium salts^38–40^, represents widely employed strategies. One recent pioneering work of direct hydrodeamination from the Oestreich group uses B(C_6_F_5_)_3_ as catalyst and super stoichiometric PhSiH_3_ as the reductant (Fig. 1a)^41^. Despite enabling the hydrodeamination of benzyl amines and α-tertiary alkylamines, this method requires high temperature and anhydrous conditions, and suffers the intolerance with nucleophilic functional groups. Later, the Levin group developed a straightforward method for the direct hydrodeamination of primary amines via isodiazene intermediates, accomplished in a single step with high tolerance for diverse functional groups^42^. However, this technique requires strict exclusion of water and air, and often careful dropwise addition of the pre-prepared anomeric amide to prevent excessive temperature rise, and the substrate scope is limited to α-primary, α-secondary alkylamines and aryl amines. Therefore, it is crucial to pursue further research focused on developing a hydrodeamination methodology that is applicable to a broad spectrum of substrates and employs straightforward experimental procedures.

Our previous research demonstrated that O-diphenylphosphinylhydroxylamine (DPPH), commonly referred to as Harger’s reagent^43^, promotes N-atom deletion^44–46^ of secondary amines, during which a rearrangement of dialkyltriazanium occurs (upper equation, Fig. 1b)^47^. We postulated that a similar intermediate, alkyltriazanium, could be generated and undergo a comparable rearrangement, resulting in the deamination of primary amines (down equation, Fig. 1b). Here, we introduce a reliable, robust, and scalable hydro- and deuteron-deamination reaction by DPPH, based on this hypothesis (Fig. 1c). This method effectively accommodates a diverse array of primary amines, spanning from α-primary to sterically hindered α-tertiary alkylamines and aryl amines, and presents numerous noteworthy benefits, including high efficiency and easy operation in a water-compatible environment. Moreover, it facilitates late-stage modifications for both pharmaceuticals and naturally occurring amines. Furthermore, a strategic approach for site-specific, degree-controlled deuteration^48^ has been developed, leveraging an amine as a traceless directing group.

## Results

### Initial optimization studies

**Fig. 2:**
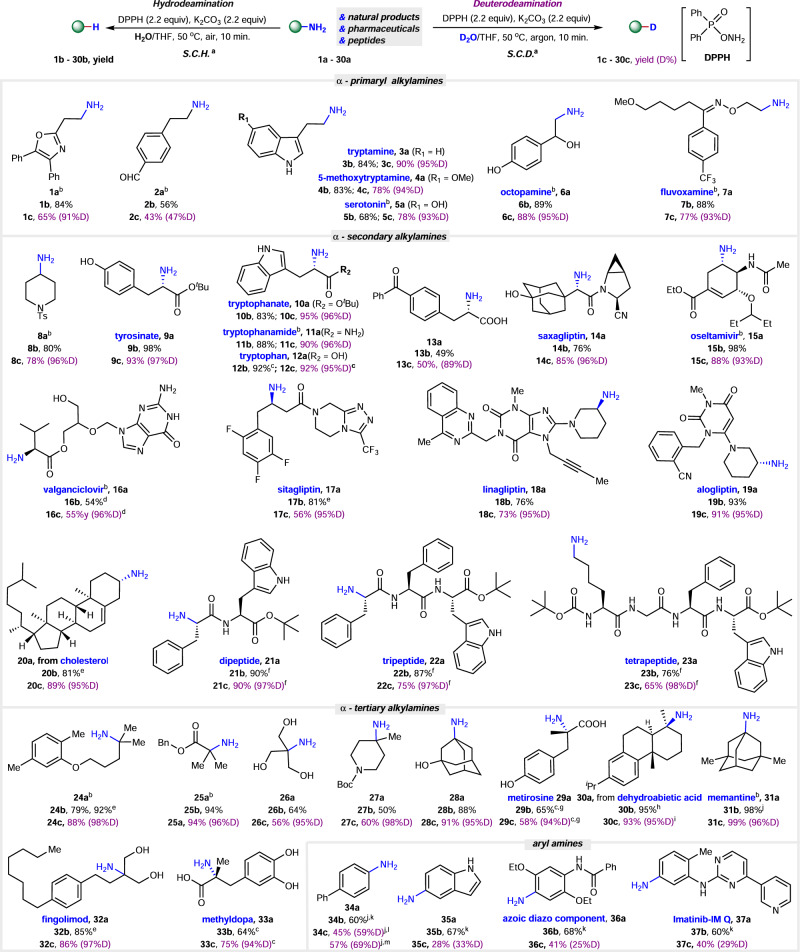
Substrate scope of hydro- and deuterodeamination. ^*a*^ Standard conditions of hydro-(***S.C.H***.) or deuterodeamination (***S.C.D***.): amine **a** (0.4 mmol), DPPH (2.2 equiv), K_2_CO_3_ (2.2 equiv), H_2_O (2.0 mL, 3.3 mL of D_2_O for deuteration), THF (2.0 mL, 3.3 mL for deuteration), 10 min, 50 °C, under air (argon for deuteration). Isolated yield. ^*b*^ Using **a**•HCl (**7a**•maleate, **15a**•H_3_PO_3_) instead of **a**, K_2_CO_3_ (3.2 equiv) was added. ^*c*^ K_2_CO_3_ (3.2 equiv), for 1 h. ^*d*^ DPPH (1.2 equiv), K_2_CO_3_ (1.2 equiv). ^*e*^ Under argon. ^*f*^ 20 min.^g^ Racemized product. ^*h*^*dr* = 1.3. ^*i*^*dr* = 1.1. ^*j*^GC yield using dodecane as a standard. ^*k*^ DPPH (3.0 equiv), K_2_CO_3_ (3.0 equiv), 18-crown-6 (40 mol%), THF (4.0 mL). ^*l*^ Using D_2_O/THF (3.3 mL/0.67 mL) instead of D_2_O/THF (3.3 mL/3.3 mL). ^*m*^ Using d_8_-THF instead of THF.

The optimization of this reaction is detailed in Figures S1–S3 of the Supplementary Information (SI). We identified the standard conditions of hydrodeamination (***S.C.H***.) that require the addition of DPPH (2.2 equiv) and K_2_CO_3_ (2.2 equiv) to a solution of amine (0.4 mmol) in a 1:1 mixture of THF/H_2_O (Fig. 2). This setup facilitates the reaction to complete within 10 min at 50 °C. Under the standard conditions of deuterodeamination (***S.C.D***.) by substituting H_2_O with D_2_O and conducting the reaction under an argon atmosphere, we successfully synthesized the deuterated product.

### Substrate scope

The versatility of both hydro- and deuteron-deamination was investigated in terms of functional-group compatibility and structural diversity. Amines with diverse electron properties, encompassing both electron-rich and electron-deficient alkylamines, serve as excellent substrates (Fig. 2). Amines with differing steric hindrance have been demonstrated as suitable substrates, arranging from α-primary (**1a-7a, 23a**), α-secondary and branched (**9a-14a, 16a-17a, 21a, 22a**), α-secondary and cyclic (**8a, 15a, 18a-20a**), α-tertiary and branched (**24a-26a, 32a, 33a**), to α-tertiary and cyclic (**27a-30a, 31a**) alkylamines. Remarkably, all these substrates demonstrated good to excellent yields for both hydro- and deuterodeamination, with high deuterium incorporation observed in the deuterodeamination reactions. These results are noteworthy given that the specific steric effects of the α-alkyl group have posed challenges in previous direct hydrodeamination reactions, as demonstrated in Levin’s Method^42^. For instance, to achieving reasonable yields for α-secondary alkylamines typically requires the careful dropwise addition of Levin’s reagent (anomeric amide), and the application of α-tertiary alkylamines has proven ineffective in their methodology^42^. In addition, the substrates contained various functional groups, including oxazolyl (**1a**), aldehyde (**2a**), ketone (**13a**), unprotected indolyl (**3a-5a, 10a-12a, 21a-23a**), purinyl (**16a**), triazolyl (**17a**), imidazolyl (**18a**), quinazolinyl (**18a**), electron-rich phenyl (**24a**), electron-deficient phenyl (**19a**), vinyl (**15a, 19a, 20a**), alkynyl (**18a**), ether (**15a**), oxime (**7a**), sulfonamido (**8a**), ester (**9a, 10a, 15a, 21a-23a, 25a**), primary amido (**11a**), secondary amido (**15a, 21a-23a**), tertiary amido (**14a, 18a, 19a**), carbamate (**27a**) and cyano (**14a, 19a**), all of which exhibited successful reactivity in our conditions. More interestingly, different nucleophilic functional groups, such as unprotected phenol group (**5a, 6a, 9a, 33a**), hydroxyl (**6a, 14a, 26a, 28a, 32a**) and carboxylic acid (**12a, 33a**) groups, were tolerant well. Various natural products and pharmaceutical molecules, including tryptamine (**3a**), 5-methoxytryptamine (**4a**), serotonin (**5a**), octopamine (**6a**), fluvoxamine (**7a**), tyrosinate (**9a**), tryptophanate (**10a**), tryptophanamide (**11a**), tryptophan (**12a**), saxagliptin (**14a**), oseltamivir (**15a**), valganciclovir (**16a**), sitagliptin (**17a**), linagliptin (**18a**), alogliptin (**19a**), memantine (**31a**), fingolimod (**32a**) and methyldopa (**33a**) were be used directly, providing ready access to value-added derivatives of feedstock compounds and their deuterated derivatives (>95% D). The valuable compounds 5-cholestene (**20b**) and its d-analogues (**20c**), and 18{19}-norabietatriene (**30b**) and its d-analogues (**30c**) were prepared from the derivatives of non-amino natural products cholesterol and dehydroabietic acid (see pages S6 and S14 in SI). The increasing recognition of the therapeutic potential of peptides has sparked numerous initiatives aimed at developing diverse late-stage functionalization of peptides^49^. Direct selective removal of the N-atom from peptides would be a useful and straightforward strategy to obtain peptide derivatives, however, it is shown to be not sufficient under the reported methods^42^. In our conditions, deamination of the terminal amino group in the dipeptide (**21a**) and tripeptide (**22a**), as well as the amino group in the side chain in the tetrapeptide (**23a**), were successfully achieved with good to excellent yields and high deuterium incorporation. The α-chiral centre is not retained during the transformation, as evidenced by the racemic product **29c** obtained from the reaction of chiral metirosine **29a**. Finally, aryl amines (**34a**-**37a**) were tested, yielding the corresponding hydrodeamination products (**34b**-**37b**) in good yields under modified reaction conditions (note k in Fig. 2, see details in Fig. S4 in SI). However, the deuterodeamination process was less efficient, resulting in a moderate yield and moderate deuterium incorporation for the desired d-aryl product **34c-37c**.

### Synthetic applications

**Fig. 3:**
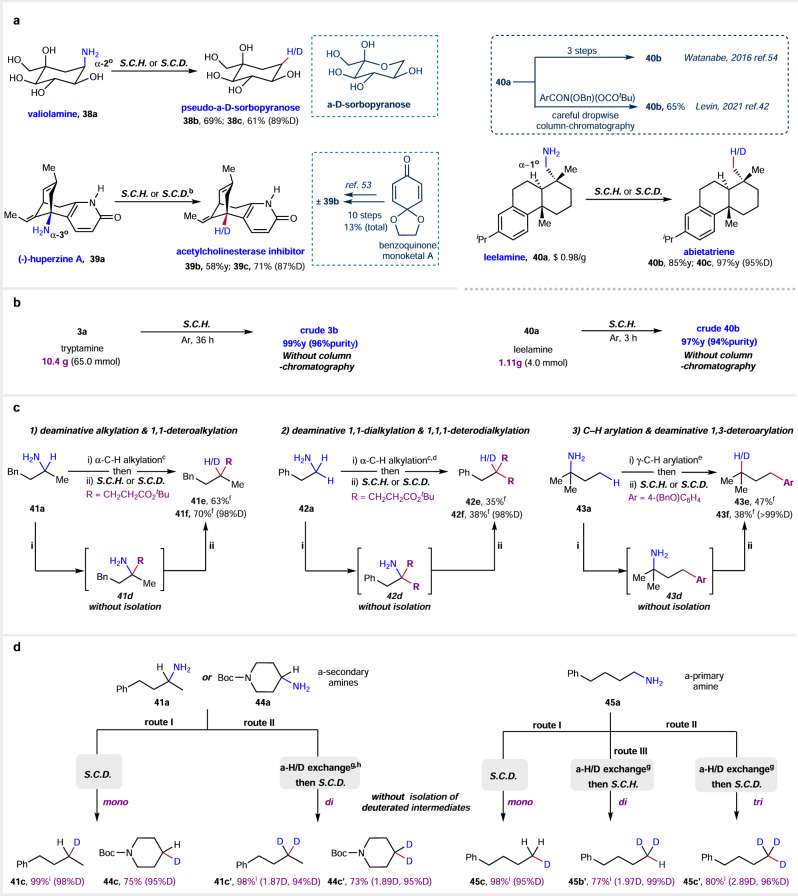
Synthetic applications. **a**. Representative examples for the convenient synthesis of valuable compounds and their deuterated derivatives. **b**. Gram-scale synthesis without column chromatograph. **c**. C–H functionalization and N-deletion synthetic sequence. **d**. Degree-controlled deuteration by using D_2_O. ^*a*^ Standard conditions of hydro- (***S.C.H***.) or deuterodeamination (***S.C.D***.): amine **a** (0.4 mmol), DPPH (2.2 equiv), K_2_CO_3_ (2.2 equiv), H_2_O (2 mL, 3.3 mL of D_2_O for deuteration), THF (2 mL, 3.3 mL for deuteration), 10 min, 50 °C, under air (argon for deuteration). ^*b*^ DPPH (4.2 equiv), K_2_CO_3_ (4.2 equiv). ^*c*^ Conditions for α-C–H alkylation: 4CzIPN or (1 mol%), Bu_4_N^+^N_3_ (10 mol%), *tert*-butyl acrylate (1.0 equiv), LED, MeCN, 20 h. ^*d*^ Using Ir[dF(CF_3_)ppy]_2_(dtbbpy)PF_6_ instead of 4CzIPN and *tert*-butyl acrylate (3.0 equiv). ^*e*^ Conditions for γ-C–H arylation: Pd(OAc)_2_ (10 mol%), HO_2_CCHO (20 mol%), AgTFA (1.5 equiv), 4-(BnO)C_6_H_4_I (1.5 equiv), HOAc, 100 ^o^C, 15 h. ^*f*^ Yield for the two steps. ^*g*^ Conditions for α-H/D exchange: 4CzIPN (1 mol%), iPr_3_SiSH (30 mol%), EtOAc, D_2_O, LED, 48 h. ^*h*^ Using 3DPA2FBN (2 mol%) instead of 4CzIPN (1 mol%). ^*i*^ GC yield using dodecane as a standard.

The direct late-stage hydro- and deuterodeamination of readily available complex molecules may provide ready access to value-added feedstock compounds and their deuterated derivatives which are difficult to be achieved by known methods. We illustrate this advancement with three illustrative examples (Fig. 3a). Pseudosugar, unlike true sugar which exhibit an anomeric and a pyranose-furanose equilibrium in an aqueous solution, offers a stable and preferred conformation in solution^50^. This stability provides a clear understanding of the exact conformation of each hydroxyl group. The N-deletion of valiolamine (**38a**), without the need for protecting multiple hydroxyl groups, enables the generation of pseudo-α-D-sorbopyranose (**38b**) and its d-analogues (**38c**) directly. The simplified model system allows for a more focused exploration of the functional aspects and structural intricacies associated with D-sorbose without the complicating factors introduced by the dynamic equilibria observed in true sugars, potentially facilitating the bioactive studies of D-sorbose. Huperzine A (**39a**), an α-tertiary alkylamine, has received approval as a palliative drug for Alzheimer’s disease in China^51^. Mechanistic studies have revealed that the bridgehead amino group of huperzine A does not directly interact with the protein^52^, prompting interest in synthesizing deamino huperzine A (**39b**)^53^. The racemic form of deamino huperzine A (±**39b**) was successfully synthesized in ten steps, commencing from benzoquinone monoketal A, with an overall yield of 13%^53^. The synthetic route for chiral **39b** from the N-atom deletion of chiral **39a** has not been established, and it is conjectured that the steric hindrance of the amine group and the existence of various double bonds with distinct reactivities render this structure susceptible to disintegration, presenting challenges in removing the N-atom by conventional methods. Interestingly, under the standard conditions, chiral **39b** and its d-analogues (**39c**) were successfully obtained in good yield and d-incorporation. Hydrodeamination of leelamine (**40a**), an α-primary alkylamine with low price, can afford abietatriene (**40b**) which is not commercially available and was used as a key starting material in several total syntheses^54^. Levin successfully devised the most efficient synthetic pathway, resulting in the isolation of **40b** with a yield of 65%, achieved through the purification using silica-gel column chromatograph^42^. Under the standard conditions we established, abietatriene (**40b**) was obtained in an 85% isolated yield, and d-abietatriene (**40c**) was achieved with a remarkable 97% yield and 95% deuterium incorporation.

Furthermore, the gram-scale hydrodeamination reactions for leelamine **40a** and tryptamine **3a** were successfully executed, yielding their respective products, **40b** and **3b**, with impressive efficiency (Fig. 3b). Specifically, **40b** was obtained with a 97% yield and 94% purity, while **3b** showcased a 99% yield and 95% purity. This was achieved simply through washing with acidic water, circumventing the need for traditional silica gel column chromatography, as detailed in SI. The results indicate that most byproducts from these reactions are water-soluble, which not only streamlines the purification process but also potentially enables the direct application of crude products in further synthesis steps. Such outcomes highlight the method’s practicality and its advantages in simplifying synthetic procedures.

Through the seamless incorporation of the N-deletion methodology into synthetic transformations involving amines, innovative retrosynthetic approaches can be introduced. The amino group is a widely used directing group in C–H functionalization^55^. The combination of N-direct C–H functionalization with the N-deletion process, can establish a reliable gateway for traceless C–H functionalization (Fig. 3c). As an illustration, leveraging the recent advancements in the direct α C–H functionalization of unprotected primary amines by Cresswell et al. ^56^ we have developed various synthetic strategies, such as deaminative alkylation (**41e**) and 1,1-deteroalkylation (**41f**) of α-secondary amine **41d**, 1,1-dialkylation (**42e**) and 1,1,1-deterodialkylation (**42f**) of α-primary amine (**42d**). Furthermore, selective γ-C–H functionalization of amines, developed by the Ge group^57^, was followed by N-deletion reactions, resulting in a C–H arylation process (**43e**) and a 1,3-deteroarylation process (**43f**) that employs nitrogen as a traceless directing group.

Inspired by recent breakthroughs in the direct substitution of α-C–H bonds in primary amines with deuterium via H/D exchange^12–18^, and the existing gap in the development of precision-controlled deuteration techniques^48^, we developed a methodology. This approach allows for precision-controlled deuteration, employing the nitrogen atom as a traceless directing group, as illustrated in Fig. 3d. Building upon the recent work of Wang et al. on the direct α-C–H deuteration of unprotected primary amines^58^, three distinct sets of reaction protocols were devised by utilize D_2_O as the deuterium source. As previous demonstrated, under the established standard conditions of deuterodeamination (***S.C.D***.), the protocol consistently yielded high monodeuteration across a variety of substrates (Figs. 2 and 3d). To achieve deaminative dideuteration of α-secondary alkylamines or trideuteration of α-primary alkylamines, the α-deuteration of amine was initially conducted via H/D exchange with D_2_O using an organophotocatalytic process^58^. After solvent removal, the resulting crude α-deuterated intermediates **41a, 44 a, 45a** were directly subjected to ***S.C.D***. With these conditions (route II), this method introduced 1.87D for the methylene in linear product **41c’**, 1.89D for the methylene in cyclic product **44c’**, and 2.89D for the methyl group in alkane **45c’**. Employing the same synthetic procedure under the standard conditions of hydrodeamination (***S.C.H***.) instead of ***S.C.D***. in the secondary step (route III) selectively installed 1.97 D in the methyl group of product **45b’**. In summary, degree-controlled deuterations can be accomplished with satisfactory overall yields and high d-incorporation. Notably, these represent only a subset of the potential applications due to the richness of amine chemistry, and the extension to a broader range of examples promises to unveil additional functionalities and applications.

### Mechanistic studies

**Fig. 4:**
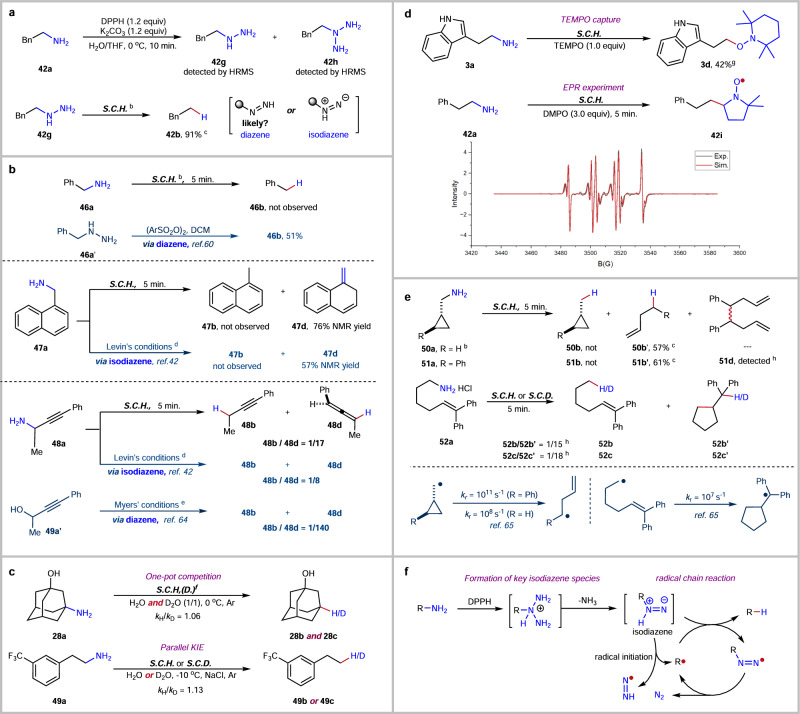
Mechanistic studies. **a**. Detection of key intermediates. **b**. Comparison of diazene and isodiazene. **c**. Kinetic isotope effect (KIE) experiments. **d**. Radical trapping reactions. **e**. Radical clock experiments. **f**. Proposed mechanism of hydrodeamination. ^*a*^ Standard conditions of hydro- (***S.C.H***.) or deuterodeamination (***S.C.D***.): amine **a** (0.4 mmol), DPPH (2.2 equiv), K_2_CO_3_ (2.2 equiv), H_2_O (2 mL, 3.3 mL of D_2_O for deuteration), THF (2 mL, 3.3 mL for deuteration), 10 min, 50 °C, under air (argon for deuteration). ^*b*^ Using CDCl_3_ instead of THF. ^*c*^ NMR yield. ^*d*^ (4-CF_3_C_6_H_4_)CON(OCO^*t*^Bu)(OBn) (1.2 equiv), THF, N_2_, 45 ° C. ^*e*^ PPh_3_ (1.5 equiv), DEAD (1.5 equiv), NBSH (1.5 equiv), THF, −15 °C. ^*f*^ Using a 1:1 mixture of D_2_O and H_2_O instead of pure H_2_O in ***S.C.H***. ^*g*^ 33% yield of deamination product **2b**. ^*h*^ Detected by GCMS.

To further understand the reaction mechanism, several experiments were performed (Fig. 4). The reaction of phenylethyl amine **42a** was conducted at 0 °C with 1.2 equivalents of DPPH for 10 min (Fig. 4a). In the HRMS analysis of the reaction mixture, we identified two notable compounds with molecular weight of 137.1070 and 152.1189. These two compounds are likely phenylethyl hydrazine **42g** (Mw + H^+^ = 137.1073) and a triazane derivative **42h** (Mw + H^+^ = 152.1182). To further explore this, we subjected commercially available phenylethyl hydrazine **42g** to our standard reaction conditions with CDCl_3_ as a co-solvent. Through this experiment, we were able to detect the formation of ethyl benzene **42b** in 91% NMR yield. This result supports the hypothesis that alkyl hydrazine functions as one of the intermediates in this process. Previous research has shown that alkyl hydrazine can produce a defunctionalized product under mild oxidative conditions, involving an alkyl diazene intermediate^59,60^. Additionally, drawing upon Levin’s findings^42,44^ and our own research on N-deletion of secondary amines^45,47,61,62^, we consider the possibility of an alkyl isodiazene intermediate forming. To investigate these intermediates further, we directly compared various reactions (Fig. 4b). For example, when we treated benzyl amine **46a** with DPPH, it failed to produce the expected deamination product, toluene, unlike the oxidation of benzyl hydrazine **46a’**, which successfully yielded toluene at a 51% yield through a diazene intermediate^60^. Treating 1-naphthalenamine **47a** did not produce any deamination product **47b**, but led to a 76% NMR yield of the deaminative rearrangement product **47** **d**. This compares to a 57% NMR yield of **47b** under Levin’s conditions^42^, suggesting the involvement of a [2,3]-sigmatropic rearrangement of an isodiazene intermediate^63^. Moreover, when propargylic amine **48a** reacted with DPPH, it resulted in the production of alkyne **48b** and allene **48** **d** at an approximate ratio of 1:17. This outcome closely aligns with the 1:8 ratio reported under the conditions described by Levin (indicative of an isodiazene intermediate) and contrasts with the 1:140 ratio observed under the conditions performed by Myers^64^, which initiates from propargylic alcohol **48a’** and includes a diazene intermediate in the process. These results suggest that an isodiazene, rather than a conventional diazene, may be involved in our reactions. To ascertain the rate-controlling step, we conducted an analysis of the kinetic isotope effect (KIE) by investigating the reaction of **28a** in a solvent mixture of H_2_O and D_2_O, as well as the corresponding reactions of amine **49a** in either H_2_O or D_2_O (as illustrated in Fig. 4c). This analysis showed a one-pot competition KIE with a ratio of k_*H*_/k_*D*_ = 1.06 and a parallel KIE with a ratio of k_*H*_/k_*D*_ = 1.13. Then, two radical trapping experiments were conducted, as depicted in Fig. 4d. In the first experiment, we used an equivalent amount of TEMPO (2,2,6,6-tetramethyl-1-piperidinyloxy) to trap radicals from the reaction of tryptamine **3a**, successfully isolating the TEMPO capture product **3** **d** with a 42% yield. In the second experiment, Electron Paramagnetic Resonance spectra were recorded using 5,5-dimethyl-1-pyrroline-N-oxide (DMPO) for spin trapping in the reaction of phenylethyl amine **42a**, and the DMPO-spin adduct **42i** was observed. Furthermore, several radical clock experiments were conducted (Fig. 4e). While no direct deamination products **50b** and **51b** were observed, ring-opening products **50b’** and **51b’** were obtained with good yield in the reactions of cyclopropylmethyl radical clock **50a** (*k*_*r*_ ≈ 10^8 ^s^−11^) and its phenyl analogous **51a** (*k*_*r*_ ≈ 10^11 ^s^−11^)^65^. In addition, the radical self-coupling products **51** **d** were detected obviously by GCMS in the reaction of **51a**. Likewise, small quantities of the hydrodeamination product **52b**, as well as the principal ring-closed products **52b’** and **52c’**, were produced from the reactions involving the 5-vinylmethyl radical clock **52a** (*k*_*r*_ ≈ 10^7 ^s^−11^)^65^. These results indicate the rate of alkyl radical abstracting hydrogen is slower than the rearrangement rates of these radical clocks, suggesting a cage-escaping (*k*_(cage escape)_ ≈ 10^10 ^s^−1^)^65,66^, free alkyl radical is involved in the deamination process. According to these experimental results and previous studies^67–70^, a mechanism was proposed in Fig. 4f. Amine reacts with two equivalents of DPPH to form possible triazanium intermediate. Its rearrangement under basic conditions generates a primary isodiazene intermediate. Its decomposition produces an initial alkyl radical and a diazane radical (radical initiation). Then, alkyl radical abstract hydrogen from primary isodiazene intermediate to produce desired deamination product and alkyl diazenyl radical species. By releasing nitrogen gas, the rearrangement of the alkyl diazenyl radical species regenerates alkyl radical, finishing a chain, hydrogen-atom transfer process.

## Discussion

In this study, we present a groundbreaking direct hydro- and deuteron-deamination method capable of accommodating a wide range of primary amines. These include α-primary, α-secondary, sterically hindered α-tertiary alkylamines, and aryl amines. The reaction demonstrates exceptional tolerance to various functional groups, including nucleophilic phenols, hydroxyls, amides, and carboxylic acids. Impressively, the method has been successfully applied to versatile bio-relevant compounds, spanning pharmaceutical molecules, amino acids, amino sugars, peptides, and natural products. The reaction of aliphatic amines exhibits high yields for both hydrodeamination (up to 98%) and deuterodeamination (up to 99%), accompanied by an impressive 96% deuterium incorporation. This approach boasts several noteworthy features, including mild aqueous conditions, rapid completion within ten minutes, easy purification due to high yield and water-soluble major byproducts, the utilization of deuterium oxide as a deuterium source, and commercially available DPPH as the deamination reagent. Abundant synthetic methods exist for the synthesis and transformations of nitrogen-containing compounds, wherein N-atom plays a pivotal role. When combined with the deamination reaction, these methods open avenues for chemical conversions, offering a unique and versatile means to guide reactions toward desired outcomes, with nitrogen atom serving as a traceless directing group. Expanding on this concept, our approach, when combined with N-directed α- or γ-C–H functionalization, unlocks diverse strategic applications. These include deaminative alkylation, 1,1-deuteroalkylation, 1,1-dialkylation, 1,1,1-deuterodialkylation, C–H arylation, and 1,3-deuteroarylation. Combing with N-direct α-H/D exchange reaction, we have devised a one-pot process for selective deaminative mono-, di-, and tri-deuteration at the original amino site, enhancing the accessibility of degree-controlled deuterated compounds. These developments contribute to diverse applications in chemical and pharmaceutical sciences, emphasizing the versatility and impact of our methodology.

## Methods

### General procedures

In a 25 mL Schlenk tube equipped with a stirring bar, amine **a** (0.4 mmol, 1.0 equiv) was added, followed by THF (2.0 mL, or 3.3 mL for deuteration), H_2_O (2.0 mL, or 3.3 mL of D_2_O for deuteration), K_2_CO_3_ (121.6 mg, 2.2 equiv), and DPPH (205.2 mg, 2.2 equiv) in sequence. The tube was sealed (under argon for deuteration) and placed on a preheated heating module at 50 °C. The mixture was stirred vigorously at 800 rpm for 10 min. After cooling to room temperature, 5.0 mL of aqueous NaCl solution was added, and the mixture was extracted three times with 5.0 mL of ethyl acetate. The combined organic layers were dried over anhydrous Na_2_SO_4_, and the product was purified by flash chromatography over silica gel to yield the desired compound.

## Supplementary information

Supplementary Information

Transparent Peer Review file

## Data Availability

The data supporting the findings of this study are available within the paper and its Supplementary Information files. Raw data are available from the corresponding author on request.
