# Supplementary material for: Hydro- and deutero-deamination of primary amines using O-diphenylphosphinylhydroxylamine
Source: Nat Commun. 2024 Nov 24;15:10190. doi: 10.1038/s41467-024-54599-y (PMC11586428; doi:10.1038/s41467-024-54599-y)
Supplement: Supplementary file 1 — Supplementary Information [file 41467_2024_54599_MOESM1_ESM.pdf]

# Supplementary Information

# Hydro- and Deutero-deamination of Primary Amines using O-Diphenylphosphinylhydroxylamine

## Content


1. General Information ....................................................................................S2
2. Preparations of Amines ..................................................................................S4
3. Optimization of the Reaction Conditions ...........................................................S17
4. General Procedure for Hydro- and Deuterodeaminatin of Primary Amines. .....S21
5. Characterization Data of Products ....................................................................S22
6. Synthetic Applications.....................................................................................S67

6.1 Synthesis of Pseudo-α-D-sorbopyranose .........................................................S67

6.2 Synthesis of Acetylcholinesterase Inhibitors **39b** & **39c** ..................................S68

6.3 Synthesis of Abietratriene **40b** & Deutorated Abietratriene **40c** .....................S69

6.4 Ten-gram-scale Preparation of **3b** without Column Chromatography............S71

6.5 Gram-scale Preparation of Abietratriene (**40b**) without Column-Chromatography.........................................................................................S72

6.6 C-H Functionalization & N-deletion Synthetic Sequence ...............................S73

6.7 Degree-controlled Deuteration by Using $D_2O$ .................................................S77

7. Mechanistic Studies.......................................................................................S85

7.1 Detection of Key Intermediates.......................................................................S85

7.2 Comparison of Diazene and Isodiazene ..........................................................S86

7.3 Kinetic Isotope Effect (KIE) Experiments .......................................................S89

7.4 TEMPO Capture Reaction ..............................................................................S91

7.5 EPR Experiment.............................................................................................S93

7.6 Radical Clock Experiments.............................................................................S93

8. Copies of NMR Spectra.................................................................................S102
9. References ..................................................................................................S203

# 1. General Information

## 1.1 Methods

All reactions were carried out in anhydrous solvents and performed under ambient conditions unless otherwise noted. Thin layer chromatography (TLC) was performed on pre-coated silica gel GF254 plates. Visualization of TLC was achieved using UV light (wavelength 254 nm), PMA stain (phosphomolybdic acid hydrate), $I_2$ chamber (dispersed in silica gel), and/or $KMnO_4$ stain.

## 1.2 Instrumentation

Column chromatography was performed on silica gel (300-400 mesh) using a proper eluent unless otherwise noted. NMR was recorded on a FT AM 400 (400 MHz). Chemical shifts were reported in parts per million (ppm) referenced to the appropriate solvent peaks (δ 7.26 ppm for $CDCl_3$, δ 2.50 ppm for DMSO-$d_6$; δ 77.0 ppm for $CDCl_3$, δ 40.0 ppm for DMSO-$d_6$ in fully decoupled $^{13}C$ spectra). The following abbreviations were used to describe peak splitting patterns: br = broad, s = singlet, d = doublet, t = triplet, q = quartet, hept = heptet, dd = doublet of doublet, td = triplet of doublet, ddd = doublet of doublet of doublet, m = multiplet. Coupling constants *J* were reported in hertz (Hz). GC-MS was recorded on a Shimadzu GCMS-QP2010 Ultra with an SH-I-5Sil MS capillary column. High resolution mass spectra were obtained with the UHD Accurate-Mass Q-TOF.

## 1.3 Materials and Reagents

*O*-(Diphenylphosphinyl) hydroxylamine (DPPH, CAS = 72804-96-7), *O*-(2,4-dinitrophenyl)hydroxylamine (CAS = 17508-17-7), (aminooxy)sulfonic acid (CAS = 2950-43-8), *O*-tosylhydroxylamine (CAS = 52913-14-1) are commercially available and were used as purchased.

Anhydrous DMSO (dimethyl sulfoxide), DMF (*N,N*-dimethylformamide), $CH_3CN$ (acetonitrile), EtOH, MeOH, 1,4-dioxane and deuterated solvents were commercially available and used as received. Anhydrous diethyl ether, THF

(tetrahydrofuran) and DCM (dichloromethane) were obtained from an Innovative Technology, INC. PS-MD-5 solvent purification system.

## 1.4 Abbreviations

equiv = equivalents, r.t. = room temperature, *d.r.* = diastereoisomer ratio, aq. = aqueous, EA = ethyl acetate, PE = petroleum ether, DCM = methylene chloride, HCl = hydrochloride, *in vacuo* = in vacuum, DBU = 1,8-Diazabicyclo[5.4.0]undec-7-ene, DIPEA = *N*,*N*-diisopropylethylamine.

## 2. Preparations of Amines

Tryptamine (**2a**) (CAS = 61-54-1), 5-methoxytryptamine (**4a**) (CAS = 608-07-1), serotonin hydrochloride (**5a**) (CAS = 153-98-0), octopamine (**6a**) (CAS = 104-14-3), fluvoxamine maleate (**7a**) (CAS = 61718-82-9), *tert*-butyl L-tyrosinate (**9a**) (CAS = 16874-12-7), L-tryptophanamide hydrochloride (**11a**) (CAS = 5022-65-1), L-tryptophan (**12a**) (CAS = 73-22-3), saxagliptin (**14a**) (CAS = 361442-04-8), oseltamivir phosphate (**15a**) (CAS = 204255-11-8), valganciclovir hydrochloride (**16a**) (CAS = 175865-59-5), sitagliptin (**17a**) (CAS = 486460-32-6), linagliptin (**18a**) (CAS = 668270-12-0), alogliptin (**19a**) (CAS = 850649-61-5), benzyl 2-amino-2-methylpropanoate hydrochloride (**25a**) (CAS = 60421-20-7), tris(hydroxymethyl)aminomethane (**26a**) (CAS = 77-86-1), 1-Boc-4-amino-4-methylpiperidine (**27a**) (CAS = 163271-08-7), 3-amino-1-hydroxyadamantane (**28a**) (CAS = 702-82-9), memantine HCl (**31a**) (CAS = 41100-52-1), fingolimod (**32a**) (CAS = 162359-55-9), methyldopa (**33a**) (CAS = 555-30-6), valiolamine (**38a**) (CAS = 83465-22-9), (-)-huperzine A (**39a**) (CAS = 102518-79-6) and leelamine (**40a**) (CAS = 1446-61-3), are commercially available and were used as purchased. Other amines were synthesized as follows.

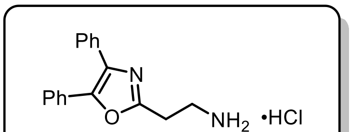

**1a**

**2-(4,5-diphenyloxazol-2-yl)ethan-1-amine hydrochloride (1a).** To a solution of oxaprozin (1.47 g, 5.0 mmol, 1.0 equiv) in toluene (17 mL) under argon, triethylamine (1.5 mL, 11.0 mmol, 2.2 equiv) and diphenylphosphoryl azide (1.2 mL, 5.5 mmol, 1.1 equiv) were added via syringe. The reaction mixture was allowed to stir at 90 °C for 2 hours before it was cooled down to room temperature. It was then diluted with EA and transferred to a separatory funnel. The organic layer was washed twice with saturated aqueous $NaHCO_3$ solution and then with brine. The organic layer was dried over

anhydrous $Na_2SO_4$, filtered and concentrated to an oil. The oil was transferred to a 25 mL round bottomed flask equipped with a stirring bar, and the residue was suspended in 5M HCl (3 mL). A condenser was attached to the flask and the mixture was heated to reflux. After 2 hours, the reaction mixture was cooled to room temperature. The aqueous phase was concentrated under reduced pressure to give a solid. The solid obtained was washed with DCM, ether and dried *in vacuo* to produce 0.29 g of compound **1a** (29% yield).

**$^1$H NMR** (400 MHz, DMSO-$d_6$) δ 8.22 (s, 3H), 7.69 – 7.31 (m, 10H), 3.32 – 3.20 (m, 4H).

**$^{13}$C NMR** (101 MHz, DMSO-$d_6$) δ 160.2, 145.6, 134.9, 132.3, 129.5, 129.2, 128.8, 127.9, 127.0, 36.6, 26.1.

**HRMS** m/z (ESI) calcd. for $C_{17}H_{17}N_2O^+$ (M + $H^+$) 265.1335, found 265.1339.

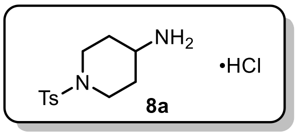


**1-tosylpiperidin-4-amine hydrochloride (8a).** Prepared from 1-tosylpiperidine-4-carboxylic acid with the same method used to synthesize 2-(4,5-diphenyloxazol-2-yl)ethan-1-amine hydrochloride **(1a)**. The white solid obtained was washed with DCM, ether and dried *in vacuo* to produce 1.02 g of compound **8a** (80% yield).

**$^1$H NMR** (400 MHz, DMSO-$d_6$) δ 8.17 (s, 3H), 7.62 (d, $J$ = 7.9 Hz, 2H), 7.46 (d, $J$ = 7.9 Hz, 2H), 3.63 (d, $J$ = 11.8 Hz, 2H), 3.03 (s, 1H), 2.41 (s, 3H), 2.31 (t, $J$ = 11.8 Hz, 2H), 1.95 (d, $J$ = 12.5 Hz, 2H), 1.62 – 1.50 (m, 2H).

**$^{13}$C NMR** (101 MHz, DMSO-$d_6$) δ 144.1, 133.0, 130.4, 127.9, 46.8, 44.6, 29.3, 21.5.

**HRMS** m/z (ESI) calcd. for $C_{12}H_{19}N_2O_2S^+$ (M + $H^+$) 255.1162, found 255.1168.

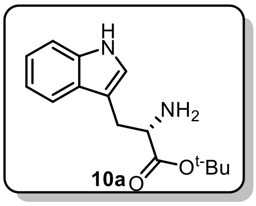


***Tert*-butyl L-tryptophanate (10a).** Synthesized according to the literature[1] from L-

tryptophan. The physical and spectroscopic data are consistent with those reported in the literature[1].

**$^1$H NMR** (400 MHz, DMSO-$d_6$) δ 10.83 (s, 1H), 7.53 (d, $J$ = 7.8 Hz, 1H), 7.33 (d, $J$ = 8.0 Hz, 1H), 7.12 (d, $J$ = 2.3 Hz, 1H), 7.06 (t, $J$ = 7.5 Hz, 1H), 6.97 (t, $J$ = 7.4 Hz, 1H), 3.52 (t, $J$ = 6.5 Hz, 1H), 3.38 (s, 2H), 3.01 – 2.84 (m, 2H), 1.30 (s, 9H).

**$^{13}$C NMR** (101 MHz, DMSO-$d_6$) δ 175.0, 136.6, 128.0, 124.0, 121.3, 119.0, 118.7, 111.8, 110.8, 80.1, 56.2, 49.1, 31.4, 28.1.

**HRMS** m/z (ESI) calcd. for $C_{15}H_{21}N_2O_2^+$ (M + H$^+$) 261.1598, found 261.1593.

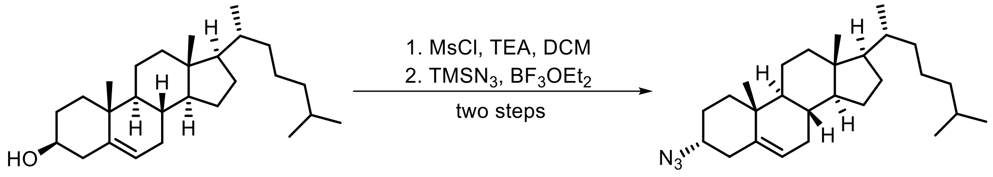


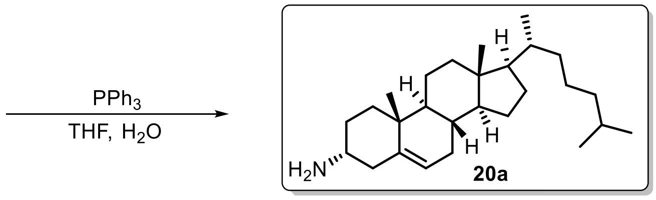


**(3R,8S,9S,10R,13R,14S,17R)-10,13-dimethyl-17-((R)-6-methylheptan-2-yl)-2,3,4,7,8,9,10,11,12,13,14,15,16,17-tetradecahydro-1H-cyclopenta[a]phenanthren-3-amine (20a).** To a solution of sterol (3.87g, 10. 0 mmol) in anhydrous DCM (100 mL) at 4 °C was added triethylamine (2.80 mL, 20.0 mmol), followed by the addition of methanesulfonyl chloride (850 μL, 11.0 mmol). The reaction was maintained at 4 °C for 30 min, warmed to room temperature and stirred for 6 h. When sterol was completely consumed as evidenced by TLC (eluent: hexanes/EA = 5/1), the reaction was diluted with DCM (200 mL). The organic phase was washed with saturated aqueous $NaHCO_3$ solution (250 mL), dried over anhydrous $Na_2SO_4$, filtered and concentrated *in vacuo*. Column chromatography (eluent: hexanes/EA = 10/1) afforded (3S,8S,9S,10R,13R,14S,17R)-10,13-dimethyl-17-((R)-6-methylheptan-2-yl)-2,3,4,7,8,9,10,11,12,13,14,15,16,17-tetradecahydro-1H-cyclopenta[a]phenanthren-3-yl methanesulfonate (3.95 g, 85% yield) as a white solid[2]. **$^1$H NMR** (400 MHz, Chloroform-$d$) δ 5.48 – 5.38 (m, 1H), 4.56 – 4.48 (m, 1H), 3.00 (s, 3H), 2.57– 2.45 (m,

2H), 2.12 – 1.71 (m, 6H), 1.65 – 0.89 (m, 26H), 0.87 – 0.85 (m, 6H), 0.67 (s, 3H).

To a solution of (3S,8S,9S,10R,13R,14S,17R)-10,13-dimethyl-17-((R)-6-methylheptan-2-yl)-2,3,4,7,8,9,10,11,12,13,14,15,16,17-tetradecahydro-1H-cyclopenta[a]phenanthren-3-yl methanesulfonate (1.86 g, 5. 0 mmol) in anhydrous DCM (50 mL) was added $TMSN_3$ (0.725 mL, 5.5 mmol), followed by $BF_3$•$OEt_2$ (1.24 mL, 10 mmol). The reaction was stirred at room temperature for 3 h. When the starting material was completely consumed as evidenced by **TLC:** $R_f$= 0.4 (eluent: petroleum ether, visualized by UV light), the reaction was slowly poured into aqueous NaOH (2.0 M, 5 mL) and stirred for 5 min. The organic layer was removed, and the aqueous layer was extracted with DCM (100 mL). The combined organic extracts were washed with saturated aqueous NaCl solution (15 mL), dried over anhydrous $Na_2SO_4$, filtered and concentrated *in vacuo* to afford the crude products as a light-yellow solid. The crude product was purified by flash chromatography over silica gel to afford (3R,8S,9S,10R,13R,14S,17R)-3-azido-10,13-dimethyl-17-((R)-6-methylheptan-2-yl)-2,3,4,7,8,9,10,11,12,13,14,15,16,17-tetradecahydro-1H-cyclopenta[a]phenanthrene (**18a-I**, 1.81 g, 88% yield) as a white solid. The physical and spectroscopic data is consistent with those reported in the literature[2]. **$^1$H NMR** (400 MHz, Chloroform-*d*) δ 5.43 – 5.34 (m, 1H), 3.20 (m, 1H), 2.29 (d, *J* = 8.1 Hz, 2H), 2.07 – 1.79 (m, 5H), 1.61 – 0.85 (m, 33H), 0.68 (s, 3H).

To a solution of **20a-I** (1.36 g, 3.3 mmol, 1.0 equiv) in anhydrous THF (6 mL) was added a solution of $PPh_3$ (8.66 g, 33.0 mmol, 10 equiv) in anhydrous THF (15 mL). 6.6 mL of $H_2O$ was added to the mixture after 30 minutes. The reaction was stirred overnight at room temperature in a flask equipped with a balloon to trap the released nitrogen gas. The reaction was diluted extensively with toluene. The mixture was concentrated under vacuum. The crude product was purified by column chromatography to afford 0.99 g of **20a** (78% yield).

**TLC:** $R_f$= 0.2 (eluent: DCM/MeOH = 2/1, visualized by UV light).

**$^1$H NMR** (400 MHz, Chloroform-*d*) δ 5.31 (d, *J* = 5.0 Hz, 1H), 2.64 – 2.56 (m, 1H), 2.30 – 0.78 (m, 42H), 0.67 (s, 3H).

**$^{13}$C NMR** (101 MHz, Chloroform-*d*) δ 120.6, 56.8, 56.2, 52.0, 50.3, 43.4, 42.3, 39.8,

39.5, 38.2, 36.6, 36.2, 35.8, 32.7, 31.9, 28.2, 28.0, 24.3, 23.8, 22.8, 22.6, 21.0, 19.5, 18.7, 11.9.

**HRMS** m/z (ESI) calcd. for $C_{27}H_{48}N^+$ (M + $H^+$) 386.3781, found 386.3783.

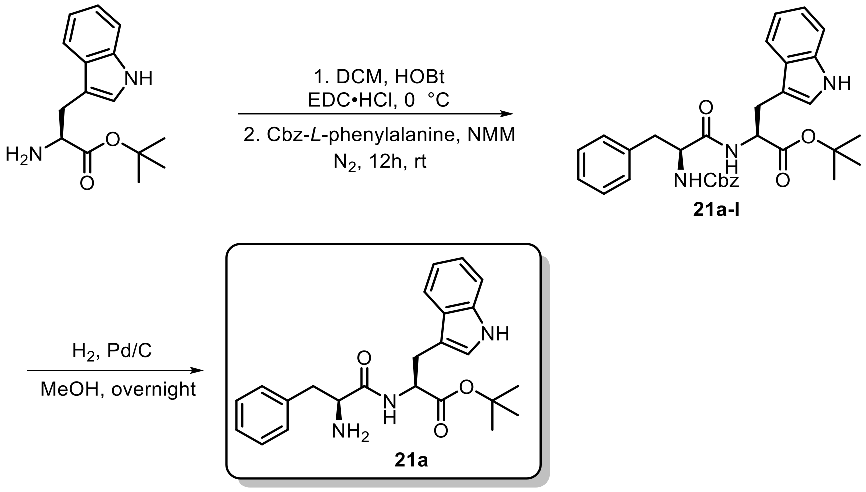


**Synthesis of *tert*-butyl L-phenylalanyl-L-tryptophanate (21a).** Cbz-L-phenylalanine (2.42 g, 8.1 mmol, 1.0 equiv) and 1-hydroxybenzotriazole (3.31 g, 9.72 mmol, 1.2 equiv) were dissolved in a mixture of dichloromethane (75 mL) and *N*, *N*-dimethylformamide (3.5 mL) under nitrogen atmosphere and subsequently cooled to 0 °C. 1-Ethyl-3- (3-dimethylaminopropyl) carbodiimide hydrochloride (1.86 g, 9.72 mmol, 1.2 equiv) was added at 0 °C and the reaction mixture was stirred for one hour. Then L-tryptophan-*tert*-butyl ester (2.23 g, 8.1 mmol, 1.0 equiv) was added followed by addition of N-methyl morpholine (1.64 g, 16.2 mmol, 2.0 equiv) and the stirring was continued for 12 hours at room temperature. After the completion of the reaction, the mixture obtained was washed twice with 10% $NaHCO_3$ aqueous and 1 M HCl aqueous respectively. The combined organic layer was washed with brine and dried over anhydrous $Na_2SO_4$. The crude product was purified by column chromatography to afford **21a-I**. **TLC:** $R_f$= 0.4 (eluent: DCM/MeOH = 30/1, visualized by UV light). **$^1$H NMR** (400 MHz, DMSO-*d*$_6$) δ 10.9 (s, 1H), 8.40 (d, *J* = 7.3 Hz, 1H), 7.55 (d, *J* = 7.8 Hz, 1H), 7.48 (d, *J* = 8.8 Hz, 1H), 7.37 – 7.17 (m, 11H), 7.08 (t, *J* = 7.4 Hz, 1H), 7.00 (t, *J* = 7.4 Hz, 1H), 4.93 (s, 2H), 4.45 (q, *J* = 7.2 Hz, 1H), 4.37 – 4.27 (m, 1H), 3.21 – 2.94 (m, 3H), 2.76 – 2.70 (m, 1H), 1.28 (s, 9H). **$^{13}$C NMR** (101 MHz, DMSO-*d*$_6$) δ 172.1, 171.3, 156.3, 138.5, 136.6,

129.7, 128.7, 128.5, 128.1, 127.9, 127.7, 126.7, 124.1, 121.4, 118.8, 118.7, 111.8, 110.0, 80.9, 65.7, 56.4, 54.2, 38.0, 28.0, 27.5.

To a stirred solution of compound **21a-I** (3.97 g, 7.1 mmol) in methanol (41 mL) was added 10% Pd/C (0.55 g). The mixture was stirred under hydrogen atmosphere at room temperature overnight after which no starting material could be detected by TLC analysis. The catalyst was removed by filtration through a pad of celite an washed with methanol several times. The filtrate was concentrated under reduced pressure to give the crude product. The crude product was purified by column chromatography to afford 2.80 g of **19a**（98% yield).

**TLC:** $R_f$ = 0.2 (eluent: DCM/MeOH = 30/1, visualized by UV light).

**$^1$H NMR** (400 MHz, DMSO-$d_6$) δ 10.86 (s, 1H), 8.16 (d, *J* = 8.0 Hz, 1H), 7.46 (d, *J* = 7.9 Hz, 1H), 7.33 (d, *J* = 8.1 Hz, 1H), 7.27 – 7.23 (m, 2H), 7.20 – 7.14 (m, 3H), 7.10 – 7.04 (m, 2H), 6.97 (t, *J* = 7.4 Hz, 1H), 4.51 (q, *J* = 7.0 Hz, 1H), 3.43 –3.40 (m, 1H), 3.07 (d, *J* = 6.7 Hz, 2H), 2.94 – 2.90 (m, 1H), 2.59 – 2.54 (m, 1H), 1.61 (s, 2H), 1.30 (s, 9H).

**$^{13}$C NMR** (101 MHz, DMSO-$d_6$) δ 174.5, 171.3, 139.0, 136.5, 129.8, 128.5, 126.6, 124.1, 121.4, 118.8, 111.8, 109.8, 81.1, 56.2, 53.6, 41.2, 28.0, 27.9.

**HRMS** m/z (ESI) calcd. for $C_{24}H_{30}N_3O_3^+$ (M + H$^+$) 408.2282, found 408.2281.

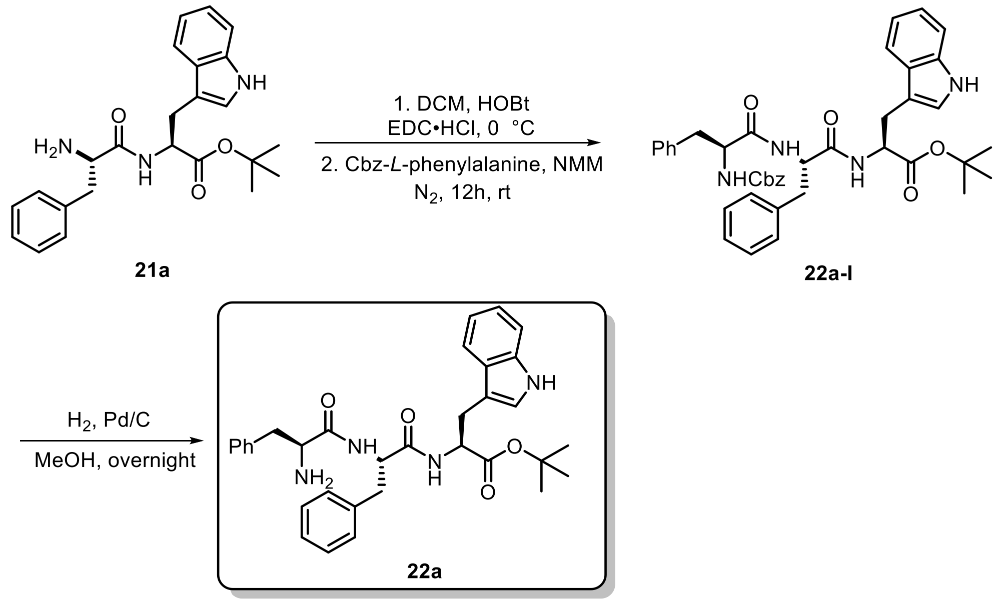


***Tert*-butyl L-phenylalanylphenylalanyl-L-tryptophanate. (22a)**: Cbz-L-

phenylalanine (1.20 g, 4.0 mmol, 1.0 equiv) and 1-hydroxybenzotriazole (0.63 g, 4.80 mmol, 1.2 equiv) were dissolved in a mixture of dichloromethane (26 mL) and *N, N*-dimethylformamide (2.1 mL) under nitrogen atmosphere and subsequently cooled to 0 °C. 1-ethyl-3- (3-dimethylaminopropyl) carbodiimide hydrochloride (0.92 g, 4.80 mmol, 1.2 equiv) was added at 0 °C and the reaction mixture was stirred for one hour. Then **21a** (1.63 g, 4.0 mmol, 1.0 equiv) was added followed by addition of N-methyl morpholine (8.0 mmol) and the stirring was continued for 12 hours at room temperature. After the completion of the reaction, the reaction mixture was washed twice with 10% $NaHCO_3$ aqueous and 1 M HCl aqueous respectively. The combined organic layer was washed with brine and dried over anhydrous $Na_2SO_4$. The crude compound was used in next step directly without purification. **TLC:** $R_f$= 0.3 (eluent: DCM/MeOH = 30/1, visualized by UV light).

To a stirred solution of crude compound (2.40 g) in methanol (21 mL) was added 10% Pd/C (0.30 g). The mixture was stirred under hydrogen atmosphere at room temperature overnight after which no starting material could be detected by TLC analysis. The catalyst was removed by filtration through a pad of celite an washed with methanol several times. The filtrate was concentrated under reduced pressure to give the crude product. The crude product was purified by column chromatography using 10% (v/v) MeOH/DCM as eluent to afford 1.88 g of **22a** (97% yield).

**TLC:** $R_f$= 0.2 (eluent: DCM/MeOH = 5/1, visualized by UV light).

**$^1$H NMR** (400 MHz, DMSO-$d_6$) δ 10.85 (s, 1H), 8.44 (d, $J$ = 7.6 Hz, 1H), 7.99 (d, $J$ = 8.7 Hz, 1H), 7.53 (d, $J$ = 7.8 Hz, 1H), 7.34 (d, $J$ = 8.0 Hz, 1H), 7.26 – 7.12 (m, 11H), 7.07 (t, $J$ = 7.0 Hz, 1H), 6.99 (t, $J$ = 7.4 Hz, 1H), 4.68 – 4.63 (m, 1H), 4.55 – 4.40 (m, 1H), 3.38 – 3.32 (m, 1H), 3.20 – 2.97 (m, 3H), 2.85 – 2.79 (m, 2H), 2.48 – 2.43 (m, 1H), 1.59 (s, 2H), 1.31 (s, 9H).

**$^{13}$C NMR** (101 MHz, DMSO-$d_6$) δ 174.2, 171.3, 137.8, 136.6, 129.9, 129.7, 128.6, 128.3, 126.7, 126.5, 124.1, 121.4, 118.8, 118.6, 111.8, 110.0, 81.0, 56.5, 54.1, 53.2, 41.0, 38.5, 28.0, 27.7.

**HRMS** m/z (ESI) calcd. for $C_{33}H_{39}N_4O_4^+$ (M + H$^+$) 555.2966, found 555.2967.

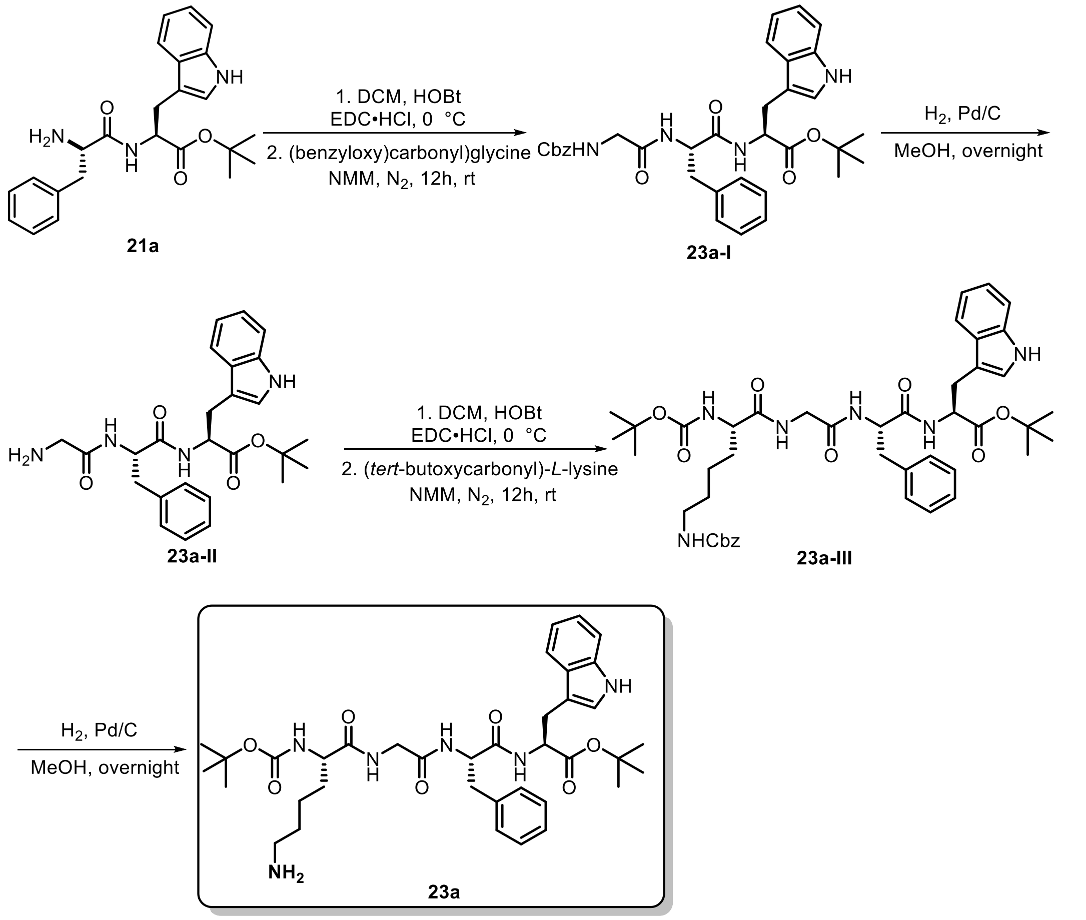


***Tert*-butyl (*tert*-butoxycarbonyl)-L-lysylglycyl-L-phenylalanyl-L-tryptophanate (23a).** (Benzyloxy)carbonyl)glycine (5.73 g, 14.0 mmol, 1.0 equiv) and 1-hydroxybenzotriazole (2.22 g, 16.8 mmol, 1.2 equiv) were dissolved in the mixture of dichloromethane (91 mL) and *N, N*-dimethylformamide (7.35 mL) under nitrogen atmosphere and subsequently cooled to 0 °C. 1-ethyl-3- (3-dimethylaminopropyl) carbodiimide hydrochloride (3.22 g, 16.8 mmol, 1.2 equiv) was added at 0 °C and the reaction mixture was stirred for one hour. Then **21a** (5.72 g, 14.0 mmol, 1.0 equiv) was added followed by addition of N-methyl morpholine (2.83 mL, 28.0 mmol, 2.0 equiv) and the stirring was continued for 12 hours at room temperature. After the completion of the reaction, the reaction mixture was washed twice with 10% $NaHCO_3$ aqueous and 1 M HCl aqueous respectively. The combined organic layer was washed with brine and dried over anhydrous $Na_2SO_4$. The crude product was purified by column chromatography to afford *tert*-butyl ((benzyloxy)carbonyl)glycyl-L-phenylalanyl-L-tryptophanate (**23a-I)** (6.12 g, 73% yield). **TLC:** $R_f$= 0.3 (eluent: DCM: MeOH = 10:1,

visualized by UV light). **$^1$H NMR** (400 MHz, DMSO-*$d_6$*) 10.84 (s, 1H), 8.45 (d, *J* = 7.4 Hz, 1H), 7.96 (d, *J* = 8.6 Hz, 1H), 7.53 (d, *J* = 7.8 Hz, 1H), 7.44 – 7.13 (m, 12H), 7.07 (t, *J* = 7.2 Hz, 1H), 6.99 (t, *J* = 7.2 Hz, 1H), 5.02 (s, 2H), 4.63 – 4.58 (m, 1H), 4.45 (q, *J* = 7.3 Hz, 1H), 3.67 – 3.45 (m, 2H), 3.22 – 2.97 (m, 3H), 2.89 (s, 1H), 2.80 – 2.73 (m, 1H), 1.28 (s, 9H). **$^{13}$C NMR** (101 MHz, DMSO-*$d_6$*) δ 171.4, 171.2, 156.9, 138.0, 136.6, 129.7, 128.8, 128.4, 128.2, 128.1, 126.7, 124.1, 121.4, 118.8, 118.6, 111.8, 109.9, 80.9, 65.9, 54.2, 53.8, 43.8, 38.2, 28.0, 27.6.

To a stirred solution of the compound **23a-I** (2.40 g, 3.3 mmol) in methanol (20 mL) was added 10% Pd/C (0.20 g). The mixture was stirred under hydrogen atmosphere at room temperature overnight after which no starting material could be detected by TLC analysis. The catalyst was removed by filtration through a pad of celite an washed with methanol several times. The filtrate was concentrated under reduced pressure and purified by column chromatography to afford **23a-II** (1.69 g, 91% yield). **TLC:** $R_f$ = 0.3 (eluent: DCM/MeOH = 5/1, visualized by UV light). **$^1$H NMR** (400 MHz, DMSO-*$d_6$*) δ 10.87 (d, *J* = 2.4 Hz, 1H), 8.46 (d, *J* = 7.6 Hz, 1H), 7.97 (s, 1H), 7.54 (d, *J* = 7.8 Hz, 1H), 7.35 (d, *J* = 8.0 Hz, 1H), 7.27 – 7.14 (m, 6H), 7.08 (t, *J* = 7.5 Hz, 1H), 7.00 (t, *J* = 7.4 Hz, 1H), 4.63 (s, 1H), 4.49 – 4.43 (m, 1H), 3.19 – 3.01 (m, 4H), 2.96 – 2.68 (m, 2H), 1.82 (s, 2H), 1.31 (s, 9H). **$^{13}$C NMR** (101 MHz, DMSO-*$d_6$*) δ 172.9, 171.4, 171.3, 137.9, 129.7, 128.4, 126.7, 124.1, 121.4, 118.8, 118.6, 111.8, 109.9, 81.0, 54.1, 53.3, 44.9, 38.5, 28.0, 27.6.

(*Tert*-butoxycarbonyl)-L-lysine (1.14 g, 3.0 mmol, 1.0 equiv) and 1-hydroxybenzotriazole (0.49 g, 3.6 mmol, 1.2 equiv) were dissolved in a mixture of dichloromethane (25.0 mL) and *N, N*-dimethylformamide (2.0 mL) under nitrogen atmosphere and subsequently cooled to 0 °C. 1-ethyl-3- (3-dimethylaminopropyl) carbodiimide hydrochloride (0.69 g, 3.6 mmol, 1.2 equiv) was added at 0 °C and the reaction mixture was stirred for one hour. Then *tert*-butyl glycyl-L-phenylalanyl-L-tryptophanate (1.39 g, 3.0 mmol, 1.0 equiv) was added followed by addition of N-methyl morpholine (0.84 mL, 7.68 mmol, 2.0 equiv) and the stirring was continued for 12 hours at room temperature. After the completion of the reaction, the mixture obtained was washed twice with 10% $NaHCO_3$ aqueous and 1 M HCl aqueous respectively. The

combined organic layer was washed with brine and dried over anhydrous $Na_2SO_4$. The crude compound was used in next step directly without purification. **TLC:** $R_f$ = 0.2 (eluent: DCM/MeOH = 10/1, visualized by UV light).

To a stirred solution of crude compound (2.47 g) in methanol (20 mL) was added 10% Pd/C (0.20 g). The mixture was stirred under hydrogen atmosphere at room temperature overnight after which no starting material could be detected by TLC analysis. The catalyst was removed by filtration through a pad of celite an washed with methanol several times. The filtrate was concentrated under reduced pressure. The crude product was purified by column chromatography to afford 1.81g of **23a** (87% yield).

**TLC:** $R_f$= 0.2 (eluent: DCM/MeOH = 5/1, visualized by UV light).

**$^1$H NMR** (400 MHz, DMSO-$d_6$) δ 10.94 (d, $J$ = 2.4 Hz, 1H),8.50 (d, $J$ = 7.4 Hz, 1H), 8.10 (d, $J$ = 8.5 Hz, 1H), 8.00 (s, 3H), 7.53 (d, $J$ = 7.8 Hz, 1H), 7.39 – 7.31 (m, 1H), 7.27 – 7.12 (m, 6H), 7.07 (t, $J$ = 7.5 Hz, 1H), 6.99 (t, $J$ = 7.5 Hz, 1H), 6.92 (d, $J$ = 7.9 Hz, 1H), 4.61 – 4.58 (m, 1H), 4.49 – 4.36 (m, 1H), 3.92 – 3.87 (m, 1H), 3.71 – 3.57 (m, 2H), 3.17 – 3.01 (m, 3H), 2.77 – 2.70 (m, 3H), 1.59 – 1.46 (m, 4H), 1.37 (s, 9H), 1.29 (s, 9H), 1.25 – 1.18 (m, 2H).

**$^{13}$C NMR** (101 MHz, DMSO-$d_6$) δ 172.7, 171.5, 171.2, 168.8, 155.8, 138.1, 136.6, 129.7, 128.4, 127.6, 126.7, 124.2, 121.4, 118.8, 118.6, 111.9, 109.9, 80.9, 78.6, 54.5, 54.2, 54.0, 45.8, 42.3, 38.9, 38.3, 31.7, 28.8, 28.0, 27.6, 27.0, 22.8, 8.9.

**HRMS** m/z (ESI) calcd. for $C_{37}H_{53}N_6O_7^+$ (M + $H^+$) 693.3970, found 693.3980.

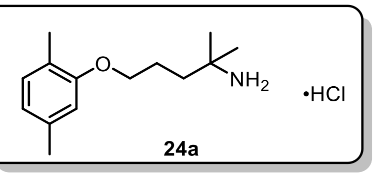

**24a**

**5-(2,5-dimethylphenoxy)-2-methylpentan-2-amine hydrochloride (24a).** Prepared from oxaprozin with the same method used to synthesize 2-(4,5-diphenyloxazol-2-yl)ethan-1-amine hydrochloride (**1a**).The white solid obtained was washed with DCM, ether and dried *in vacuo* to produce 1.06 g of compound **24a** (82% yield).

**$^{1}$H NMR** (400 MHz, DMSO-$d_6$) δ 8.10 (s, 3H), 6.99 (d, *J* = 7.5 Hz, 1H), 6.73 (s, 1H), 6.63 (d, *J* = 7.4 Hz, 1H), 3.93 (t, *J* = 5.6 Hz, 2H), 2.25 (s, 3H), 2.10 (s, 3H), 1.83 – 1.65 (m, 4H), 1.26 (s, 6H).

**$^{13}$C NMR** (101 MHz, DMSO-$d_6$) δ 156.9, 136.6, 130.6, 122.9, 121.1, 112.6, 67.7, 53.8, 36.8, 25.4, 23.9, 21.6, 16.1.

**HRMS** m/z (ESI) calcd. for $C_{14}H_{24}NO^+$ (M + H$^+$) 222.1852, found 222.1853.

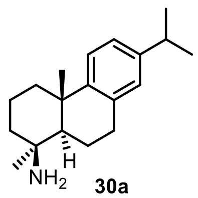


**30a**

**(1S,4aS,10aR)-7-isopropyl-1,4a-dimethyl-1,2,3,4,4a,9,10,10a-octahydrophenanthren-1-amine (30a).** Prepared from dehydroabietlc acid with the same method used to synthesize 2-(4,5-diphenyloxazol-2-yl)ethan-1-amine hydrochloride (**1a**). The crude product obtained was dissolved in 10 mL of 10% $K_2CO_3$ aqueous. Then the aqueous was extracted with EA three times. The organic layer was dried over anhydrous $Na_2SO_4$, dried *in vacuo* and purified by column chromatography to produce 0.48 g of compound **30a** (35% yield).

**TLC:** $R_f$= 0.2 (eluent: DCM/MeOH = 5/1, visualized by $I_2$ chamber).

**$^{1}$H NMR** (400 MHz, Chloroform-*d*) δ 7.20 (d, *J* = 8.2 Hz, 1H), 7.03 – 7.01 (m, 1H), 6.92 (s, 1H), 3.04 – 2.79 (m, 3H), 2.31 – 2.26 (m, 1H), 2.11 – 2.04 (m, 1H), 1.80 – 1.67 (m, 6H), 1.47 – 1.41 (m, 2H), 1.33 – 1.23 (m, 7H), 1.20 (s, 3H), 1.12 (s, 3H).

**$^{13}$C NMR** (101 MHz, Chloroform-*d*) δ 146.8, 145.6, 134.7, 126.9, 124.4, 123.9, 53.1, 51.8, 43.4, 38.4, 38.1, 33.5, 30.5, 24.7, 24.0, 24.0, 22.7, 20.2, 18.3.

**HRMS** m/z (ESI) calcd. for $C_{19}H_{30}N^+$ (M + H$^+$) 272.2373, found 272.2369.

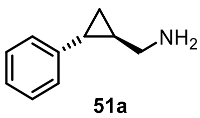


**51a**

To a solution of (1R,2R)-2-phenylcyclopropane-1-carboxylic acid (1.6 g, 10 mmol)

in dichloromethane (50 mL) was added 2-3 drops of *N, N*-dimethylformamide and oxalyl chloride (12.1 mL). The reaction was stirred at room temperature for 3 h. The mixture was then cooled down to 0 °C and a solution of 28% v/v aqueous ammonium hydroxide (5 mL) in acetonitrile (50 mL) was added. The reaction mixture was then stirred at room temperature for 16 h. The reaction mixture was diluted with EA (30 mL) and washed sequentially with $H_2O$ and brine. The organic layer was dried with anhydrous $Na_2SO_4$, filtered and concentrated *in vacuo* to yield the product as white solid (1.6 g, quant.). **TLC:** $R_f$= 0.3 (eluent: DCM/MeOH = 10/1, visualized by PMA stain).

2-Phenylcyclopropanecarboxamide (1.60 g, 8.0 mmol, 1.0 equiv.) and $LiAlH_4$ (1.22 g, 32 mmol, 4.0 equiv.) were added to 200 mL of anhydrous THF under argon protection. The mixture was heated to reflux for 24 h and then allowed to cool down to room temperature. After the reaction was carefully quenched with a small amount of ice water, the mixture was filtered through a short pad of silica gel and the filter cake was rinsed with THF. Removed THF under vacuum and extracted the mixture with EA. The obtained organic phase was concentrated under vacuum and the crude product was purified by silica gel chromatography column to give 1.02 g of product **51a** (90% yield). **TLC:** $R_f$=0.2 (eluent: DCM/MeOH = 10/1, visualized by $I_2$ chamber).

**$^1$H NMR** (400 MHz, DMSO-*d*$_6$) δ 7.27 – 7.23 (m, 2H), 7.16 – 7.10 (m, 3H), 6.45 (s, 2H), 2.87 – 2.71 (m, 2H), 2.02 – 1.97 (m, 1H), 1.33 – 1.30 (m, 1H), 1.06 – 0.93 (m, 2H).

**$^{13}$C NMR** (101 MHz, DMSO-*d*$_6$) δ 142.6, 128.6, 126.3, 126.0, 43.5, 22.0, 21.6, 14.7.

**HRMS** m/z (ESI) calcd. for $C_{10}H_{14}N^+$ (M + $H^+$) 148.1121, found 148.1122.

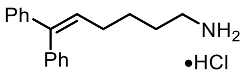


52a

**6,6-diphenylhex-5-en-1-amine hydrochloride (52a).** Synthesized according to the literature[3, 4].

**$^1$H NMR** (400 MHz, DMSO-*d*$_6$) δ 8.14 (s, 3H), 7.48 – 7.07 (m, 10H), 6.11 (t, *J* = 7.4 Hz, 1H), 2.69 (t, *J* = 7.2 Hz, 2H), 2.04 (q, *J* = 7.2 Hz, 2H), 1.65 – 1.40 (m, 4H).

$^{13}$**C NMR** (101 MHz, DMSO-$d_6$) δ 142.4, 141.8, 140.0, 129.9, 129.5, 128.9, 128.7, 127.6, 127.5, 127.2, 39.1, 29.2, 27.0, 26.6.

**HRMS** m/z (ESI) calcd. for $C_{18}H_{23}ClN^+$ (M + $H^+$) 252.1747, found 252.1757.

# 3. Optimization of the Reaction Conditions

## Figure S1: Screening of Hydrodeaminatin Conditions of α-Tertiary Amine 28a

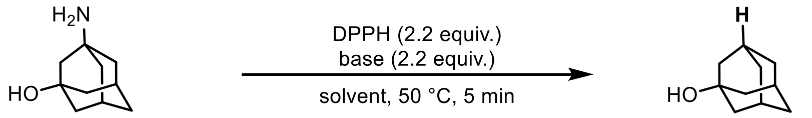


**28a**, 0.1 mmol  **28b**

| entry | base | solvent | temperature (°C) | yield (%)[a] |
|---|---|---|---|---|
| 1 | $K_2CO_3$ | THF (0.1 M) | 50 | 38 |
| 2 | $K_2CO_3$ | DMAc (0.1 M) | 50 | 41 |
| 3 | $K_2CO_3$ | $CH_3CN$ (0.1 M) | 50 | 20 |
| 4 | $K_2CO_3$ | 1,4-dioxane (0.1 M) | 50 | 47 |
| **5** | **$K_2CO_3$** | **$H_2O$/THF (v:v = 1/1) (0.1 M)** | **50** | **89 (87)[b]** |
| 6 | $K_2CO_3$ | $H_2O$/1,4-dioxane (v:v = 1/1) (0.1 M) | 50 | 25 |
| 7 | $K_2CO_3$ | $H_2O/CH_3CN$ (v:v = 1/1) (0.1 M) | 50 | 70 |
| 8 | $K_2CO_3$ | $H_2O$/DMF (v:v = 1/1) (0.1 M) | 50 | 42 |
| 9 | $K_2CO_3$ | $H_2O$/DMAc (v:v = 1/1) (0.1 M) | 50 | 77 |
| 10 | $Cs_2CO_3$ | THF (0.1 M) | 50 | 69 |
| 11 | DIPEA | THF (0.1 M) | 50 | 23 |
| 12 | MeOK | THF (0.1 M) | 50 | 54 |
| 13 | PhOK | THF (0.1 M) | 50 | 57 |
| 14 | DBU | THF (0.1 M) | 50 | 61 |

[a] A screw capped vial with a stirring bar was charged with the amine (0.1 mmol, 1.0 equiv), the base, the solvent under air, and finally, the hydroxylamine was added last. The mixture was stirred at the chosen temperature for 5 min. The reaction was diluted by 1 mL of NaCl aq. and 1 mL of EA. Dodecane (0.1 mmol) was added as the internal standard. 5 uL of the upper organic phase was taken and diluted to 1.5 mL with EA. The yield was detected using a Shimadzu GCMS-QP2010 Ultra with an SH-I-5Sil MS capillary column. The yields were calibrated with a curve obtained from authentic commercially available products. [b] isolated yield.

## Figure S2: Screening of Hydrodeaminatin Conditions of α-Secondary Amine 41a

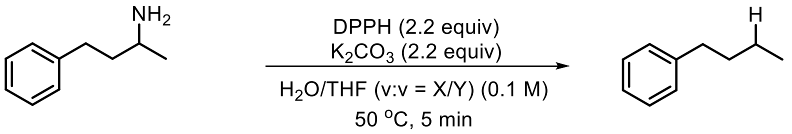


**41a**, 0.1 mmol — **41b**

| entry | base | solvent | temperature (°C) | yield (%)[a] |
|---|---|---|---|---|
| 1 | $K_2CO_3$ | THF (0.1 M) | 50 | 37 |
| 2 | $K_2CO_3$ | $CH_3CN$ (0.1 M) | 50 | 34 |
| 3 | $K_2CO_3$ | DMF (0.1 M) | 50 | 45 |
| 4 | $K_2CO_3$ | 1,4-dioxane (0.1 M) | 50 | 51 |
| **5** | **$K_2CO_3$** | **$H_2O$/THF (v:v = 1/1) (0.1 M)** | **50** | **99** |
| 6 | $K_2CO_3$ | $H_2O$/1,4-dioxane (v:v = 1/1) (0.1 M) | 50 | 76 |
| 7 | $K_2CO_3$ | $H_2O/CH_3CN$ (v:v = 1/1) (0.1 M) | 50 | 76 |
| 8 | $K_2CO_3$ | $H_2O$/DMF (v:v = 1/1) (0.1 M) | 50 | 84 |
| 9 | $K_2CO_3$ | $H_2O/CDCl_3$ (v:v = 1/1) (0.1 M) | 50 | 63 |
| 10 | $K_2CO_3$ | $H_2O$ (0.1 M) | 50 | 59 |
| 11 | $Cs_2CO_3$ | THF (0.1 M) | 50 | 87 |
| 12 | $Na_2CO_3$ | THF (0.1 M) | 50 | 91 |
| 13 | KOAc | THF (0.1 M) | 50 | 44 |
| 14 | LiO*t*-Bu | THF (0.1 M) | 50 | 24 |
| 15 | DIPEA | THF (0.1 M) | 50 | 56 |
| 16 | DMAP | THF (0.1 M) | 50 | 62 |
| 17 | $K_2CO_3$ | $H_2O$/THF (v:v = 1/1) (0.1 M) | 50 | 62[b] |
| 18 | $K_2CO_3$ | $H_2O$/THF (v:v = 1/1) (0.1 M) | 50 | 11[c] |
| 19 | $K_2CO_3$ | $H_2O$/THF (v:v = 1/1) (0.1 M) | 50 | NP[d] |
| 20 | $K_2CO_3$ | $H_2O$/THF (v:v = 1/1) (0.1 M) | 50 | trace[e] |

[a] Determined by GC-MS, dodecane as internal standard. [b] DPPH (1.2 equiv). [c] O-(2,4-dinitrophenyl) hydroxylamine instead of DPPH. [d] (aminooxy)sulfonic acid instead of DPPH. [e] O-tosylhydroxylamine instead of DPPH.

# Figure S3: Screening of Deuterodeaminatin Conditions of α-Secondary Amine 41a

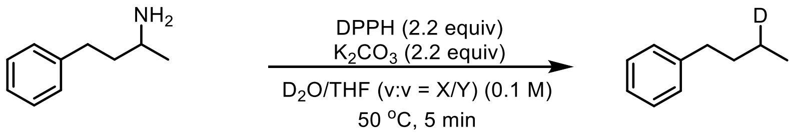


| entry | $D_2O$/THF (v:v = X/Y) | GC-MS yield %[a] | D (%)[b] |
|---|---|---|---|
| 1 | 1:1 | > 99% | 95%D |
| 2 | 1:3 | > 99% | 90%D |
| 3 | 1:5 | > 99% | 87%D |
| 4 | 1:7 | > 99% | 84%D |
| **5** | **5:1** | > **99%** | **97%D** |
| 6 | 3:1 | > 99% | 96%D |
| 7 | 5:1[c] | 98% | 96%D |

[a]Determined by GC-MS, dodecane as internal standard. [b] Deuteration rate measured by GC-MS.[c] $d_8$-THF instead of THF.

## Figure S4: Screening of Hydrodeaminatin Conditions of Aryl Amine 31a

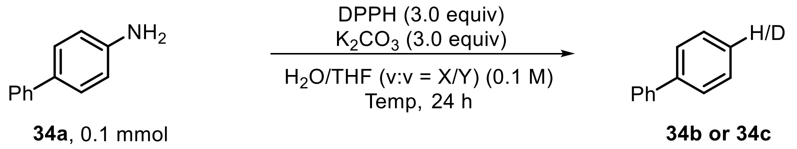


| entry | base | solvent | temperature (°C) | yield (%)[a] |
|---|---|---|---|---|
| 1 | $K_2CO_3$ | THF (0.1 M) | 60 | 31 |
| 2 | $Cs_2CO_3$ | $H_2O$/THF (v:v = 5/1) (0.1 M) | 60 | 53 |
| 3 | NaOAc | $H_2O$/THF (v:v = 5/1) (0.1 M) | 60 | 9 |
| 4 | DBU | $H_2O$/THF (v:v = 5/1) (0.1 M) | 60 | 32 |
| 5 | DIPEA | $H_2O$/THF (v:v = 5/1) (0.1 M) | 60 | 52 |
| 6 | $K_2CO_3$ | $H_2O$/THF (v:v = 5/1) (0.1 M) | 80 | 43 |
| 7 | $K_2CO_3$ | $H_2O$/2-MeTHF (v:v = 1/1) (0.1 M) | 80 | 50 |
| 8 | $K_2CO_3$ | $H_2O$/CPME (v:v = 1/1) (0.1 M) | 80 | 43 |
| **9** | **$K_2CO_3$** | **THF (0.1 M)** | **80** | **74[b](60)[d]** |
| 10 | $K_2CO_3$ | $D_2O$/THF (v:v = 1/1) (0.1 M) | 60 | 46(32%D)[c] |
| 11 | $K_2CO_3$ | $D_2O$/THF (v:v = 5/1) (0.1 M) | 60 | 51(60%D)[c] |
| **12** | **$K_2CO_3$** | **$D_2O$/THF (v:v = 5/1) (0.1 M)** | **80** | **52(45[d],59%D)[c]** |
| 13 | $K_2CO_3$ | $D_2O$/THF (v:v = 5/1) (0.1 M) | 100 | 52(59%D)[c] |
| 14 | $K_2CO_3$ | $D_2O$/$d_8$-THF (v:v = 5/1) (0.1 M) | 80 | 57(69%D) |

[a] Determined by GC-MS, dodecane as internal standard. [b] 18-crown-6 (40 mmol%). [c]The deuterium incorporation was determined by GCMS.[d] isolated.

## 4. General Procedure for Hydro- and Deuterodeaminatin of Primary Amines.

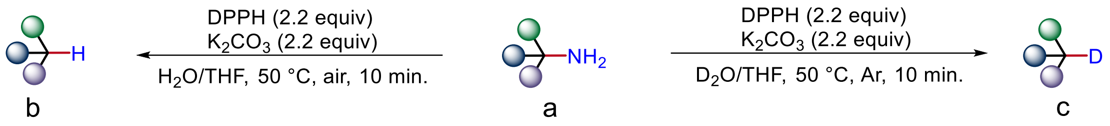


To a 25 mL Schlenk tube with a stirring bar, amine (or amine•HCl salt, 0.4 mmol), THF (2 mL, 3.3 mL for deuteration), $H_2O$ (2 mL, 3.3 mL of $D_2O$ for deuteration), $K_2CO_3$ (121.6 mg, 2.2 equiv; 176.9 mg, 3.2 equiv if using amine•HCl salt, maleate salt or $H_3PO_3$ salt), DPPH (*O*-(Diphenylphosphinyl) hydroxylamine, 205.2 mg, 2.2 equiv) was added orderly. The Schlenk tube was sealed up (under argon for deuteration), placed on a heating module preheated to 50 °C, and kept stirring at the same temperature vigorously (800 rpm) for 10 min. The reaction was cooled to room temperature, diluted with 5 mL of NaCl aq., extracted three times with 5 mL of EA. The organic layers were combined, dried over anhydrous $Na_2SO_4$, and purified with flash chromatography over silica gel to afford the desired product.

# 5. Characterization Data of Products

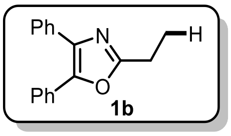

**2-ethyl-4,5-diphenyloxazole (1b).** Prepared according to general procedure from 0.4 mmol of 2-(4,5-diphenyloxazol-2-yl)ethan-1-amine hydrochloride (**1a**), 83.8 mg of **1b** was obtained (84% yield).

**TLC**: $R_f$ = 0.40 (eluent: PE/EA = 5/1, visualized by UV light).

**$^1$H NMR** (400 MHz, Chloroform-*d*) δ 7.77 – 7.58 (m, 4H), 7.49 – 7.25 (m, 6H), 2.93 (q, *J* = 7.6 Hz, 2H), 1.47 (t, *J* = 7.6 Hz, 3H).

**$^{13}$C NMR** (101 MHz, Chloroform-*d*) δ 164.5, 145.1, 132.7, 129.2, 128.6, 128.6, 128.4, 128.3, 128.0, 128.0 126.4, 21.8, 11.4.

**HRMS** m/z (ESI) calcd. for $C_{17}H_{16}NO^+$ (M + H$^+$) 250.1226, found 250.1229.

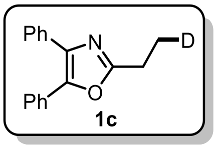

**2-(ethyl-2-d)-4,5-diphenyloxazole (1c).** Prepared according to general procedure from 0.4 mmol of 2-(4,5-diphenyloxazol-2-yl)ethan-1-amine hydrochloride (**1a**), 65.1 mg of **1c** was obtained (65% yield, 91%D). The deuterium incorporation was determined by ESI-HRMS.

**TLC**: $R_f$ = 0.40 (eluent: PE/EA = 5/1, visualized by UV light).

**$^1$H NMR** (400 MHz, Chloroform-*d*) δ 7.69 – 7.63 (m, 2H), 7.62 – 7.57 (m, 2H), 7.40 – 7.28 (m, 6H), 2.90 – 2.86 (m, 2H), 1.46 – 1.39 (m, 2H).

**$^{13}$C NMR** (101 MHz, Chloroform-*d*) δ 164.5, 145.1, 135.1, 132.7, 129.2, 128.6, 128.5, 128.3, 128.0, 126.4, 21.7, 11.1 (t, *J* = 19.9 Hz).

**HRMS** m/z (ESI) calcd. for $C_{17}H_{14}DNNaO^+$ (M + Na$^+$) 273.1109, found 273.1102.

**ESI-HRMS of 1c:**

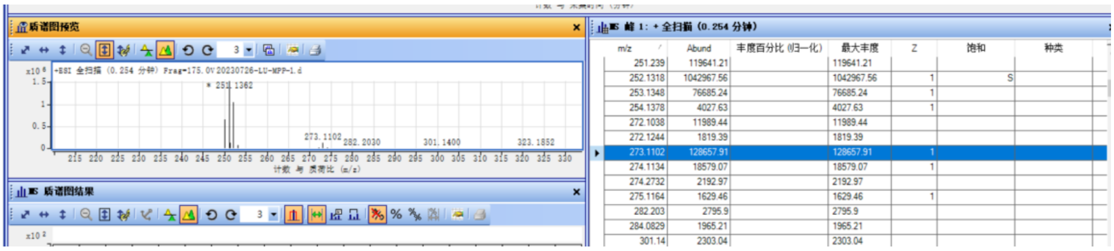


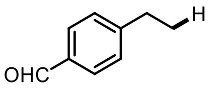


**2b**

**4-ethylbenzaldehyde (2b).** Prepared according to procedure from 0.4 mmol of 4-(2-aminoethyl)benzaldehyde hydrochloride (**2a**), 30.1 mg of **2b** was obtained (56% yield). The deuterium incorporation was determined by GC-MS.

**TLC**: $R_f$ = 0.50 (eluent: PE/EA = 10/1, visualized by UV light).

**$^1$H NMR** (400 MHz, Chloroform-*d*) δ 9.99 (s, 1H), 7.82 (d, *J* = 8.2 Hz, 2H), 7.38 (d, *J* = 8.0 Hz, 2H), 2.75 (q, *J* = 7.6 Hz, 2H), 1.30 (d, *J* = 7.6 Hz, 3H).

**$^{13}$C NMR** (101 MHz, Chloroform-*d*) δ 191.9, 151.6, 134.4, 129.9, 128.5, 29.1, 15.1.

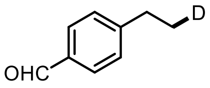


**2c**

**4-(ethyl-2-d)benzaldehyde (2c).** Prepared according to procedure from 0.4 mmol of 4-(2-aminoethyl)benzaldehyde hydrochloride (**2a**), 23.3 mg of **2c** was obtained (43% yield, 47%D). The deuterium incorporation was determined by GC-MS.

**TLC**: $R_f$ = 0.50 (eluent: PE/EA = 10/1, visualized by UV light).

**$^1$H NMR** (400 MHz, Chloroform-*d*) δ 9.96 (s, 1H), 7.79 (d, *J* = 8.1 Hz, 2H), 7.34 (d, *J* = 7.9 Hz, 2H), 2.71 (t, *J* = 7.4 Hz, 2H), 1.23 – 1.18 (m, 3H).

**$^{13}$C NMR** (101 MHz, Chloroform-*d*) δ 191.8, 151.6, 134.5, 129.9, 128.5, 29.1, 14.8 (t, *J* = 19.5 Hz).

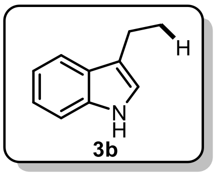

**3b**

**3-ethyl-1H-indole (3b).** Prepared according to general procedure from 0.4 mmol of tryptamine (**3a**), 47.1 mg of **3b** was obtained (84% yield).

**TLC**: $R_f$ = 0.40 (eluent: PE/EA = 5/1, visualized by UV light).

**$^{1}$H NMR** (400 MHz, Chloroform-*d*) δ 7.75 (s, 1H), 7.61 (d, *J* = 7.8 Hz, 1H), 7.30 (d, *J* = 8.0 Hz, 1H), 7.17 (t, *J* = 7.5 Hz, 1H), 7.10 (t, *J* = 7.4 Hz, 1H), 6.91 (s, 1H), 2.78 (q, *J* = 7.5 Hz, 2H), 1.32 (t, *J* = 7.5 Hz, 3H).

**$^{13}$C NMR** (101 MHz, Chloroform-*d*) δ 136.4, 127.5, 121.9, 120.5, 119.1, 119.0, 118.8, 111.1, 18.4, 14.5.

**HRMS** m/z (ESI) calcd. for $C_{10}H_{12}N^+$ (M + H$^+$) 146.0964, found 146.0958.

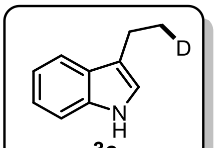

**3c**

**3-(ethyl-2-d)-1H-indole (3c).** Prepared according to general procedure from 0.4 mmol of tryptamine (**3a**), 52.6 mg of **3c** was obtained (90% yield, 95%D). The deuterium incorporation was determined by ESI-HRMS.

**TLC**: $R_f$ = 0.40 (eluent: PE/EA = 5/1, visualized by UV light).

**$^{1}$H NMR** (400 MHz, Chloroform-*d*) δ 7.82 (s, 1H), 7.72 (d, *J* = 7.7 Hz, 1H), 7.40 (d, *J* = 8.1 Hz, 1H), 7.29 (t, *J* = 7.5 Hz, 1H), 7.22 (t, *J* = 7.5 Hz, 1H), 7.00 (s, 1H), 2.88 (t, *J* = 7.0 Hz, 1H), 1.44 – 1.39 (m, 2H).

**$^{13}$C NMR** (101 MHz, Chloroform-*d*) δ 136.5, 127.5, 121.9, 120.5, 119.1, 119.0, 118.9, 111.1, 18.3, 14.2 (t, *J* = 19.5 Hz).

**HRMS** m/z (ESI) calcd. for $C_{10}H_{11}DN^+$ (M + H$^+$) 147.1027, found 147.1021.

**ESI-HRMS of 3c:**

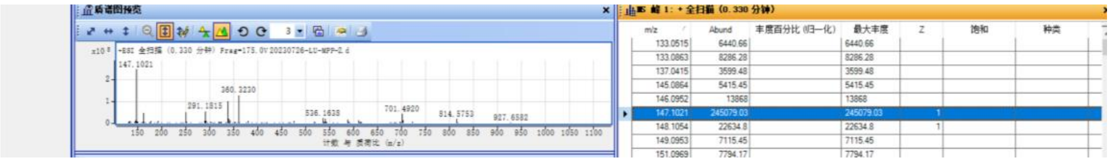

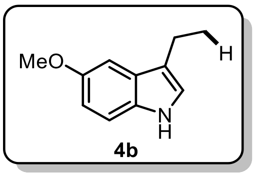

**3-ethyl-5-methoxy-1H-indole (4b).** Prepared according to general procedure from 0.4 mmol of 5-methoxytryptamine (**4a**), 58.2 mg of **4b** was obtained (83% yield).

**TLC**: $R_f$ = 0.40 (eluent: PE/EA = 5/1, visualized by UV light).

**$^1$H NMR** (400 MHz, Chloroform-*d*) δ 7.76 (s, 1H), 7.15 (d, *J* = 8.8 Hz, 1H), 7.04 (d, *J* = 2.6 Hz, 1H), 6.89 – 6.80 (m, 2H), 3.84 (s, 3H), 2.73 (q, *J* = 7.5 Hz, 2H), 1.31 (t, *J* = 7.5 Hz, 3H).

**$^{13}$C NMR** (101 MHz, Chloroform-*d*) δ 153.8, 131.8, 127.9, 121.5, 118.5, 112.0, 111.9, 101.1, 56.1, 18.4, 14.4.

**HRMS** m/z (ESI) calcd. for $C_{11}H_{14}NO^+$ (M + $H^+$) 176.1070, found 176.1062.

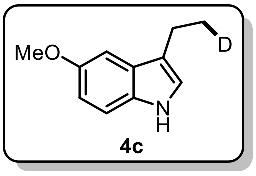

**3-(ethyl-2-d)-5-methoxy-1H-indole (4c).** Prepared according to general procedure from 0.4 mmol of tryptamine (**4a**), 55.0 mg of **4c** was obtained (78% yield, 94%D). The deuterium incorporation was determined by ESI-HRMS.

**TLC**: $R_f$ = 0.40 (eluent: PE/EA = 5/1, visualized by UV light).

**$^1$H NMR** (400 MHz, Chloroform-*d*) δ 7.75 (s, 1H), 7.16 (d, *J* = 8.8 Hz, 1H), 7.04 (d, *J* = 2.4 Hz, 1H), 6.92 – 6.81 (m, 2H), 3.85 (s, 3H), 2.73 (t, *J* = 7.5 Hz, 2H), 1.33 – 1.27 (m, 2H).

**$^{13}$C NMR** (101 MHz, Chloroform-*d*) δ 153.8, 131.7, 127.9, 121.5, 118.5, 112.0, 111.8, 101.0, 56.1, 18.3, 14.1 (t, *J* = 19.4 Hz).

**HRMS** m/z (ESI) calcd. for $C_{11}H_{13}DNO^+$ (M + $H^+$) 177.1133, found 177.1128.

**ESI-HRMS of 4c:**

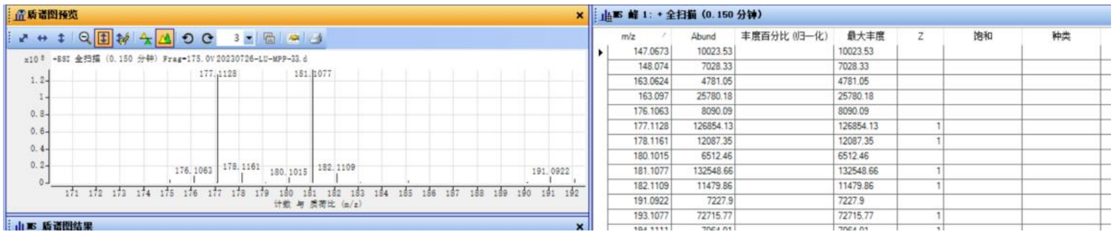


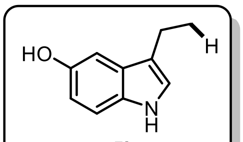


**5b**

**3-ethyl-1H-indol-5-ol (5b).** Prepared according to general procedure from 0.4 mmol of Serotonin hydrochloride (**5a**), 43.8 mg of **5b** was obtained (68% yield).

**TLC**: $R_f$ = 0.50 (eluent: PE/EA = 1/1, visualized by UV light).

**$^1$H NMR** (400 MHz, Chloroform-*d*) δ 7.76 (s, 1H), 7.18 (d, *J* = 8.6 Hz, 1H), 7.00 (d, *J* = 2.4 Hz, 1H), 6.93 (s, 1H), 6.75 (dd, *J* = 8.7, 2.5 Hz, 1H), 4.70 (d, *J* = 12.3 Hz, 1H), 2.70 (q, *J* = 7.5 Hz, 2H), 1.30 (t, *J* = 7.5 Hz, 3H).

**$^{13}$C NMR** (101 MHz, Chloroform-*d*) δ 149.1, 131.8, 128.2, 121.7, 118.3, 111.6, 103.6, 18.3, 14.3.

**HRMS** m/z (ESI) calcd. for $C_{10}H_{12}NO^+$ (M + $H^+$) 162.0913, found 162.0907.

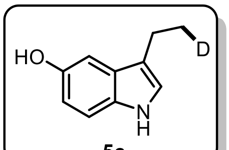


**5c**

**3-(ethyl-2-d)-1H-indol-5-ol (5c).** Prepared according to general procedure from 0.4 mmol of Serotonin hydrochloride (**5a**), 50.6 mg of **5c** was obtained (78% yield, 93%D). The deuterium incorporation was determined by ESI-HRMS.

**TLC**: $R_f$ = 0.50 (eluent: PE/EA = 1/1, visualized by UV light).

$^1$**H NMR** (400 MHz, Chloroform-*d*) δ 7.77 (s, 1H), 7.20 (d, *J* = 8.6 Hz, 1H), 7.02 (d, *J* = 2.4 Hz, 1H), 6.95 (s, 1H), 6.77 (dd, *J* = 8.6, 2.5 Hz, 1H), 4.65 (s, 1H), 2.72 (t, *J* = 7.5 Hz, 2H), 1.32 – 1.27 (m, 2H).

$^{13}$**C NMR** (101 MHz, Chloroform-*d*) δ 149.1, 131.8, 128.2, 121.7, 118.3, 111.6, 103.6, 18.3, 14.2, 14.0 (t, *J* = 19.5 Hz).

**HRMS** m/z (ESI) calcd. for $C_{10}H_{11}DNO^+$ (M + H$^+$) 163.0976, found 163.0971.

**ESI-HRMS of 5c:**

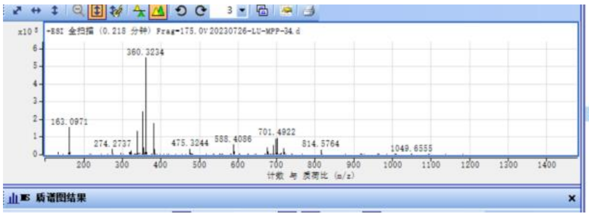


| m/z | Abund | 丰度百分比 (归一化) | 最大丰度 | Z | 饱和 | 种类 |
|---|---|---|---|---|---|---|
| 133.0517 | 12333.95 | | 12333.95 | | | |
| 134.0591 | 6192.39 | | 6192.39 | | | |
| 146.0595 | 3703 | | 3703 | | | |
| 160.075 | 9714.34 | | 9714.34 | | | |
| 161.081 | 9050.45 | | 9050.45 | | | |
| 162.0904 | 11735.05 | | 11735.05 | | | |
| 163.0971 | 154670.52 | | 154670.52 | 1 | | |
| 164.1004 | 13161.43 | | 13161.43 | 1 | | |
| 218.2107 | 5705.8 | | 5705.8 | | | |
| 246.2423 | 4697.23 | | 4697.23 | | | |
| 274.2737 | 30868.94 | | 30868.94 | 1 | | |
| 275.277 | 4557.69 | | 4557.69 | 1 | | |
| 296.255 | 6923.47 | | 6923.47 | | | |

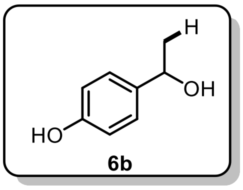

**4-(1-hydroxyethyl)phenol (6b).** Prepared according to general procedure from 0.4 mmol of octopamine hydrochloride (**6a**), 49.2 mg of **6b** was obtained (89% yield).

**TLC**: $R_f$ = 0.20 (eluent: DCM/MeOH = 15/1, visualized by $KMnO_4$ stain).

$^1$**H NMR** (400 MHz, DMSO-$d_6$) δ 9.18 (s, 1H), 7.12 (d, *J* = 8.3 Hz, 2H), 6.68 (d, *J* = 8.4 Hz, 2H), 4.92 (d, *J* = 4.1 Hz, 1H), 4.63– 4.57 (m, 1H), 1.27 (d, *J* = 6.4 Hz, 3H).

$^{13}$**C NMR** (101 MHz, DMSO-$d_6$) δ 156.4, 138.1, 126.9, 115.1, 68.2, 26.4.

**HRMS** m/z (ESI) calcd. for $C_8H_{10}NaO_2^+$ (M + Na$^+$) 161.0573, found 161.0568.

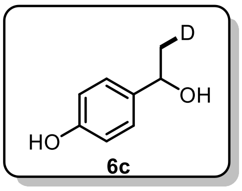

**4-(1-hydroxyethyl-2-d)phenol (6c).** Prepared according to general procedure from 0.4 mmol of octopamine hydrochloride (**6a**), 50.0 mg of **6c** was obtained (88% yield, 95%D). The deuterium incorporation was determined by $^1$H NMR.

**TLC**: $R_f$ = 0.20 (eluent: DCM/MeOH = 15/1, visualized by $KMnO_4$ stain).

**¹H NMR** (400 MHz, DMSO-$d_6$) δ 9.13 (s, 1H), 7.12 (d, *J* = 8.3 Hz, 2H), 6.69 (d, *J* = 8.4 Hz, 2H), 4.88 (d, *J* = 4.1 Hz, 1H), 4.65 – 4.57 (m, 1H), 1.28 – 1.25 (m, 2H).

**¹³C NMR** (101 MHz, DMSO-$d_6$) δ 156.4, 138.1, 126.9, 115.1, 68.2, 26.1 (t, *J* = 19.2 Hz).

**HRMS** m/z (ESI) calcd. for $C_8H_9DNaO_2^+$ (M + Na$^+$) 162.0636, found 162.0635.

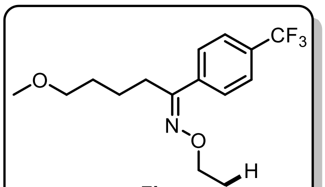

7b

**(Z)-5-methoxy-1-(4-(trifluoromethyl)phenyl)pentan-1-one O-ethyl oxime (7b).** Prepared according to general procedure from 0.4 mmol of fluvoxamine maleate (**7a**), 106.8 mg of **7b** was obtained (88% yield).

**TLC**: R$_f$ = 0.20 (eluent: PE/EA = 10/1, visualized by UV light).

**¹H NMR** (400 MHz, Chloroform-*d*) δ 7.75 (d, *J* = 8.2 Hz, 2H), 7.60 (d, *J* = 8.3 Hz, 2H), 4.25 (q, *J* = 7.0 Hz, 2H), 3.38 (t, *J* = 5.9 Hz, 2H), 3.31 (s, 3H), 2.80 – 2.77 (m, 2H), 1.69 – 1.55 (m, 4H), 1.33 (t, *J* = 7.1 Hz, 3H).

**¹³C NMR** (101 MHz, Chloroform-*d*) δ 156.7 139.4, 130.6 (q, *J* = 32.6 Hz), 126.5, 125.3 (q, *J* = 3.8 Hz), 124.1 (q, *J* = 271.9 Hz), 72.3, 69.9, 58.5, 29.5, 26.0, 23.1, 14.7.

**¹⁹F NMR** (376 MHz, Chloroform-*d*) δ -62.7.

**HRMS** m/z (ESI) calcd. for $C_{15}H_{21}F_3NO_2^+$ (M + H$^+$) 304.1519, found 304.1513.

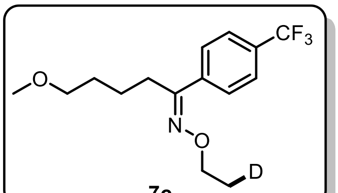

7c

**(Z)-5-methoxy-1-(4-(trifluoromethyl)phenyl)pentan-1-one O-ethyl-2-d oxime (7c).** Prepared according to general procedure from 0.4 mmol of fluvoxamine maleate (**7a**), 93.7 mg of **7c** was obtained (77% yield, 93%D). The deuterium incorporation was determined by ESI-HRMS.

**$^1$H NMR** (400 MHz, Chloroform-*d*) δ 7.75 (d, *J* = 7.9 Hz, 2H), 7.60 (d, *J* = 8.3 Hz, 2H), 4.26 – 4.33 (m, 2H), 3.38 (t, *J* = 6.0 Hz, 2H), 3.31 (s, 3H), 2.79 (t, *J* = 7.4 Hz, 2H), 1.68 – 1.55 (m, 4H), 1.33 – 1.29 (m, 2H).

**$^{13}$C NMR** (101 MHz, Chloroform-*d*) δ 156.6, 139.5, 130.6 (q, *J* = 32.5 Hz), 126.5, 125.3 (q, *J* = 3.9 Hz), 124.1 (q, *J* = 272.0 Hz), 72.3, 69.9, 58.5, 29.5, 26.0, 23.1, 14.4 (t, *J* = 19.5 Hz).

**$^{19}$F NMR** (376 MHz, Chloroform-*d*) δ -62.78.

**HRMS** m/z (ESI) calcd. for $C_{15}H_{20}DF_3NO_2^+$ (M + H$^+$) 305.1582, found 305.1575.

**TLC**: $R_f$ = 0.20 (eluent: PE/EA = 10/1, visualized by UV light).

**ESI-HRMS of 7c:**

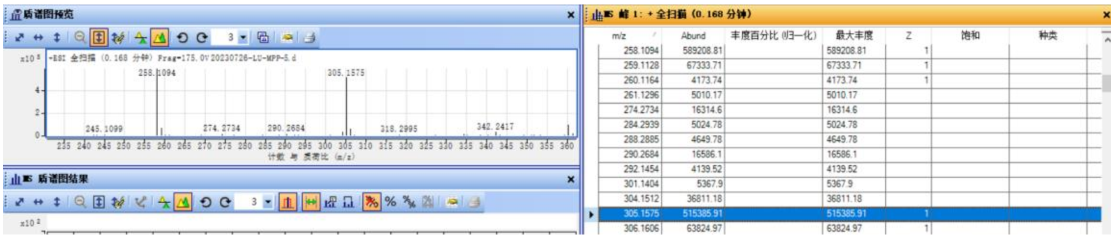


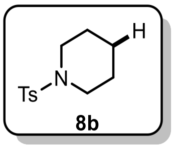

**1-tosylpiperidine (8b).** Prepared according to general procedure from 0.4 mmol of 1-tosylpiperidin-4-amine hydrochloride (**8a**), 76.6 mg of **8b** was obtained (80% yield).

**TLC**: $R_f$ = 0.60 (eluent: PE/EA = 10/1, visualized by UV light).

**$^1$H NMR** (400 MHz, Chloroform-*d*) δ 7.64 (d, *J* = 8.0 Hz, 2H), 7.33 (d, *J* = 8.0 Hz, 2H), 2.96 (t, *J* = 5.4 Hz, 4H), 2.44 (s, 3H), 1.67– 1.61 (m, 4H), 1.42 – 1.39 (m, 2H).

**$^{13}$C NMR** (101 MHz, Chloroform-*d*) δ 143.3, 129.6, 127.7, 47.0, 25.2, 23.5, 21.5.

**HRMS** m/z (ESI) calcd. for $C_{12}H_{18}NO_2S^+$ (M + H$^+$) 240.1053, found 240.1050.

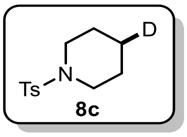

**1-tosylpiperidine-4-d (8c).** Prepared according to general procedure from 0.4 mmol of 1-tosylpiperidin-4-amine hydrochloride (**8a**), 75.0 mg of **8c** was obtained (78% yield, 96%D). The deuterium incorporation was determined by ESI-HRMS.

**TLC**: $R_f$ = 0.60 (eluent: PE/EA = 10/1, visualized by UV light).

**$^1$H NMR** (400 MHz, Chloroform-*d*) δ 7.63 (d, *J* = 8.3 Hz, 2H), 7.31 (d, *J* = 8.0 Hz, 2H), 3.02 – 2.91 (m, 4H), 2.42 (s, 3H), 1.64 – 1.60 (m, 4H),1.40 – 1.36 (m, 1H).

**$^{13}$C NMR** (101 MHz, Chloroform-*d*) δ 143.2, 133.5, 129.5, 127.7, 46.9, 25.1, 23.2 (t, *J* = 19.5 Hz), 21.5.

**HRMS** m/z (ESI) calcd. for $C_{12}H_{17}DNO_2S^+$ (M + H$^+$) 241.1116, found 241.1111.

**ESI-HRMS of 8c:**

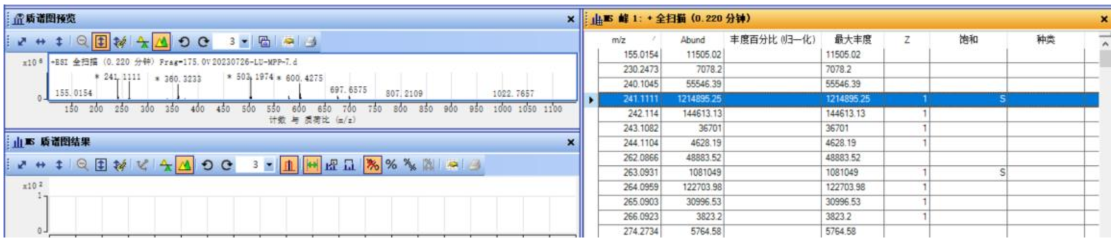


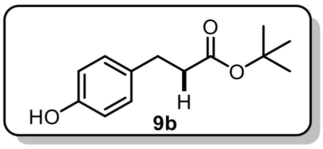

**9b**

***Tert*-butyl 3-(4-hydroxyphenyl) propanoate (9b).** Prepared according to general procedure from 0.4 mmol of tyrosinate (**9a**), 87.1 mg of **9b** was obtained (98% yield).

**TLC**: $R_f$ = 0.40 (eluent: DCM/MeOH = 15/1, visualized by UV light).

**$^1$H NMR** (400 MHz, Chloroform-*d*) δ 7.04 (d, *J* = 8.2 Hz, 2H), 6.73 (d, *J* = 8.4 Hz, 2H), 5.45 (s, 1H), 2.83 (t, *J* = 7.7 Hz, 2H), 2.51 (t, *J* = 7.7 Hz, 2H), 1.42 (s, 9H).

**$^{13}$C NMR** (101 MHz, Chloroform-*d*) δ 172.8, 154.1, 132.7, 129.4, 115.2, 80.6, 37.4, 30.3, 28.1.

**HRMS** m/z (ESI) calcd. for $C_{13}H_{18}NaO_3^+$ (M + Na$^+$) 245.1148, found 245.1143.

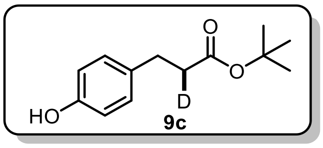

**9c**

***Tert*-butyl (S)-3-(4-hydroxyphenyl)propanoate-2-d (9c).** Prepared according to general procedure from 0.4 mmol of tyrosinate (**9a**), 83.1 mg of **9c** was obtained (93% yield, 97%D). The deuterium incorporation was determined by ESI-HRMS.

**TLC**: $R_f$ = 0.40 (eluent: DCM/MeOH = 15/1, visualized by UV light).

**$^1$H NMR** (400 MHz, Chloroform-*d*) δ 7.03 (d, *J* = 8.4 Hz, 2H), 6.74 (d, *J* = 8.4 Hz, 2H), 6.12 (s, 1H), 2.82 (d, *J* = 7.7 Hz, 2H), 2.52 – 2.47 (m, 1H), 1.42 (s, 9H).

**$^{13}$C NMR** (101 MHz, Chloroform-*d*) δ 173.1, 154.3, 132.4, 129.4, 115.3, 80.7, 37.2 (t, *J* = 19.9 Hz), 30.3, 28.1.

**HRMS** m/z (ESI) calcd. for $C_{13}H_{17}DNaO_3^+$ (M + Na$^+$) 246.1211, found 246.1204.

**ESI-HRMS of 9c:**

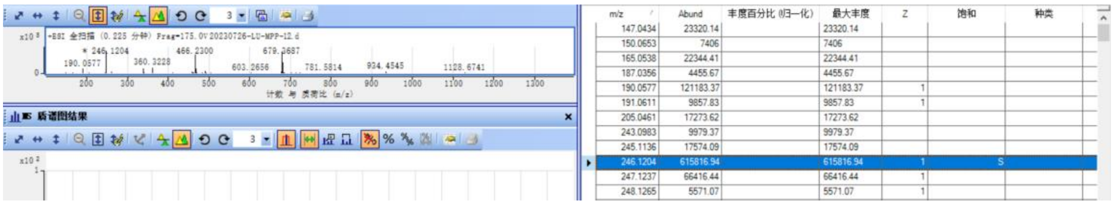

| m/z | Abund | 丰度百分比 (归一化) | 最大丰度 | Z | 饱和 | 种类 |
|---|---|---|---|---|---|---|
| 147.0434 | 23320.14 | | 23320.14 | | | |
| 150.0653 | 7406 | | 7406 | | | |
| 165.0538 | 22344.41 | | 22344.41 | | | |
| 187.0356 | 4455.67 | | 4455.67 | | | |
| 190.0577 | 121183.37 | | 121183.37 | 1 | | |
| 191.0611 | 9857.83 | | 9857.83 | 1 | | |
| 205.0461 | 17273.62 | | 17273.62 | | | |
| 243.0983 | 9979.37 | | 9979.37 | | | |
| 245.1136 | 17574.09 | | 17574.09 | | | |
| 246.1204 | 615816.94 | | 615816.94 | 1 | S | |
| 247.1237 | 66416.44 | | 66416.44 | 1 | | |
| 248.1265 | 5571.07 | | 5571.07 | 1 | | |

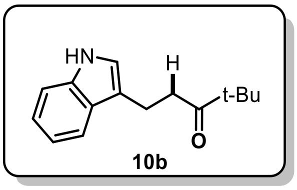

**10b**

***Tert*-butyl 3-(1H-indol-3-yl) propanoate (10b).** Prepared according to general procedure from 0.4 mmol of tryptophanate (**10a**), 81.4 mg of **10b** was obtained (83% yield).

**TLC**: $R_f$ = 0.30 (eluent: PE/EA = 5/1, visualized by UV light).

**$^1$H NMR** (400 MHz, Chloroform-*d*) δ 8.01 (s, 1H), 7.65 (d, *J* = 6.7 Hz, 1H), 7.38 (d, *J* = 8.1 Hz, 1H), 7.22 (t, *J* = 7.5 Hz, 1H), 7.15 (t, *J* = 7.4 Hz, 1H), 7.03 (s, 1H), 3.10 (t, *J* = 7.6 Hz, 2H), 2.67 (t, *J* = 7.5 Hz, 2H), 1.47 (s, 9H).

**$^{13}$C NMR** (101 MHz, Chloroform-*d*) δ 172.8, 136.3, 127.3, 122.0, 121.3, 119.2, 118.8, 115.3, 111.1, 80.2, 36.1, 28.1, 20.7.

**HRMS** m/z (ESI) calcd. for $C_{15}H_{19}NNaO_2^+$ (M + Na$^+$) 268.1308, found 268.1303.

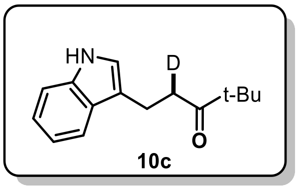


***Tert*-butyl (S)-3-(1H-indol-3-yl) propanoate-2-d (10c).** Prepared according to general procedure from 0.4 mmol of tryptophanate (**10a**), 93.6 mg of **10c** was obtained (95% yield, 96%D). The deuterium incorporation was determined by ESI-HRMS.

**TLC**: $R_f$ = 0.30 (eluent: PE/EA = 5/1, visualized by UV light).

**$^1$H NMR** (400 MHz, Chloroform-*d*) δ 8.08 (s, 1H), 7.67 (d, *J* = 7.9 Hz, 1H), 7.38 (d, *J* = 8.0 Hz, 1H), 7.25 (t, *J* = 7.5 Hz, 1H), 7.18 (t, *J* = 7.4 Hz, 1H), 7.01 (s, 1H), 3.12 (d, *J* = 7.7 Hz, 2H), 2.71 – 2.66 (m, 1H), 1.50 (s, 9H).

**$^{13}$C NMR** (101 MHz, Chloroform-*d*) δ 172.9, 136.4, 127.3, 122.0, 121.4, 119.2, 118.8, 115.2, 111.1, 80.3, 35.9 (t, *J* = 19.9 Hz), 28.2, 20.7.

**HRMS** m/z (ESI) calcd. for $C_{15}H_{18}DNNaO_2^+$ (M + Na$^+$) 269.1371, found 269.1365.

**ESI-HRMS of 10c:**

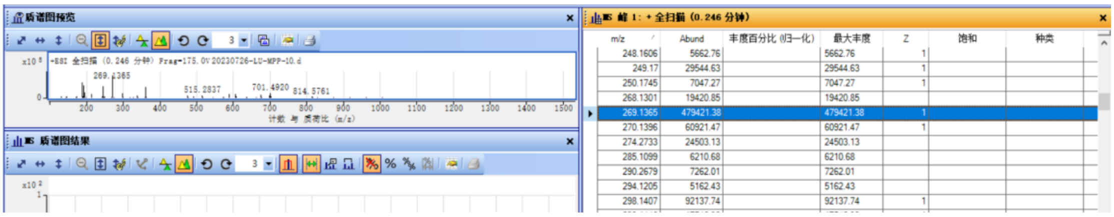


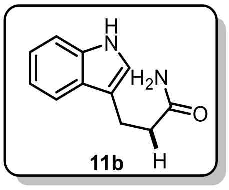


**3-(1H-indol-3-yl) propanamide (11b).** Prepared according to general procedure from 0.4 mmol of tryptophanamide (**11a**), 66.3 mg of **11b** was obtained (88% yield).

**TLC**: $R_f$ = 0.60 (eluent: DCM/MeOH = 15/1, visualized by UV light).

**$^1$H NMR** (400 MHz, DMSO-*d*$_6$) δ 10.74 (s, 1H), 7.52 (d, *J* = 7.9 Hz, 1H), 7.39 – 7.25 (m, 2H), 7.13 – 6.92 (m, 3H), 6.74 (s, 1H), 2.90 (t, *J* = 7.3 Hz, 2H), 2.41 (*J* = 7.3 Hz, 2H).

**$^{13}$C NMR** (101 MHz, DMSO-*d*$_6$) δ 174.5, 136.7, 127.5, 122.5, 121.3, 118.8, 118.6, 114.4, 111.7, 36.4, 21.3.

**HRMS** m/z (ESI) calcd. for $C_{11}H_{12}N_2NaO^+$ (M + $Na^+$) 211.0842, found 211.0838.

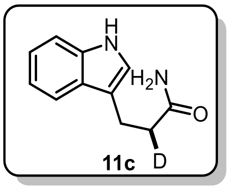

**(S)-3-(1H-indol-3-yl)propanamide (11c).** Prepared according to general procedure from 0.4 mmol of tryptophanamide (**11a**), 66.3 mg of **11c** was obtained (90% yield, 96%D). The deuterium incorporation was determined by ESI-HRMS.

**TLC**: $R_f$ = 0.60 (eluent: DCM/MeOH = 15/1, visualized by UV light).

**$^1$H NMR** (400 MHz, DMSO-$d_6$) δ 10.73 (s, 1H), 7.53 (d, $J$ = 7.8 Hz, 1H), 7.34 – 7.29 (m, 2H), 7.12 – 7.02 (m, 2H), 6.97 (t, $J$ = 7.4 Hz, 1H), 6.73 (s, 1H), 2.90 (d, $J$ = 7.7 Hz, 2H), 2.45 – 2.39 (m, 1H).

**$^{13}$C NMR** (101 MHz, DMSO-$d_6$) δ 174.5, 136.7, 127.5, 122.5, 121.3, 118.8, 118.6, 114.5, 111.7, 36.3, 36.1 (t, $J$ = 19.8 Hz), 35.9, 21.2.

**HRMS** m/z (ESI) calcd. for $C_{11}H_{11}DN_2NaO^+$ (M + $Na^+$) 212.0905, found 212.0899.

**ESI-HRMS of 11c:**

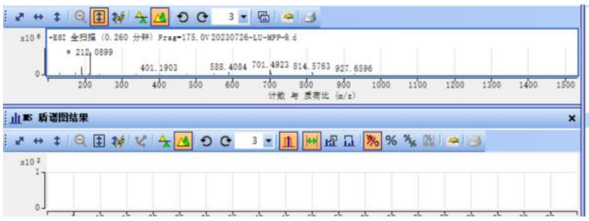

| m/z | Abund | 丰度百分比 (归一化) | 最大丰度 | Z | 饱和 | 种类 |
|---|---|---|---|---|---|---|
| 191.1111 | 31725.4 | | 31725.4 | 1 | | |
| 201.0923 | 5724.5 | | 5724.5 | | | |
| 208.578 | 4198.89 | | 4198.89 | | | |
| 209.0811 | 30253.26 | | 30253.26 | 2 | | |
| 209.5825 | 8154.31 | | 8154.31 | 2 | | |
| 211.0833 | 40920.26 | | 40920.26 | | | |
| 212.0899 | 959369.06 | | 959369.06 | 1 | S | |
| 213.0931 | 91063.36 | | 91063.36 | 1 | | |
| 214.0959 | 5066.09 | | 5066.09 | 1 | | |
| 217.1072 | 3723.3 | | 3723.3 | | | |
| 222.5809 | 4143.35 | | 4143.35 | | | |
| 228.0557 | 6897.72 | | 6897.72 | | | |
| 239.0894 | 29835.38 | | 29835.38 | | | |

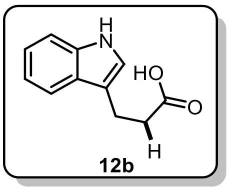

**3-(1H-indol-3-yl)propanoic acid (12b).** Prepared according to general procedure from 0.4 mmol of tryptophan (**12a**), 69.6 mg of **12b** was obtained (92% yield).

**TLC**: $R_f$ = 0.20 (eluent: DCM/MeOH = 5/1, visualized by UV light).

**$^1$H NMR** (400 MHz, DMSO-$d_6$) δ 12.05 (s, 1H), 10.77 (s, 1H), 7.51 (d, $J$ = 7.9 Hz, 1H), 7.33 (d, $J$ = 8.1 Hz, 1H), 7.10 (s, 1H), 7.06 (t, $J$ = 7.5 Hz, 1H), 6.97 (t, $J$ = 7.4 Hz, 1H),

2.92 (t, $J$ = 7.6 Hz, 2H), 2.58 (t, $J$ = 7.6 Hz, 2H).

**$^{13}$C NMR** (101 MHz, DMSO-$d_6$) δ 174.7, 136.7, 127.4, 122.7, 121.4, 118.7, 113.9, 111.8, 35.1, 20.8.

**HRMS** m/z (ESI) calcd. for $C_{11}H_{12}NO_2^+$ (M + H$^+$) 190.0863, found 190.0857.

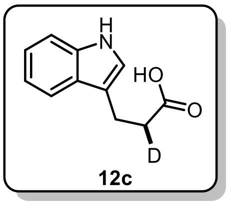

12c

**3-(1H-indol-3-yl)propanoic-2-d acid (12c).** Prepared according to general procedure from 0.4 mmol of tryptophan (**12a**), 70.0mg of **12c** was obtained (92% yield, 95%D). The deuterium incorporation was determined by ESI-HRMS.

**TLC**: $R_f$ = 0.20 (eluent: DCM/MeOH = 5/1, visualized by UV light).

**$^1$H NMR** (400 MHz, DMSO-$d_6$) δ 12.04 (s, 1H), 10.75 (s, 1H), 7.51 (d, $J$ = 7.8 Hz, 1H), 7.33 (d, $J$ = 8.0 Hz, 1H), 7.11 (d, $J$ = 2.3 Hz, 1H), 7.09 – 7.03 (m, 1H), 7.01 – 6.94 (m, 1H), 2.93 (d, $J$ = 7.5 Hz, 2H), 2.57 (t, $J$ = 7.6 Hz, 1H).

**$^{13}$C NMR** (101 MHz, DMSO-$d_6$) δ 174.7, 136.7, 127.4, 122.7, 121.4, 118.7, 113.9, 111.8, 34.8 (t, $J$ = 19.9 Hz), 20.7.

**HRMS** m/z (ESI) calcd. for $C_{11}H_{11}DNO_2^+$ (M + H$^+$) 191.0925, found 191.0918.

**ESI-HRMS of 12c:**

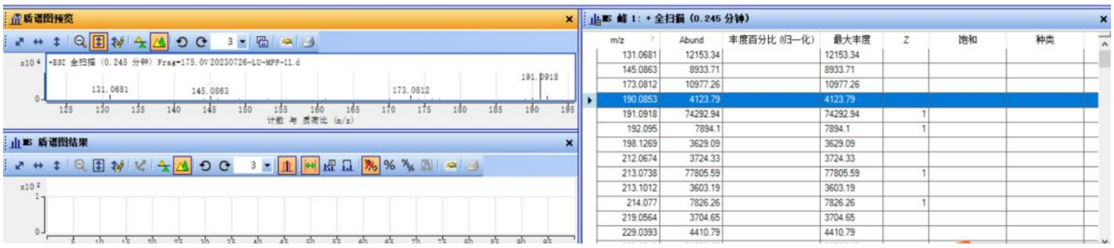


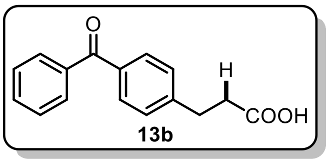

13b

**3-(4-benzoylphenyl)propanoic acid (13b).** Prepared according to general procedure from 0.4 mmol of (S)-2-amino-3-(4-benzoylphenyl)propanoic acid (**13a**), 49.8 mg of **13b** was obtained (49% yield).

**TLC**: $R_f$ = 0.40 (eluent: PE/EA = 2/1, visualized by UV light).

**$^{1}$H NMR** (400 MHz, Chloroform-*d*) δ 7.80 – 7.75 (m, 4H), 7.62 – 7.54 (m, 1H), 7.50 – 7.46 (m, 2H), 7.33 (d, *J* = 8.0 Hz, 2H), 3.05 (t, *J* = 7.7 Hz, 2H), 2.74 (t, *J* = 7.7 Hz, 2H).

**$^{13}$C NMR** (101 MHz, Chloroform-*d*) δ 196.4, 178.0, 145.1, 137.7, 135.9, 132.3, 130.5, 130.0, 128.2, 35.0, 30.5.

**HRMS** m/z (ESI) calcd. for $C_{16}H_{14}O_3Na^+$ (M + $Na^+$) 277.0835, found 277.0842.

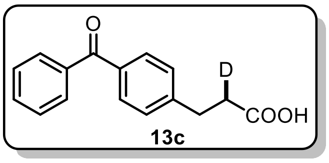


**3-(4-benzoylphenyl)propanoic-2-d acid (13c).** Prepared according to general procedure from 0.4 mmol of (S)-2-amino-3-(4-benzoylphenyl)propanoic acid (**13a**), 51.1 mg of **13c** was obtained (50% yield, 89%D). The deuterium incorporation was determined by ESI-HRMS.

**TLC**: $R_f$ = 0.40 (eluent: PE/EA = 2/1, visualized by UV light).

**$^{1}$H NMR** (400 MHz, Chloroform-*d*) δ 7.88 – 7.74 (m, 4H), 7.68 – 7.56 (m, 1H), 7.50 (t, *J* = 7.7 Hz, 2H), 7.35 (d, *J* = 7.9 Hz, 2H), 3.07 (d, *J* = 7.6 Hz, 2H), 2.79 – 2.73 (m, 1H).

**$^{13}$C NMR** (101 MHz, Chloroform-*d*) δ 196.3, 177.7, 145.1, 137.7, 135.9, 132.3, 130.5, 130.0, 128.2, 30.5.

**HRMS** m/z (ESI) calcd. for $C_{16}H_{13}DO_3Na^+$ (M + $Na^+$) 278.0898, found 278.0904.

**ESI-HRMS of 13c:**

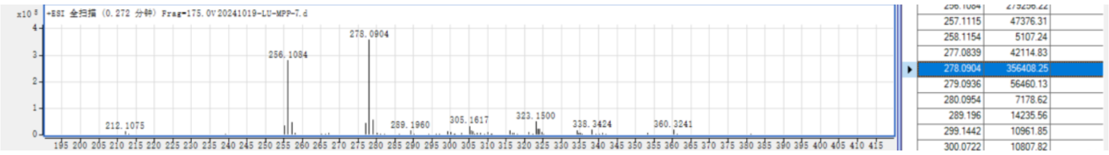

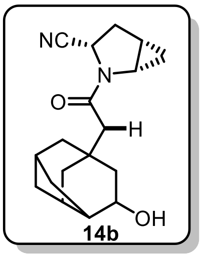

**(1S,3S,5S)-2-(2-(7-hydroxyoctahydro-5H-2,5-methanoinden-5-yl)acetyl)-2-azabicyclo[3.1.0]hexane-3-carbonitrile (14b).** Prepared according to general procedure from 0.4 mmol of saxagliptin (**14a**), 91.3 mg of **14b** was obtained (76% yield).

**TLC**: $R_f$ = 0.40 (eluent: DCM/MeOH = 10/1, visualized by UV light).

**$^1$H NMR** (400 MHz, Chloroform-*d*) δ 4.95 (dd, *J* = 10.4, 2.1 Hz, 1H), 3.51 – 3.47 (m, 1H), 2.57 – 2.50 (m, 1H), 2.40 – 2.34 (m, 3H), 2.23– 2.20 (m, 2H), 1.86 – 1.80 (m, 1H), 1.71 – 1.61 (m, 11H), 1.55 (s, 2H), 1.10 – 1.07 (m, 1H), 1.00 – 0.94 (m, 1H).

**$^{13}$C NMR** (101 MHz, Chloroform-*d*) δ 169.9, 119.7, 68.7, 50.3, 46.8, 44.9, 44.5, 44.4, 41.3, 41.1, 37.6, 37.2, 35.2, 30.7, 30.7, 30.6, 17.1, 12.8.

**HRMS** m/z (ESI) calcd. for $C_{18}H_{25}N_2O_2^+$ (M + $H^+$) 301.1911, found 301.1907.

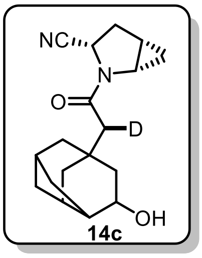

**(1S,3S,5S)-2-(2-(7-hydroxyoctahydro-5H-2,5-methanoinden-5-yl)acetyl-2-d)-2-azabicyclo[3.1.0]hexane-3-carbonitrile (14c).** Prepared according to general procedure from 0.4 mmol of saxagliptin (**14a**), 102.4 mg of **14c** was obtained (85% yield, 96%D). The deuterium incorporation was determined by ESI-HRMS.

**TLC**: $R_f$ = 0.40 (eluent: DCM/MeOH = 10/1, visualized by UV light).

**$^1$H NMR** (400 MHz, Chloroform-*d*) δ 4.95 (dd, *J* = 10.4, 2.1 Hz, 1H), 3.51 – 3.47 (m, 1H), 2.57 – 2.50 (m, 1H), 2.39 – 2.27 (m, 2H), 2.23– 2.20 (m, 2H), 1.86– 1.80 (m, 1H), 1.71– 1.60 (m, 11H), 1.56 (s, 2H), 1.10 – 1.07 (m, 1H), 1.00 – 0.94 (m, 1H).

**$^{13}$C NMR** (101 MHz, Chloroform-*d*) δ 169.9, 119.7, 68.7, 50.3, 46.5 (t, *J* = 19.5 Hz), 44.9, 44.5, 44.4, 41.3, 41.1, 37.6, 37.1, 35.2, 30.7, 30.6, 30.6, 17.1, 12.8.

**HRMS** m/z (ESI) calcd. for $C_{18}H_{24}DN_2O_2^+$ (M + H$^+$) 302.1973, found 302.1968.

**ESI-HRMS of 14c:**

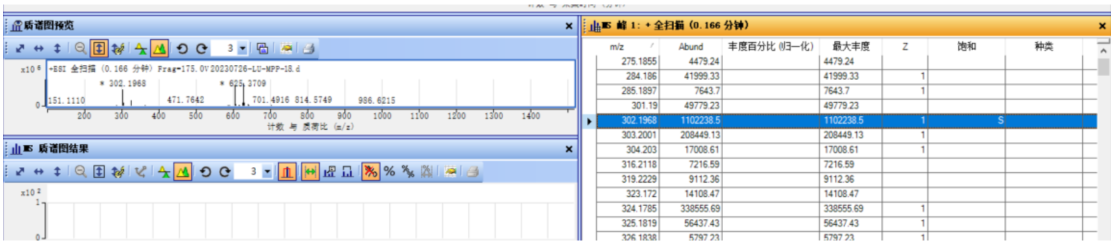

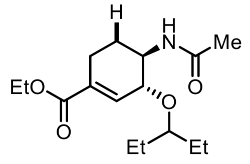


**15b**

**(ethyl (3R,4R)-4-acetamido-3-(pentan-3-yloxy)cyclohex-1-ene-1-carboxylate (15b).** Prepared according to general procedure from 0.4 mmol of oseltamivir phosphate (**15a**), 116.6 mg of **15b** was obtained (98% yield).

**TLC**: R$_f$ = 0.40 (eluent: DCM/MeOH = 20/1, visualized by $KMnO_4$ stain).

**$^1$H NMR** (400 MHz, Chloroform-*d*) δ 6.81 – 6.73 (m, 1H), 5.87 (d, *J* = 8.7 Hz, 1H), 4.15 (q, *J* = 7.1 Hz, 2H), 4.05 – 4.00 (m, 1H), 3.86 – 3.80 (m, 1H), 3.42 – 3.40 (m, 1H), 2.45 – 2.35 (m, 1H), 2.27 – 2.15 (m, 1H), 1.91 (s, 4H), 1.75 – 1.67 (m, 1H), 1.52 – 1.42 (m, 4H), 1.23 (t, *J* = 7.2 Hz, 3H), 0.86 (q, *J* = 7.4 Hz, 6H).

**$^{13}$C NMR** (101 MHz, Chloroform-*d*) δ 169.9, 166.7, 135.8, 132.5, 81.5, 72.7, 60.7, 47.8, 26.5, 26.3, 23.4, 23.3, 21.3, 14.2, 9.8, 9.3.

**HRMS** m/z (ESI) calcd. for $C_{16}H_{27}NNaO^+$ (M + Na$^+$) 320.1832, found 320.1840.

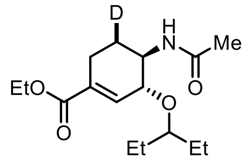


**15c**

**ethyl (3R,4R)-4-acetamido-3-(pentan-3-yloxy)cyclohex-1-ene-1-carboxylate-5-d (15c).** Prepared according to general procedure from 0.4 mmol of oseltamivir phosphate (**15a**), 105.0 mg of **15c** was obtained (88% yield, 93%D). The deuterium incorporation was determined by ESI-HRMS.

**TLC**: $R_f$ = 0.40 (eluent: DCM/MeOH = 20/1, visualized by $KMnO_4$ stain).

**$^1$H NMR** (400 MHz, Chloroform-*d*) δ 6.88 – 6.80 (m, 1H), 5.42 (d, *J* = 7.8 Hz, 1H), 4.21 (q, *J* = 7.1 Hz, 2H), 4.09 – 4.05 (m, 1H), 3.91 – 3.83 (m, 1H), 3.49 – 3.42 (m, 1H), 2.50 – 2.44 (m, 1H), 2.31 – 2.16 (m, 1H), 2.13 – 2.00 (m, 1H), 1.96 (s, 3H), 1.58 – 1.46 (m, 4H), 1.29 (t, *J* = 7.1 Hz, 3H), 0.95 – 0.89 (m, 6H).

**$^{13}$C NMR** (101 MHz, Chloroform-*d*) δ 169.7, 166.7, 135.7, 132.6, 81.5, 72.5, 60.7, 47.7, 26.5, 26.4, 23.5, 21.1, 14.2, 9.9, 9.3.

**HRMS** m/z (ESI) calcd. for $C_{16}H_{26}DNNaO^+$ (M + $Na^+$) 321.1895, found 321.1899.

**ESI-HRMS of 15c:**

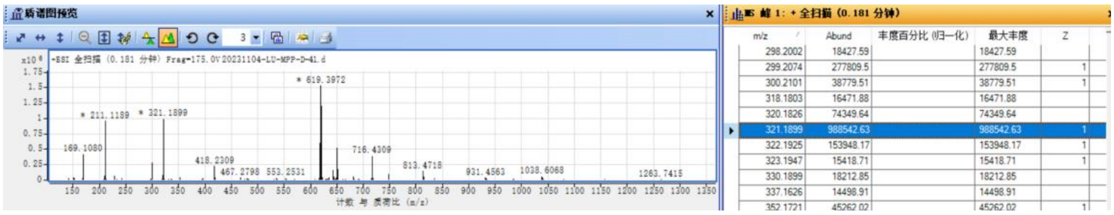


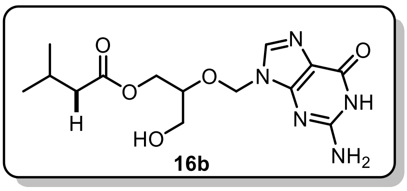

16b

**2-((2-amino-6-oxo-1,6-dihydro-9H-purin-9-yl)methoxy)-3-hydroxypropyl 3-methylbutanoate (16b).** Prepared according to general procedure from 0.4 mmol of Valganciclovir hydrochloride (**16a**), 73.3 mg of **16b** was obtained (54% yield).

**TLC**: $R_f$ = 0.30 (eluent: DCM/MeOH = 10/1, visualized by UV light).

**$^1$H NMR** (400 MHz, DMSO-*d*$_6$) δ 10.66 (s, 1H), 7.80 (s, 1H), 6.52 (s, 2H), 5.46 – 5.36 (m, 2H), 4.87 (t, *J* = 5.5 Hz, 1H), 4.10 – 4.07 (m, 1H), 3.91 – 3.86 (m, 1H), 3.82 – 3.77 (m, 1H), 3.46 – 3.33 (m, 2H), 1.99 (d, *J* = 7.1 Hz, 2H), 1.89 – 1.79 (m, 1H), 0.83 (d, *J* = 6.6 Hz, 6H).

**$^{13}$C NMR** (101 MHz, DMSO-*d*$_6$) δ 173.3, 157.6, 151.8, 138.8, 116.4, 77.4, 71.8, 63.6,

60.6, 42.7, 38.9, 38.7, 25.4, 22.2.

**HRMS** m/z (ESI) calcd. for $C_{14}H_{22}N_5O_5^+$ (M + H$^+$) 340.1615, found 340.1608.

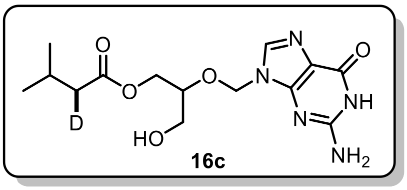


**16c**

**2-((2-amino-6-oxo-1,6-dihydro-9H-purin-9-yl)methoxy)-3-hydroxypropyl (2S)-3-methylbutanoate-2-d (16c).** Prepared according to general procedure from 0.4 mmol of valganciclovir hydrochloride (**16a**), 74.9 mg of **16c** was obtained (55% yield, 96%D). The deuterium incorporation was determined by ESI-HRMS.

**TLC**: $R_f$ = 0.30 (eluent: DCM/MeOH = 10/1, visualized by UV light).

**$^1$H NMR** (400 MHz, DMSO-$d_6$) δ 10.71 (s, 1H), 7.79 (s, 1H), 6.48 (s, 2H), 5.44 –5.35 (m, 2H), 4.99 – 4.96 (m, 1H), 4.06 – 4.02 (m, 1H), 3.89 – 3.78 (m, 2H), 3.46 –3.35 (m, 2H), 1.94 – 1.91 (m, 1H), 1.82 – 1.73 (m, 1H), 0.79 (d, $J$ = 6.6 Hz, 6H).

**$^{13}$C NMR** (101 MHz, DMSO-$d_6$) δ 172.6, 157.4, 154.2, 151.8, 138.3, 116.8, 77.4, 71.7, 63.5, 60.8, 42.4 (t, $J$ = 18.9 Hz), 25.4, 22.4, 22.4.

**HRMS** m/z (ESI) calcd. for $C_{14}H_{21}DN_5O_5^+$ (M + H$^+$) 341.1678, found 341.1671.

**ESI-HRMS of 16c:**

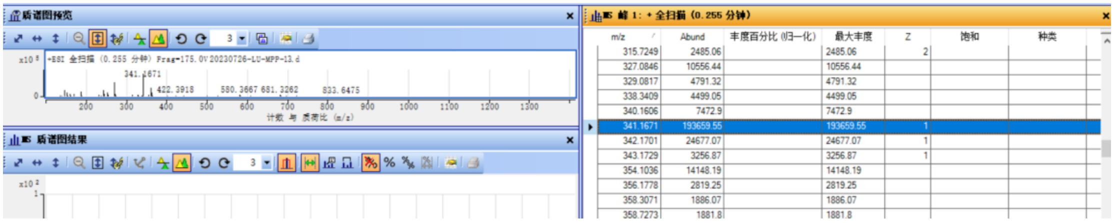


| m/z | Abund | 丰度百分比 (归一化) | 最大丰度 | Z | 饱和 | 种类 |
|---|---|---|---|---|---|---|
| 315.7249 | 2485.06 | | 2485.06 | 2 | | |
| 327.0846 | 10556.44 | | 10556.44 | | | |
| 329.0817 | 4791.32 | | 4791.32 | | | |
| 338.3409 | 4499.05 | | 4499.05 | | | |
| 340.1606 | 7472.9 | | 7472.9 | | | |
| 341.1671 | 193659.55 | | 193659.55 | 1 | | |
| 342.1701 | 24677.07 | | 24677.07 | 1 | | |
| 343.1729 | 3256.87 | | 3256.87 | 1 | | |
| 354.1036 | 14148.19 | | 14148.19 | | | |
| 356.1778 | 2819.25 | | 2819.25 | | | |
| 358.3071 | 1886.07 | | 1886.07 | | | |
| 358.7273 | 1881.8 | | 1881.8 | | | |

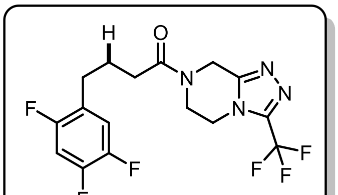


**17b**

**1-(3-(trifluoromethyl)-5,6-dihydro-[1,2,4]triazolo[4,3-a]pyrazin-7(8H)-yl)-4-(2,4,5-trifluorophenyl)butan-1-one (17b).** Prepared according to general procedure

from 0.4 mmol of sitagliptin (**17a**), 127.1 mg of **17b** was obtained (81% yield).

**TLC**: $R_f$ = 0.20 (eluent: DCM/MeOH = 15/1, visualized by UV light).

**$^1$H NMR** (400 MHz, Chloroform-*d*) δ 7.00 – 6.94 (m, 1H), 6.85 – 6.78 (m, 1H), 4.95 – 4.86 (m, 2H), 4.32 – 3.86 (m, 4H), 2.61 (t, *J* = 7.6 Hz, 2H), 2.42 (t, *J* = 7.3 Hz, 2H), 1.95 – 1.88 (m, 2H).

**$^{13}$C NMR** (101 MHz, Chloroform-*d*) δ 171.2, 170.9, 157.0, 155.8 (ddd, *J* = 243.3, 9.0, 2.2 Hz), 154.7, 150.5, 149.7, 149.6, 147.8, 147.1, 124.5 (dt, *J* = 18.5, 4.9 Hz), 122.2, 119.6, 117.9 (dd, *J* = 19.1, 6.4 Hz), 105.2 (dd, *J* = 28.7, 20.7 Hz), 43.6, 43.3, 42.3, 41.4, 39.1, 38.0, 32.0, 27.5, 24.7.

**$^{19}$F NMR** (376 MHz, Chloroform-*d*) δ -63.14 (d, *J* = 36.9 Hz), -120.39 (d, *J* = 15.4 Hz), -137.00 (d, *J* = 20.7 Hz), -143.45 (dd, *J* = 21.3, 15.1 Hz).

**HRMS** m/z (ESI) calcd. for $C_{16}H_{15}F_6N_4O^+$ (M + $H^+$) 393.1145, found 393.1137.

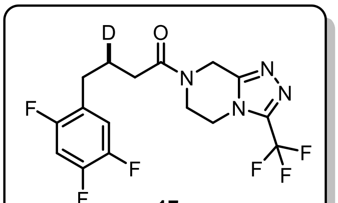


17c

**(R)-1-(3-(trifluoromethyl)-5,6-dihydro-[1,2,4]triazolo[4,3-a]pyrazin-7(8H)-yl)-4-(2,4,5-trifluorophenyl)butan-1-one-3-d (17c).** Prepared according to general procedure from 0.4 mmol of sitagliptin (**17c**), 88.1 mg of **17c** was obtained (56% yield, 95%D). The deuterium incorporation was determined by ESI-HRMS.

**TLC**: $R_f$ = 0.20 (eluent: DCM/MeOH = 15/1, visualized by UV light).

**$^1$H NMR** (400 MHz, Chloroform-*d*) δ 7.01 – 6.95 (m, 1H), 6.87 – 6.80 (m, 1H), 4.97 – 4.87 (m, 2H), 4.27 – 3.89 (m, 4H), 2.63 (d, *J* = 7.5 Hz, 2H), 2.42 (d, *J* = 7.3 Hz, 2H), 1.95 – 1.88 (m, 1H).

**$^{13}$C NMR** (101 MHz, Chloroform-*d*) δ 171.2, 155.8 (ddd, *J* = 234.4, 9.2, 2.6 Hz), 150.5, 149.7, 149.7, 147.9, 147.1, 145.5, 124.5 (dt, *J* = 18.4, 4.9 Hz), 118.2 (q, *J* = 270.2 Hz), 118.0 (dd, *J* = 18.8, 6.4 Hz), 105.5, 105.3, 105.2, 105.0, 77.3, 43.6, 43.3, 42.3, 41.5, 39.2, 38.0, 32.0, 31.9, 27.5, 24.4 (t, *J* = 19.8 Hz).

**$^{19}$F NMR** (376 MHz, Chloroform-*d*) δ -63.10 (d, *J* = 41.7 Hz), -120.38 (d, *J* = 15.1 Hz),

-136.90 (d, $J$ = 20.6 Hz), -143.36 (dd, $J$ = 21.3, 15.3 Hz).

**HRMS** m/z (ESI) calcd. for $C_{16}H_{14}DF_6N_4O^+$ (M + H$^+$) 394.1207, found 394.1202.

**ESI-HRMS of 17c:**

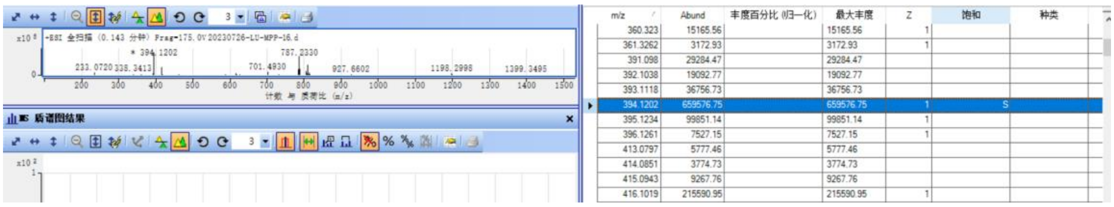

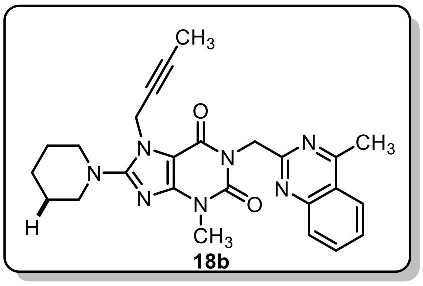

**7-(but-2-yn-1-yl)-3-methyl-1-((4-methylquinazolin-2-yl)methyl)-8-(piperidin-1-yl)-3,7-dihydro-1H-purine-2,6-dione (18b).** Prepared according to general procedure from 0.4 mmol of linagliptin (**18a**), 139.1 mg of **18b** was obtained (76% yield).

**TLC**: $R_f$ = 0.20 (eluent: PE/EA = 1/2, visualized by UV light).

**$^1$H NMR** (400 MHz, Chloroform-$d$) δ 7.95 (d, $J$ = 8.3 Hz, 1H), 7.82 (d, $J$ = 8.4 Hz, 1H), 7.70 (t, $J$ = 7.7 Hz, 1H), 7.46 (t, $J$ = 7.6 Hz, 1H), 5.54 (s, 2H), 4.82 (s, 2H), 3.53 (s, 3H), 3.35 (t, $J$ = 5.3 Hz, 4H), 2.83 (s, 3H), 1.79 – 1.57 (m, 9H)

**$^{13}$C NMR** (101 MHz, Chloroform-$d$) δ 168.3, 161.2, 156.7, 154.3, 151.9, 149.9, 133.1, 128.8, 126.5, 124.7, 123.0, 81.0, 73.3, 51.0, 46.2, 35.8, 29.68, 25.6, 24.1, 21.7, 3.6.

**HRMS** m/z (ESI) calcd. for $C_{25}H_{28}N_7O_2^+$ (M + H$^+$) 458.2299, found 458.2294.

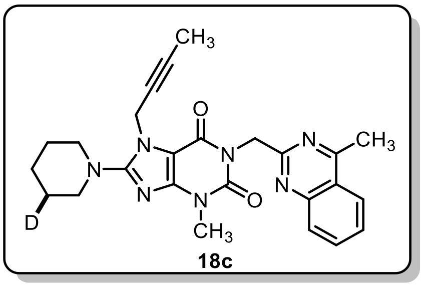

**(S)-7-(but-2-yn-1-yl)-3-methyl-1-((4-methylquinazolin-2-yl)methyl)-8-(piperidin-1-yl-3-d)-3,7-dihydro-1H-purine-2,6-dione (18c).** Prepared according to general

procedure from 0.4 mmol of linagliptin (**18a**), 133.9 mg of **18c** was obtained (73% yield, 95%D). The deuterium incorporation was determined by ESI-HRMS.

**TLC**: $R_f$ = 0.20 (eluent: PE/EA = 1/2, visualized by UV light).

**$^1$H NMR** (400 MHz, Chloroform-*d*) δ 8.00 (d, *J* = 8.3 Hz, 1H), 7.86 (d, *J* = 8.4 Hz, 1H), 7.74 (t, *J* = 7.7 Hz, 1H), 7.50 (t, *J* = 7.6 Hz, 1H), 5.56 (s, 2H), 4.86 – 4.85 (m, 2H), 3.55 (s, 3H), 3.38 (d, *J* = 5.4 Hz, 4H), 2.87 (s, 3H), 1.79 – 1.65 (m, 8H).

**$^{13}$C NMR** (101 MHz, Chloroform-*d*) δ 168.3, 161.3, 156.8, 154.4, 152.0, 150.0, 148.2, 133.1, 129.0, 126.5, 124.8, 123.2, 104.5, 81.0, 73.3, 51.1, 51.0, 46.3, 35.8, 29.7, 25.6, 25.3 (t, *J* = 19.1 Hz), 24.0, 21.7, 3.6.

**HRMS** m/z (ESI) calcd. for $C_{25}H_{27}DN_7O_2^+$ (M + H$^+$) 459.2362, found 459.2364.

**ESI-HRMS of 18c:**

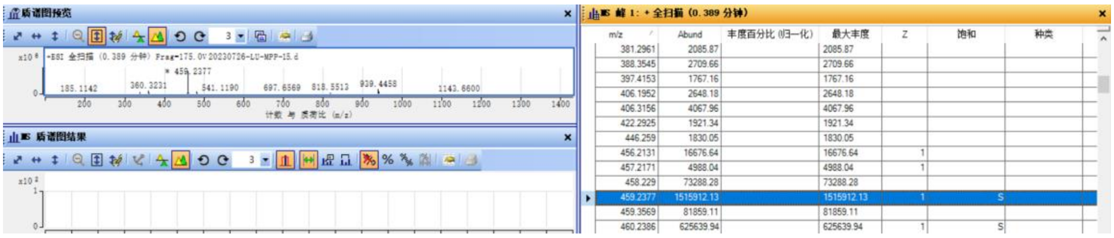

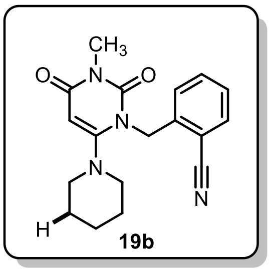


**2-((3-methyl-2,4-dioxo-6-(piperidin-1-yl)-3,4-dihydropyrimidin-1(2H)-yl)methyl)benzonitrile (19b).** Prepared according to general procedure from 0.4 mmol of alogliptin (**19a**), 120.7 mg of **19b** was obtained (93% yield).

**TLC**: $R_f$ = 0.30 (eluent: PE/EA = 1/1, visualized by UV light).

**$^1$H NMR** (400 MHz, Chloroform-*d*) δ 7.64 (d, *J* = 7.7 Hz, 1H), 7.52 (t, *J* = 7.7 Hz, 1H), 7.34 (t, *J* = 7.6 Hz, 1H), 7.09 (d, *J* = 7.9 Hz, 1H), 5.32 (s, 1H), 5.24 (s, 2H), 3.27 (s, 3H), 2.88 – 2.70 (m, 4H), 1.58 – 1.52 (m, 6H).

**$^{13}$C NMR** (101 MHz, Chloroform-*d*) δ 163.2, 160.4, 152.8, 140.9, 133.1, 133.0, 127.8, 126.5, 117.1, 90.1, 52.4, 46.4, 27.9, 25.2, 23.7.

**HRMS** m/z (ESI) calcd. for $C_{18}H_{20}N_4NaO_2^+$ (M + Na+) 347.1478, found 347.1472.

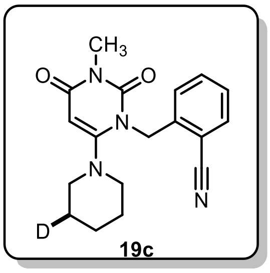

**19c**

**(R)-2-((3-methyl-2,4-dioxo-6-(piperidin-1-yl-3-d)-3,4-dihydropyrimidin-1(2H)-yl)methyl)benzonitrile (19c).** Prepared according to general procedure from 0.4 mmol of alogliptin (**19a**), 118.4 mg of **19c** was obtained (91% yield, 95%D). The deuterium incorporation was determined by ESI-HRMS.

**TLC**: $R_f$ = 0.30 (eluent: PE/EA = 1/1, visualized by UV light).

**$^1$H NMR** (400 MHz, Chloroform-*d*) δ 7.62 (d, *J* = 7.7 Hz, 1H), 7.50 (t, *J* = 7.7 Hz, 1H), 7.32 (t, *J* = 7.4 Hz, 1H), 7.08 (d, *J* = 7.9 Hz, 1H), 5.30 (s, 1H), 5.22 (s, 2H), 3.24 (s, 3H), 2.79 – 2.72 (m, 4H), 1.55 –1.49 (m, 5H).

**$^{13}$C NMR** (101 MHz, Chloroform-*d*) δ 163.1, 160.3, 152.7, 140.9, 133.1, 133.0, 127.7, 126.5, 117.0, 110.8, 90.1, 52.4, 52.3, 46.3, 27.8, 25.1, 24.8 (t, *J* = 19.5 Hz), 23.5.

**HRMS** m/z (ESI) calcd. for $C_{18}H_{20}DN_4O_2^+$ (M + H+) 326.1722, found 326.1725.

**ESI-HRMS of 19c:**

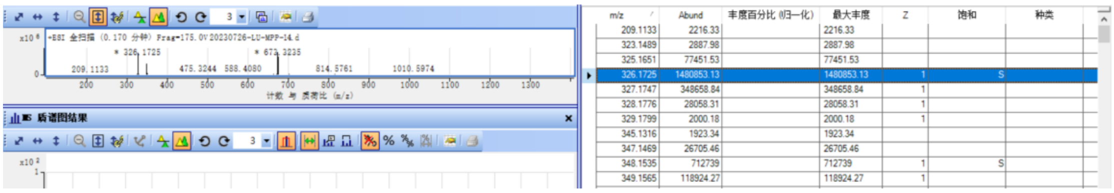


| m/z | Abund | 丰度百分比 (归一化) | 最大丰度 | Z | 饱和 | 种类 |
|---|---|---|---|---|---|---|
| 209.1133 | 2216.33 | | 2216.33 | | | |
| 323.1489 | 2887.98 | | 2887.98 | | | |
| 325.1651 | 77451.53 | | 77451.53 | | | |
| 326.1725 | 1480853.13 | | 1480853.13 | 1 | S | |
| 327.1747 | 348658.84 | | 348658.84 | 1 | | |
| 328.1776 | 28058.31 | | 28058.31 | 1 | | |
| 329.1799 | 2000.18 | | 2000.18 | 1 | | |
| 345.1316 | 1923.34 | | 1923.34 | | | |
| 347.1469 | 26705.46 | | 26705.46 | | | |
| 348.1535 | 712739 | | 712739 | 1 | S | |
| 349.1565 | 118924.27 | | 118924.27 | 1 | | |

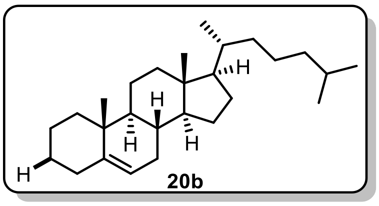

**20b**

**(8S,9S,10R,13R,14S,17R)-10,13-dimethyl-17-((R)-6-methylheptan-2-yl)-2,3,4,7,8,9,10,11,12,13,14,15,16,17-tetradecahydro-1H-cyclopenta[a]phenanthrene (20b).** Prepared according to general procedure from 0.4 mmol of (8S,9S,10R,13R,14S,17R)-10,13-dimethyl-17-((R)-6-methylheptan-2-yl)-

2,3,4,7,8,9,10,11,12,13,14,15,16,17-tetradecahydro-1H-cyclopenta[a]phenanthren-3-amine (**20a**), 120.1 mg of **20b** was obtained (81% yield).

**$^1$H NMR** (400 MHz, Chloroform-*d*) δ 5.29 – 5.25 (m, 1H), 2.28 – 2.19 (m, 1H), 2.05 – 1.90 (m, 3H), 1.89 – 1.77 (m, 2H), 1.77 – 1.68 (m, 1H), 1.59 – 0.98 (m, 26H), 0.92 (d, *J* = 6.5 Hz, 3H), 0.87 – 0.86 (m, 6H), 0.68 (s, 3H).

**$^{13}$C NMR** (101 MHz, Chloroform-*d*) δ 143.7, 119.0, 56.9, 56.2, 50.6, 39.9, 39.5, 37.6, 36.2, 35.8, 32.9, 31.9, 31.9, 28.3, 28.1, 28.0, 24.3, 23.9, 22.8, 22.6, 22.6, 20.8, 19.5, 18.7, 11.9.

**EI-HRMS** m/z (EI) calcd. for $C_{27}H_{46}^{\bullet+}$ $(M)^{\bullet+}$ 370.3594, found 370.3596.

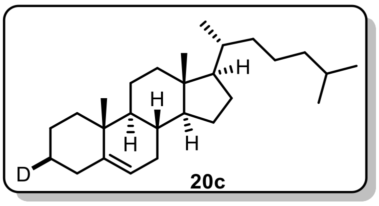


**(3S,8S,9S,10R,13R,14S,17R)-10,13-dimethyl-17-((R)-6-methylheptan-2-yl)-2,3,4,7,8,9,10,11,12,13,14,15,16,17-tetradecahydro-1H-cyclopenta[a]phenanthrene-3-d (20c).** Prepared according to general procedure from 0.4 mmol of (8S,9S,10R,13R,14S,17R)-10,13-dimethyl-17-((R)-6-methylheptan-2-yl)-2,3,4,7,8,9,10,11,12,13,14,15,16,17-tetradecahydro-1H-cyclopenta[a]phenanthren-3-amine (**20a**), 120.1 mg of **20c** was obtained (89% yield, 95%D). The deuterium incorporation was determined by EI-HRMS.

**$^1$H NMR** (400 MHz, Chloroform-*d*) δ 5.28 – 5.26 (m, 1H), 2.26 – 2.20 (m, 1H), 2.03 – 1.91 (m, 3H), 1.86 – 1.78 (m, 2H), 1.70 (s, 1H), 1.57 – 0.99 (m, 25H), 0.92 (d, *J* = 6.5 Hz, 3H), 0.88 – 0.86 (m, 6H), 0.68 (s, 3H).

**$^{13}$C NMR** (101 MHz, Chloroform-*d*) δ 143.7, 119.0, 56.9, 56.2, 50.6, 42.3, 39.9, 39.6, 37.6, 36.2, 35.8, 32.8, 31.9, 31.9, 28.3, 28.0, 27.7 (t, *J* = 18.9 Hz), 24.3, 23.9, 22.8, 22.6, 22.5, 20.8, 19.5, 18.7, 11.9.

**HRMS** m/z (EI) calcd. for $C_{27}H_{45}D^{\bullet+}$ $(M)^{\bullet+}$ 371.3657, found 371.3666.

**EI-HRMS of 20c:**

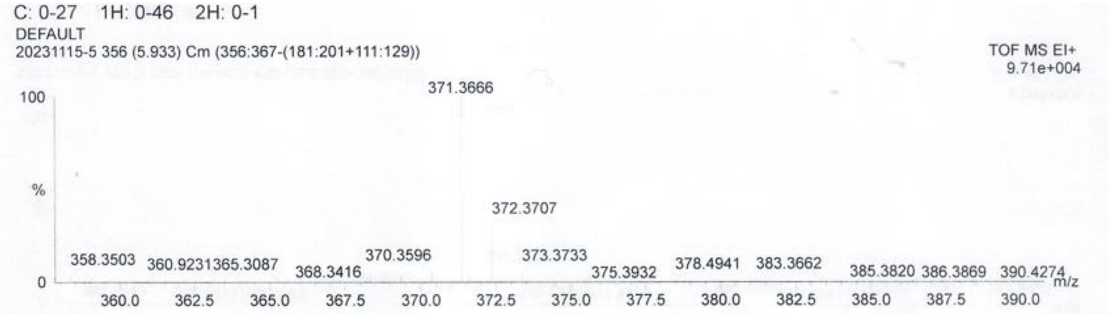


| | | | | | | | |
|---|---|---|---|---|---|---|---|
| Minimum: | 4.00 | | | | -1.5 | | |
| Maximum: | 100.00 | | 30.0 | 5.0 | 50.0 | | |
| Mass | RA | Calc. Mass | mDa | PPM | DBE | i-FIT | Formula |
| 370.3596 | 4.92 | 370.3600 | -0.4 | -1.1 | 5.0 | 61242.5 | C27 1H46 |
| | | 370.3584 | 1.2 | 3.2 | 5.5 | 62495.1 | C27 1H44 2H |
| 371.3666 | 100.00 | 371.3662 | 0.4 | 1.1 | 5.0 | 4575.3 | C27 1H45 2H |

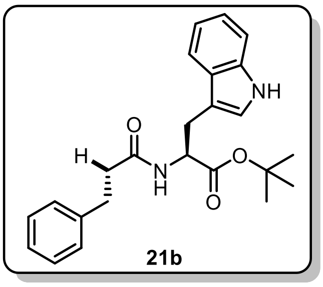

**21b**

***Tert*-butyl (3-phenylpropanoyl)-L-tryptophanate (21b).** Prepared according to general procedure from 0.4 mmol of *tert*-butyl L-phenylalanyl-L-tryptophanate (**21a**), 141.3 mg of **21b** was obtained (90% yield).

**TLC**: $R_f$ = 0.30 (eluent: DCM/MeOH = 30/1, visualized by UV light).

**$^1$H NMR** (400 MHz, Chloroform-*d*) δ 8.33 (s, 1H), 7.56 (d, *J* = 7.9 Hz, 1H), 7.37 (d, *J* = 8.2 Hz, 1H), 7.31 – 7.27 (m, 2H), 7.23 – 7.13 (m, 5H), 6.86 (s, 1H), 6.02 (d, *J* = 7.7 Hz, 1H), 4.92 – 4.87 (m, 1H), 3.29 – 3.26 (m, 2H), 3.03 – 2.87 (m, 2H), 2.59 – 2.33 (m, 2H), 1.41 (s, 9H).

**$^{13}$C NMR** (101 MHz, Chloroform-*d*) δ 171.6, 171.2, 140.8, 136.0, 128.5, 128.4, 126.2, 122.7, 122.1, 119.5, 118.9, 111.2, 110.3, 82.2, 53.4, 38.3, 31.4, 28.0, 27.6.

**HRMS** m/z (ESI) calcd. for $C_{24}H_{29}N_2O_3^+$ (M + $H^+$) 393.2173, found 393.2163.

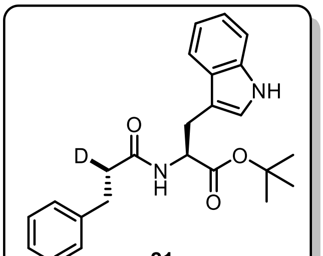

**21c**

***Tert*-butyl (3-phenylpropanoyl-2-d)-L-tryptophanate (21c).** Prepared according to general procedure from 0.4 mmol of *tert*-butyl L-phenylalanyl-L-tryptophanate (**21a**), 141.7 mg of **21c** was obtained (90% yield, 97%D). The deuterium incorporation was determined by ESI-HRMS.

**TLC**: $R_f$ = 0.30 (eluent: DCM/MeOH = 30/1, visualized by UV light).

**$^1$H NMR** (400 MHz, Chloroform-*d*) δ 8.30 (s, 1H), 7.56 (d, *J* = 7.9 Hz, 1H), 7.37 (d, *J* = 8.1 Hz, 1H), 7.33 – 7.27 (m, 2H), 7.24 – 7.08 (m, 5H), 6.87 (s, 1H), 6.01 (d, *J* = 7.8 Hz, 1H), 4.91 – 4.87 (m, 1H), 3.29 – 3.26 (m, 2H), 2.99 – 2.87 (m, 2H), 2.50 – 2.39 (m, 1H), 1.41 (s, 9H).

**$^{13}$C NMR** (101 MHz, Chloroform-*d*) δ 171.6, 171.2, 140.8, 136.0, 128.5, 128.4, 127.9, 126.2, 122.7, 122.1, 119.5, 118.9, 111.2, 110.3, 82.1, 53.4, 38.0 (t, *J* = 19.5 Hz), 31.4, 28.0, 27.6.

**HRMS** m/z (ESI) calcd. for $C_{24}H_{27}DN_2NaO_3^+$ (M + Na$^+$) 416.2055, found 416.2047.

**ESI-HRMS of 21c:**

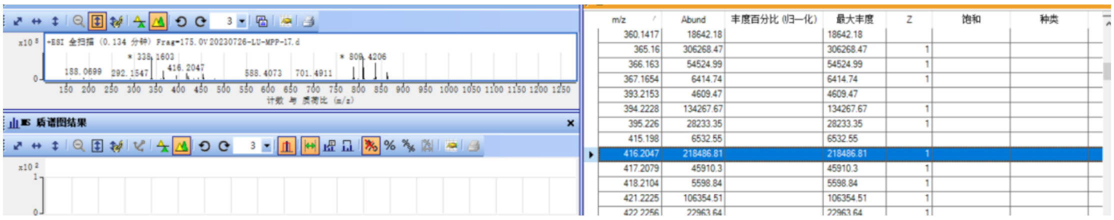

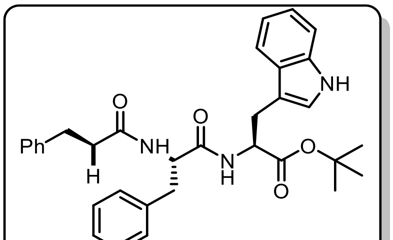

**22b**

***Tert*-butyl (3-phenylpropanoyl)phenylalanyl-L-tryptophanate (22b).** Prepared

according to general procedure from 0.4 mmol of *tert*-butyl L-phenylalanylphenylalanyl-L-tryptophanate (**22a**), 187.8 mg of **22b** was obtained (87% yield).

**TLC**: $R_f$ = 0.20 (eluent: DCM/MeOH = 30/1, visualized by UV light).

**$^1$H NMR** (400 MHz, Chloroform-*d*) δ 8.13 (s, 1H), 7.37 (d, *J* = 8.0 Hz, 1H), 7.28 – 6.99 (m, 13H), 6.88 (s, 1H), 6.36 (d, *J* = 7.6 Hz, 1H), 5.87 (d, *J* = 8.1 Hz, 1H), 4.76 – 4.62 (m, 2H), 3.27 – 3.12 (m, 2H), 3.01 – 2.91 (m, 2H), 2.85 – 2.72 (m, 2H), 2.33 – 2.17 (m, 2H), 1.36 (s, 9H).

**$^{13}$C NMR** (101 MHz, Chloroform-*d*) δ 171.8, 170.3, 170.2, 140.7, 136.4, 136.0, 129.4, 128.5, 128.5, 128.3, 127.8, 126.9, 126.2, 122.9, 122.1, 119.5, 118.8, 111.2, 110.1, 82.1, 54.0, 53.5, 38.1, 37.8, 31.3, 27.9, 27.5.

**HRMS** m/z (ESI) calcd. for $C_{33}H_{37}N_3NaO_4^+$ (M + Na$^+$) 562.2676, found 562.2664.

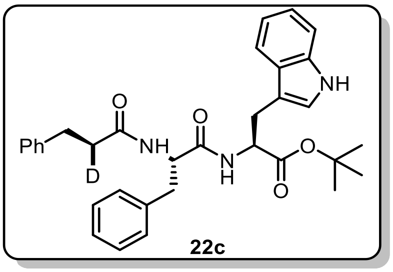


**22c**

***Tert*-butyl ((S)-3-phenylpropanoyl-2-d)phenylalanyl-L-tryptophanate (22c).** Prepared according to general procedure from 0.4 mmol of *tert*-butyl L-phenylalanylphenylalanyl-L-tryptophanate (**22a**), 162.2 mg of **22c** was obtained (75% yield, 97%D). The deuterium incorporation was determined by ESI-HRMS.

**TLC**: $R_f$ = 0.20 (eluent: DCM/MeOH = 30/1, visualized by UV light).

**$^1$H NMR** (400 MHz, Chloroform-*d*) δ 8.41 (d, *J* = 2.5 Hz, 1H), 7.39 (d, *J* = 7.9 Hz, 1H), 7.23 – 6.99 (m, 13H), 6.84 (s, 1H), 6.75 (d, *J* = 7.6 Hz, 1H), 6.16 (d, *J* = 8.3 Hz, 1H), 4.83 – 4.68 (m, 2H), 3.23 –3.09 (m, 2H), 3.00 – 2.90 (m, 2H), 2.82 – 2.65 (m, 2H), 2.23 – 2.16 (m, 1H), 1.35 (s, 9H).

**$^{13}$C NMR** (101 MHz, Chloroform-*d*) δ 172.1, 170.7, 170.5, 140.7, 136.5, 136.1, 129.5, 128.5, 128.3, 127.7, 126.9, 126.2, 123.1, 122.0, 119.4, 118.7, 111.4, 109.9, 82.2, 54.0, 53.6, 38.3, 37.6, 37.4 (t, *J* = 18.3 Hz), 37.3, 31.3, 28.0, 27.5.

**HRMS** m/z (ESI) calcd. for $C_{33}H_{37}DN_3O_4^+$ (M + $H^+$) 541.2920, found 541.2913.

**ESI-HRMS of 22c:**

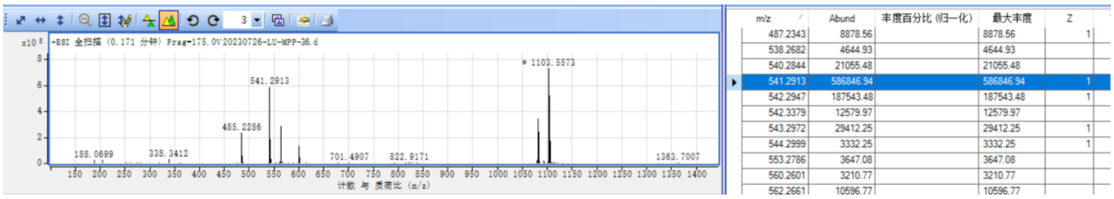


| m/z | Abund | 丰度百分比 (归一化) | 最大丰度 | Z |
|---|---|---|---|---|
| 487.2343 | 8878.56 | | 8878.56 | 1 |
| 538.2682 | 4644.93 | | 4644.93 | |
| 540.2844 | 21055.48 | | 21055.48 | |
| 541.2913 | 586846.94 | | 586846.94 | 1 |
| 542.2947 | 187543.48 | | 187543.48 | 1 |
| 542.3379 | 12579.97 | | 12579.97 | |
| 543.2972 | 29412.25 | | 29412.25 | 1 |
| 544.2999 | 3332.25 | | 3332.25 | 1 |
| 553.2786 | 3647.08 | | 3647.08 | |
| 560.2601 | 3210.77 | | 3210.77 | |
| 562.2661 | 10596.77 | | 10596.77 | |

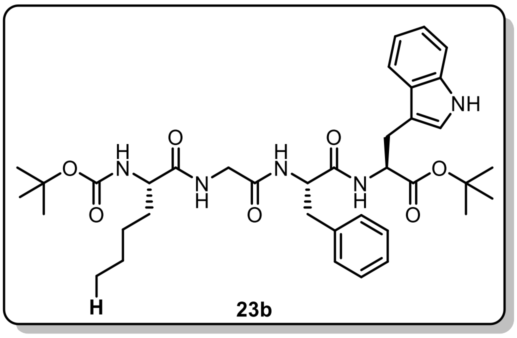

***Tert*-butyl ((S)-2-((*tert*-butoxycarbonyl)amino)hexanoyl)glycyl-L-phenylalanyl-L-tryptophanate (23b).** Prepared according to general procedure from 0.4 mmol of *tert*-butyl (*tert*-butoxycarbonyl)-L-lysylglycyl-L-phenylalanyl-L-tryptophanate (**23a**), 206.1 mg of **23b** was obtained (76% yield).

**TLC**: $R_f$ = 0.30 (eluent: PE/EA = 1/2, visualized by UV light).

**$^1$H NMR** (400 MHz, Chloroform-*d*) δ 8.97 (s, 1H), 7.48 (d, *J* = 7.9 Hz, 1H), 7.30 – 7.07 (m, 8H), 7.05 – 6.98 (m, 1H), 6.90 – 6.86 (m, 2H), 5.40 – 5.39 (m, 1H), 4.80 – 4.74 (m, 2H), 4.13 – 4.05 (m, 1H), 3.65 – 3.43 (m, 2H), 3.32 – 3.27 (m, 1H), 3.18 – 3.01 (m, 2H), 2.92 – 2.86 (m, 1H), 2.16 (s, 1H), 1.43 (s, 9H), 1.39 (s, 9H), 1.32 – 1.22 (m, 6H), 0.88 – 0.82 (m, 3H).

**$^{13}$C NMR** (101 MHz, Chloroform-*d*) δ 172.9, 170.8, 170.5, 168.8, 156.0, 136.5, 136.1, 129.3, 128.5, 127.7, 126.8, 123.2, 121.8, 119.1, 118.6, 111.3, 109.7, 82.2, 80.2, 54.6, 54.2, 53.3, 42.5, 37.7, 32.4, 28.4, 27.9, 27.7, 27.4, 22.3, 13.9.

**HRMS** m/z (ESI) calcd. for $C_{37}H_{51}N_5NaO_7^+$ (M + $Na^+$) 700.3681, found 700.3689.

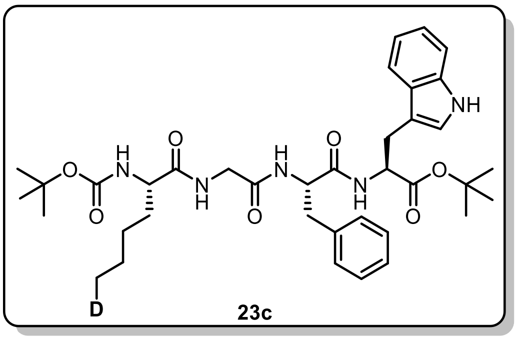

D

23c

***Tert*-butyl ((S)-2-((*tert*-butoxycarbonyl)amino)hexanoyl-6-d)glycyl-L-phenylalanyl-L-tryptophanate (23c).** Prepared according to general procedure from 0.4 mmol of *tert*-butyl (*tert*-butoxycarbonyl)-L-lysylglycyl-L-phenylalanyl-L-tryptophanate (**23a**), 176.5 mg of **23c** was obtained (65% yield, 98%D). The deuterium incorporation was determined by ESI-HRMS.

**TLC**: $R_f$ = 0.30 (eluent: PE/EA = 1/2, visualized by UV light).

**$^1$H NMR** (400 MHz, Chloroform-*d*) δ 8.96 (s, 1H), 7.48 (d, *J* = 7.8 Hz, 1H), 7.28 – 7.06 (m, 8H), 7.01 (t, *J* = 7.5 Hz, 1H), 6.89 – 6.80 (m, 2H), 5.37 – 5.36 (m, 1H), 4.81 – 4.73 (m, 2H), 4.13 – 4.07 (m, 1H), 3.59 – 3.46 (m, 2H), 3.32 – 3.28 (m, 1H), 3.14 – 3.05 (m, 2H), 2.92 – 2.87 (m, 1H), 2.08 (s, 1H), 1.44 (s, 9H), 1.40 (s, 9H), 1.36– 1.24 (m, 6H), 0.87 – 0.81 (m, 2H).

**$^{13}$C NMR** (101 MHz, Chloroform-*d*) δ 172.9, 170.8, 170.4, 168.7, 156.0, 136.5, 136.1, 129.3, 128.5, 127.7, 126.9, 123.2, 121.8, 119.1, 118.6, 111.4, 109.7, 82.2, 80.2, 54.6, 54.2, 53.2, 42.5, 37.6, 32.4, 28.4, 28.0, 27.7, 27.4, 22.3, 13.6 (t, *J* = 19.2 Hz).

**HRMS** m/z (ESI) calcd. for $C_{37}H_{50}DN_5NaO_7^+$ (M + Na$^+$) 701.3743, found 701.3755.

**ESI-HRMS of 23c:**

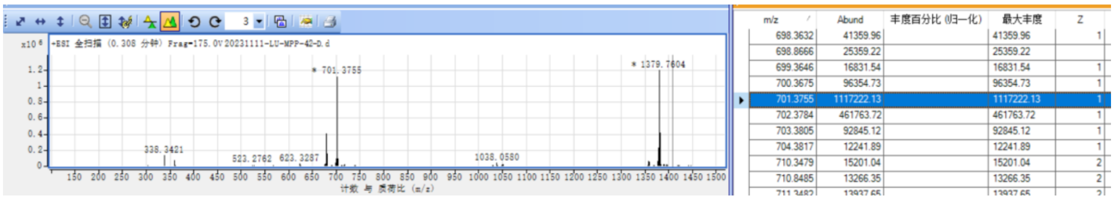


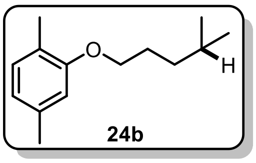

24b

**1,4-dimethyl-2-((4-methylpentyl)oxy)benzene (24b).** Prepared according to general

procedure from 0.4 mmol of 5-(2,5-dimethylphenoxy)-2-methylpentan-2-amine hydrochloride (**24a**), 65.2 mg of **24b** was obtained (79% yield); 75.9 mg of **24b** was obtained 92% yield under Argon.

**TLC**: $R_f$ = 0.50 (eluent: PE/EA = 20/1, visualized by UV light).

**$^1$H NMR** (400 MHz, Chloroform-*d*) δ 7.09 (d, *J* = 7.4 Hz, 1H), 6.79 – 6.68 (m, 2H), 4.00 (t, *J* = 6.5 Hz, 2H), 2.40 (s, 3H), 2.28 (s, 3H), 1.95 – 1.83 (m, 2H), 1.76 – 1.66 (m, 1H), 1.51 – 1.40 (m, 2H), 1.02 (d, *J* = 6.6 Hz, 6H).

**$^{13}$C NMR** (101 MHz, Chloroform-*d*) δ 157.2, 136.5, 130.3, 123.7, 120.6, 112.0, 68.2, 35.4, 27.9, 27.4, 22.7, 21.5, 15.9.

**HRMS** m/z (ESI) calcd. for $C_{14}H_{23}O^+$ (M + H$^+$) 207.1743, found 207.1737.

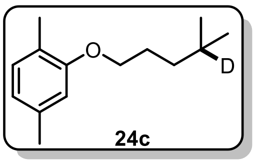


**24c**

**1,4-dimethyl-2-((4-methylpentyl-4-d)oxy)benzene (24c).** Prepared according to general procedure from 0.4 mmol of 5-(2,5-dimethylphenoxy)-2-methylpentan-2-amine hydrochloride (**24a**), 65.2 mg of **24c** was obtained (88% yield, 98% D). The deuterium incorporation was determined by ESI-HRMS.

**TLC**: $R_f$ = 0.50 (eluent: PE/EA = 20/1, visualized by UV light).

**$^1$H NMR** (400 MHz, Chloroform-*d*) δ 7.05 (d, *J* = 7.5 Hz, 1H), 6.72 – 6.68 (m, 2H), 3.98 (t, *J* = 6.6 Hz, 2H), 2.36 (s, 3H), 2.24 (s, 3H), 1.87 – 1.83 (m, 2H), 1.44 – 1.39 (m, 2H), 0.98 (d, J = 2.9 Hz, 6H).

**$^{13}$C NMR** (101 MHz, Chloroform-*d*) δ 157.2, 136.4, 130.3, 123.7, 120.6, 112.1, 68.3, 35.3, 27.3, 27.3 (t, *J* = 19.3 Hz), 22.5, 21.4, 15.8.

**HRMS** m/z (ESI) calcd. for $C_{14}H_{22}DO^+$ (M + H$^+$) 208.1806, found 208.1802.

**ESI-HRMS of 24c:**

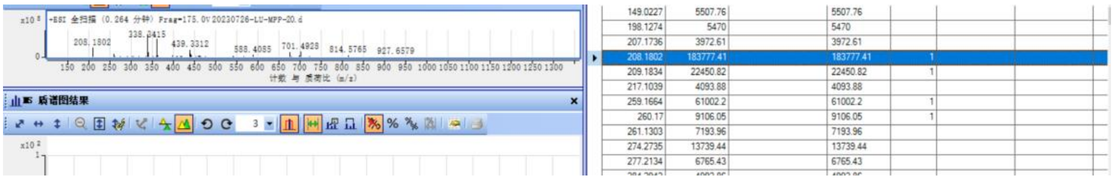

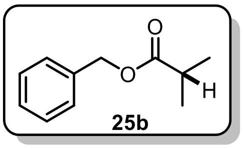

**benzyl isobutyrate (25b).** Prepared according to general procedure from 0.4 mmol of benzyl 2-amino-2-methylpropanoate (**25a**), 67.0 mg of **25b** was obtained (94% yield).

**TLC**: $R_f$ = 0.50 (eluent: PE/EA = 10/1, visualized by PMA stain).

**$^1$H NMR** (400 MHz, Chloroform-*d*) δ 7.43 – 7.29 (m, 5H), 5.12 (s, 2H), 2.67 – 2.56 (m, 1H), 1.20 (d, *J* = 7.0 Hz, 6H).

**$^{13}$C NMR** (101 MHz, Chloroform-*d*) δ 176.9, 136.3, 128.5, 128.1, 128.0, 66.0, 34.0, 19.0.

**HRMS** m/z (ESI) calcd. for $C_{11}H_{15}O_2^+$ (M + $H^+$) 179.1067, found 179.1059.

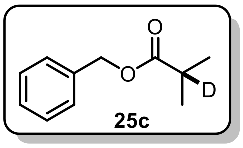

**benzyl 2-methylpropanoate-2-d (25c).** Prepared according to general procedure from 0.4 mmol of benzyl 2-amino-2-methylpropanoate (**25a**), 67.4 mg of **25c** was obtained (94% yield, 96%D). The deuterium incorporation was determined by $^1$H NMR.

**TLC**: $R_f$ = 0.50 (eluent: PE/EA = 10/1, visualized by PMA stain).

**$^1$H NMR** (400 MHz, Chloroform-*d*) δ 7.42 – 7.33 (m, 5H), 5.15 (s, 2H), 1.22 (s, 6H).

**$^{13}$C NMR** (101 MHz, Chloroform-*d*) δ 176.9, 136.4, 128.5, 128.0, 127.9, 66.0, 33.7 (t, *J* = 19.9 Hz), 18.8.

**HRMS** m/z (ESI) calcd. for $C_{11}H_{14}DO_2^+$ (M + $H^+$) 180.1129, found 180.1135.

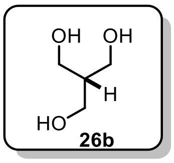

**2-(hydroxymethyl)propane-1,3-diol (26b).** Prepared according to general procedure from 0.4 mmol of 2-amino-2-(hydroxymethyl)propane-1,3-diol (**26a**), 27.2 mg of **26b** was obtained (64% yield). The spectroscopic data matched with those in literature.[5]

**TLC**: $R_f$ = 0.20 (eluent: acetone/DCM = 2/1, visualized by $KMnO_4$ stain).

**$^1$H NMR** (400 MHz, Deuterium Oxide) δ 3.56 (d, *J* = 6.0 Hz, 6H), 1.85 – 1.76 (m, 1H).
**$^{13}$C NMR** (101 MHz, Deuterium Oxide) δ 59.9, 44.7.

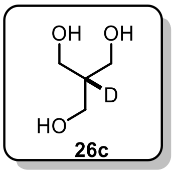

**2-(hydroxymethyl)propane-2-d-1,3-diol (26c).** Prepared according to general procedure from 0.4 mmol of 2-amino-2-(hydroxymethyl)propane-1,3-diol (**26a**), 24.1mg of **26c** was obtained (56% yield, 95%D). The deuterium incorporation was determined by $^1$H NMR.

**TLC**: $R_f$ = 0.20 (eluent: acetone/DCM = 2/1, visualized by $KMnO_4$ stain).
**$^1$H NMR** (400 MHz, Deuterium Oxide) δ 3.60 (s, 6H).
**$^{13}$C NMR** (101 MHz, Deuterium Oxide) δ 59.9, 44.3 (t, *J* = 19.4 Hz).

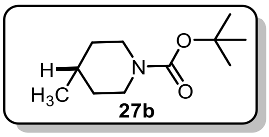

***Tert*-butyl 4-methylpiperidine-1-carboxylate (27b).** Prepared according to general procedure from 0.4 mmol of *tert*-butyl 4-amino-4-methylpiperidine-1-carboxylate (**27a**), 39.9 mg of **27b** was obtained (50% yield).

**TLC**: $R_f$ = 0.60 (eluent: PE/EA = 10/1, visualized with PMA stain).
**$^1$H NMR** (400 MHz, Chloroform-*d*) δ 4.04 – 4.00 (m, 2H), 2.65 (t, *J* = 13.2 Hz, 2H), 1.59 – 1.54 (m, 2H), 1.48 – 1.42 (m, 10H), 1.10 – 1.01 (m, 2H), 0.91 (d, *J* = 6.5 Hz, 3H).
**$^{13}$C NMR** (101 MHz, Chloroform-*d*) δ 154.9, 79.1, 44.0, 34.0, 30.9, 28.4, 21.8.
**HRMS** m/z (ESI) calcd. for $C_{11}H_{21}NNaO_2^+$ (M + $Na^+$) 222.1465, found 222.1463.

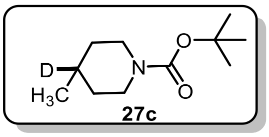

***Tert*-butyl 4-methylpiperidine-1-carboxylate-4-d (27c).** Prepared according to general procedure from 0.4 mmol of *tert*-butyl 4-amino-4-methylpiperidine-1-carboxylate (**27a**), 39.9 mg of **27c** was obtained (60% yield, 98%D). The deuterium incorporation was determined by ESI-HRMS.

**TLC**: $R_f$ = 0.60 (eluent: PE/EA = 10/1, visualized with PMA stain).

**$^1$H NMR** (400 MHz, Chloroform-*d*) δ 4.04 – 4.00 (m, 2H), 2.66 (t, *J* = 12.7 Hz, 2H), 1.56 (d, *J* = 12.9 Hz, 2H), 1.43 (s, 9H), 1.09 – 1.01 (m, 2H), 0.90 (s, 3H).

**$^{13}$C NMR** (101 MHz, Chloroform-*d*) δ 154.9, 79.0, 44.0, 33.9, 33.4 (t, *J* = 19.1 Hz), 28.4, 21.7.

**HRMS** m/z (ESI) calcd. for $C_{11}H_{20}DNNaO_2^+$ (M + Na$^+$) 223.1527, found 223.1525.

**ESI-HRMS of 27c:**

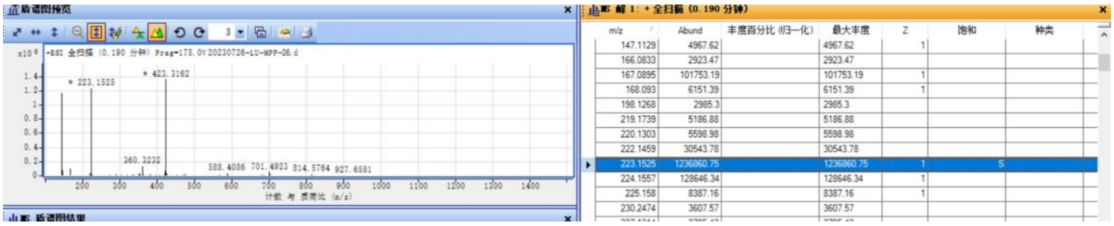

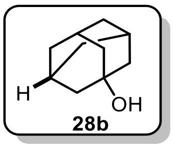


**adamantan-1-ol (28b).** Prepared according to general procedure from 0.4 mmol of 3-aminoadamantan-1-ol (**28a**), 53.6 mg of **28b** was obtained (88% yield).

**TLC**: $R_f$ = 0.40 (eluent: DCM/MeOH = 15/1, visualized by $KMnO_4$ stain).

**$^1$H NMR** (400 MHz, Chloroform-*d*) δ 2.14 (s, 3H), 1.73 – 1.54 (m, 12H), 1.43 (s, 1H).

**$^{13}$C NMR** (101 MHz, Chloroform-*d*) δ 68.2, 45.4, 36.1, 30.7.

**HRMS** m/z (ESI) calcd. for $C_{10}H_{16}NaO^+$ (M + Na$^+$) 175.1093, found 175.1085.

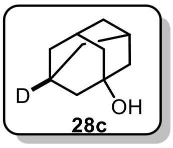


**adamantan-3-d-1-ol (28c).** Prepared according to general procedure from 0.4 mmol of

3-aminoadamantan-1-ol (**28a**), 53.6 mg of **28c** was obtained (91% yield, 95%D). The deuterium incorporation was determined by ESI-HRMS.

**TLC**: $R_f$ = 0.40 (eluent: DCM/MeOH = 15/1, visualized by $KMnO_4$ stain).

**$^1$H NMR** (400 MHz, Chloroform-*d*) δ 2.18 – 2.15 (m, 2H), 1.74 – 1.73 (m, 6H), 1.67 – 1.55 (m, 6H), 1.44 (s, 1H).

**$^{13}$C NMR** (101 MHz, Chloroform-*d*) δ 68.2, 45.4, 45.3, 36.1, 36.0, 30.3 (t, $J$ = 20.5 Hz).

**HRMS** m/z (ESI) calcd. for $C_{10}H_{15}DNaO^+$ (M + $Na^+$) 176.1156, found 176.1150.

**ESI-HRMS of 28c:**

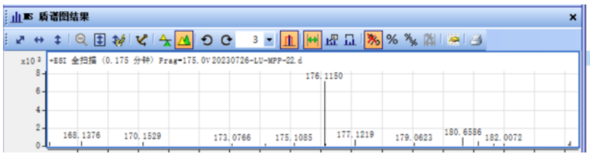


| | | | | | | |
|---|---|---|---|---|---|---|
| 170.1529 | 457.99 | | 457.99 | | | |
| 173.0766 | 282.58 | | 282.58 | | | |
| 174.184 | 231.53 | | 231.53 | | | |
| 175.1085 | 349.25 | | 349.25 | | | |
| 176.115 | 7165.1 | | 7165.1 | 1 | | |
| 176.139 | 266.75 | | 266.75 | | | |
| 177.1219 | 649.3 | | 649.3 | 1 | | |
| 179.0623 | 325.14 | | 325.14 | | | |
| 180.6586 | 946.52 | | 946.52 | 2 | | |
| 181.1592 | 477.98 | | 477.98 | 2 | | |

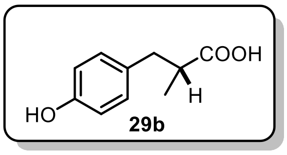


**3-(4-hydroxyphenyl)-2-methylpropanoic acid (29b).** Prepared according to general procedure from 0.4 mmol of (S)-2-amino-3-(4-hydroxyphenyl)-2-methylpropanoic acid (**29a**), 46.1 mg of **29b** was obtained (64% yield).

**TLC**: $R_f$ = 0.20 (eluent: PE/EA = 1/1, visualized by $KMnO_4$ stain).

**$^1$H NMR** (400 MHz, Chloroform-*d*) δ 7.07 – 6.98 (m, 2H), 6.76 – 6.68 (m, 2H), 2.97 – 2.92 (m, 1H), 2.75 – 2.62 (m, 2H), 1.18 (d, $J$ = 6.8 Hz, 3H).

**$^{13}$C NMR** (101 MHz, Chloroform-*d*) δ 181.4, 154.1, 131.2, 130.1, 115.3, 41.4, 38.6, 16.6.

**HRMS** m/z (ESI) calcd. for $C_{10}H_{12}O_3Na^+$ (M + $Na^+$) 203.0679, found 203.0678.

**HPLC** (Chiralpak AD-H): n-Hexane/i-PrOH = 90/10, flow rate 1.0 mL/min, λ = 220 nm, $t_{R1}$ = 12.634 min, $t_{R2}$ = 14.818 min.

<Chromatogram>

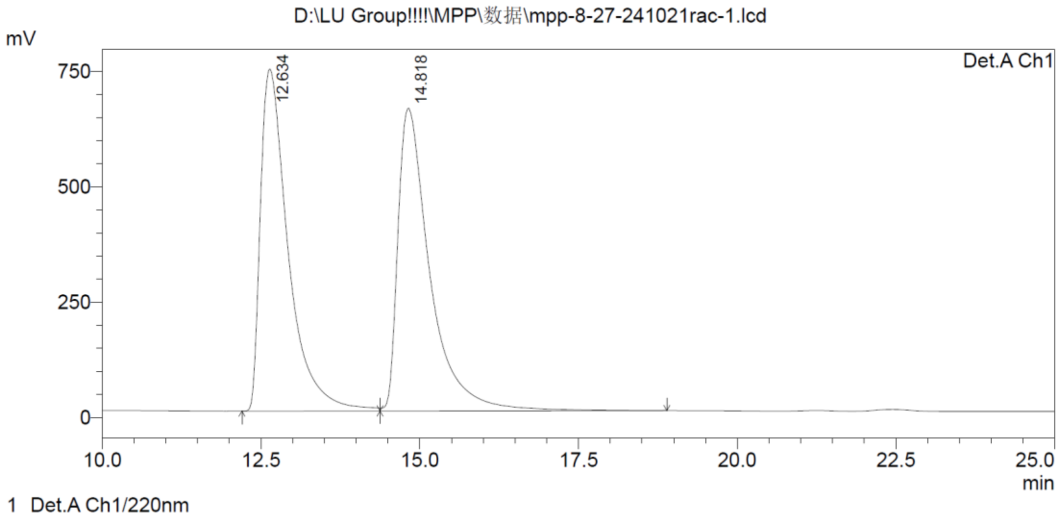


PeakTable

Detector A Ch1 220nm

| Peak# | Ret. Time | Area | Height | Area % | Height % |
|---|---|---|---|---|---|
| 1 | 12.634 | 22583564 | 741203 | 49.548 | 53.052 |
| 2 | 14.818 | 22995262 | 655921 | 50.452 | 46.948 |
| Total | | 45578826 | 1397124 | 100.000 | 100.000 |

<Chromatogram>

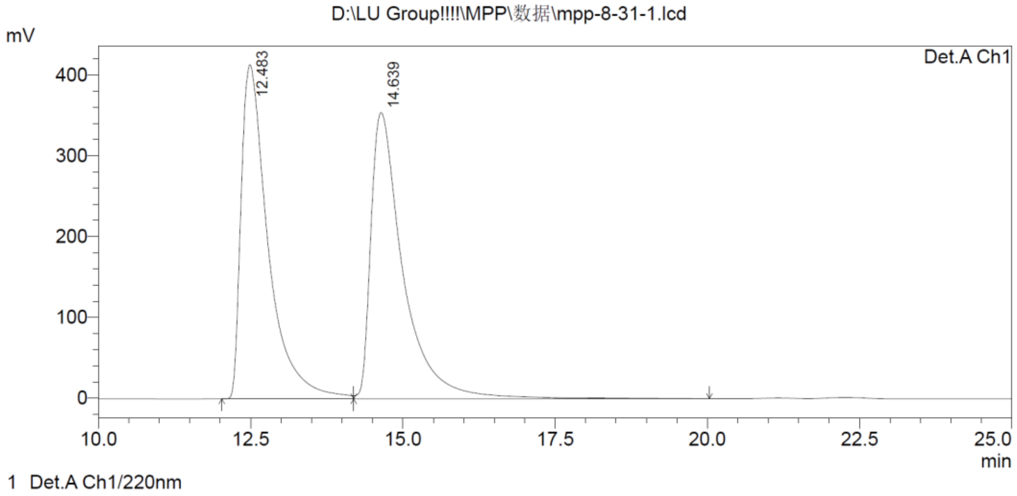


PeakTable

Detector A Ch1 220nm

| Peak# | Ret. Time | Area | Height | Area % | Height % |
|---|---|---|---|---|---|
| 1 | 12.483 | 12808254 | 413579 | 49.489 | 53.853 |
| 2 | 14.639 | 13072760 | 354397 | 50.511 | 46.147 |
| Total | | 25881014 | 767977 | 100.000 | 100.000 |

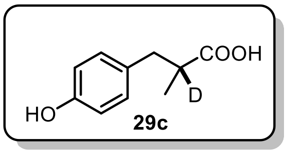


**3-(4-hydroxyphenyl)-2-methylpropanoic-2-d acid (29c).** Prepared according to general procedure from 0.4 mmol of (S)-2-amino-3-(4-hydroxyphenyl)-2-

methylpropanoic acid (**29a**), 46.1 mg of **29c** was obtained (58% yield, 94%D). The deuterium incorporation was determined by ESI-HRMS.

**TLC**: $R_f$ = 0.20 (eluent: PE/EA = 1/1, visualized by $KMnO_4$ stain).

**$^1$H NMR** (400 MHz, Chloroform-*d*) δ 7.08 – 6.97 (m, 2H), 6.78 – 6.69 (m, 2H), 2.94 (d, *J* = 13.8 Hz, 1H), 2.64 (d, *J* = 13.8 Hz, 1H), 1.18 (s, 3H).

**$^{13}$C NMR** (101 MHz, Chloroform-*d*) δ 185.5, 154.1, 131.2, 130.1, 115.3, 38.5, 16.5.

**HRMS** m/z (ESI) calcd. for $C_{10}H_{11}DO_3Na^+$ (M + $Na^+$) 204.0741, found 204.0738.

**ESI-HRMS of 29c:**

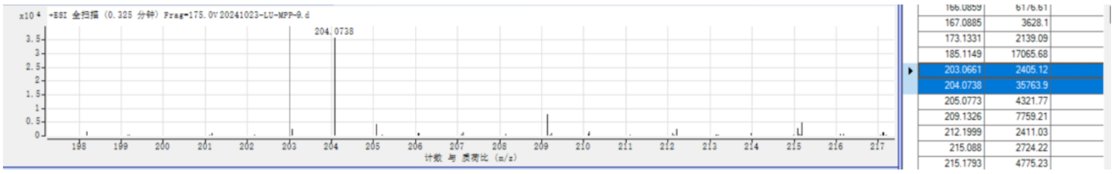


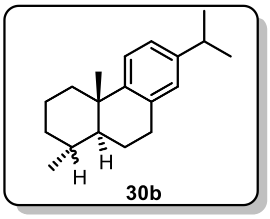


**(1R,4aS,10aS)-7-isopropyl-1,4a-dimethyl-1,2,3,4,4a,9,10,10a-octahydrophenanthrene (30b).** Prepared according to general procedure from 0.4 mmol of (1S,4aS,10aR)-7-isopropyl-1,4a-dimethyl-1,2,3,4,4a,9,10,10a-octahydrophenanthren-1-amine (**30a**), 97.4 mg of **30b** was obtained (95% yield, *dr* =1.29).

**$^1$H NMR** (400 MHz, Chloroform-*d*) δ 7.22 – 7.13 (m, 1H), 7.00 – 6.97(m, 1H), 6.90 – 6.88 (m, 1H), 2.90 – 2.82 (m, 3H), 2.28 – 2.19 (m, 1H), 2.01 – 1.90 (m, 2H), 1.75 – 1.36 (m, 7H), 1.23 – 1.21 (m, 6H), 1.17 (s, 2H), 1.09 (s, 1H), 1.00 (d, *J* = 7.6 Hz, 2H), 0.92 (d, *J* = 6.4 Hz, 1H).

**$^{13}$C NMR** (101 MHz, Chloroform-*d*) δ 147.2,145.8, 145.5, 145.4, 135.3, 135.1, 127.0, 126.9, 124.6, 124.3, 123.8, 123.7, 49.1, 44.5, 38.7, 38.3, 37.3, 37.1, 36.2, 34.0, 33.5, 33.1, 31.7, 30.4, 29.7, 25.6, 24.6, 24.0, 24.0, 22.8, 22.2, 21.4, 20.5, 18.1, 15.2.

**HRMS** m/z (EI) calcd. for $C_{19}H_{28}^{\bullet+}$ $(M)^{\bullet+}$ 256.2186, found 256.2187.

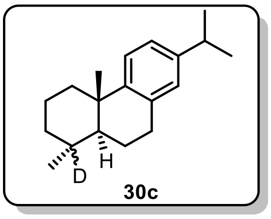


**(1R,4aS,10aS)-7-isopropyl-1,4a-dimethyl-1,2,3,4,4a,9,10,10a-octahydrophenanthrene-1-d (30c).** Prepared according to general procedure from 0.4 mmol of (1S,4aS,10aR)-7-isopropyl-1,4a-dimethyl-1,2,3,4,4a,9,10,10a-octahydrophenanthren-1-amine (**30a**), 97.4 mg of **30c** was obtained (93% yield, 95%D, *dr* =1.07). The deuterium incorporation was determined by EI-HRMS.

**$^1$H NMR** (400 MHz, Chloroform-*d*) δ 7.21 – 7.14 (m, 1H), 7.00 – 6.97 (m, 1H), 6.90 – 6.88 (m, 1H), 3.77 – 3.71 (m, 2H), 2.92 – 2.77 (m, 3H), 2.27 – 2.21 (m, 1H), 2.05 – 1.90 (m, 1H), 1.87 – 1.83 (m, 2H), 1.73 – 1.48 (m, 5H), 1.23 – 0.89 (m, 12H).

**$^{13}$C NMR** (101 MHz, Chloroform-*d*) δ 147.2, 145.8, 145.5, 145.4, 135.2, 135.0, 126.9, 126.9, 124.6, 124.3, 123.8, 123.7, 68.0, 48.9, 44.4, 38.7, 38.2, 37.3, 37.1, 36.1, 33.5, 33.0, 31.1 (t, *J* = 19.1 Hz), 30.3, 29.7, 25.67, 25.57, 24.6, 24.0, 24.0, 22.7, 22.1, 21.3, 20.3, 18.1, 15.0.

**HRMS** m/z (EI) calcd. for $C_{19}H_{27}D^{\bullet+}$ $(M)^{\bullet+}$ 257.2248, found 257.2251.

**EI-HRMS of 30c:**

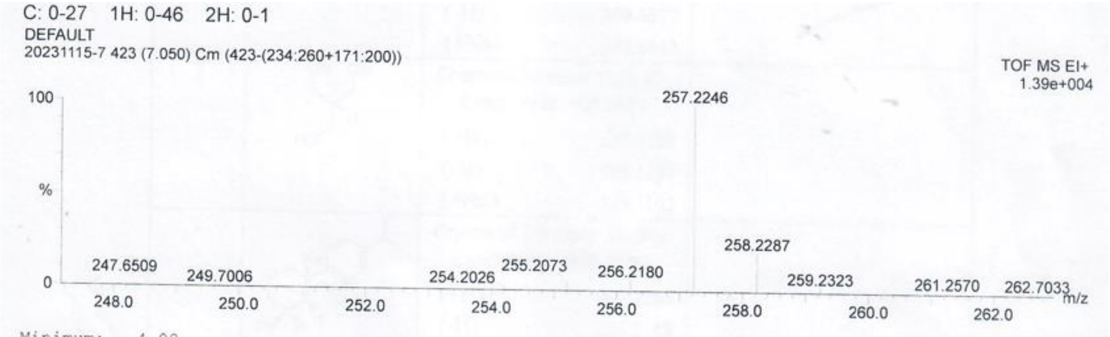


Minimum: 4.00 -1.5
Maximum: 100.00 30.0 5.0 50.0

| Mass | RA | Calc. Mass | mDa | PPM | DBE | i-FIT | Formula |
|---|---|---|---|---|---|---|---|
| 256.2180 | 5.43 | 256.2191 | -1.1 | -4.3 | 6.0 | 8225.9 | C19 1H28 |
| | | 256.2176 | 0.4 | 1.6 | 6.5 | 8366.7 | C19 1H26 2H |
| 257.2246 | 100.00 | 257.2254 | -0.8 | -3.1 | 6.0 | 433.0 | C19 1H27 2H |

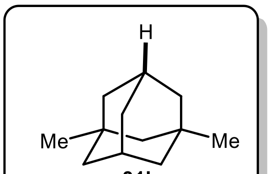


**31b**

**(1r,3r,5s,7s)-1,3-dimethyladamantane (31b).** Prepared according to general procedure from 0.4 mmol of Memantine HCl (**31a**), **31b** was tested by GC-MS with 98% yield.

**Figure S5: GC-MS of the Standard Product 31b**

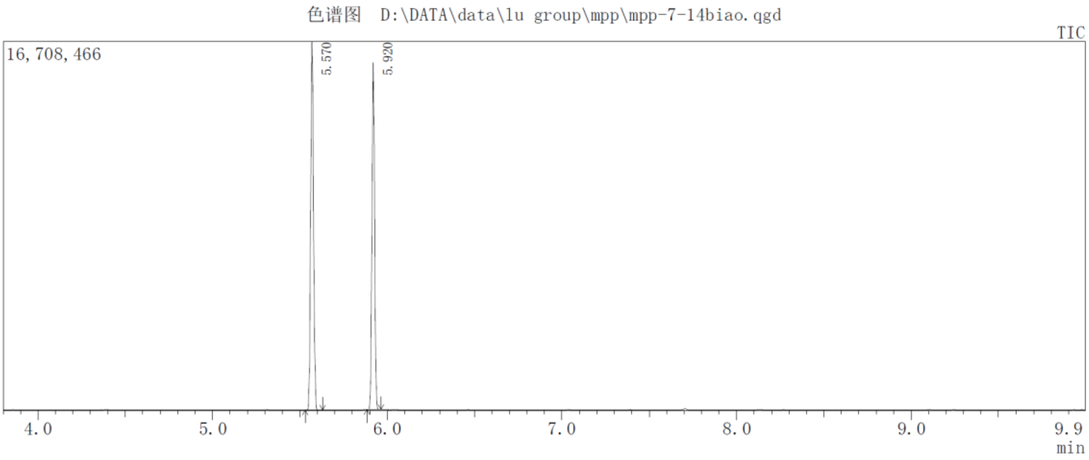


| peak | RT/min | peak area | peak area % | content |
|---|---|---|---|---|
| 1 | 5.570 | 19527108 | 53.97 | **31b** |
| 2 | 5.920 | 16654854 | 46.03 | Ddecane |

**Figure S6: GC-MS of the Crude Product 31b**

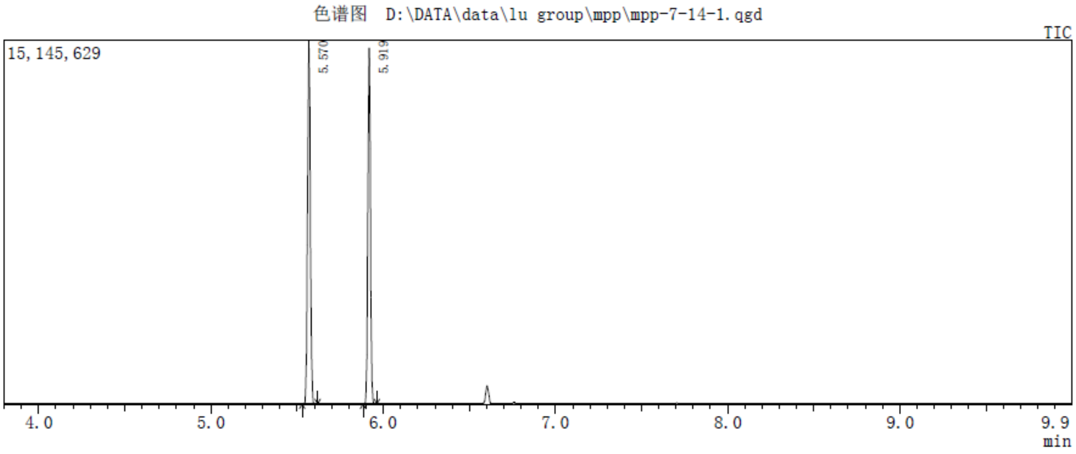

| peak | RT/min | peak area | peak area % | content |
|---|---|---|---|---|
| 1 | 5.570 | 17329196 | 52.32 | **31b** |
| 2 | 5.919 | 15794460 | 47.68 | Ddecane |

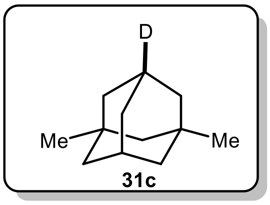


**(1R,3S,5r,7s)-1,3-dimethyladamantane-5-d (31c).** Prepared according to general procedure from 0.4 mmol of Memantine HCl (**31a**), **31c** was tested by GC-MS with 99% yield, 96%D. The deuterium incorporation was determined by GC-MS.

**Figure S7: GC-MS of the Crude Product 31c**

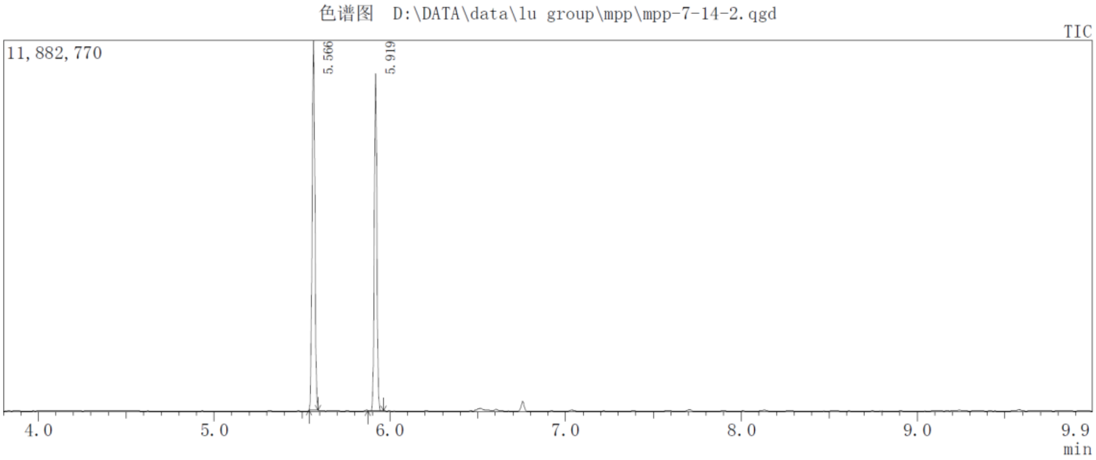


| peak | RT/min | peak area | peak area % | content |
|---|---|---|---|---|
| 1 | 5.566 | 13545107 | 54.29 | **31c** |
| 2 | 5.919 | 11405865 | 45.71 | Ddecane |

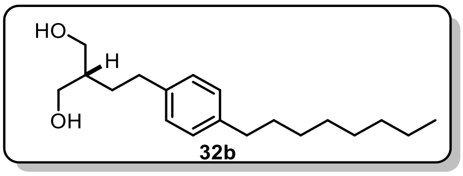


**2-(4-octylphenethyl)propane-1,3-diol (32b).** Prepared according to general procedure from 0.4 mmol of fingolimod (**32a**), 99.4 mg of **32b** was obtained (85% yield).

**TLC**: $R_f$ = 0.20 (eluent: DCM/MeOH = 15/1, visualized by $KMnO_4$ stain).

**$^1$H NMR** (400 MHz, Chloroform-*d*) δ 7.09 (s, 4H), 3.86 – 3.82 (m, 2H), 3.71 – 3.67 (m, 2H), 2.72 – 2.42 (m, 6H), 1.81 – 1.78 (m, 1H), 1.62 – 1.56 (m, 4H), 1.32 – 1.27 (m, 10H), 0.88 (t, *J* = 6.5 Hz, 3H).

**$^{13}$C NMR** (101 MHz, Chloroform-*d*) δ 140.5, 139.1, 128.4, 128.1, 66.2, 41.5, 35.6, 33.0, 31.9, 31.6, 29.5, 29.4, 29.3, 22.7, 14.1.

**HRMS** m/z (ESI) calcd. for $C_{19}H_{32}NaO_2^+$ (M + $Na^+$) 315.2295, found 315.2290.

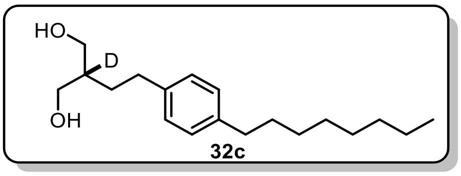

**32c**

**2-(4-octylphenethyl)propane-2-d-1,3-diol (32c).** Prepared according to general procedure from 0.4 mmol of fingolimod (**32a**), 101.0 mg of **32c** was obtained (86% yield, 97%D). The deuterium incorporation was determined by ESI-HRMS.

**TLC**: $R_f$ = 0.20 (eluent: DCM/MeOH = 15/1, visualized by $KMnO_4$ stain).

**$^1$H NMR** (400 MHz, Chloroform-*d*) δ 7.11 (s, 4H), 3.86 (d, *J* = 10.6 Hz, 2H), 3.72 (d, *J* = 10.6 Hz, 2H), 2.68 – 2.57 (m, 4H), 2.30 (s, 2H), 1.68 – 1.55 (m, 4H), 1.33 – 1.29 (m, 10H), 0.96 – 0.87 (m, 3H).

**$^{13}$C NMR** (101 MHz, Chloroform-*d*) δ 140.5, 139.1, 128.4, 128.1, 66.2, 40.9 (t, *J* = 19.1 Hz), 35.6, 32.9, 31.9, 31.6, 29.5, 29.4, 29.3, 22.7, 14.1.

**HRMS** m/z (ESI) calcd. for $C_{19}H_{31}DNaO_2^+$ (M + $Na^+$) 316.2357, found 316.2355.

**ESI-HRMS of 32c:**

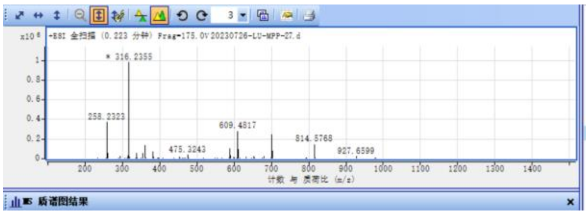


| m/z | Abund | 丰度百分比 (归一化) | 最大丰度 | Z | 饱和 | 种类 |
|---|---|---|---|---|---|---|
| 260.2389 | 4945.2 | | 4945.2 | 1 | | |
| 290.2686 | 4819.25 | | 4819.25 | | | |
| 294.2534 | 22064.14 | | 22064.14 | 1 | | |
| 295.2568 | 3850.1 | | 3850.1 | 1 | | |
| 305.156 | 4562.22 | | 4562.22 | | | |
| 311.2799 | 3722.4 | | 3722.4 | | | |
| 313.2263 | 4463.59 | | 4463.59 | | | |
| 315.2288 | 34453.95 | | 34453.95 | | | |
| 316.2355 | 981428.63 | | 981428.63 | 1 | S | |
| 317.2388 | 167095.13 | | 167095.13 | 1 | | |
| 317.2716 | 10040.83 | | 10040.83 | | | |
| 318.2417 | 14991.56 | | 14991.56 | 1 | | |
| 334.2954 | 4398.47 | | 4398.47 | | | |

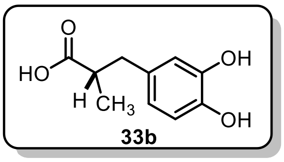

**33b**

**3-(3,4-dihydroxyphenyl)-2-methylpropanoic acid (33b).** Prepared according to general procedure from 0.4 mmol of methyldopa (**33a**), 50.2 mg of **33b** was obtained (64% yield).

**TLC**: $R_f$ = 0.20 (eluent: PE/EA = 1/1, visualized by $KMnO_4$ stain).

**$^1$H NMR** (400 MHz, DMSO-$d_6$) δ 8.69 – 8.63 (m, 2H), 6.61 (d, $J$ = 8.0 Hz, 1H), 6.55 (s, 1H), 6.41 (d, $J$ = 8.0 Hz, 1H), 2.74 – 2.69 (m, 1H), 2.54 – 2.37 (m, 2H), 1.00 (d, $J$ = 6.8 Hz, 3H).

**$^{13}$C NMR** (101 MHz, DMSO-$d_6$) δ 177.4, 145.3, 143.9, 130.8, 120.0, 116.7, 115.8, 41.4, 38.9, 17.1.

**HRMS** m/z (ESI) calcd. for $C_{10}H_{12}NaO_4^+$ (M + $Na^+$) 219.0628, found 219.0618.

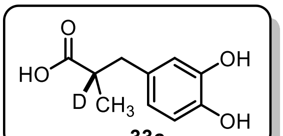

33c

**3-(3,4-dihydroxyphenyl)-2-methylpropanoic-2-d acid (33c).** Prepared according to general procedure from 0.4 mmol of methyldopa (**33a**), 59.2 mg of **33c** was obtained (75% yield, 94%D). The deuterium incorporation was determined by ESI-HRMS.

**TLC**: $R_f$ = 0.20 (eluent: PE/EA = 1/1, visualized by $KMnO_4$ stain).

**$^1$H NMR** (400 MHz, DMSO-$d_6$) δ 11.98 (s, 1H), 8.64 (s, 2H), 6.61 (d, $J$ = 7.9 Hz, 1H), 6.55 (s, 1H), 6.41 (d, $J$ = 8.0 Hz, 1H), 2.71 (d, $J$ = 13.6 Hz, 1H), 2.40 (d, $J$ = 13.6 Hz, 1H), 1.00 (s, 3H).

**$^{13}$C NMR** (101 MHz, DMSO-$d_6$) δ 177.4, 145.3, 143.9, 130.8, 120.0, 116.7, 115.8, 38.9, 17.0.

**HRMS** m/z (ESI) calcd. for $C_{10}H_{11}DNaO_4^+$ (M + $Na^+$) 220.0691, found 220.0685.

**ESI-HRMS of 33c:**

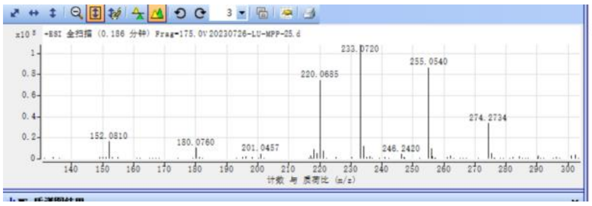


| m/z | Abund | 丰度百分比 (归一化) | 最大丰度 | Z | 饱和 | 种类 |
|---|---|---|---|---|---|---|
| 152.081 | 16334.06 | | 16334.06 | | | |
| 180.076 | 10211.67 | | 10211.67 | | | |
| 201.0457 | 4644.05 | | 4644.05 | | | |
| 218.0528 | 9342.95 | | 9342.95 | | | |
| 218.2107 | 5629.54 | | 5629.54 | | | |
| 219.0589 | 5221.65 | | 5221.65 | | | |
| 220.0685 | 74645.39 | | 74645.39 | 1 | | |
| 221.072 | 7534.31 | | 7534.31 | 1 | | |
| 233.072 | 108145.13 | | 108145.13 | 1 | | |
| 234.0752 | 12312.51 | | 12312.51 | 1 | | |
| 246.242 | 4426.82 | | 4426.82 | | | |
| 255.054 | 86519.12 | | 86519.12 | 1 | | |

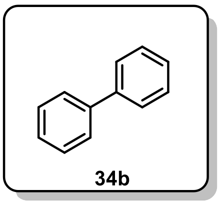

**1,1'-biphenyl (34b).** Prepared according to procedure from 0.4 mmol of 4-Aminobiphenyl (**34a**), $K_2CO_3$ (169.5 mg, 1.2 mmol, 3.0 equiv), 18-crown-6 (42.3 mg, 0.16 mmol, 40 mmol%), THF (4.0 mL) and DPPH (279.8 mg, 1.2 mmol, 3.0 equiv). The Schlenk tube was placed on a heating module preheated to 80 °C, and kept stirring at the same temperature for 24 h. 40.0 mg of **34b** was obtained (60% yield). The spectroscopic data matched with those in literature.[6]

**TLC**: $R_f$ = 0.70 (eluent: PE, visualized by UV light).

**$^1$H NMR** (400 MHz, Chloroform-*d*) δ 7.67 – 7.62 (m, 4H), 7.52 – 7.46 (m, 4H), 7.43 – 7.36 (m, 2H).

**$^{13}$C NMR** (101 MHz, Chloroform-*d*) δ 141.3, 141.3, 128.8, 127.3, 127.2.

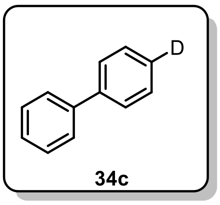

**1,1'-biphenyl-4-d (34c).** Prepared according to procedure from 0.4 mmol of 4-Aminobiphenyl (**34c**), $K_2CO_3$ (169.5 mg, 1.2 mmol, 3.0 equiv), $D_2O$ (3.3 mL), THF (0.7 mL) and DPPH (279.8 mg, 1.2 mmol, 3.0 equiv). The Schlenk tube was placed on a heating module preheated to 80 °C, and kept stirring at the same temperature for 24 h. 27.9 mg of **34c** was obtained (45% yield, 59%D). The deuterium incorporation was determined by GC-MS.

**TLC**: $R_f$ = 0.70 (eluent: PE, visualized by UV light).

**$^1$H NMR** (400 MHz, Chloroform-*d*) δ7.71 – 7.62 (m, 4H), 7.54 – 7.46 (m, 4H), 7.44 – 7.36 (m, 1H).

**$^{13}$C NMR** (101 MHz, Chloroform-*d*) δ 141.3, 128.7, 128.6, 127.2, 127.2.

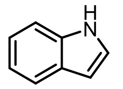

35b

**Indole (35b).** Prepared according to procedure from 0.4 mmol of 5-Aminoindole (**35a**), $K_2CO_3$ (169.5 mg, 1.2 mmol, 3.0 equiv), 18-crown-6 (42.3 mg, 0.16 mmol, 40 mmol%), THF (4.0 mL) and DPPH (279.8 mg, 1.2 mmol, 3.0 equiv). The Schlenk tube was placed on a heating module preheated to 80 °C, and kept stirring at the same temperature for 24 h. 31.4 mg of **35b** was obtained (67% yield). The spectroscopic data matched with those in literature.[7]

**TLC**: $R_f$ = 0.50 (eluent: PE/EA = 10/1, visualized by UV light).

**$^1$H NMR** (400 MHz, Chloroform-*d*) δ 7.91 (s, 1H), 7.64 (d, *J* = 7.8 Hz, 1H), 7.31 (d, *J* = 8.1 Hz, 1H), 7.18 (t, *J* = 7.7 Hz, 1H), 7.14 – 7.06 (m, 2H), 6.53 (s, 1H).

**$^{13}$C NMR** (101 MHz, Chloroform-*d*) δ 135.8, 127.9, 124.2, 122.0, 120.8, 119.9, 111.1, 102.6.

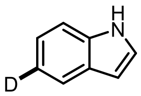

35c

**1H-indole-5-d (35c).** Prepared according to procedure from 0.4 mmol of 5-Aminoindole (**35a**), $K_2CO_3$ (169.5 mg, 1.2 mmol, 3.0 equiv), $D_2O$ (3.3 mL), THF (0.7 mL) and DPPH (279.8 mg, 1.2 mmol, 3.0 equiv). The Schlenk tube was placed on a heating module preheated to 80 °C, and kept stirring at the same temperature for 24 h. 13.2 mg of **35c** was obtained (28% yield, 33%D). The deuterium incorporation was determined by GC-MS.

**TLC**: $R_f$ = 0.50 (eluent: PE/EA = 10/1, visualized by UV light).

**$^1$H NMR** (400 MHz, Chloroform-*d*) δ 8.05 (s, 1H), 7.70 – 7.60 (m, 1H), 7.36 (d, *J* = 8.1 Hz, 1H), 7.21 – 7.08 (m, 3H), 6.55 – 6.54(m, 1H).

**$^{13}$C NMR** (101 MHz, Chloroform-*d*) δ 135.8, 127.9, 124.1, 122.0, 121.9, 120.7, 120.6, 119.8, 111.0, 102.7.

**HRMS** m/z (ESI) calcd. for $C_8H_6DNNa^+$ (M + $Na^+$) 141.0533, found 141.0523.

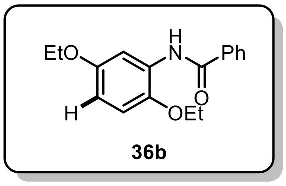

**N-(2,5-diethoxyphenyl)benzamide (36b).** Prepared according to procedure from 0.4 mmol of N-(4-amino-2,5-diethoxyphenyl)benzamide (**36a**), $K_2CO_3$ (169.5 mg, 1.2 mmol, 3.0 equiv), 18-crown-6 (42.3 mg, 0.16 mmol, 40 mmol%), THF (4.0 mL) and DPPH (279.8 mg, 1.2 mmol, 3.0 equiv). The Schlenk tube was placed on a heating module preheated to 80 °C, and kept stirring at the same temperature for 24 h. 77.6mg of **33b** was obtained (68% yield).

**TLC**: $R_f$ = 0.30 (eluent: PE/EA = 3/1, visualized by UV light).

**$^1$H NMR** (400 MHz, Chloroform-*d*) δ 8.66 (s, 1H), 8.29 (s, 1H), 7.94 – 7.85 (m, 2H), 7.57 – 7.48 (m, 3H), 6.81 (d, *J* = 8.8 Hz, 1H), 6.61 – 6.58 (m, 1H), 4.11 – 4.03 (m, 4H), 1.47 – 1.39 (m, 6H).

**$^{13}$C NMR** (101 MHz, Chloroform-*d*) δ 165.0, 153.3, 141.6, 135.3, 131.7, 128.8, 128.7, 126.9, 112.1, 109.8, 106.5, 65.0, 64.1, 15.1, 14.9.

**HRMS** m/z (ESI) calcd. for $C_{17}H_{20}NO_3$+ (M + $H^+$) 286.1438, found 286.1449.

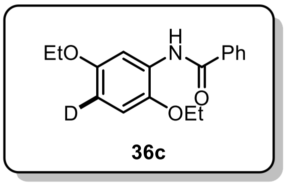

**N-(2,5-diethoxyphenyl-4-d)benzamide (36c).** Prepared according to procedure from 0.4 mmol of N-(4-amino-2,5-diethoxyphenyl)benzamide (**36a**), $K_2CO_3$ (169.5 mg, 1.2 mmol, 3.0 equiv), $D_2O$ (3.3 mL), THF (0.7 mL) and DPPH (279.8 mg, 1.2 mmol, 3.0 equiv). The Schlenk tube was placed on a heating module preheated to 80 °C, and kept stirring at the same temperature for 24 h. 47.0 mg of **36c** was obtained (41% yield, 25%D). The deuterium incorporation was determined by ESI-HRMS.

**TLC**: $R_f$ = 0.30 (eluent: PE/EA = 3/1, visualized by UV light).

**$^{1}$H NMR** (400 MHz, Chloroform-*d*) δ 8.66 (s, 1H), 8.29 (d, *J* = 3.0 Hz, 1H), 7.95 – 7.84 (m, 2H), 7.61 – 7.45 (m, 3H), 6.82 (d, *J* = 8.9 Hz, 1H), 6.61 – 6.58 (m, 1H), 4.12 – 4.03 (m, 4H), 1.47 – 1.39 (m, 6H).

**$^{13}$C NMR** (101 MHz, Chloroform-*d*) δ 164.9, 153.3, 141.5, 135.3, 131.7, 128.8, 128.7, 126.9, 112.1, 109.8, 106.5, 65.0, 64.1, 15.0, 14.9.

**HRMS** m/z (ESI) calcd. for $C_{17}H_{19}DNO_3Na^+$ (M + $Na^+$) 309.1320, found 309.1314.

**ESI-HRMS of 36c:**

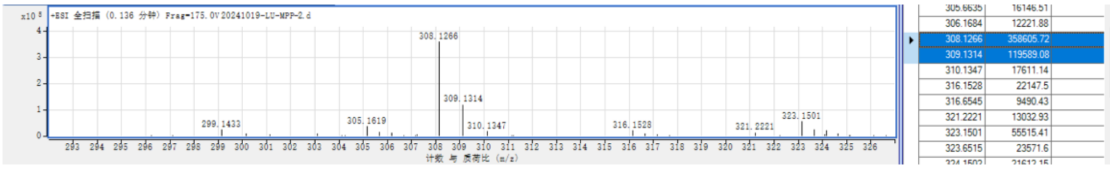


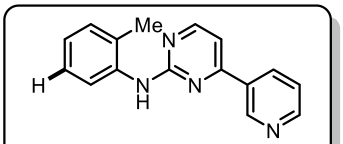

**37b**

**4-(pyridin-3-yl)-N-(o-tolyl)pyrimidin-2-amine (37b).** Prepared according to procedure from 0.4 mmol of 4-(pyridin-3-yl)-N-(o-tolyl)pyrimidin-2-amine (**37a**), $K_2CO_3$ (169.5 mg, 1.2 mmol, 3.0 equiv), 18-crown-6 (42.3 mg, 0.16 mmol, 40 mmol%), THF (4.0 mL) and DPPH (279.8 mg, 1.2 mmol, 3.0 equiv). The Schlenk tube was placed on a heating module preheated to 80 °C, and kept stirring at the same temperature for 24 h. 63.0mg of **37b** was obtained (60% yield).

**TLC**: $R_f$ = 0.20 (eluent: PE/EA = 1/1, visualized by UV light).

**$^{1}$H NMR** (400 MHz, Chloroform-*d*) δ 9.26 (d, *J* = 1.8 Hz, 1H), 8.71 – 8.70 (m, 1H), 8.48 (d, *J* = 5.1 Hz, 1H), 8.34 – 8.31 (m, 1H), 8.06 (d, *J* = 8.1 Hz, 1H), 7.42 – 7.39 (m, 1H), 7.33 – 7.20 (m, 2H), 7.14 (d, *J* = 5.1 Hz, 1H), 7.08 – 7.04 (m, 2H), 2.36 (s, 3H).

**$^{13}$C NMR** (101 MHz, Chloroform-*d*) δ 162.6, 159.1, 151.5, 148.6, 134.5, 132.7, 130.6, 126.6, 123.9, 123.6, 122.0, 108.1, 18.2.

**HRMS** m/z (ESI) calcd. for $C_{16}H_{14}N_4Na^+$ (M + $Na^+$) 285.1111, found 285.1101.

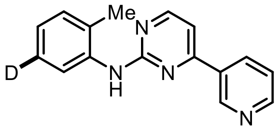


**37c**

**N-(2-methylphenyl-5-d)-4-(pyridin-3-yl)pyrimidin-2-amine (37c).** Prepared according to procedure from 0.4 mmol of 4-(pyridin-3-yl)-N-(o-tolyl)pyrimidin-2-amine (**37a**), $K_2CO_3$ (169.5 mg, 1.2 mmol, 3.0 equiv), $D_2O$ (3.3 mL), THF (0.7 mL) and DPPH (279.8 mg, 1.2 mmol, 3.0 equiv). The Schlenk tube was placed on a heating module preheated to 80 °C, and kept stirring at the same temperature for 24 h. 42.1 mg of **37c** was obtained (40% yield, 29%D). The deuterium incorporation was determined by ESI-HRMS.

**TLC**: $R_f$ = 0.20 (eluent: PE/EA = 1/1, visualized by UV light).

**$^1$H NMR** (400 MHz, Chloroform-*d*) δ 9.27 (d, *J* = 2.3 Hz, 1H), 8.72 – 8.71 (m, 1H), 8.49 (d, *J* = 5.1 Hz, 1H), 8.33 (d, *J* = 8.0 Hz, 1H), 8.06 (d, *J* = 8.2 Hz, 1H), 7.42 – 7.39 (m 1H), 7.29 – 7.19 (m, 2H), 7.14 (d, *J* = 5.2 Hz, 1H), 7.10 – 7.06 (m, 1H), 2.37 (s, 3H).

**$^{13}$C NMR** (101 MHz, Chloroform-*d*) δ 162.6, 160.9, 159.1, 151.4, 148.6, 137.3, 134.4, 132.7, 130.6, 129.1, 126.6, 124.0, 123.6, 122.2, 108.0, 18.1.

**HRMS** m/z (ESI) calcd. for $C_{16}H_{14}DN_4^+$ (M + $H^+$) 264.1354, found 264.1348.

**ESI-HRMS of 37c:**

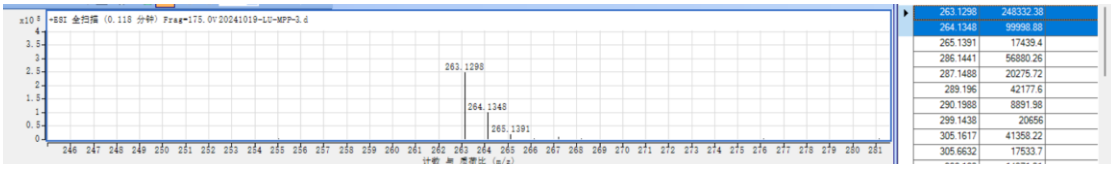

# 6. Synthetic Applications

## 6.1 Synthesis of Pseudo-α-D-sorbopyranose

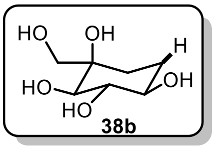

**(1R,2R,3S,4R)-1-(hydroxymethyl)cyclohexane-1,2,3,4-tetraol (38b).** Prepared according to general procedure from 0.4 mmol of valiolamine (**38a**). The reaction solution was removed and the anion exchange resin was added to the crude product. After two days, it was filtered, removed *in vacuo* and flash chromatography over silica gel. 49.2 mg of **38b** was obtained (69% yield).

**TLC**: $R_f$ = 0.30 (eluent: DCM/MeOH = 4/1, visualized by $KMnO_4$ stain).

**$^1$H NMR** (400 MHz, Deuterium Oxide) δ 3.50 – 3.27 (m, 5H), 1.79 – 1.70 (m, 1H), 1.67 – 1.37 (m, 3H).

**$^{13}$C NMR** (101 MHz, Deuterium Oxide) δ 75.8, 74.5, 73.6, 72.4, 66.0, 28.1, 26.4.

**HRMS** m/z (ESI) calcd. for $C_7H_{14}O_5Na^+$ (M + $Na^+$) 201.0733, found 201.0728.

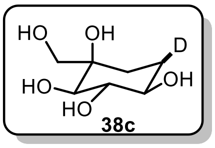

**(1R,2R,3S,4R)-1-(hydroxymethyl)cyclohexane-5-d-1,2,3,4-tetraol (38c).** Prepared according to general procedure from 0.4 mmol of valiolamine (**38a**). The reaction solution was removed and the anion exchange resin was added to the crude product. After two days, it was filtered, removed *in vacuo* and flash chromatography over silica gel. 45.0 mg of **38c** was obtained (63% yield, 89%D). The deuterium incorporation was determined by ESI-HRMS.

**TLC**: $R_f$ = 0.30 (eluent: DCM/MeOH = 4/1, visualized by $KMnO_4$ stain).

**$^1$H NMR** (400 MHz, Deuterium Oxide) δ 3.47 – 3.14 (m, 5H), 1.71 – 1.27 (m, 3H).

**$^{13}$C NMR** (101 MHz, Deuterium Oxide) δ 75.7, 74.4, 73.5, 72.3, 65.9, 27.9, 26.0 (t, *J* = 20.3 Hz).

**HRMS** m/z (ESI) calcd. for $C_7H_{13}DNaO_5^+$ (M + Na$^+$) 202.0796, found 202.0792.

**ESI-HRMS of 38c:**

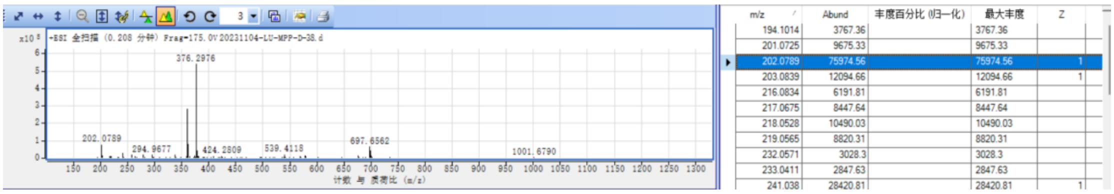


## 6.2 Synthesis of Acetylcholinesterase Inhibitors 39b & 39c

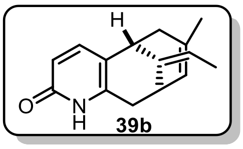


**(9R,E)-11-ethylidene-7-methyl-5,6,9,10-tetrahydro-5,9-methanocycloocta[b]pyridine-2(1H)-one (39b).** Prepared according to general procedure from 0.4 mmol of (-)-huperzine A (**39a**), $K_2CO_3$ (4.2 equiv;), DPPH (O-(Diphenylphosphinyl) hydroxylamine, 4.2 equiv). 52.7 mg of **39b** was obtained (58% yield).

**TLC**: $R_f$ = 0.20 (eluent: PE/EA = 1/1, visualized by $KMnO_4$ stain).

**$^1$H NMR** (400 MHz, Chloroform-*d*) δ 7.12 (d, *J* = 9.1 Hz, 1H), 6.50 (d, *J* = 9.1 Hz, 1H), 5.44 – 5.42 (m, 1H), 5.36 (q, *J* = 6.7 Hz, 1H), 5.14 (s, 1H), 3.48 – 3.44 (m, 1H), 3.19 (d, *J* = 5.2 Hz, 1H), 3.09 – 3.04 (m, 1H), 2.79 – 2.73 (m, 1H), 2.57 – 2.45 (m, 1H), 2.00 (d, *J* = 16.9 Hz, 1H), 1.62 (d, *J* = 6.7 Hz, 3H), 1.54 (s, 3H).

**$^{13}$C NMR** (101 MHz, Chloroform-*d*) δ 161.1, 143.5, 138.9, 136.6, 133.1, 124.8, 119.9, 115.5, 113.8, 41.8, 41.3, 34.9, 30.7, 22.9, 12.2.

**HRMS** m/z (ESI) calcd. for $C_{15}H_{17}NNaO^+$ (M + Na$^+$) 250.1202, found 250.1195.

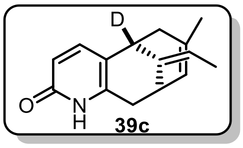


**(9R,E)-11-ethylidene-7-methyl-5,6,9,10-tetrahydro-5,9-methanocycloocta[b]pyridin-2(1H)-one-5-d (39b).** Prepared according to general

procedure from 0.4 mmol of (-)-huperzine A (**39a**), 64.8 mg of **39b** was obtained (71% yield, 87%D). The deuterium incorporation was determined by $^{1}$H NMR.

**TLC**: $R_f$ = 0.20 (eluent: PE/EA = 1/1, visualized by $KMnO_4$ stain).

**$^{1}$H NMR** (400 MHz, Chloroform-*d*) δ 7.12 (d, *J* = 9.1 Hz, 1H), 6.51 (d, *J* = 9.1 Hz, 1H), 5.48 – 5.46 (m, 1H), 5.36 (q, *J* = 6.7 Hz, 1H), 5.13 (s, 1H), 3.51 – 3.48 (m, 1H), 3.10 (d, *J* = 17.7 Hz, 1H), 2.83 – 2.77 (m, 1H), 2.57 – 2.44 (m, 1H), 2.00 (d, *J* = 17.0 Hz, 1H), 1.66 (d, *J* = 6.8 Hz, 3H), 1.59 (s, 3H).

**$^{13}$C NMR** (101 MHz, Chloroform-*d*) δ 161.1, 143.5, 138.8, 136.5, 133.1, 124.8, 119.8, 115.5, 113.7, 41.5 (t, *J* = 25.5 Hz), 41.2, 34.9, 30.7, 22.9, 12.2.

**HRMS** m/z (ESI) calcd. for $C_{15}H_{16}DNO^{+}$ (M + $H^{+}$) 229.1446, found 229.1442.

## 6.3 Synthesis of Abietratriene 40b & Deutorated Abietratriene 40c

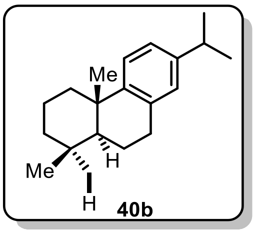


**(4aS,10aS)-7-isopropyl-1,1,4a-trimethyl-1,2,3,4,4a,9,10,10a-octahydrophenanthrene (40b).** Prepared according to general procedure from 0.4 mmol of leelamine (**40a**), 91.6 mg of **40b** was obtained (85% yield).

**$^{1}$H NMR** (400 MHz, Chloroform-*d*) δ 7.28 (d, *J* = 8.1 Hz, 1H), 7.09 (d, *J* = 8.1 Hz, 1H), 6.99 (s, 1H), 3.07 – 2.87 (m, 3H), 2.39 – 2.36 (m, 1H), 2.00 – 1.94 (m, 1H), 1.90 – 1.65 (m, 3H), 1.61 – 1.43 (m, 3H), 1.35 – 1.31 (s, 7H), 1.29 (s, 3H), 1.04 (d, *J* = 6.9 Hz, 6H).

**$^{13}$C NMR** (101 MHz, Chloroform-*d*) δ 147.7, 145.4, 135.0, 126.9, 124.3, 123.9, 50.5, 41.8, 38.9, 33.5, 33.5, 33.4, 30.6, 25.0, 24.1, 24.1, 21.7, 19.4, 19.2.

**HRMS** m/z (ESI) calcd. for $C_{20}H_{31}^{+}$ (M + $H^{+}$) 271.2420, found 271.2411.

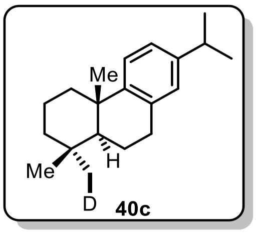

40c

**(1R,4aS,10aS)-7-isopropyl-1,4a-dimethyl-1-(methyl-d)-1,2,3,4,4a,9,10,10a-octahydrophenanthrene (40c).** Prepared according to general procedure from 0.4 mmol of leelamine (**40a**), 105.3 mg of **40c** was obtained (97% yield, 95%D). The deuterium incorporation was determined by EI-HRMS.

**$^1$H NMR** (400 MHz, Chloroform-*d*) δ 7.17 (d, *J* = 8.2 Hz, 1H), 6.98 (d, *J* = 8.2, 1H), 6.88 (s, 1H), 3.00 – 2.75 (m, 3H), 2.29 – 2.24 (m, 1H), 1.87 – 1.33 (m, 8H), 1.23 – 1.18 (m, 9H), 0.98 – 0.87 (m, 5H).

**$^{13}$C NMR** (101 MHz, Chloroform-*d*) δ 147.8, 145.48, 135.0, 126.9, 124.3, 123.8, 50.5, 41.8, 39.0, 37.6, 33.5, 33.1 (t, *J* = 19.1 Hz), 30.6, 25.0, 24.1, 24.1, 24.0, 21.7, 19.4, 19.2.

**HRMS** m/z (EI) calcd. for $C_{20}H_{29}D^{\bullet+}$ $(M)^{\bullet+}$ 271.2405, found 271.2411.

**EI-HRMS of 40c:**

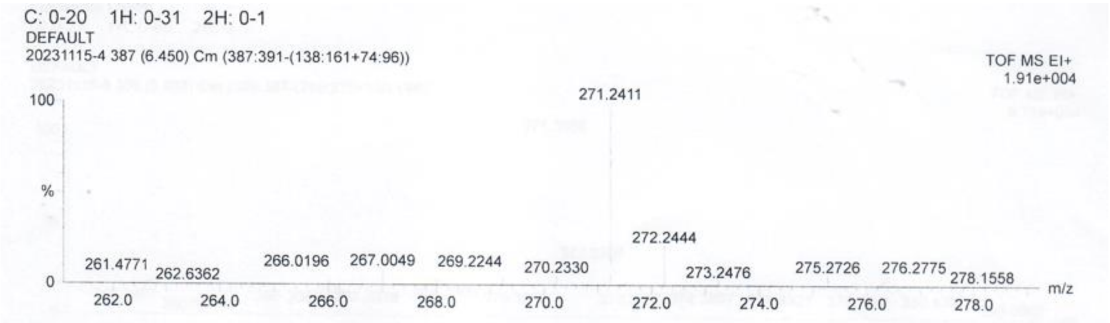


Minimum: 4.00 -1.5
Maximum: 100.00 30.0 10.0 50.0

| Mass | RA | Calc. Mass | mDa | PPM | DBE | i-FIT | Formula |
|---|---|---|---|---|---|---|---|
| 270.2330 | 4.66 | 270.2348 | -1.8 | -6.7 | 6.0 | 11360.0 | C20 1H30 |
| | | 270.2332 | -0.2 | -0.7 | 6.5 | 11541.1 | C20 1H28 2H |
| 271.2411 | 100.00 | 271.2426 | -1.5 | -5.5 | 5.5 | 9.8 | C20 1H31 |
| | | 271.2410 | 0.1 | 0.4 | 6.0 | 575.0 | C20 1H29 2H |

## 6.4 Ten-gram-scale Preparation of 3b without Column Chromatography

To a 1 L vial with a stirring bar was added tryptamine (**3a**) (10.41 g, 65.0 mmol, 1.0 equiv), $K_2CO_3$ (19.76 g, 143.0 mmol, 2.2 equiv), THF (325 mL) and $H_2O$ (325 mL) under Argon. DPPH (33.35 g, 143.0 mmol, 2.2 equiv) was then added portionwise over 1 h. The reaction was stirred at 50 °C for 36 h. When the starting material was completely consumed as evidenced by TLC. Then, the reaction was cooled to room temperature. The mixture was extracted with EA (200 mL for three consecutive times). The combined organic layers were washed three times with 1M aqueous HCl solution and 10% aqueous $K_2CO_3$ solution consecutively. The combined organic layer was washed with brine and dried over anhydrous $Na_2SO_4$, concentrated *in vacuo* to afford 9.33 g of crude **3b** (99% yield, see the crude $^1$H NMR spectrum below). The crude product was purified by column chromatography to isolate 9.03g of **3b** (96%).

**Figure S8: $^1$H NMR spectrum of crude 3b**

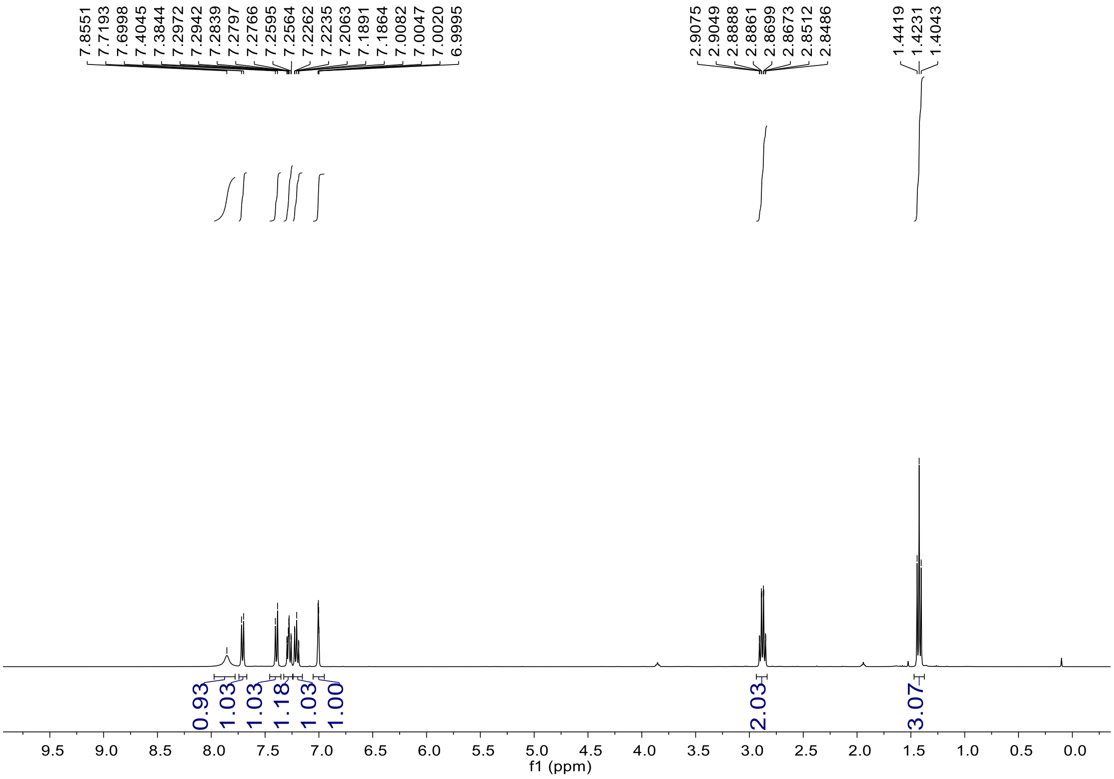

## 6.5 Gram-scale Preparation of Abietratriene (40b) without Column-Chromatography

To a 100 mL vial with a stirring bar was added leelamine (**40a**) (1.11 g, 4.0 mmol, 1.0 equiv), $K_2CO_3$ (1.22 g, 8.8 mmol, 2.2 equiv), THF (20 mL) and $H_2O$ (20 mL) under Argon. DPPH (2.00 g, 8.8 mmol, 2.2 equiv) was then added portionwise over 15 min. The vial was sealed up and stirred at 50 °C for 3 h. When the starting material was completely consumed as evidenced by TLC, the reaction was cooled to room temperature. The mixture was extracted with PE (20 mL for three consecutive times). The combined organic layers were washed three times with 1 M aqueous HCl and 10% aqueous $K_2CO_3$ solution consecutively. The combined organic layers were washed with brine and dried over anhydrous $Na_2SO_4$, concentrated *in vacuo* to afford 1.05 g of abietratriene (**40b**) (97% yield, see the $^1$H NMR spectrum of crude **40b** below). The crude product was further purified by flash chromatography over silica gel to afford 0.98 g of compound **40b** (91% yield).

**Figure S9: $^1$H NMR spectrum of crude abietratriene 40b**

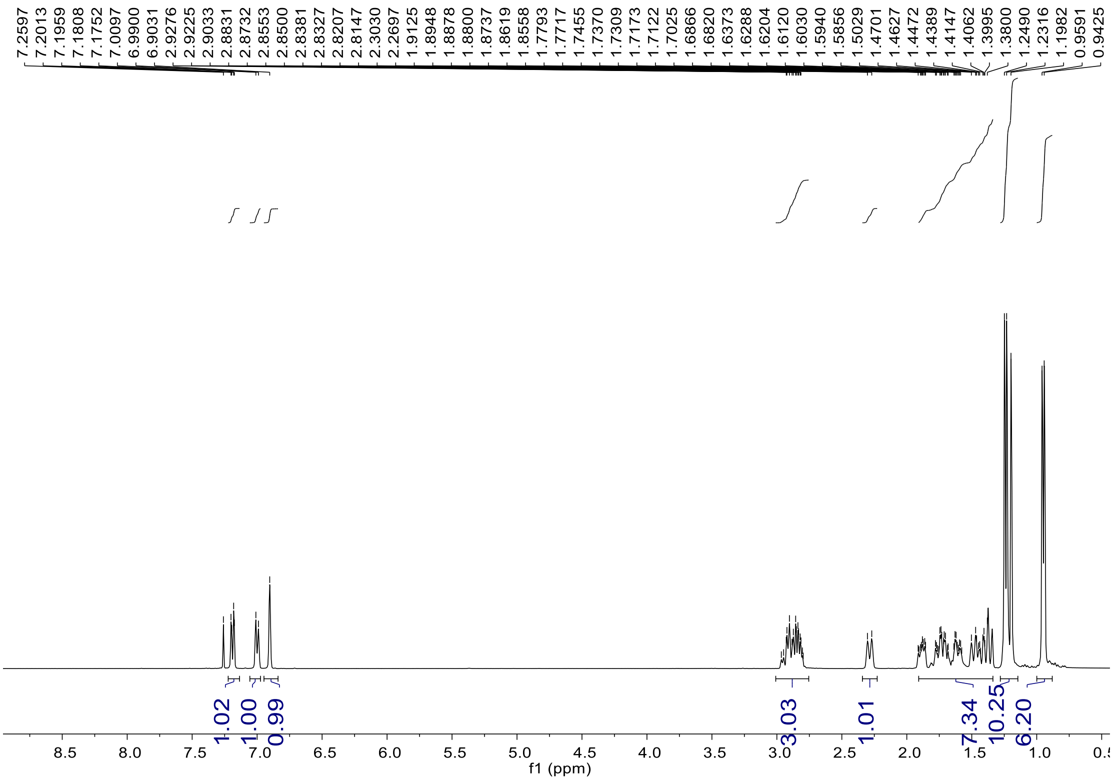

## 6.6 C-H Functionalization & N-deletion Synthetic Sequence

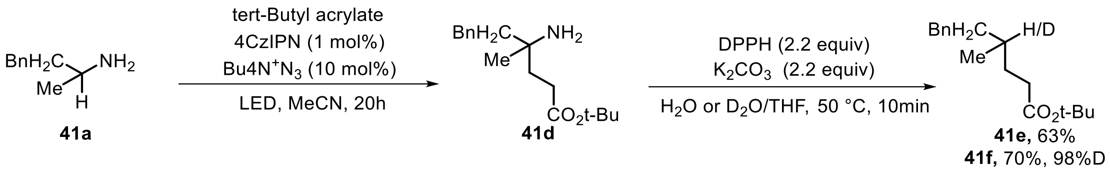


To a 25 mL Schlenk tube with a stirring bar, 4-phenylbutan-2-amine **41a** (72 μL, 67.0 mg, 0.45 mmol, 1.0 equiv), *tert*-butyl acrylate (66 μL, 57.7 mg, 0.45 mmol, 1.0 equiv), 4CzIPN (2.28 mM in MeCN, 1.98 mL, 4.5 μmol, 1 mol%), tetrabutylammonium azide (70.3 mM in MeCN, 640 μL, 45 μmol, 10 mol%), and MeCN (390 μL) were added. The tube was reacted for 20 h with a 425 nm LED lamp. The solution was concentrated *in vacuo*, the residue was dissolved in THF (2.25 mL, 3.75 mL for deuteration), $H_2O$ (2.25 mL, 3.75 mL of $D_2O$ for deuteration). $K_2CO_3$ (136.8 mg, 0.99 mmol, 2.2 equiv), DPPH (230.9 mg, 0.99 mmol, 2.2 equiv) was then added orderly. The vial was sealed up (argon for deuteration), placed on a heating module preheated to 50 °C, and kept stirring at the same temperature vigorously (800 rpm) for 10 min. The reaction was cooled to room temperature, diluted with 5 mL of NaCl aq., extracted three times with 5 mL of EA. The organic layers were combined, dried over anhydrous $Na_2SO_4$, filtrated, concentrated *in vacuo* and purified with flash chromatography over silica gel to afford 74.4 mg of desired product **41e** (63% yield) or 83.0 mg of desired deutero-product **41f** (70% yield, 98%D). The deuterium incorporation was determined by ESI-HRMS.

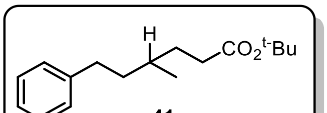


**41e**

**TLC**: $R_f$ = 0.40 (eluent: PE/EA = 20/1, visualized by PMA stain).

**$^1$H NMR** (400 MHz, Chloroform-*d*) δ 7.28 – 7.24 (m, 2H), 7.21 – 7.13 (m, 3H), 2.73 – 2.54 (m, 2H), 2.29 – 2.14 (m, 2H), 1.71 – 1.60 (m, 2H), 1.48 – 1.42 (m, 12H), 0.94 (d, *J* = 6.0 Hz, 3H).

**$^{13}$C NMR** (101 MHz, Chloroform-*d*) δ 173.3, 142.8, 128.3, 128.3, 125.6, 79.9, 38.6,

33.4, 33.3, 32.1, 32.0, 28.1, 19.2.

**HRMS** m/z (ESI) calcd. for $C_{17}H_{26}NaO_2^+$ (M + Na$^+$) 285.1825, found 285.1830.

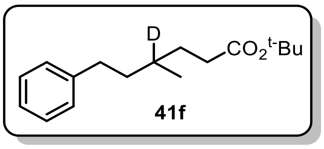


**41f**

**TLC**: $R_f$ = 0.40 (eluent: PE/EA = 20/1, visualized by PMA stain).

**$^1$H NMR** (400 MHz, Chloroform-*d*) δ 7.35 – 7.29 (m, 2H), 7.27 – 7.15 (m, 3H), 2.78 – 2.56 (m, 2H), 2.36 – 2.20 (m, 2H), 1.77 – 1.65 (m, 2H), 1.54 – 1.47 (m, 11H), 0.99 (s, 3H).

**$^{13}$C NMR** (101 MHz, Chloroform-*d*) δ 173.3, 142.8, 128.3, 128.3, 125.6, 79.9, 38.5, 33.3, 33.3, 31.9, 31.6 (t, *J* = 19.3 Hz), 28.1, 19.1.

**HRMS** m/z (ESI) calcd. for $C_{17}H_{25}DNaO_2^+$ (M + Na$^+$) 286.1888, found 286.1895.

**ESI-HRMS of 41f:**

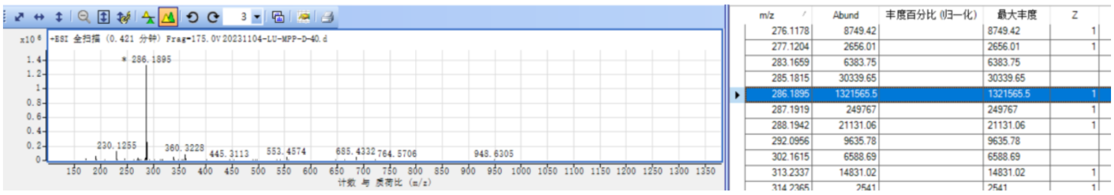


| m/z | Abund | 丰度百分比 (归一化) | 最大丰度 | Z |
|---|---|---|---|---|
| 276.1178 | 8749.42 | | 8749.42 | 1 |
| 277.1204 | 2656.01 | | 2656.01 | 1 |
| 283.1659 | 6383.75 | | 6383.75 | |
| 285.1815 | 30339.65 | | 30339.65 | |
| 286.1895 | 1321565.5 | | 1321565.5 | 1 |
| 287.1919 | 249767 | | 249767 | 1 |
| 288.1942 | 21131.06 | | 21131.06 | 1 |
| 292.0956 | 9635.78 | | 9635.78 | |
| 302.1615 | 6588.69 | | 6588.69 | |
| 313.2337 | 14831.02 | | 14831.02 | 1 |
| 314.2365 | 2541 | | 2541 | 1 |

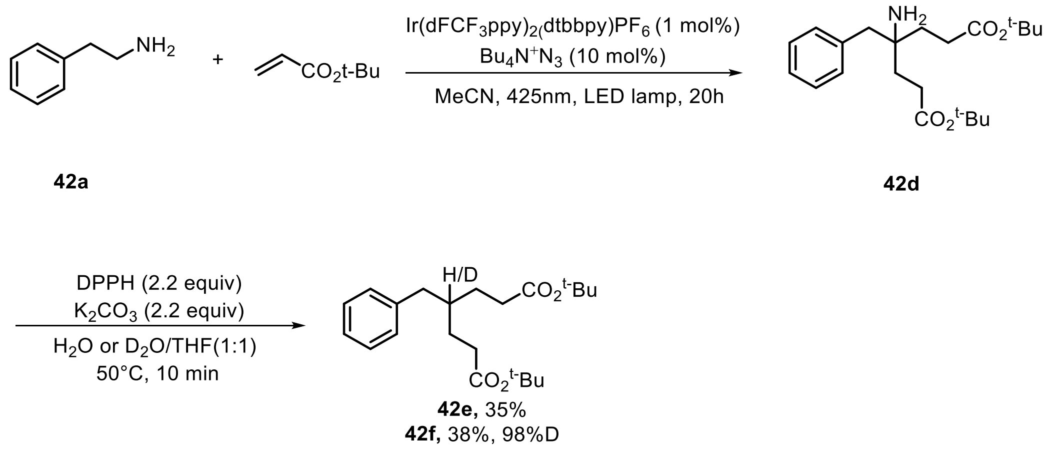


To a 25 mL Schlenk tube with a stirring bar, 2-phenethylamine **42a** (55.3 mg, 0.45 mmol, 1.0 equiv), 2-methoxyethylacrylate (179.4 mg, 1.35 mmol, 3.0 equiv), Ir[dF(CF$_3$)ppy]$_2$(dtbbpy))PF$_6$ (2.23 mM in MeCN, 2.02 mL, 4.5 μmol, 1 mol%), tetrabutylammonium azide (70.3 mM in MeCN, 640 μL, 45 μmol, 10 mol%), and

MeCN (340 μL) were added. The tube was irradiated for 20 h with a 425 nm LED lamp. The solution was concentrated *in vacuo*, the residue was dissolved in THF (2.25 mL, 3.75 mL for deuteration)), $H_2O$ (2.25 mL) {$D_2O$ (3.75 mL) for deuteration}. $K_2CO_3$ (136.8 mg, 0.99 mmol, 2.2 equiv), DPPH (230.9 mg, 0.99 mmol, 2.2 equiv) was then added orderly. The vial was sealed up (argon for deuteration), placed on a heating module preheated to 50 °C, and kept stirring at the same temperature vigorously (800 rpm) for 10 min. The reaction was cooled to room temperature, diluted with 5 mL of NaCl aq., extracted three times with 5 mL of EA. The organic layers were combined, dried over anhydrous $Na_2SO_4$, concentrated *in vacuo* and purified with flash chromatography over silica gel to afford 57.1 mg of desired product **42e** (35% yield over two steps) or 62.2 mg of desired deutero-product **42f** (38% yield over two steps, 98%D). The deuterium incorporation was determined by ESI-HRMS.

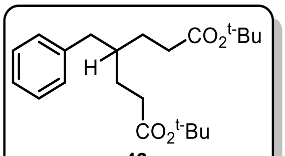

**42e**

**TLC**: $R_f$ = 0.60 (eluent: PE/EA = 10/1, visualized by PMA stain).

**$^1$H NMR** (400 MHz, Chloroform-*d*) δ 7.32 – 7.27 (m, 2H), 7.23 – 7.14 (m, 3H), 2.57 (d, *J* = 6.7 Hz, 2H), 2.37 – 2.16 (m, 5H), 1.44 (s, 18H).

**$^{13}$C NMR** (101 MHz, Chloroform-*d*) δ 173.0, 140.6, 129.1, 128.3, 125.9, 80.1, 40.1, 38.8, 35.2, 32.8, 28.2, 28.1, 24.5.

**HRMS** m/z (ESI) calcd. for $C_{22}H_{34}NaO_4^+$ (M + $Na^+$) 385.2349, found 385.2350.

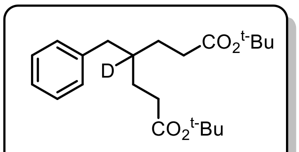

**42f**

**TLC**: $R_f$ = 0.60 (eluent: PE/EA = 10/1, visualized by PMA stain).

**$^1$H NMR** (400 MHz, Chloroform-*d*) δ 7.30 – 7.25 (m, 2H), 7.21 – 7.11 (m, 3H), 2.54 (s, 2H), 2.30 – 2.18 (m, 4H), 1.61 – 1.53 (m, 4H), 1.42 (s, 18H).

**$^{13}$C NMR** (101 MHz, Chloroform-*d*) δ 173.0, 140.6, 129.1, 128.3, 125.9, 80.1, 39.9, 38.3 (t, *J* = 18.9 Hz), 35.2, 32.8, 28.1, 24.5.

**HRMS** m/z (ESI) calcd. for $C_{22}H_{33}DNaO_4^+$ (M + Na$^+$) 386.2412, found 386.2423.

**ESI-HRMS of 42f:**

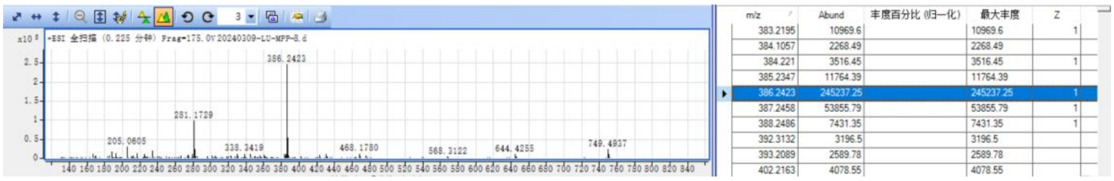


| m/z | Abund | 丰度百分比 (归一化) | 最大丰度 | Z |
|---|---|---|---|---|
| 383.2195 | 10969.6 | | 10969.6 | 1 |
| 384.1057 | 2268.49 | | 2268.49 | |
| 384.221 | 3516.45 | | 3516.45 | 1 |
| 385.2347 | 11764.39 | | 11764.39 | |
| 386.2423 | 245237.25 | | 245237.25 | 1 |
| 387.2458 | 53855.79 | | 53855.79 | 1 |
| 388.2486 | 7431.35 | | 7431.35 | 1 |
| 392.3132 | 3196.5 | | 3196.5 | |
| 393.2089 | 2589.78 | | 2589.78 | |
| 402.2163 | 4078.55 | | 4078.55 | |

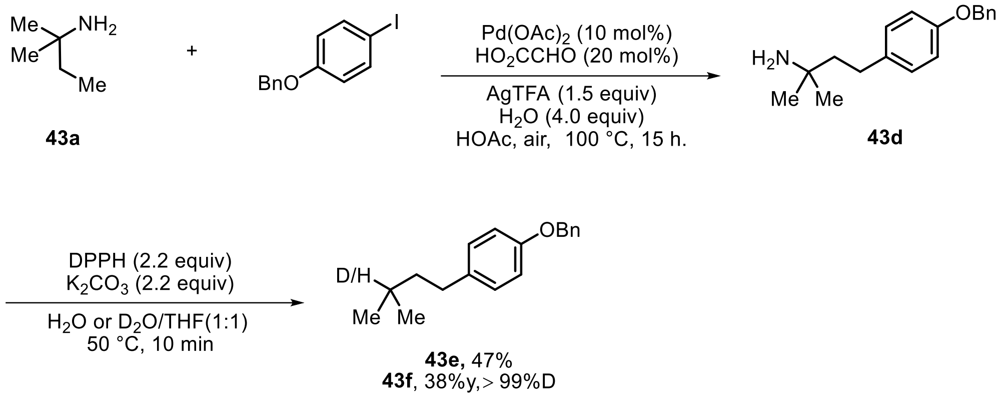


To a 25 mL Schlenk tube with a stirring bar were added $Pd(OAc)_2$ (6.7 mg, 0.03 mmol), glyoxylic acid monohydrate (5.5 mg, 0.06 mmol), AgTFA (99.4 mg, 0.45 mmol), HOAc (2 mL), *tert*-amylamine (26.1 mg, 0.3 mmol), iodobenzene (91.8 mg, 0.45 mmol) and $H_2O$ (21.6 μL, 1.2 mmol). The tube was then sealed, and the reaction mixture was stirred at room temperature for 15 min before heated to 100 ˚C for 15 h. The mixture was cooled to room temperature and concentrated under reduced pressure. The residue was dissolved in THF (2.25 mL, 3.75 mL for deuteration) and $H_2O$ (2.25 mL, 3.75 mL of $D_2O$ for deuteration), $K_2CO_3$ (136.8 mg, 0.99 mmol, 2.2 equiv), DPPH (230.9 mg, 0.99 mmol, 2.2 equiv) was then added orderly. The vial was sealed up (argon for deuteration), placed on a heating module preheated to 50 °C, and kept stirring at the same temperature vigorously (800 rpm) for 10 min. The reaction was cooled to room temperature, diluted with 5 mL of NaCl aq., extracted three times with 5 mL of EA. The organic layers were combined, dried over anhydrous $Na_2SO_4$, concentrated *in vacuo* and purified with flash chromatography over silica gel to afford 53.8 mg of

desired product **43e** (47% yield) or 43.7 mg of desired deutero-product **43f** (38% yield, 99%D). The deuterium incorporation was determined by ESI-HRMS.

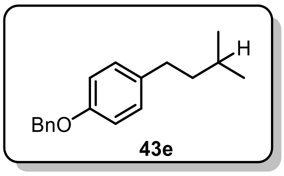

**TLC**: $R_f$ = 0.60 (eluent: PE/EA = 10/1, visualized by PMA stain).

**$^1$H NMR** (400 MHz, Chloroform-d) δ 7.47 – 7.32 (m, 5H), 7.12 (d, *J* = 8.5 Hz, 2H), 6.92 (d, *J* = 8.5 Hz, 2H), 5.06 (s, 2H), 2.64 – 2.53 (m, 2H), 1.64 – 1.46 (m, 3H), 0.95 (d, *J* = 6.6 Hz, 6H).

**$^{13}$C NMR** (101 MHz, Chloroform-d) δ 156.8, 137.3, 135.5, 129.2, 128.6, 127.9, 127.5, 114.7, 70.1, 41.1, 32.9, 27.6, 22.6.

**HRMS** m/z (ESI) calcd. for $C_{18}H_{22}NaO^+$ (M + $Na^+$) 277.1563, found 277.1550.

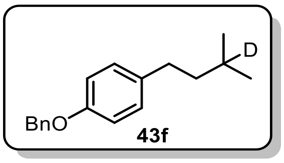

**TLC**: $R_f$ = 0.60 (eluent: PE/EA = 10/1, visualized by PMA stain).

**$^1$H NMR** (400 MHz, Chloroform-*d*) δ 7.52 – 7.33 (m, 5H), 7.16 (d, *J* = 8.5 Hz, 2H), 6.96 (d, *J* = 8.5 Hz, 2H), 5.10 (s, 2H), 2.68 – 2.56 (m, 2H), 1.56 – 1.50 (m, 2H), 0.99 (s, 6H).

**$^{13}$C NMR** (101 MHz, Chloroform-*d*) δ 156.9, 137.4, 135.5, 129.2, 128.5, 127.9, 127.5, 114.8, 70.1, 41.0, 32.9, 27.1 (t, *J* = 19.2 Hz), 22.4.

**HRMS** m/z (ESI) calcd. for $C_{18}H_{21}DNaO^+$ (M + $Na^+$) 277.1626, found 278.1618.

**ESI-HRMS of 43f:**

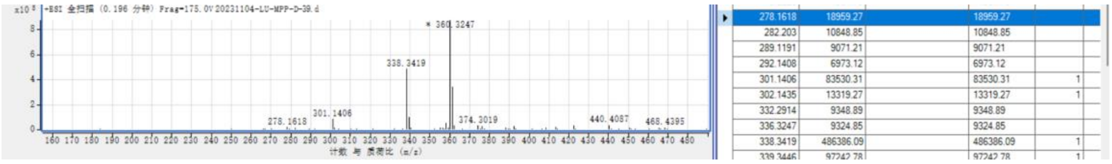

## 6.7 Degree-controlled Deuteration by Using $D_2O$

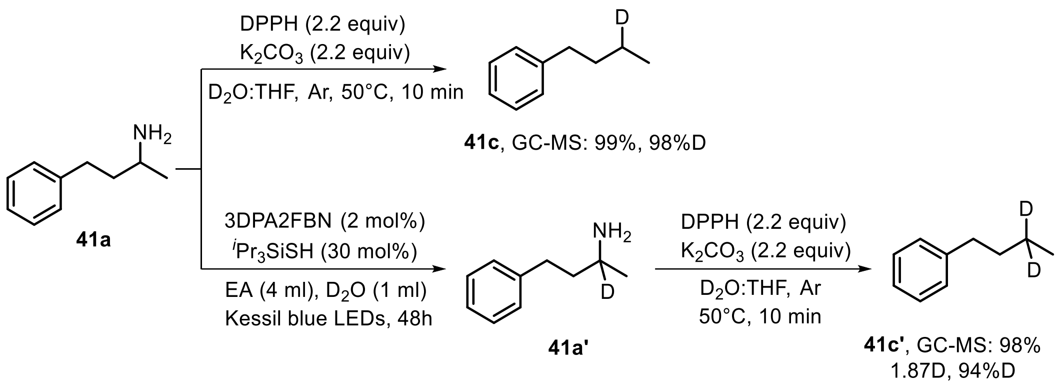


To a 10 mL Schlenk tube equipped with a stirring bar was added 4-phenylbutan-2-amine (29.8 mg, 0.2 mmol, 1.0 equiv), THF (1.67 mL), $D_2O$ (1.67 mL), $K_2CO_3$ (60.8 mg, 0.44 mmol, 2.2 equiv) and DPPH (102.6 mg, 0.44 mmol, 2.2 equiv) under argon. The Schlenk tube was placed on a heating module preheated to 50 °C, and kept stirring at the same temperature vigorously (800 rpm) for 10 min. The reaction was cooled to room temperature, diluted with 2 mL of NaCl aq. and 2 mL of EA. Dodecane (34.1 mg, 0.2 mmol) was added as the internal standard. The yield of **41c** by GC-MS was 99% (98%D). The deuterium incorporation was determined by GC-MS.

**Figure S10: GC-MS of butylbenzene**

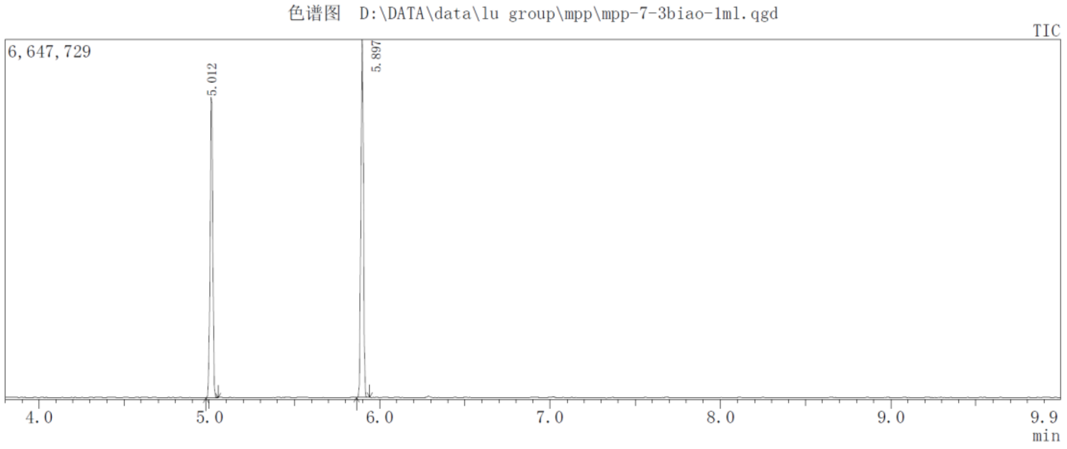


| peak | RT/min | peak area | peak area % | content |
|---|---|---|---|---|
| 1 | 5.012 | 6150800 | 46.99 | Butylbenzene |
| 2 | 5.897 | 6937736 | 53.01 | Dodecane |

**Figure S11: GC-MS of the crude product 41c**

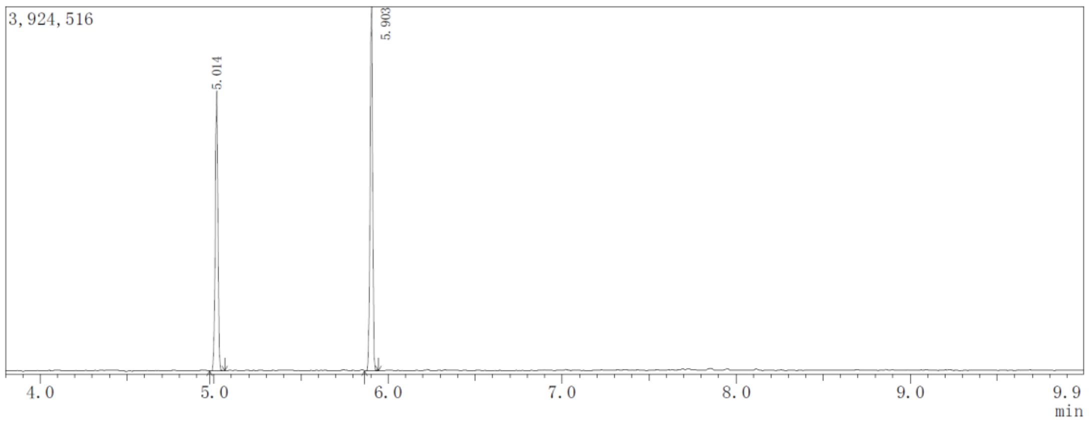


| peak | RT/min | peak area | peak area % | content |
|---|---|---|---|---|
| 1 | 5.014 | 3207108 | 44.24 | **41c** |
| 2 | 5.903 | 4041739 | 55.76 | Ddecane |

To a 10 mL Schlenk tube equipped with a stirring bar was added 4-phenylbutan-2-amine (**41a**) (29.8 mg, 0.2 mmol, 1.0 equiv), photocatalyst 3DPA2FBN (2.6 mg, 2 mol%), triisopropylsilanethiol (12 uL, 30 mol%), anhydrous EA (4 mL), and $D_2O$ (1 mL) under Argon. The solution was then stirred at room temperature under the irradiation of two 40 W Kessil Blue LEDs for 48h. After completion of the reaction, the organic layer was removed, and the aqueous layer was extracted with EA (5 mL × 3). The combined organic extracts were washed with saturated aqueous NaCl solution (15 mL), dried over anhydrous $Na_2SO_4$, filtered and concentrated *in vacuo*. The mixture was dissolved in THF (1.67 mL) and $D_2O$ (1.67 mL). $K_2CO_3$ (60.8 mg, 0.44 mmol, 2.2 equiv) and DPPH (102.6 mg, 0.44 mmol, 2.2 equiv) were then added orderly under argon. The vial was sealed up, placed on a heating module preheated to 50 °C, and kept stirring at the same temperature vigorously (800 rpm) for 10 min. The reaction was cooled to room temperature, diluted with 2 mL of NaCl aq. and 2 mL of EA. Dodecane (34.1 mg, 0.2 mmol) was added as the internal standard. The yield of **41c'** by GC-MS was 98% (1.87D, 94%D). The deuterium incorporation was determined by GC-MS.

## Figure S12: GC-MS of the crude product 41c'

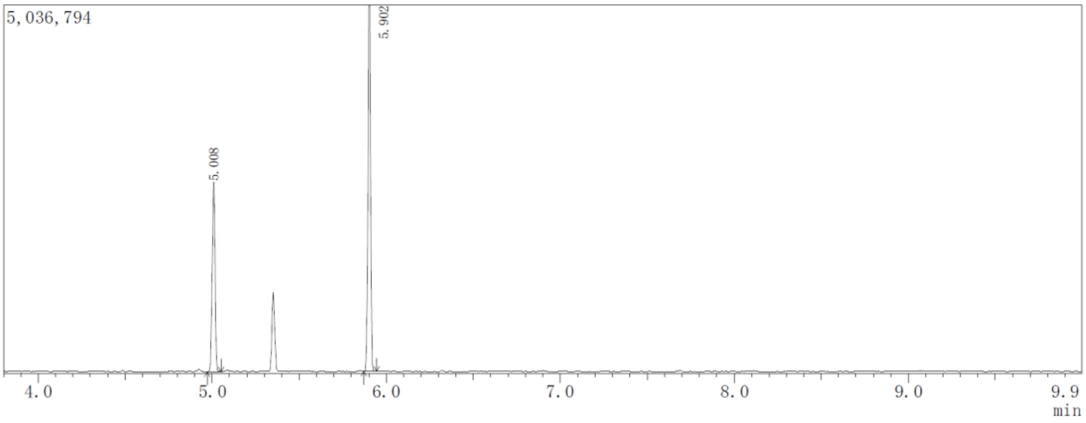


| peak | RT/min | peak area | peak area % | content |
|---|---|---|---|---|
| 1 | 5.008 | 2909483 | 35.21 | **41c'** |
| 2 | 5.902 | 5352997 | 64.79 | Ddecane |

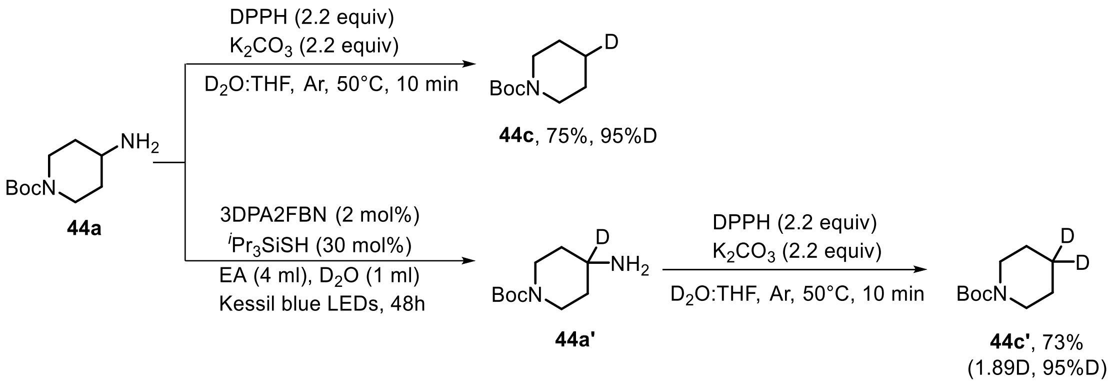


To a 10 mL Schlenk tube equipped with a stirring bar was added *tert*-butyl 4-aminopiperidine-1-carboxylate (40.1 mg, 0.2 mmol, 1.0 equiv), $K_2CO_3$ (60.8 mg, 0.44 mmol, 2.2 equiv), THF (1.67 mL), $D_2O$ (1.67 mL) and DPPH (102.6 mg, 0.44 mmol, 2.2 equiv) under argon. The schlenk tube was placed on a heating module preheated to 50 °C and kept stirring at the same temperature vigorously (800 rpm) for 10 min. The reaction was cooled to room temperature, diluted with 2 mL of NaCl aq and 2 mL of EA. The organic layers were combined, dried over anhydrous $Na_2SO_4$, concentrated *in vacuo* and purified with flash chromatography over silica gel to afford 27.9 mg of **44c** with 75% yield (95%D). The deuterium incorporation was determined by ESI-HRMS.

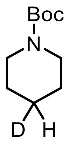


**44c**

**TLC**: $R_f$ = 0.30 (eluent: PE/EA = 10/1, visualized by $KMnO_4$ stain).

**$^1$H NMR** (400 MHz, Chloroform-*d*) δ 3.39 – 3.28 (m, 4H), 1.44 (m, 14H).

**$^{13}$C NMR** (101 MHz, Chloroform-*d*) δ 154.9, 79.1, 44.6, 28.5, 25.6, 24.1 (t, *J* = 19.5 Hz).

**HRMS** m/z (ESI) calcd. for $C_{10}H_{18}DNNaO_2^+$ (M + $Na^+$) 209.1371, found 209.1379.

**ESI-HRMS of 44c:**

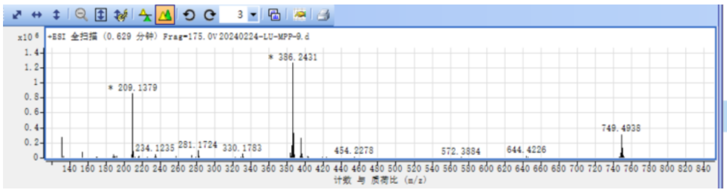


| m/z | Abund | 丰度百分比 (归一化) | 最大丰度 | Z |
|---|---|---|---|---|
| 192.1679 | 2832.22 | | 2832.22 | 1 |
| 196.0946 | 2109.95 | | 2109.95 | |
| 197.096 | 2825.77 | | 2825.77 | |
| 198.1026 | 3865.03 | | 3865.03 | |
| 199.067 | 2452.12 | | 2452.12 | |
| 208.1307 | 44568.47 | | 44568.47 | |
| 209.1379 | 852621.56 | | 852621.56 | 1 |
| 210.1404 | 91459.32 | | 91459.32 | 1 |
| 211.1426 | 8116.85 | | 8116.85 | 1 |
| 213.0761 | 4018.47 | | 4018.47 | |
| 215.1067 | 7597.88 | | 7597.88 | |

To a 10 mL Schlenk tube equipped with a stirring bar was added *tert*-butyl 4-aminopiperidine-1-carboxylate (40.1 mg, 0.2 mmol, 1.0 equiv), photocatalyst 3DPA2FBN (2.6 mg, 2 mol%), triisopropylsilanethiol (12 uL, 30 mol%), anhydrous EA (4 mL) and $D_2O$ (1 mL) under Argon. The solution was then stirred at room temperature under the irradiation of two 40 W Kessil Blue LEDs for 48 h. After completion of the reaction, the organic layer was removed, and the aqueous layer was extracted with EA (5 mL × 3). The combined organic extracts were washed with saturated aqueous NaCl solution (15 mL), dried over anhydrous $Na_2SO_4$, filtered and concentrated *in vacuo*. The mixture was dissolved in THF (1.67 mL), $D_2O$ (1.67 mL), $K_2CO_3$ (60.8 mg, 0.44 mmol, 2.2 equiv) and DPPH (102.6 mg, 0.44 mmol, 2.2 equiv). The vial was sealed up, placed on a heating module preheated to 50 °C, and kept stirring at the same temperature vigorously (800 rpm) for 10 min. The reaction was cooled to room temperature, diluted with 2 mL of NaCl aq and 2 mL of EA. The organic layers were combined, dried over anhydrous $Na_2SO_4$, concentrated *in vacuo* and purified with flash chromatography over silica gel to afford 27.3 mg of **44c'** with 73% yield (1.89D, 95%D). The deuterium incorporation was determined by ESI-HRMS.

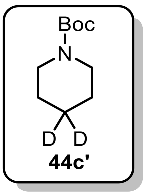


**TLC**: $R_f$ = 0.30 (eluent: PE/EA = 10/1, visualized by $KMnO_4$ stain).

**$^1$H NMR** (400 MHz, Chloroform-*d*) δ 3.40 – 3.31 (m, 4H), 1.46 (m, 13H).

**$^{13}$C NMR** (101 MHz, Chloroform-*d*) δ 154.9, 79.1, 44.6, 28.5, 25.5, 24.73 – 23.27 (m).

**HRMS** m/z (ESI) calcd. for $C_{10}H_{17}D_2NNaO_2^+$ (M + $Na^+$) 210.1434, found 210.1441.

**ESI-HRMS of 44c':**

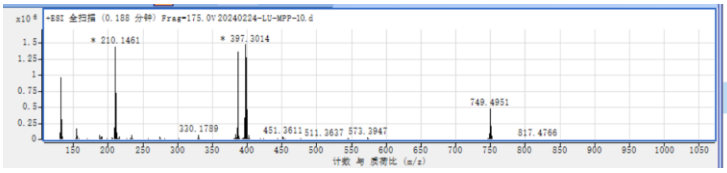


| | | | | | |
|---|---|---|---|---|---|
| 190.1592 | 36755.39 | | 36755.39 | | |
| 191.1651 | 34985 | | 34985 | | |
| 198.1025 | 7919.35 | | 7919.35 | | |
| 205.06 | 30089.66 | | 30089.66 | | |
| 206.0965 | 7273.85 | | 7273.85 | | |
| 209.1375 | 173926.25 | | 173926.25 | | |
| 210.1461 | 1440351.25 | | 1440351.25 | | |
| 211.1498 | 722730.81 | | 722730.81 | 1 | |
| 212.1541 | 98719.53 | | 98719.53 | 1 | |
| 213.1026 | 10336.88 | | 10336.88 | | |
| [illegible] | [illegible] | | [illegible] | 1 | |

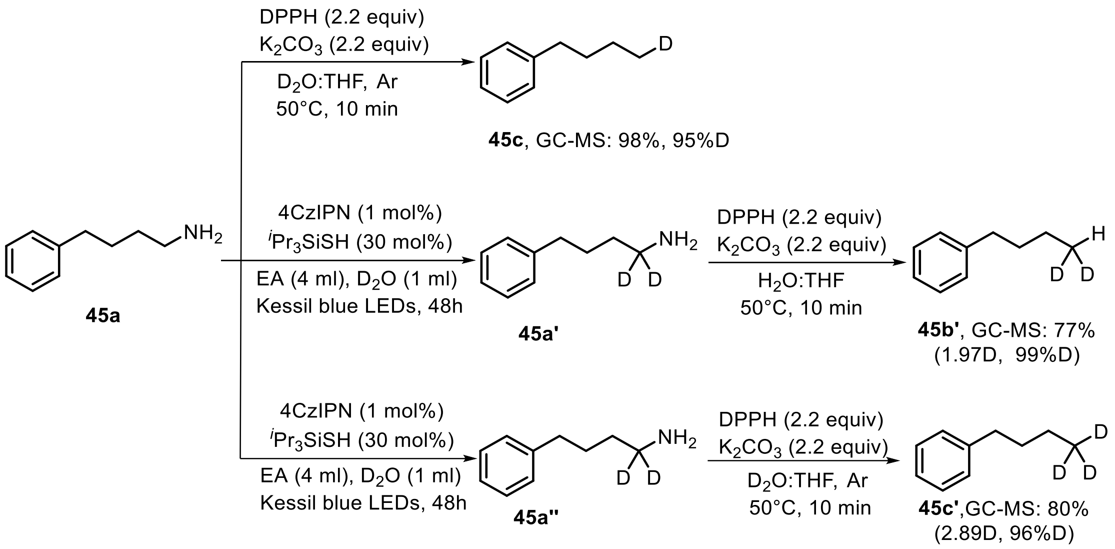


According to the standard conditions, the result of **45c** by GC-MS was 98% yield (95%D). The deuterium incorporation was determined by GC-MS.

**Figure S13: GC-MS of the crude product 45c**

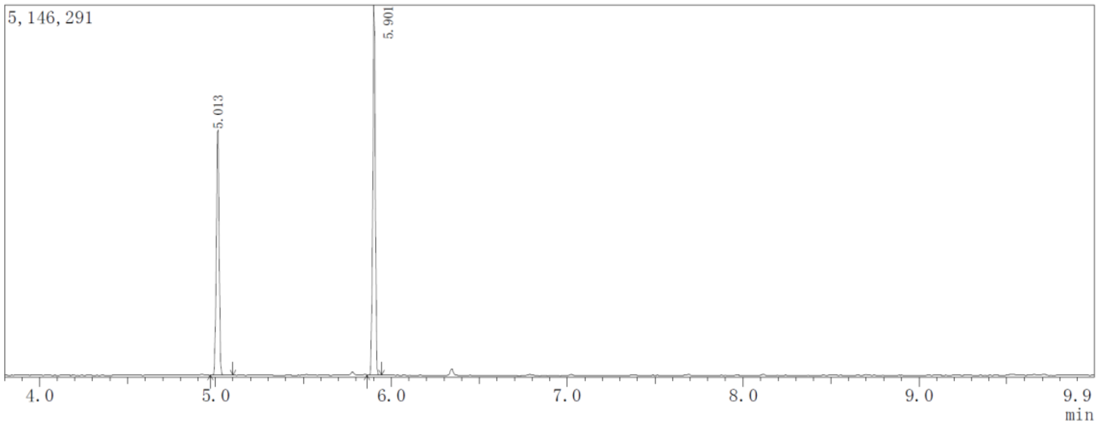


| peak | RT/min | peak area | peak area % | content |
|---|---|---|---|---|
| 1 | 5.013 | 3849603 | 42.37 | **45c** |
| 2 | 5.901 | 5236481 | 57.63 | Ddecane |

To a 25 mL Schlenk tube equipped with a stirring bar was added 4-phenylbutan-1-amine (**45a**) (29.8 mg, 0.2 mmol, 1.0 equiv), photocatalyst 4CzIPN (1.6 mg, 1 mol%), triisopropylsilanethiol (12 µL, 30 mol%), anhydrous EA (4 mL) and $D_2O$ (1 mL) under Argon. The solution was then stirred at room temperature under the irradiation of two 40 W Kessil Blue LEDs for 48 h. After completion of the reaction, the organic layer was removed, and the aqueous layer was extracted with EA (5 mL × 3). The combined organic extracts were washed with saturated aqueous NaCl solution (15 mL), dried over anhydrous $Na_2SO_4$, filtered and concentrated *in vacuo*. The mixture was dissolved in THF (1 mL, 1.67 mL for deuteration)), $H_2O$ (1 mL) {$D_2O$ (1.67 mL) for deuteration}. $K_2CO_3$ (2.2 equiv) and DPPH (102.6 mg, 0.44 mmol, 2.2 equiv) were then added orderly. The vial was sealed up (argon for deuteration), placed on a heating module preheated to 50 °C, and kept stirring at the same temperature vigorously (800 rpm) for 10 min. The reaction was cooled to room temperature, diluted with 2 mL of NaCl aq and 2 mL of EA. Dodecane as internal standard. The result of GC-MS for **45b'**: 77% yield, 1.97D, 99%D; for **45c'**: 80% yield, 2.89D, 96%D. The deuterium incorporation was determined by GC-MS.

## Figure S14: GC-MS of the crude product 45b'

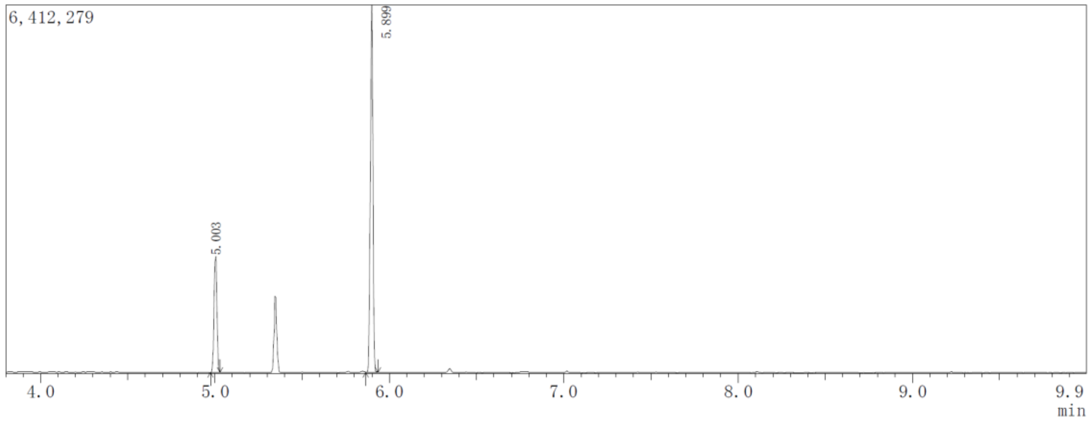


| peak | RT/min | peak area | peak area % | content |
|---|---|---|---|---|
| 1 | 4.975 | 2266804 | 25.82 | **45b'** |
| 2 | 5.865 | 6511382 | 74.18 | Ddecane |

## Figure S15: GC-MS of the crude product 45c'

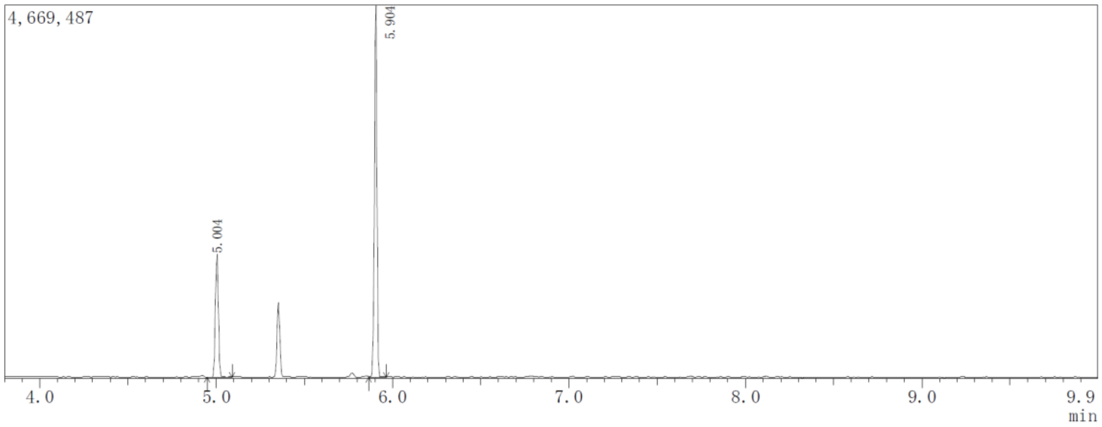


| peak | RT/min | peak area | peak area % | content |
|---|---|---|---|---|
| 1 | 4.950 | 1789092 | 27.39 | **45c'** |
| 2 | 5.865 | 4742052 | 72.61 | Ddecane |

# 7. Mechanistic Studies

## 7.1 Detection of Key Intermediates

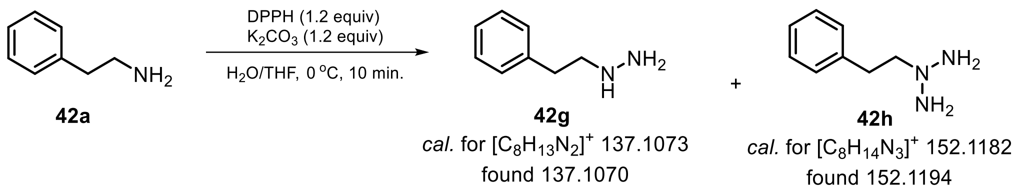


To a 10 mL vial with a stirring bar was added 2-phenylethan-1-amine **42a** (12.01 mg, 0.1 mmol, 1.0 equiv), THF (0.5 mL) and $H_2O$ (0.5 mL). $K_2CO_3$ (30.4 mg, 0.22 mmol, 2.2 equiv) and DPPH (51.3 mg, 0.22 mmol, 2.2 equiv) were then added orderly. The reaction was stirred at 0 °C for 10 min. The stirring was stopped, and a sample of the reaction solution was diluted by methanol that has been cooled to 0 °C in advance. The HRMS test was conducted immediately.

**Figure S16: ESI HRMS of the reaction**

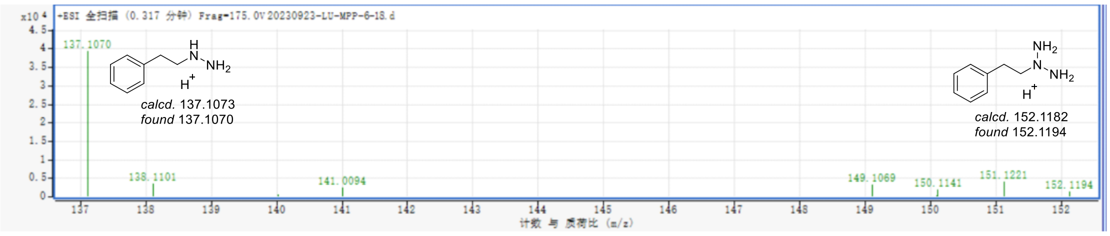


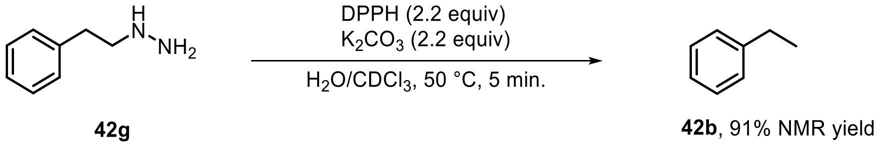


To a 10 mL vial with a stirring bar was added phenethylhydrazine **42g** (13.6 mg, 0.1 mmol, 1.0 equiv), $H_2O$ (0.5 mL) and $CDCl_3$ (0.5 mL). DPPH (51.3 mg, 0.22 mmol, 2.2 equiv) and $K_2CO_3$ (30.4 mg, 0.22 mmol, 2.2 equiv) were then added orderly. The reaction was stirred at 50 °C for 5 min. After the completion of reaction, the aqueous layer was removed by separatory funnel. The aqueous layer was extracted with small amounts of $CDCl_3$ (twice). The organic phases were combined and $CH_2Br_2$ (0.1 mmol) was added as internal standard. The sample was tested with $^1$H NMR (**42b** with 91% NMR yield).

**Figure S17. ¹H NMR of 42b in $CDCl_3$**

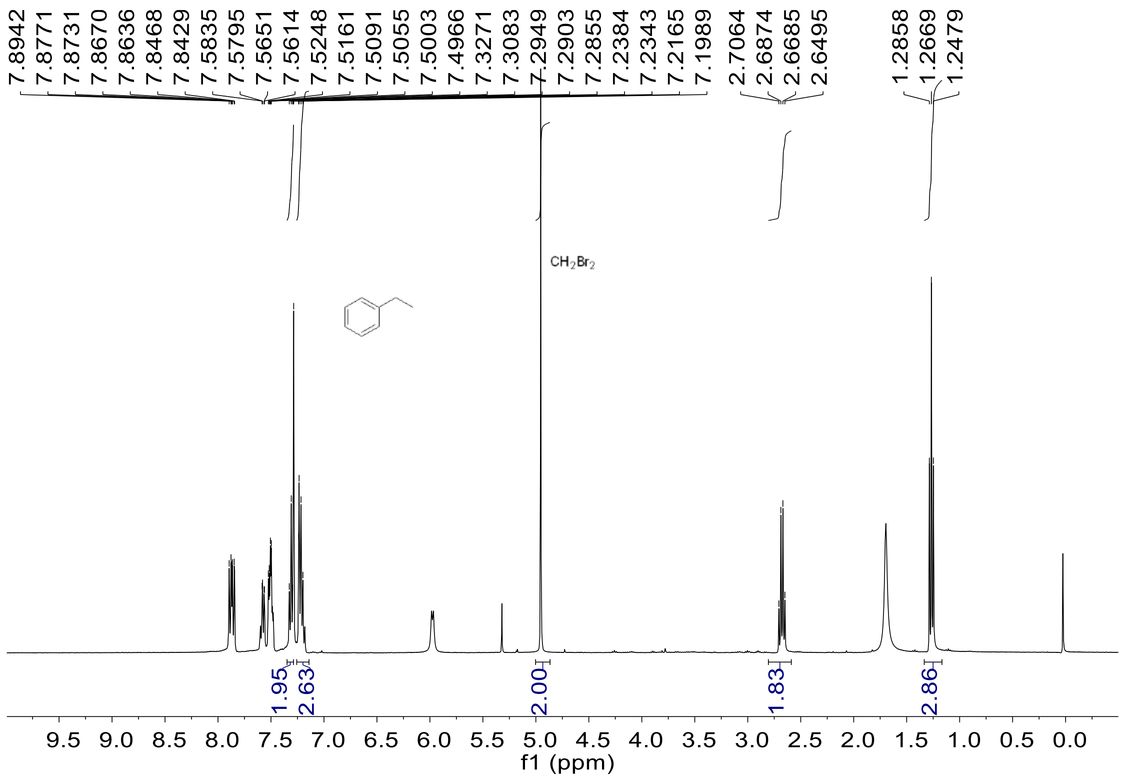


## 7.2 Comparison of Diazene and Isodiazene

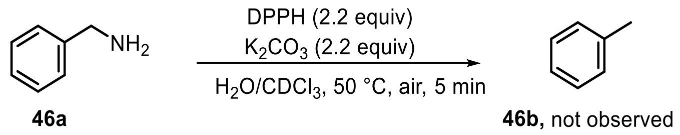


To a 10 mL vial with a stirring bar was added phenylmethanamine (**46a**) (10.7 mg, 0.1 mmol, 1.0 equiv), $CDCl_3$ (0.5 mL), $H_2O$ (0.5 mL). $K_2CO_3$ (30.4 mg, 0.22 mmol, 2.2 equiv) and DPPH (51.3 mg, 0.22 mmol, 2.2 equiv) was then added orderly. The reaction was stirred at 50 °C for 5 min. The reaction was cooled to room temperature, diluted with 5 mL of NaCl aq., extracted three times with 5 mL of DCM. The organic layers were combined, dried over anhydrous $Na_2SO_4$. The crude product was tested by $^1H$ NMR (with 0.1 mmol of $CH_2Br_2$ as the internal standard).

**Figure S18: $^1$H NMR of crude 46b in $CDCl_3$**

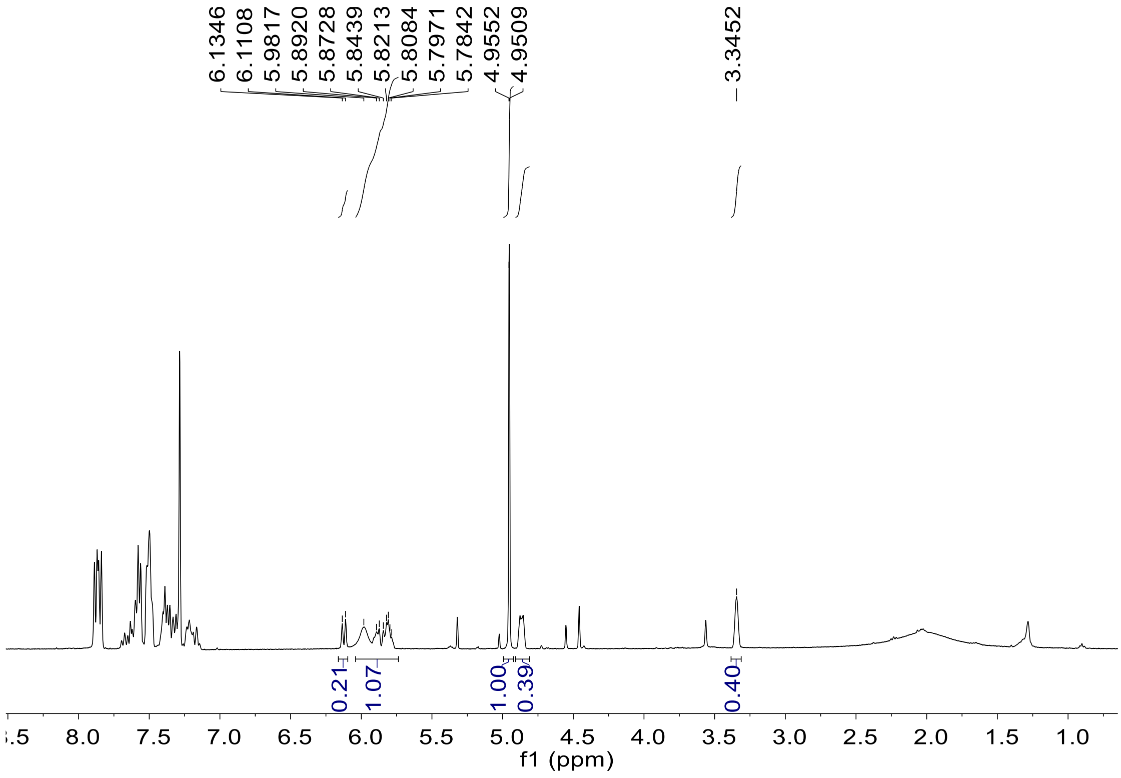


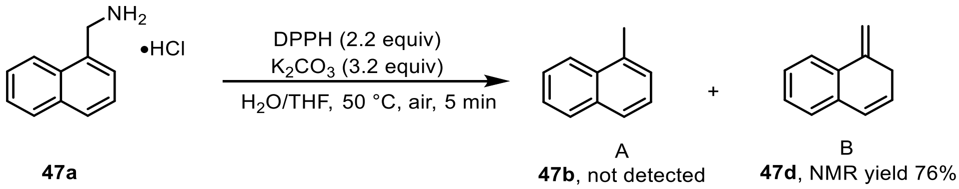


To a 10 mL vial with a stirring bar was added 1-(aminomethyl)naphthalene hydrochloride (**47a**) (19.4 mg, 0.1 mmol, 1.0 equiv), $K_2CO_3$ (44.2 mg, 0.32 mmol, 3.2 equiv), THF (0.5 mL), $H_2O$ (0.5 mL) and DPPH (51.3 mg, 0.22 mmol, 2.2 equiv). The reaction was stirred at 50 °C for 5 min. The reaction was cooled to room temperature, diluted with 5 mL of NaCl aq., extracted three times with 5 mL of EA. The organic layers were combined, dried over anhydrous $Na_2SO_4$. The crude product was tested by $^1$H NMR (with 0.1 mmol of $CH_2Br_2$ as the internal standard).

**Figure S19: $^{1}$H NMR of the reaction mixture for 47a**

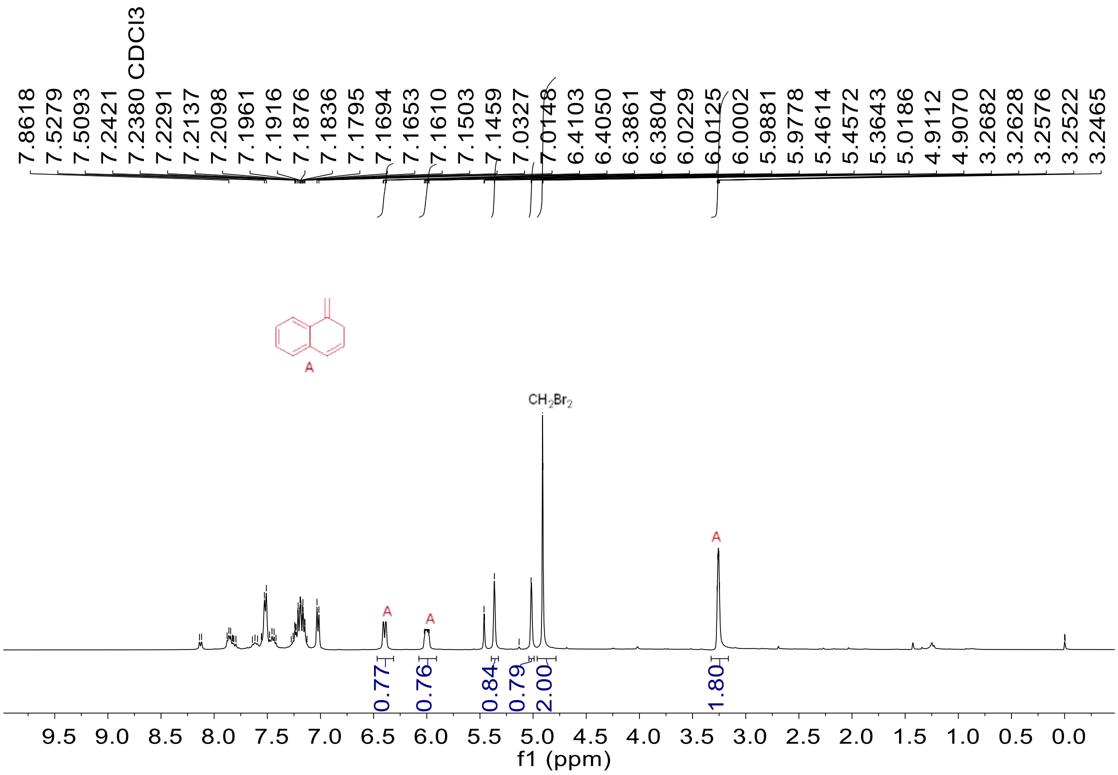

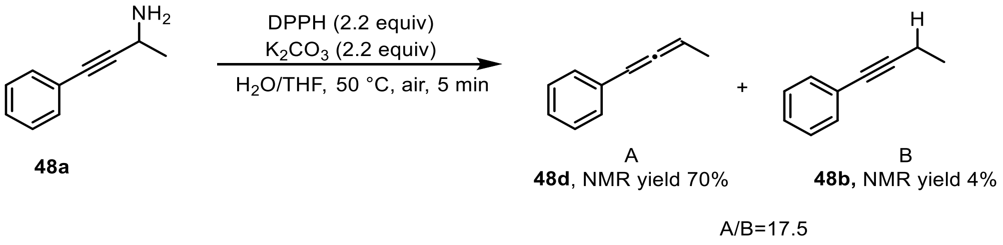


To a 10 mL vial with a stirring bar was added 4-phenylbut-3-yn-2-amine (14.5 mg, 0.1 mmol, 1.0 equiv) THF (0.5 mL) and $H_2O$ (0.5 mL). $K_2CO_3$ (30.4 mg, 0.22 mmol, 2.2 equiv) and DPPH (51.3 mg, 0.22 mmol, 2.2 equiv) was then added orderly. The reaction was stirred at 50 °C for 5 min. The reaction was cooled to room temperature, diluted with 5 mL of NaCl aq., extracted three times with 5 mL of DCM. The organic layers were combined, dried over anhydrous $Na_2SO_4$. The crude product was tested by $^{1}$H NMR (with 0.1 mmol of $CH_2Br_2$ as the internal standard). The same process was repeated three times (A/B=17.5 ± 0.5/1).

## Figure S20: $^1H$ NMR of the reaction mixture for 48a

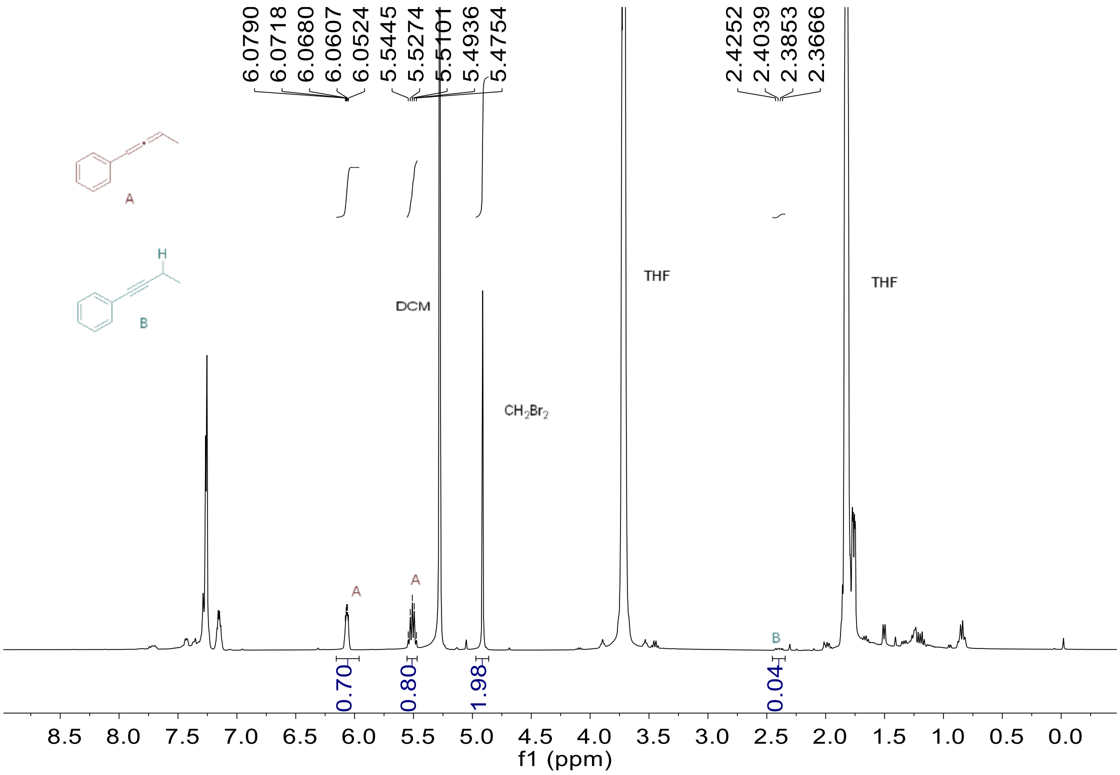


# 7.3 Kinetic Isotope Effect (KIE) Experiments

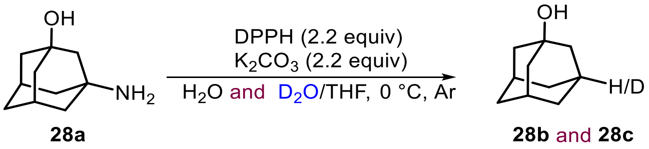


To a 10 mL vial with a stirring bar was added (1s,3r,5r,7s)-3-aminoadamantan -1-ol **28a** (16.7 mg, 0.10 mmol, 1.0 equiv), $K_2CO_3$ (30.4 mg, 0.22 mmol, 2.2 equiv), THF (0.5 mL), $H_2O$ (2.49 mL), $D_2O$ (2.51 mL) and DPPH (51.3 mg, 0.22 mmol, 2.2 equiv). The reaction was stirred at 0 °C. The first sample was taken at 10 minutes, and every 5 minutes afterwards. When sampling, 10 μL of the reaction mixture was extracted and added to a mixture of 1 mL of saturated NaCl aqueous and 1 mL of EA at -20°C, well shaken, and dilute 0.5 mL of the supernatant into a 1.5 mL GC vial, diluted to 1.0 mL with EA. The sample was test by GC-MS. The KIE was $K_H/ K_D$ =1.06.

| T/min | 10min | 15min | 20min | 25min | 30min | 35min | 40min |
|---|---|---|---|---|---|---|---|
| $K_H/ K_D$ | 1.12 | 1.00 | 1.02 | 1.06 | 1.11 | 1.08 | 1.04 |

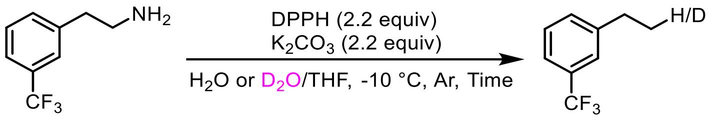


**49a** **49b** or **49c**

To a 10 mL vial with a stirring bar was added 2-(3-(trifluoromethyl) phenyl) ethan -1-amine **49a** (189.1 mg, 1.0 mmol, 1.0 equiv), $K_2CO_3$ (304.1 mg, 2.2 mmol, 2.2 equiv), THF (5.0 mL), $H_2O$ /$D_2O$ (5.0 mL) and DPPH (513.0 mg, 2.2 mmol, 2.2 equiv). NaCl (1. 0 g) was then added. $PhCF_3$ (146.1 mg, 1.0 mmol) was added as the internal standard. The reaction was stirred at -10 °C. The first sample was taken at 10 minutes, and every 10 minutes afterwards. When sampling, 10 μL of the reaction mixture was extracted, quenched with hydrochloric acid and added 0.5 mL of $CDCl_3$. The product was tested by $^{19}F$ NMR. The KIE was $K_H/ K_D$=1.13.

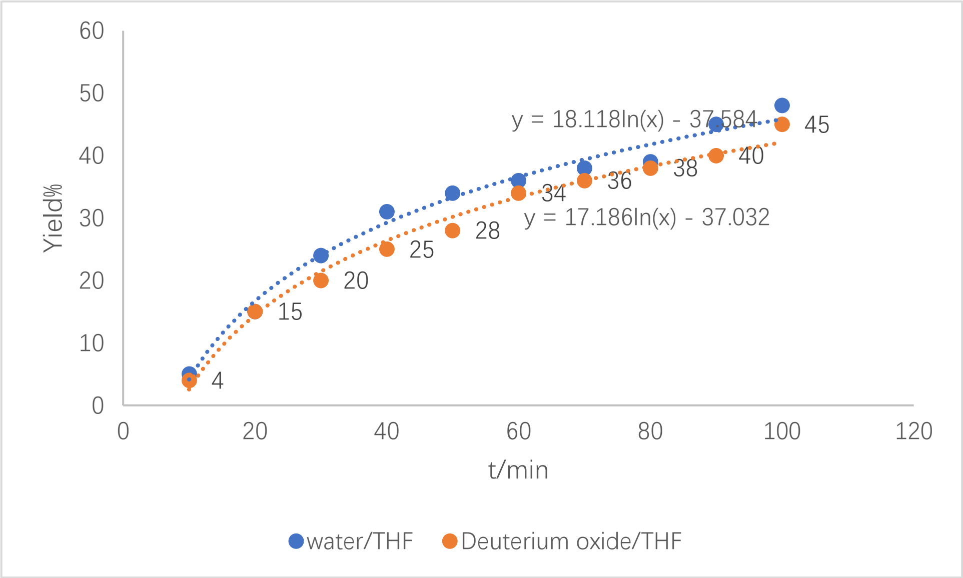

## 7.4 TEMPO Capture Reaction

To a 25 mL vial with a stirring bar was added tryptamine **3a** (64.1 mg, 0.4 mmol, 1.0 equiv), $K_2CO_3$ (121.6 mg, 0.88 mmol, 2.2 equiv), TEMPO (62.5 mg, 0.4 mmol, 1.0 equiv), THF (2.0 mL), $H_2O$ (2.0 mL) and DPPH (205.2 mg, 0.88 mmol, 2.2 equiv). The reaction was stirred at 50 °C for 10 min. The mixture was cooled to room temperature, diluted with 5 mL of NaCl aq., extracted three times with 5 mL of EA. The organic layers were combined, dried over anhydrous $Na_2SO_4$. The crude was finally purified with flash chromatography over silica gel to obtain 3-ethyl-1H-indole **3b** (19.2 mg, 33% yield) and 3-(2-((2,2,6,6-tetramethylpiperidin-1-yl)oxy)ethyl)-1H-indole **3d** (50.5 mg, 42% yield).

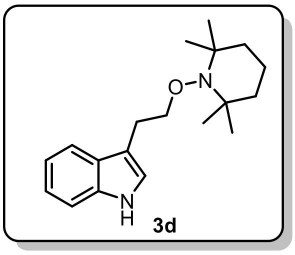

**3-(2-((2,2,6,6-tetramethylpiperidin-1-yl)oxy)ethyl)-1H-indole (3d):**

**TLC**: $R_f$ = 0.40 (eluent: PE/EA = 20/1, visualized by UV light).

**$^1$H NMR** (400 MHz, Chloroform-*d*) δ 7.94 (s, 1H), 7.73 (d, *J* = 8.0 Hz, 1H), 7.39 (d, *J* = 8.0 Hz, 1H), 7.31 – 7.18 (m, 2H), 7.08 (s, 1H), 4.15 (t, *J* = 7.3 Hz, 2H), 3.09 (t, *J* = 7.3 Hz, 2H), 1.69 – 1.33 (m, 6H), 1.23 (d, *J* = 12.8 Hz, 12H).

**$^{13}$C NMR** (101 MHz, Chloroform-*d*) δ 136.2, 127.9, 122.0, 119.3, 113.6, 111.1, 59.8, 39.8, 33.2, 24.6, 20.4, 17.3.

**HRMS** m/z (ESI) calcd. for $C_{19}H_{29}N_2O^+$ (M + $H^+$) 301.2274, found 301.2286.

**Figure S21: $^{1}$H NMR of 3-(2-((2,2,6,6-tetramethylpiperidin-1-yl)oxy)ethyl)-1H-indole (3d) in $CDCl_3$**

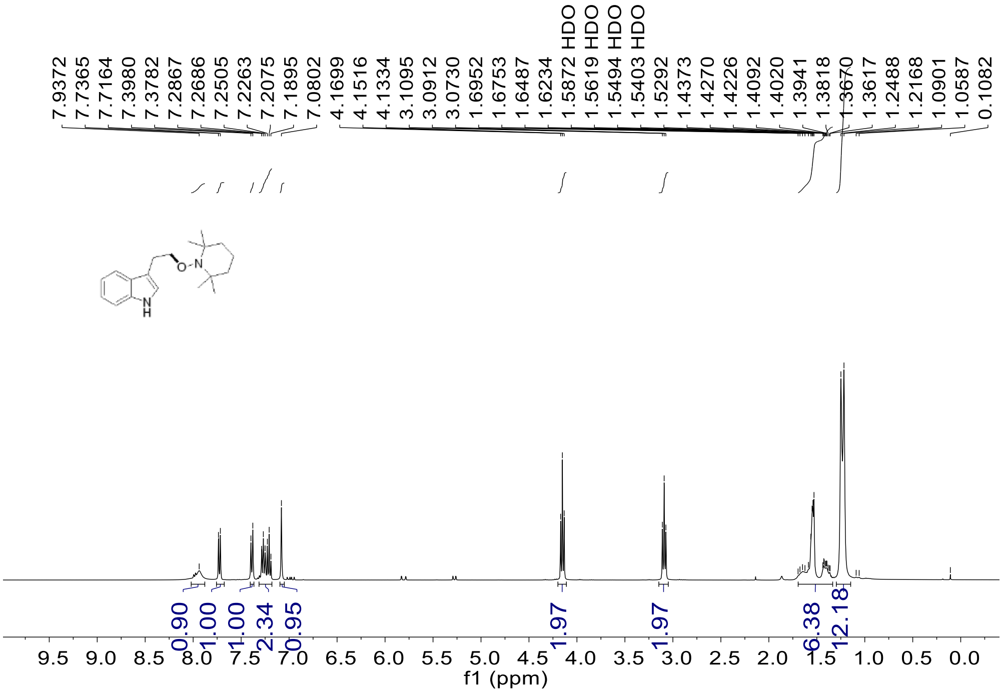


**Figure S22: $^{13}$C NMR of 3-(2-((2,2,6,6-tetramethylpiperidin-1-yl)oxy)ethyl)-1H-indole (3d) in $CDCl_3$**

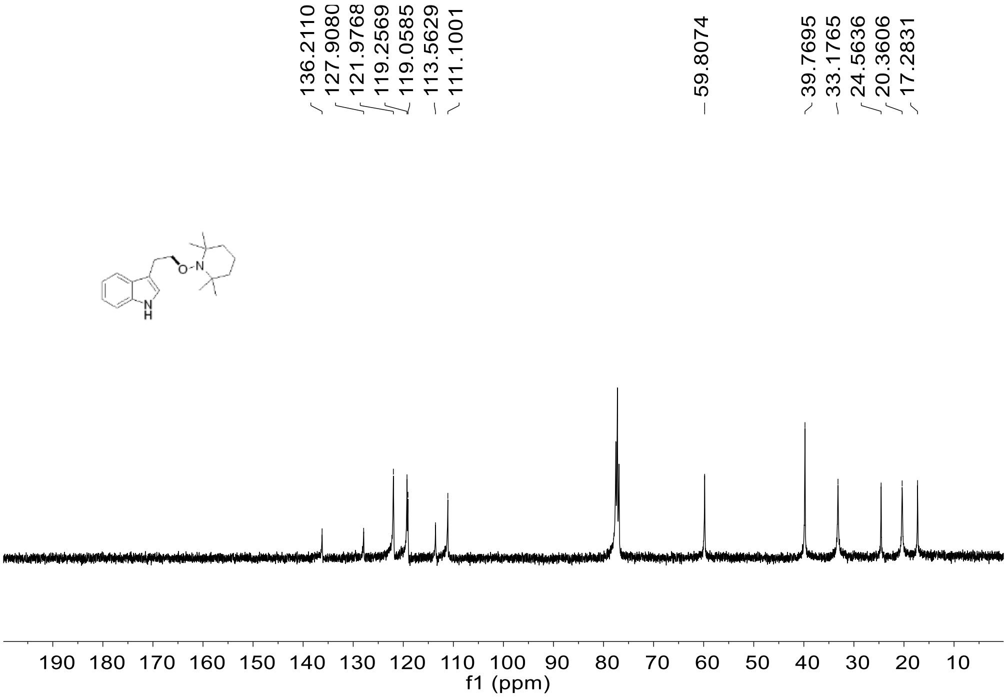

## 7.5 EPR Experiment

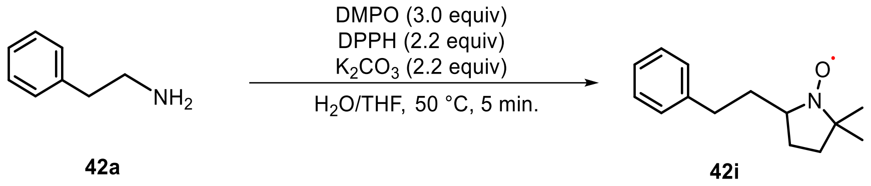


To a 10 mL vial with a stirring bar was added 2-phenylethan-1-amine (**42a**) (12.1 mg, 0.1 mmol, 1.0 equiv), $K_2CO_3$ (30.4 mg, 0.22 mmol, 2.2 equiv), DMPO (33.9 mg, 0.3 mmol, 3.0 equiv), THF (0.5 mL), $H_2O$ (0.5 mL) and DPPH (51.3 mg, 0.22 mmol, 2.2 equiv). The reaction was stirred at 50 °C for 5 min. The stirring was stopped, and cooled to room temperature. The solution was directly applied in EPR experiment. The EPR result matched well with simulation.

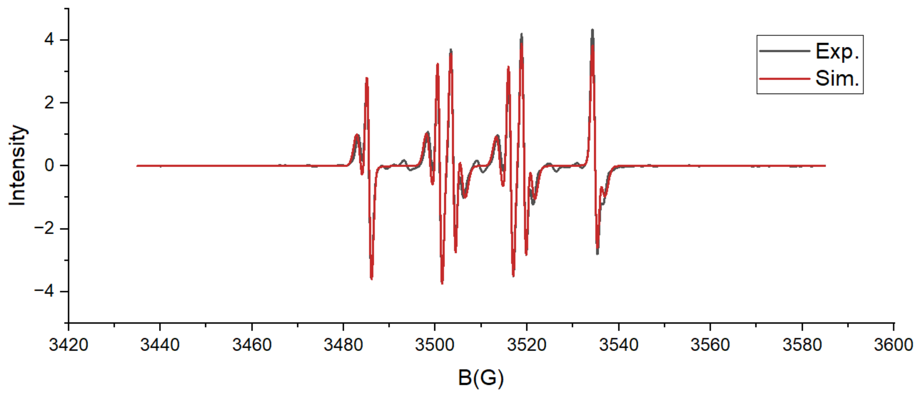


## 7.6 Radical Clock Experiments

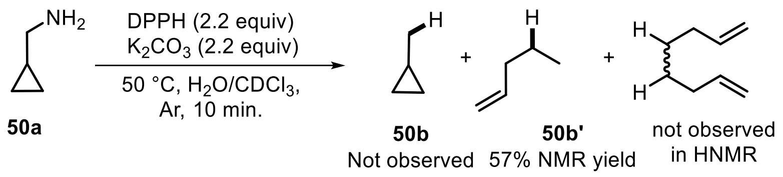


To a 25 mL vial with a stirring bar was added cyclopropylmethanamine (**50a**) (28.4 mg, 0.4 mmol, 1.0 equiv), $K_2CO_3$ (121.6 mg, 0.88 mmol, 2.2 equiv), $CDCl_3$ (2.0 mL), $H_2O$ (2.0 mL) and DPPH (205.2 mg, 0.88 mmol, 2.2 equiv). The reaction was stirred at 50 °C under argon for 10 min. The stirring was stopped, and the aqueous layer was removed by separatory funnel. The aqueous layer was washed with small amount of

CDCl3 (twice). The organic phase was combined. The ring opening product was observed on crude $^1$H NMR (with 0.4 mmol of $CH_2Br_2$ as the internal standard).

**Figure S23: $^1$H NMR of the reaction of 50a in $CDCl_3$**

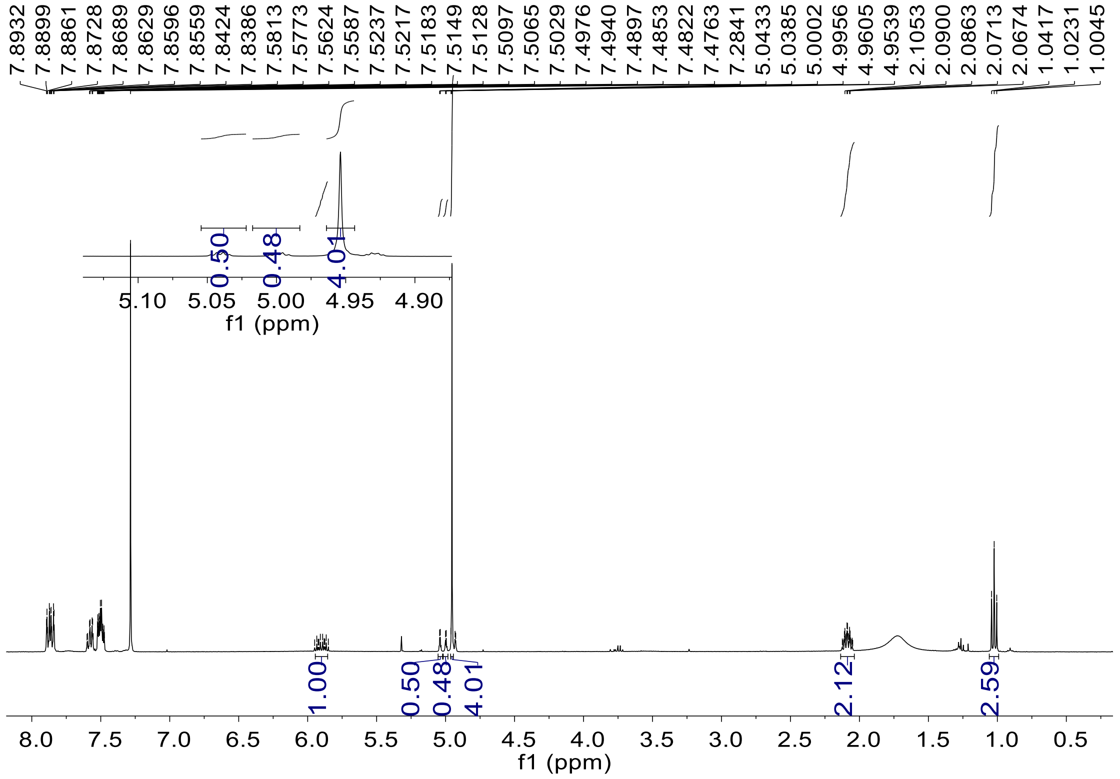


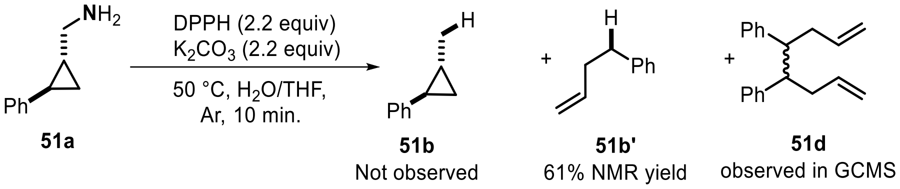


To a 25 mL vial with a stirring bar was added ((1R,2R)-2-phenylcyclopropyl) methanamine (**51a**) (58.9 mg, 0.4 mmol, 1.0 equiv), $K_2CO_3$ (121.6 mg, 0.88 mmol, 2.2 equiv), THF (2.0 mL), $H_2O$ (2.0 mL) and DPPH (205.2 mg, 0.88 mmol, 2.2 equiv). The reaction was stirred at 50 °C under argon for 10 min. The reaction was cooled to room temperature, diluted with 5 mL of NaCl aq., extracted three times with 5 mL of $Et_2O$. The organic layers were combined and dried over anhydrous $Na_2SO_4$, then concentrated under vacuum to give the crude product. The ring opening product was observed on crude $^1$H NMR and GC-MS (with 0.4 mmol of $CH_2Br_2$ as the internal standard).

## Figure S24: $^{1}H$ NMR of the reaction of 51a in $CDCl_3$

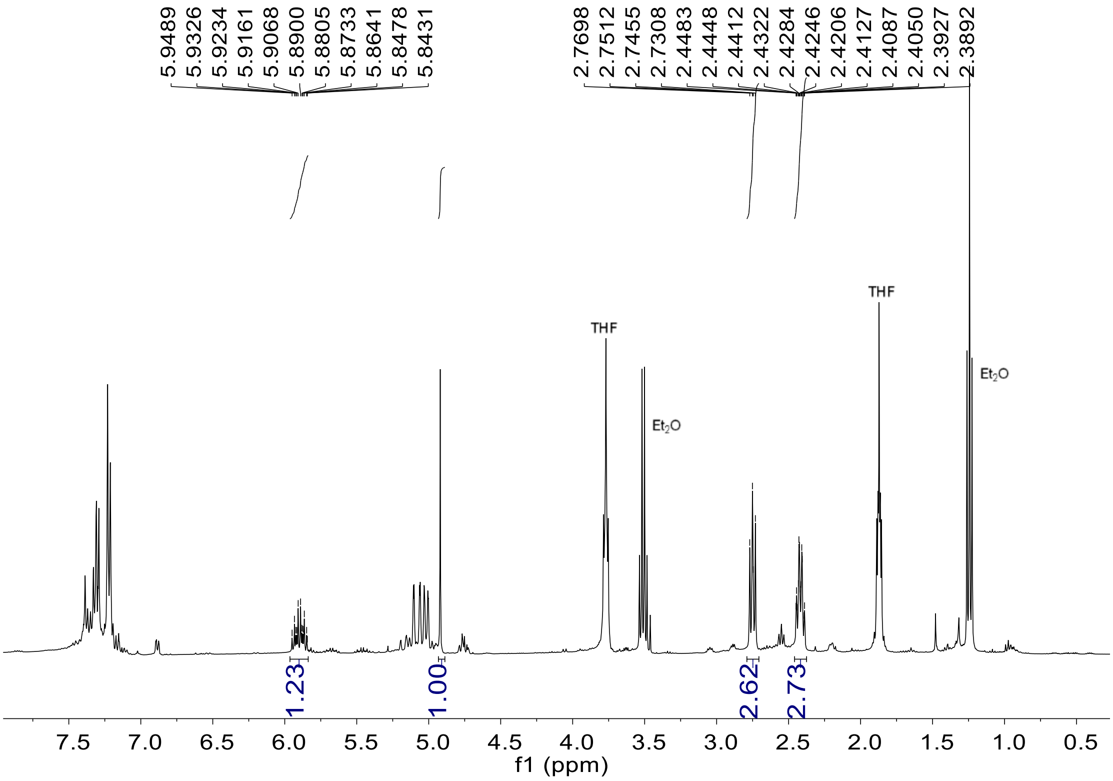


## Figure S25: GC-MS of the reaction of 51a

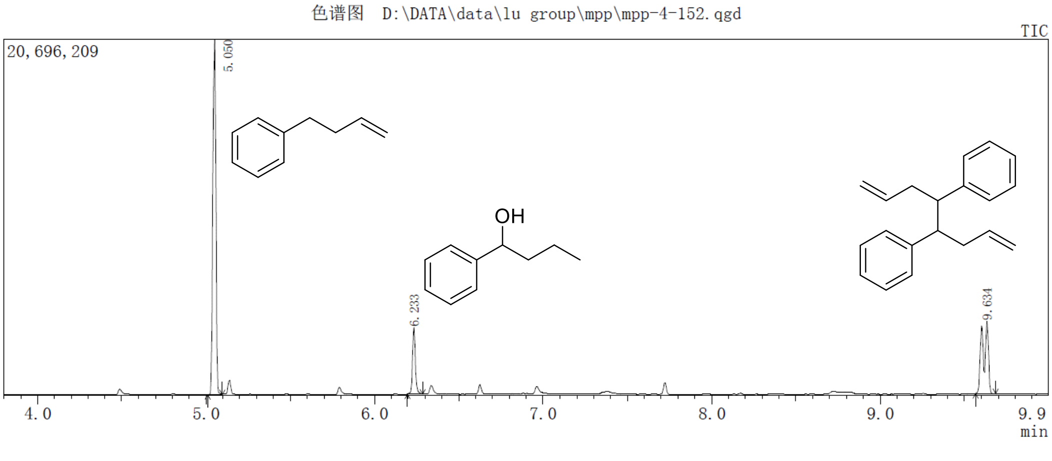


| peak | RT/min | peak area | peak area % | content |
|---|---|---|---|---|
| 1 | 5.050 | 25618962 | 63.26 | but-3-en-1-ylbenzene (**51b'**) |
| 2 | 6.233 | 4744982 | 11.72 | 1-phenylbutan-1-ol |
| 3 | 9.634 | 10134391 | 25.02 | octa-1,7-diene-4,5-diyldibenzene (**51d**) |

*RT=5.050 min*

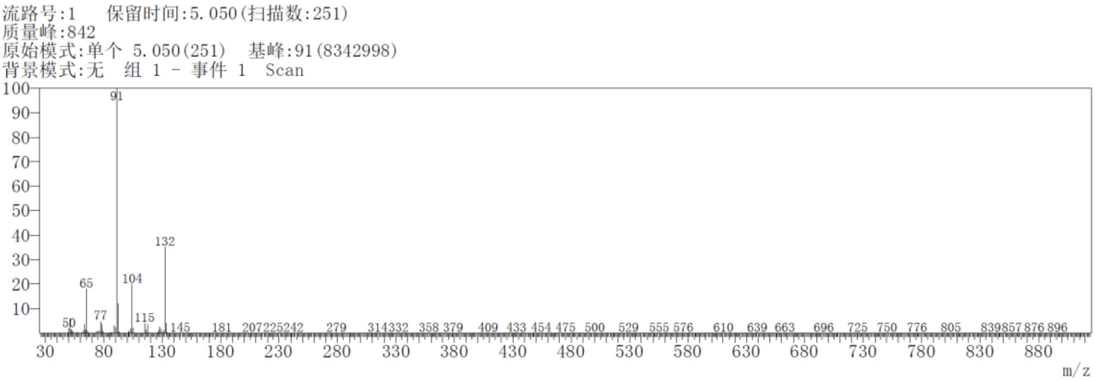


*RT=6.235 min*

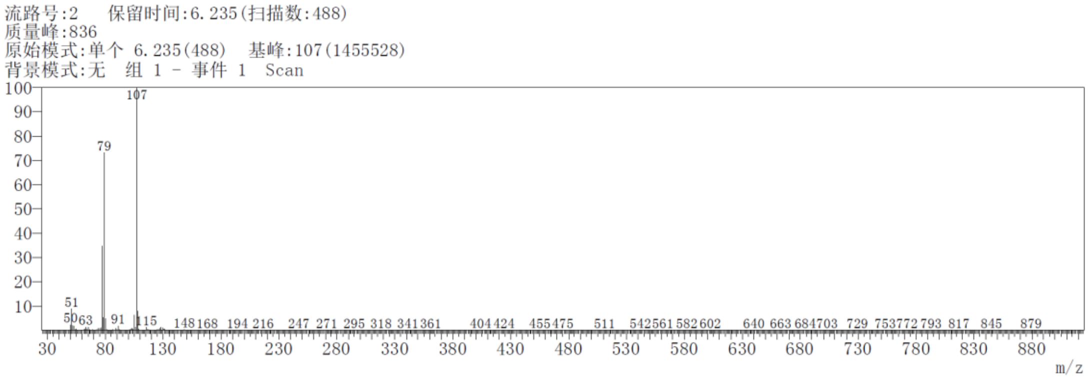


*RT=9.635 min*

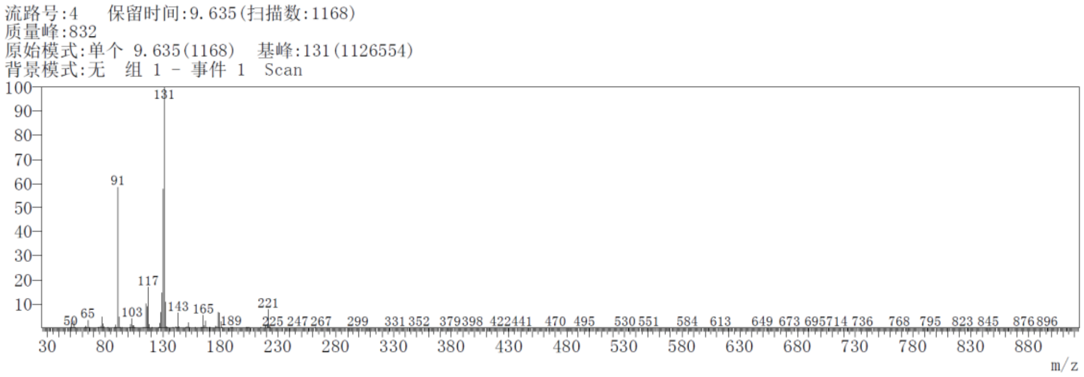


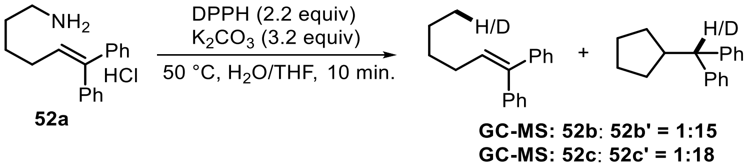


To a 10 mL vial with a stirring bar was added 6,6-diphenylhex-5-en-1-amine hydrochloride (28.8 mg, 0.1 mmol, 1.0 equiv), $K_2CO_3$ (30.4 mg, 0.22 mmol, 2.2 equiv),

THF (0.5 mL), $H_2O$ (0.5 mL) and DPPH (51.3 mg, 0.22 mmol, 2.2 equiv). The reaction was stirred at 50 °C for 5 min. The reaction was cooled to room temperature, diluted with 5 mL of NaCl aq., extracted three times with 5 mL of PE. The organic layers were combined, dried over anhydrous $Na_2SO_4$, then concentrated under vacuum to give the crude product. The cyclization product was observed on crude $^1H$ NMR and GC-MS (with 0.1 mmol of $CH_2Br_2$ as the internal standard).

**Figure S26: $^1$H NMR of the hydrodeamination reaction of 52a**

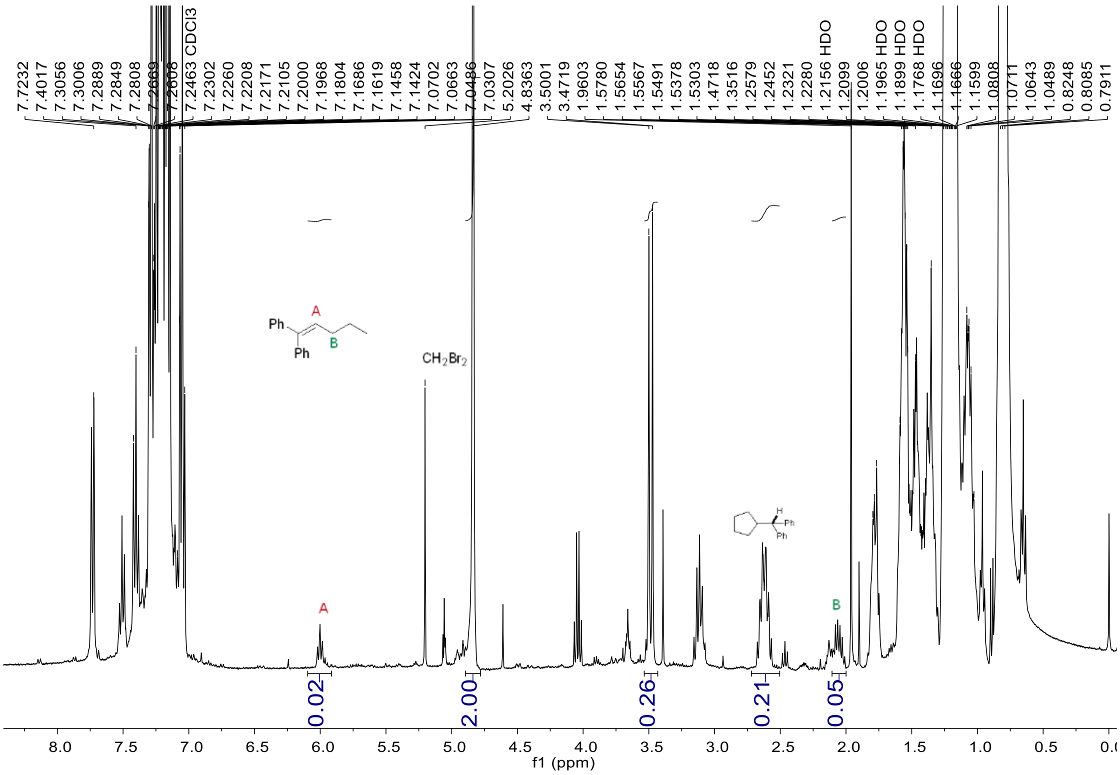

## Figure S27: GC-MS of the deuterodeamination reaction of 52a

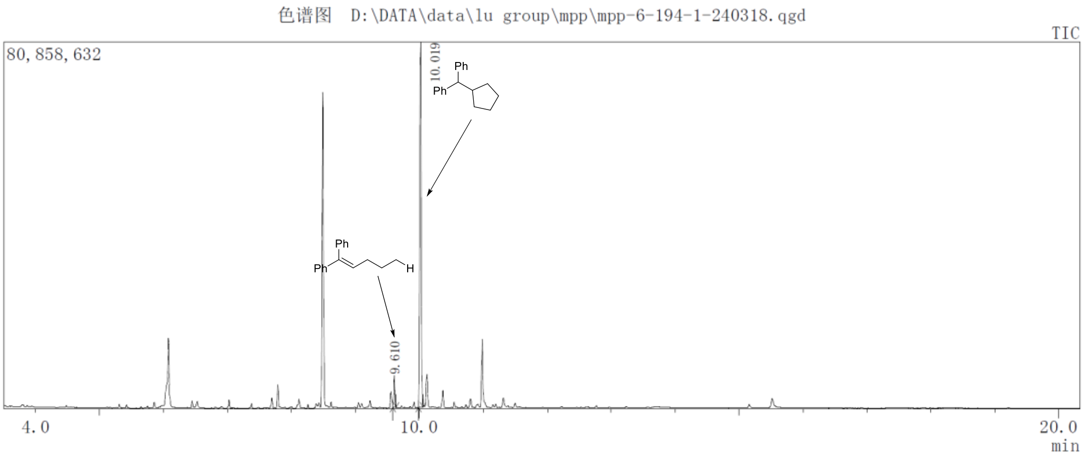


| peak | RT/min | peak area | peak area % | content |
|---|---|---|---|---|
| 1 | 9.610 | 8477834 | 6.24 | hex-1-ene-1,1-diyldibenzene (**52b**) |
| 2 | 10.019 | 127402547 | 93.76 | (cyclopentylmethylene)dibenzene (**52b'**) |

*RT=9.610 min*

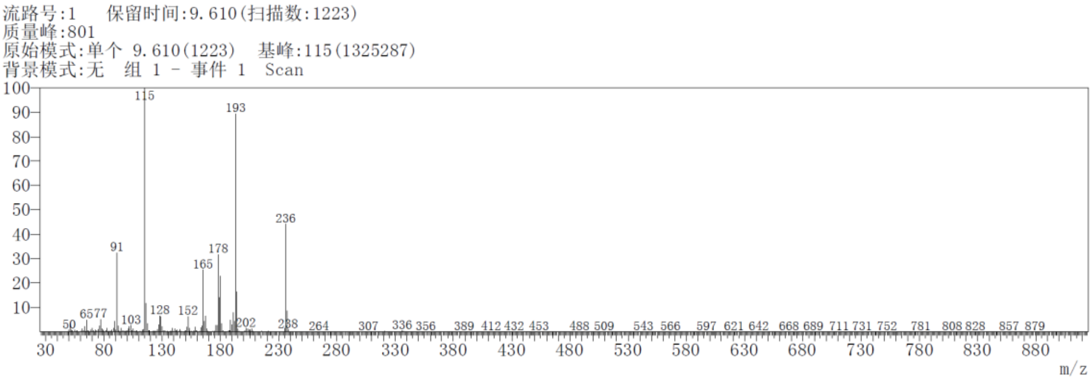

*RT=10.025 min*

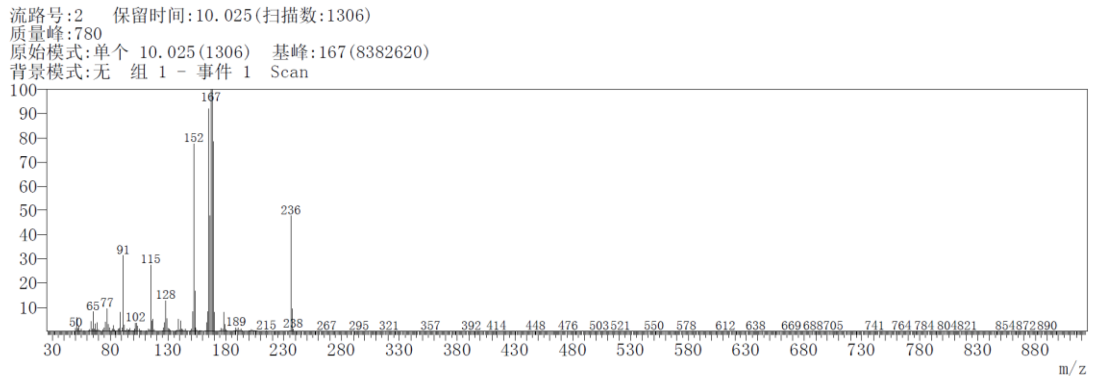


**Figure S28: $^{1}$H NMR of the deuterodeamination reaction of 52a**

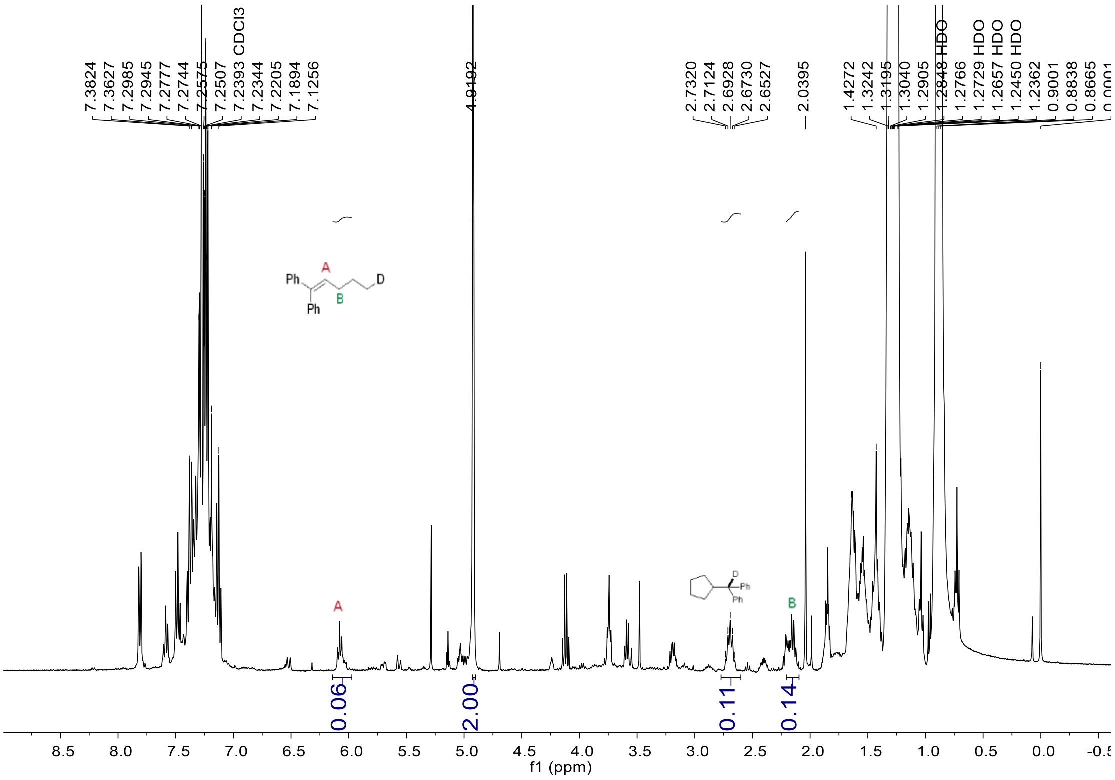

## Figure S29: GC-MS of the deuterodeamination reaction of 52a

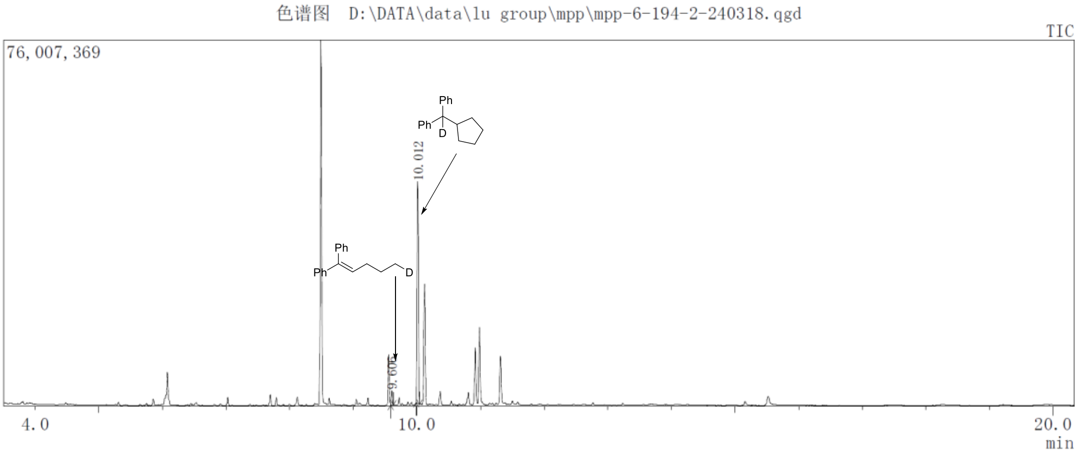


| peak | RT/min | peak area | peak area % | content |
|---|---|---|---|---|
| 1 | 9.630 | 3725033 | 5.15 | hex-1-ene-1,1-diyldibenzene (**52b**) |
| 2 | 10.012 | 68546555 | 94.85 | (cyclopentylmethylene-*d*)dibenzene (**52b'**) |

*RT=9.605 min*

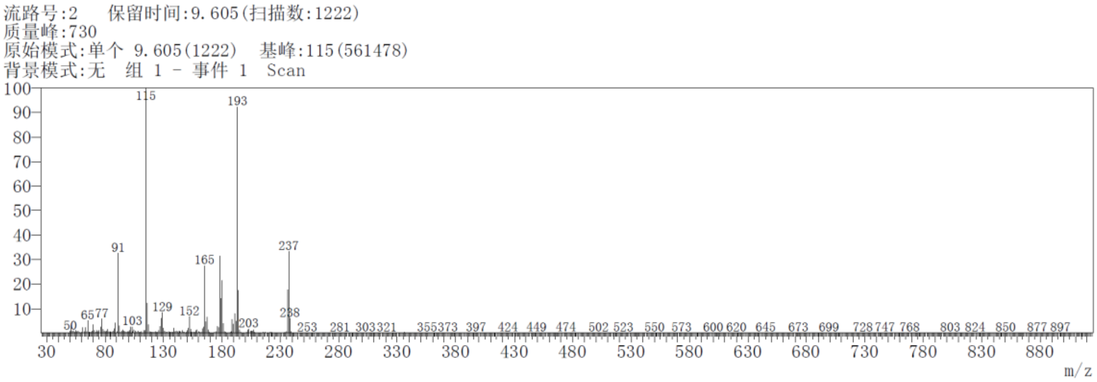


*RT=10.010 min*

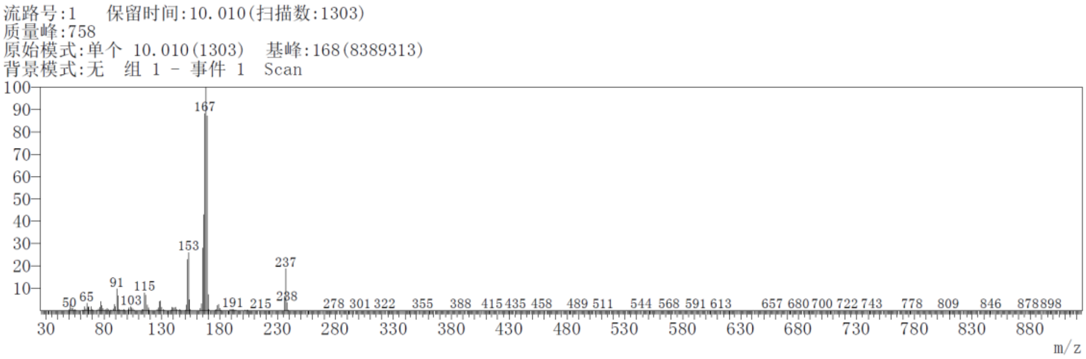

**Figure S30: GC-MS of the isolated 52b'**

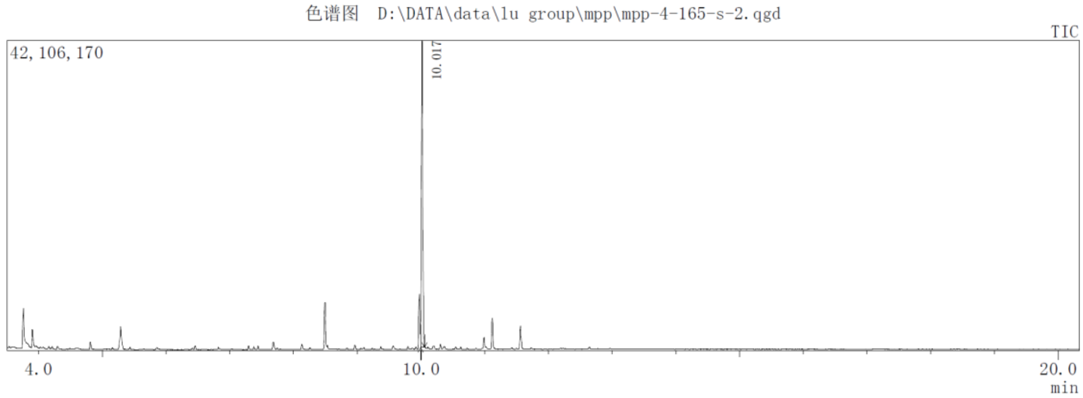


RT=10.015 min

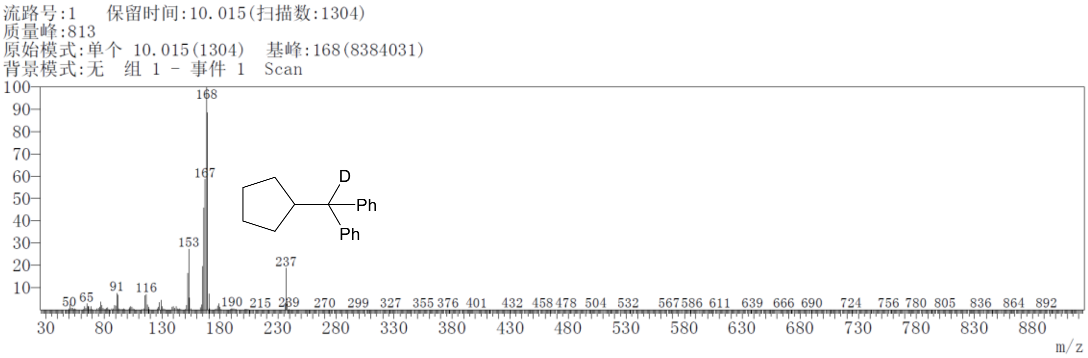

## 8. Copies of NMR Spectra

**1a:** $^{1}H$ NMR (400 MHz, DMSO-$d_6$)

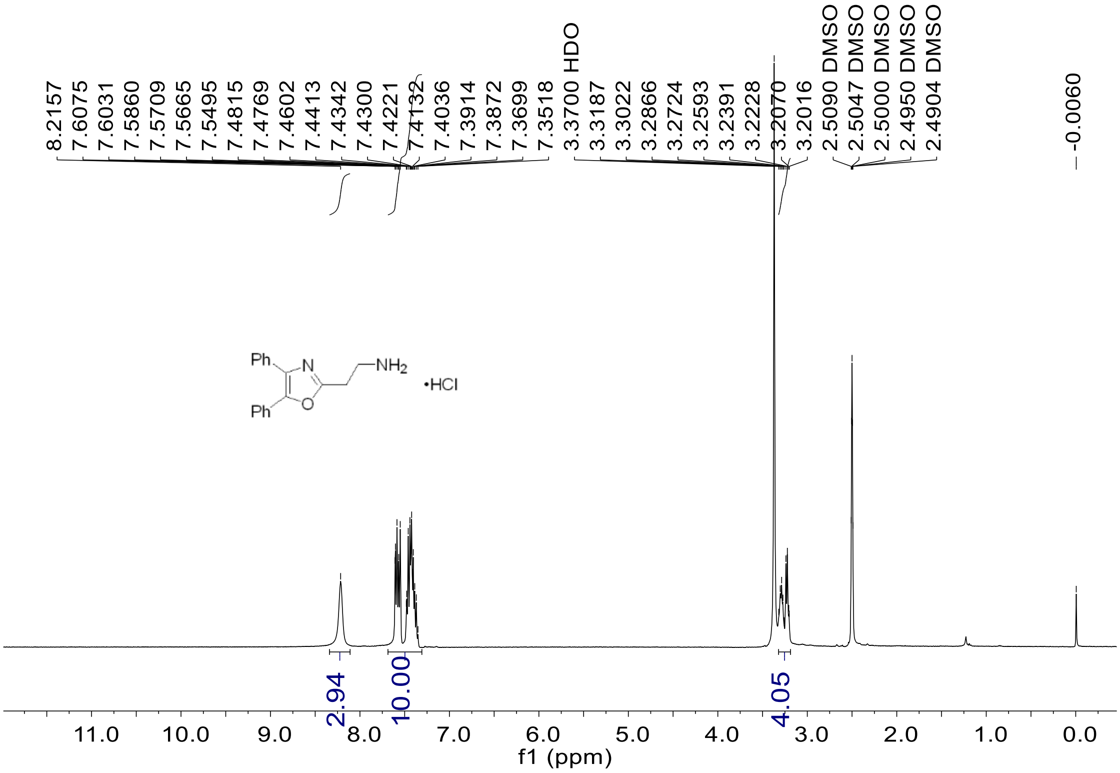


**1a:** $^{13}C$ NMR (101 MHz, DMSO-$d_6$)

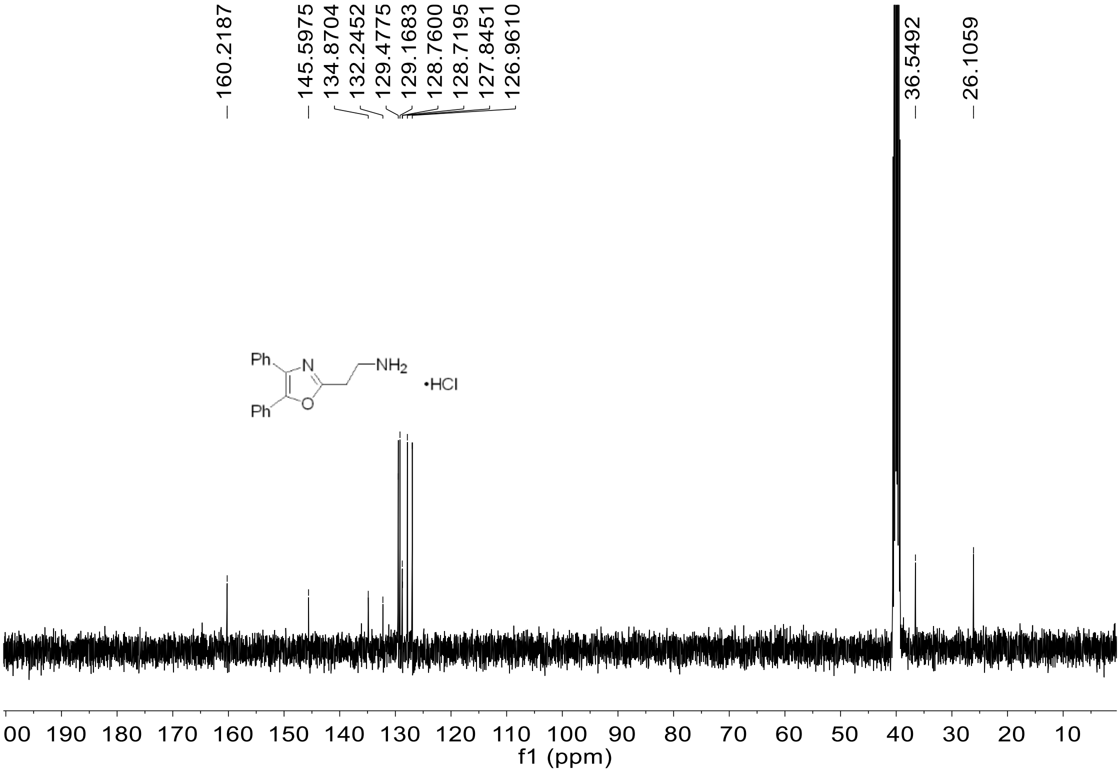

**8a:**$^{1}$H NMR (400 MHz, DMSO-$d_6$)

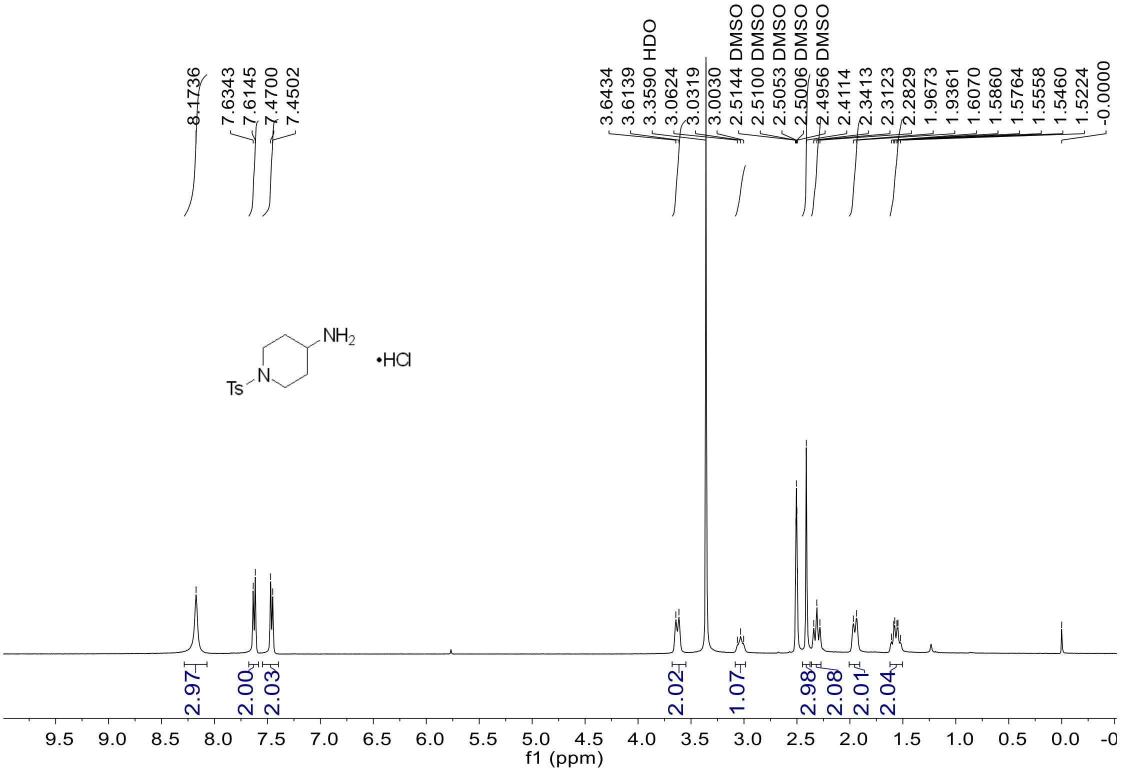


**8a:**$^{13}$C NMR (101 MHz, DMSO-$d_6$)

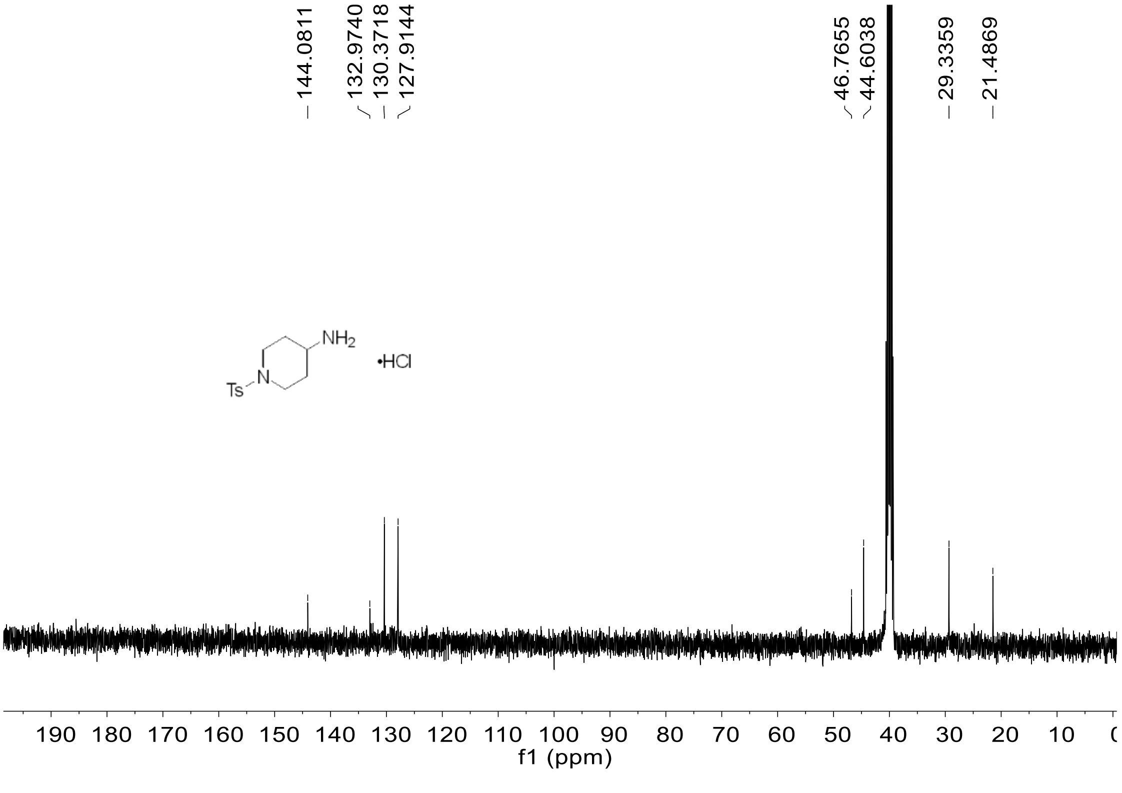

**10a:** $^{1}$H NMR (400 MHz, DMSO-$d_6$)

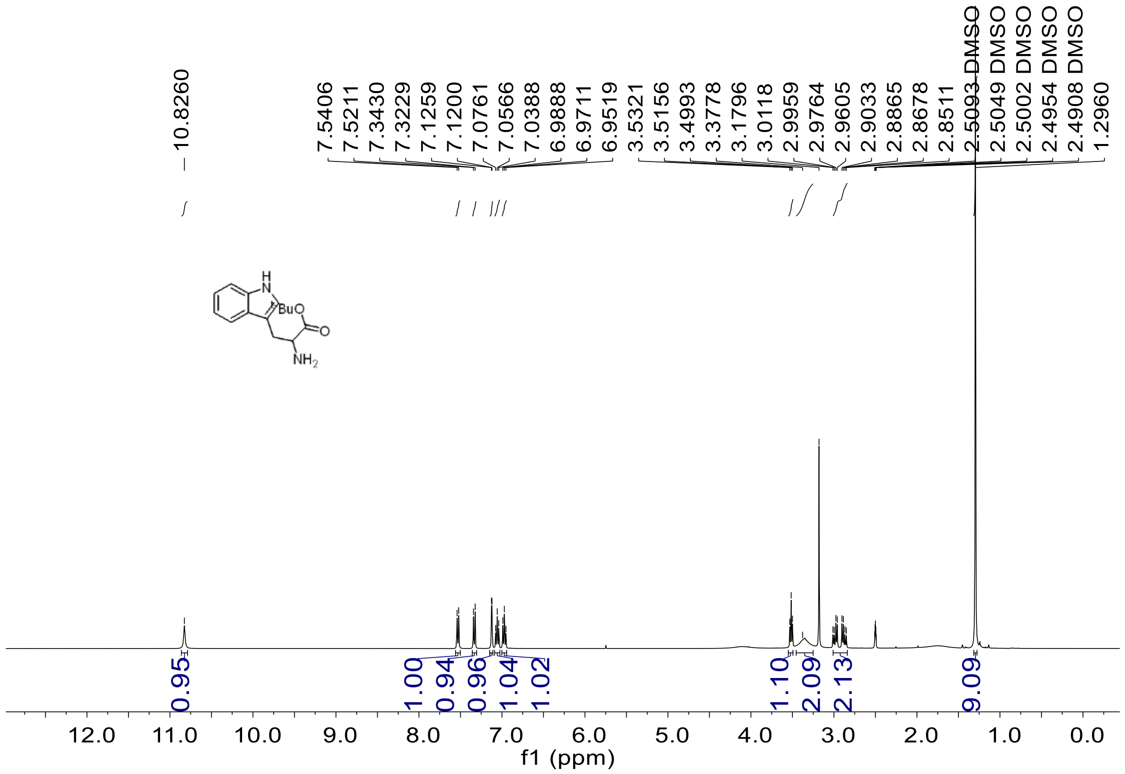


**10a:** $^{13}$C NMR (101 MHz, DMSO-$d_6$)

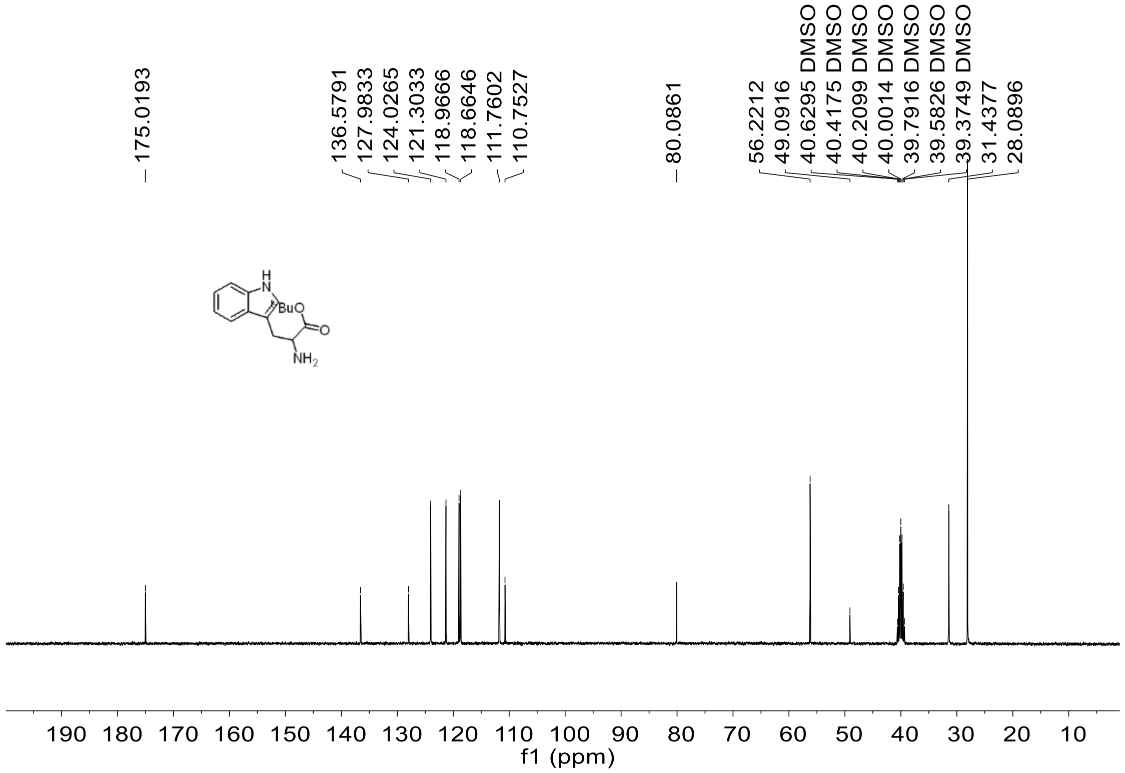

**20a:** $^{1}$H NMR (400 MHz, Chloroform-*d*)

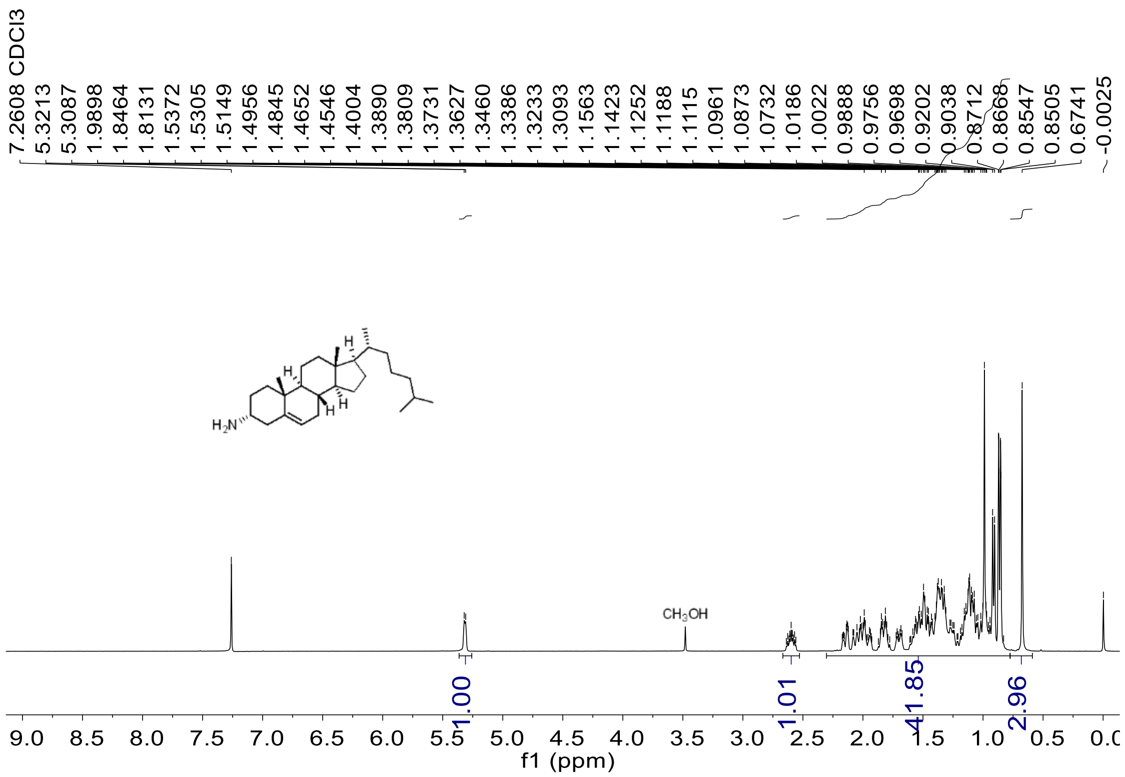


**20a:** $^{13}$C NMR (101 MHz, Chloroform-*d*)

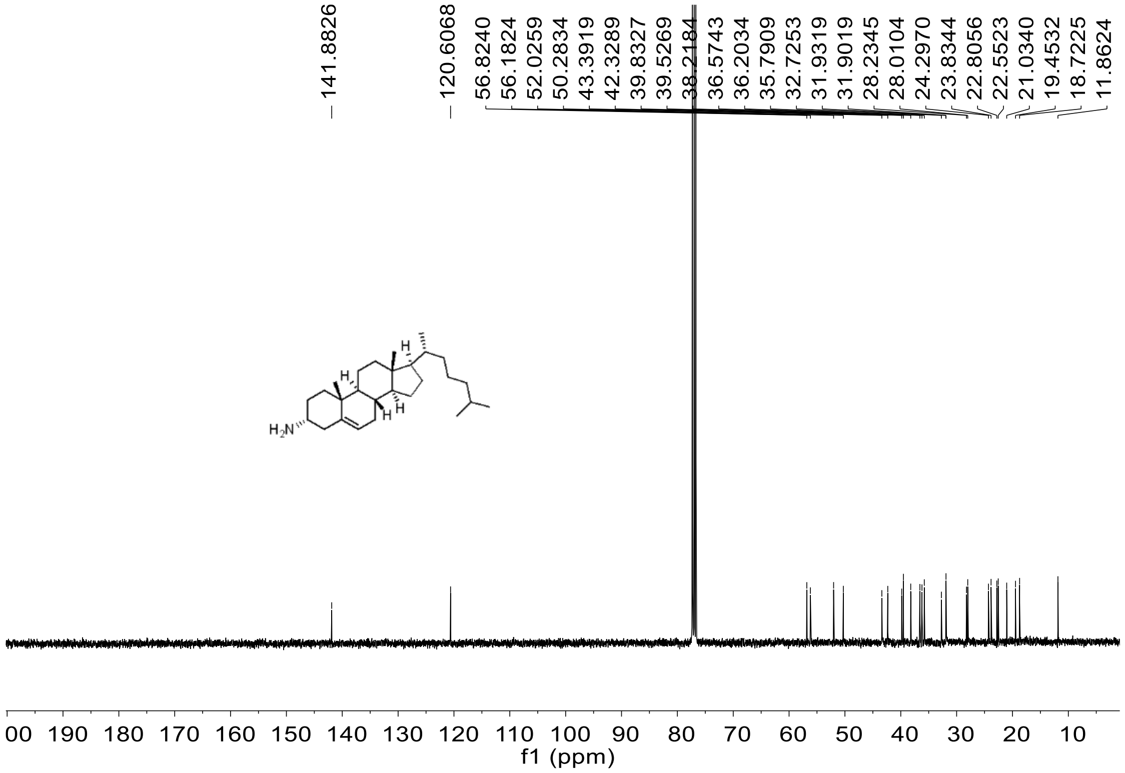

**21a:** $^{1}$H NMR (400 MHz, DMSO-$d_6$)

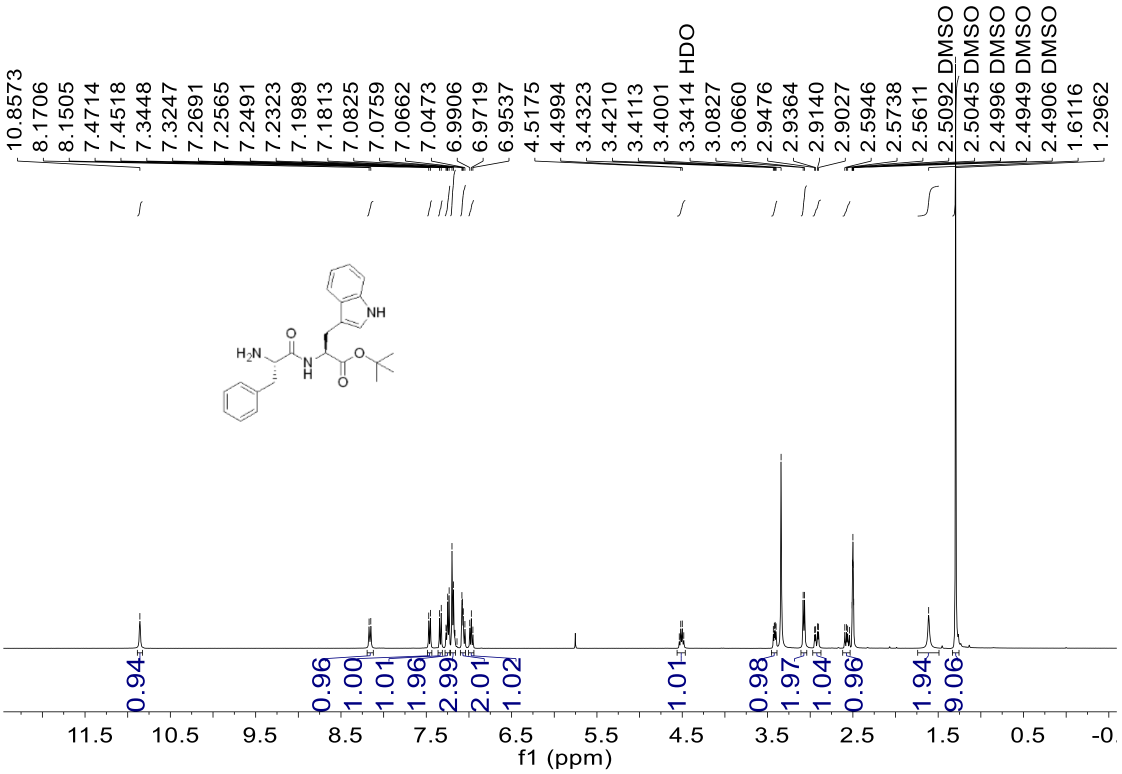


**21a:** $^{13}$C NMR (101 MHz, DMSO-$d_6$)

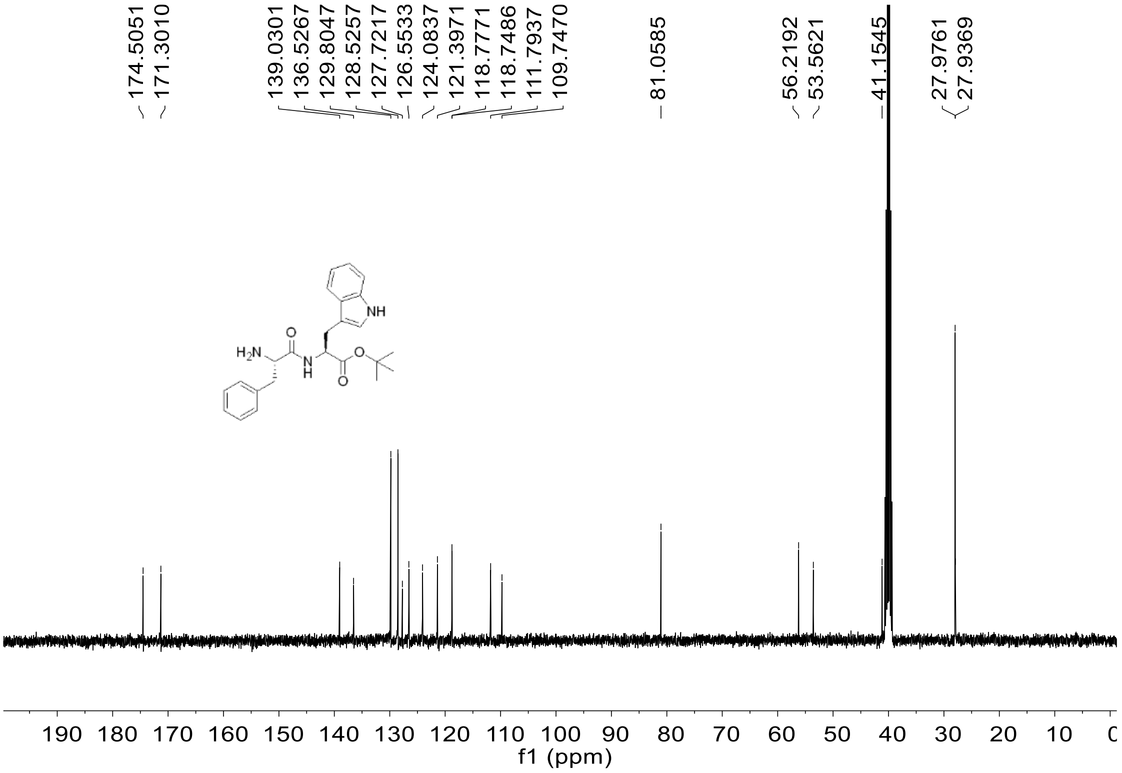

**22a:** $^{1}$H NMR (400 MHz, DMSO-$d_6$)

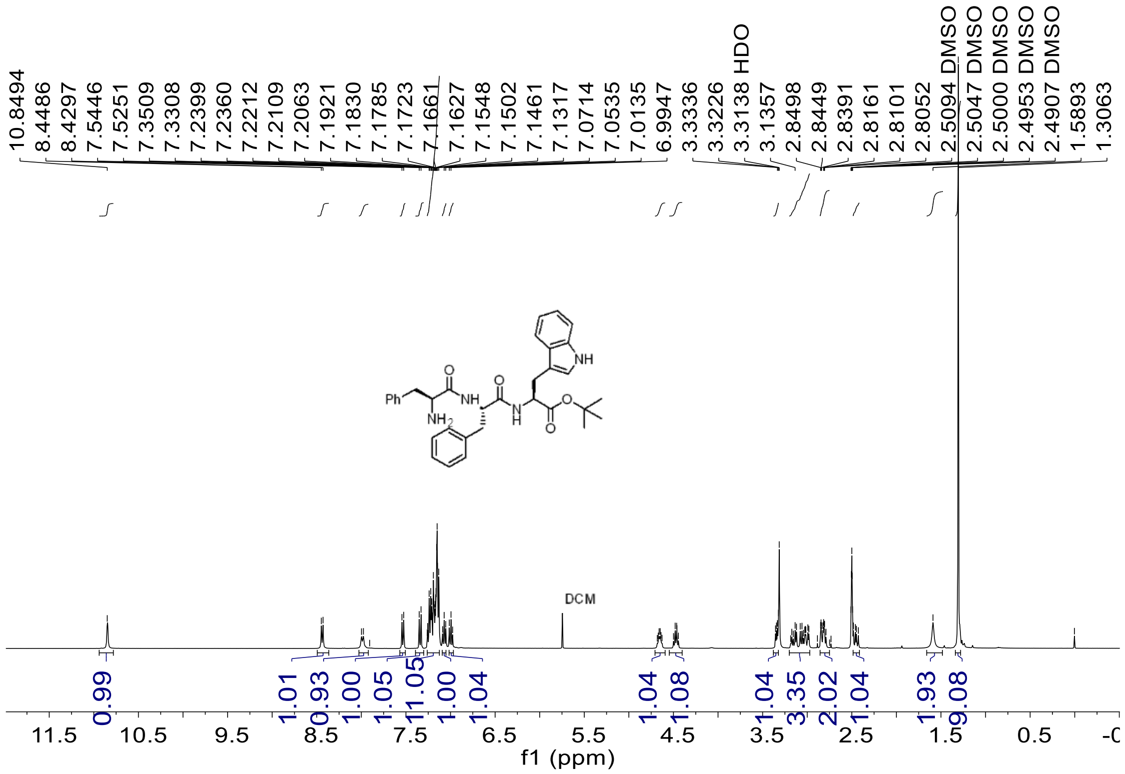


**22a:** $^{13}$C NMR (101 MHz, DMSO-$d_6$)

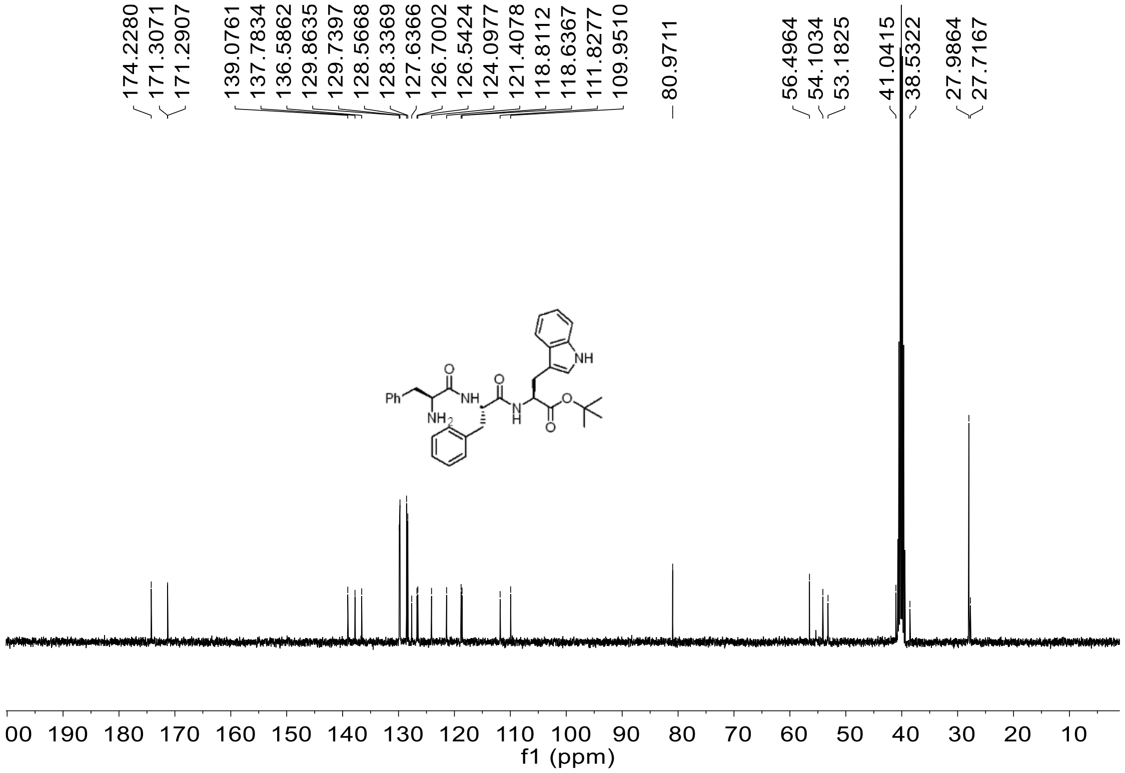

**23a:** $^{1}$H NMR (400 MHz, DMSO-$d_6$)

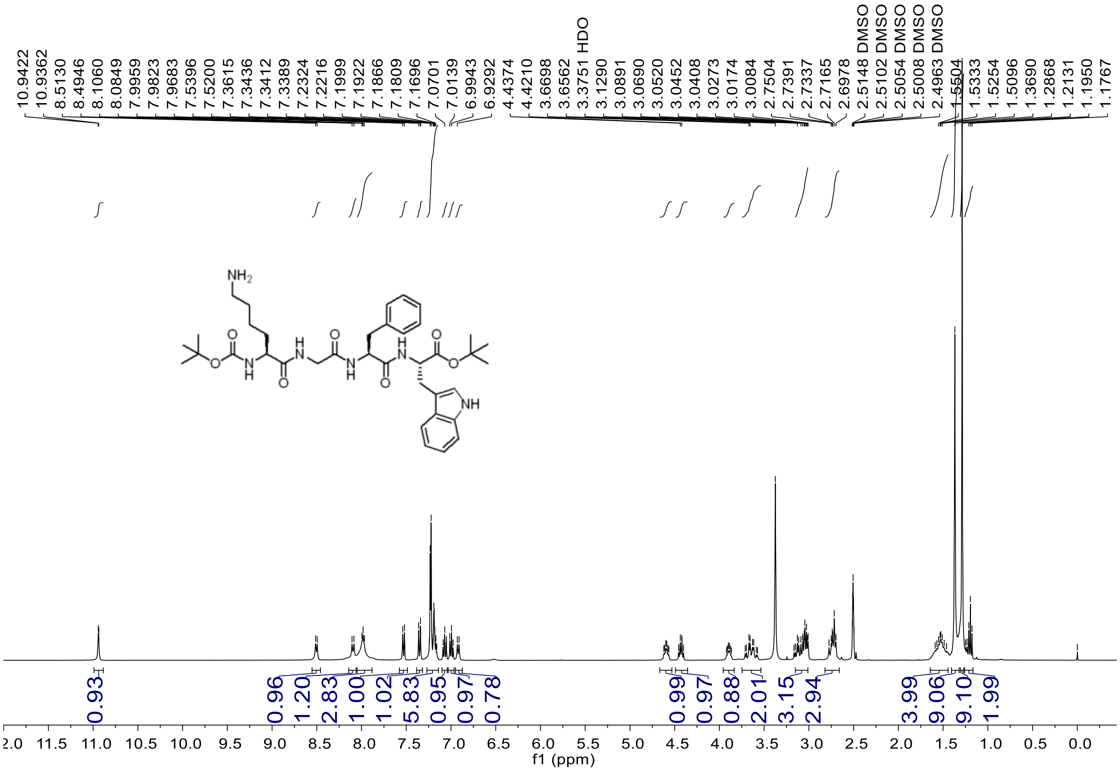

**23a:** $^{13}$C NMR (101 MHz, DMSO-$d_6$)

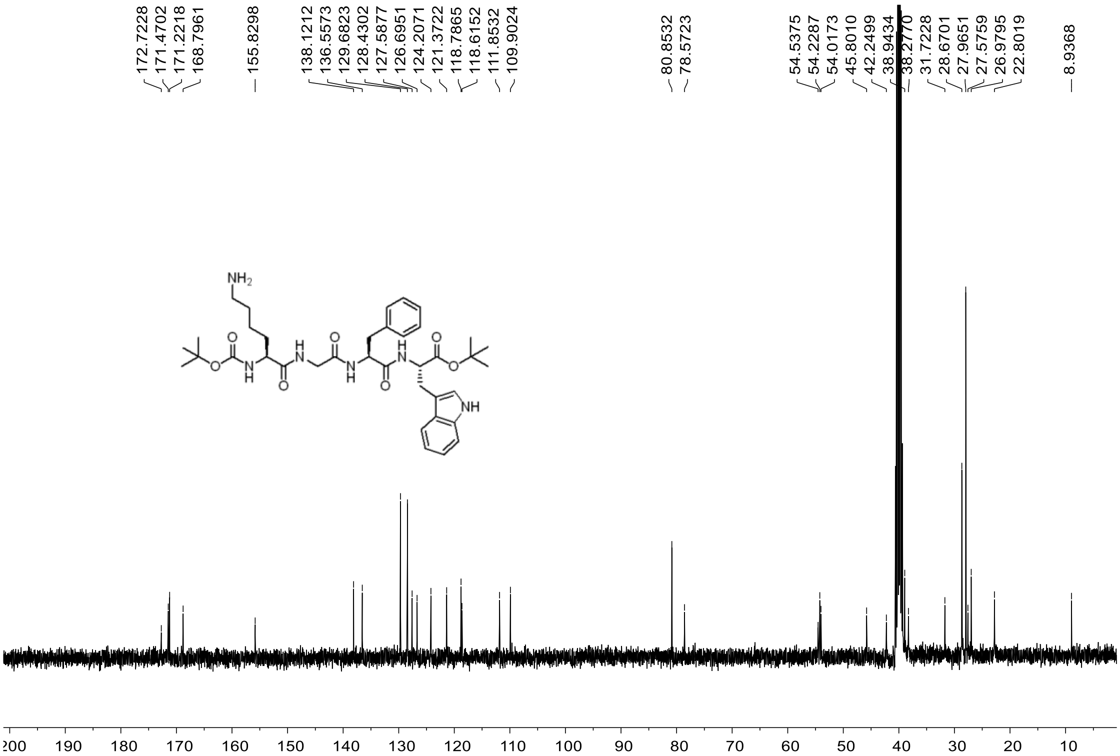

**24a:** $^{1}H$ NMR (400 MHz, DMSO-$d_6$)

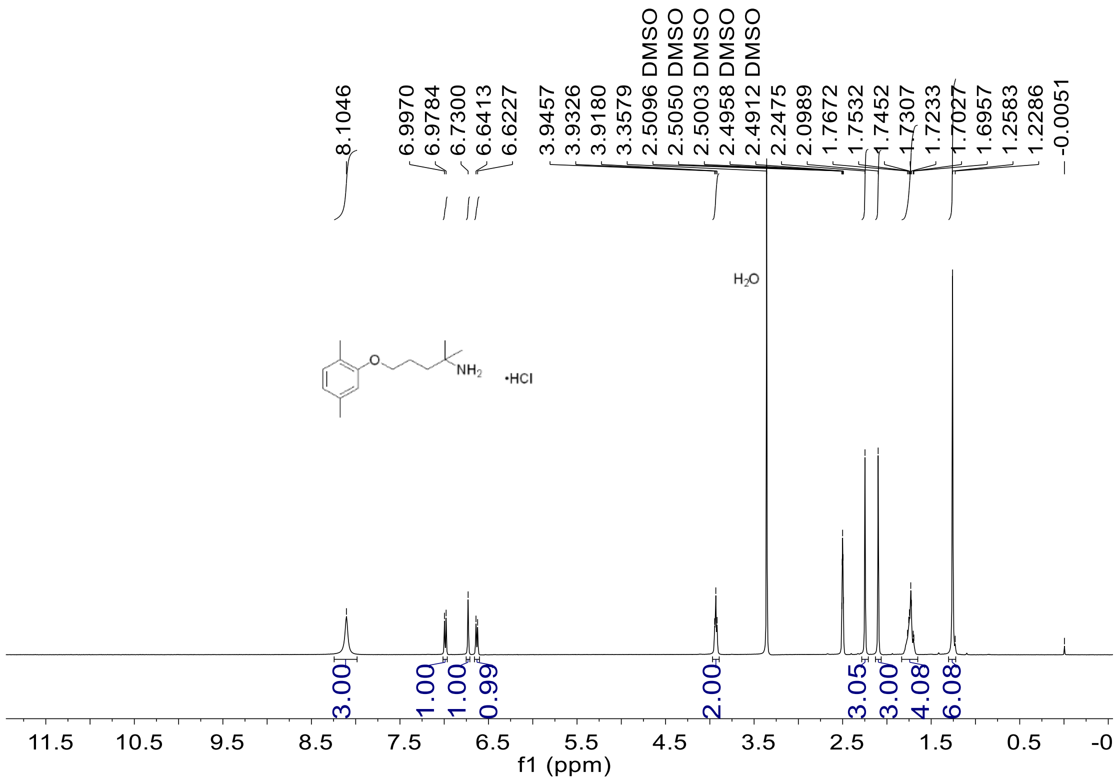


**24a:** $^{13}C$ NMR (101 MHz, DMSO-$d_6$)

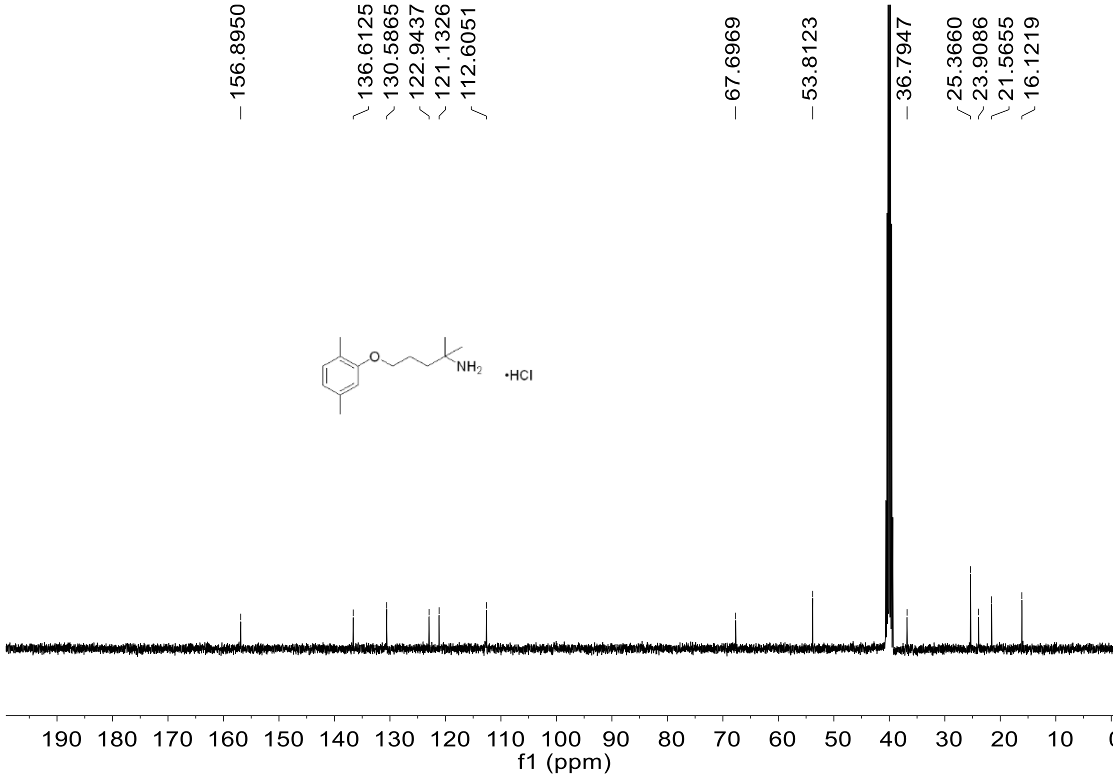

**30a:** $^{1}$H NMR (400 MHz, Chloroform-*d*)

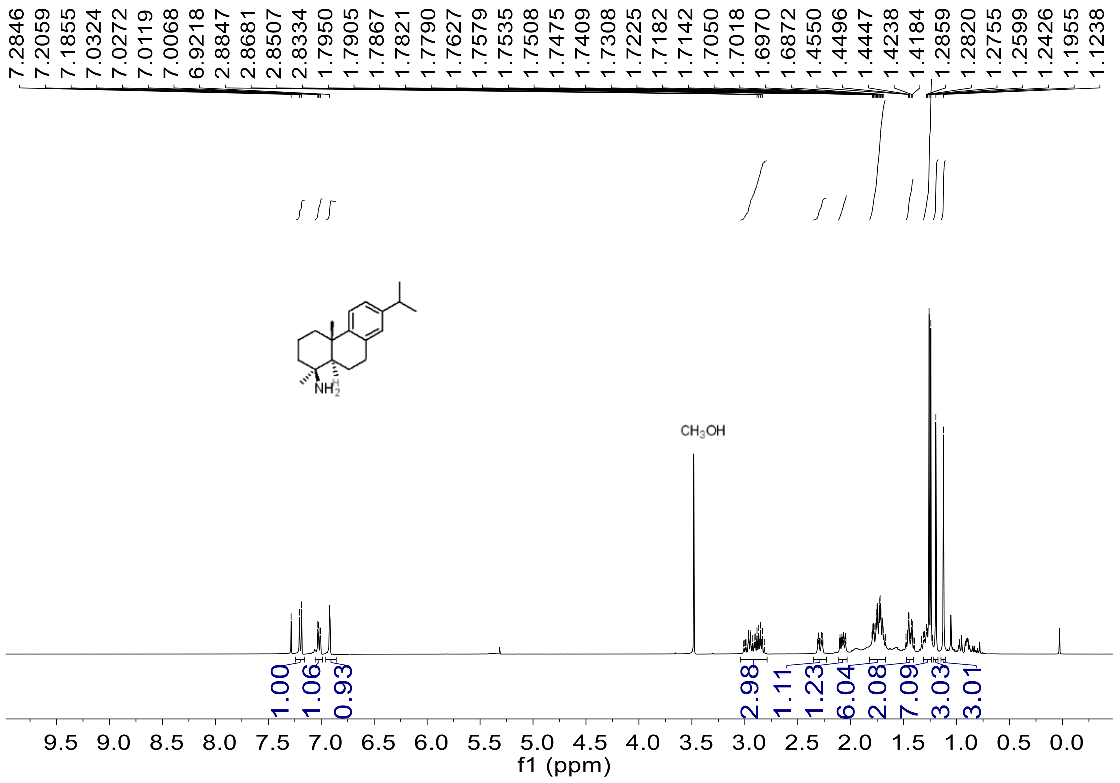

**30a:** $^{13}$C NMR (101 MHz, Chloroform-*d*)

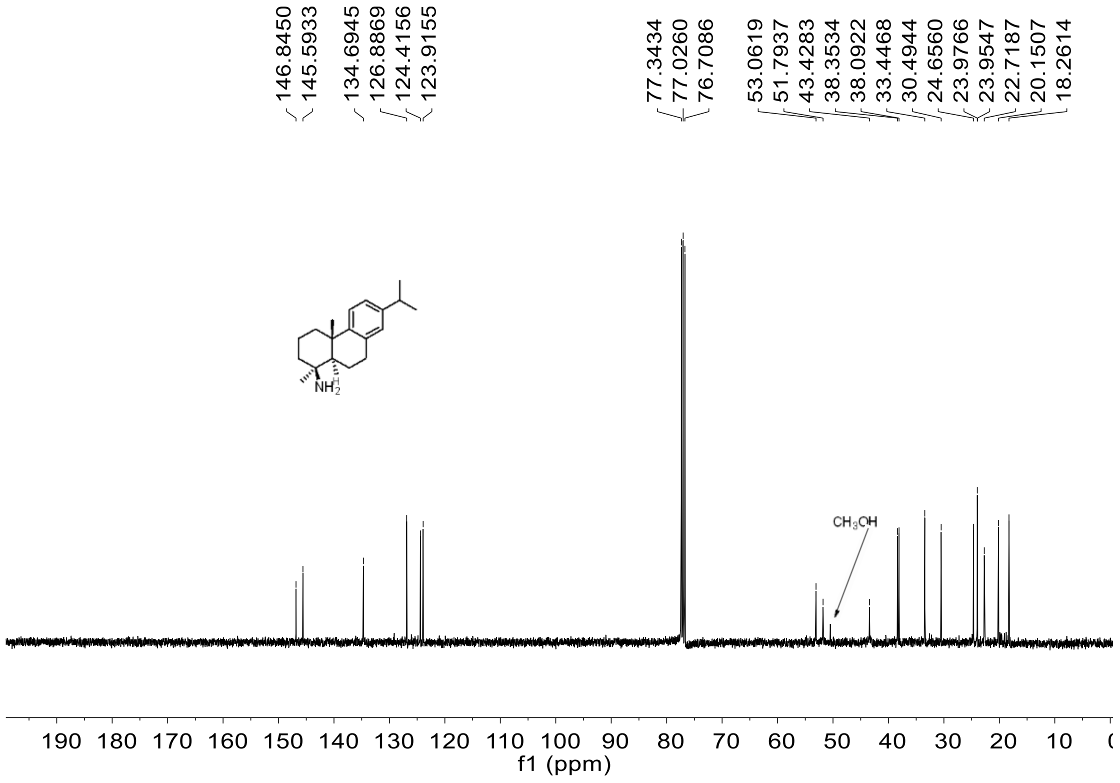

**51a:** $^{1}$H NMR (400 MHz, DMSO-$d_6$)

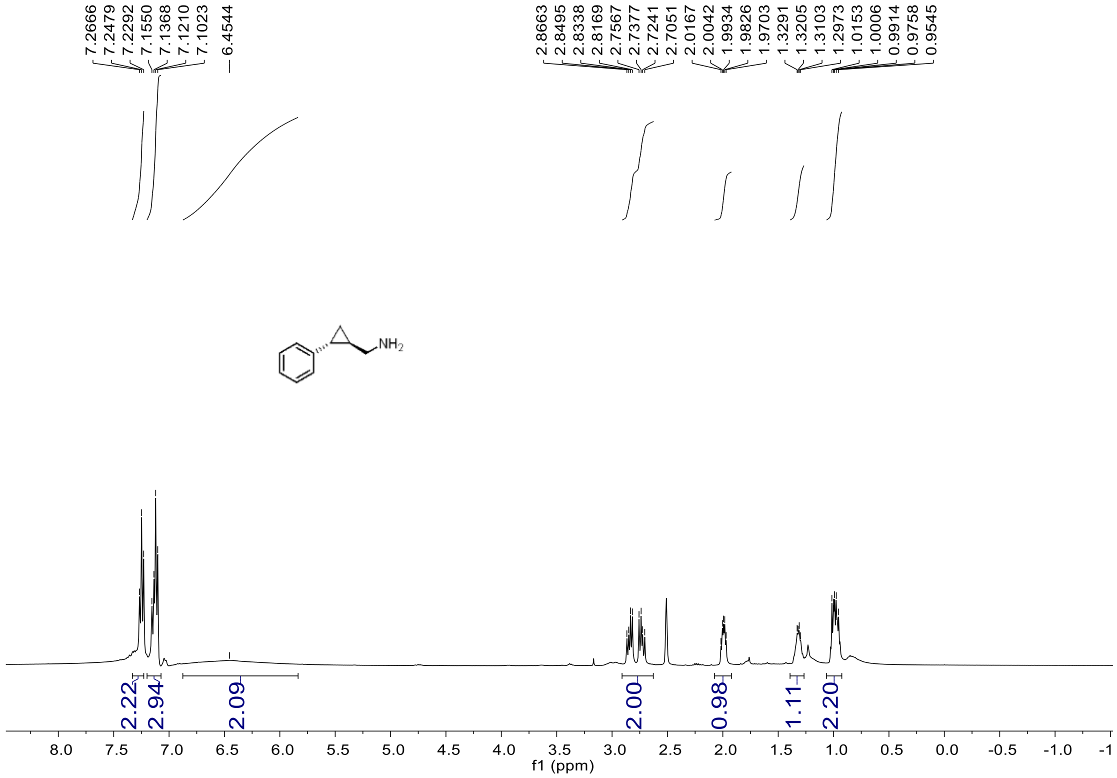


**51a:** $^{13}$C NMR (101 MHz, DMSO-$d_6$)

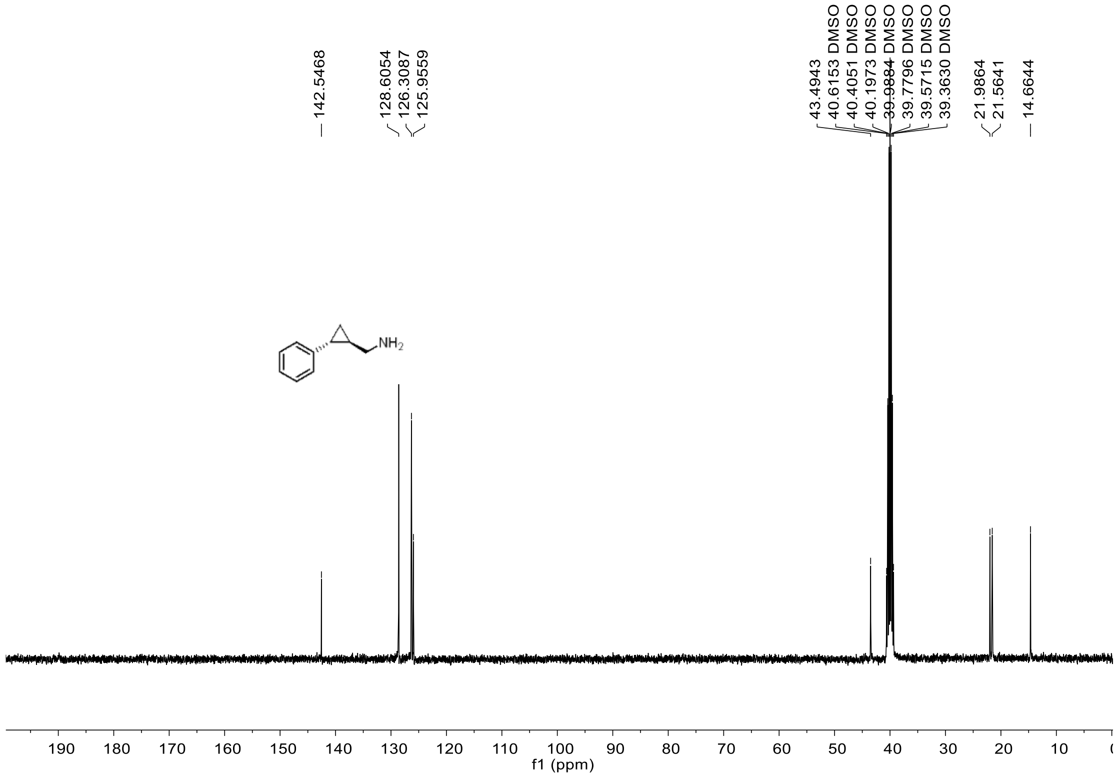

**52a:** $^{1}$H NMR (400 MHz, DMSO-$d_6$)

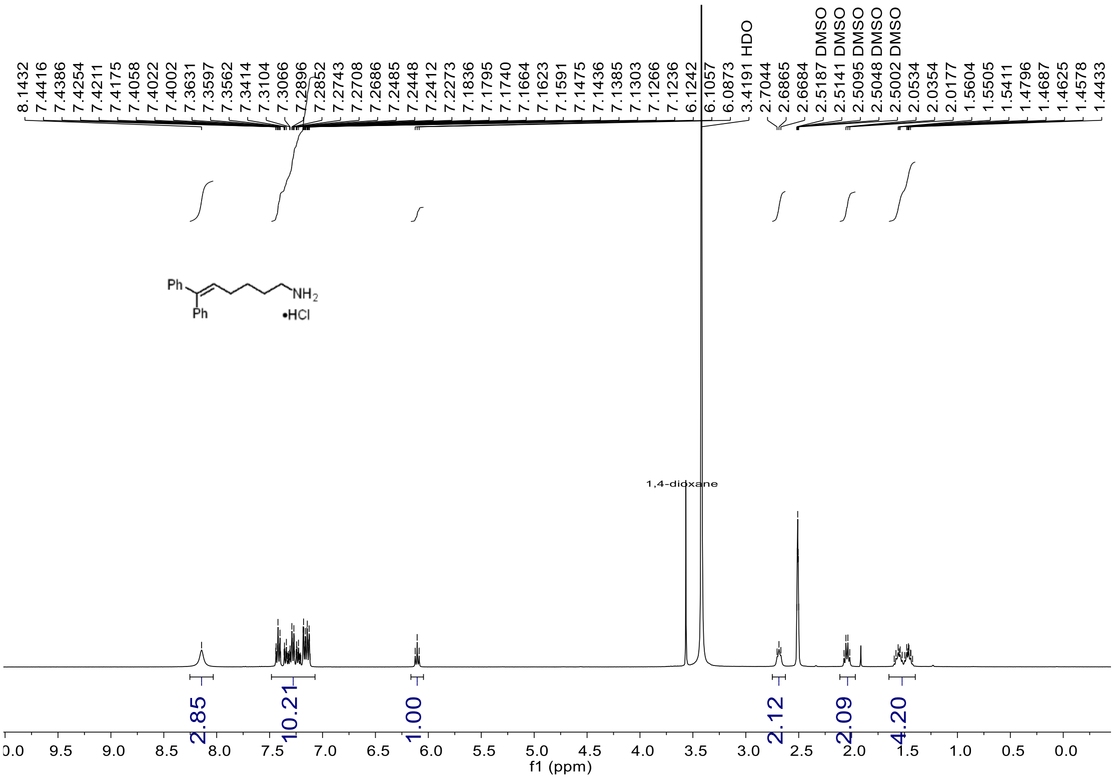

**52a:** $^{13}$C NMR (101 MHz, DMSO-$d_6$)

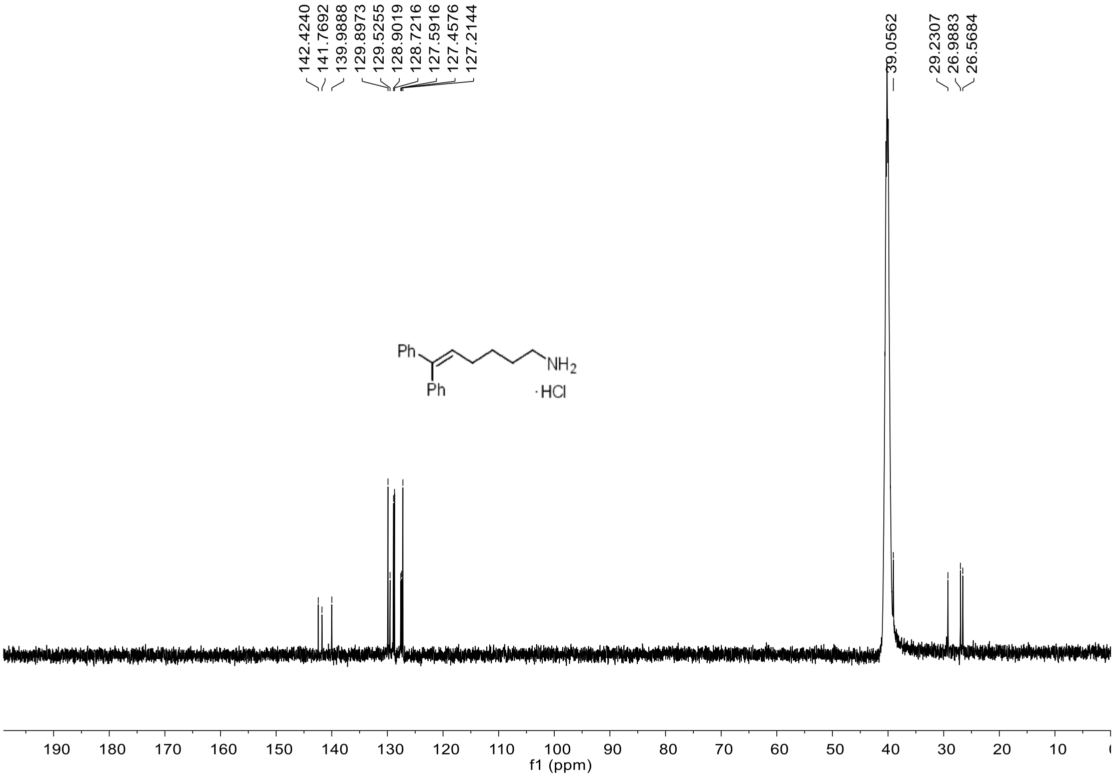

**1b:** $^{1}$H NMR (400 MHz, Chloroform-*d*)

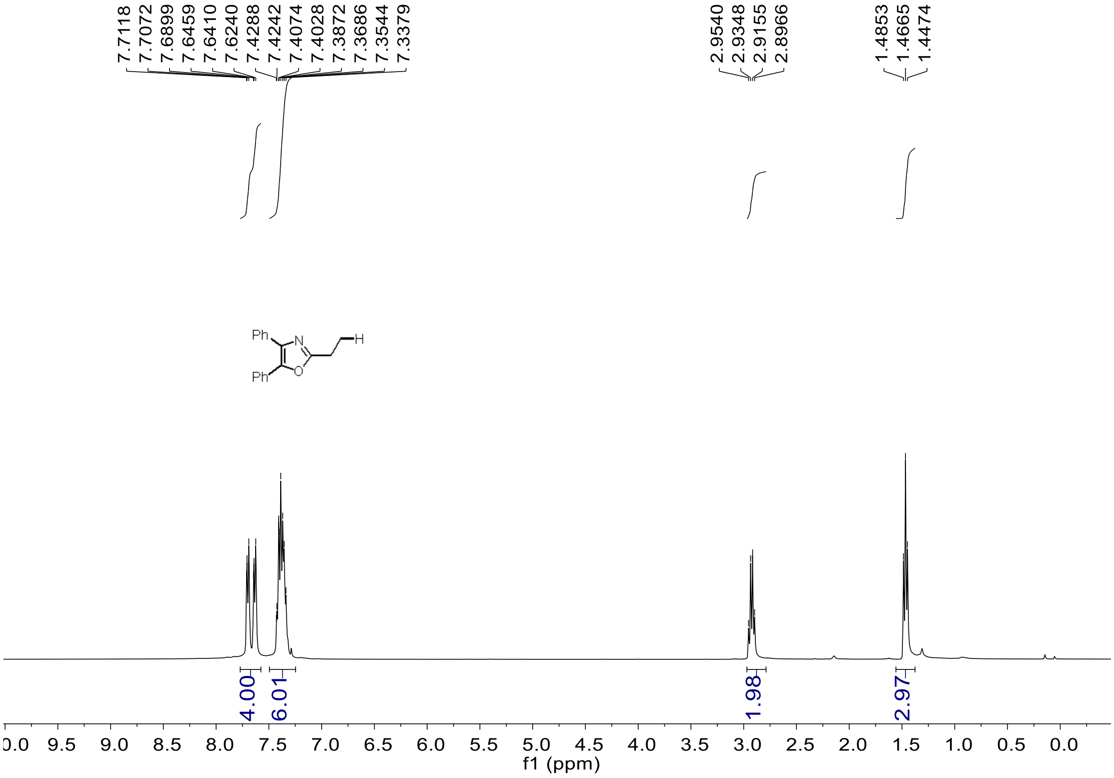


**1b:** $^{13}$C NMR (101 MHz, Chloroform-*d*)

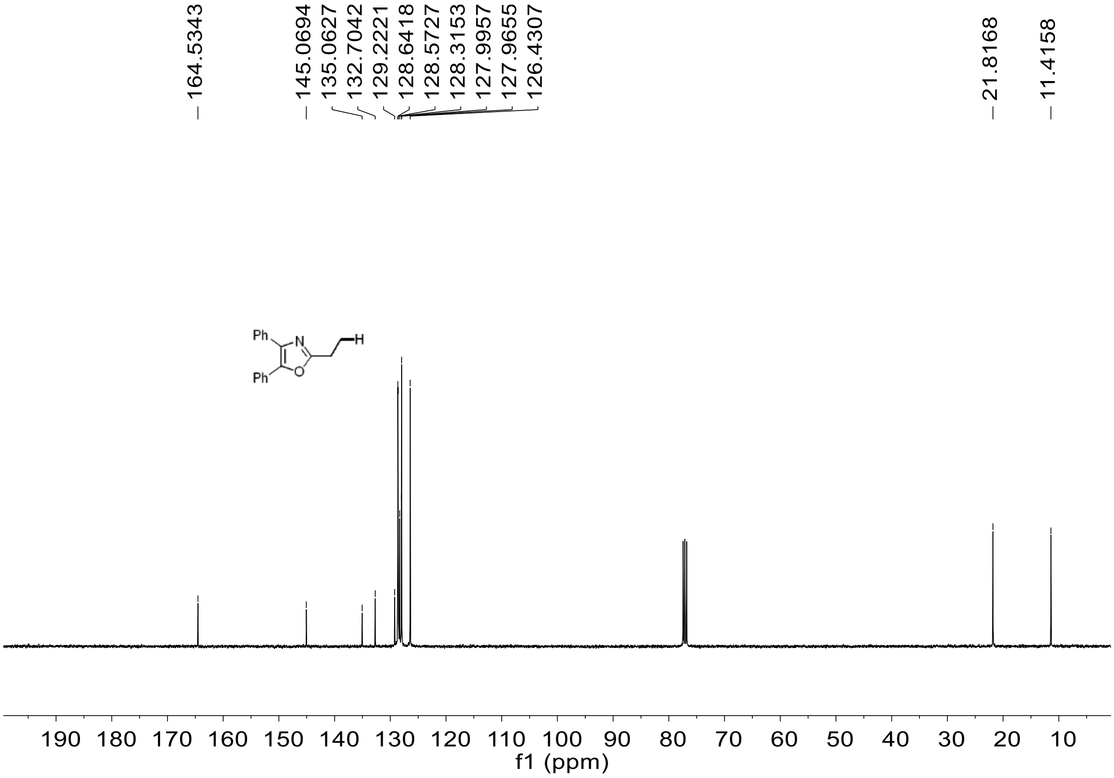

**1c:** $^{1}$H NMR (400 MHz, Chloroform-*d*)

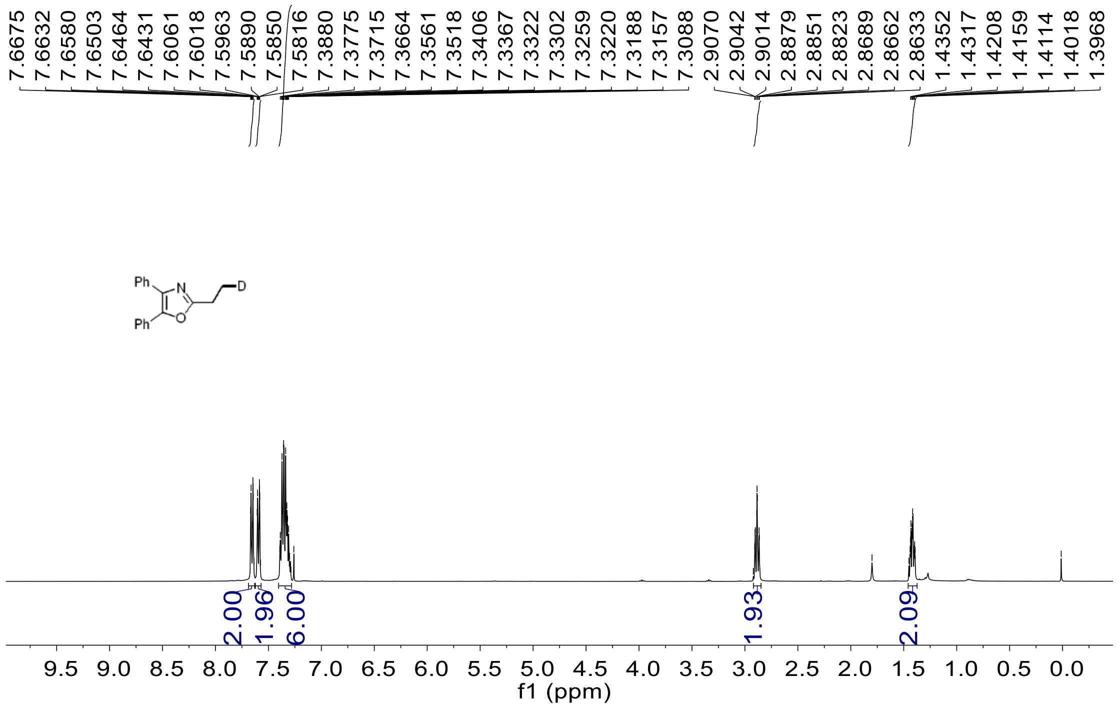


**1c:** $^{13}$C NMR (101 MHz, Chloroform-*d*)

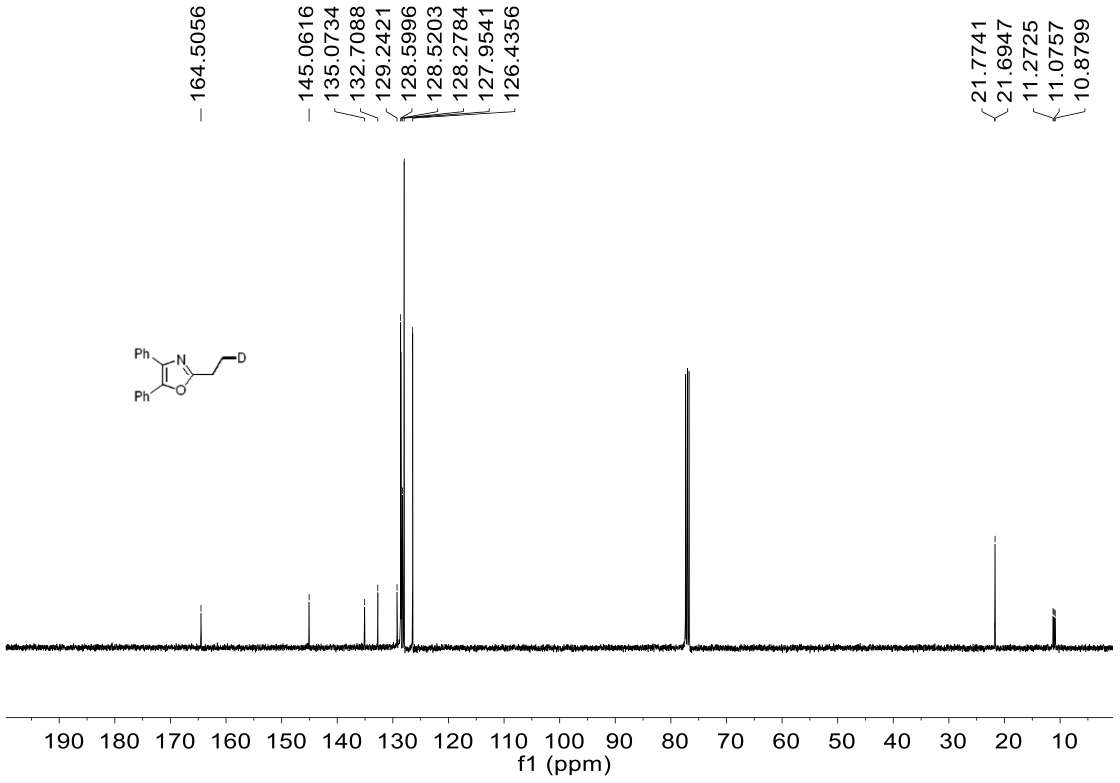

**2b:** $^{1}$H NMR (400 MHz, Chloroform-*d*)

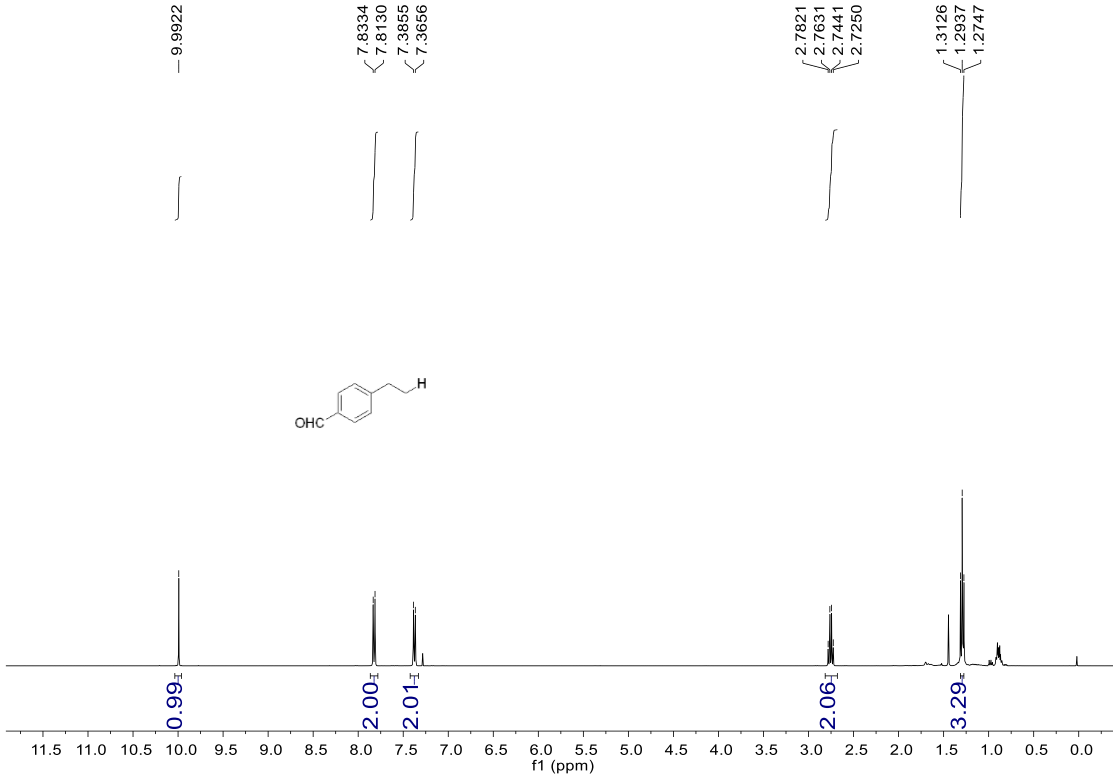


**2b:** $^{13}$C NMR (101 MHz, Chloroform-*d*)

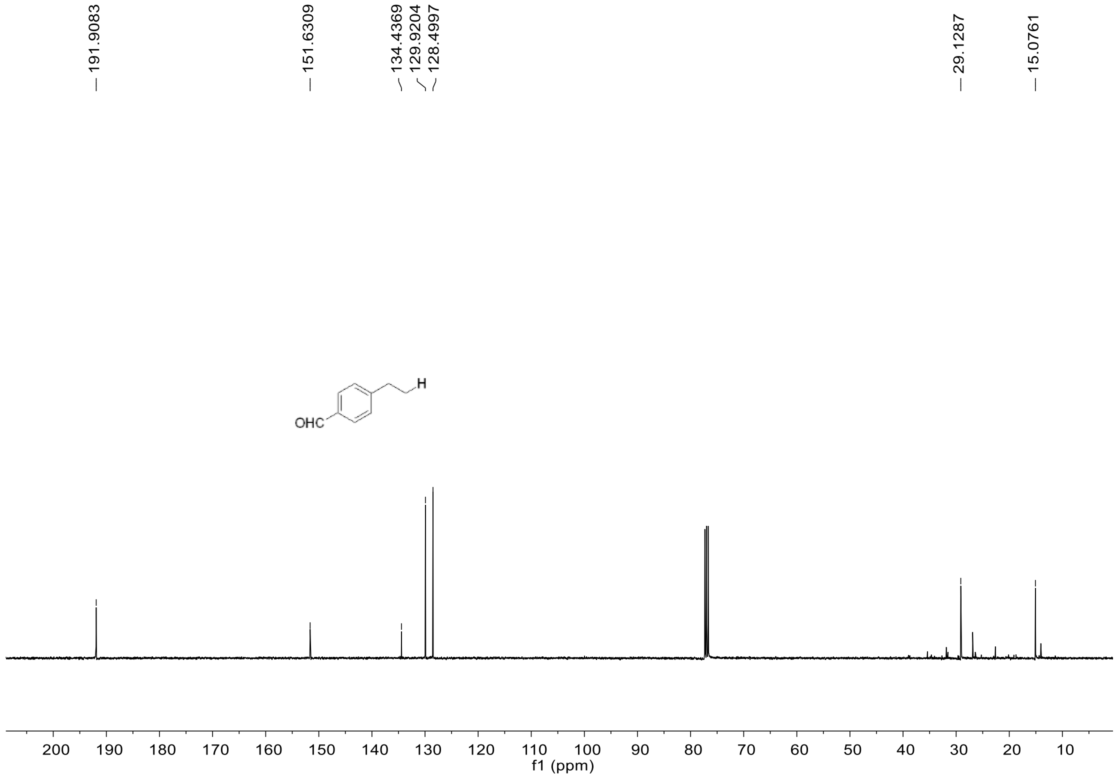

**2c:** $^{1}$H NMR (400 MHz, Chloroform-*d*)

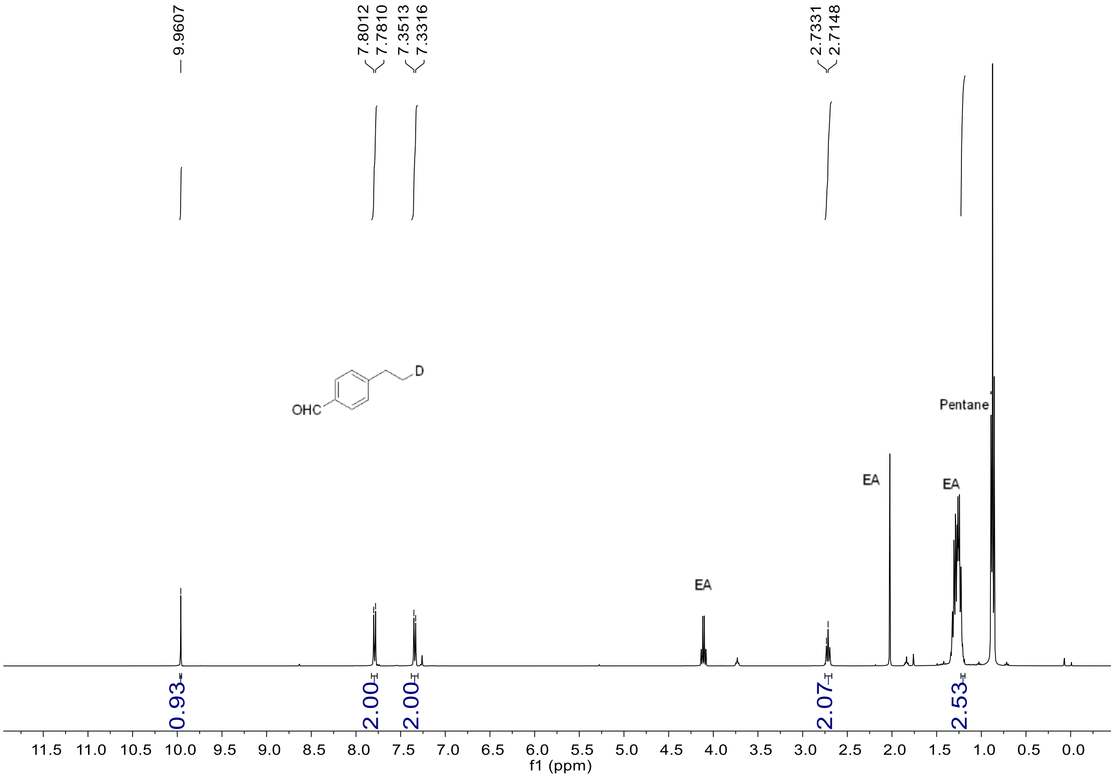


**2c:** $^{13}$C NMR (101 MHz, Chloroform-*d*)

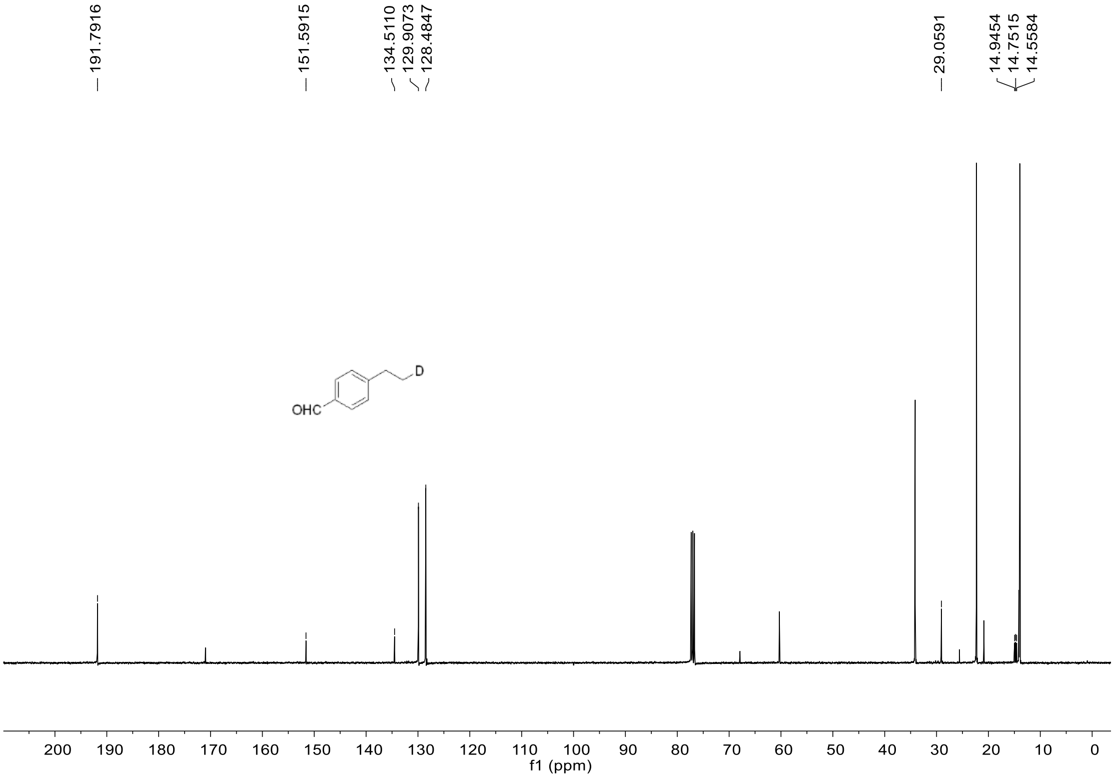

**3b:** $^{1}$H NMR (400 MHz, Chloroform-*d*)

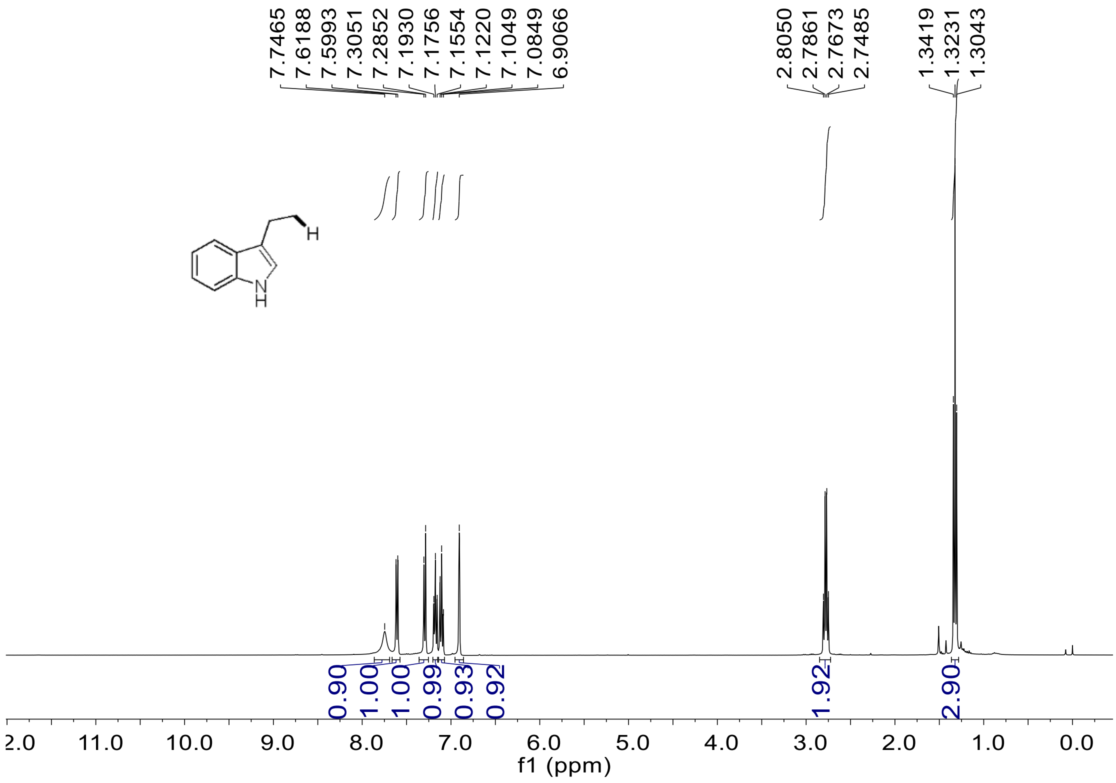


**3b:** $^{13}$C NMR (101 MHz, Chloroform-*d*)

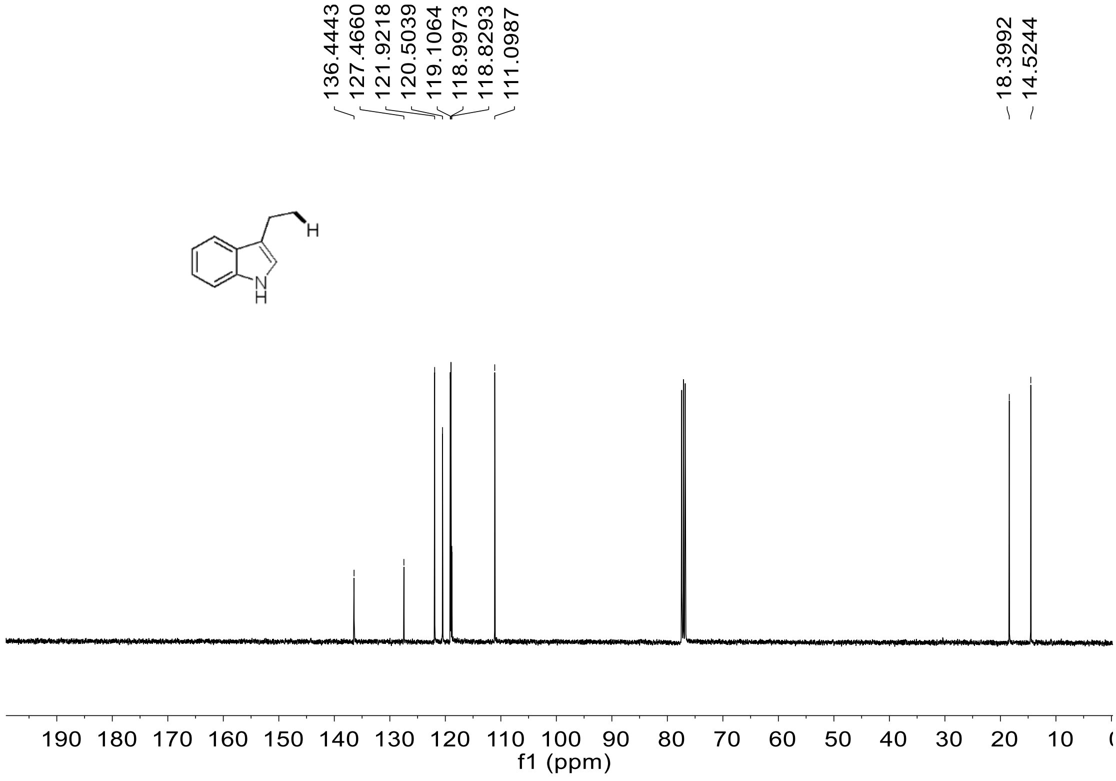

**3c:** $^{1}$H NMR (400 MHz, Chloroform-*d*)

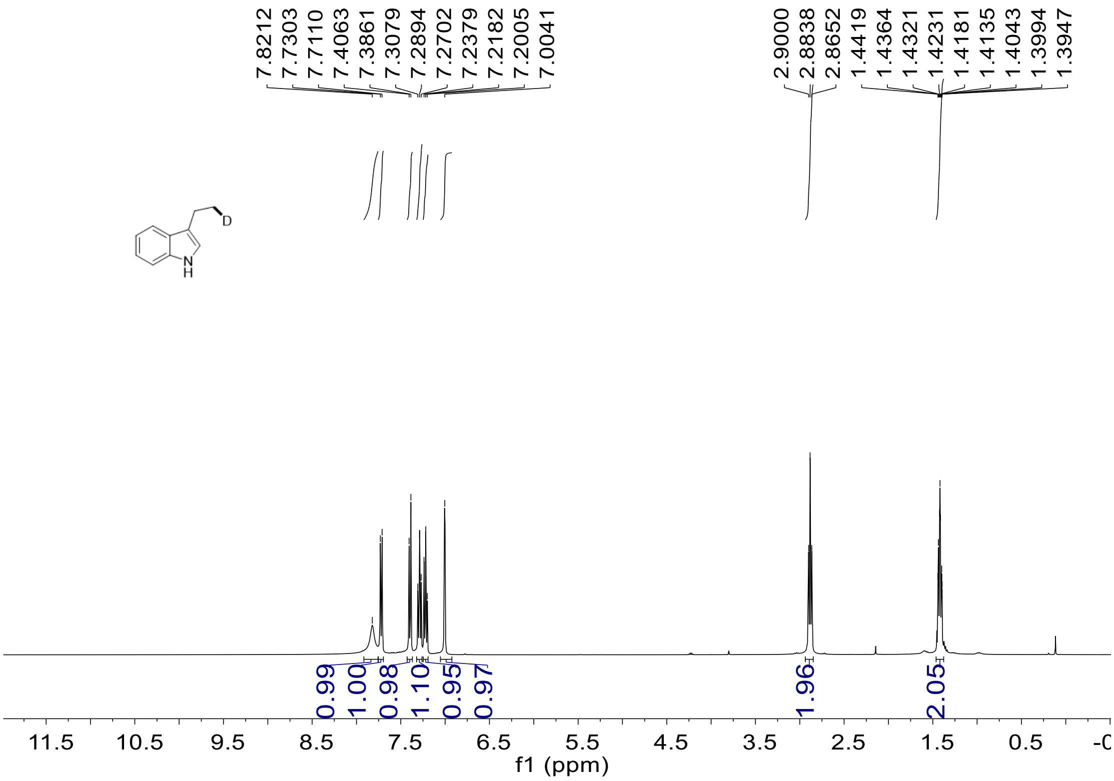


**3c:** $^{13}$C NMR (101 MHz, Chloroform-*d*)

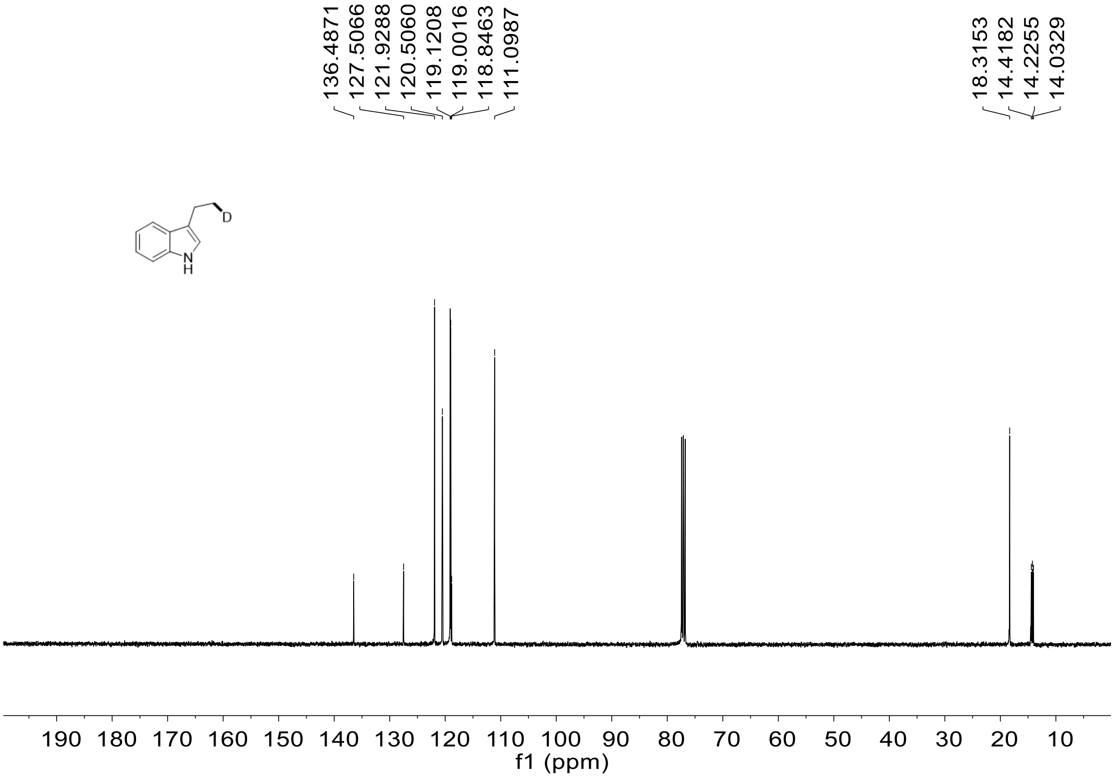

**4b:** $^{1}$H NMR (400 MHz, Chloroform-*d*)

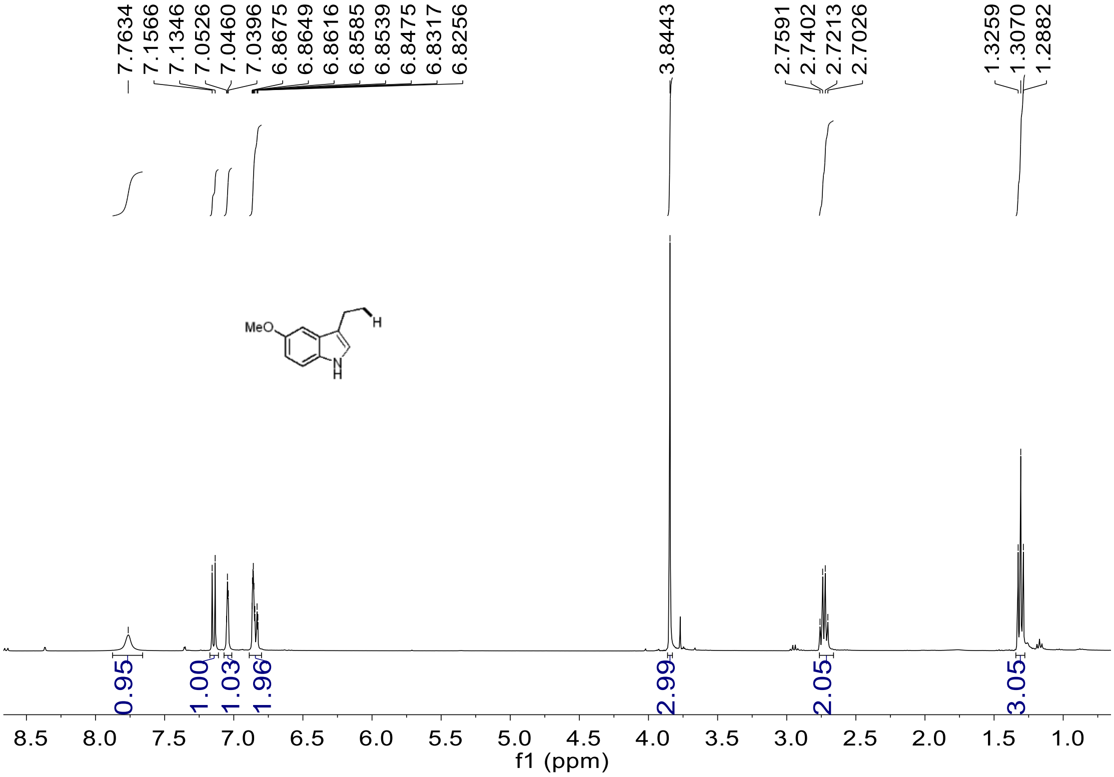


**4b:** $^{13}$C NMR (101 MHz, Chloroform-*d*)

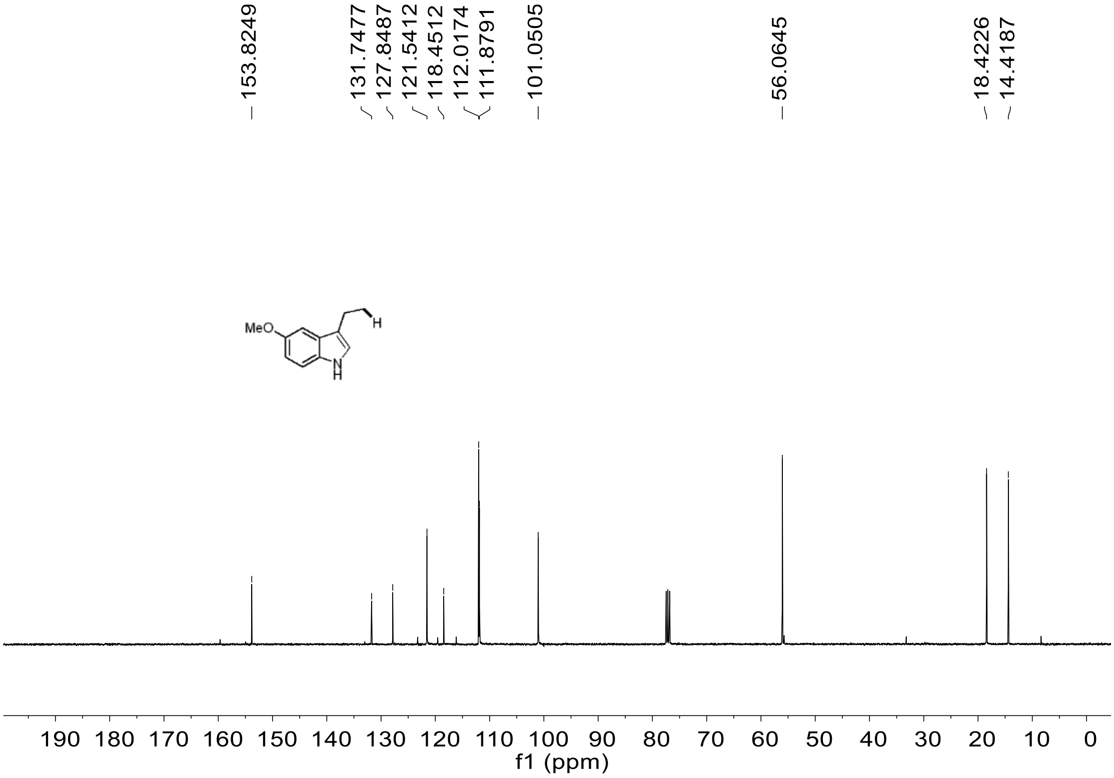

**4c:** $^{1}$H NMR (400 MHz, Chloroform-*d*)

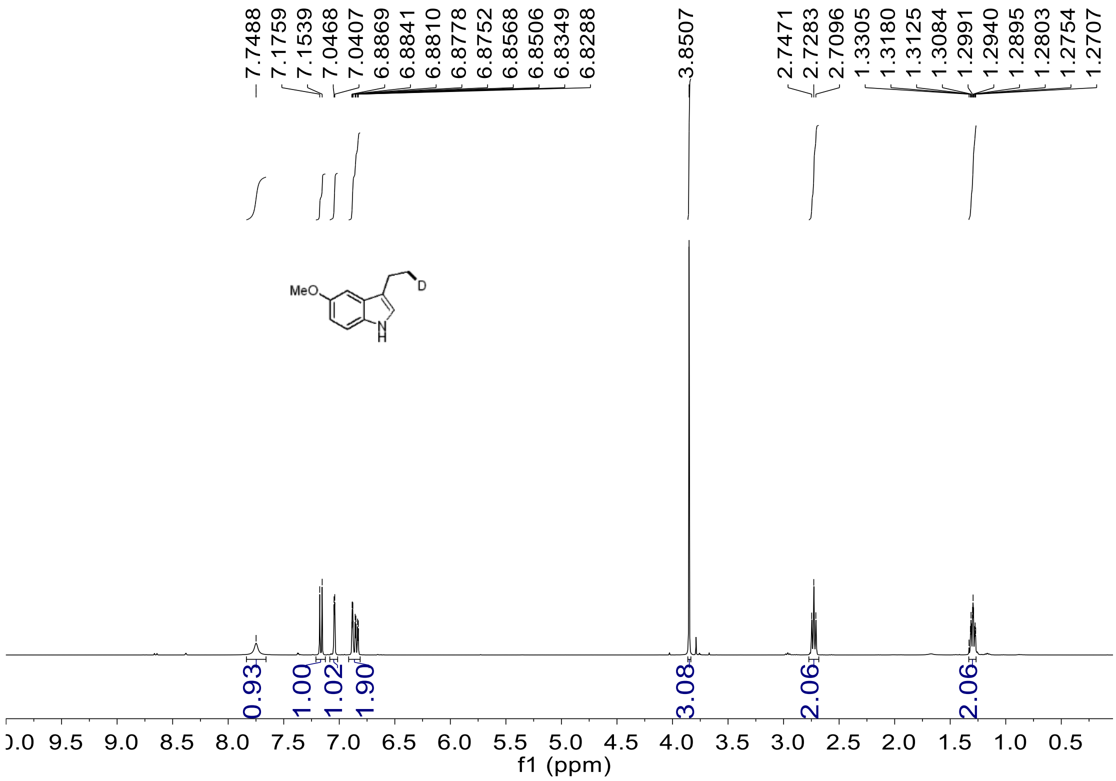


**4c:** $^{13}$C NMR (101 MHz, Chloroform-*d*)

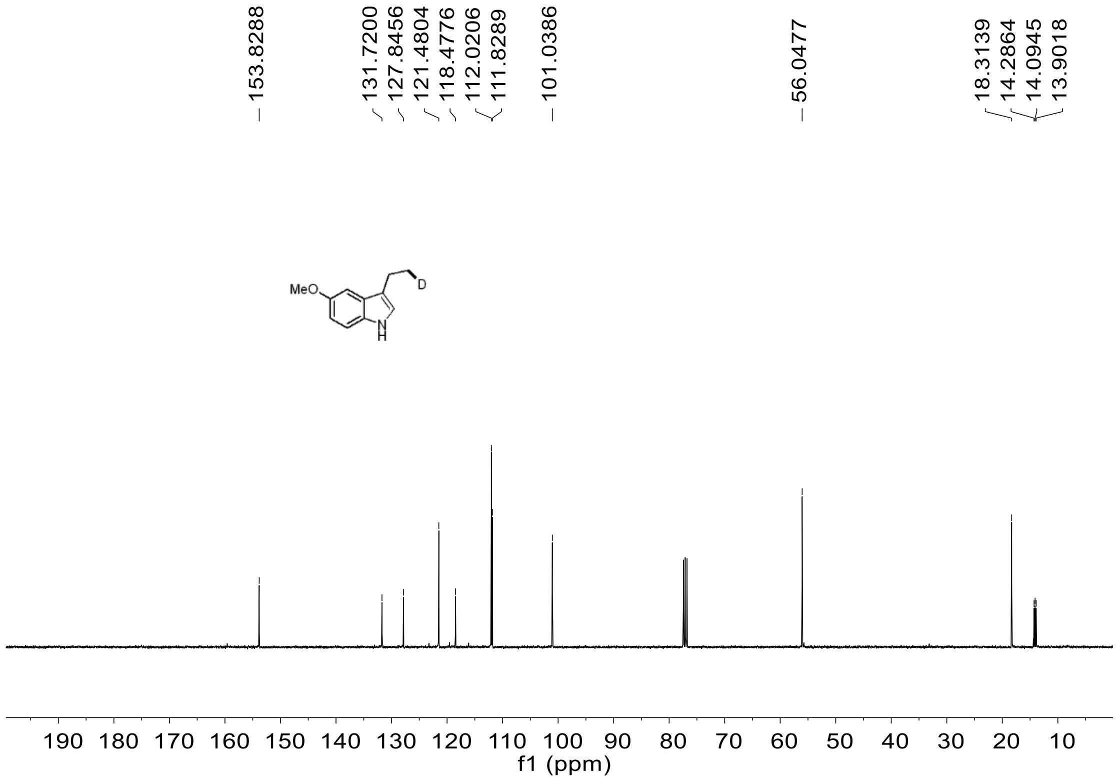

**5b:** $^{1}$H NMR (400 MHz, Chloroform-*d*)

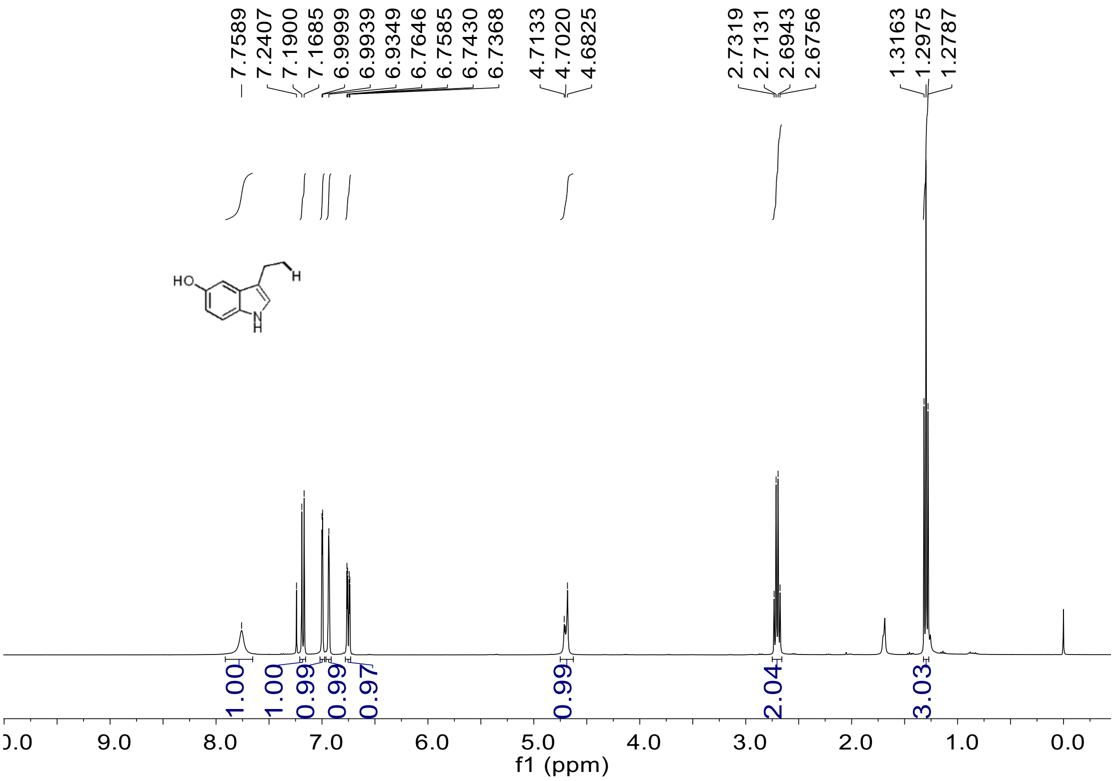


**5b:** $^{13}$C NMR (101 MHz, Chloroform-*d*)

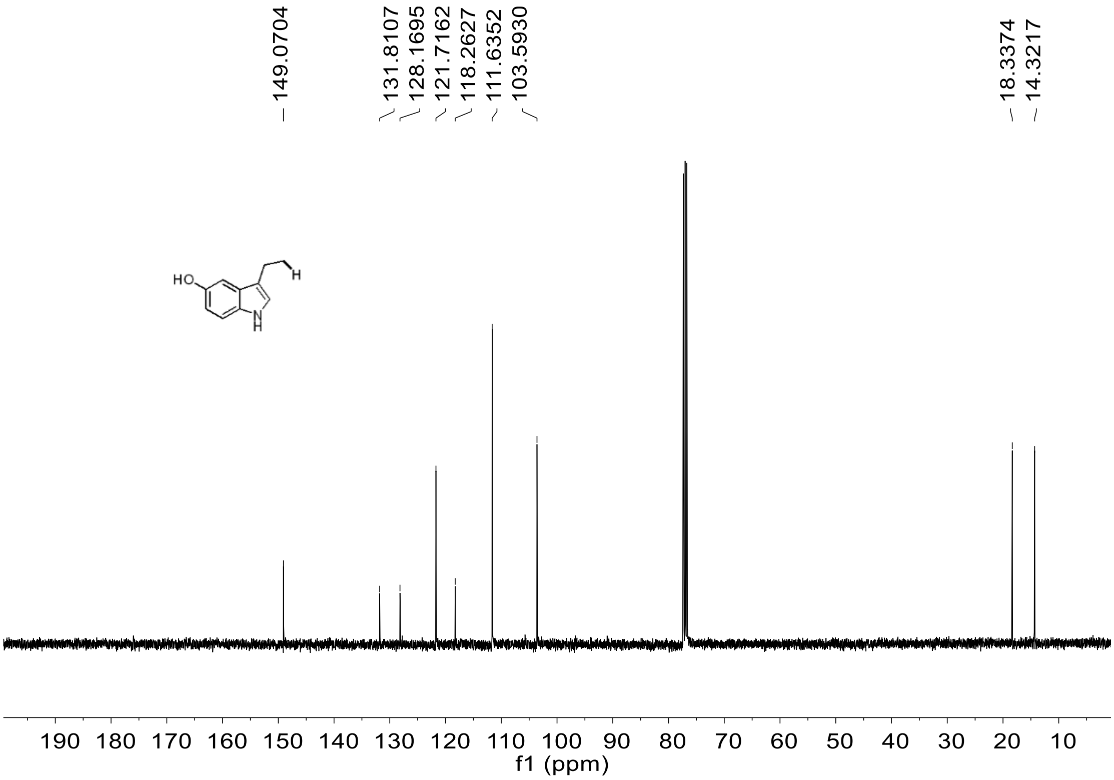

**5c:** $^{1}$H NMR (400 MHz, Chloroform-*d*)

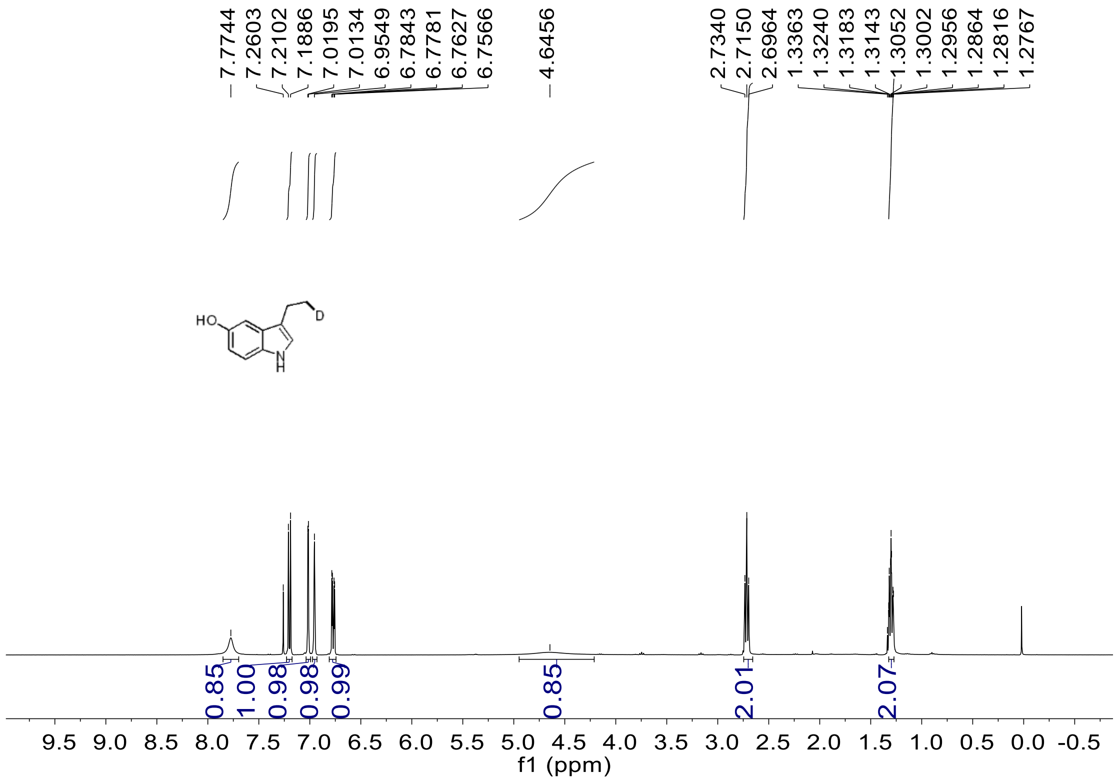


**5c:** $^{13}$C NMR (101 MHz, Chloroform-*d*)

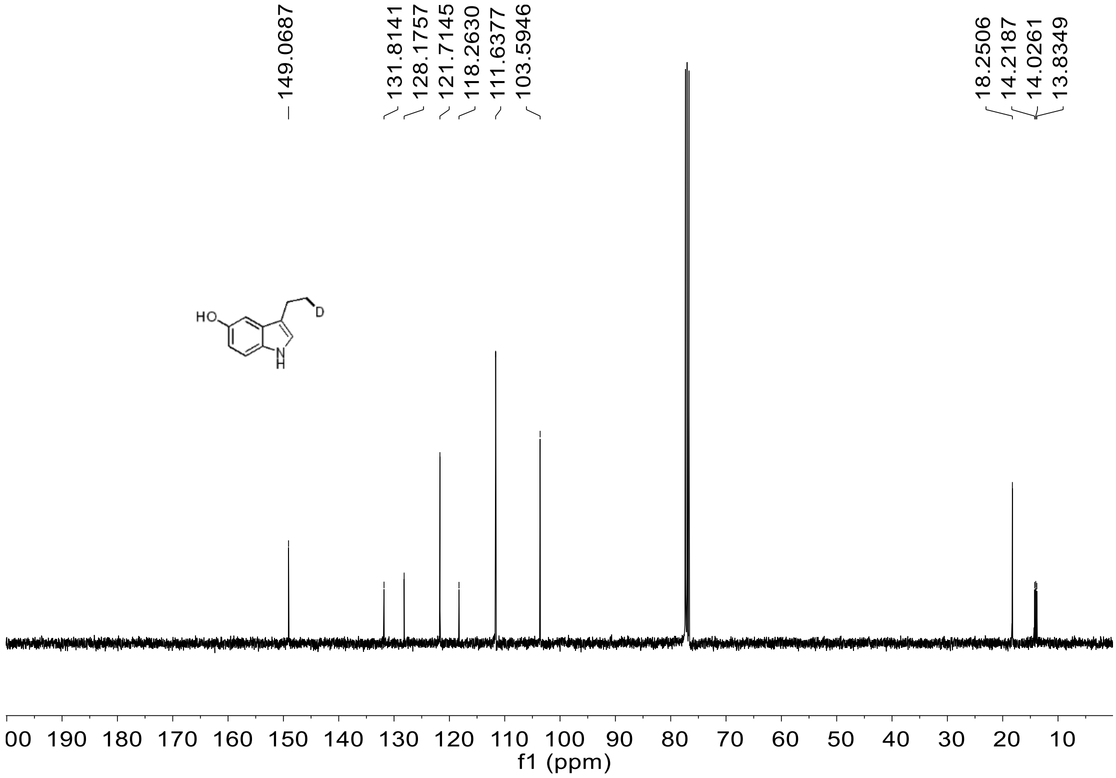

**6b:** $^{1}$H NMR (400 MHz, Chloroform-*d*)

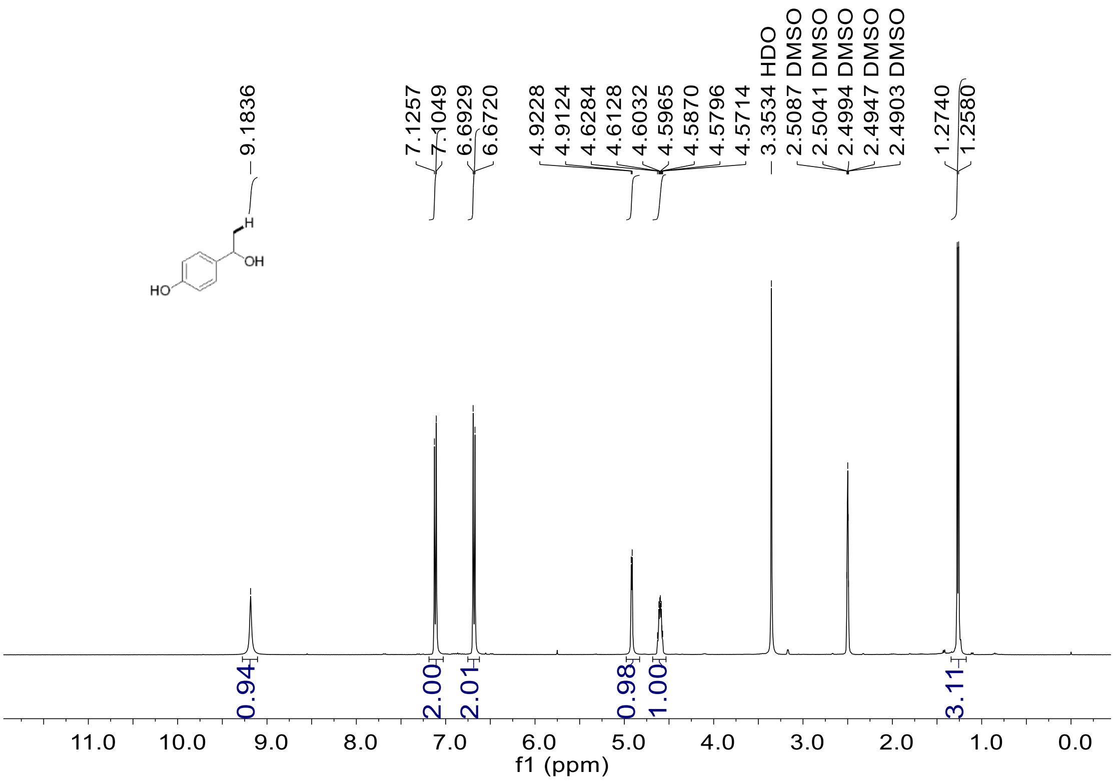


**6b:** $^{13}$C NMR (101 MHz, Chloroform-*d*)

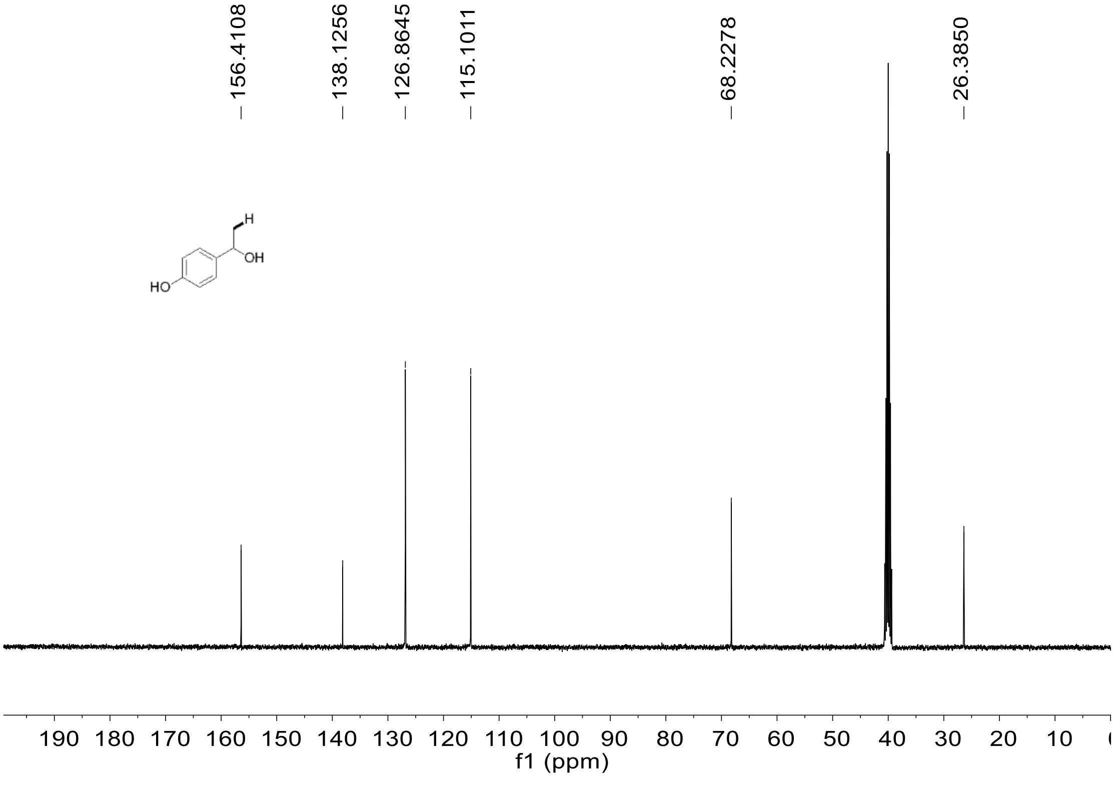

**6c:** $^{1}$H NMR (400 MHz, Chloroform-*d*)

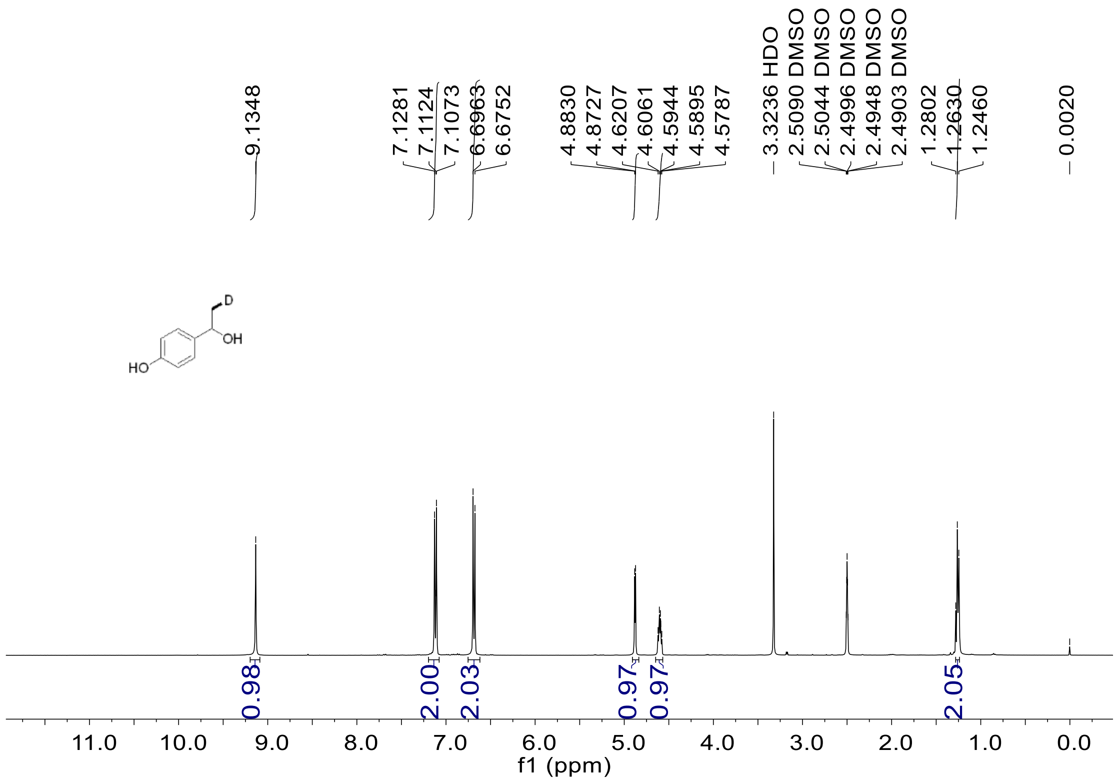


**6c:** $^{13}$C NMR (101 MHz, Chloroform-*d*)

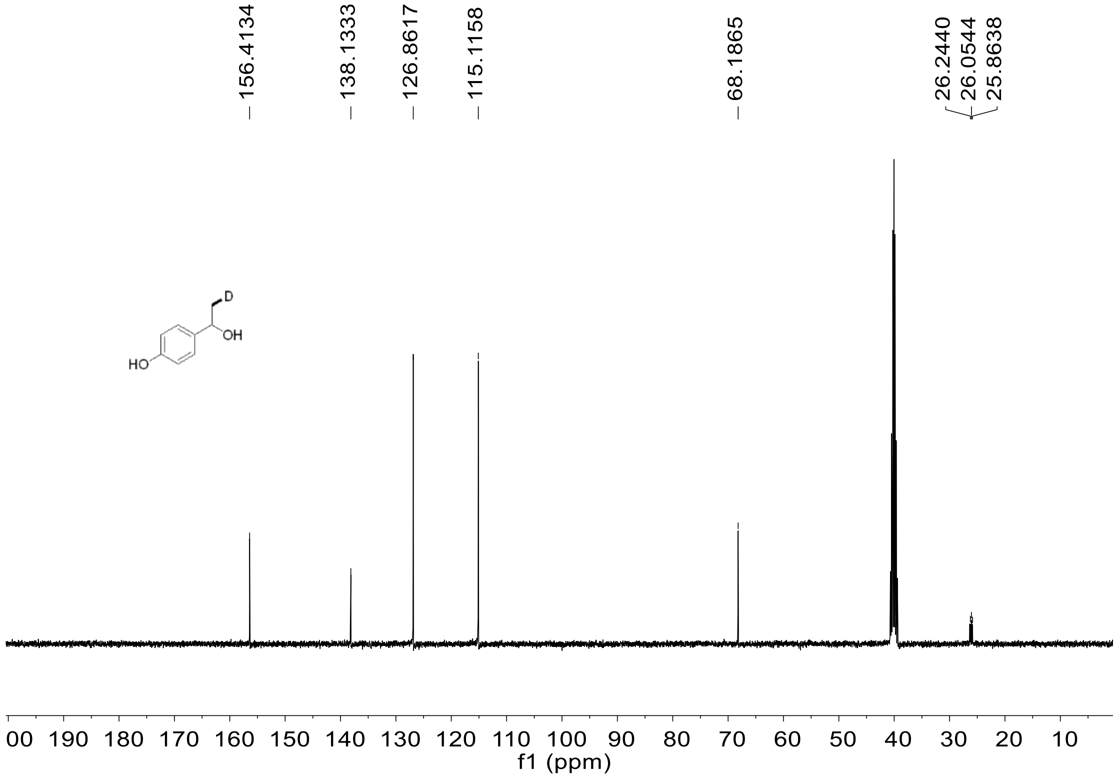

**7b:** $^{1}$H NMR (400 MHz, Chloroform-$d$)

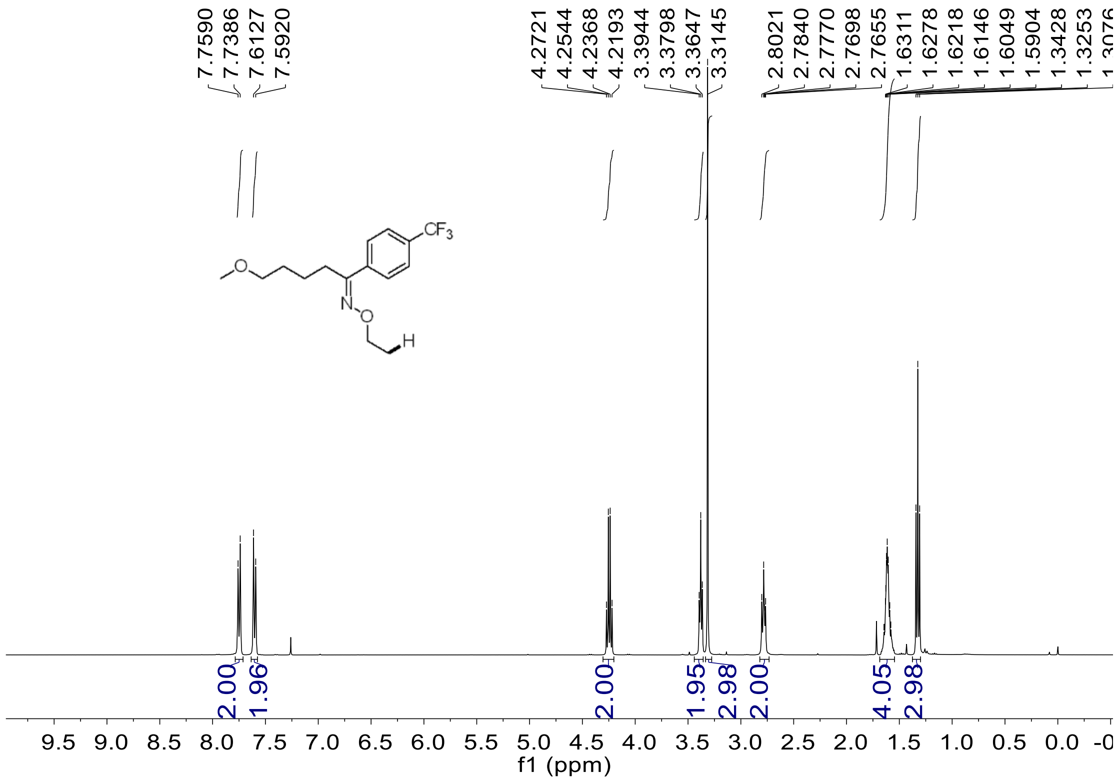


**7b:** $^{13}$C NMR (101 MHz, Chloroform-$d$)

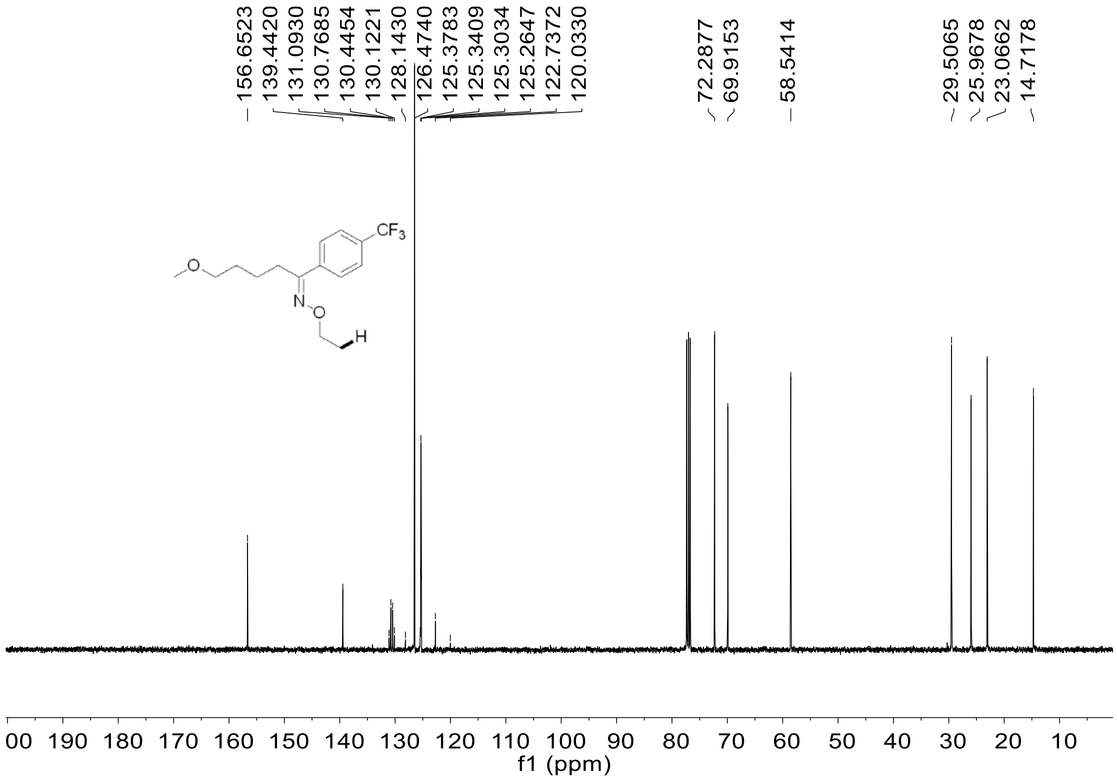

**7b:** $^{19}$F NMR (376 MHz, Chloroform-*d*)

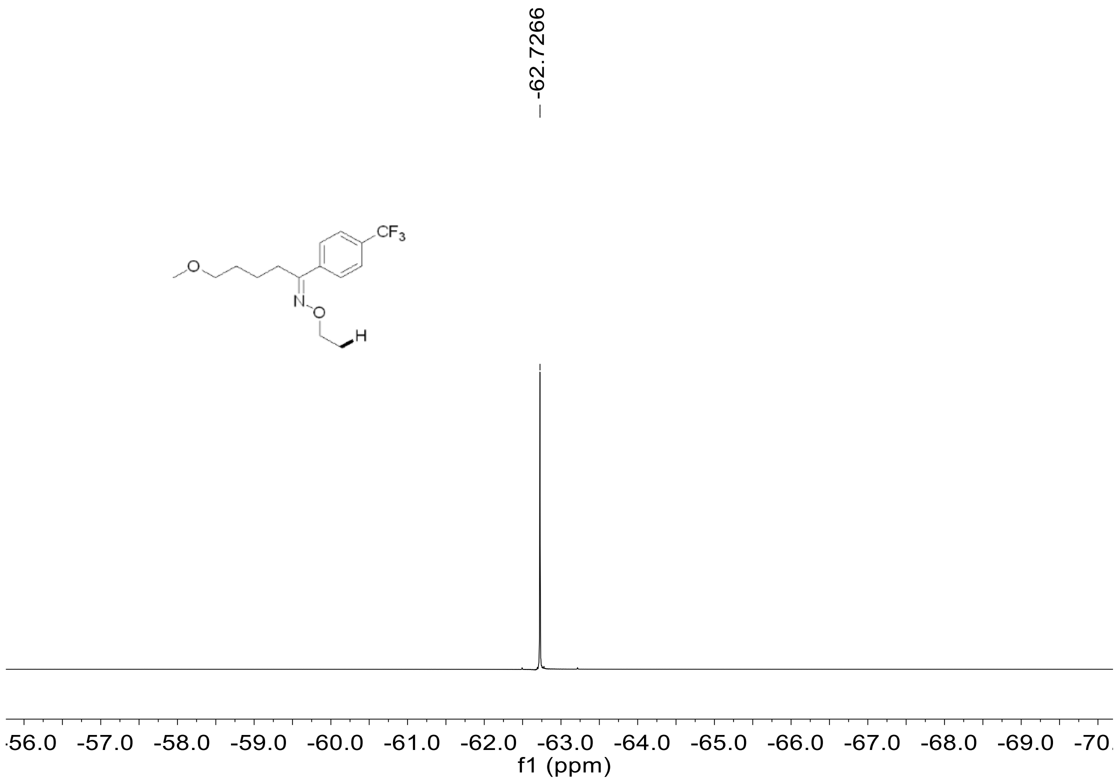

**7c:** $^{1}$H NMR (400 MHz, Chloroform-*d*)

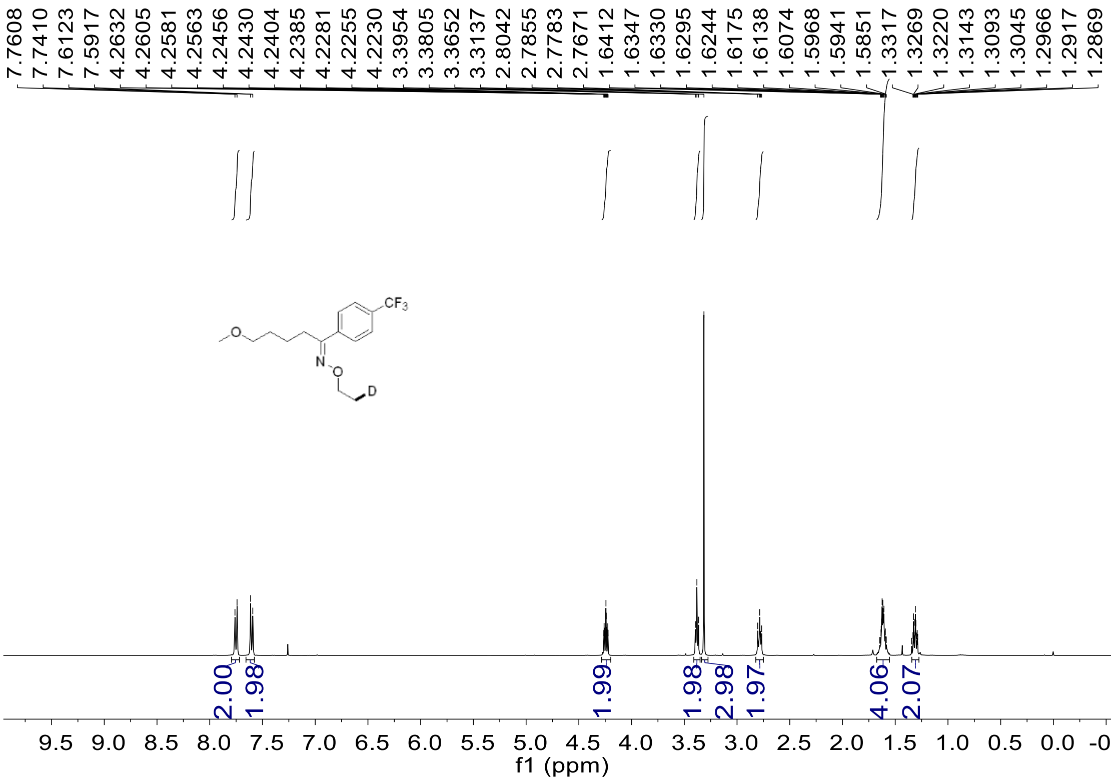


**7c:** $^{13}$C NMR (101 MHz, Chloroform-*d*)

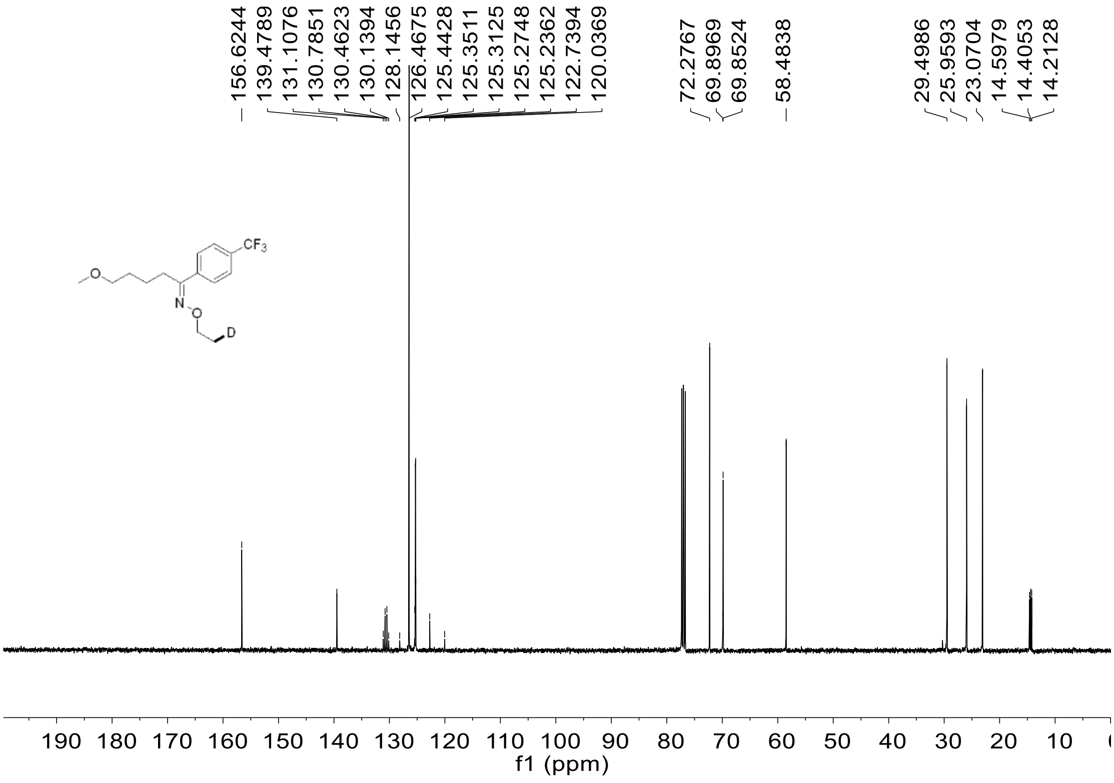

**7c:** $^{19}$F NMR (376 MHz, Chloroform-*d*)

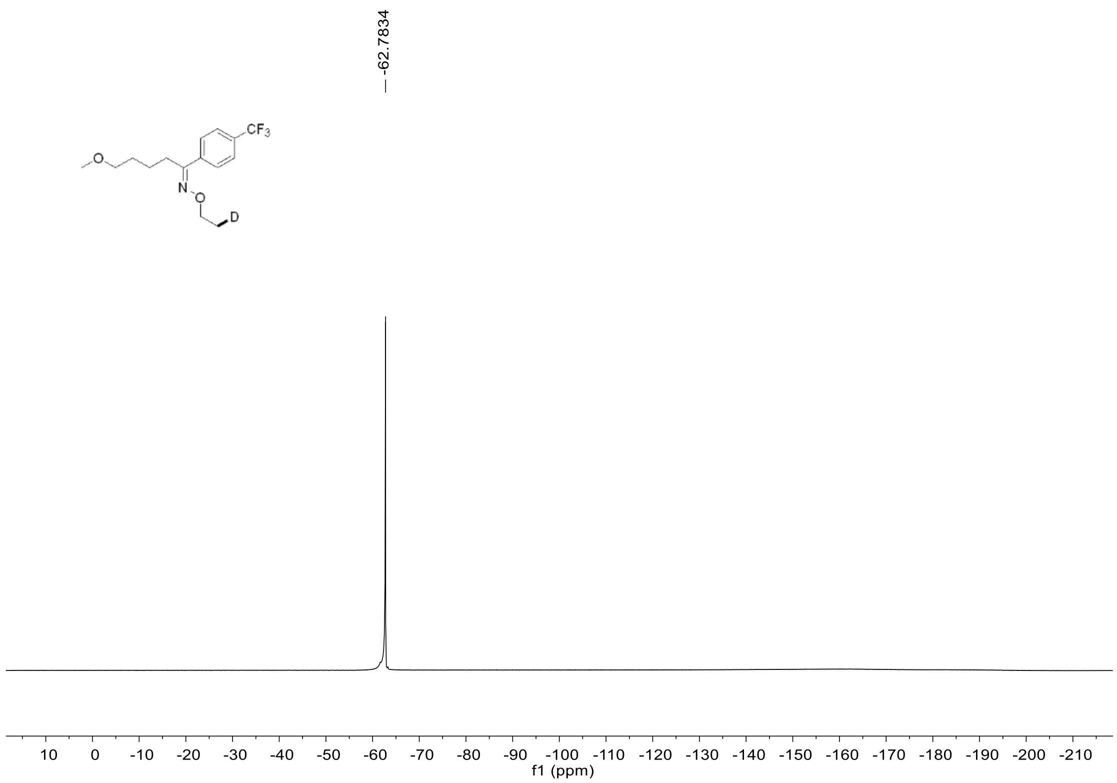

**8b:** $^{1}$H NMR (400 MHz, Chloroform-*d*)

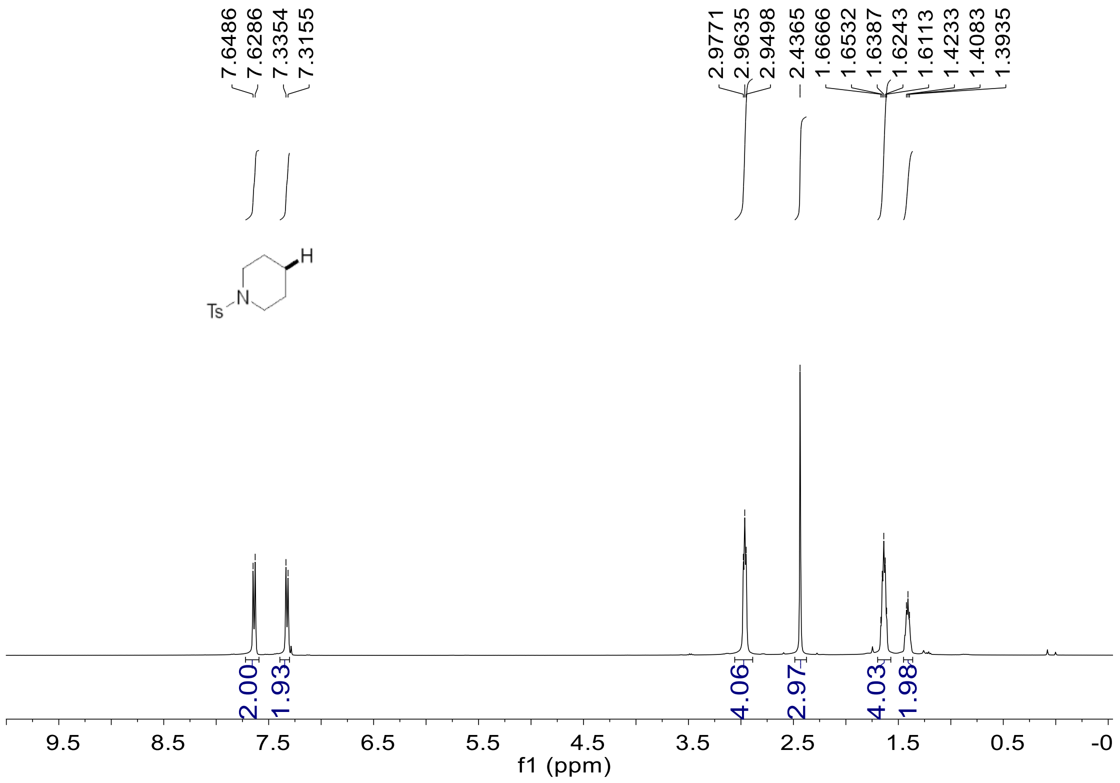


**8b:** $^{13}$C NMR (101 MHz, Chloroform-*d*)

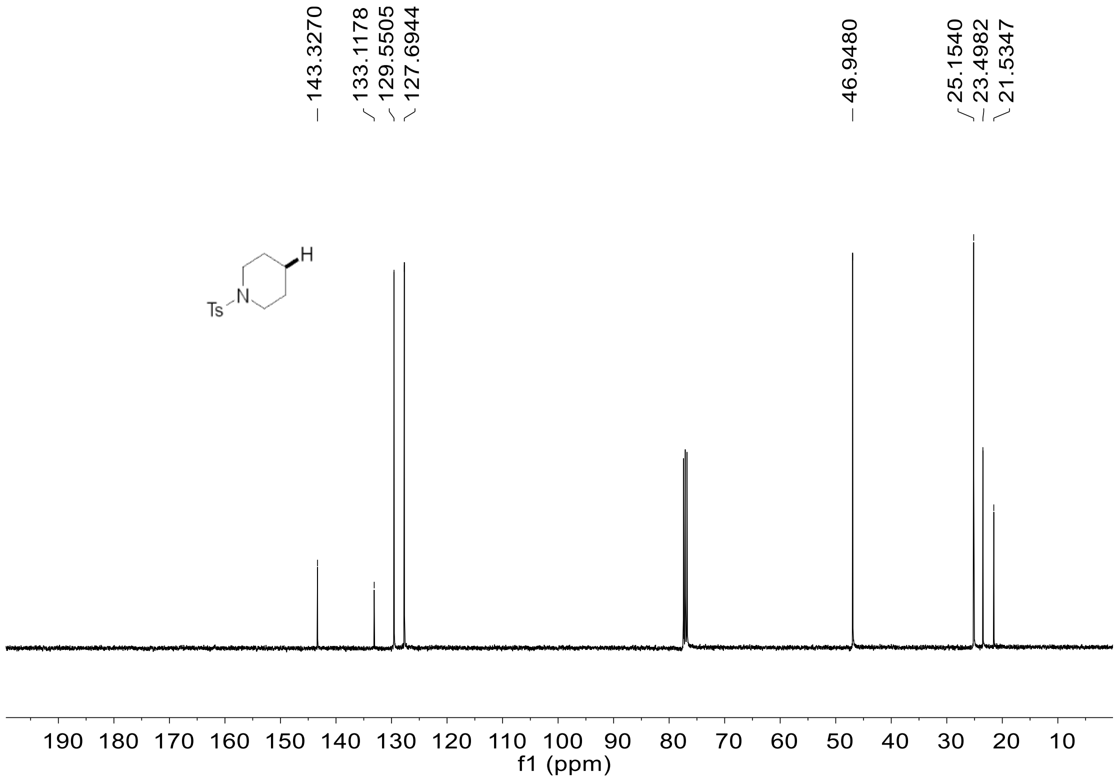

**8c:** $^{1}$H NMR (400 MHz, Chloroform-*d*)

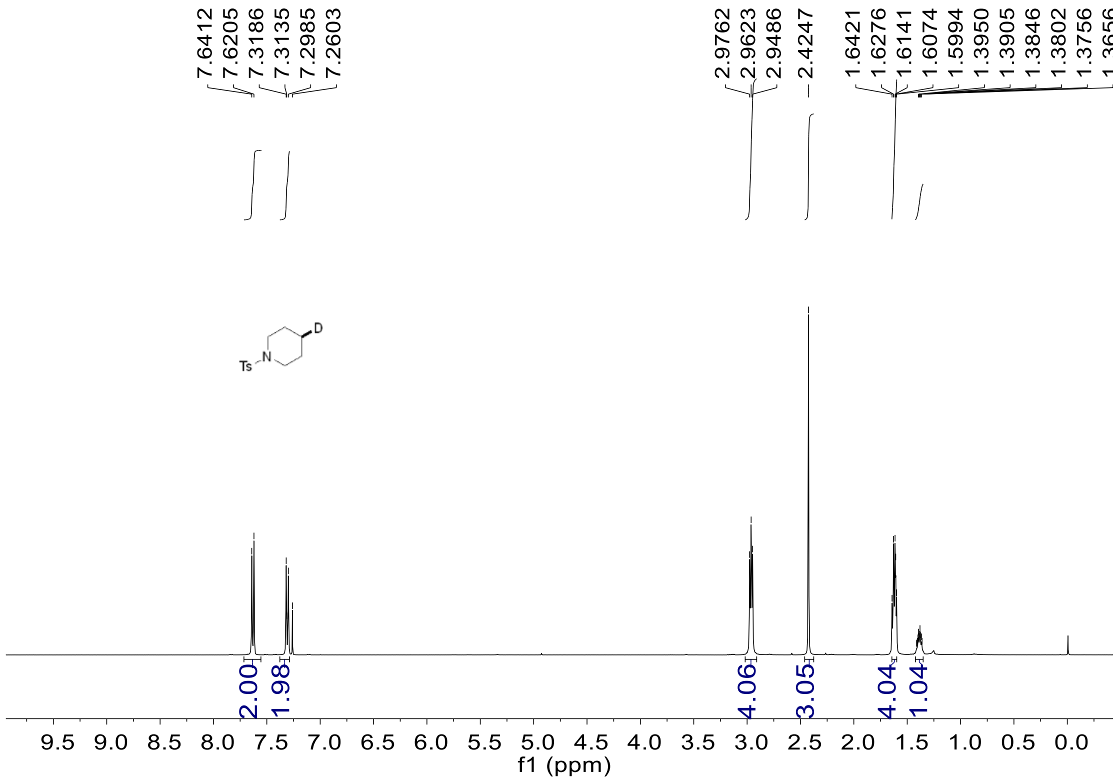


**8c:** $^{13}$C NMR (101 MHz, Chloroform-*d*)

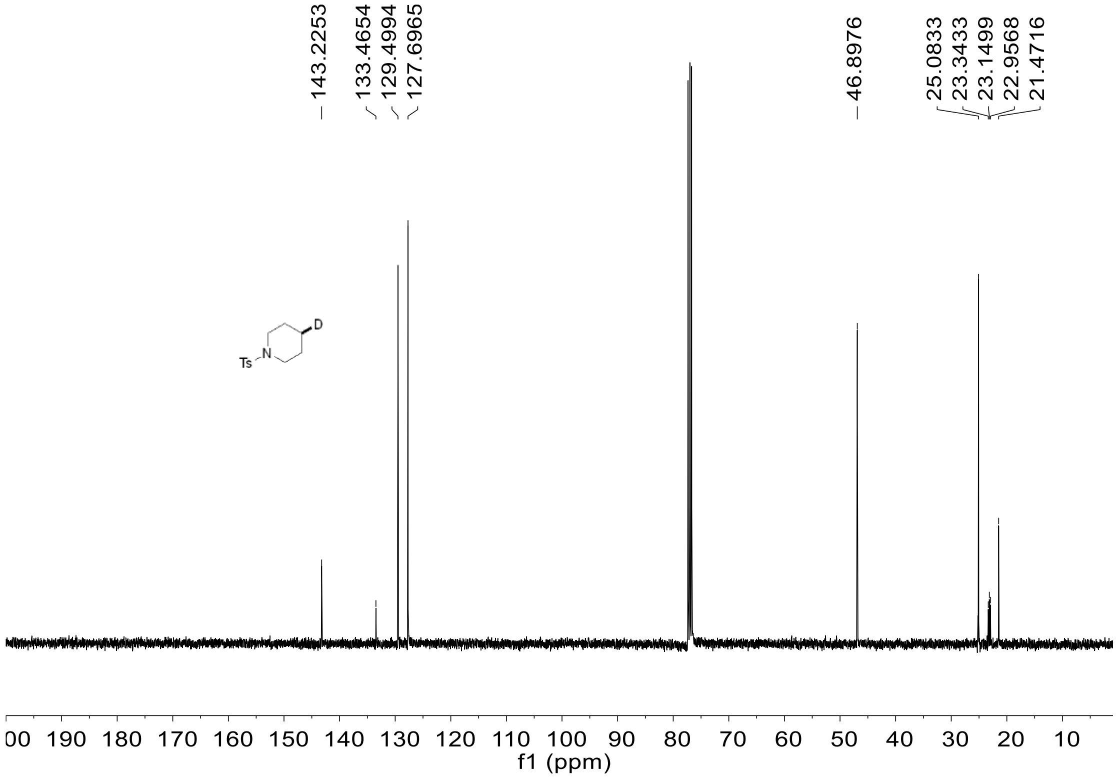

**9b:** $^{1}$H NMR (400 MHz, Chloroform-*d*)

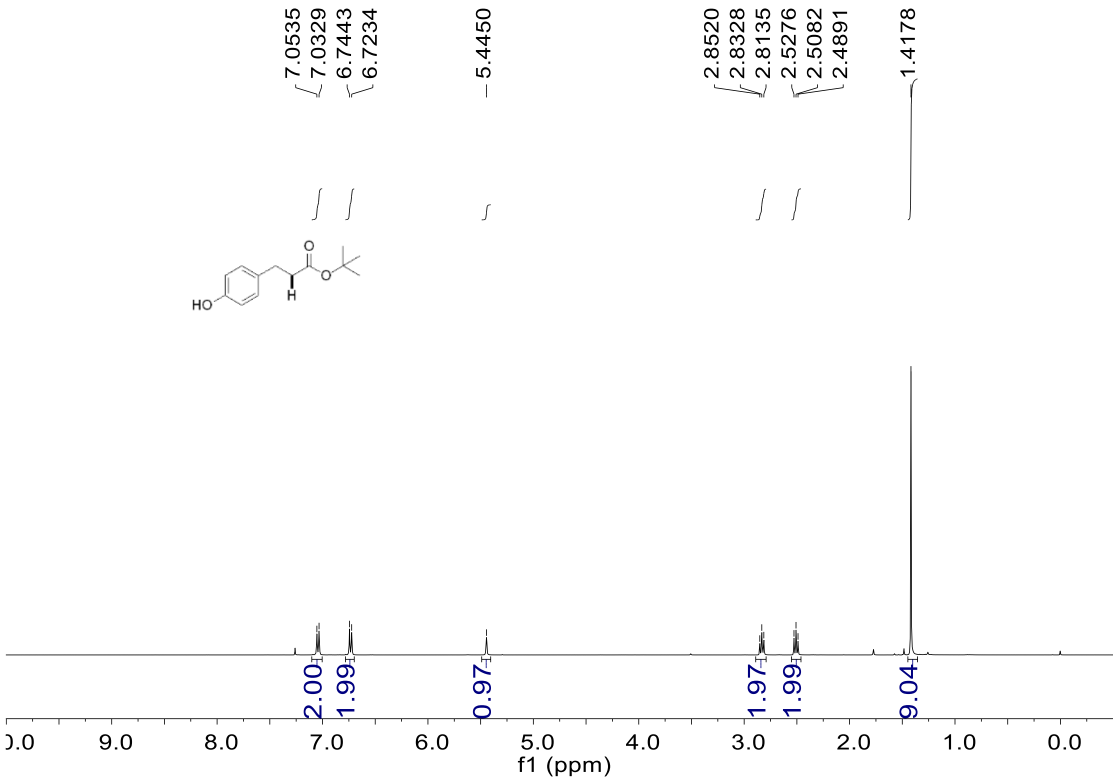


**9b:** $^{13}$C NMR (101 MHz, Chloroform-*d*)

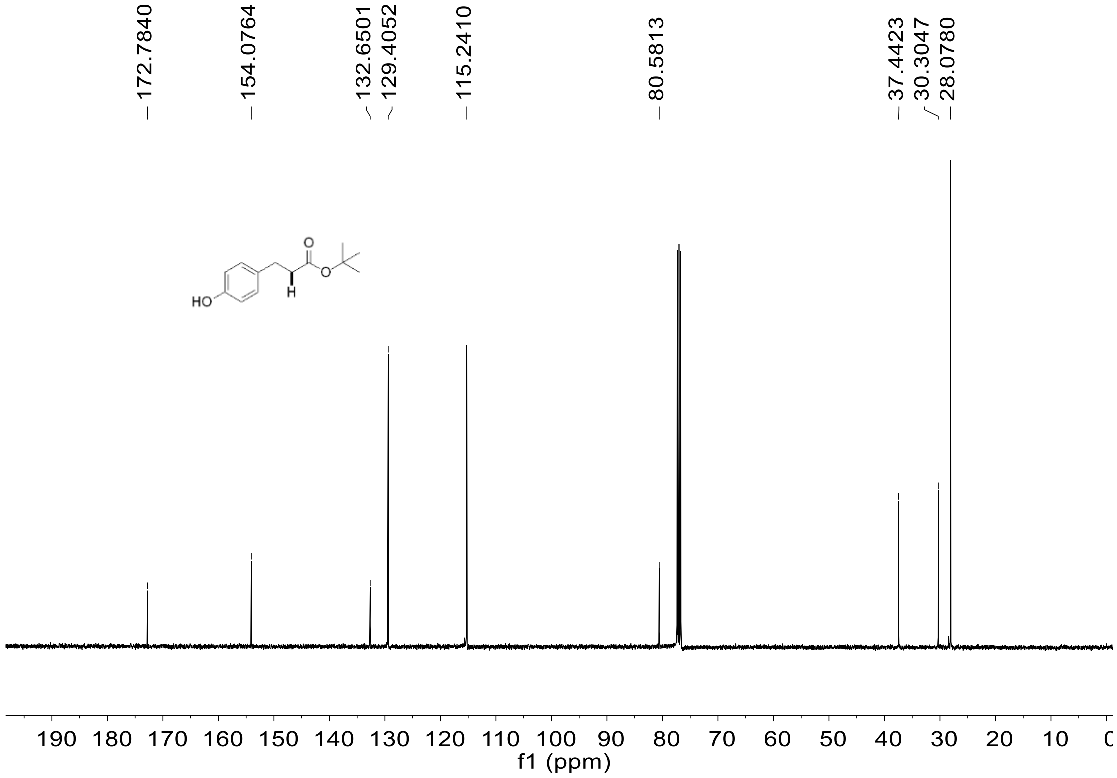

**9c:** $^{1}$H NMR (400 MHz, Chloroform-*d*)

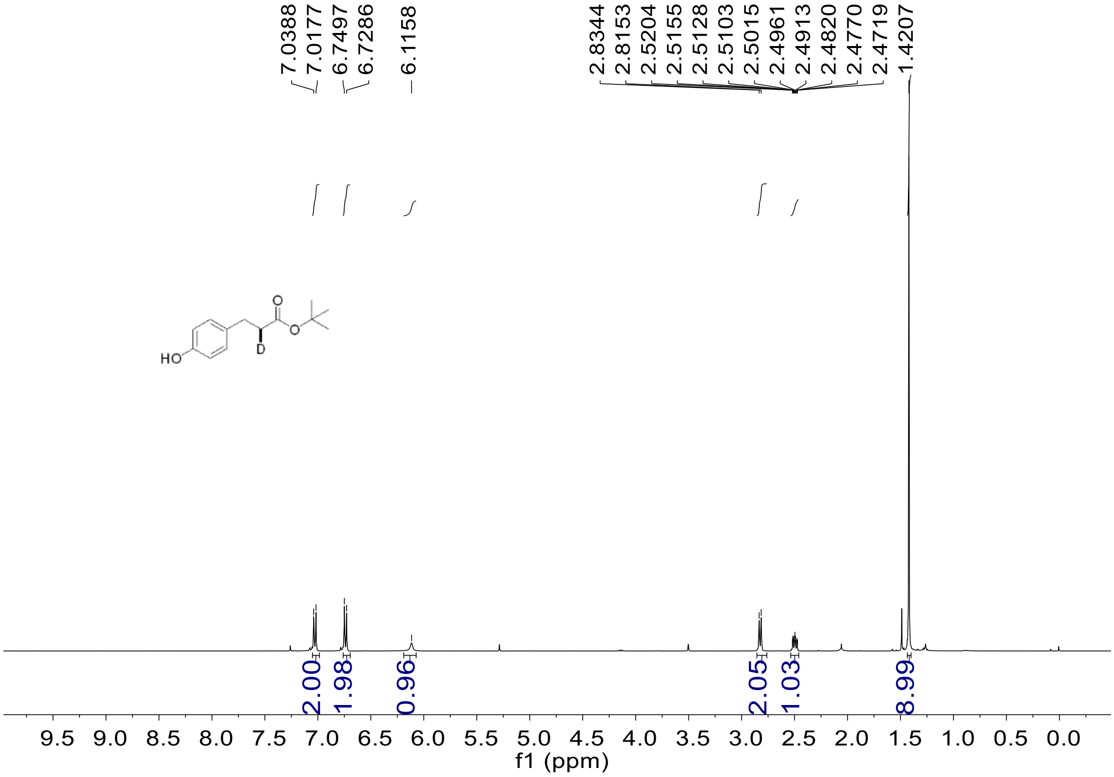


**9c:** $^{13}$C NMR (101 MHz, Chloroform-*d*)

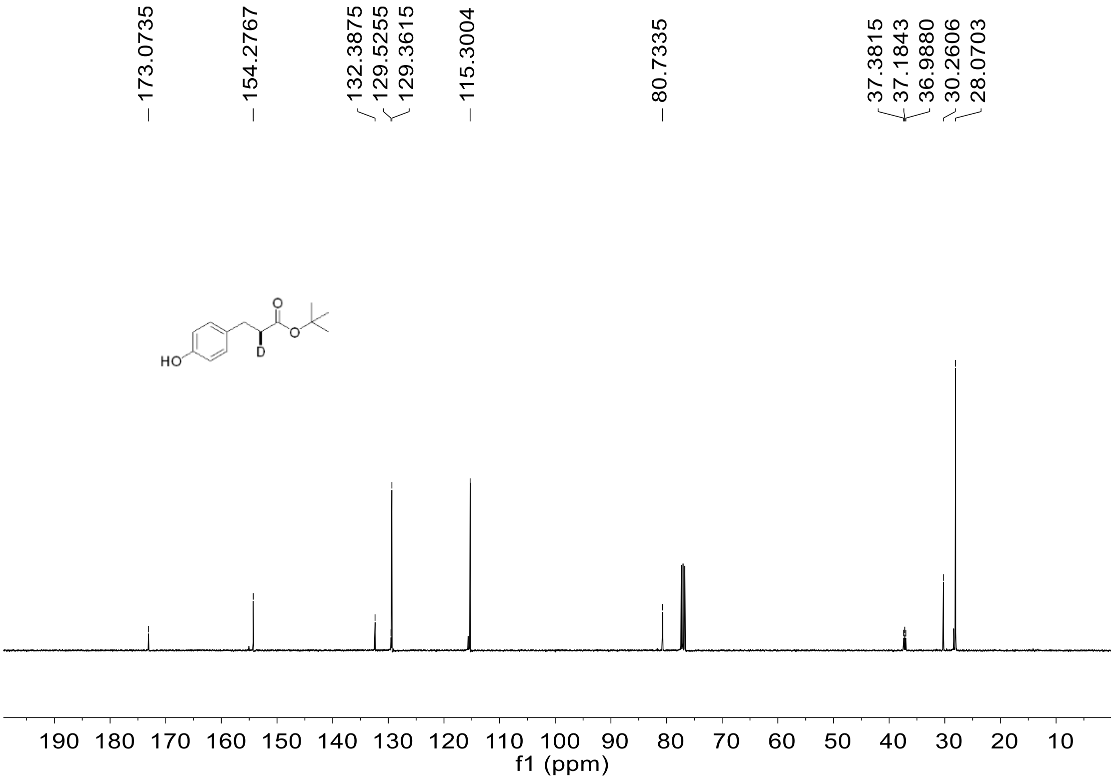

**10b:** $^{1}$H NMR (400 MHz, Chloroform-*d*)

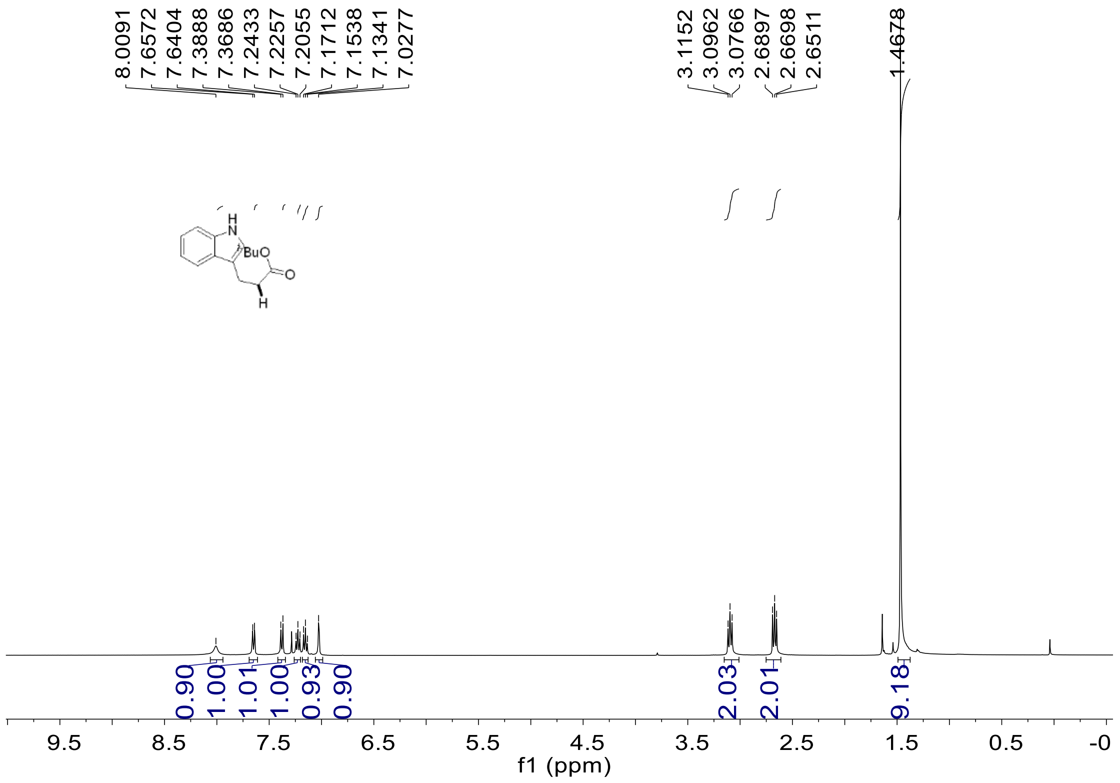


**10b:** $^{13}$C NMR (101 MHz, Chloroform-*d*)

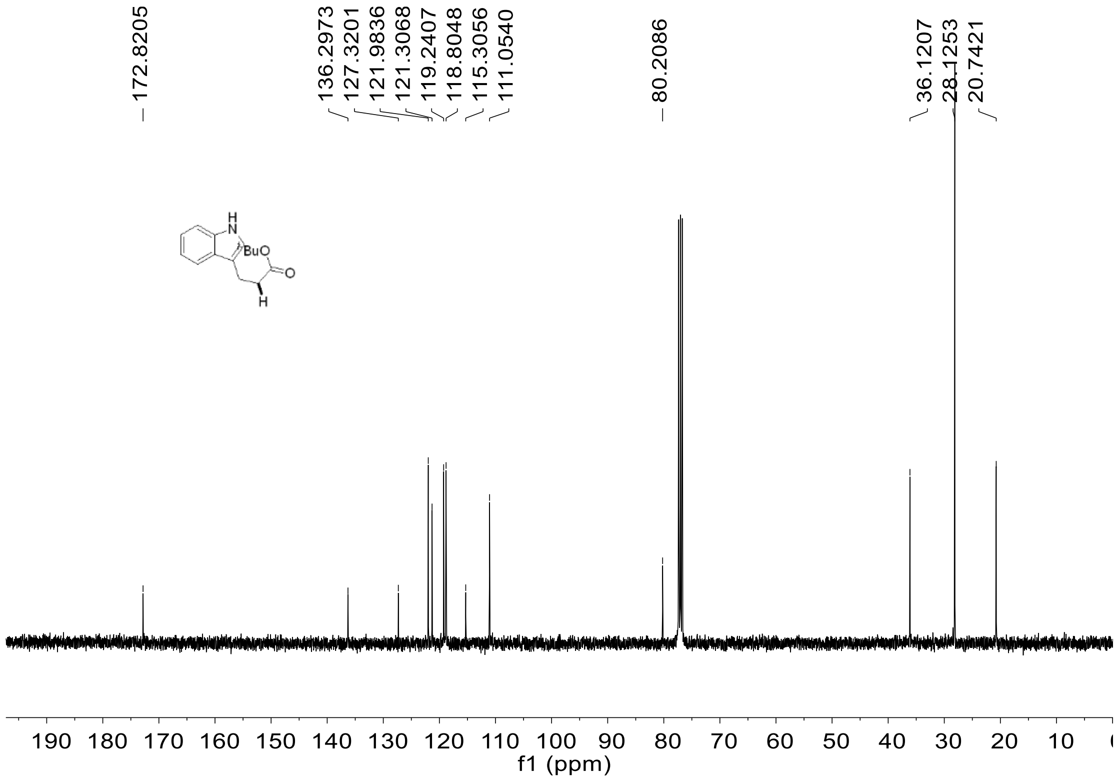

**10c:** $^{1}$H NMR (400 MHz, Chloroform-*d*)

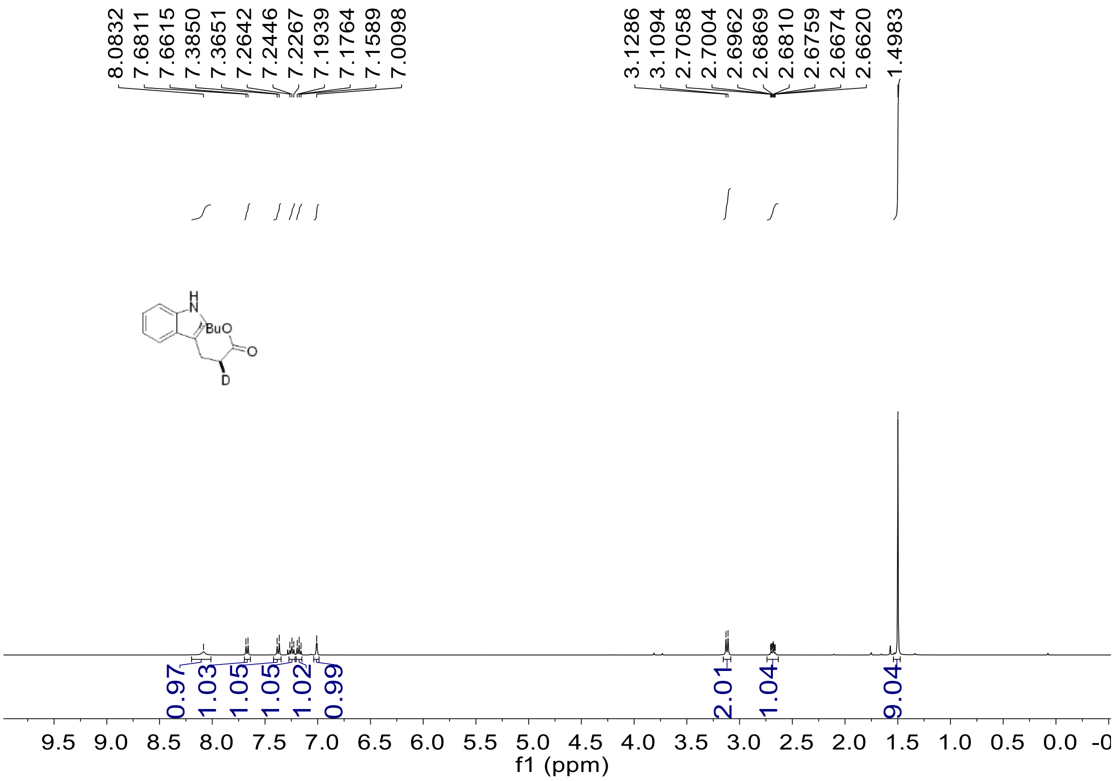


**10c:** $^{13}$C NMR (101 MHz, Chloroform-*d*)

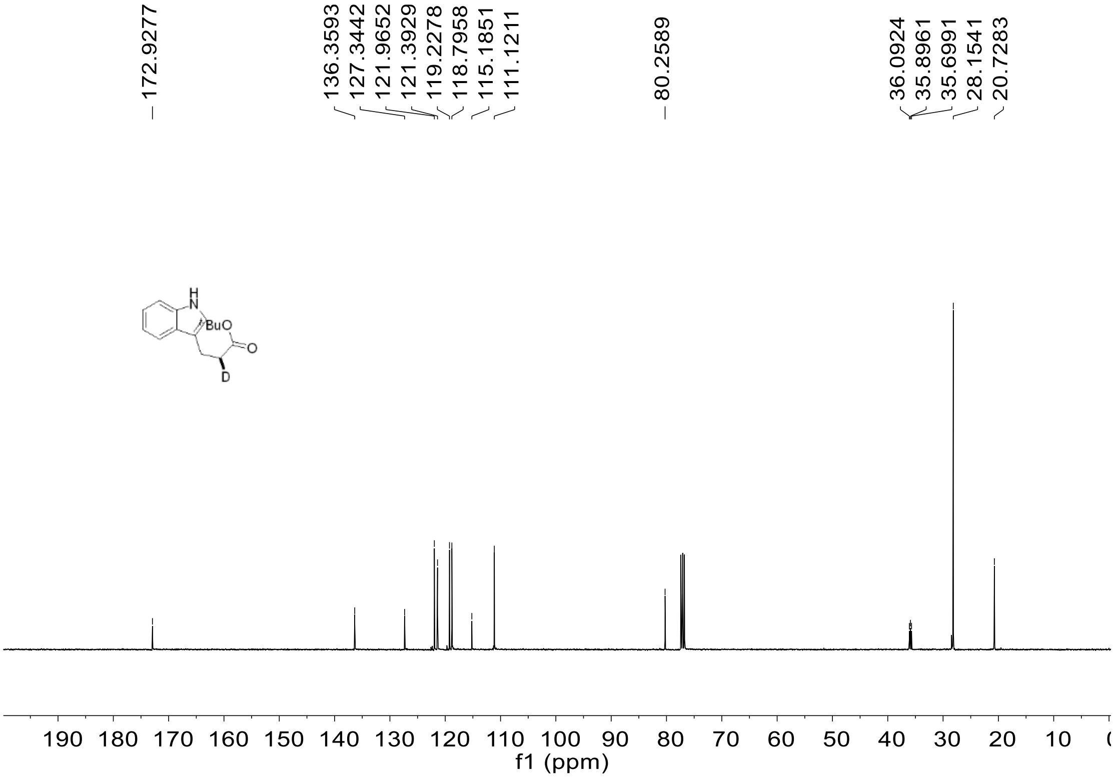

**11b:** $^{1}H$ NMR (400 MHz, DMSO-$d_6$)

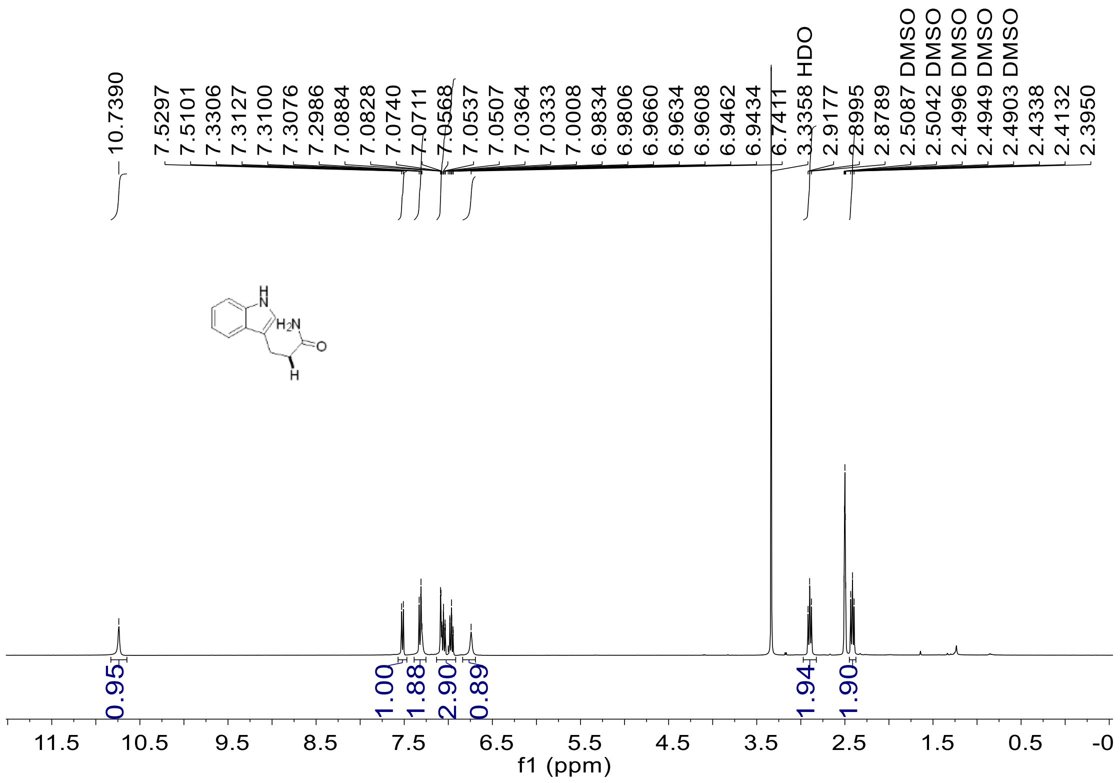

**11b:** $^{13}C$ NMR (101 MHz, DMSO-$d_6$)

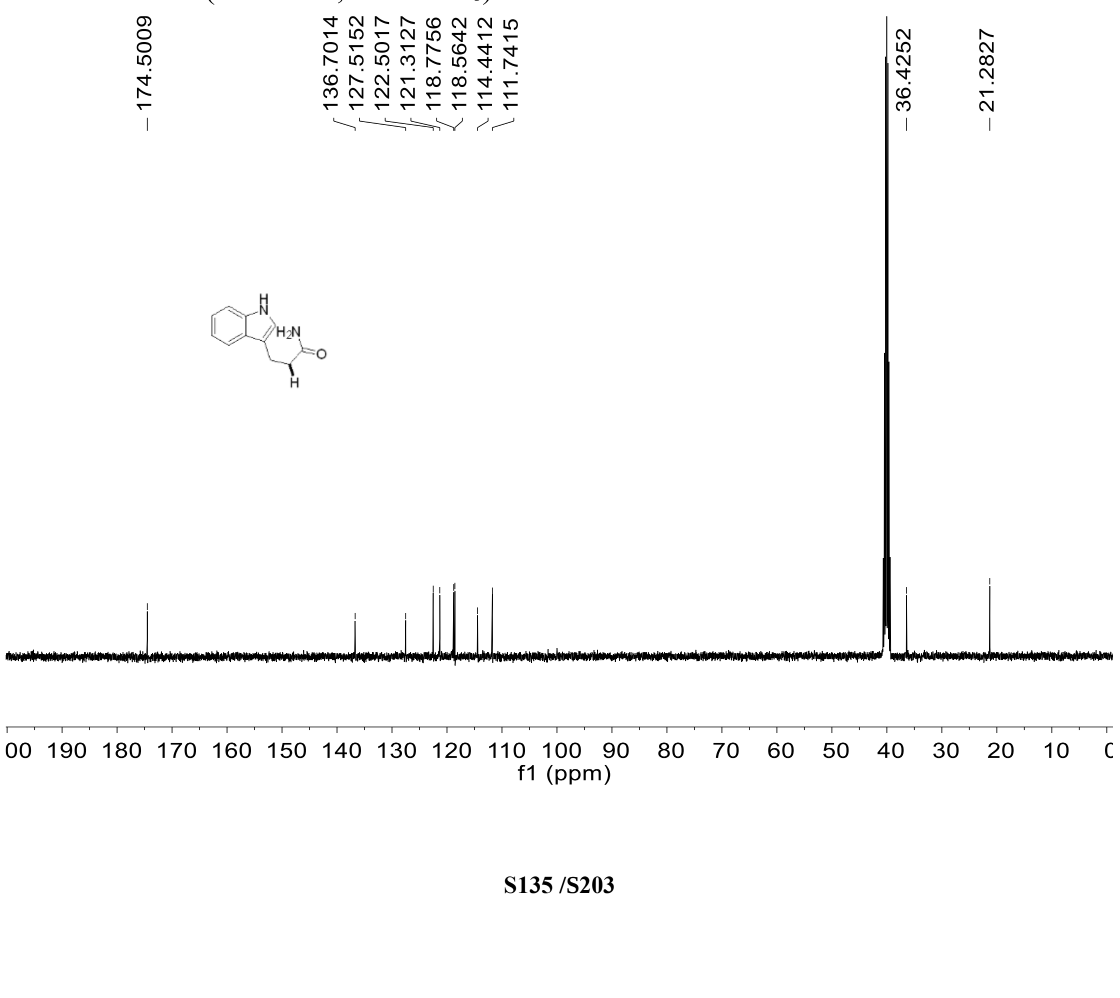

**11c:** $^{1}$H NMR (400 MHz, DMSO-$d_6$)

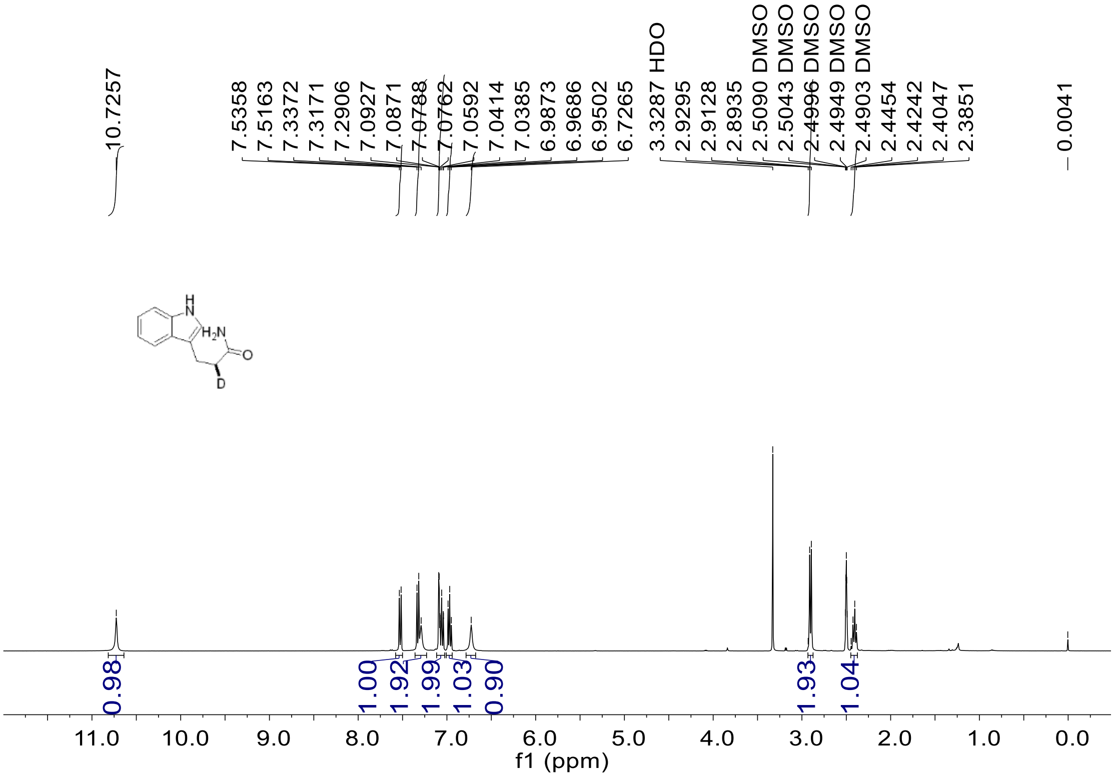


**11c:** $^{13}$C NMR (101 MHz, DMSO-$d_6$)

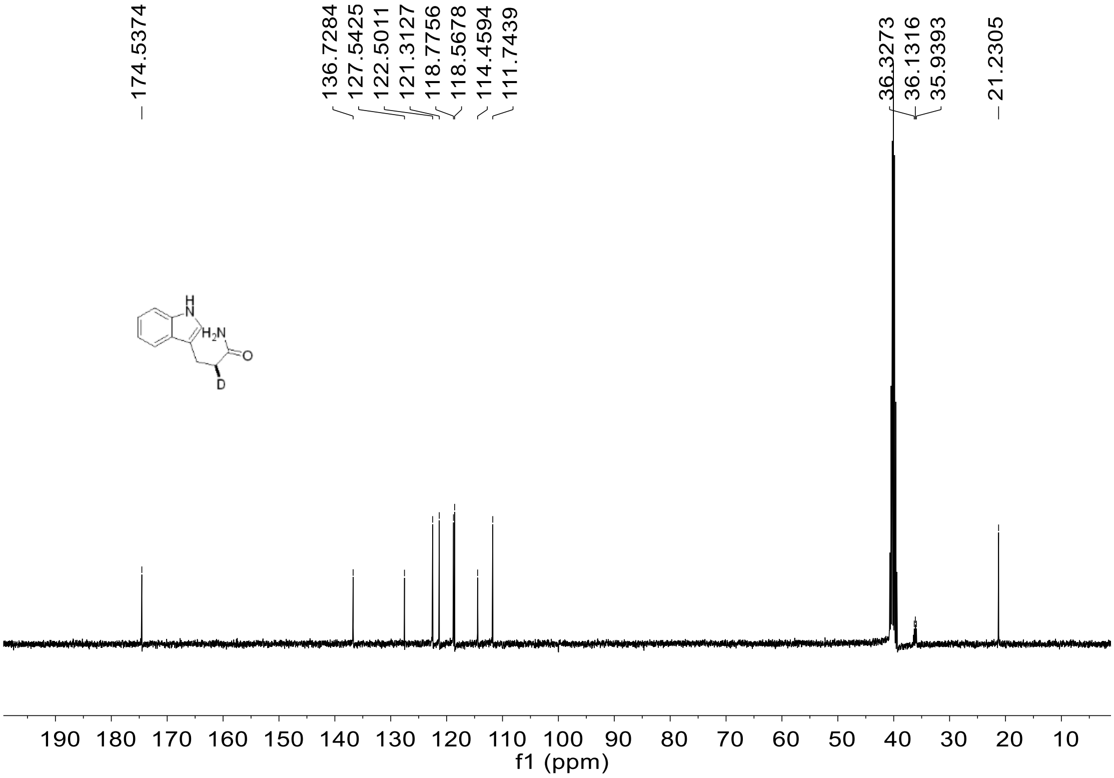

**12b:** $^{1}$H NMR (400 MHz, DMSO-$d_6$)

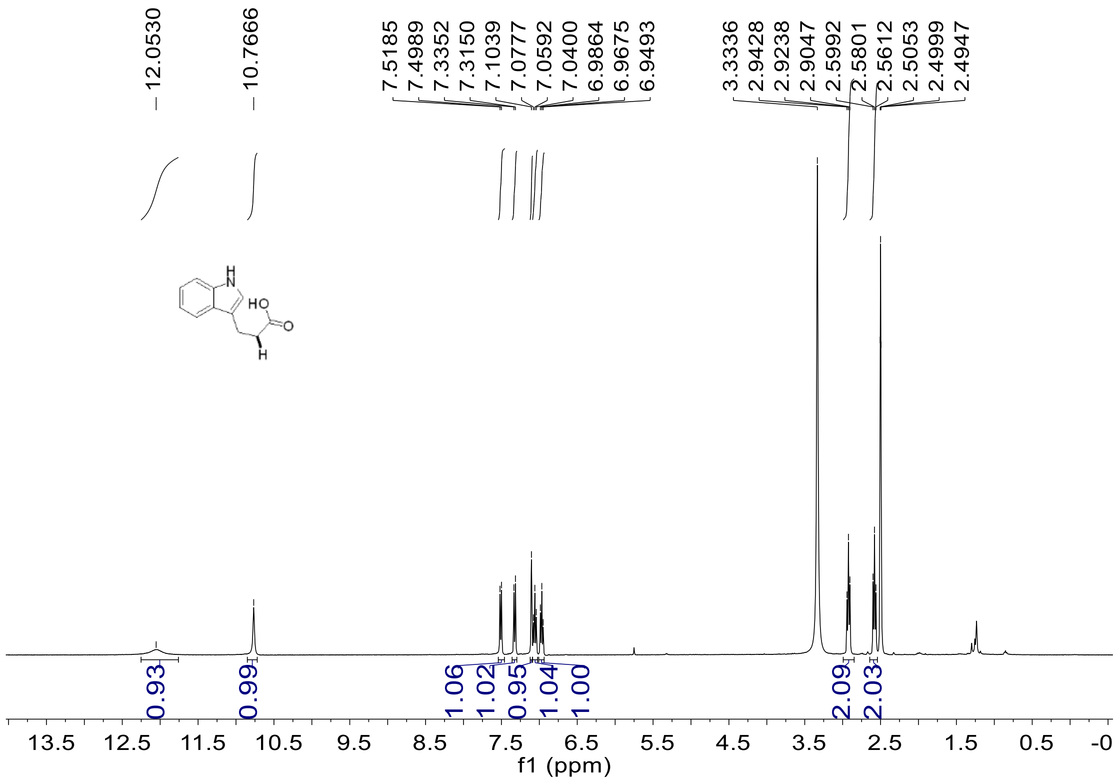


**12b:** $^{13}$C NMR (101 MHz, DMSO-$d_6$)

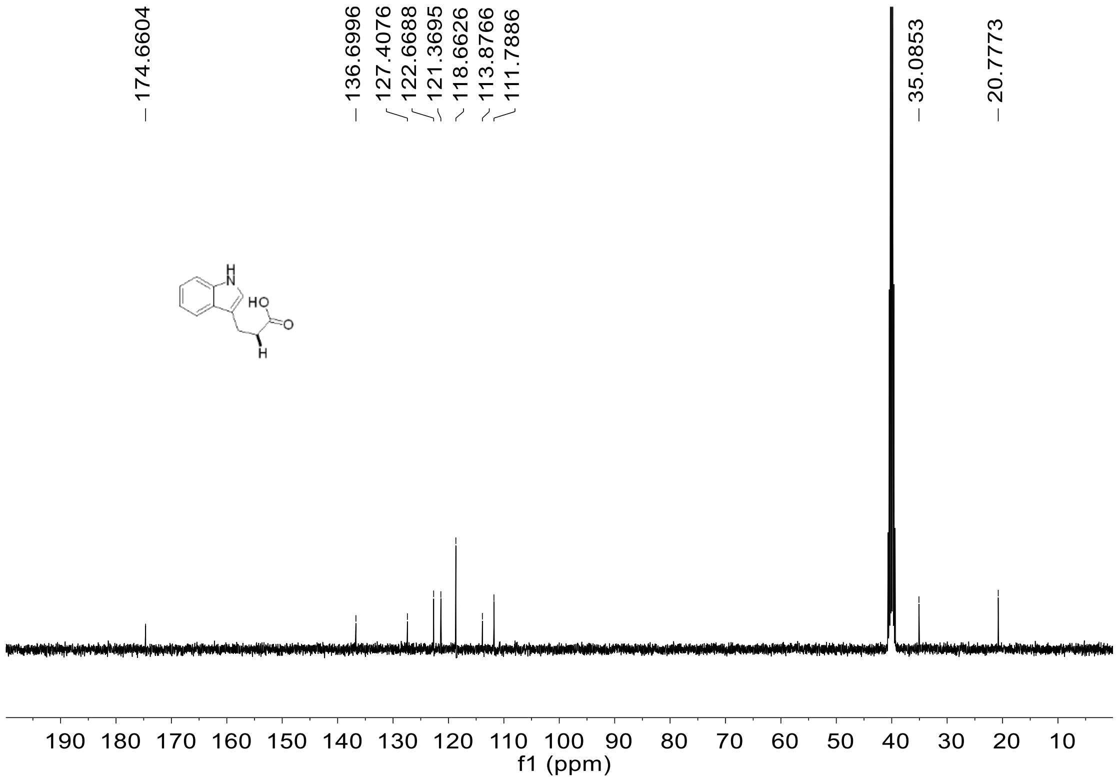

**12c:** $^{1}$H NMR (400 MHz, DMSO-$d_6$)

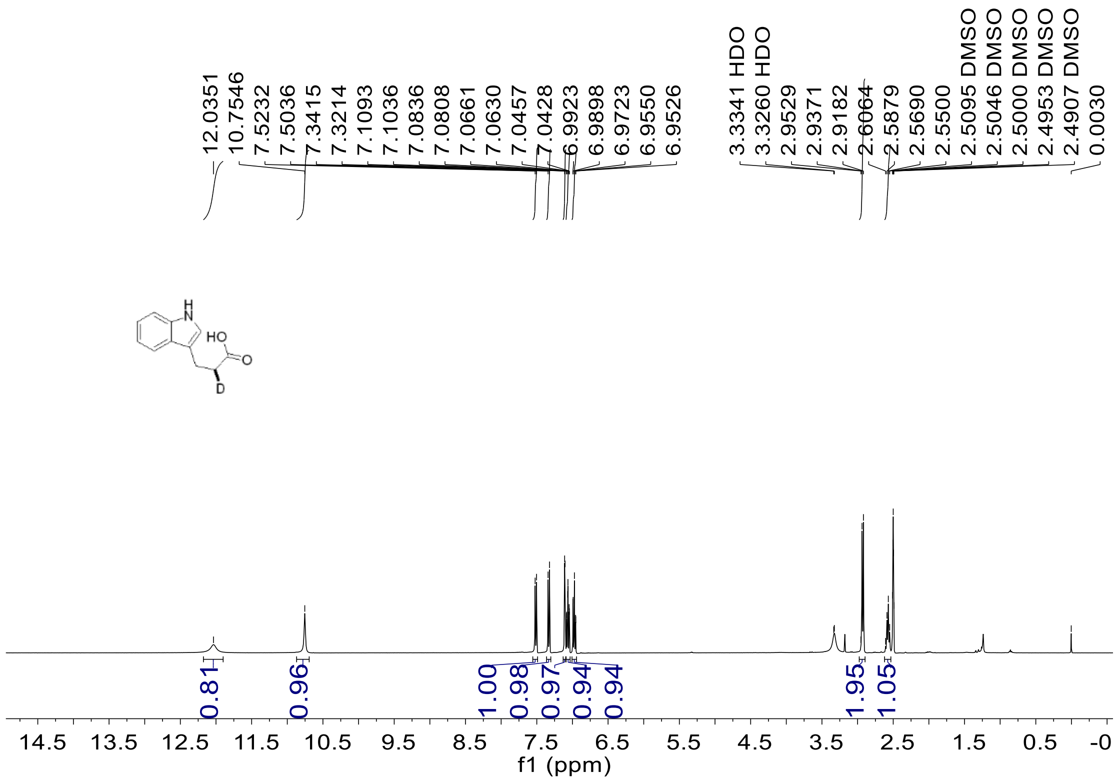


**12c:** $^{13}$C NMR (101 MHz, DMSO-$d_6$)

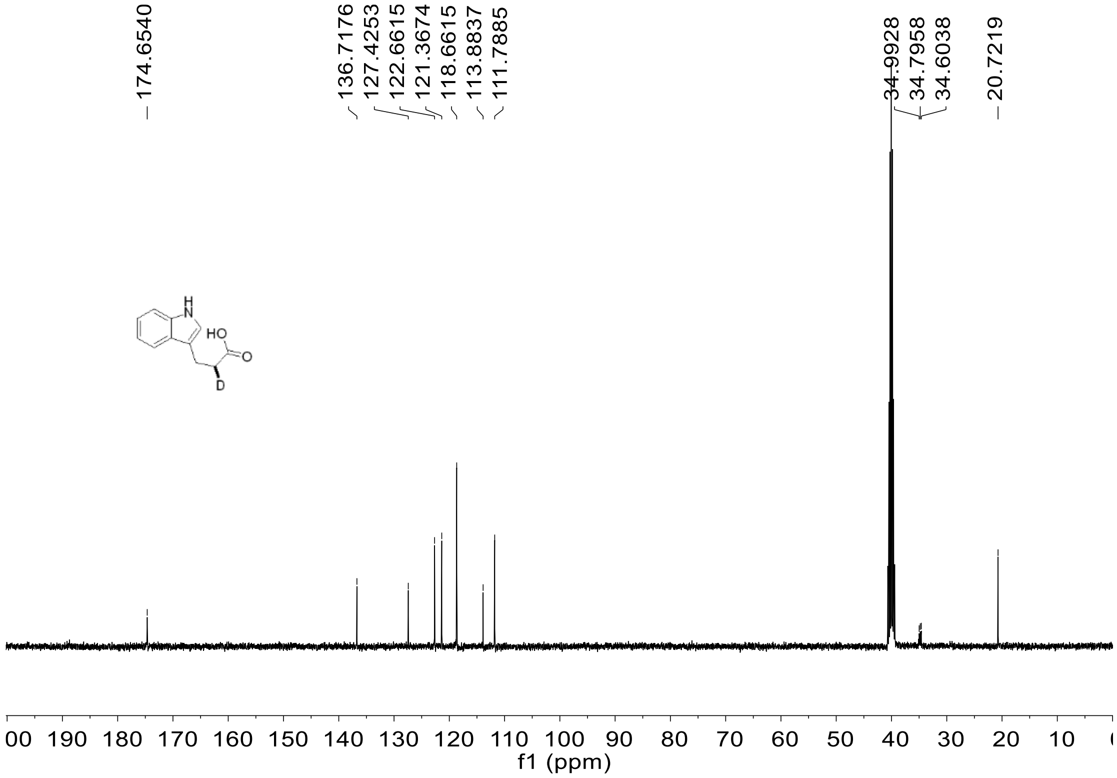

**13b:** $^{1}$H NMR (400 MHz, Chloroform-*d*)

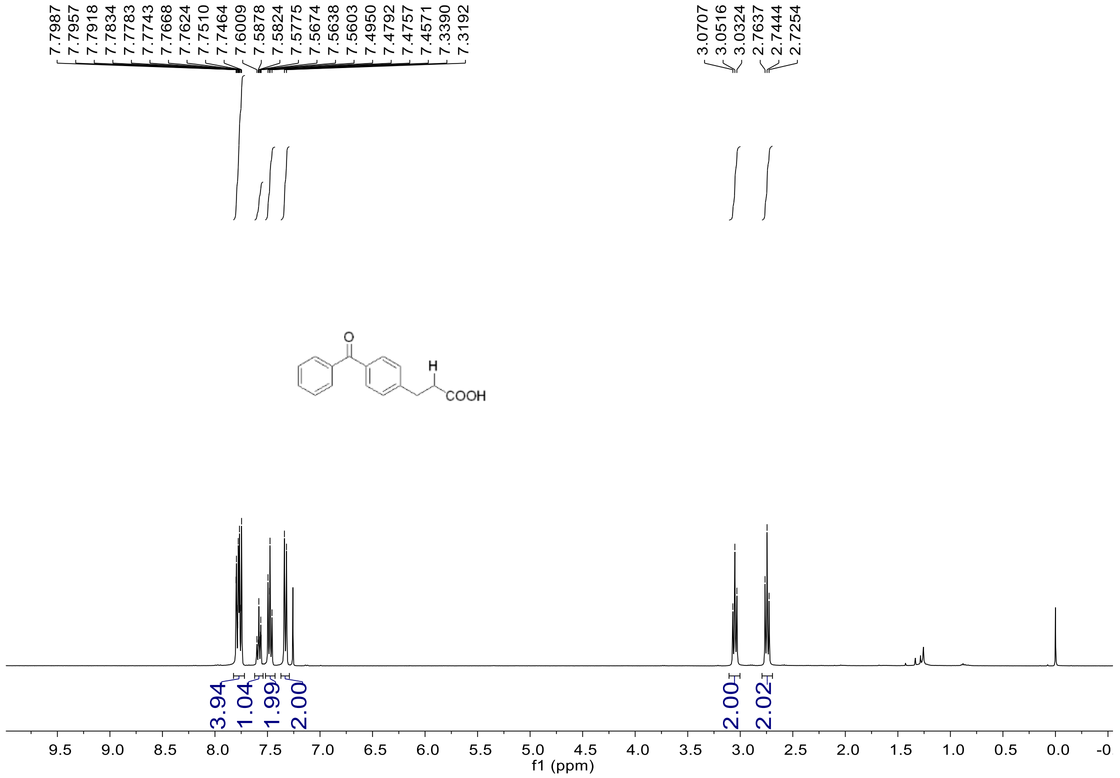

**13b:** $^{13}$C NMR (101 MHz, Chloroform-*d*)

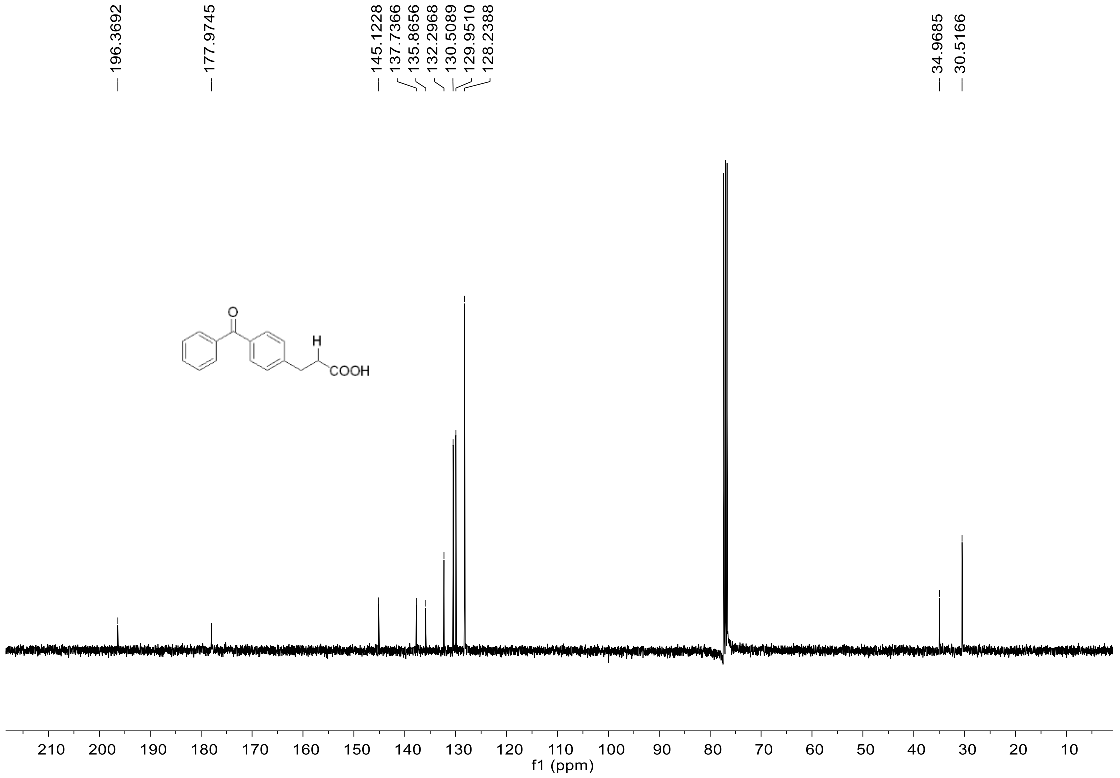

**13c:** $^{1}$H NMR (400 MHz, Chloroform-*d*)

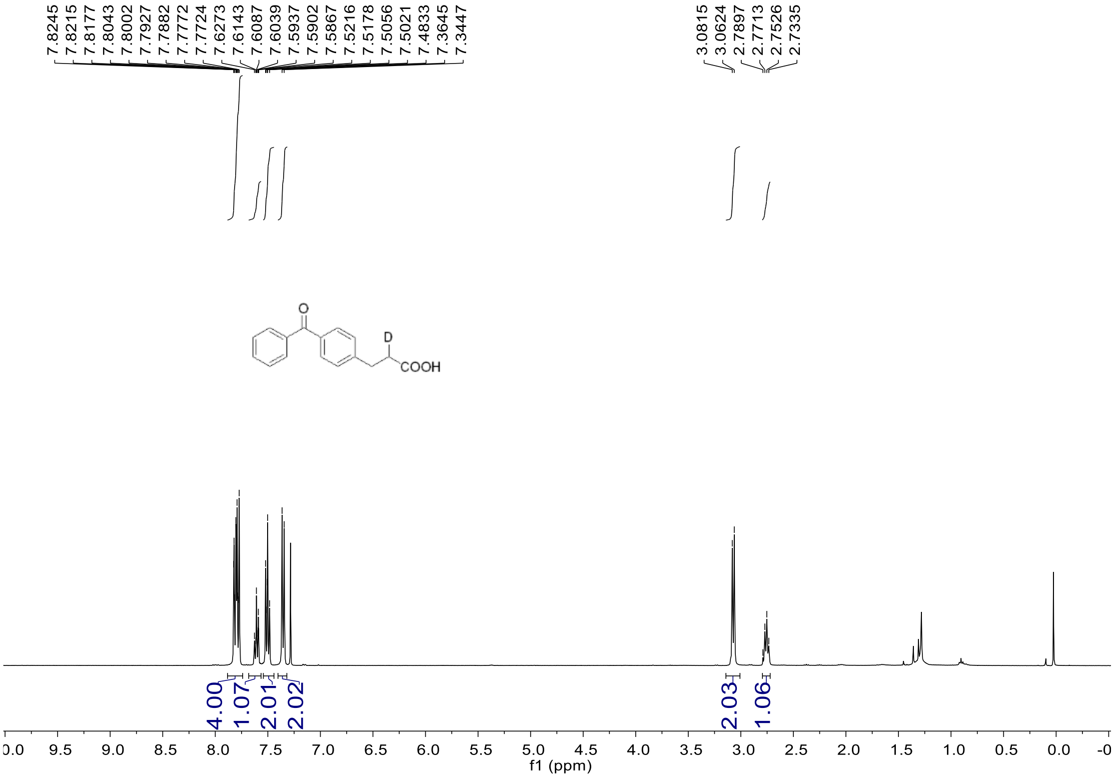


**13c:** $^{13}$C NMR (101 MHz, Chloroform-*d*)

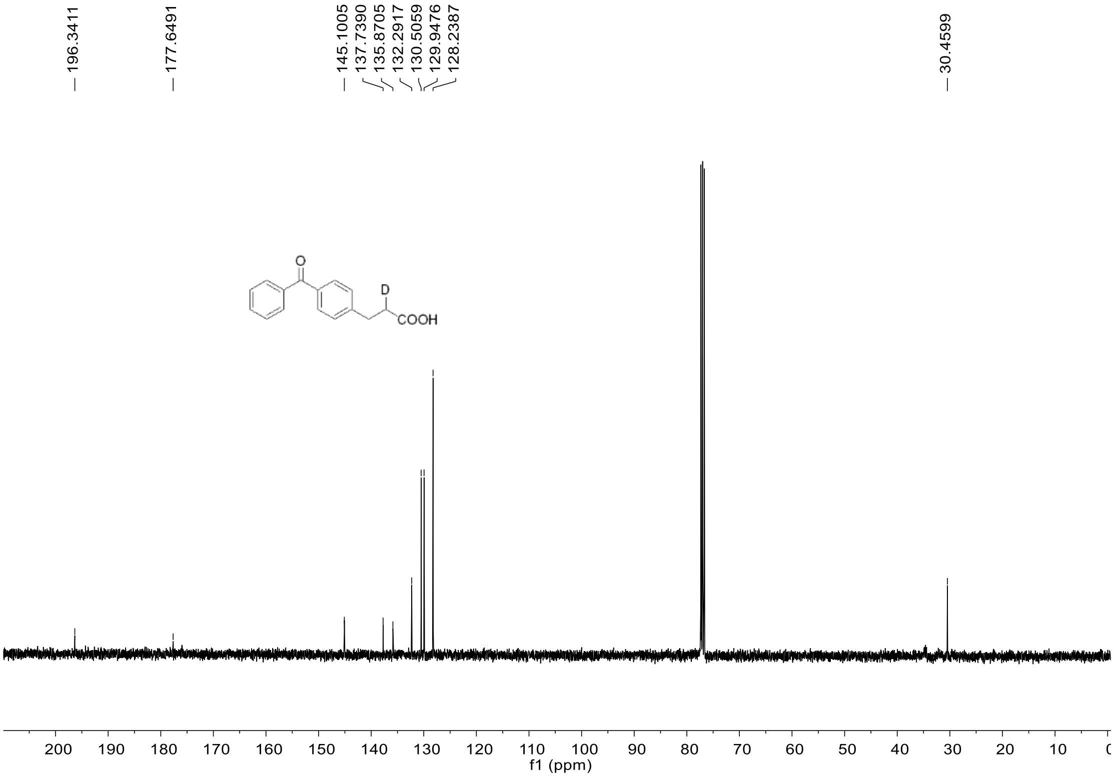

**14b:** $^{1}$H NMR (400 MHz, Chloroform-*d*)

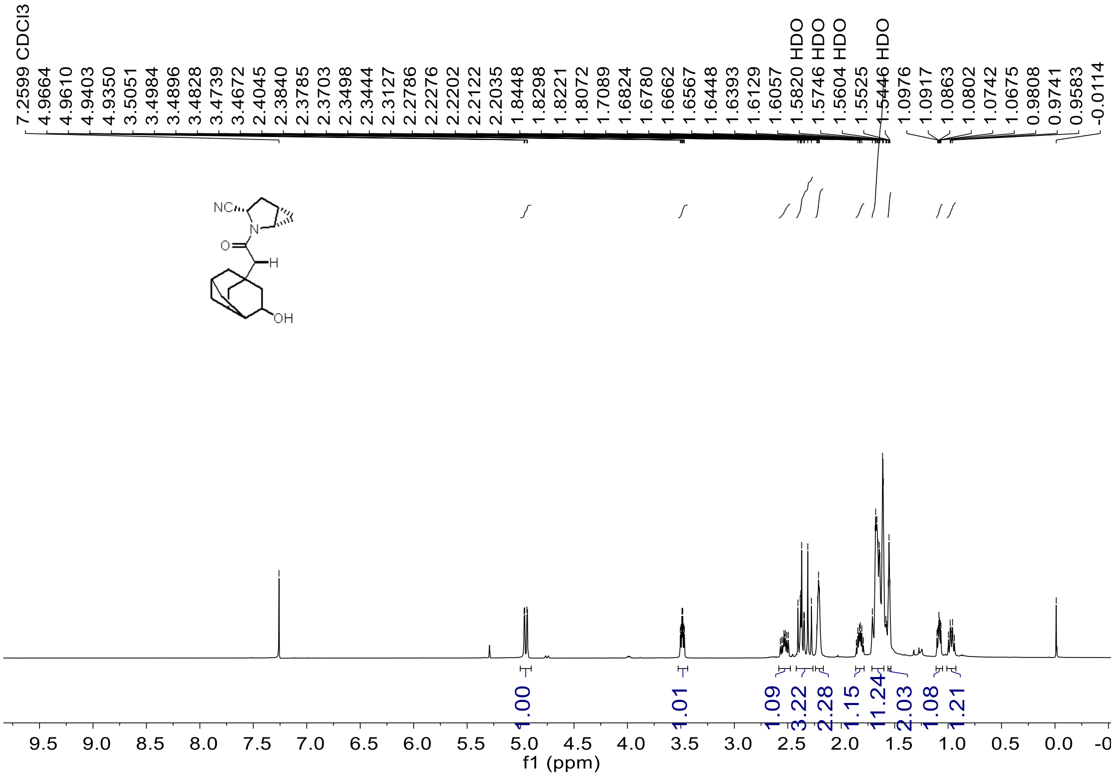


**14b:** $^{13}$C NMR (101 MHz, Chloroform-*d*)

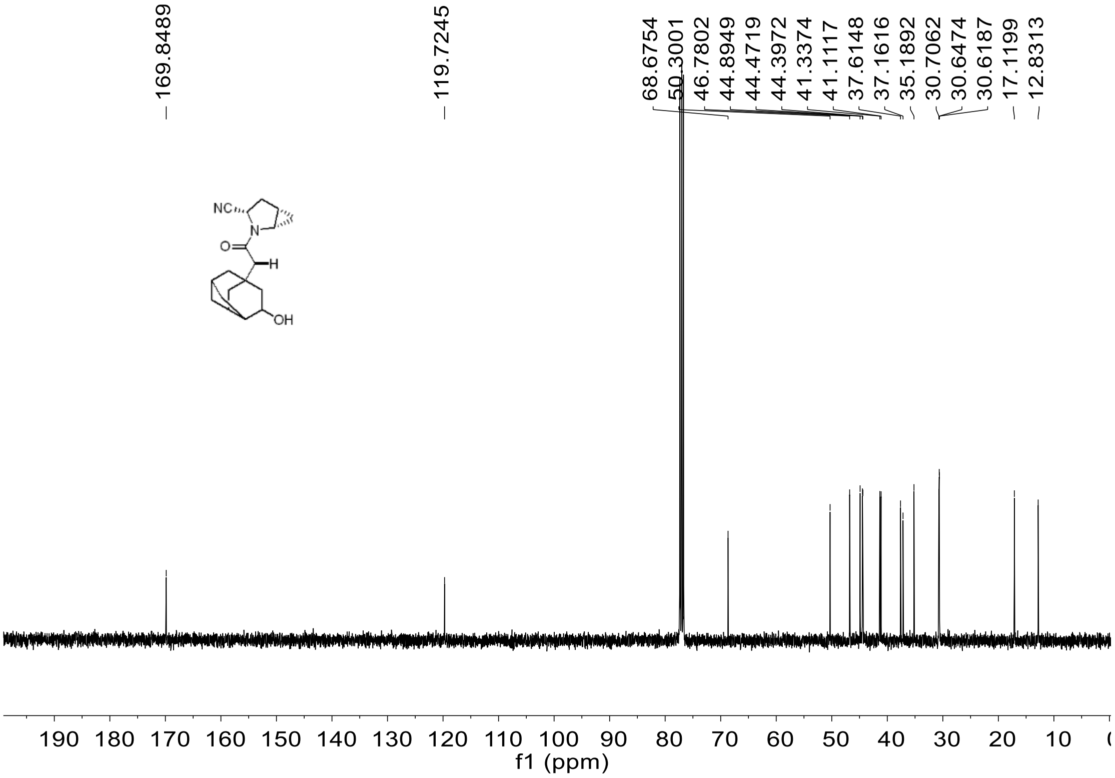

**14c:** $^{1}$H NMR (400 MHz, Chloroform-*d*)

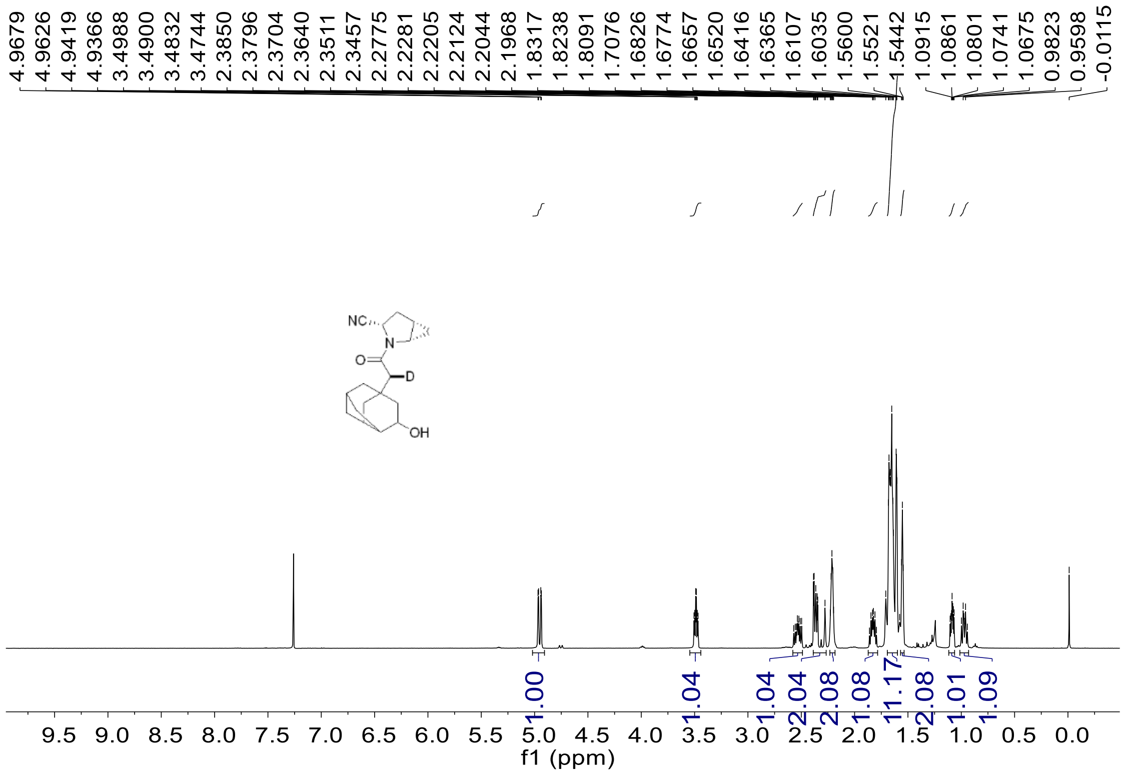


**14c:** $^{13}$C NMR (101 MHz, Chloroform-*d*)

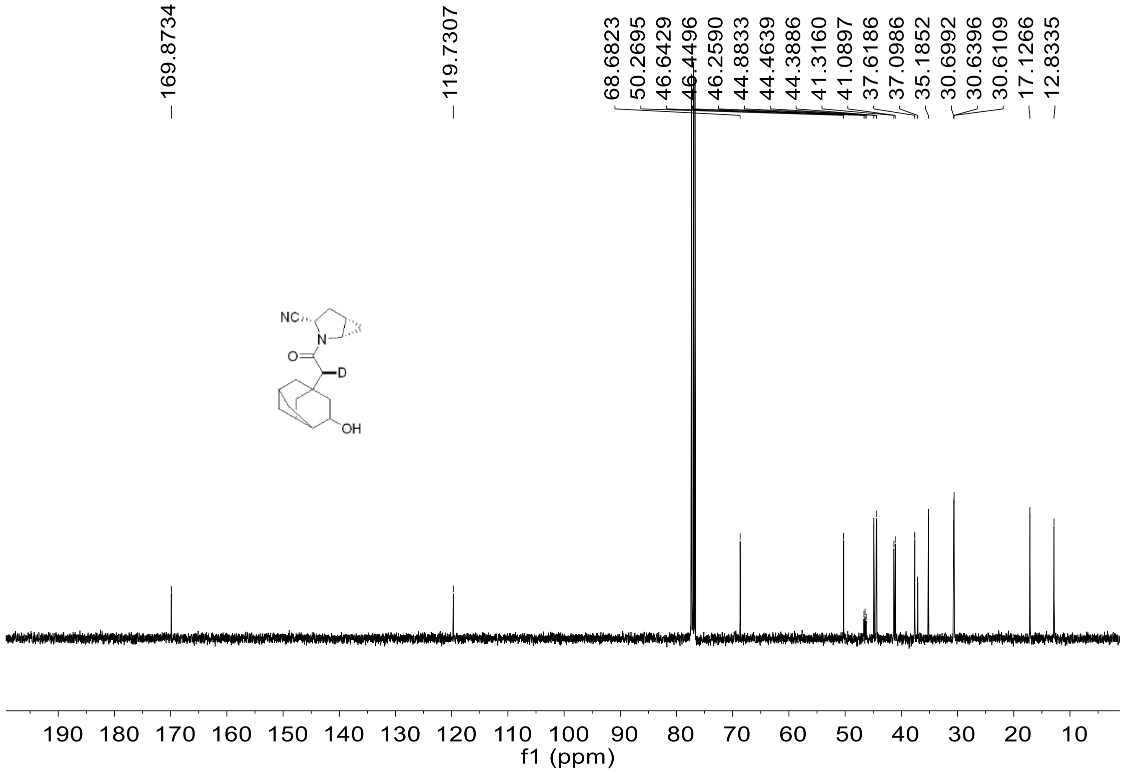

**15b:** $^{1}$H NMR (400 MHz, Chloroform-*d*)

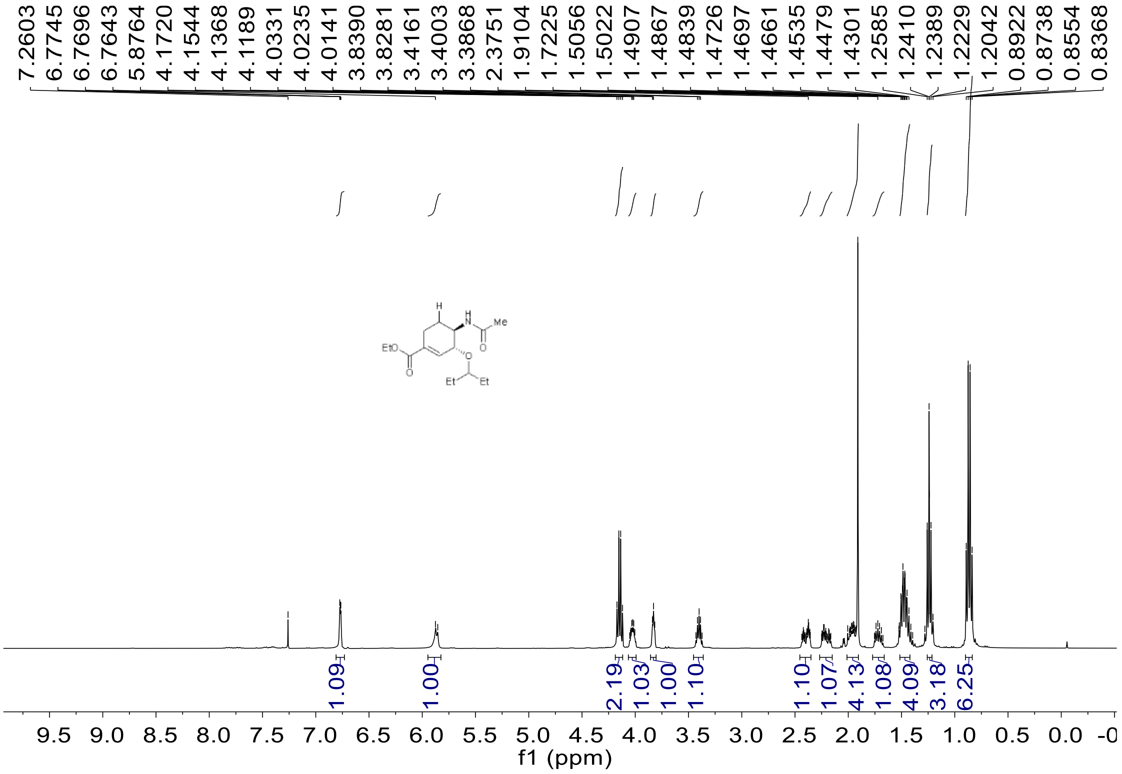


**15b:** $^{13}$C NMR (101 MHz, Chloroform-*d*)

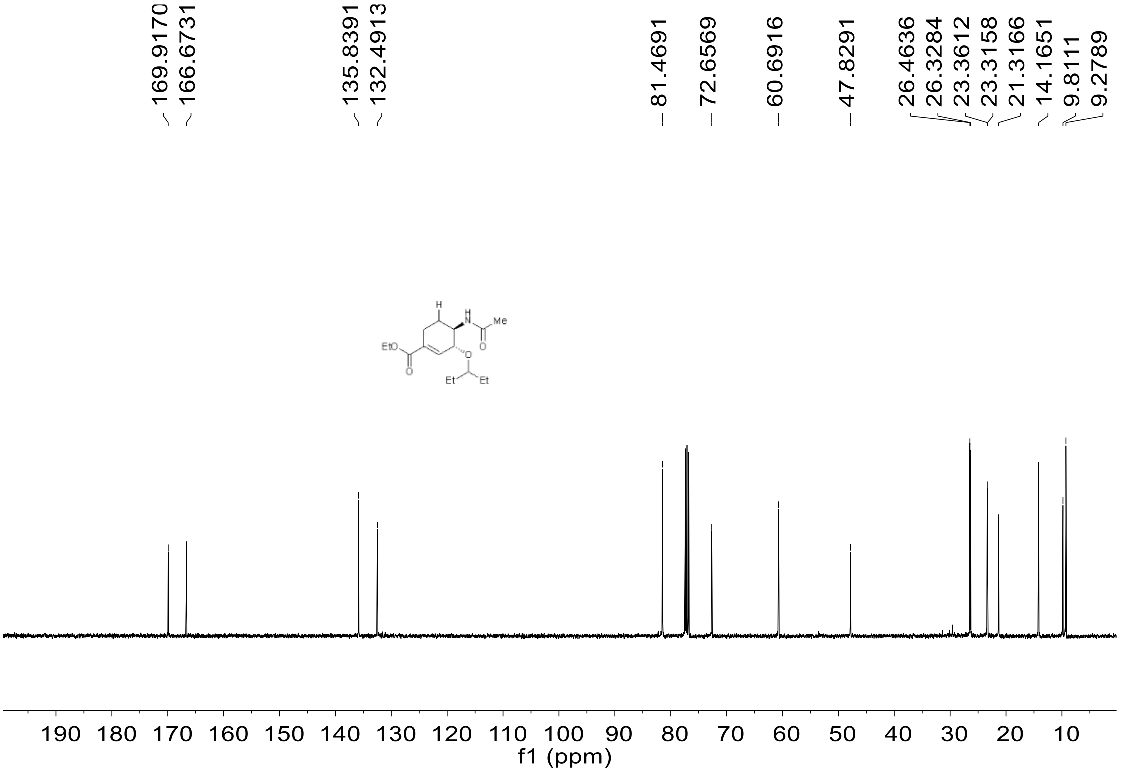

**15c:** $^{1}$H NMR (400 MHz, Chloroform-*d*)

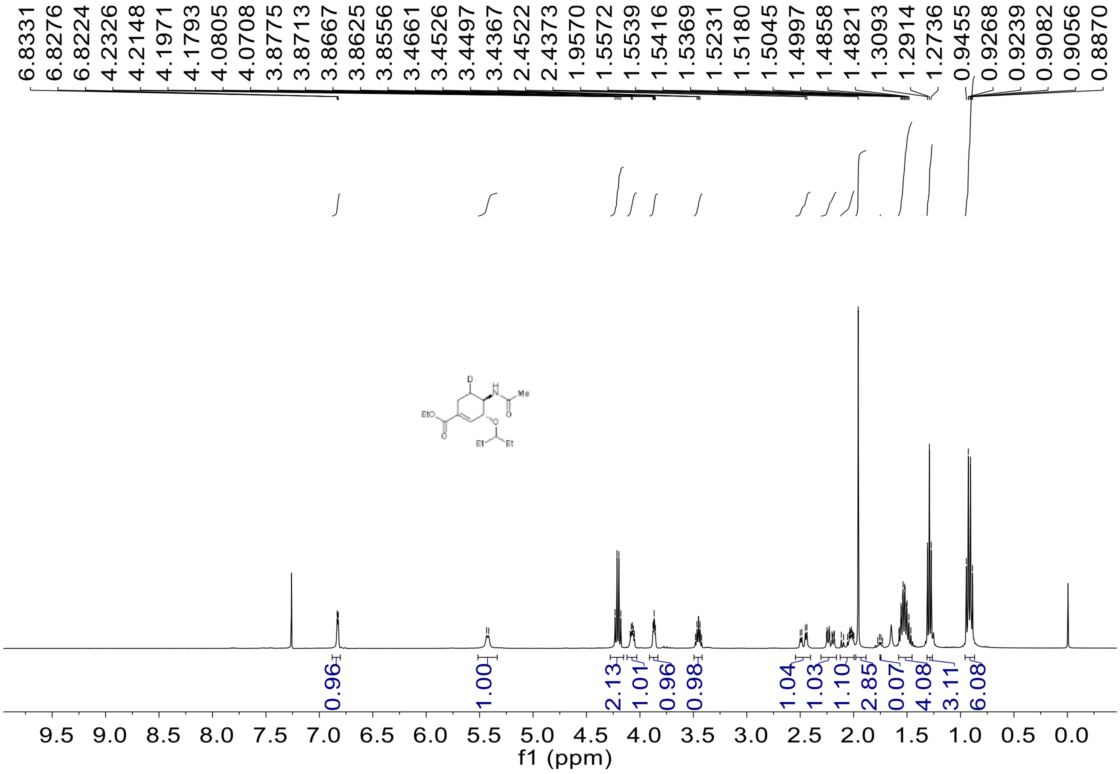

**15c:** $^{13}$C NMR (101 MHz, Chloroform-*d*)

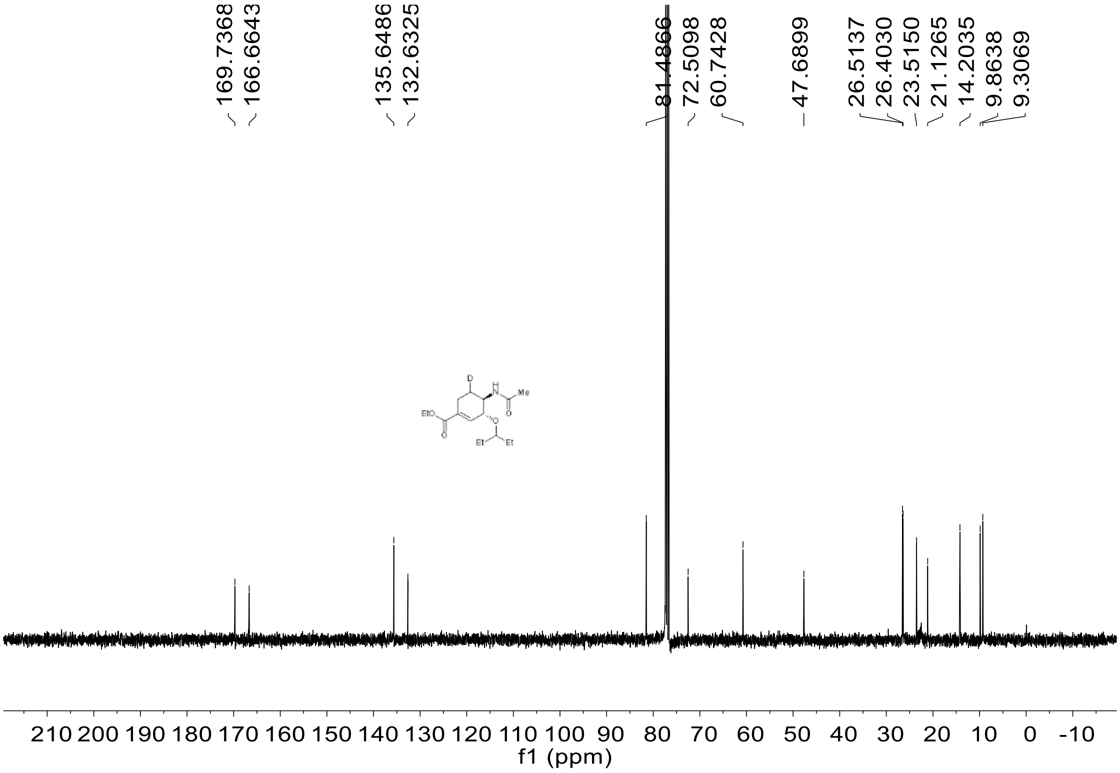

**16b:** $^{1}$H NMR (400 MHz, DMSO-$d_6$)

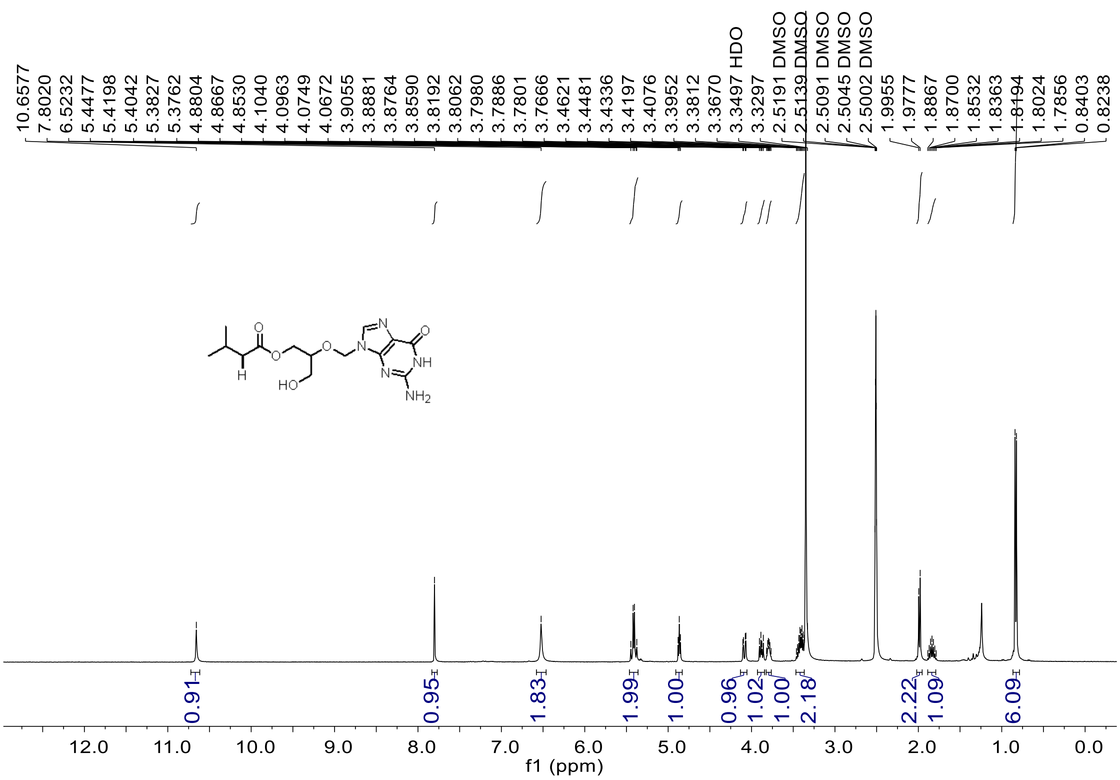


**16b:** $^{13}$C NMR (101 MHz, DMSO-$d_6$)

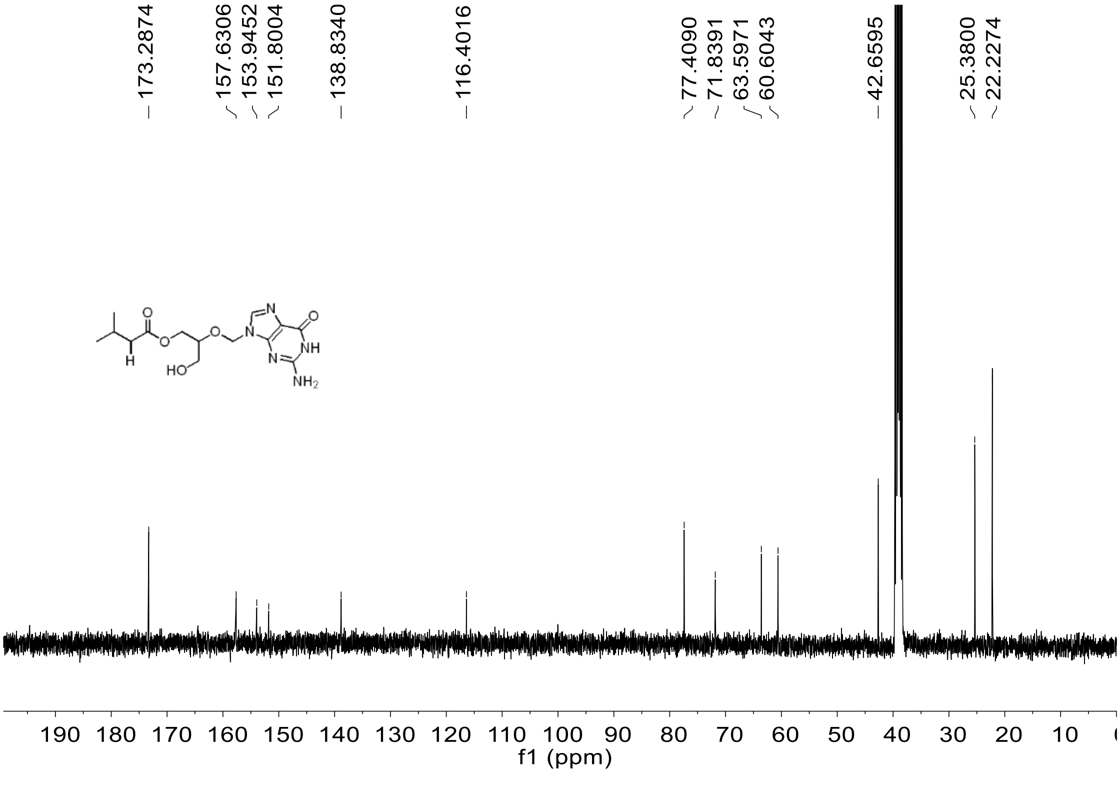

**16c:** $^{1}$H NMR (400 MHz, DMSO-$d_6$)

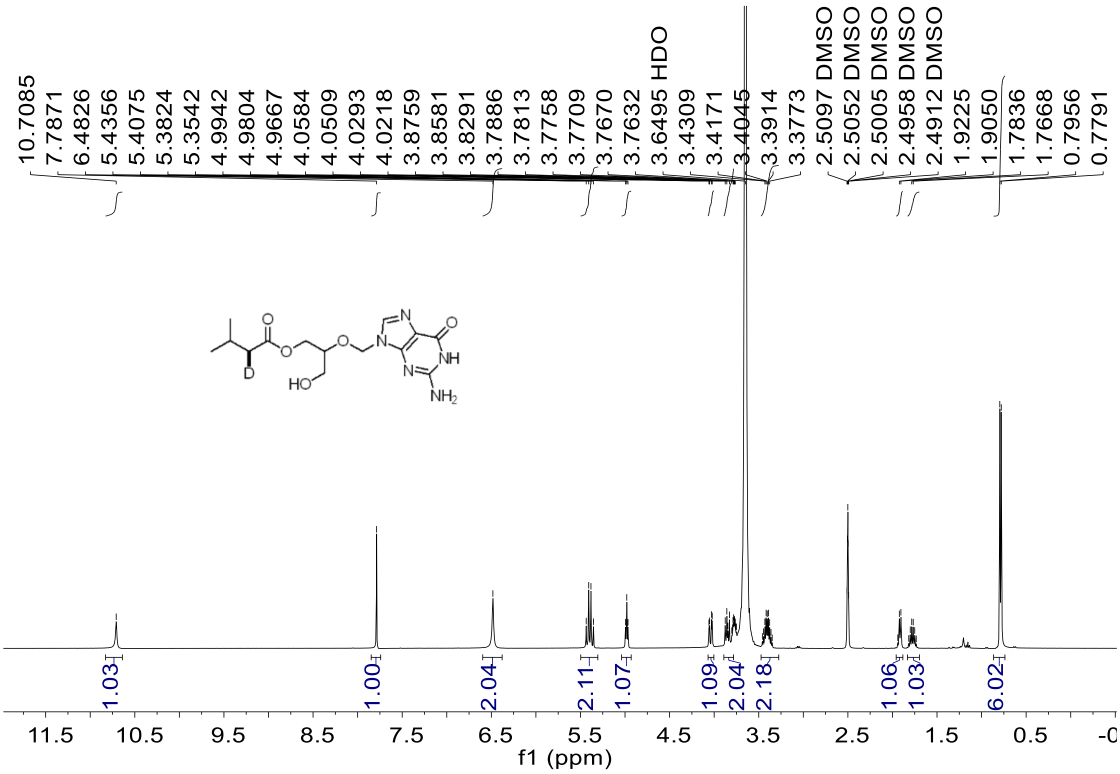


**16c:** $^{13}$C NMR (101 MHz, DMSO-$d_6$)

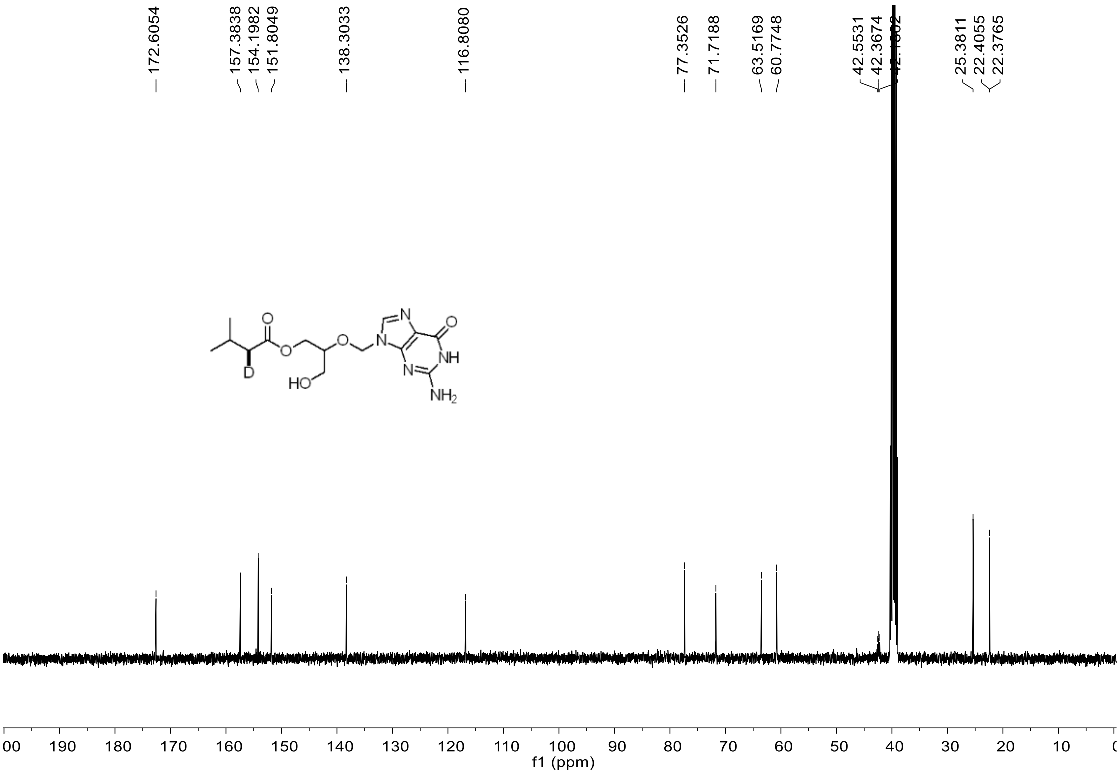

**17b:** $^{1}$H NMR (400 MHz, Chloroform-*d*)

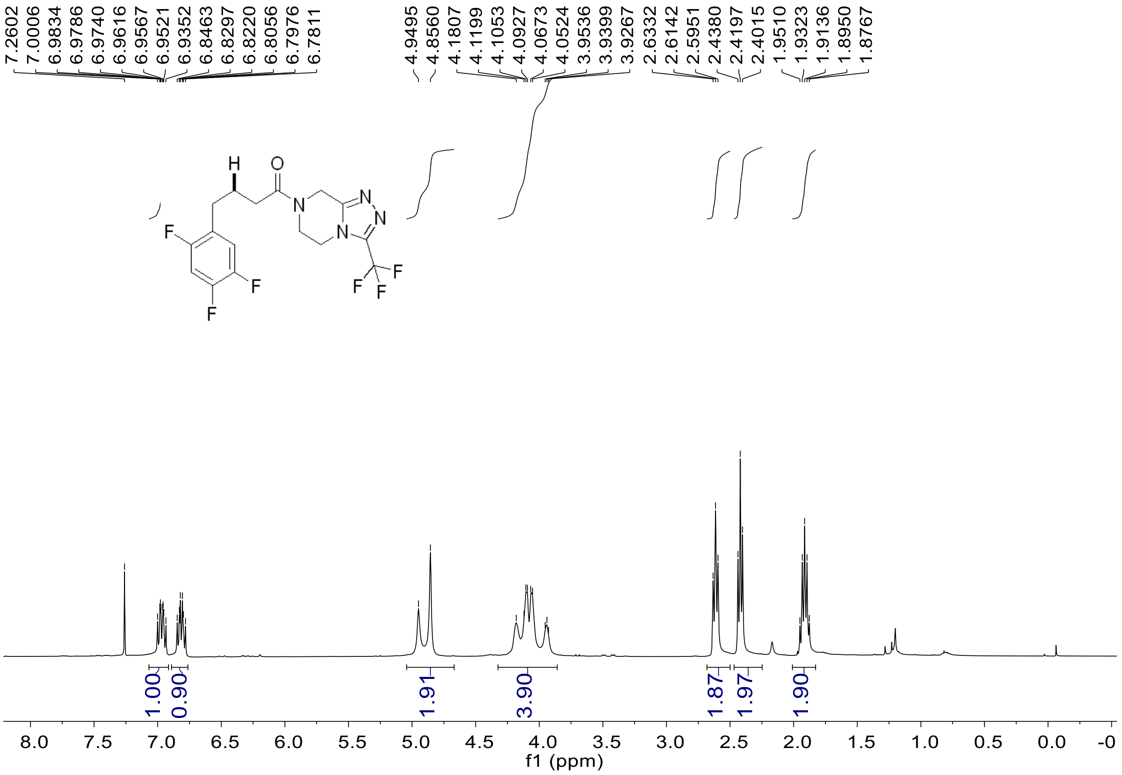


**17b:** $^{13}$C NMR (101 MHz, Chloroform-*d*)

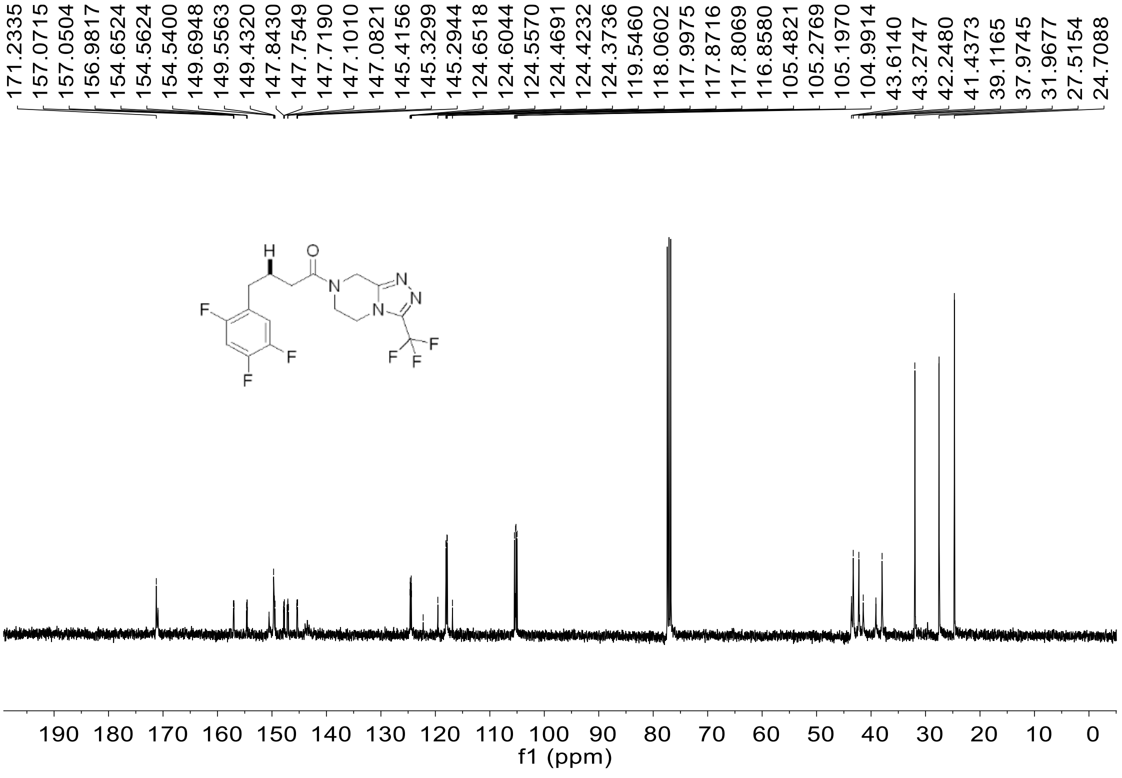

**17b:** $^{19}$F NMR (376 MHz, Chloroform-$d$)

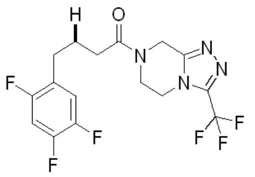

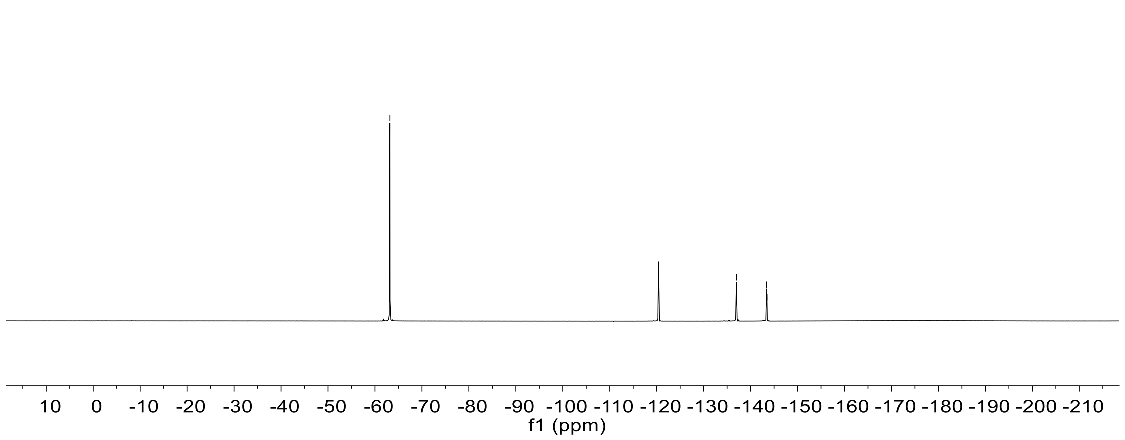

**17c:** $^{1}$H NMR (400 MHz, Chloroform-*d*)

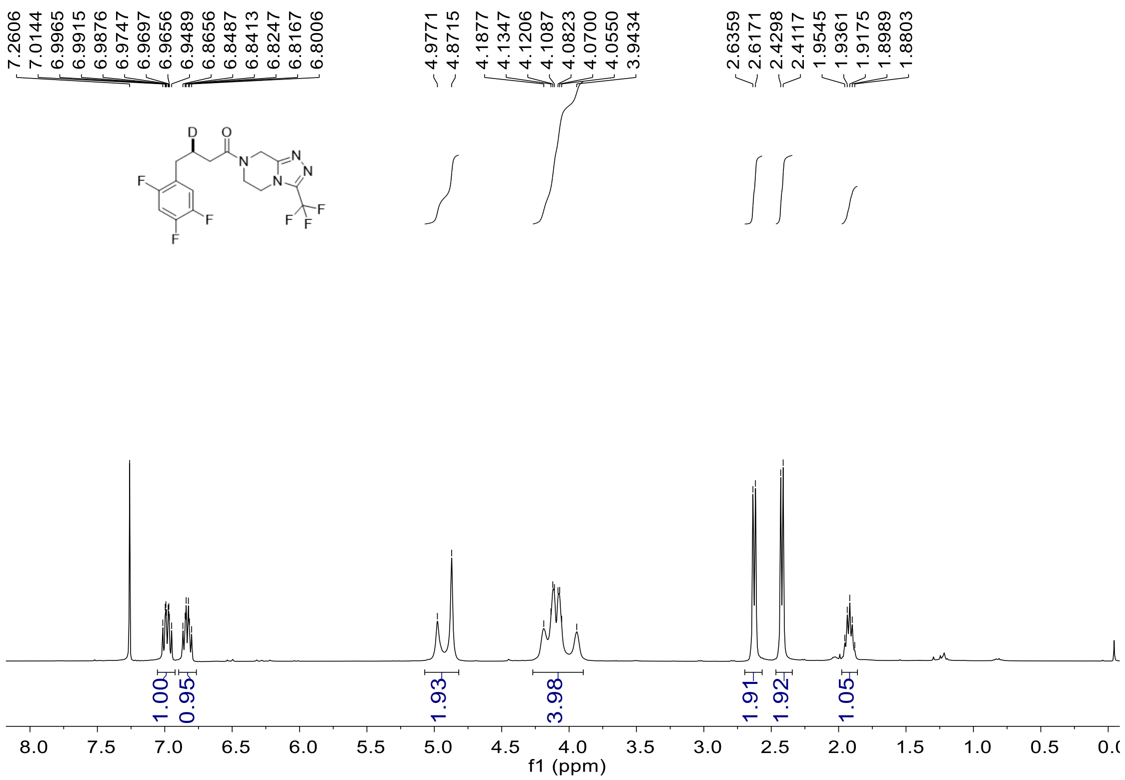


**17c:** $^{13}$C NMR (101 MHz, Chloroform-*d*)

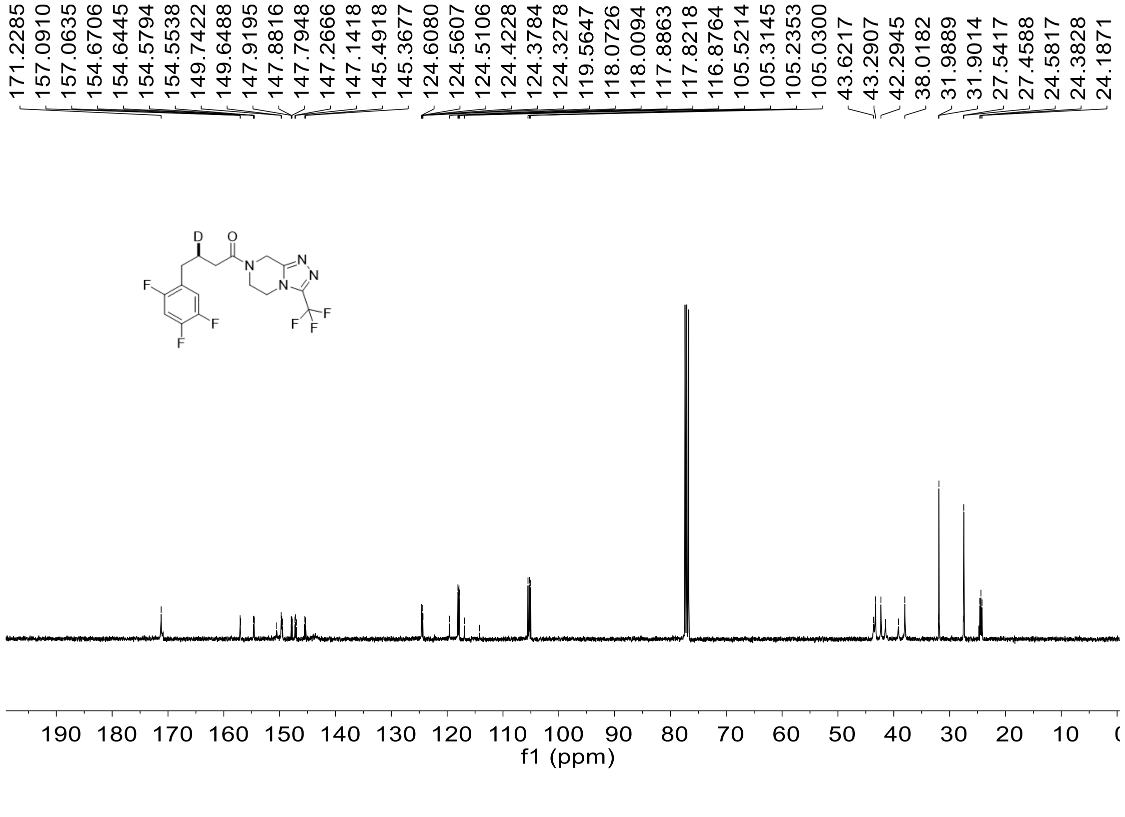

**17c:** $^{19}$F NMR (376 MHz, Chloroform-$d$)

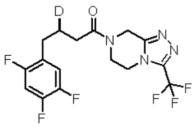

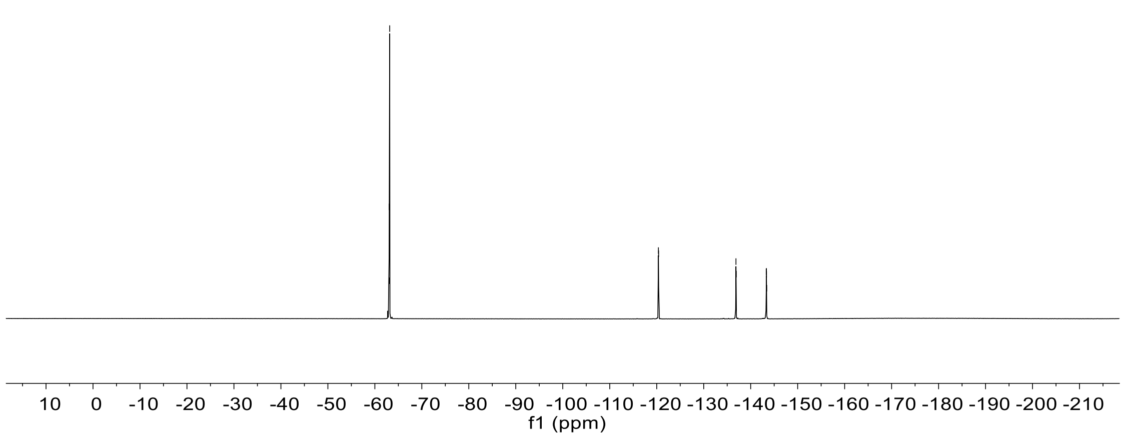

**18b:** $^{1}$H NMR (400 MHz, Chloroform-*d*)

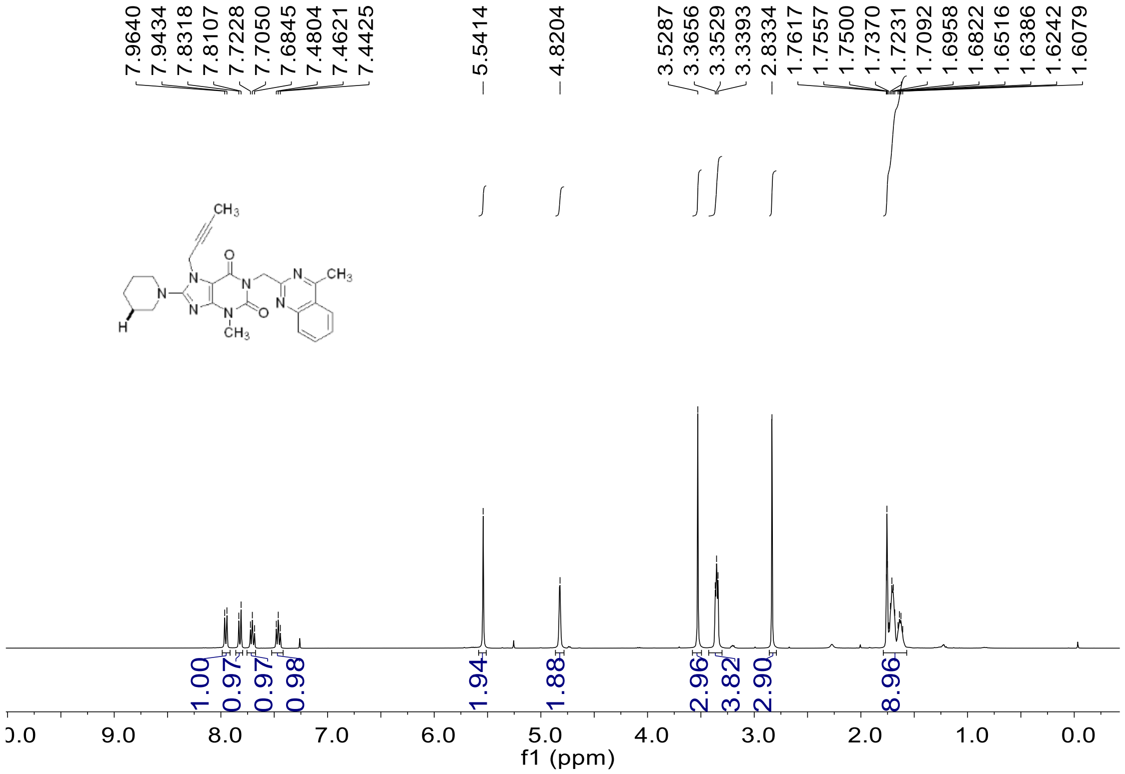


**18b:** $^{13}$C NMR (101 MHz, Chloroform-*d*)

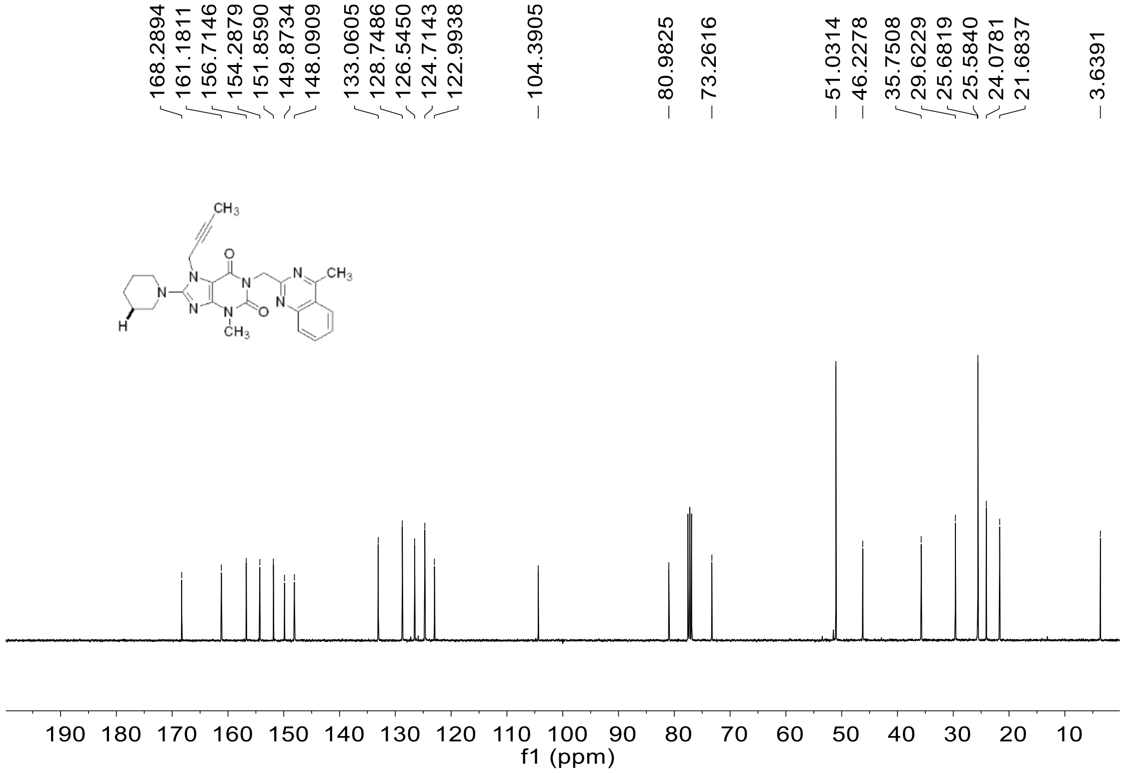

**18c:** $^{1}$H NMR (400 MHz, Chloroform-*d*)

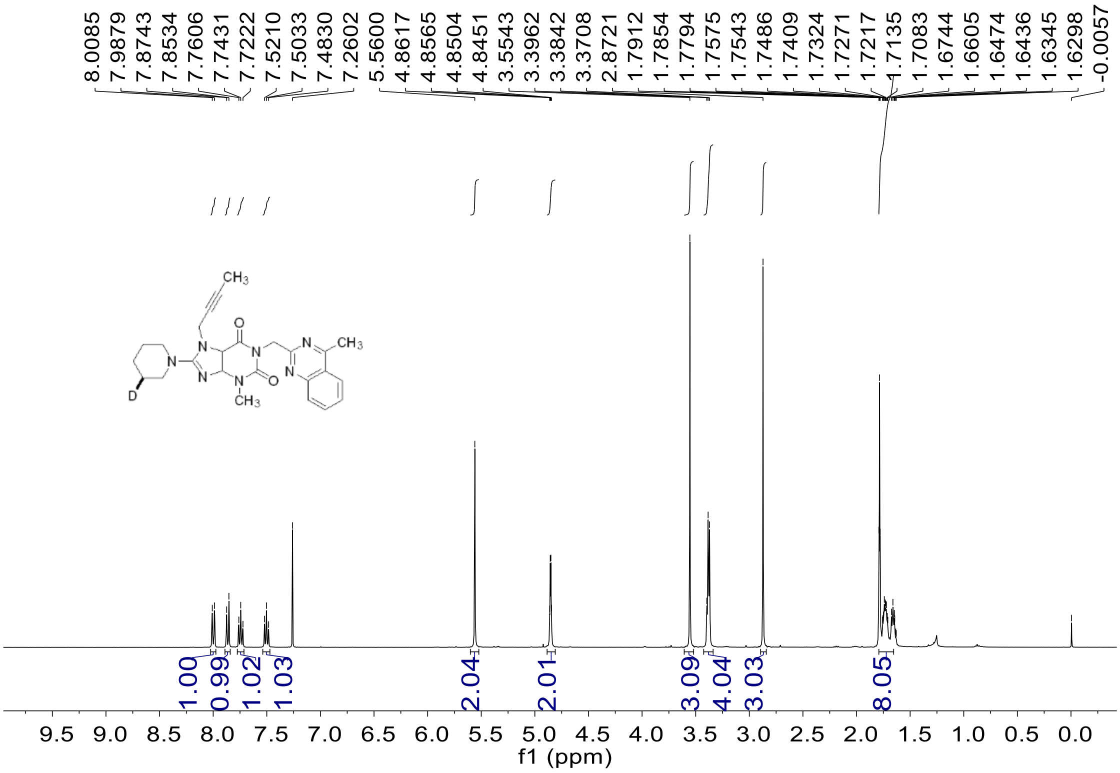


**18c:** $^{13}$C NMR (101 MHz, Chloroform-*d*)

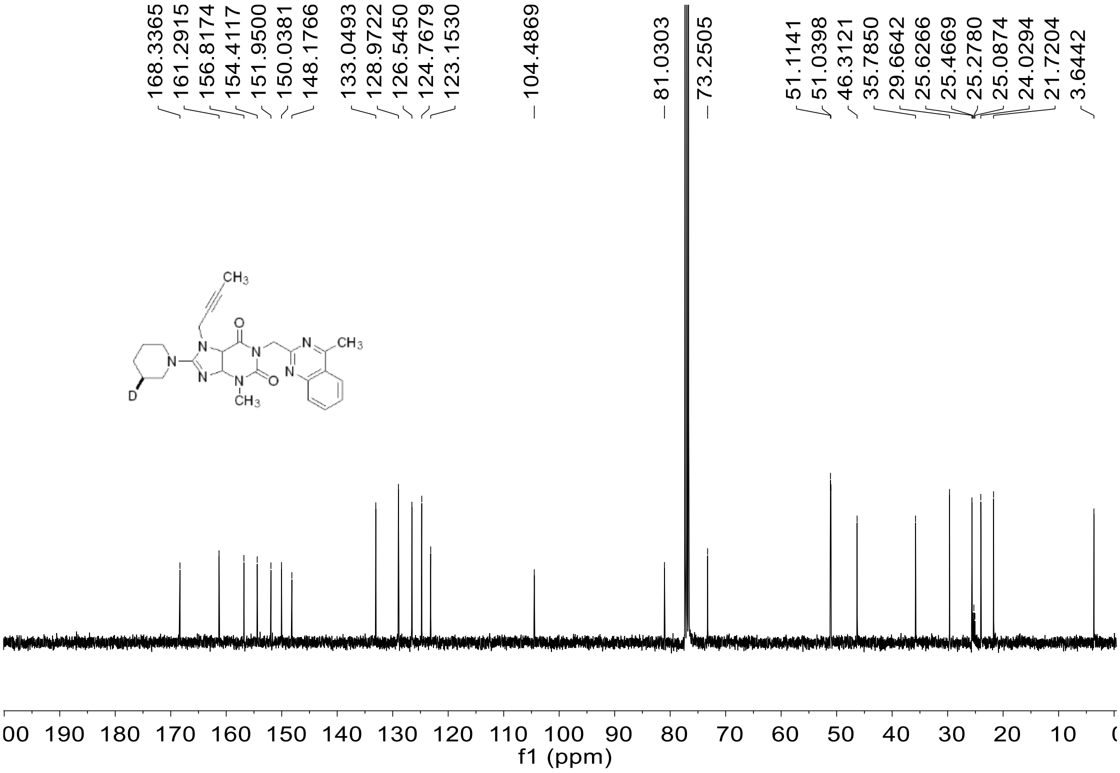

**19b:** $^{1}$H NMR (400 MHz, Chloroform-*d*)

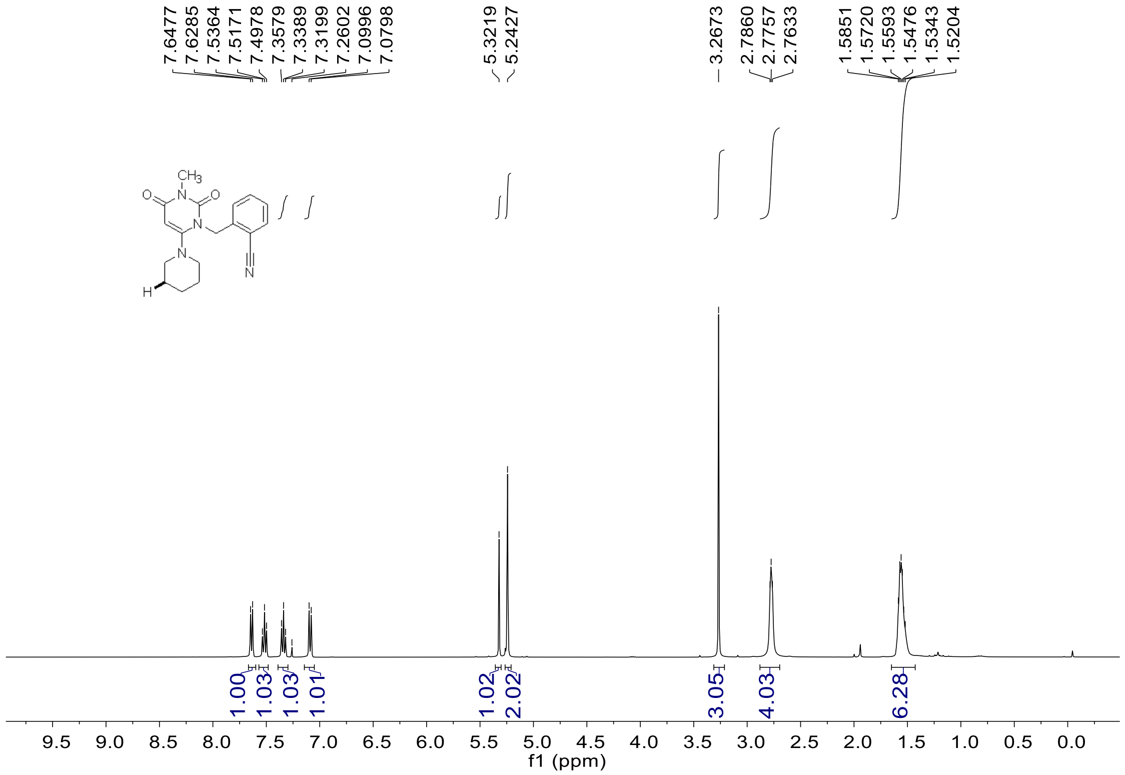


**19b:** $^{13}$C NMR (101 MHz, Chloroform-*d*)

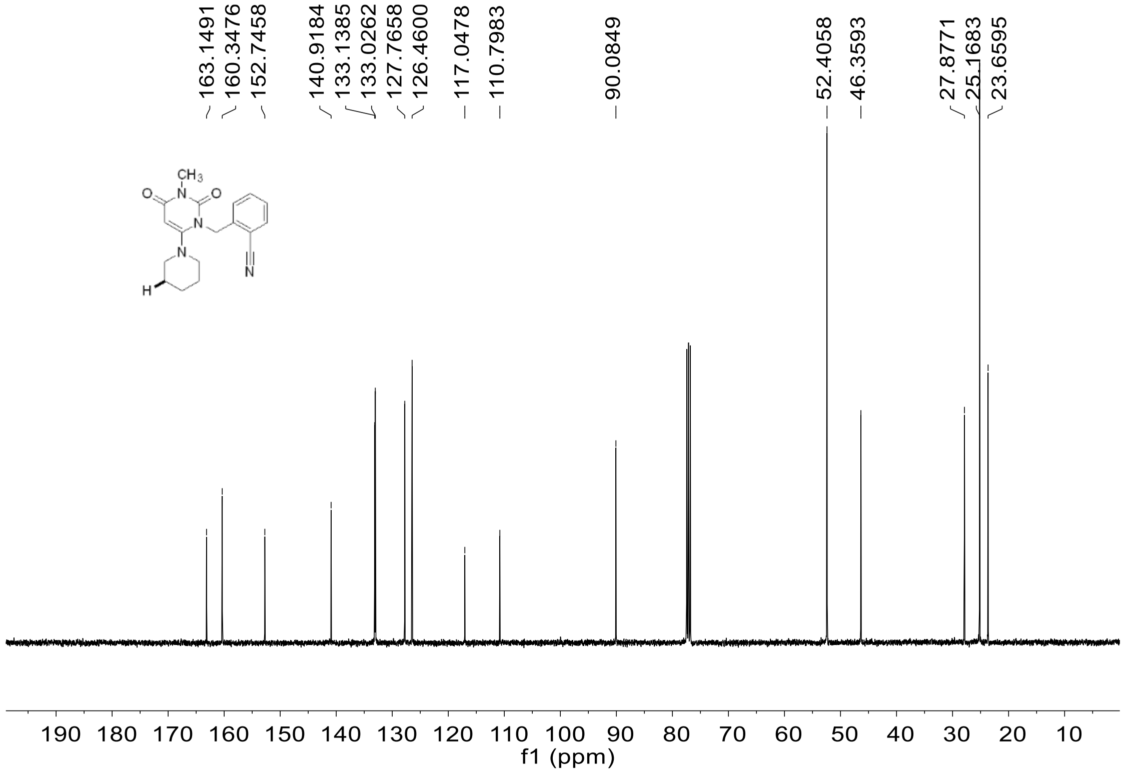

**19c:** $^{1}$H NMR (400 MHz, Chloroform-*d*)

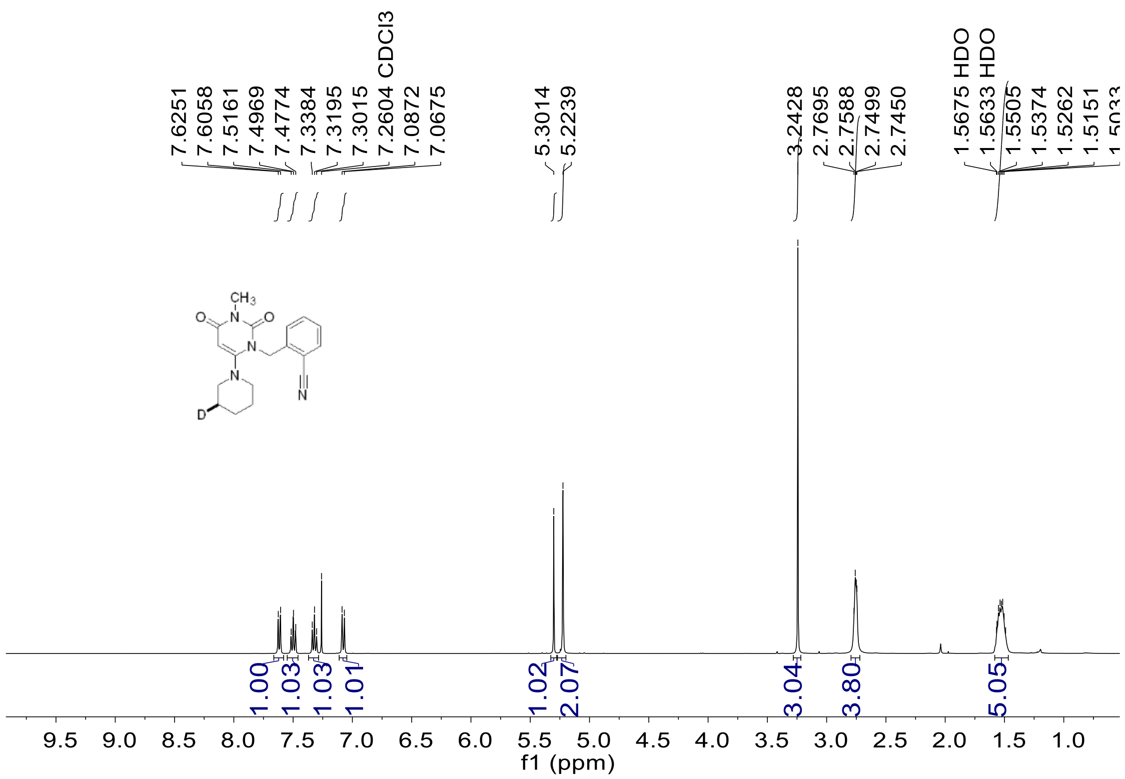

**19c:** $^{13}$C NMR (101 MHz, Chloroform-*d*)

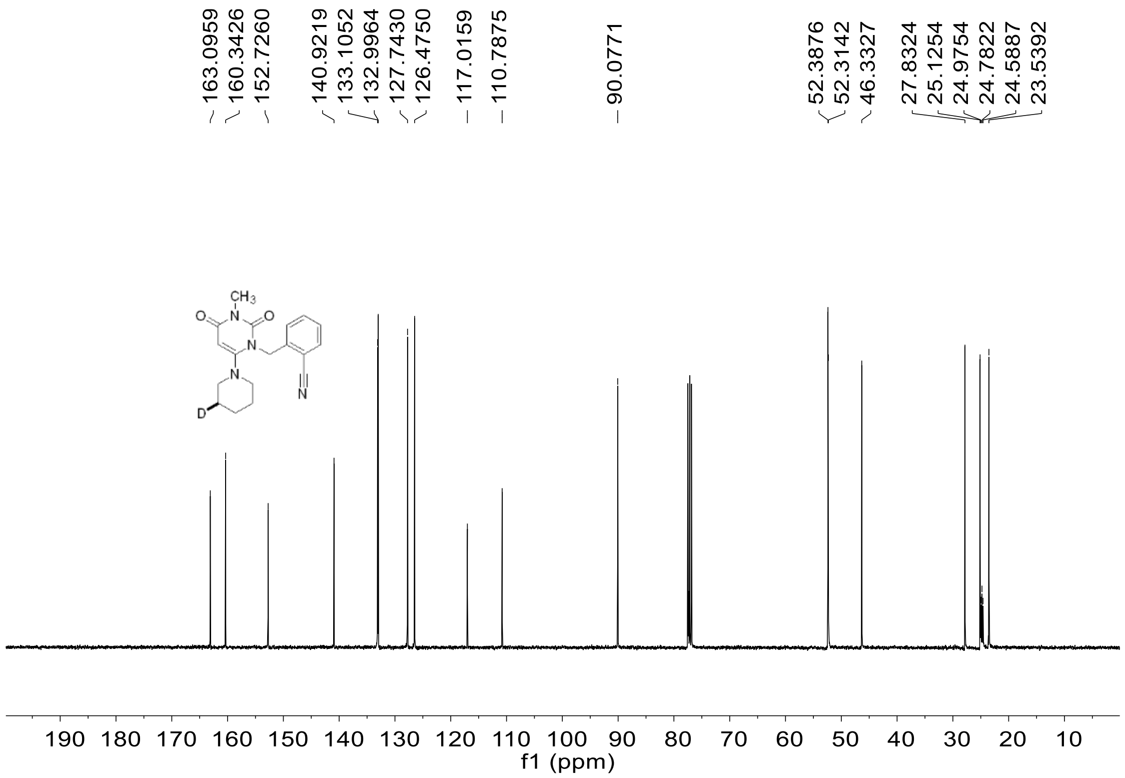

**20b:** $^{1}$H NMR (400 MHz, Chloroform-*d*)

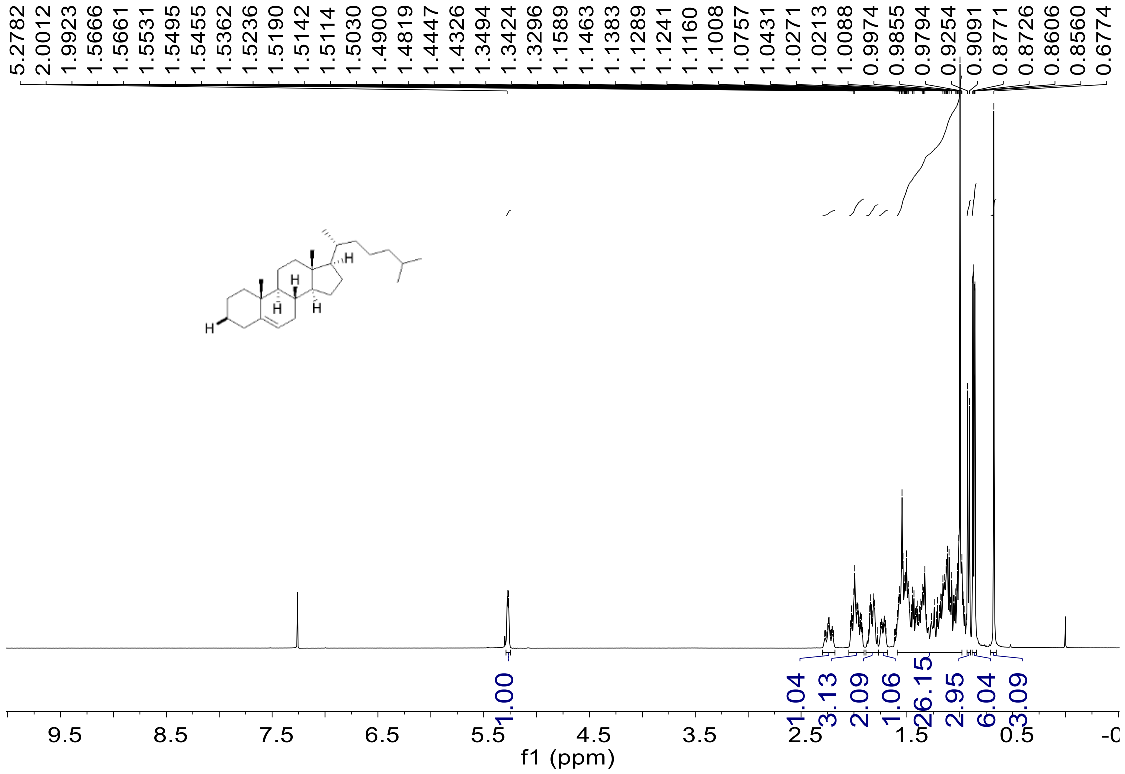


**20b:** $^{13}$C NMR (101 MHz, Chloroform-*d*)

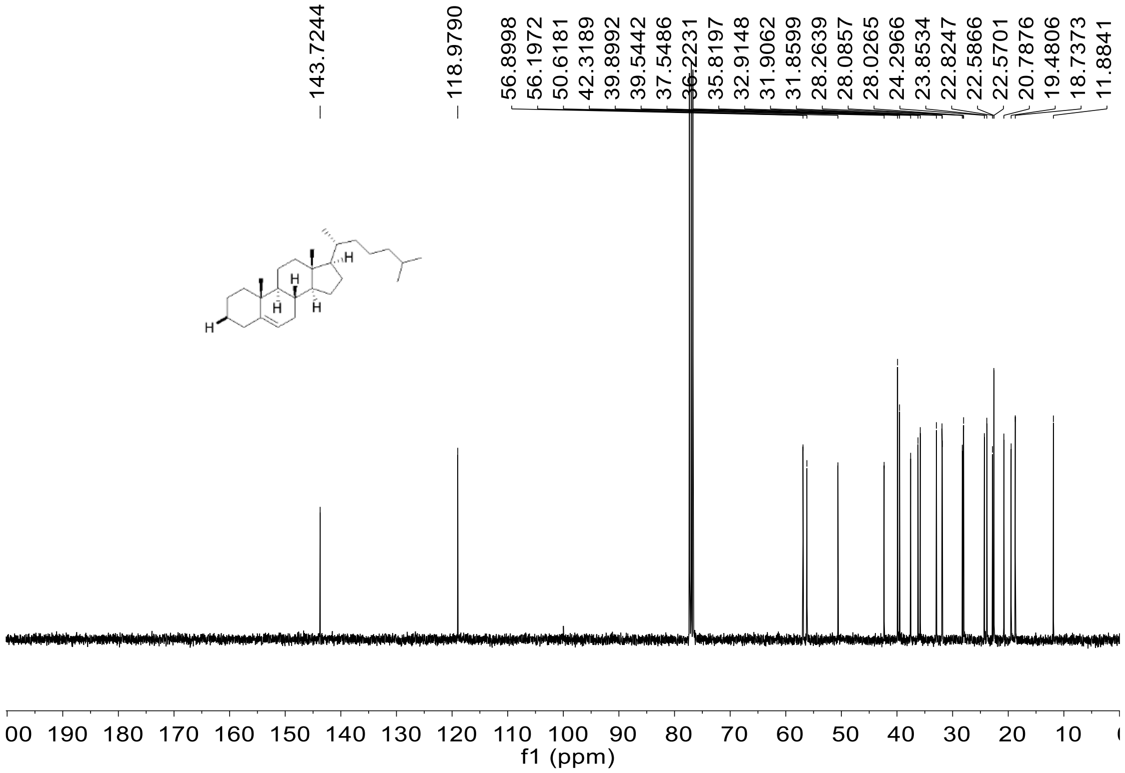

**20c:** $^{1}$H NMR (400 MHz, Chloroform-*d*)

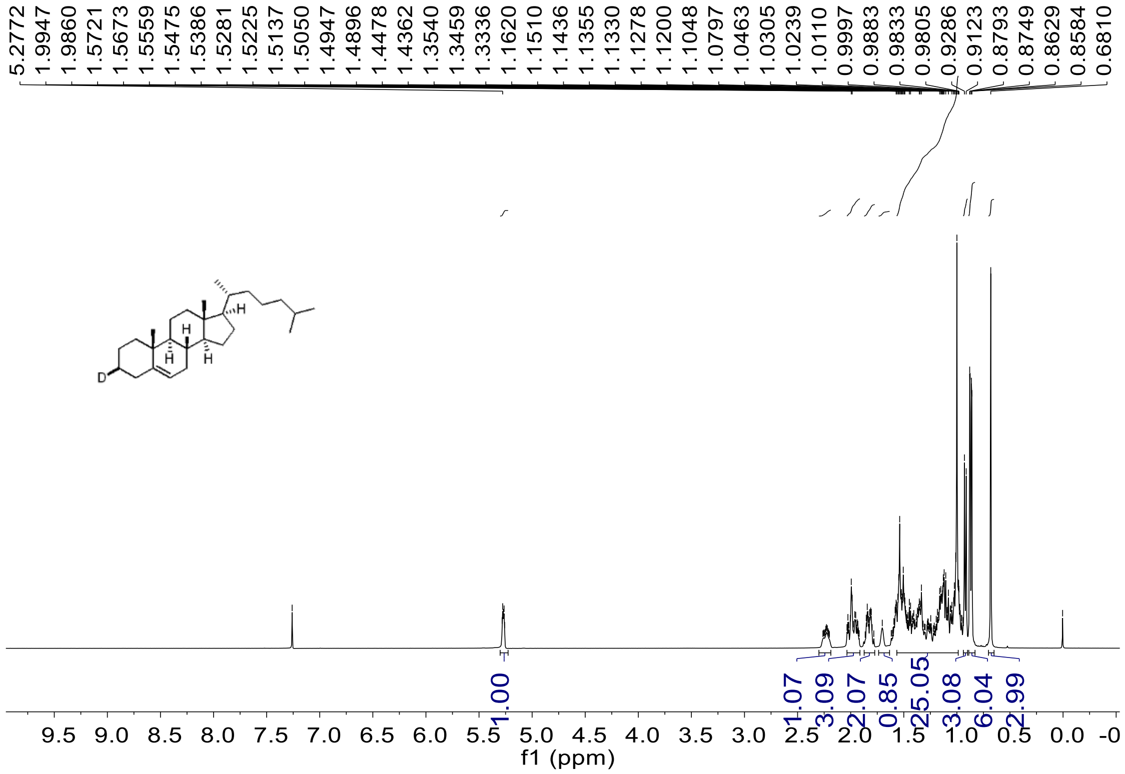


**20c:** $^{13}$C NMR (101 MHz, Chloroform-*d*)

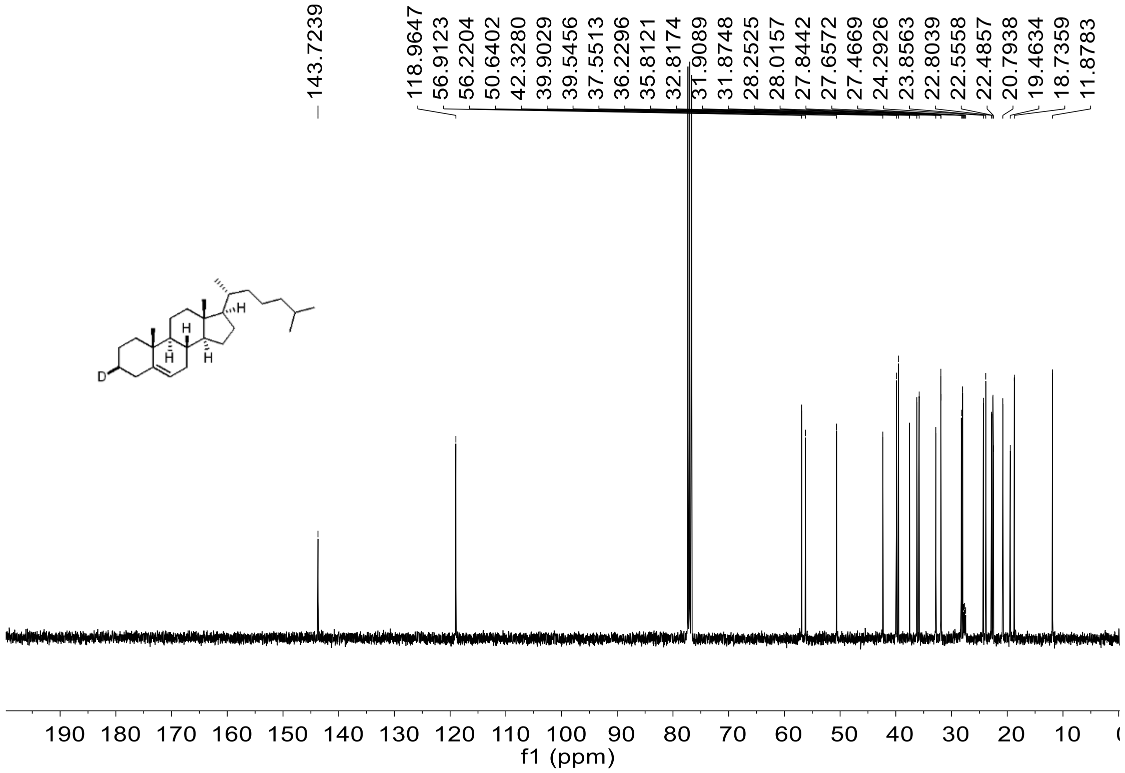

**21b:** $^{1}$H NMR (400 MHz, Chloroform-*d*)

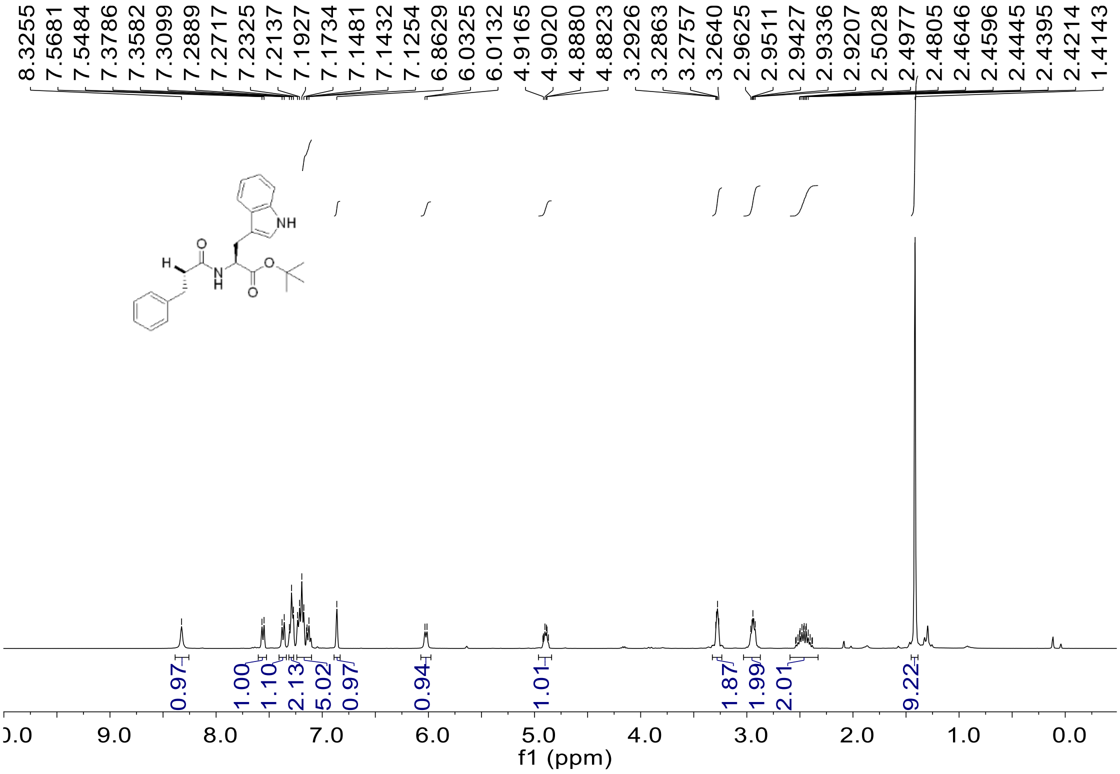


**21b:** $^{13}$C NMR (101 MHz, Chloroform-*d*)

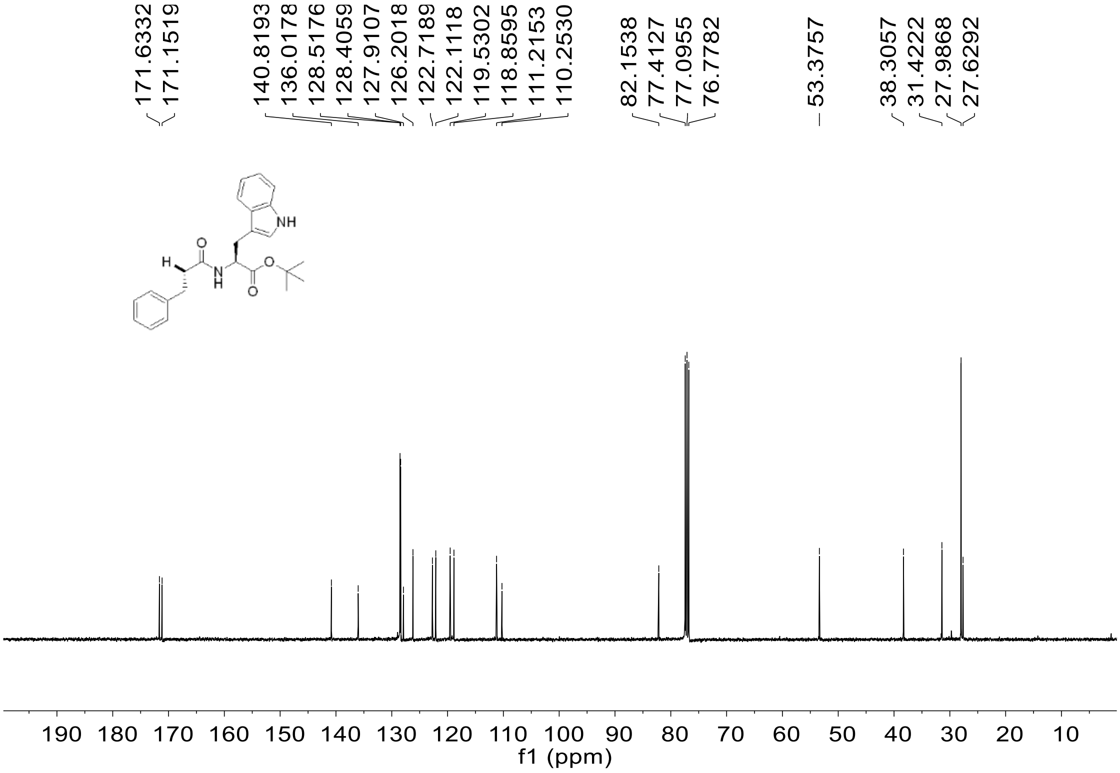

**21c:** $^{1}$H NMR (400 MHz, Chloroform-*d*)

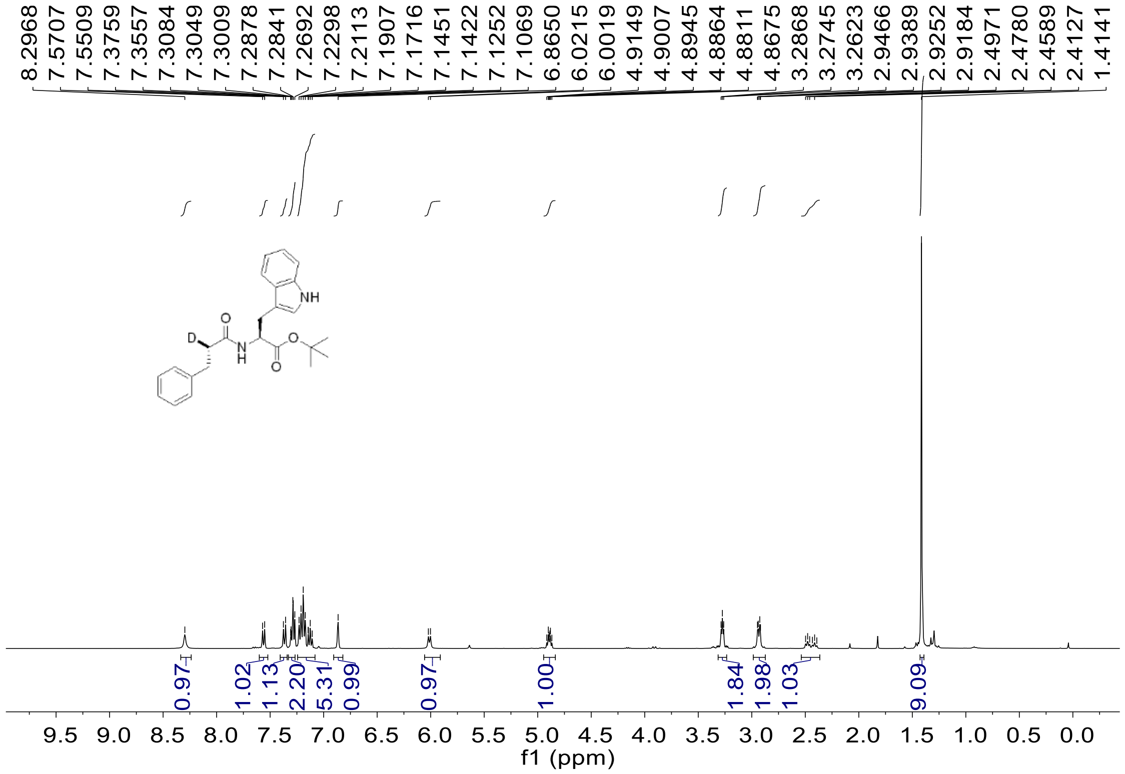


**21c:** $^{13}$C NMR (101 MHz, Chloroform-*d*)

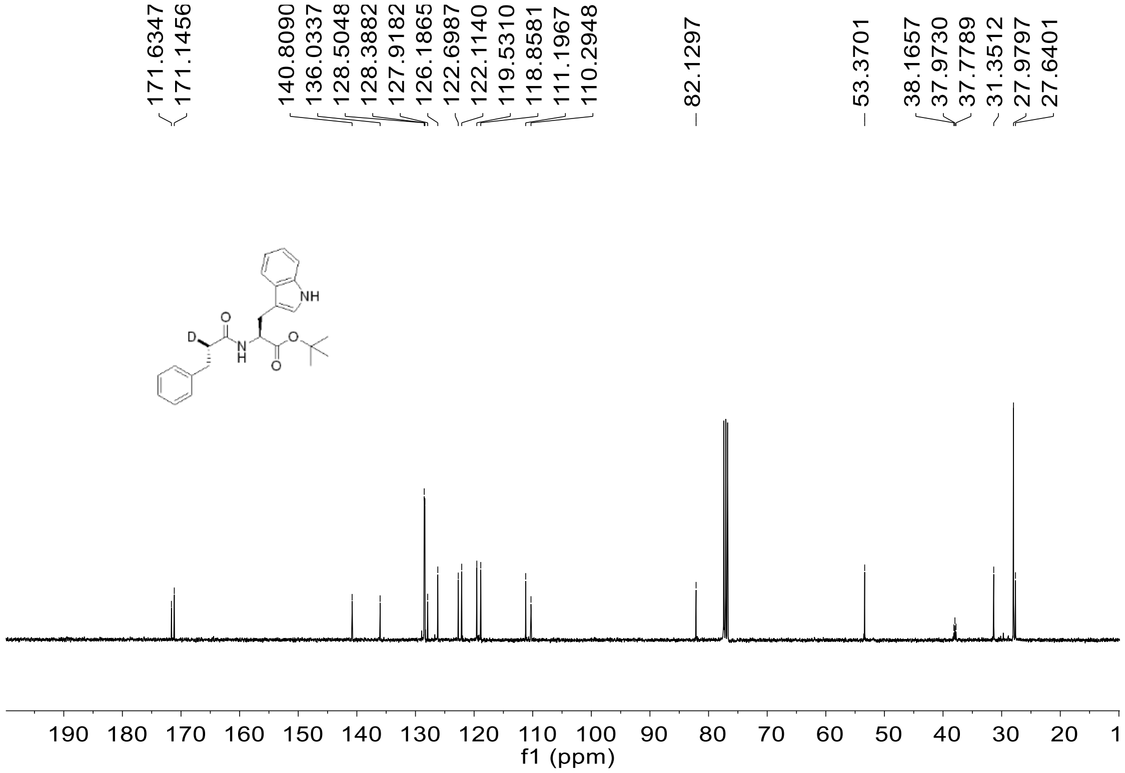

**22b:** $^{1}$H NMR (400 MHz, Chloroform-*d*)

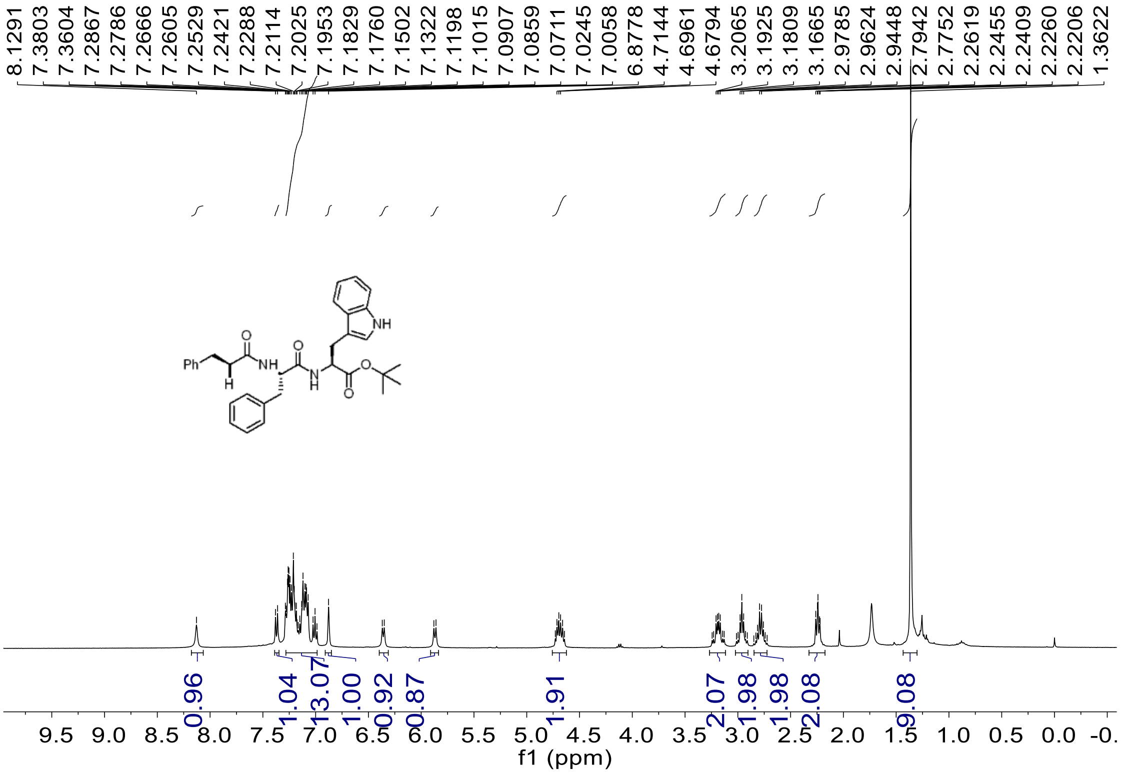


**22b:** $^{13}$C NMR (101 MHz, Chloroform-*d*)

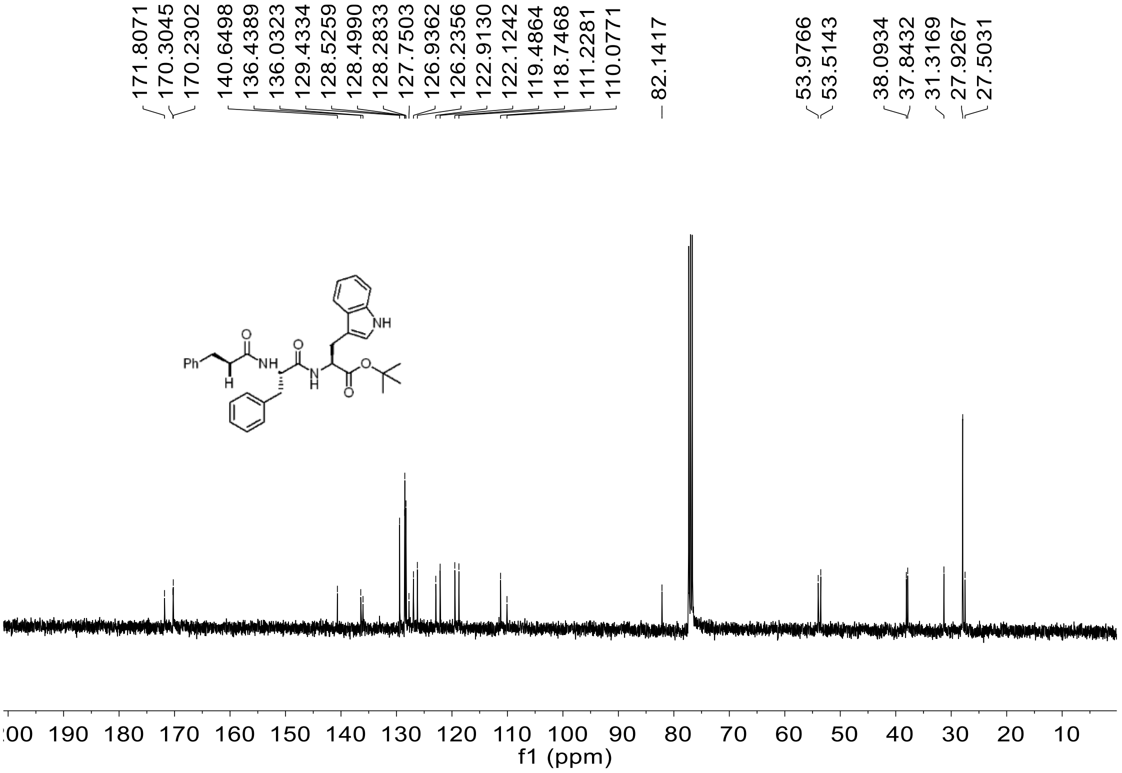

**22c:** $^{1}$H NMR (400 MHz, Chloroform-*d*)

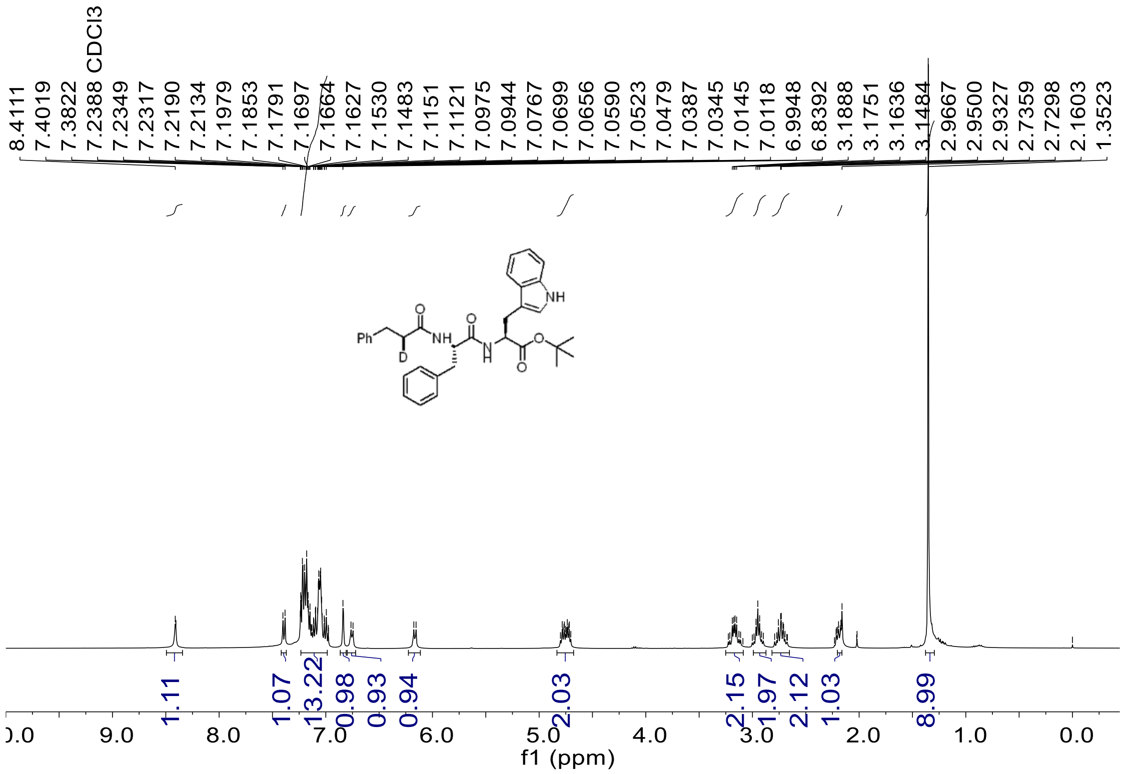


**22c:** $^{13}$C NMR (101 MHz, Chloroform-*d*)

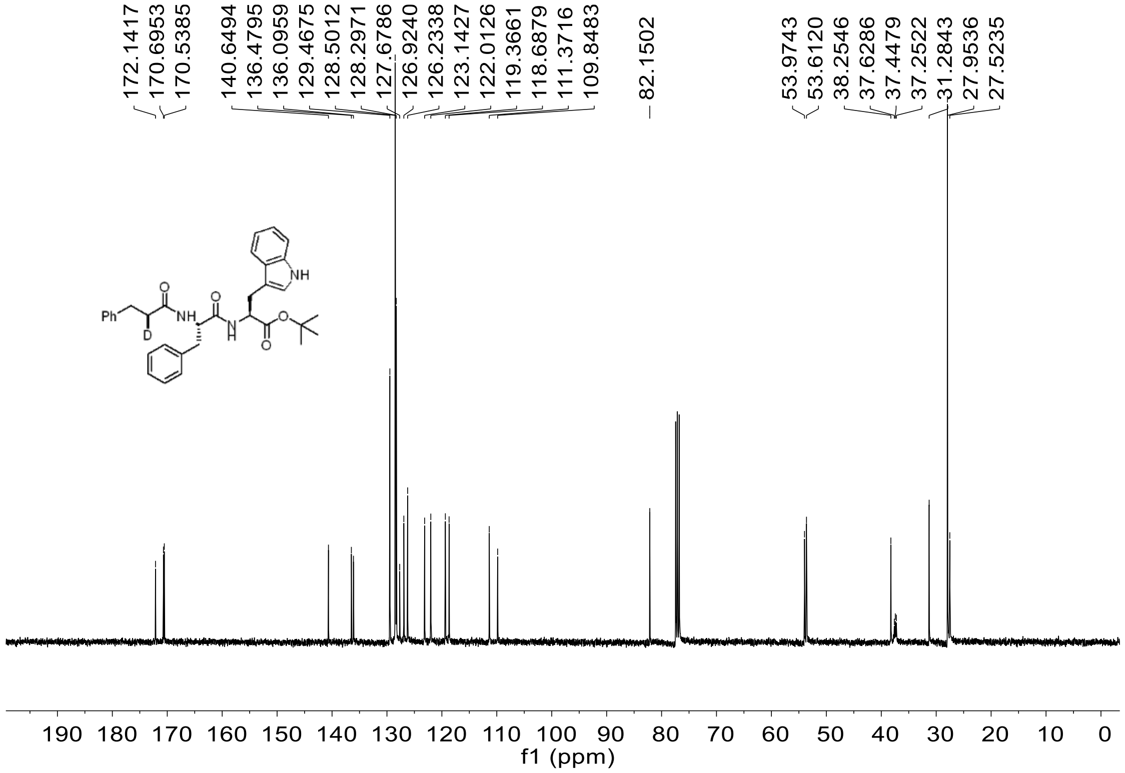

**23b:** $^{1}$H NMR (400 MHz, Chloroform-*d*)

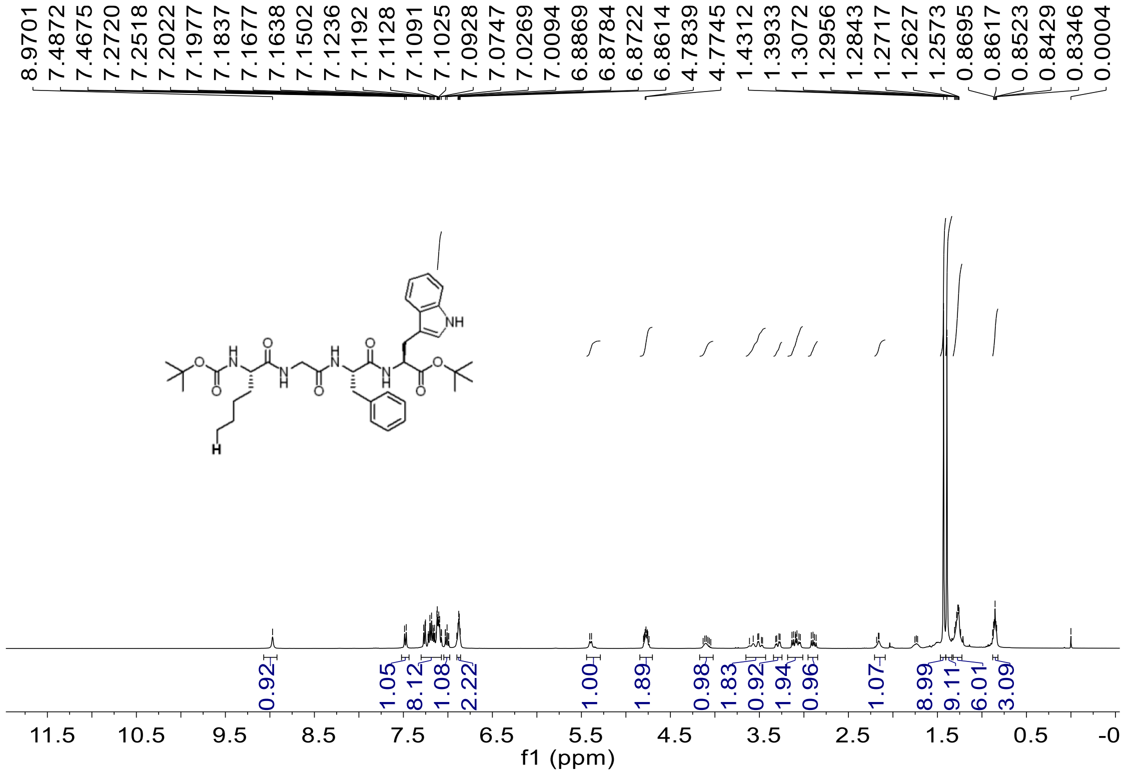


**23b:** $^{13}$C NMR (101 MHz, Chloroform-*d*)

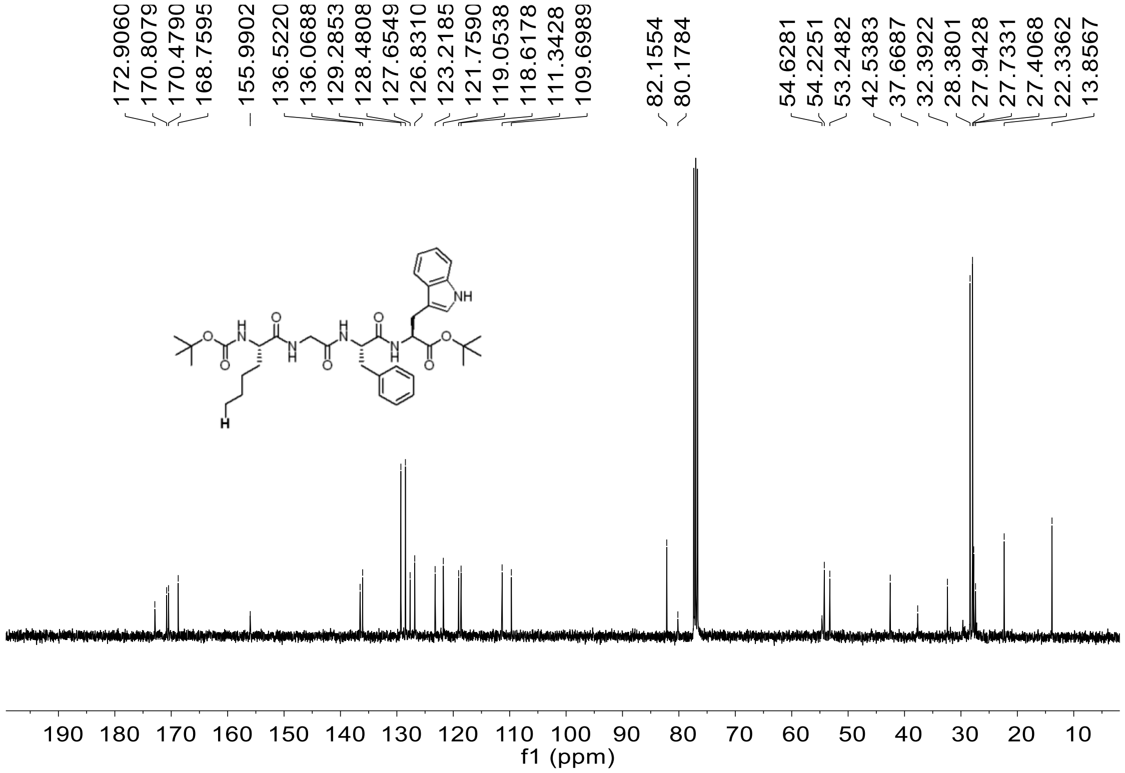

**23c:** $^{1}$H NMR (400 MHz, Chloroform-*d*)

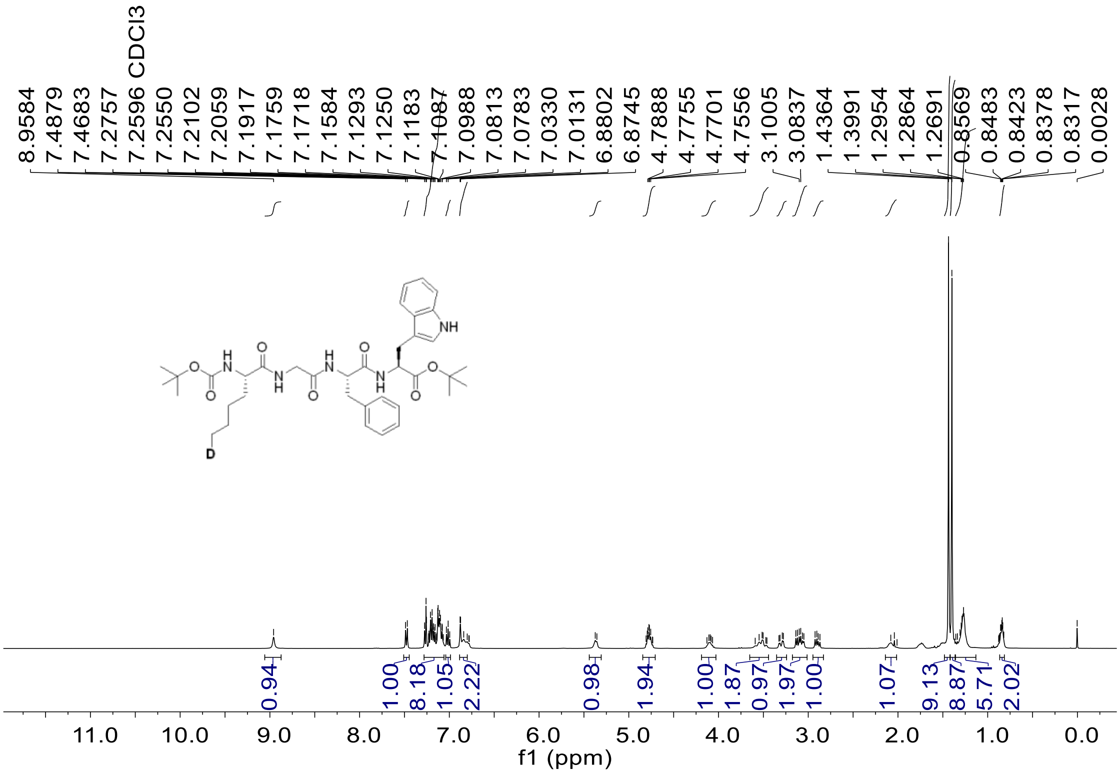


**23c:** $^{13}$C NMR (101 MHz, Chloroform-*d*)

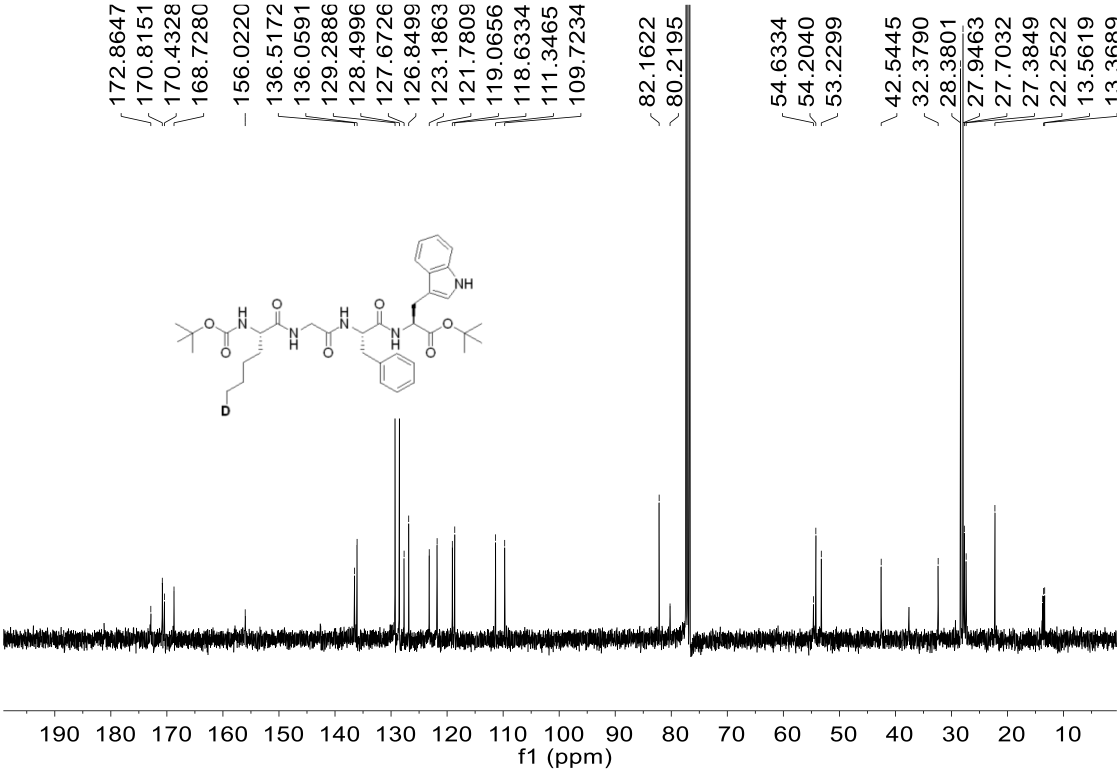

**24b:** $^{1}$H NMR (400 MHz, Chloroform-*d*)

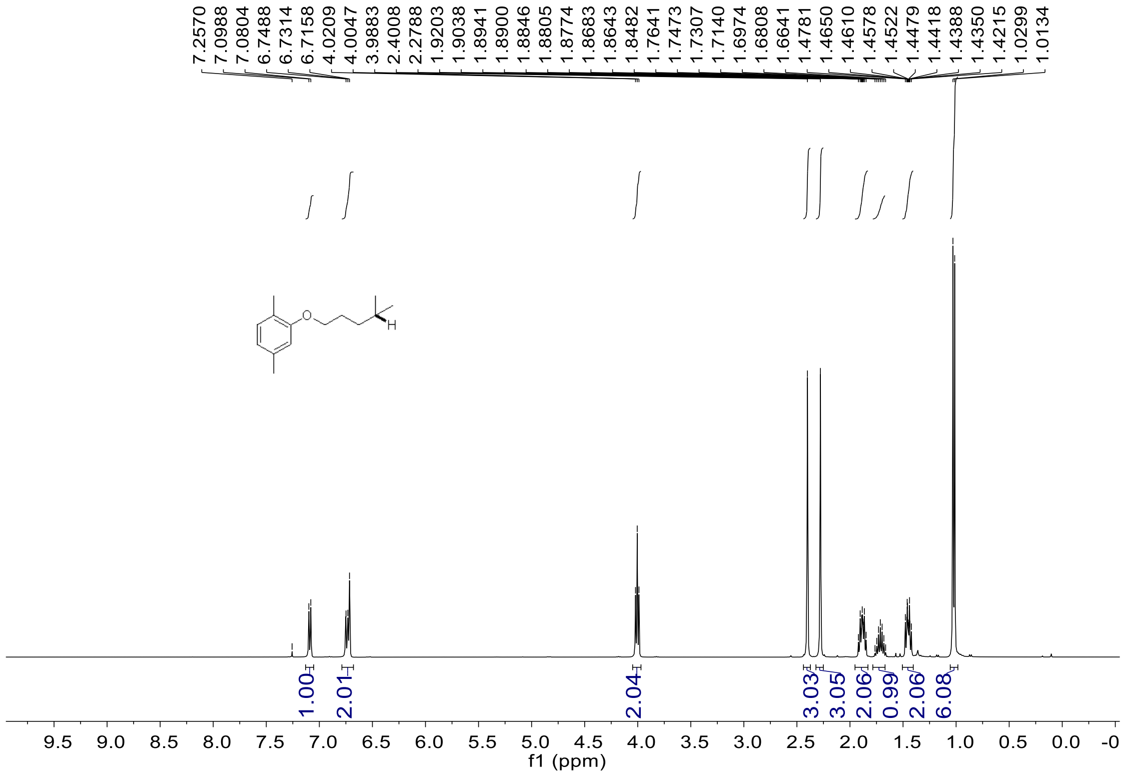

**24b:** $^{13}$C NMR (101 MHz, Chloroform-*d*)

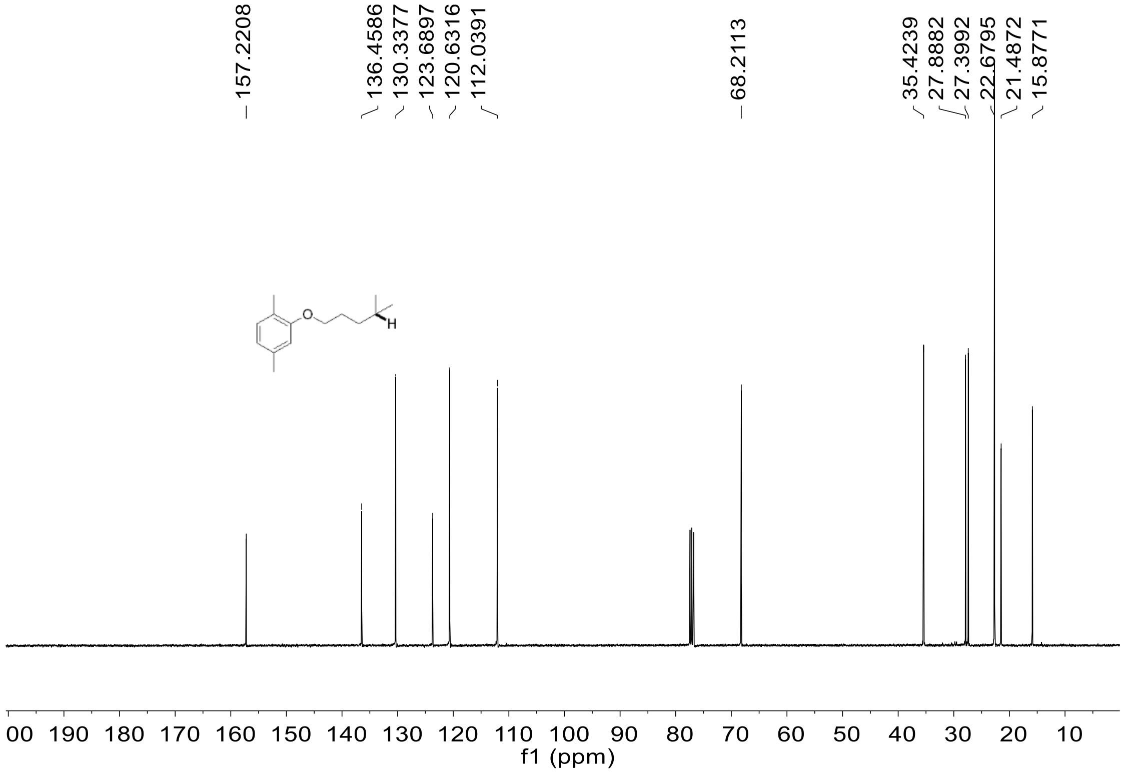

**24c:** $^{1}$H NMR (400 MHz, Chloroform-*d*)

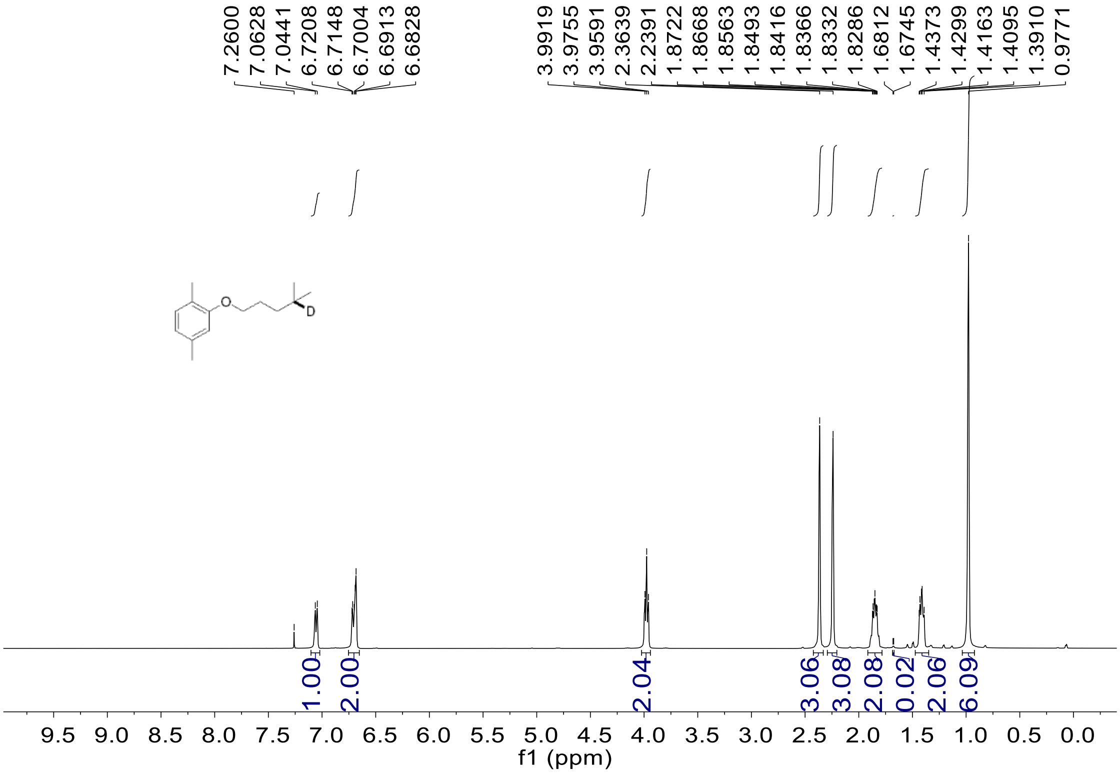


**24c:** $^{13}$C NMR (101 MHz, Chloroform-*d*)

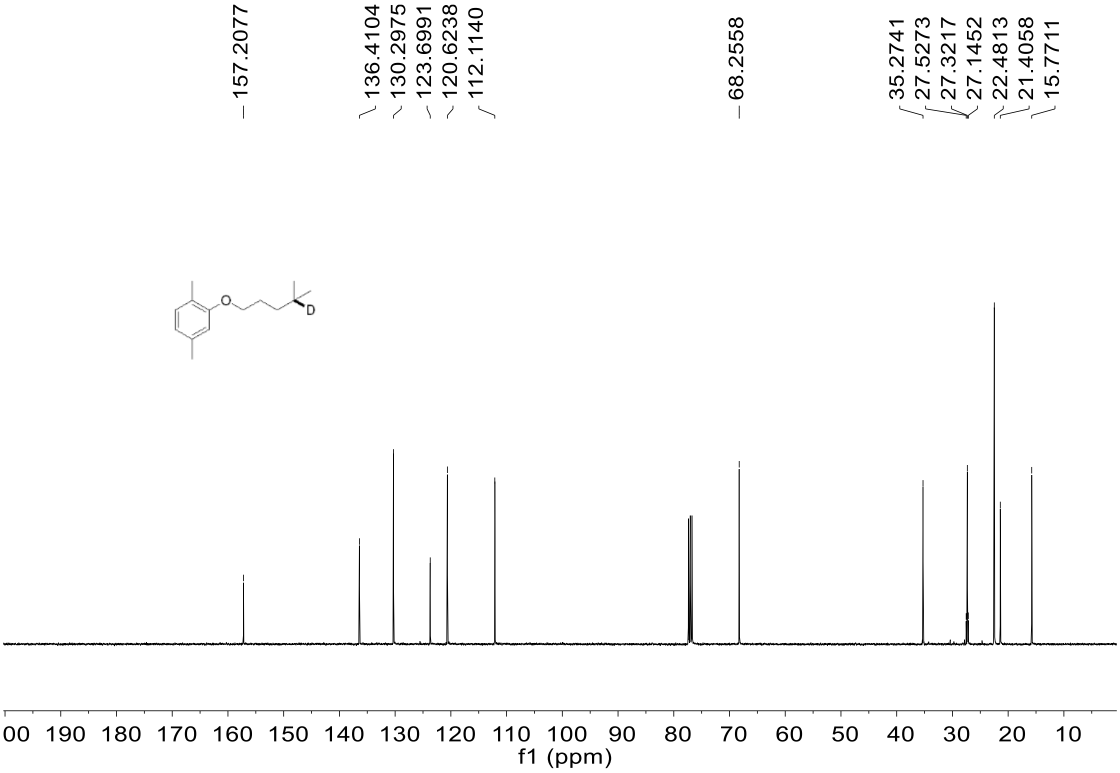

**25b:** $^{1}$H NMR (400 MHz, Chloroform-*d*)

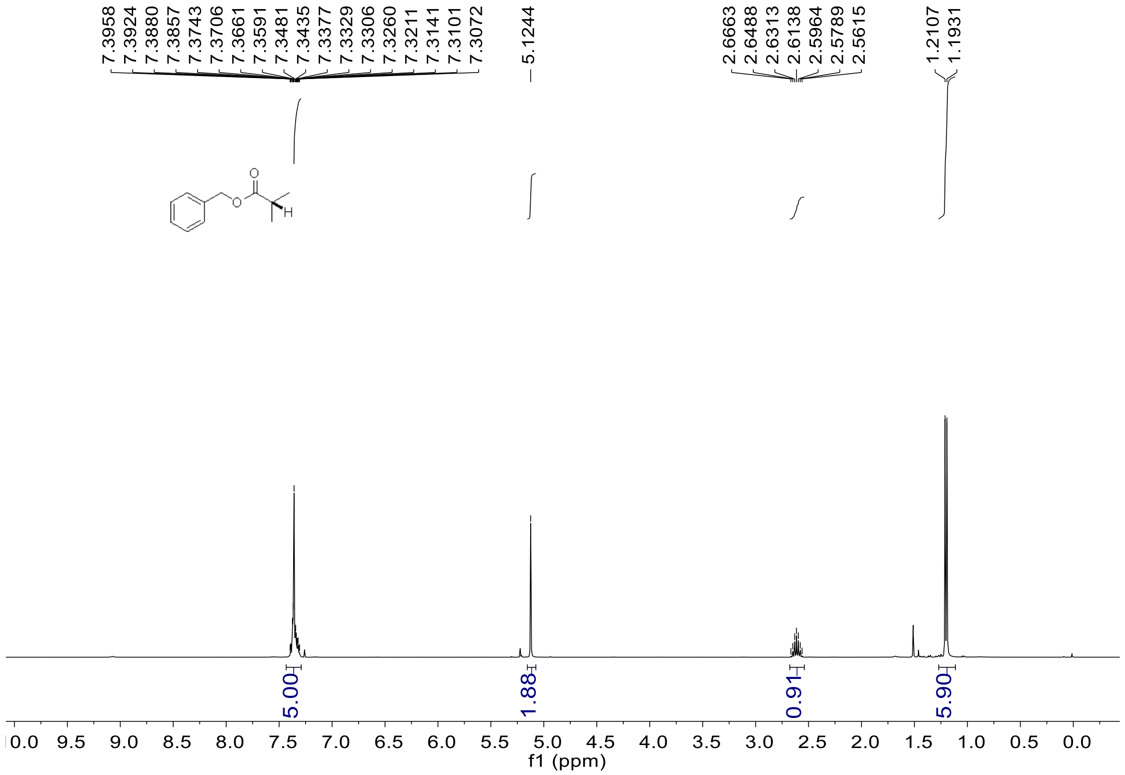


**25b:** $^{13}$C NMR (101 MHz, Chloroform-*d*)

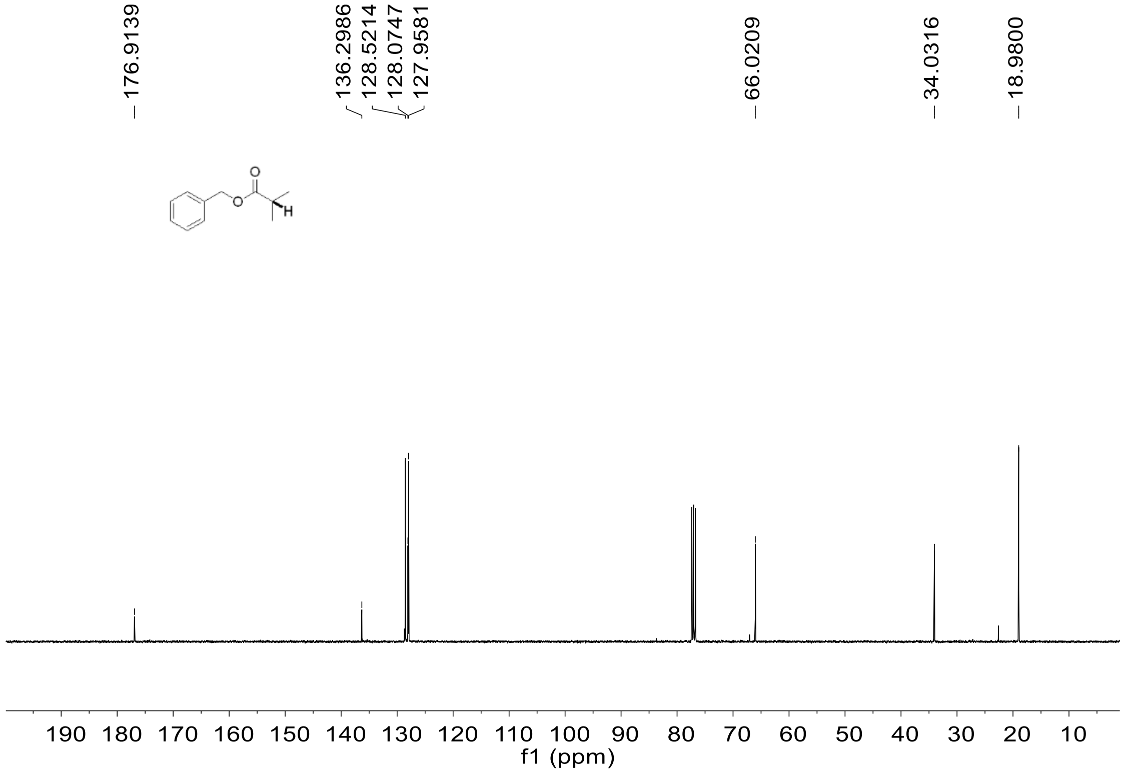

**25c:** $^{1}$H NMR (400 MHz, Chloroform-*d*)

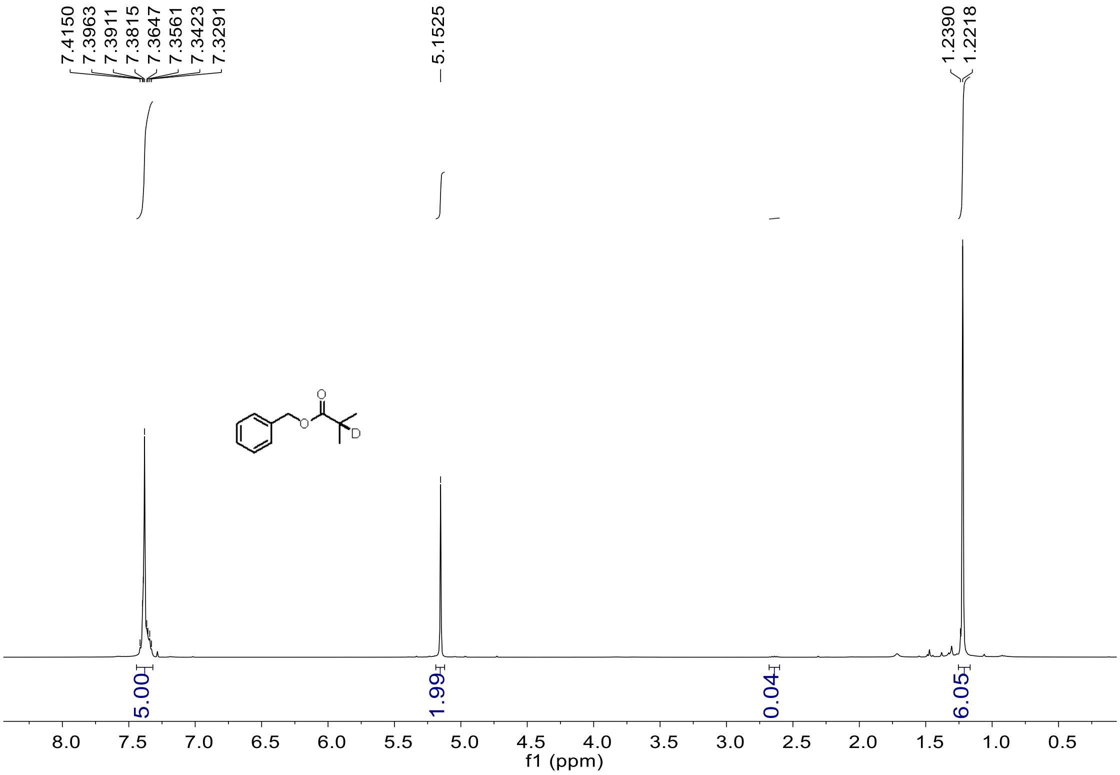


**25c:** $^{13}$C NMR (101 MHz, Chloroform-*d*)

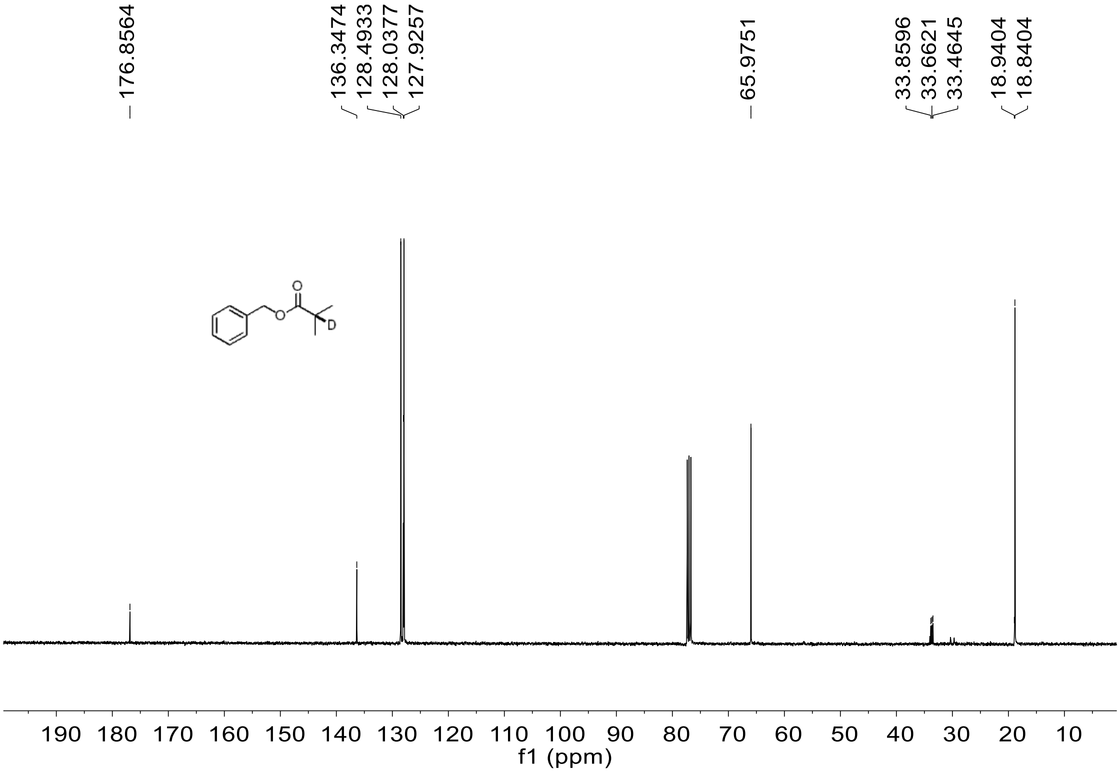

**26b:** $^{1}$H NMR (400 MHz, Deuterium Oxide)

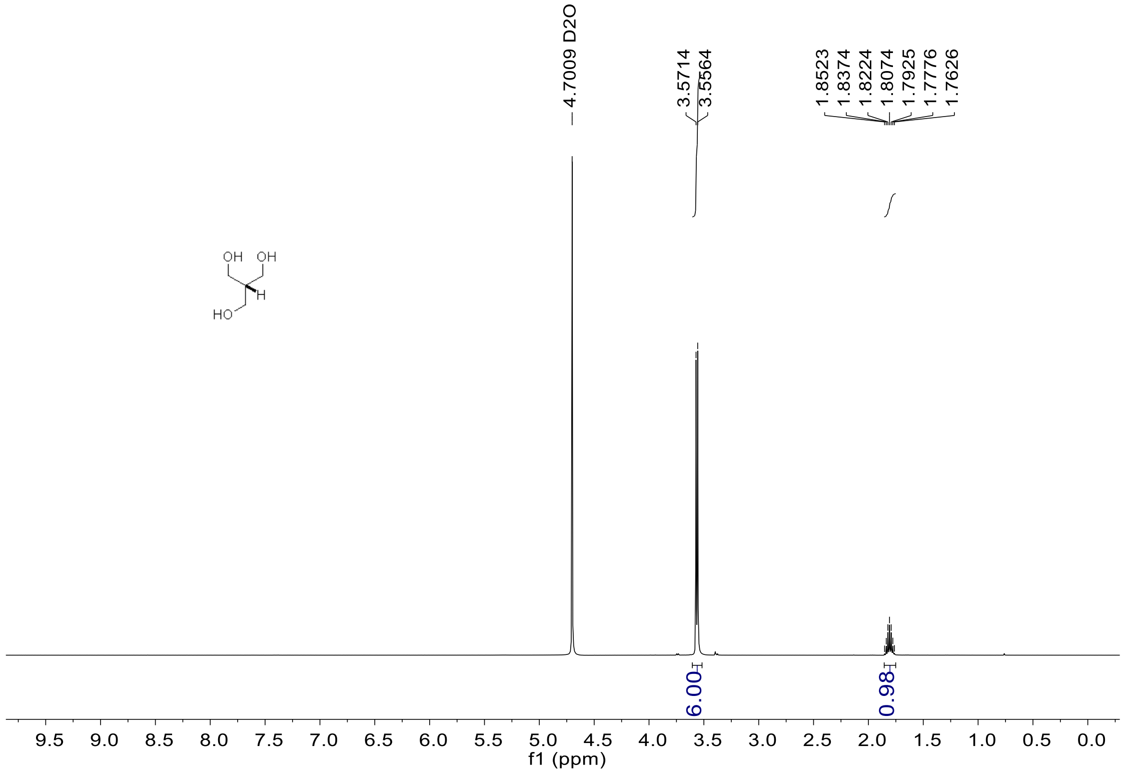


**26b:** $^{13}$C NMR (101 MHz, Deuterium Oxide)

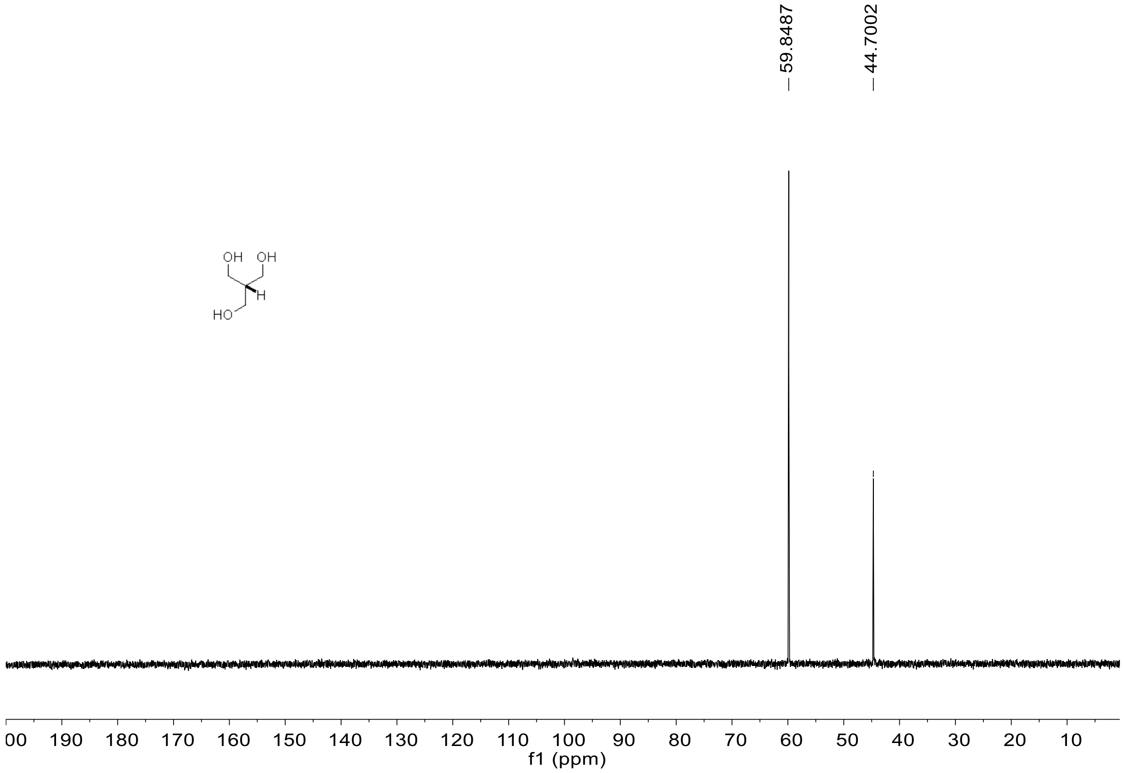

**26c:** $^{1}$H NMR (400 MHz, Deuterium Oxide)

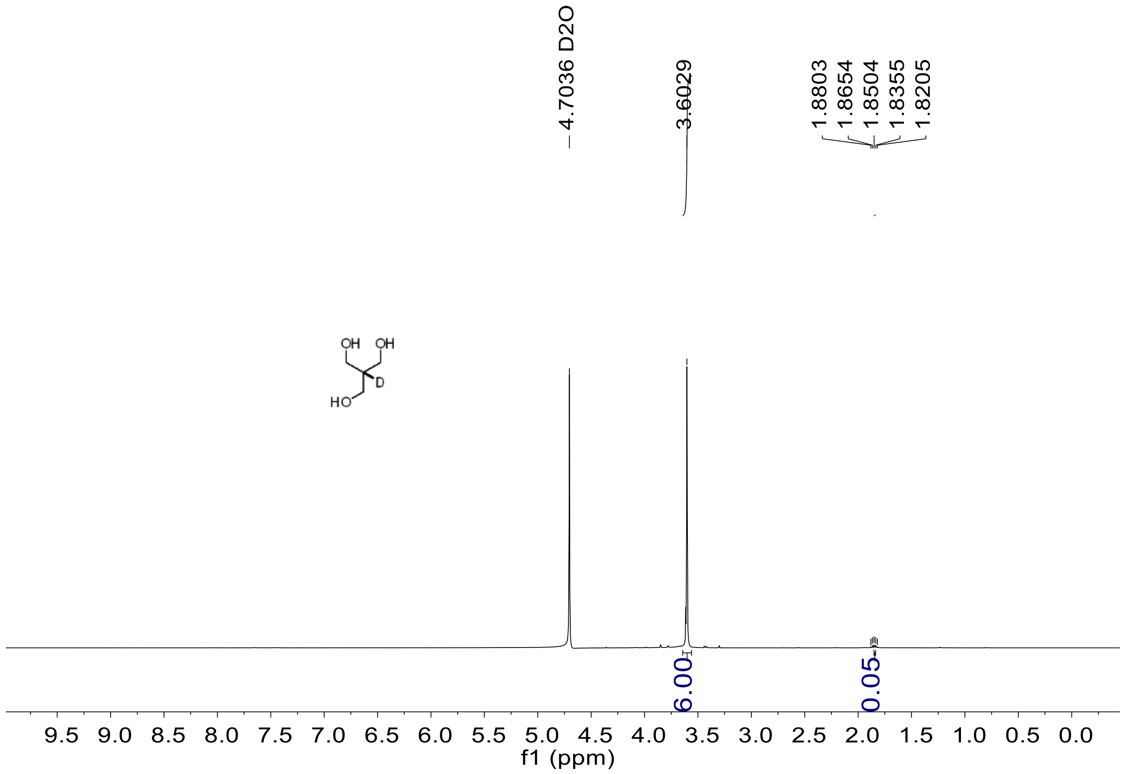


**26c:** $^{13}$C NMR (101 MHz, Deuterium Oxide)

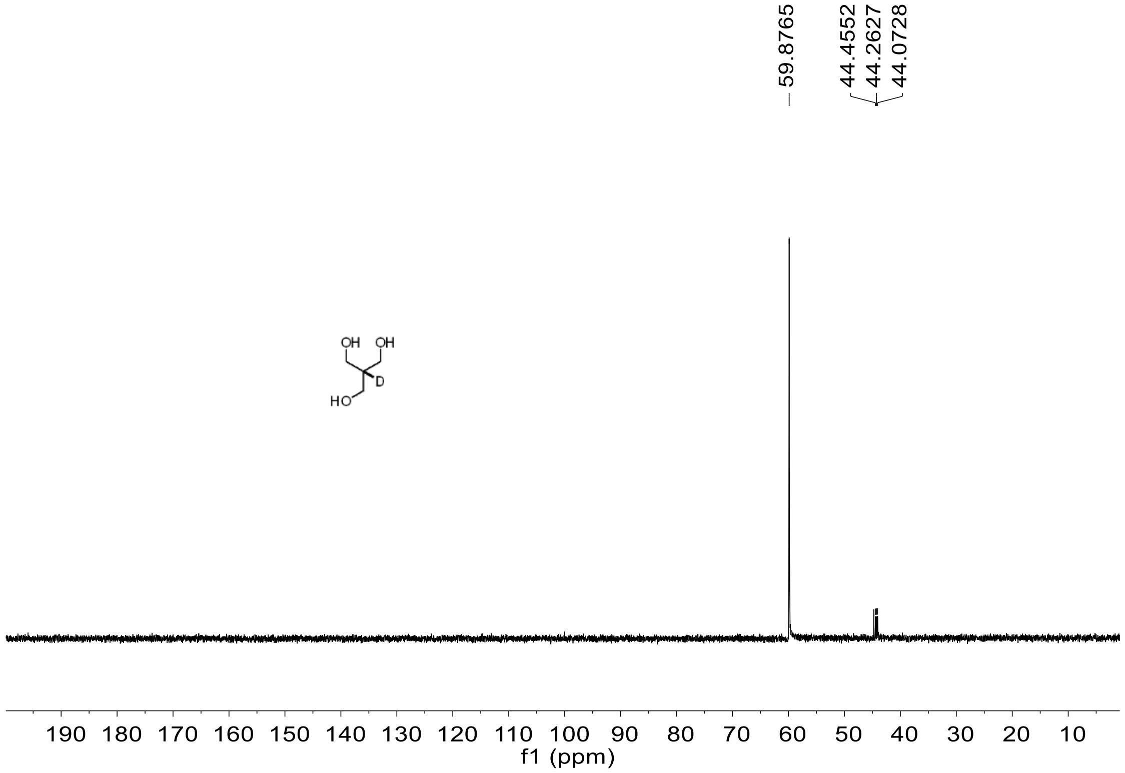

**27b:** $^{1}$H NMR (400 MHz, Chloroform-*d*)

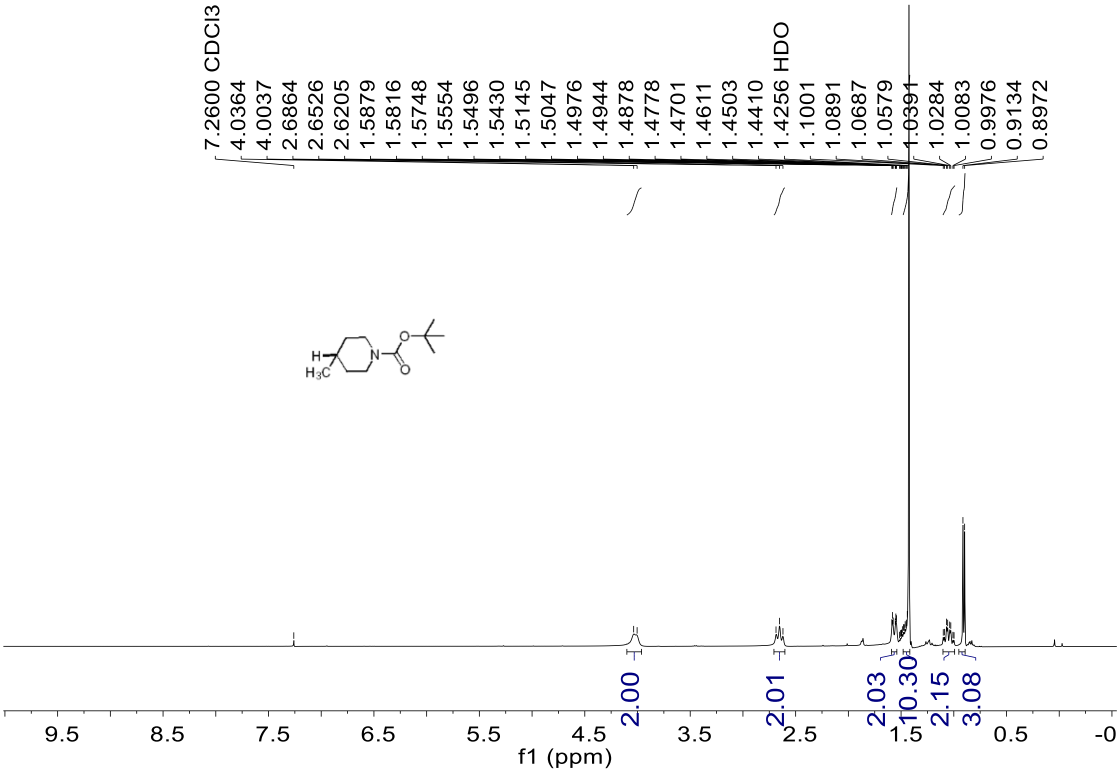


**27b:** $^{13}$C NMR (101 MHz, Chloroform-*d*)

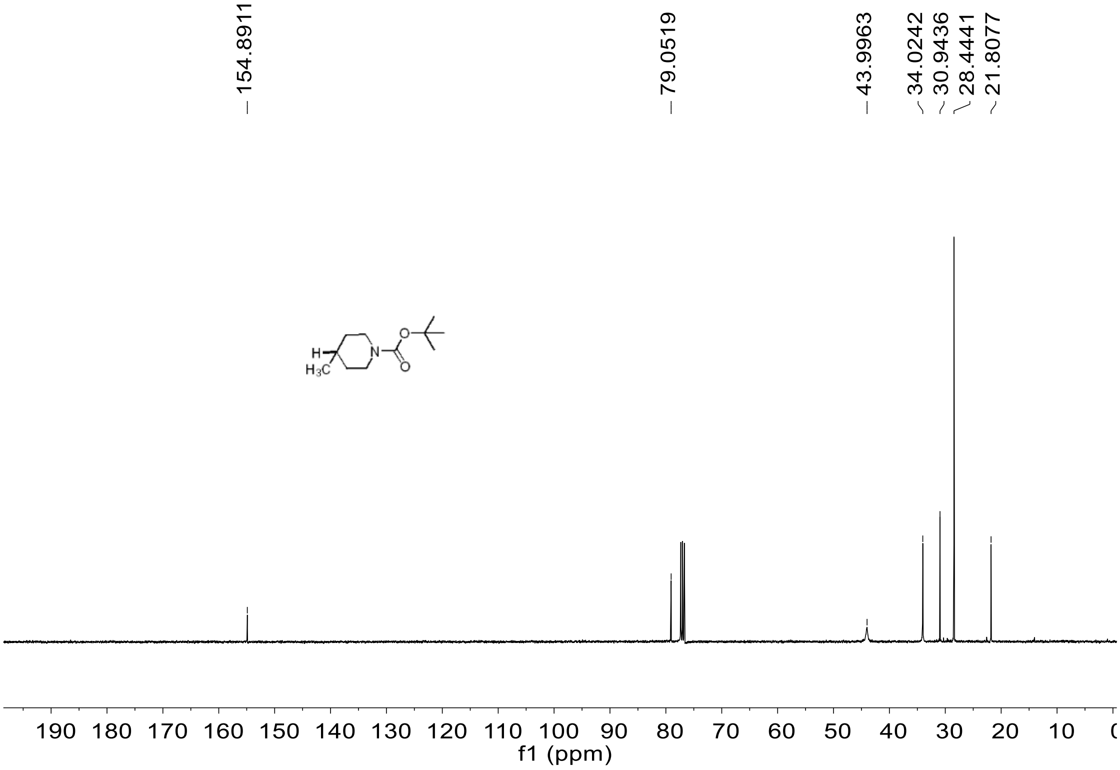

**27c:** $^{1}$H NMR (400 MHz, Chloroform-*d*)

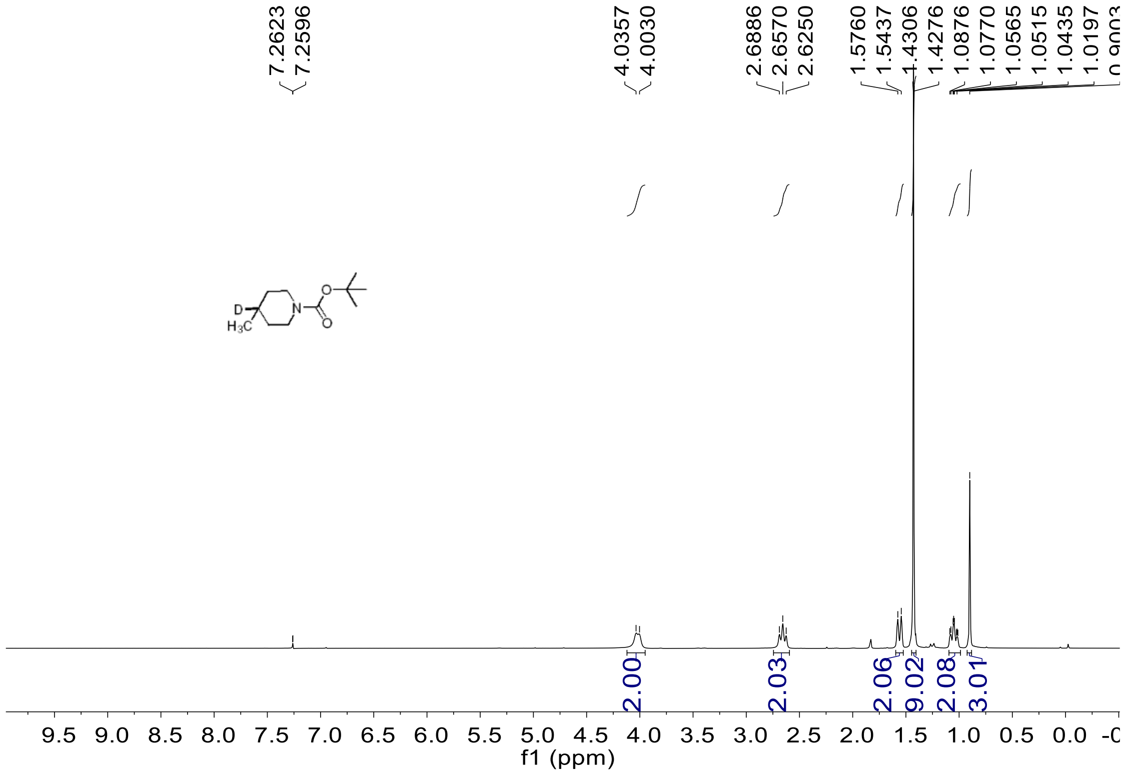


**27c:** $^{13}$C NMR (101 MHz, Chloroform-*d*)

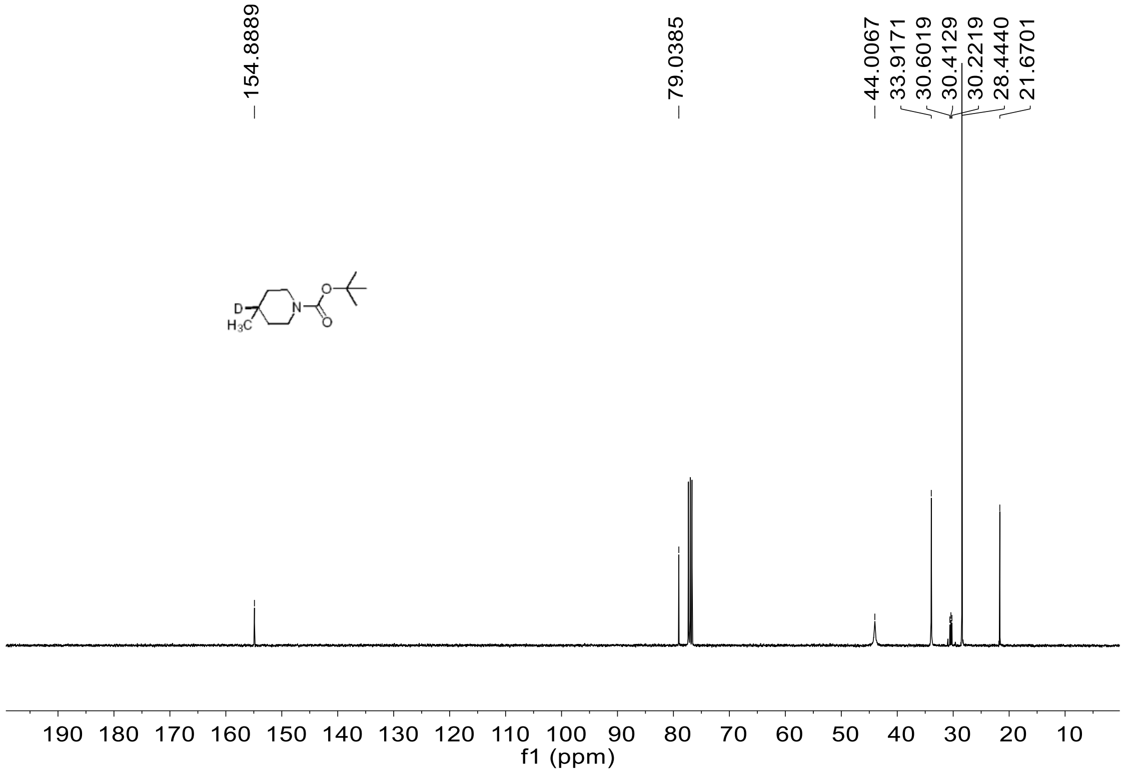

**28b:** $^{1}$H NMR (400 MHz, Chloroform-*d*)

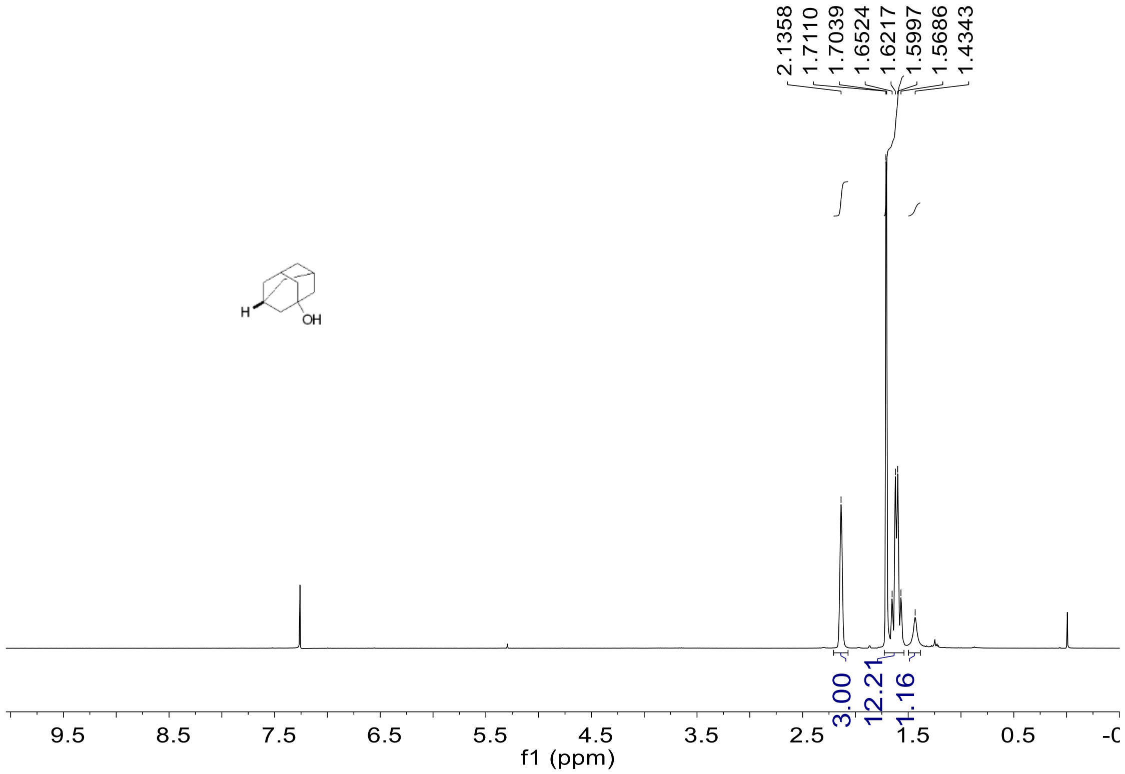


**28b:** $^{13}$C NMR (101 MHz, Chloroform-*d*)

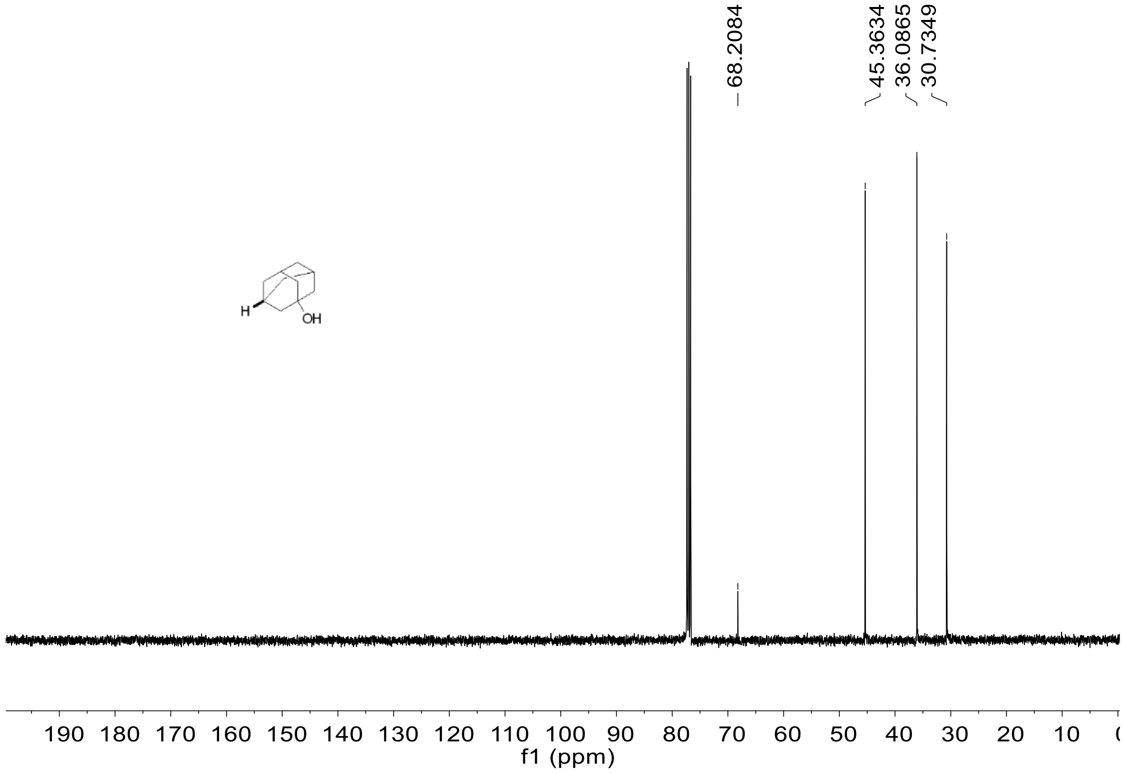

**28c:** $^{1}$H NMR (400 MHz, Chloroform-$d$)

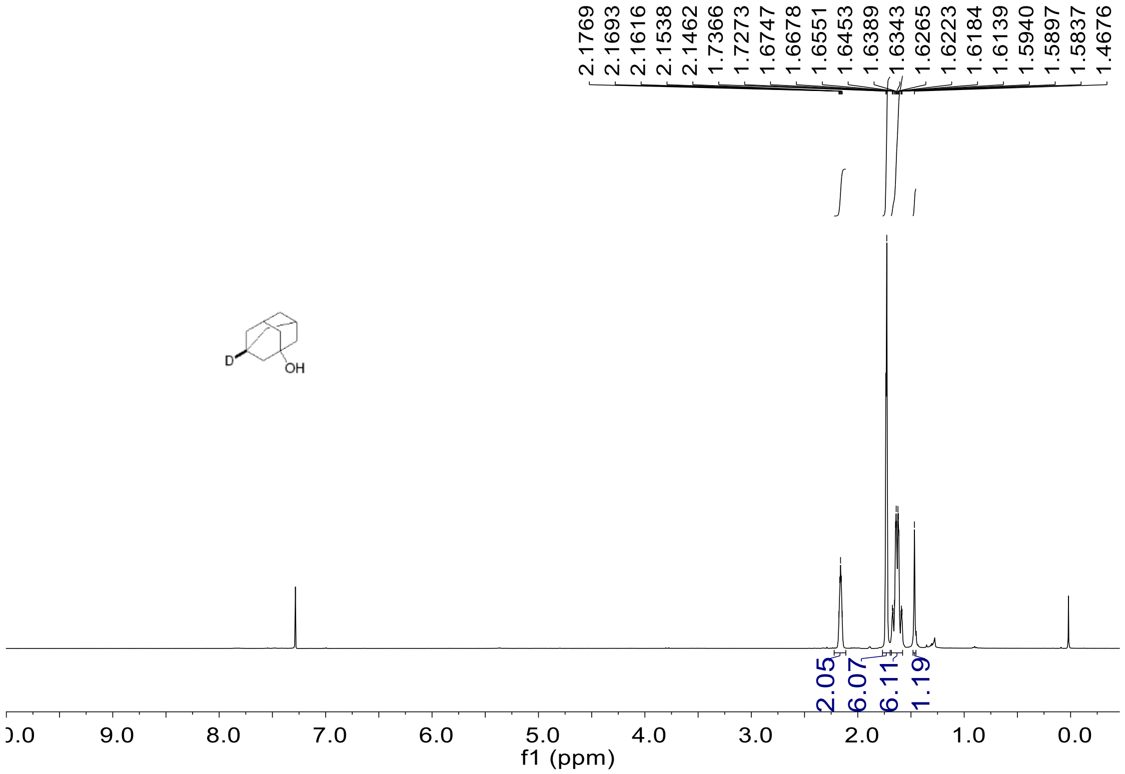


**28c:** $^{13}$C NMR (101 MHz, Chloroform-$d$)

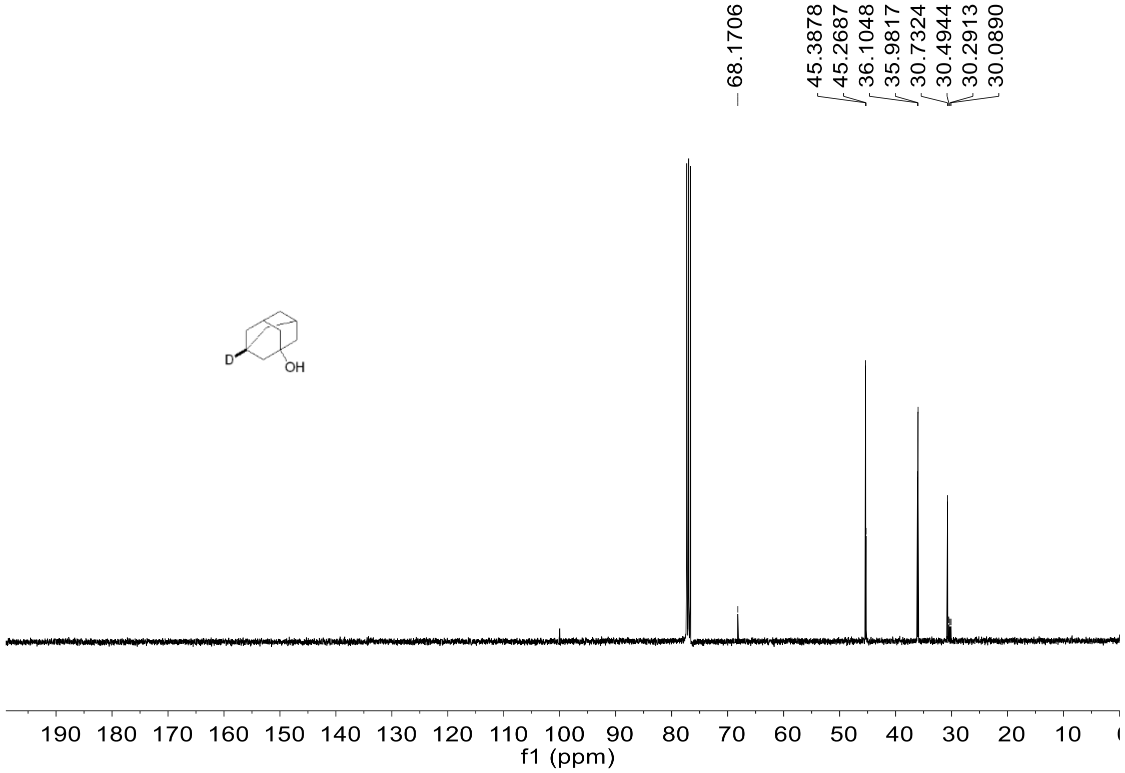

**29b:** $^{1}$H NMR (400 MHz, Chloroform-*d*)

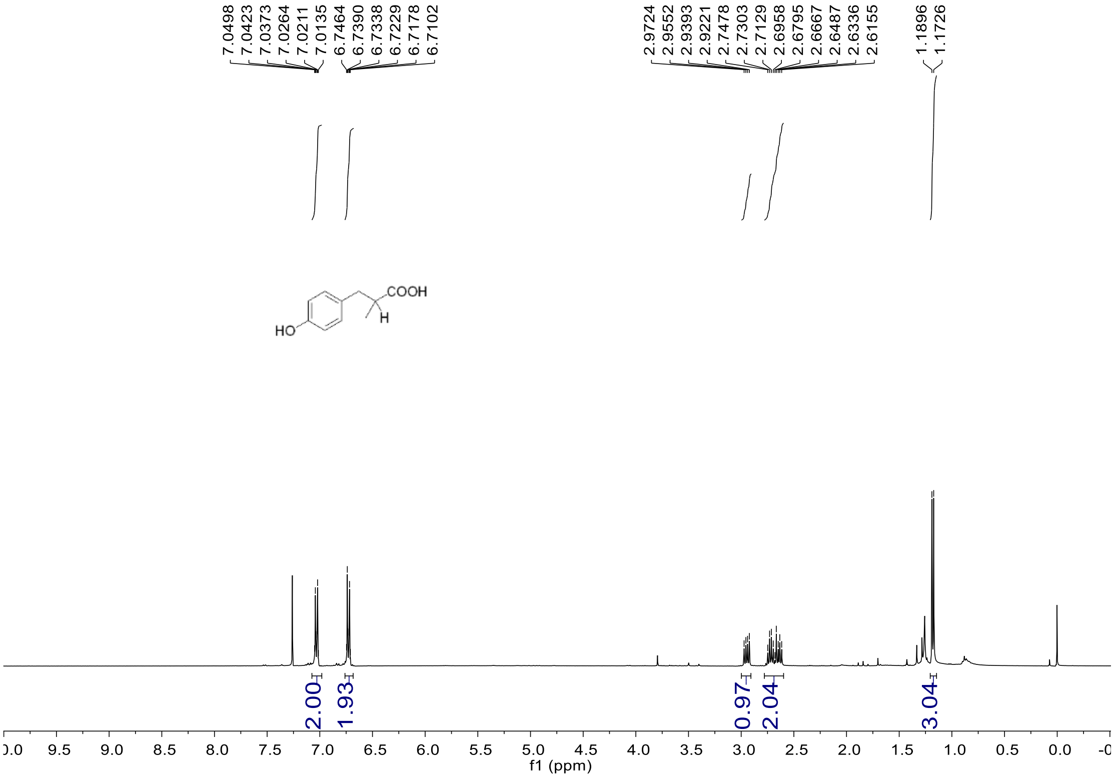


**29b:** $^{13}$C NMR (101 MHz, Chloroform-*d*)

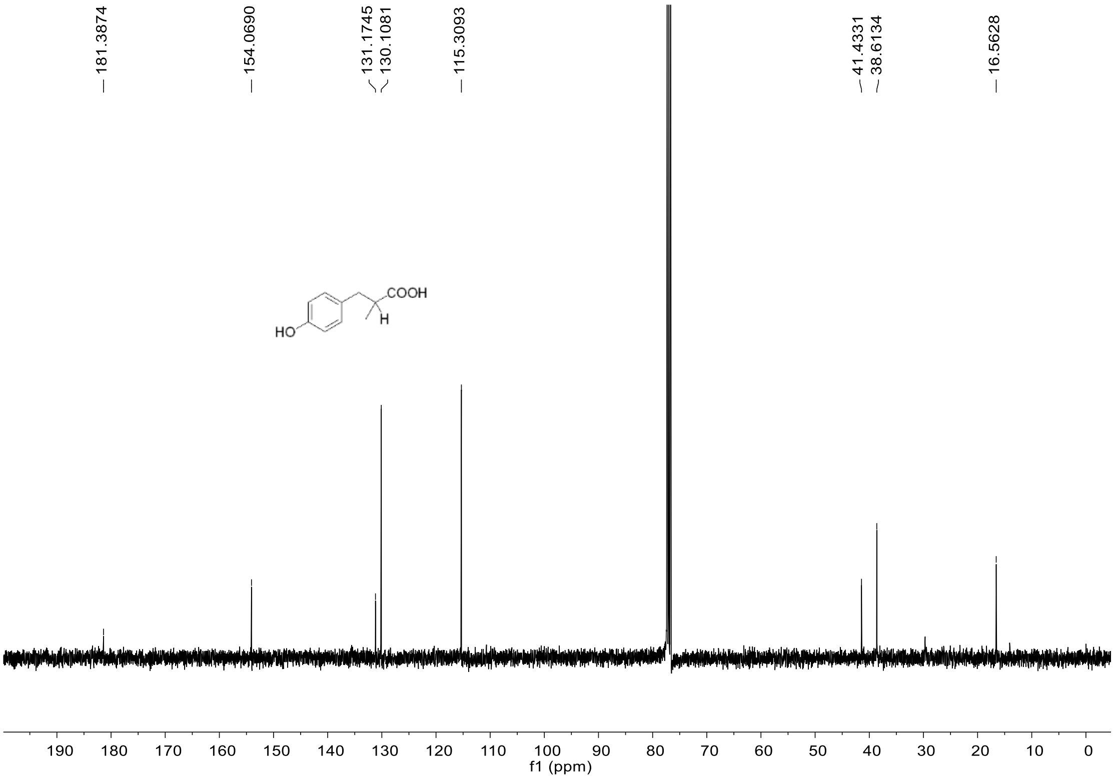

**29c:** $^{1}$H NMR (400 MHz, Chloroform-*d*)

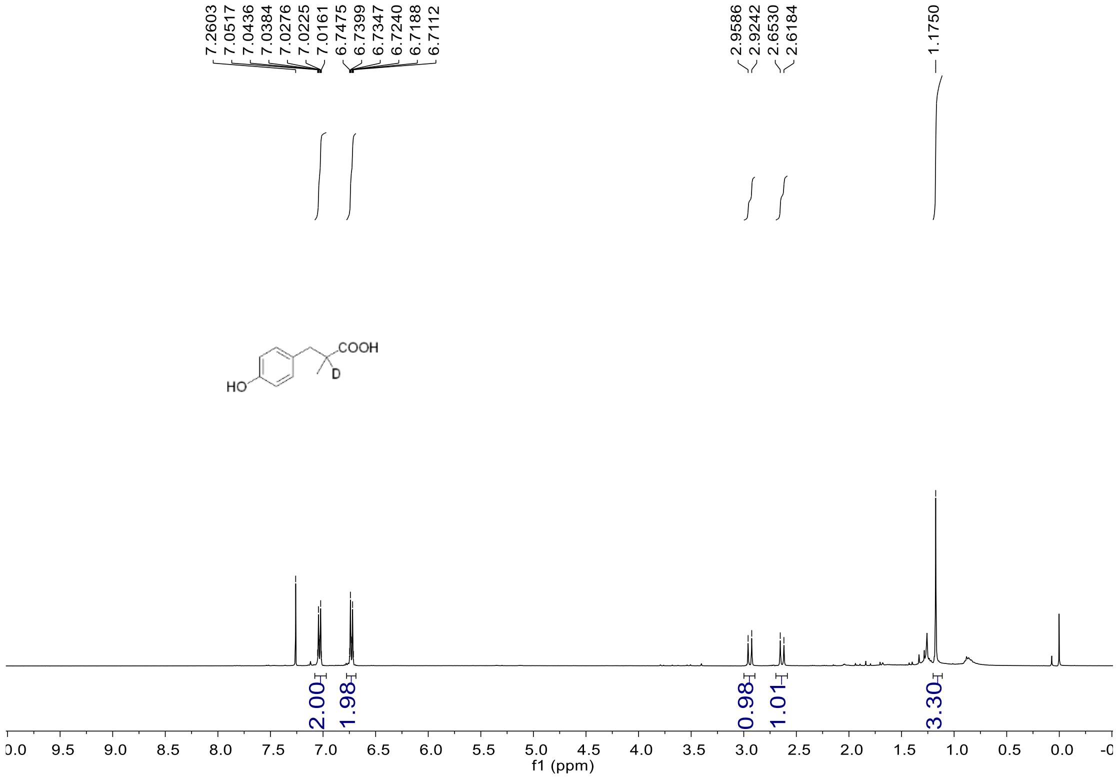


**29c:** $^{13}$C NMR (101 MHz, Chloroform-*d*)

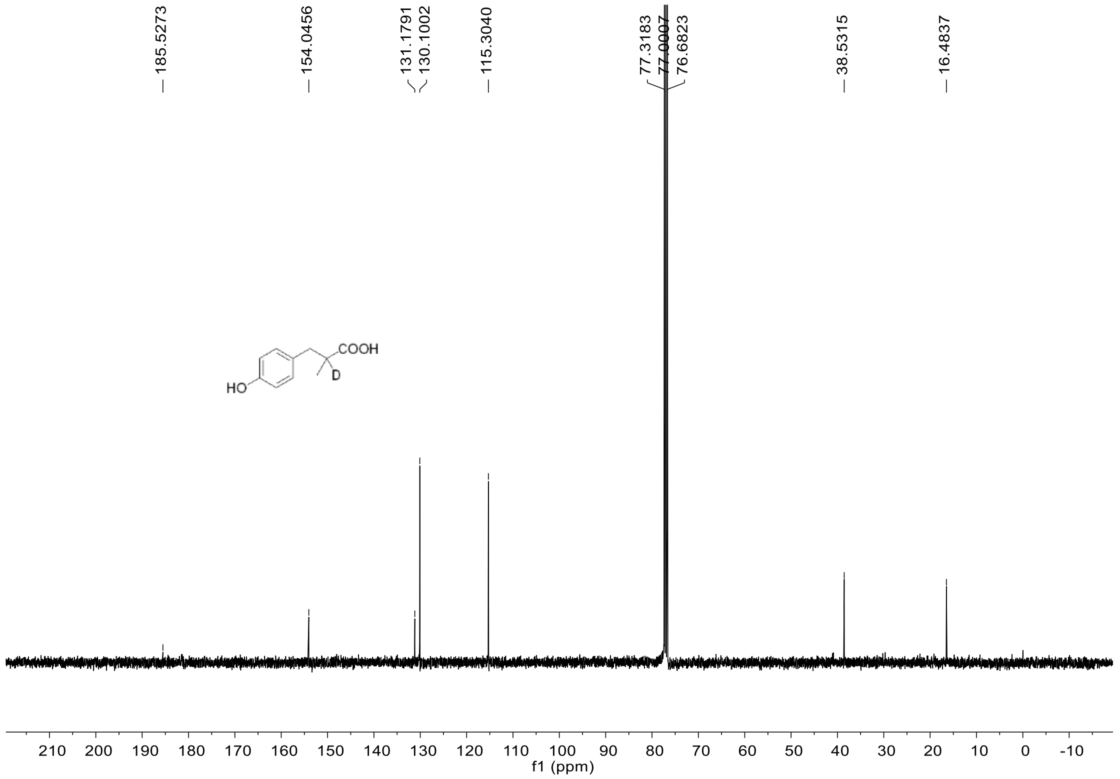

**30b:** $^{1}$H NMR (400 MHz, Chloroform-*d*)

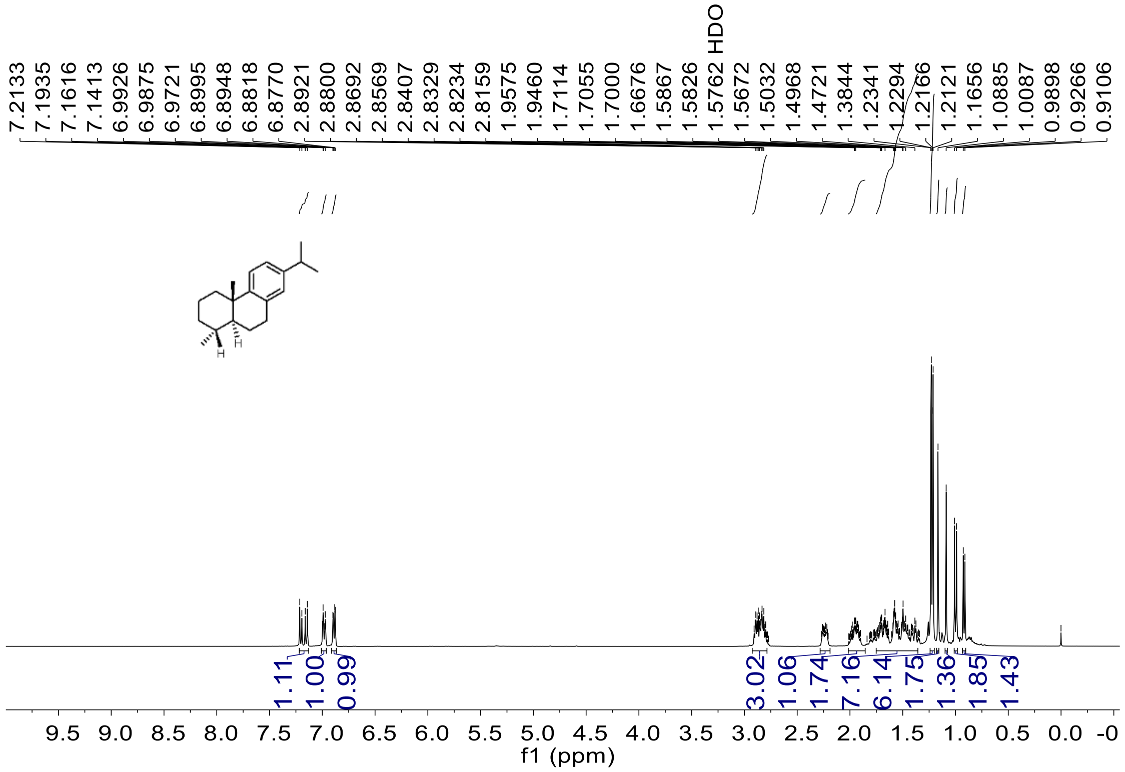


**30b:** $^{13}$C NMR (101 MHz, Chloroform-*d*)

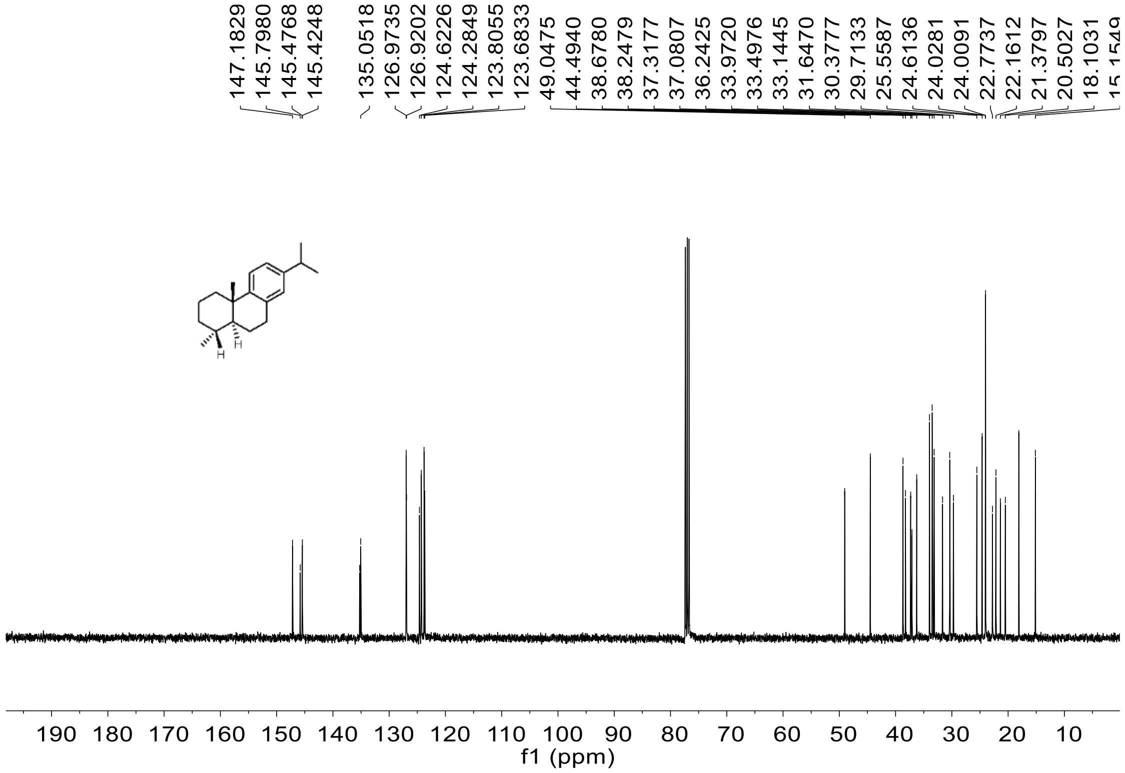

**30c:** $^{1}$H NMR (400 MHz, Chloroform-*d*)

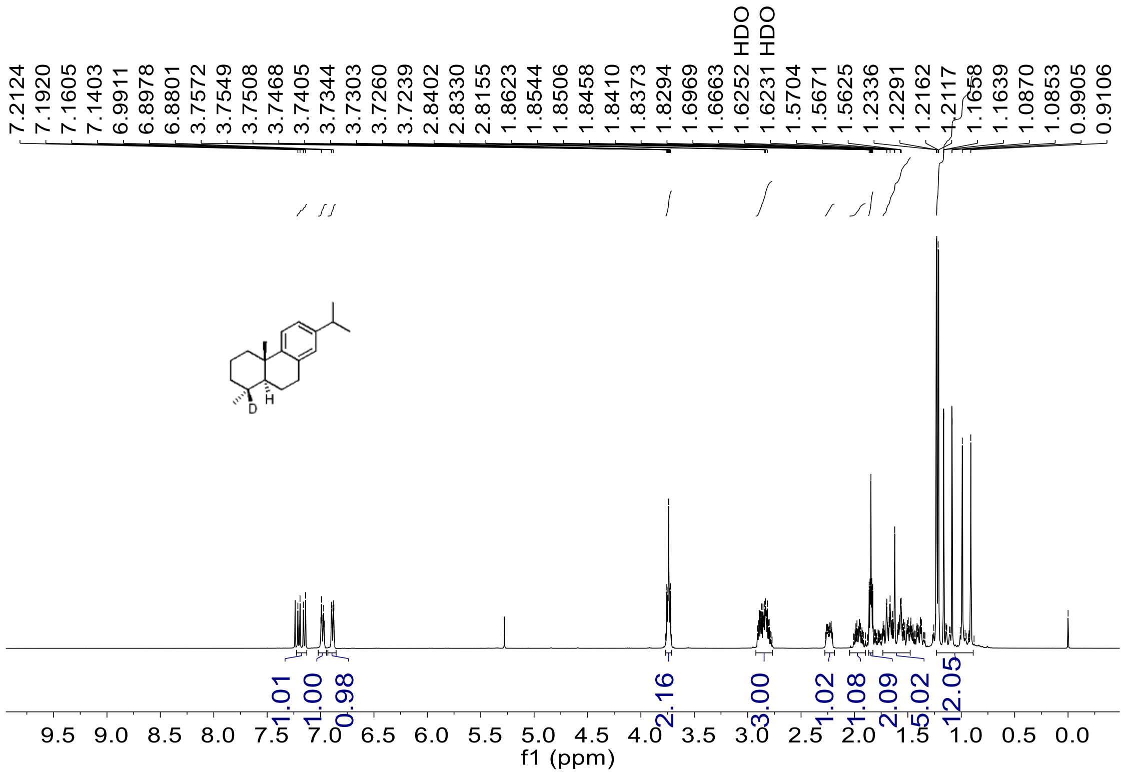


**30c:** $^{13}$C NMR (101 MHz, Chloroform-*d*)

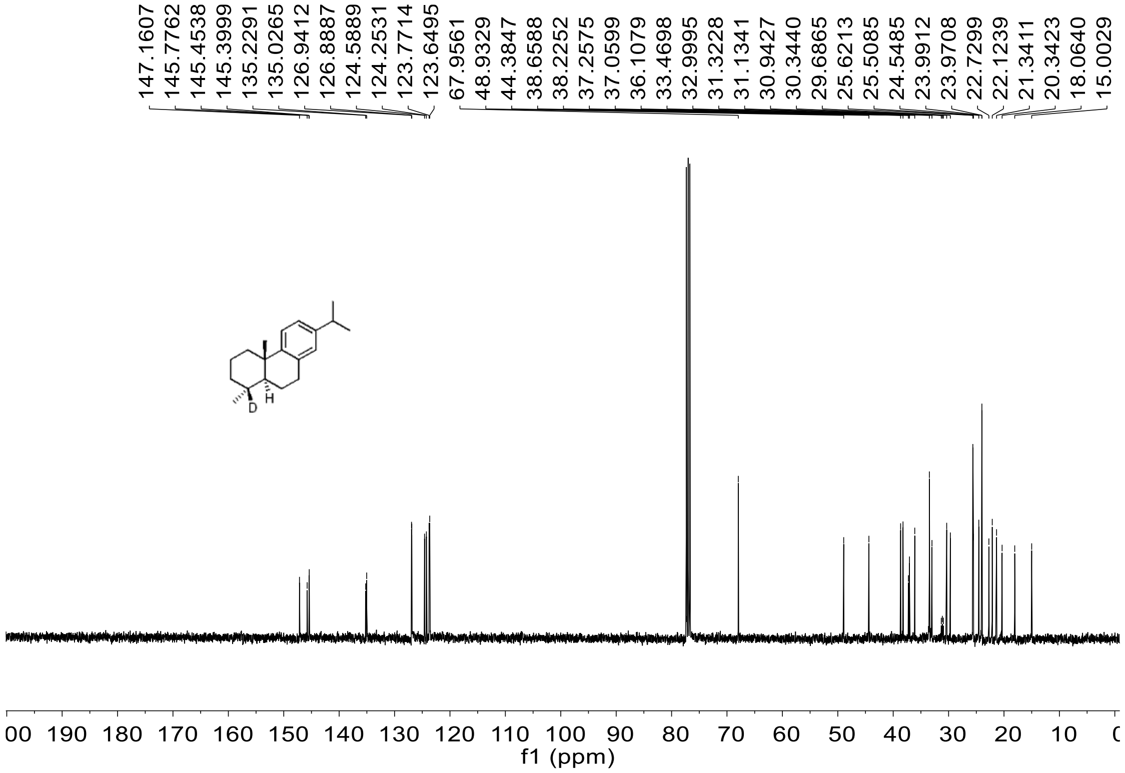

**32b:** $^{1}$H NMR (400 MHz, Chloroform-*d*)

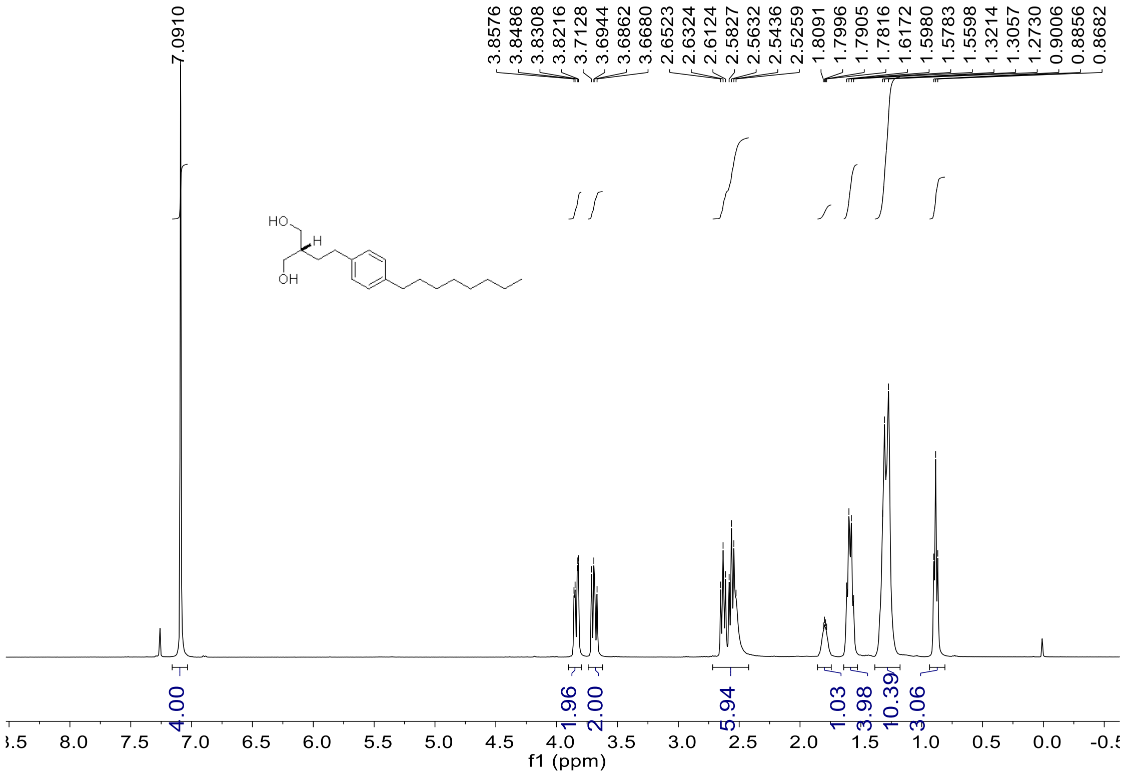


**32b:** $^{13}$C NMR (101 MHz, Chloroform-*d*)

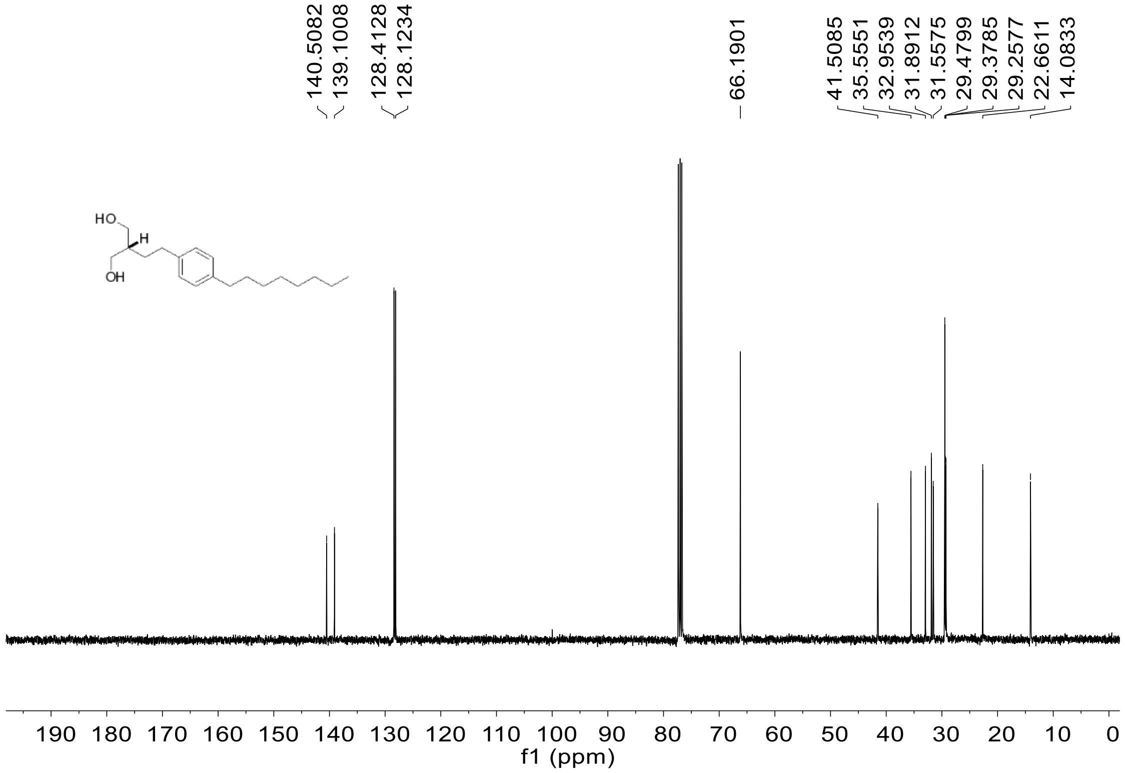

**32c:** $^{1}$H NMR (400 MHz, Chloroform-*d*)

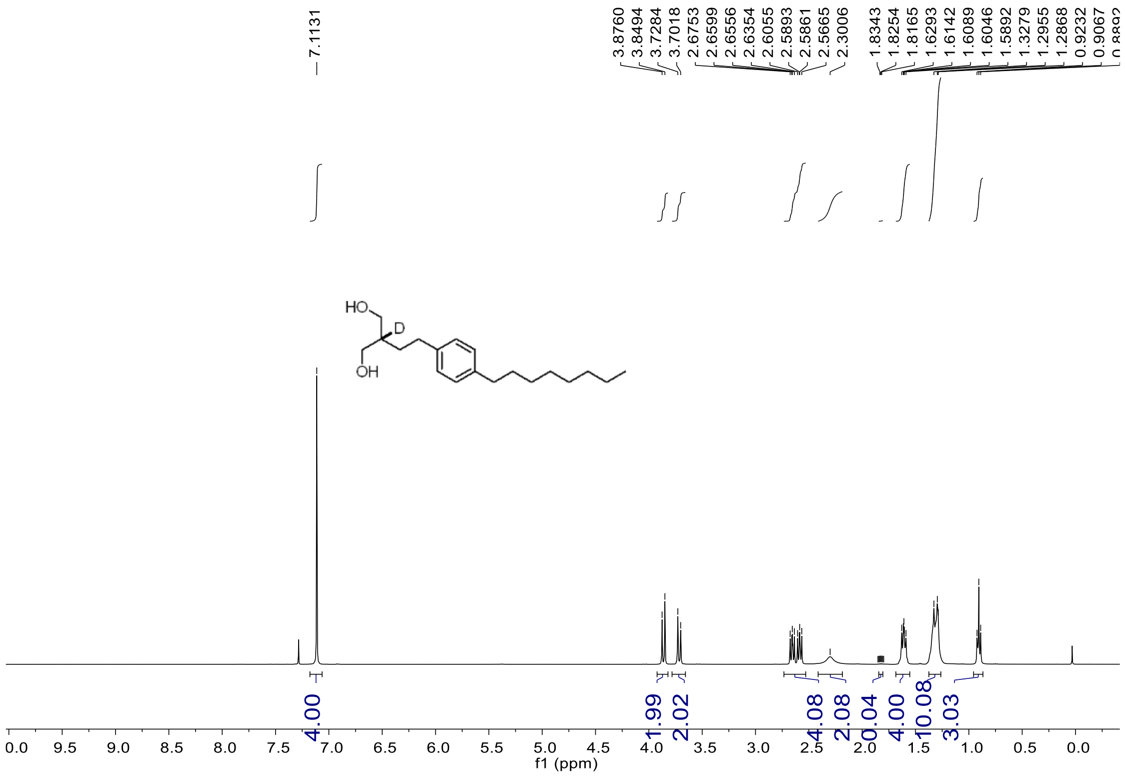


**32c:** $^{13}$C NMR (101 MHz, Chloroform-*d*)

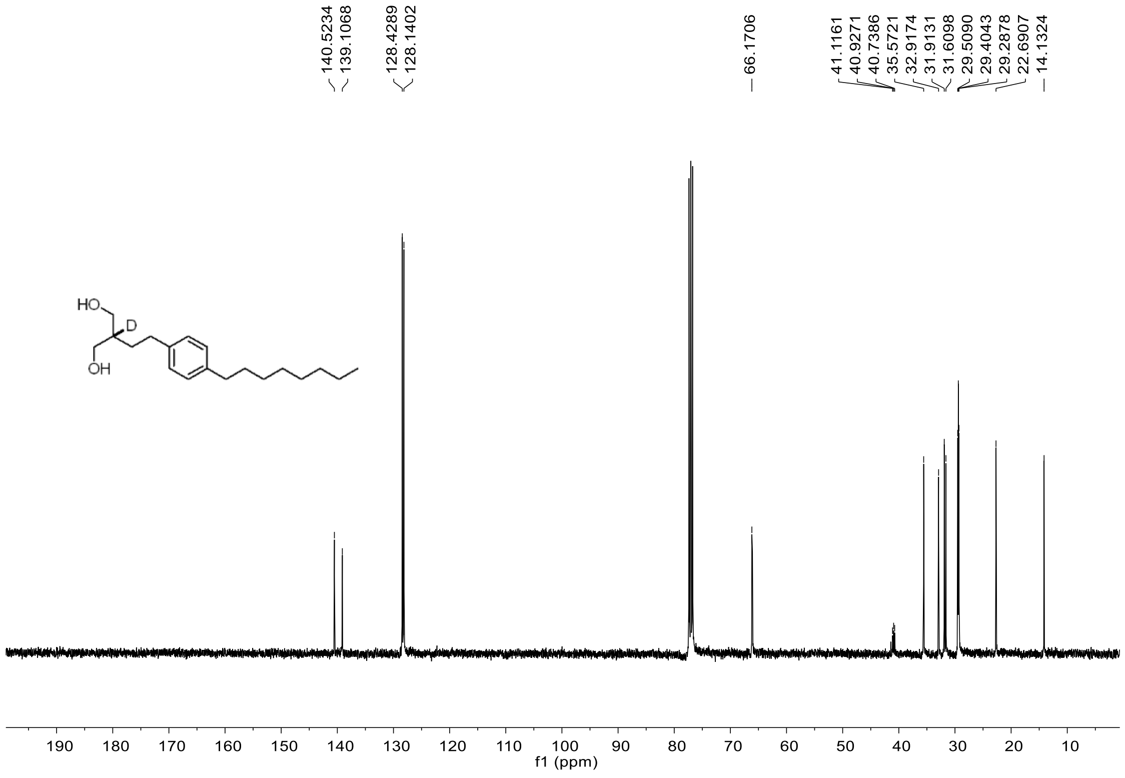

**33b:** $^{1}$H NMR (400 MHz, DMSO-$d_6$)

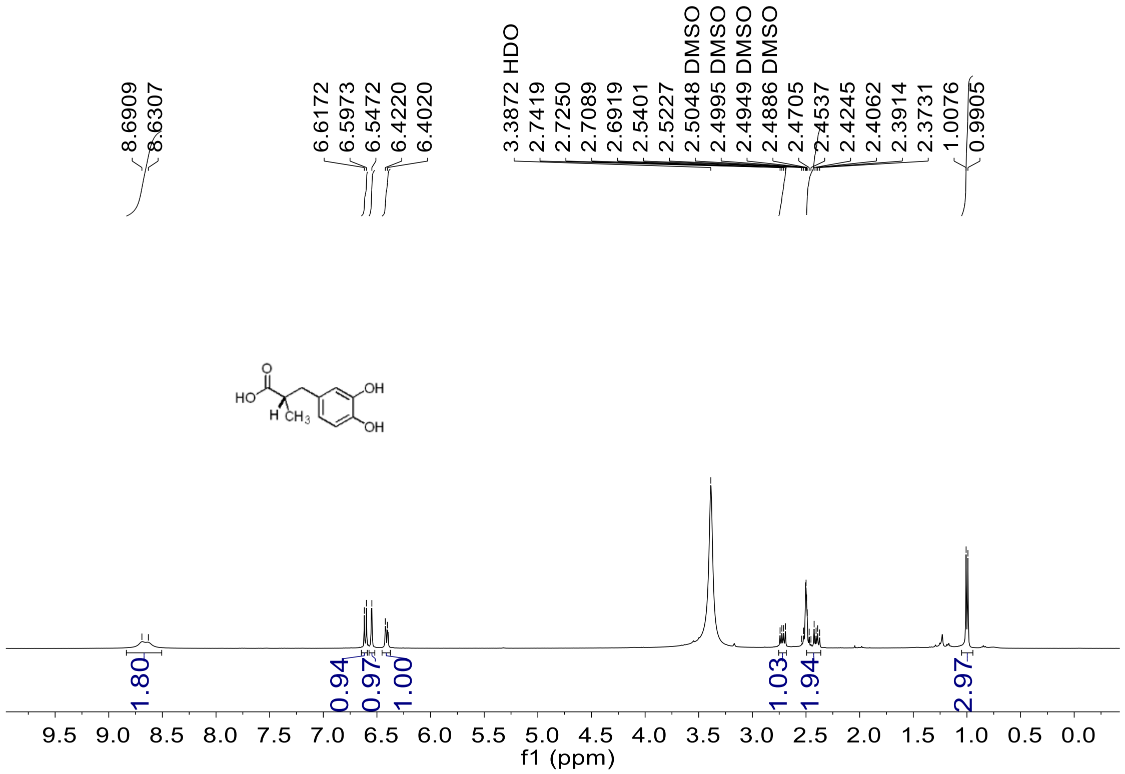


**33b:** $^{13}$C NMR (101 MHz, DMSO-$d_6$)

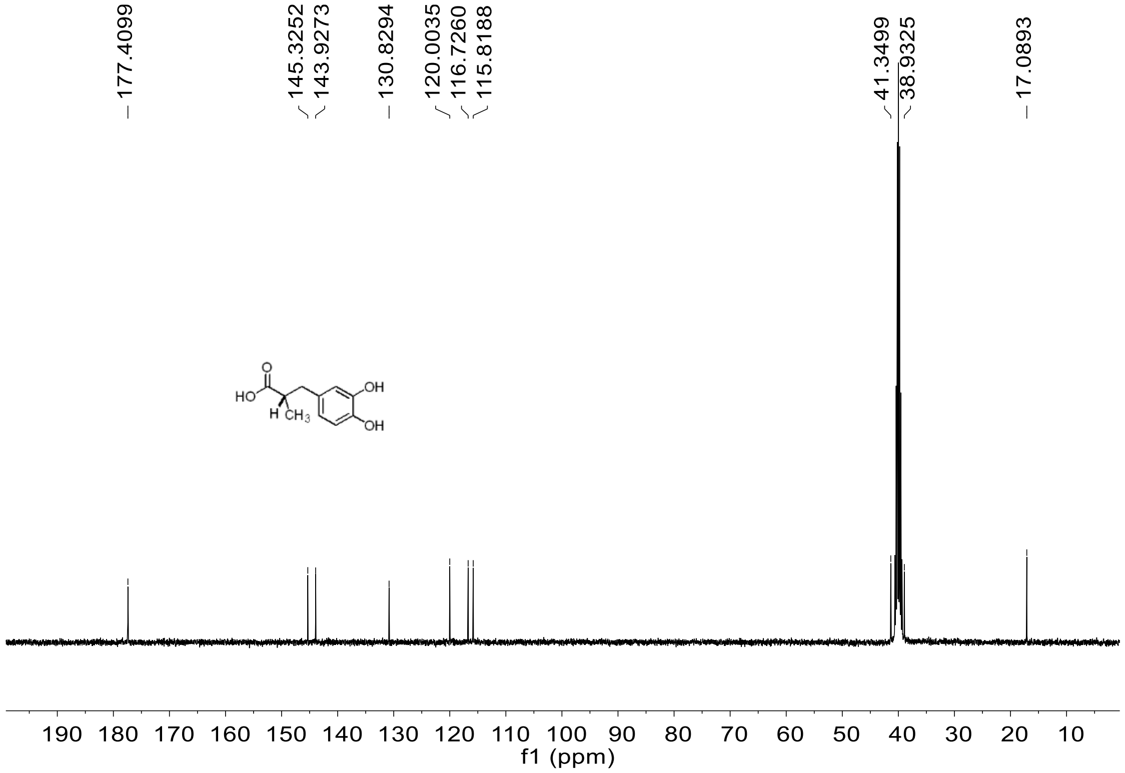

**33c:** $^{1}$H NMR (400 MHz, DMSO-$d_6$)

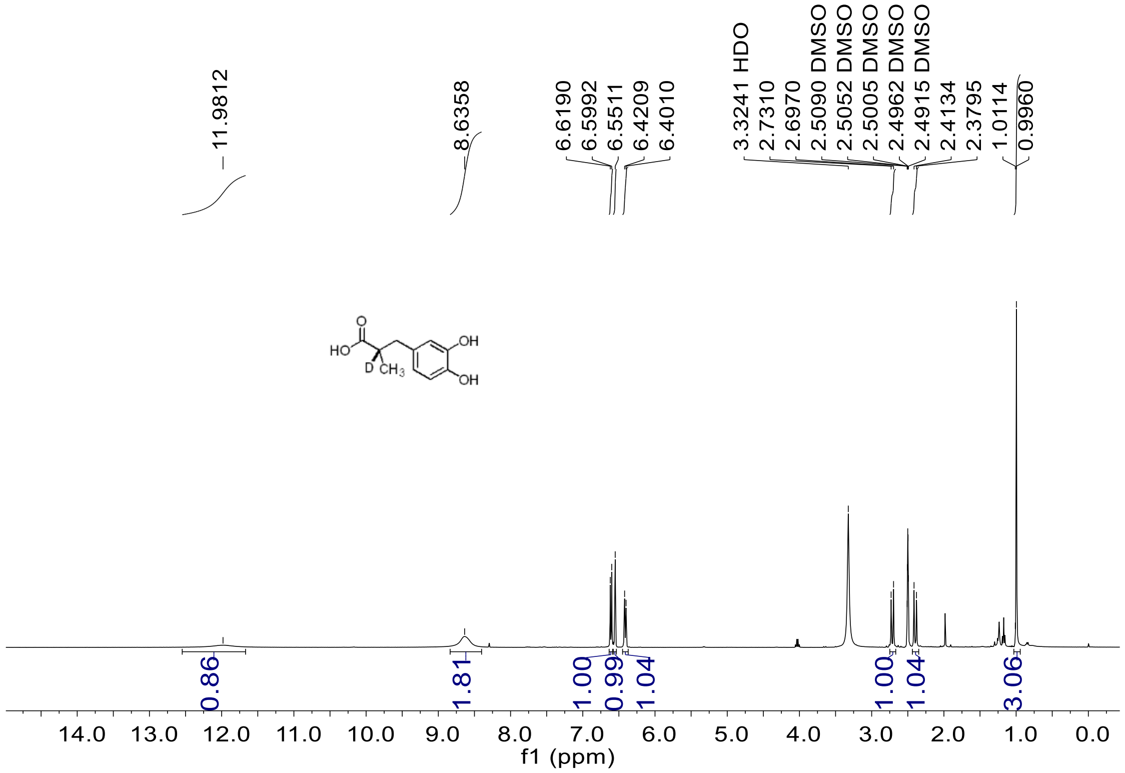


**33c:** $^{13}$C NMR (101 MHz, DMSO-$d_6$)

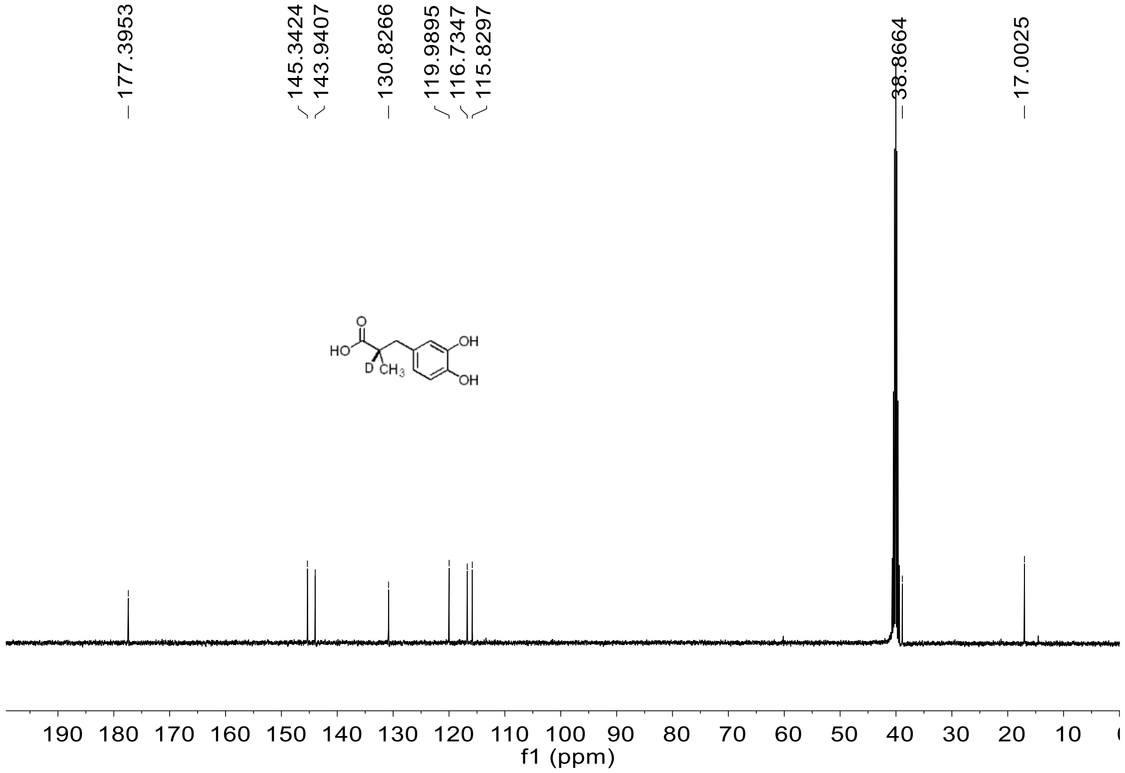

**34b**:$^{1}$H NMR (400 MHz, Chloroform-*d*)

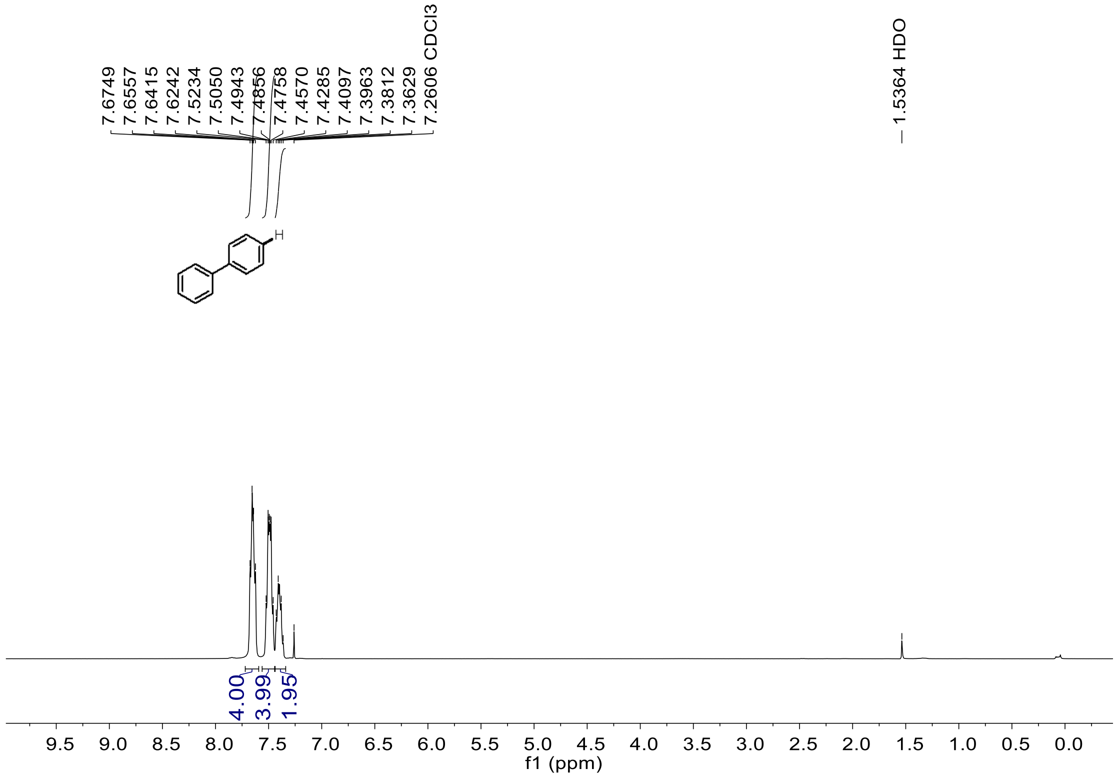


**34b**:$^{13}$C NMR (101 MHz, Chloroform-*d*)

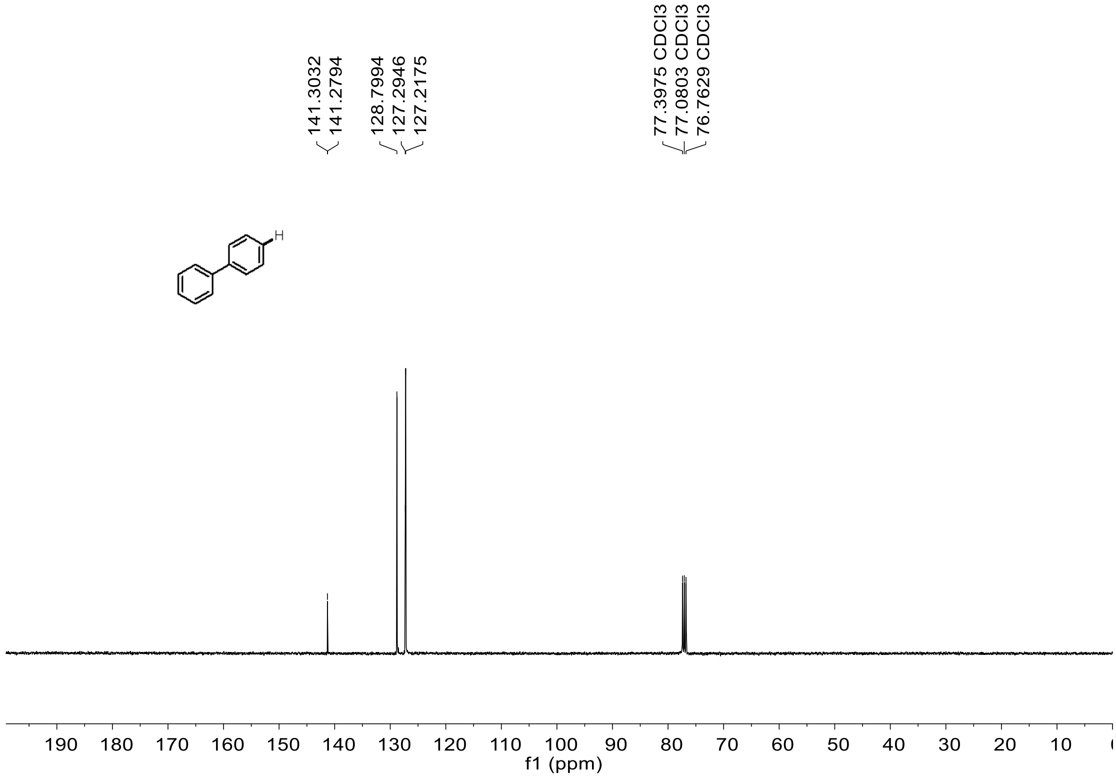

**34c**:¹H NMR (400 MHz, Chloroform-*d*)

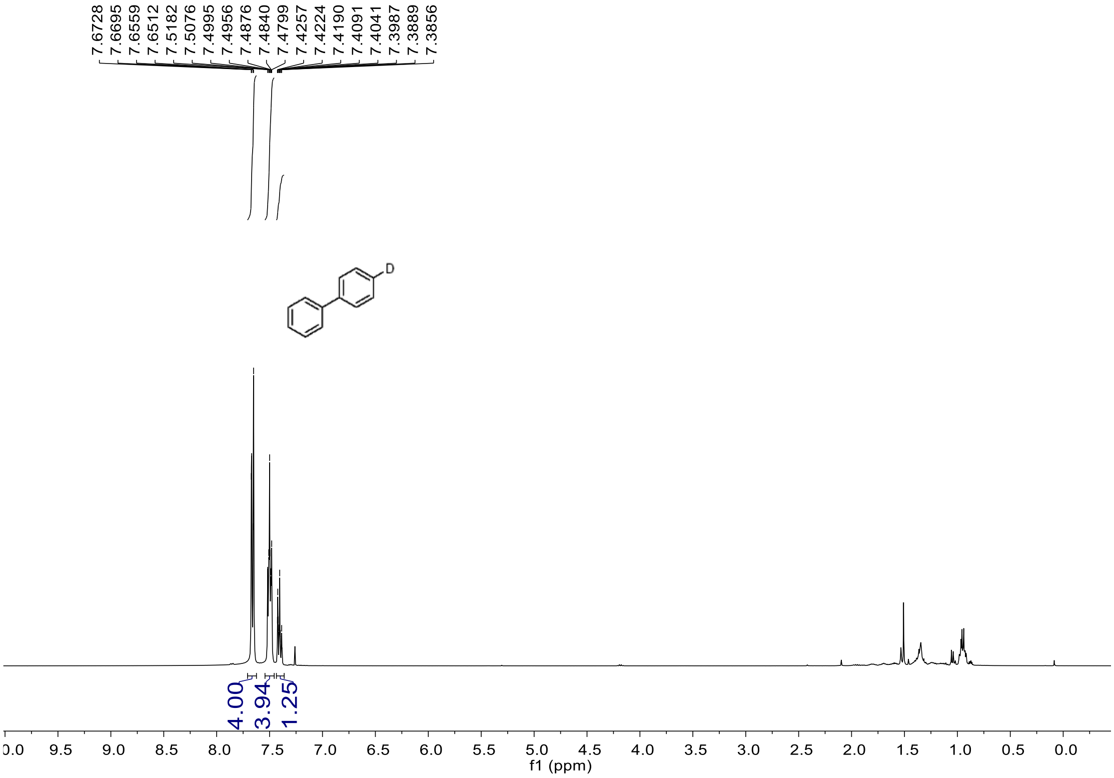


**34c**:¹³C NMR (101 MHz, Chloroform-*d*)

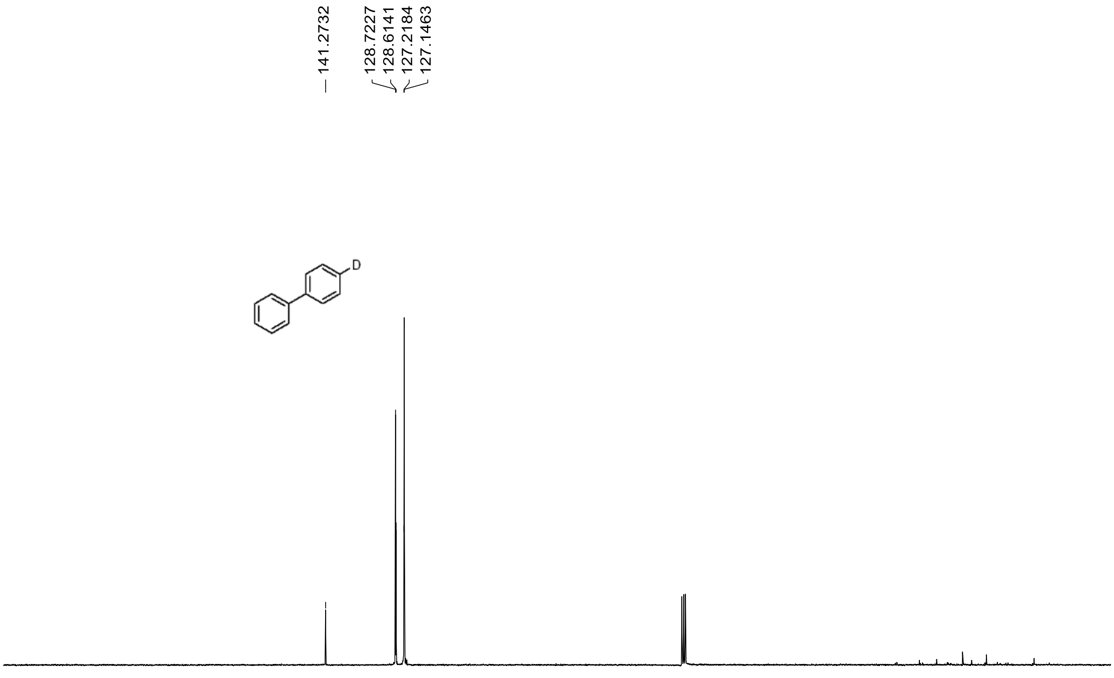

**35b**: $^{1}$H NMR (400 MHz, Chloroform-*d*)

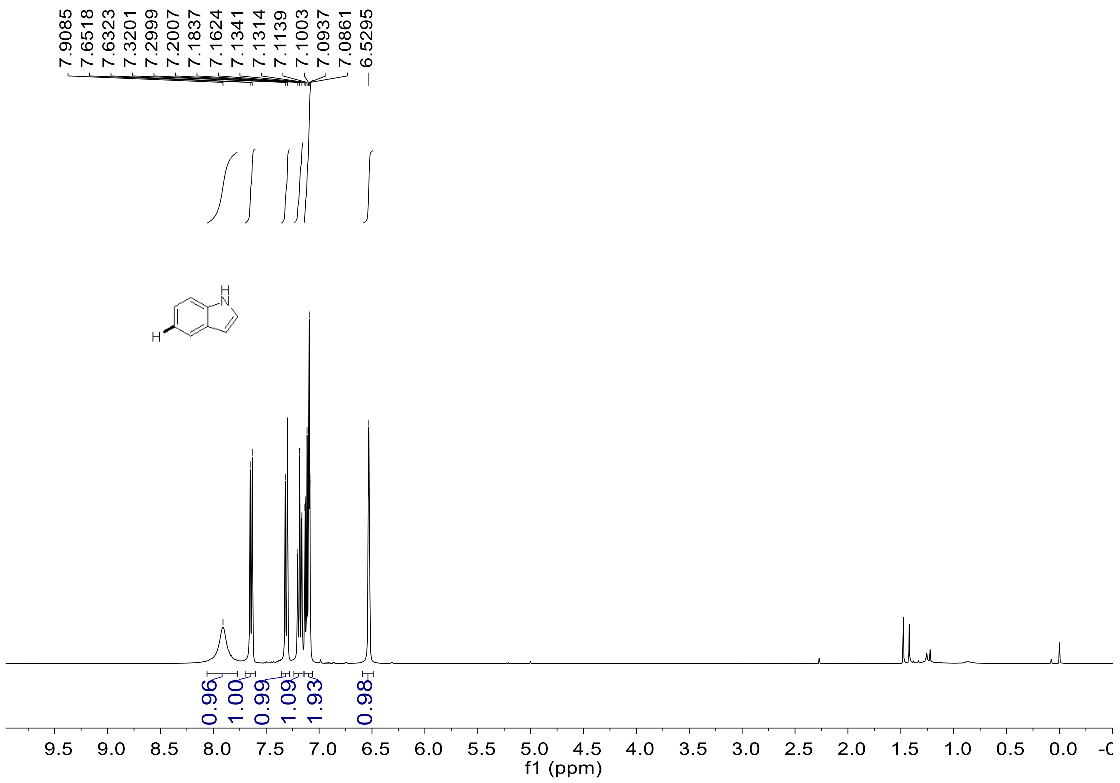


**35b**:$^{13}$C NMR (101 MHz, Chloroform-*d*)

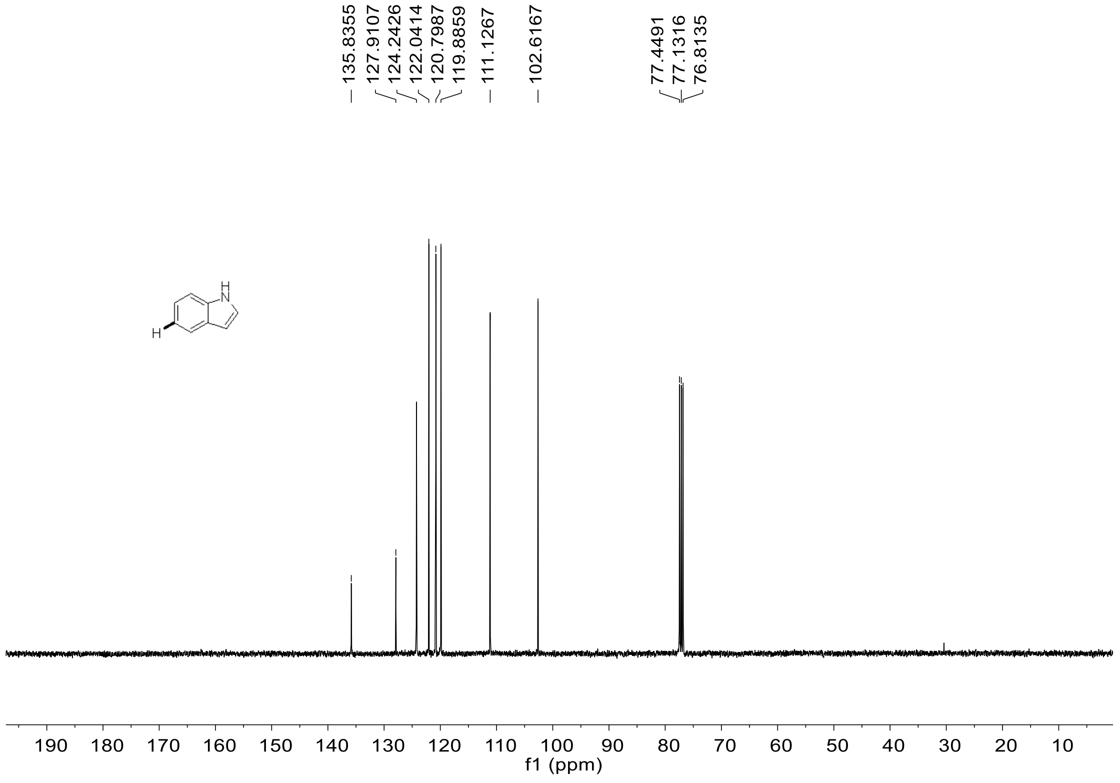

**35c**: $^{1}$H NMR (400 MHz, Chloroform-*d*)

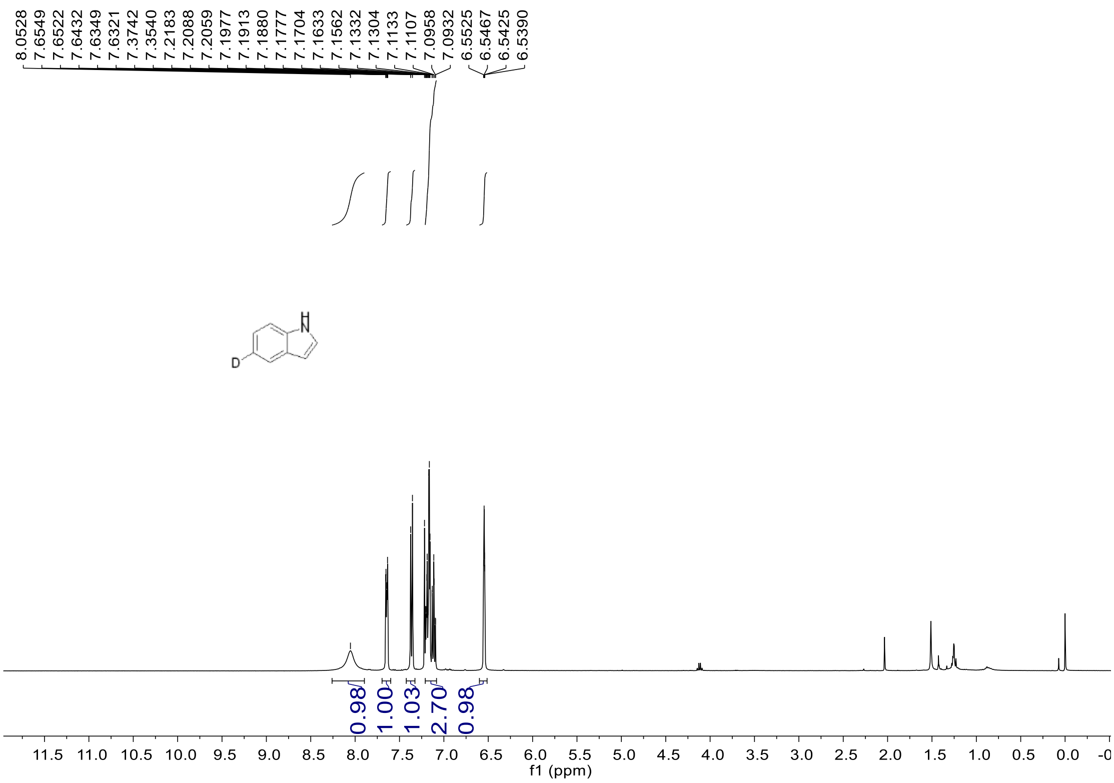


**35c**:$^{13}$C NMR (101 MHz, Chloroform-*d*)

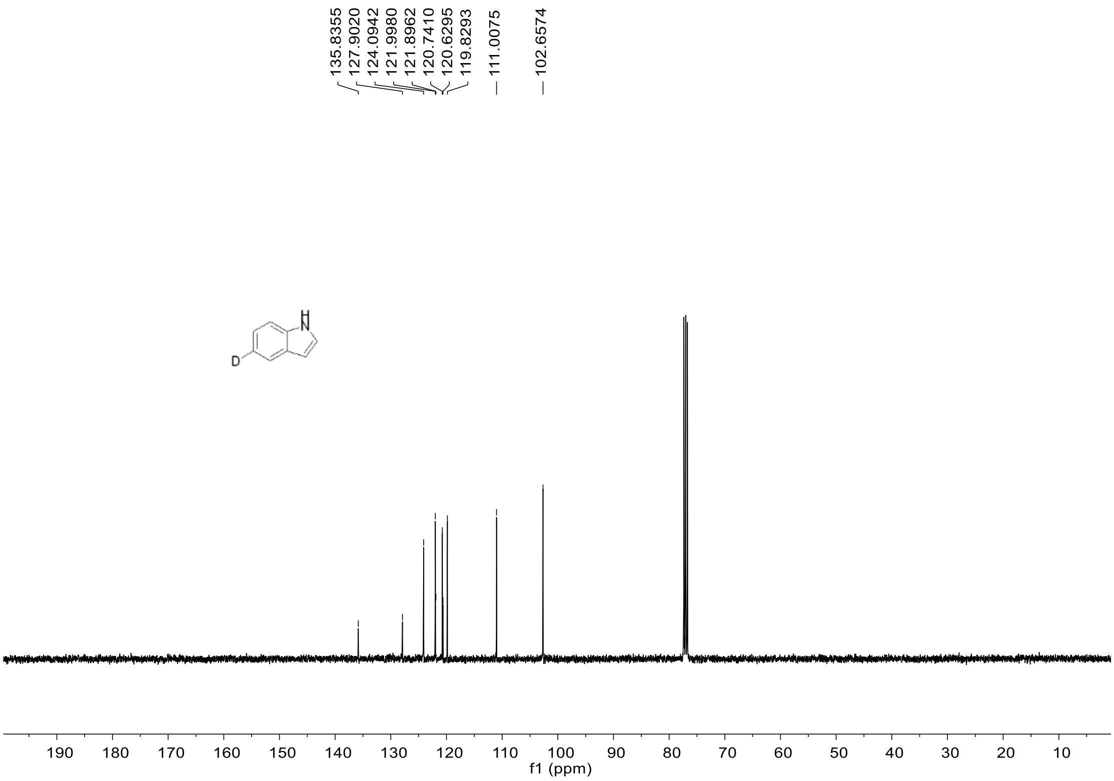

**36b**:$^{1}$H NMR (400 MHz, Chloroform-*d*)

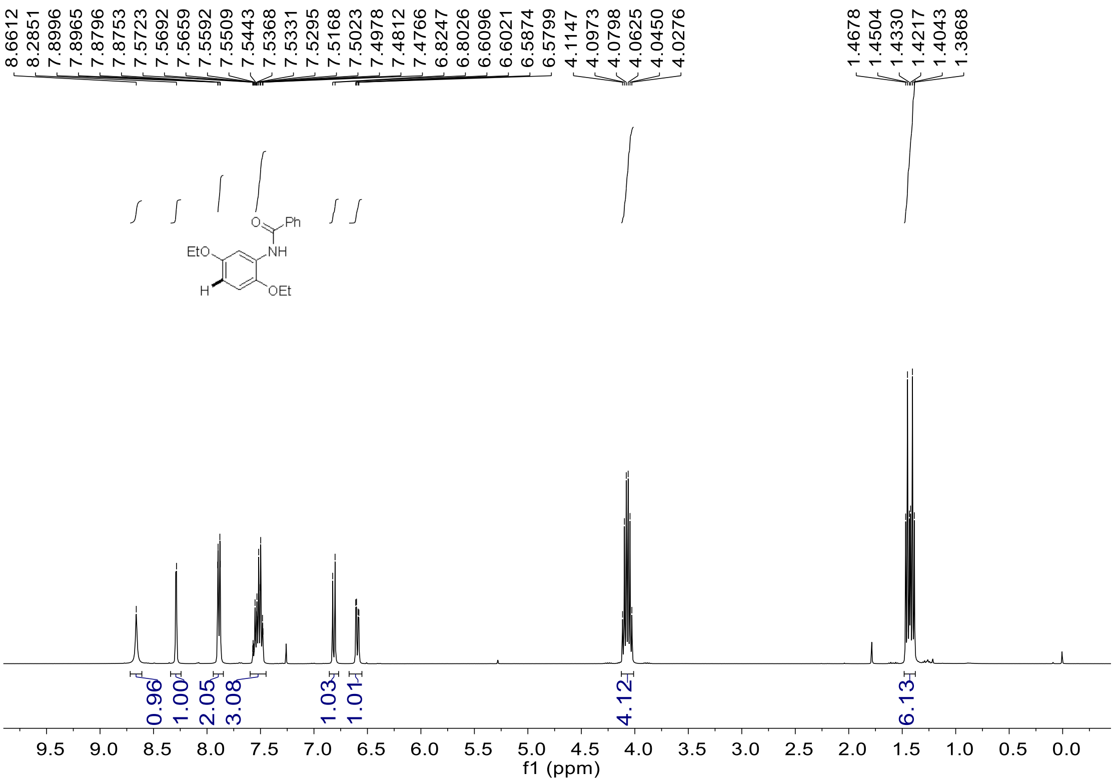


**36b** :$^{13}$C NMR (101 MHz, Chloroform-*d*)

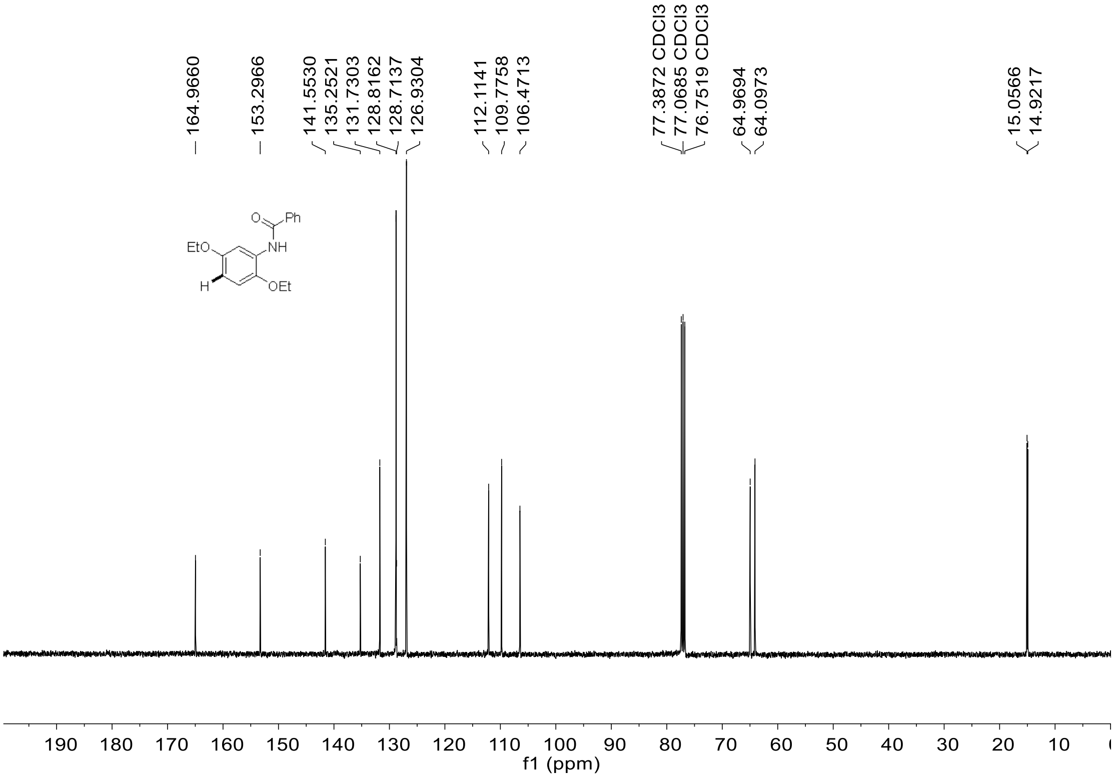

36c:$^{1}$H NMR (400 MHz, Chloroform-*d*)

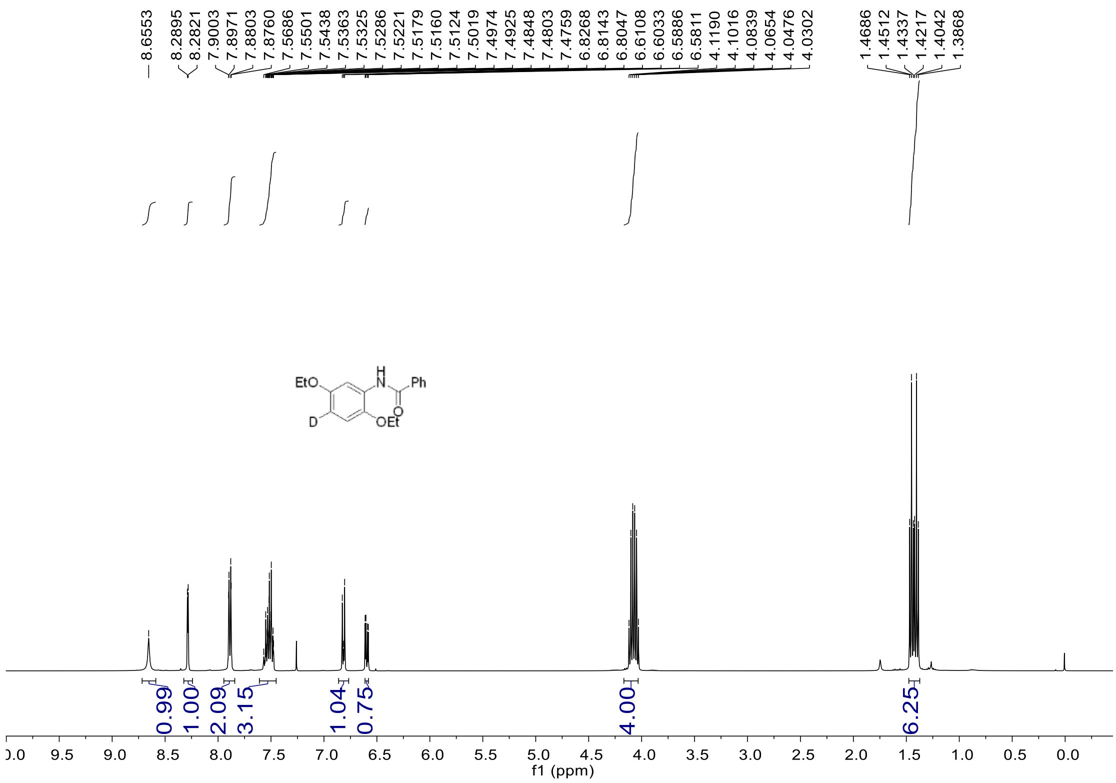


36c :$^{13}$C NMR (101 MHz, Chloroform-*d*)

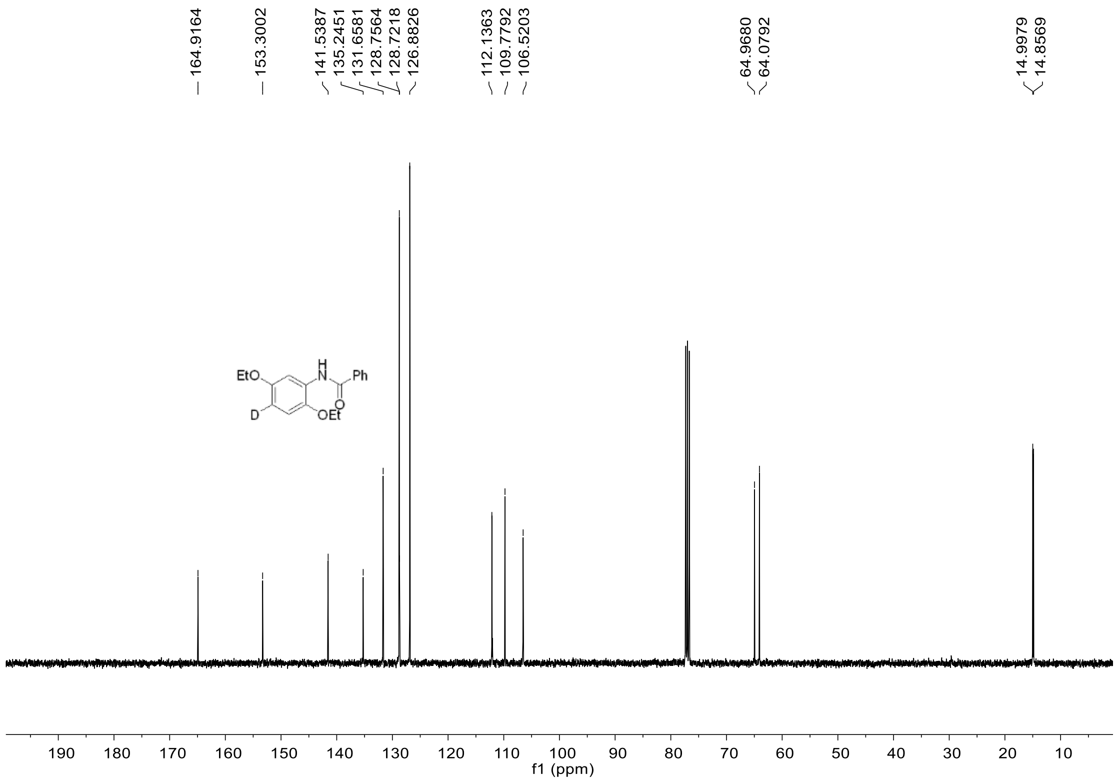

**37b** :$^{1}$H NMR (400 MHz, Chloroform-*d*)

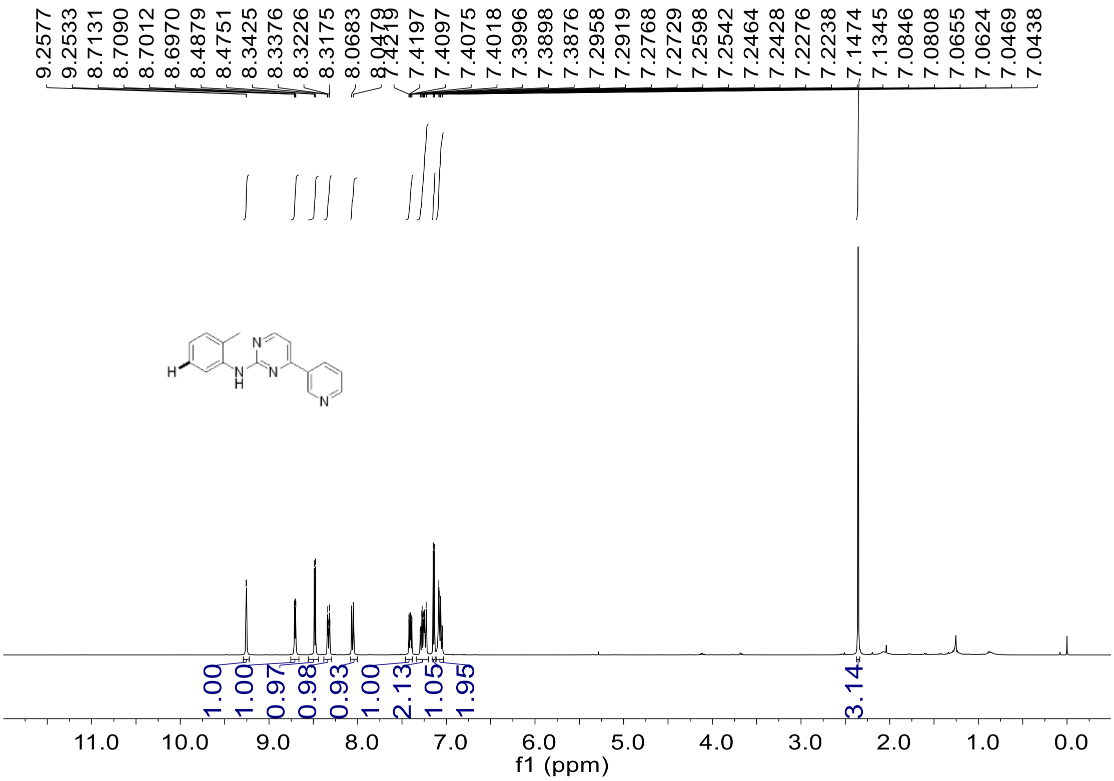


**37b** :$^{13}$C NMR (101 MHz, Chloroform-*d*)

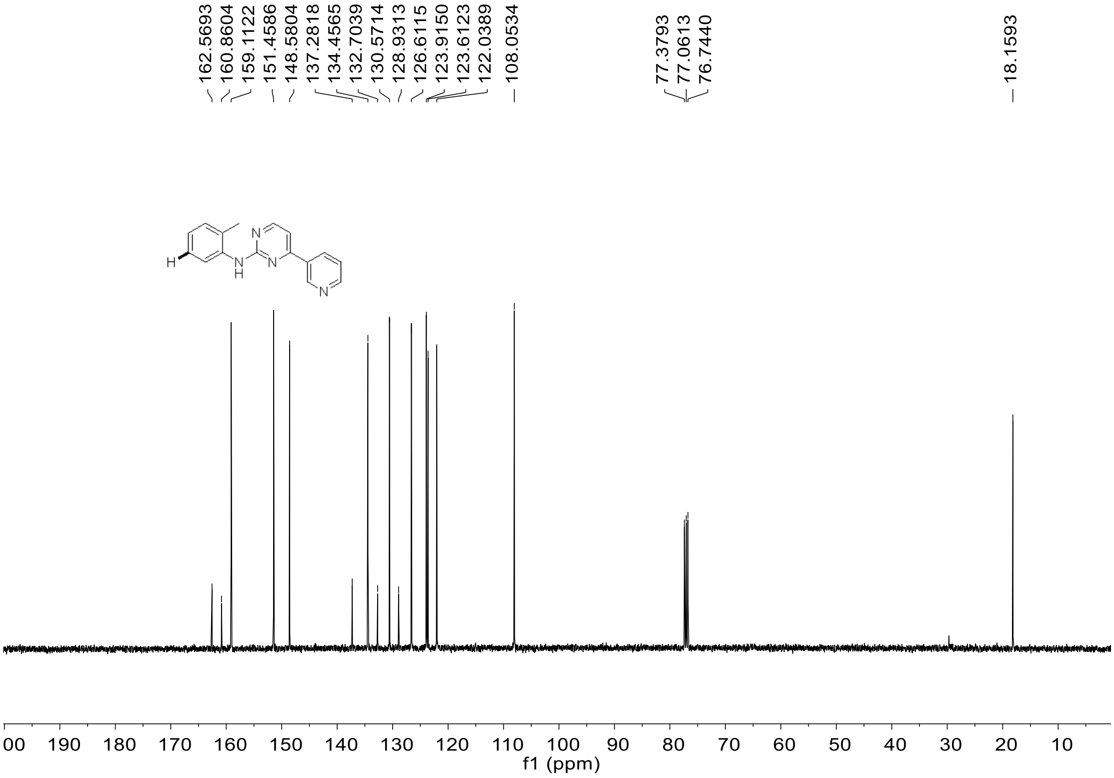

**37c** :$^{1}$H NMR (400 MHz, Chloroform-*d*)

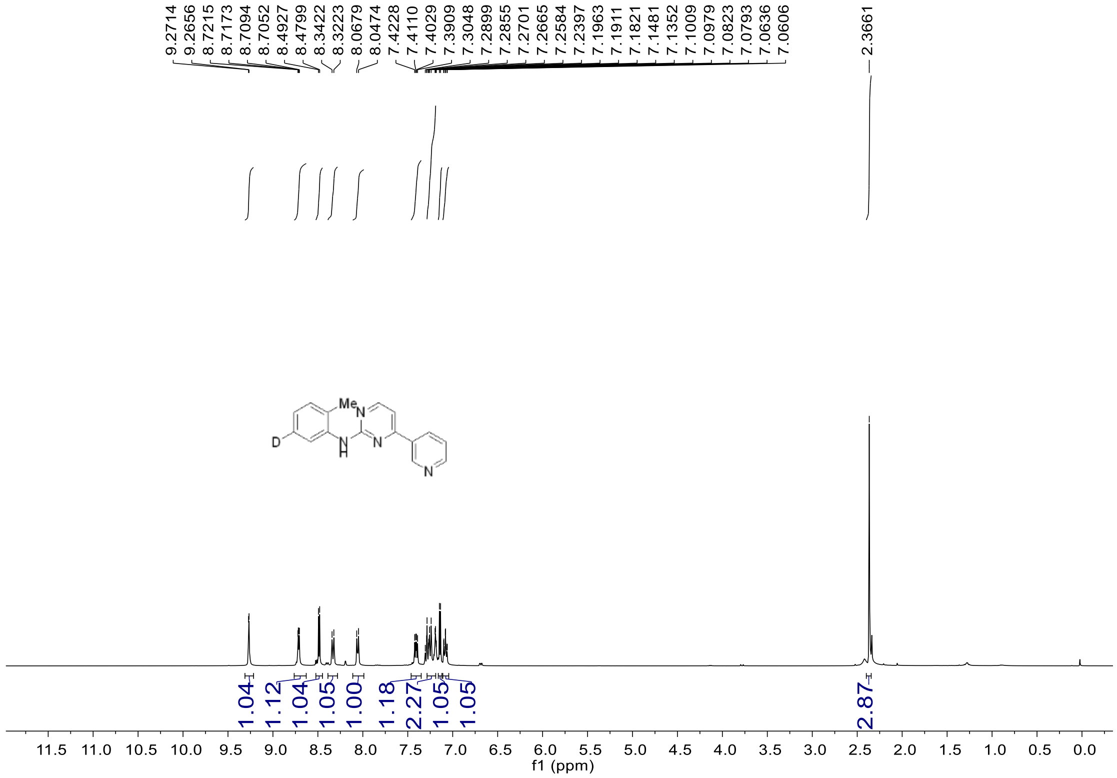


**37c** :$^{13}$C NMR (101 MHz, Chloroform-*d*)

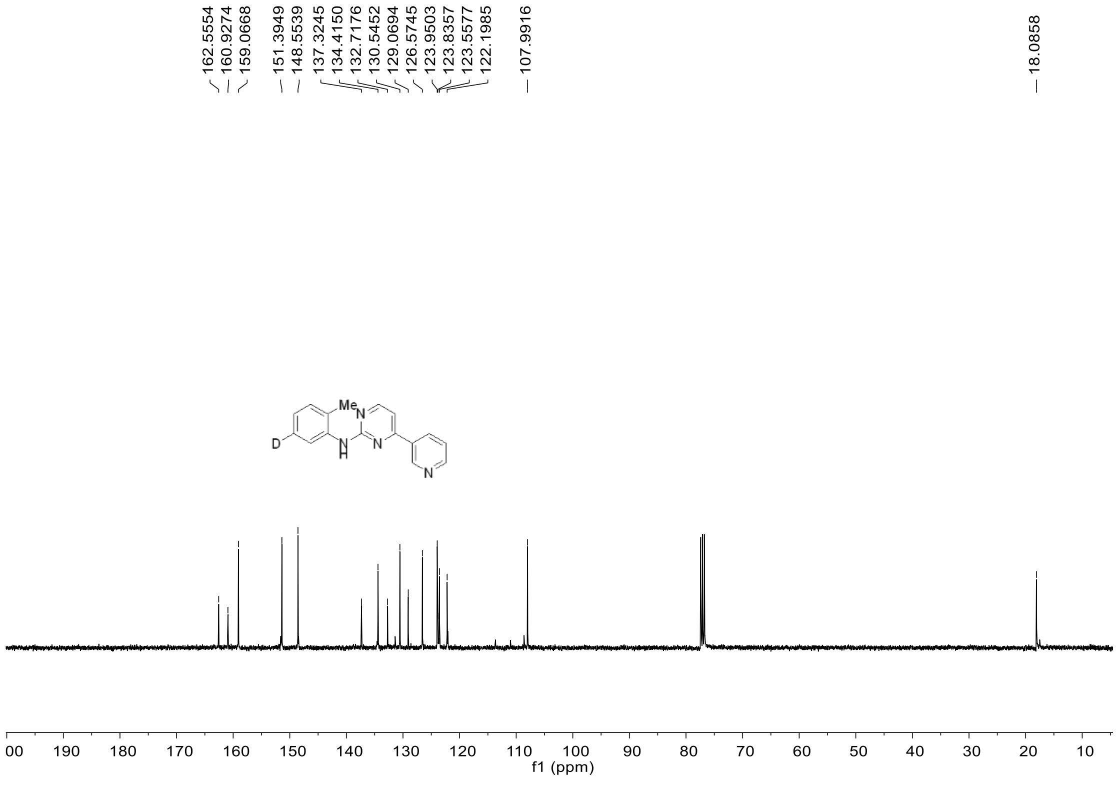

**38b:** $^{1}$H NMR (400 MHz, Deuterium Oxide)

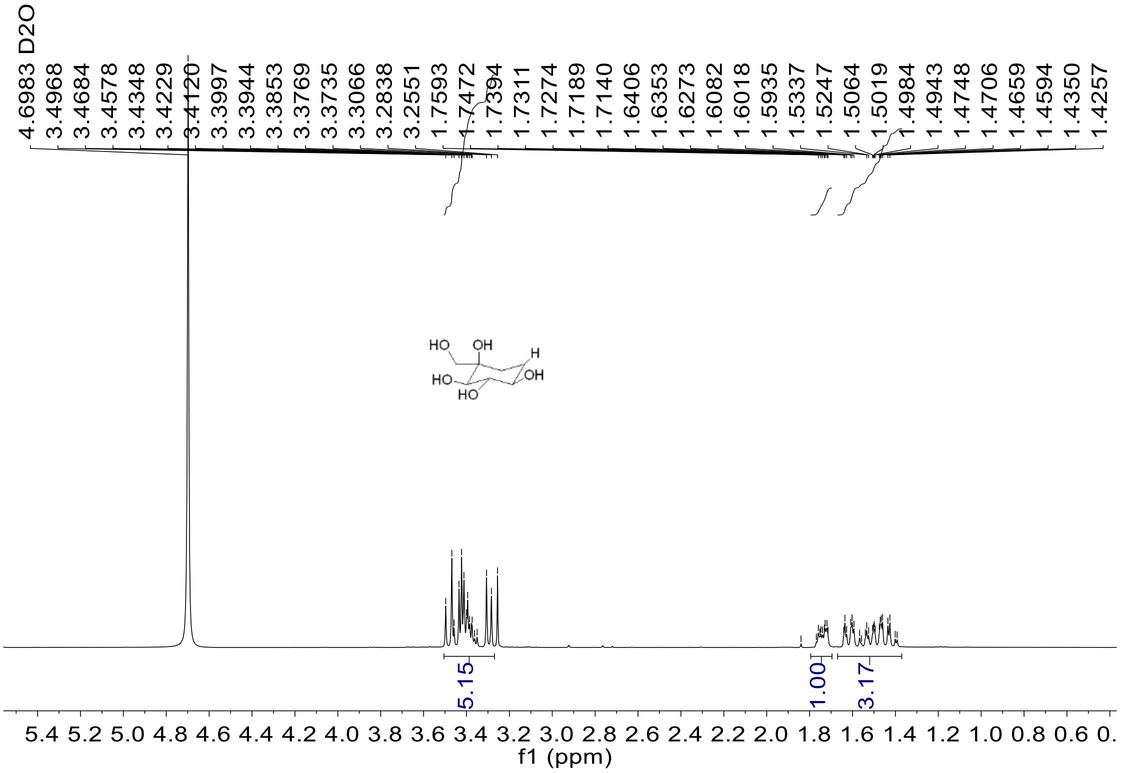


**38b:** $^{13}$C NMR (101 MHz, Deuterium Oxide)

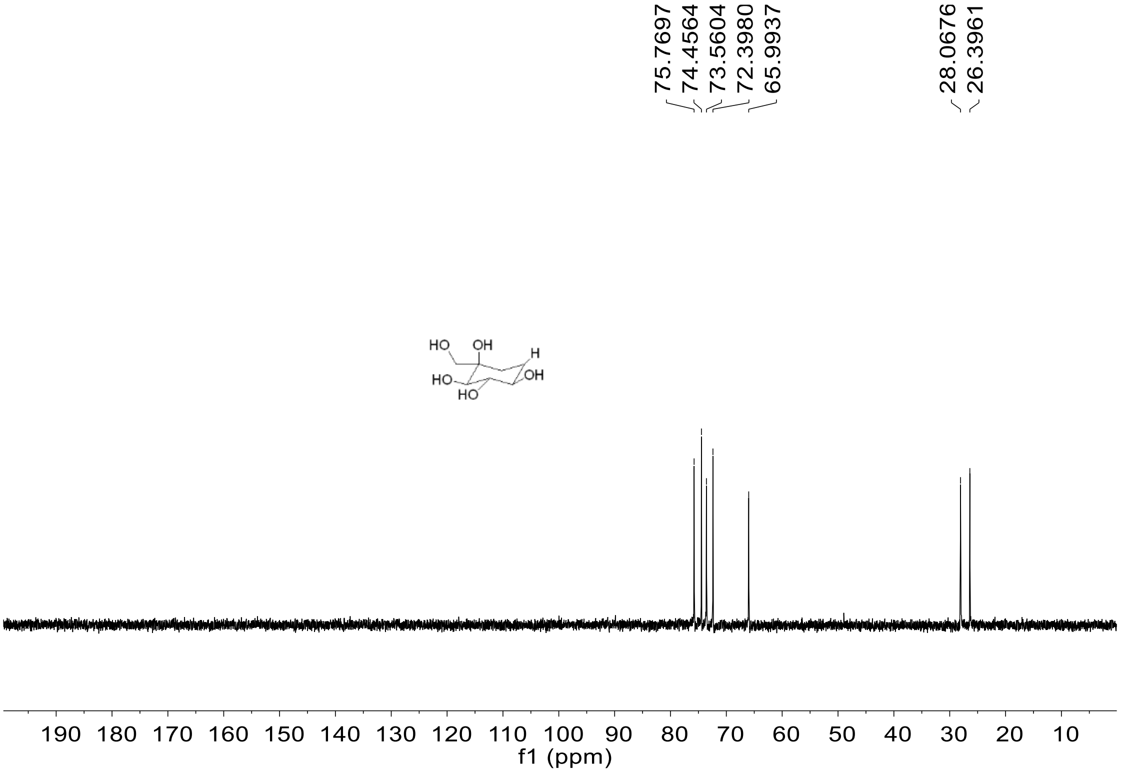

**38c:** $^{1}$H NMR (400 MHz, Deuterium Oxide)

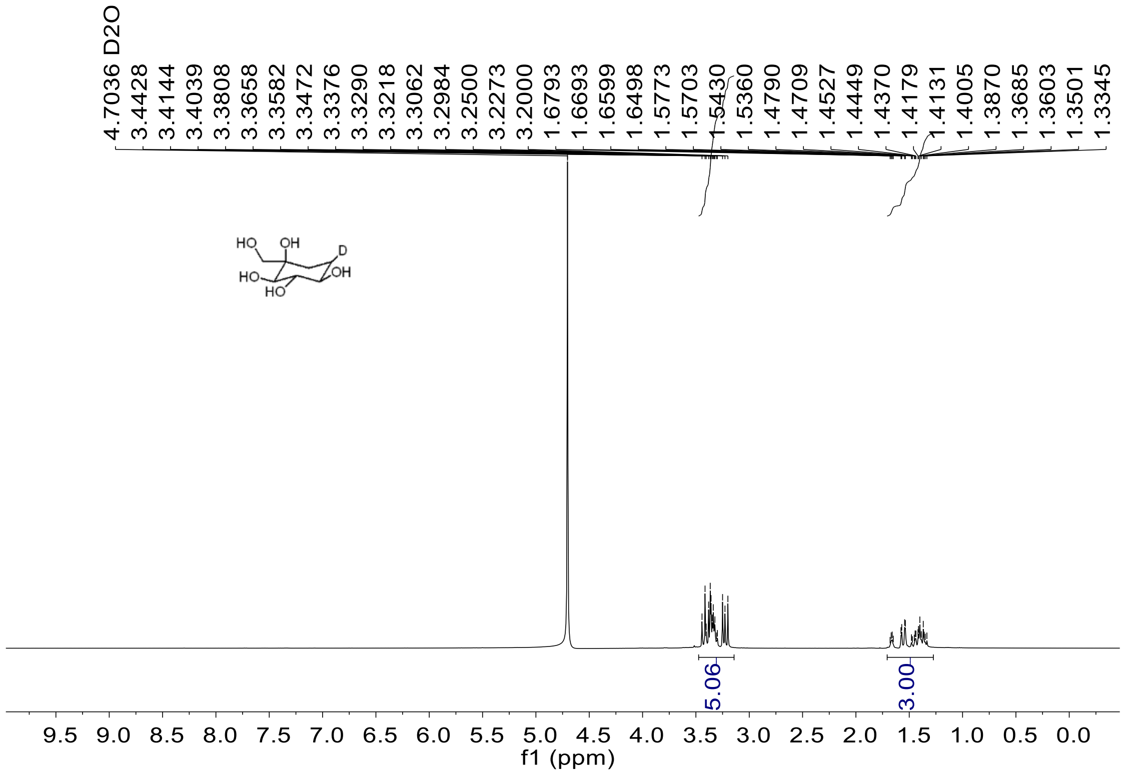


**38c:** $^{13}$C NMR (101 MHz, Deuterium Oxide)

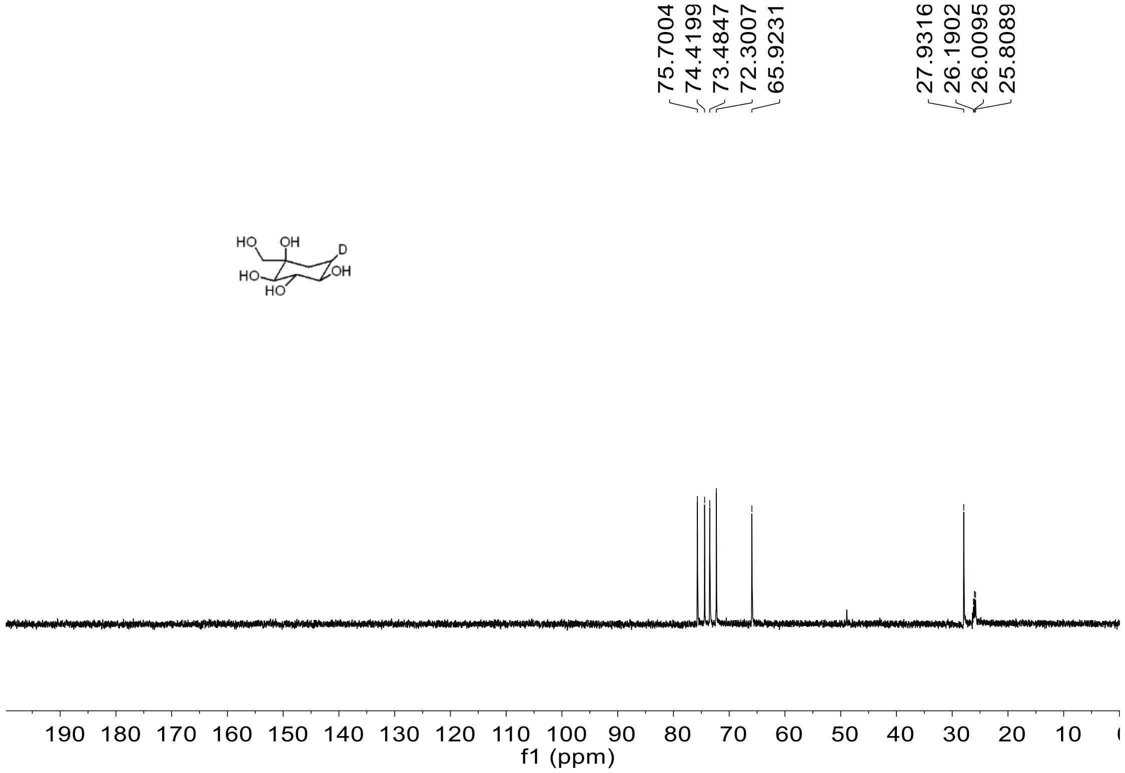

**39b:** $^{1}$H NMR (400 MHz, Chloroform-*d*)

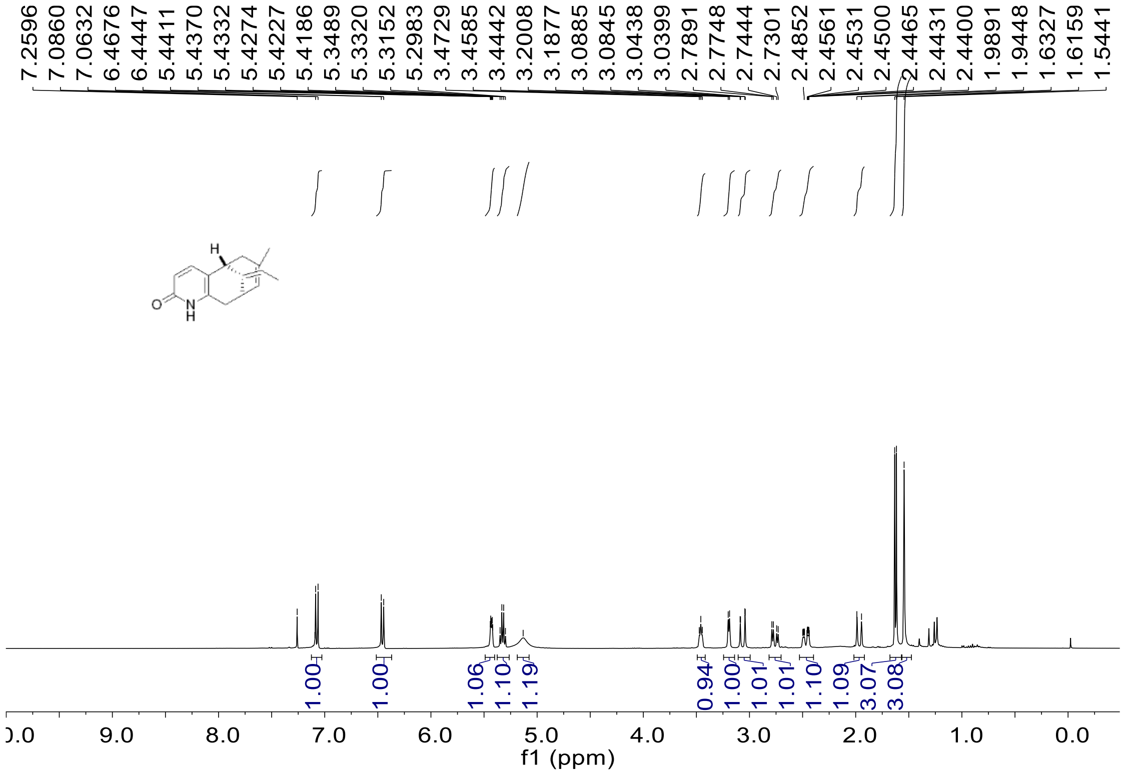


**39b:** $^{13}$C NMR (101 MHz, Chloroform-*d*)

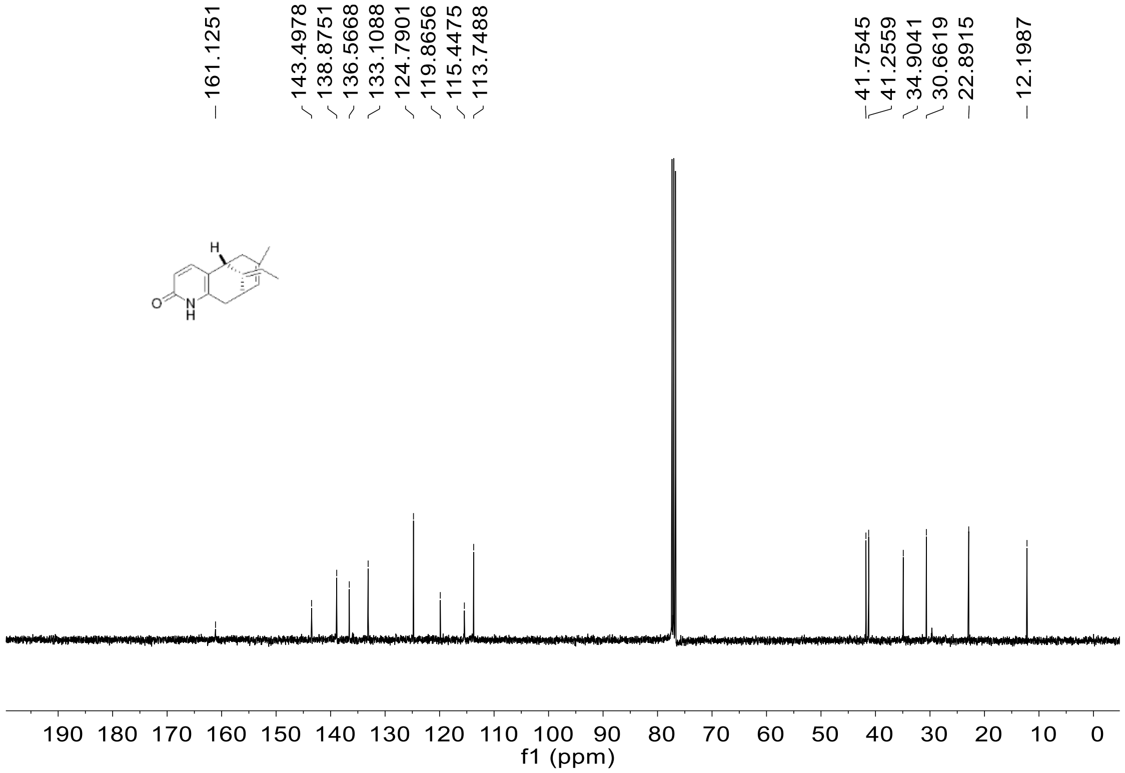

**39c:** $^{1}$H NMR (400 MHz, Chloroform-*d*)

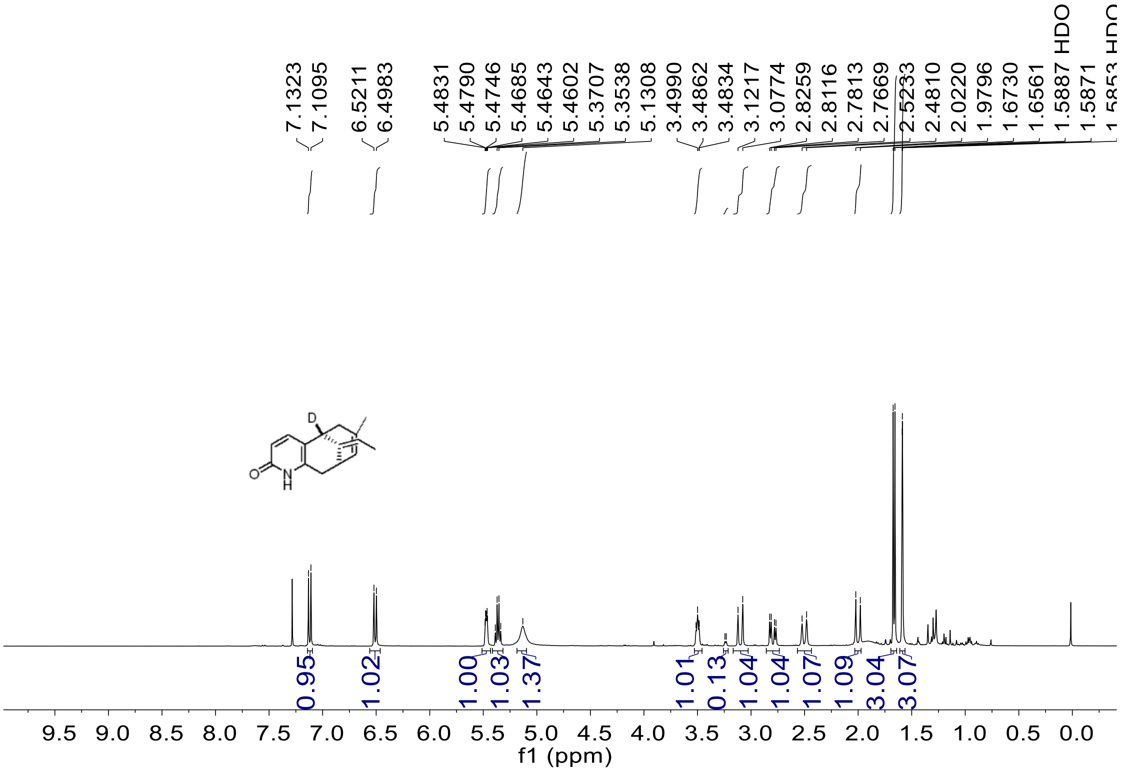


**39c:** $^{13}$C NMR (101 MHz, Chloroform-*d*)

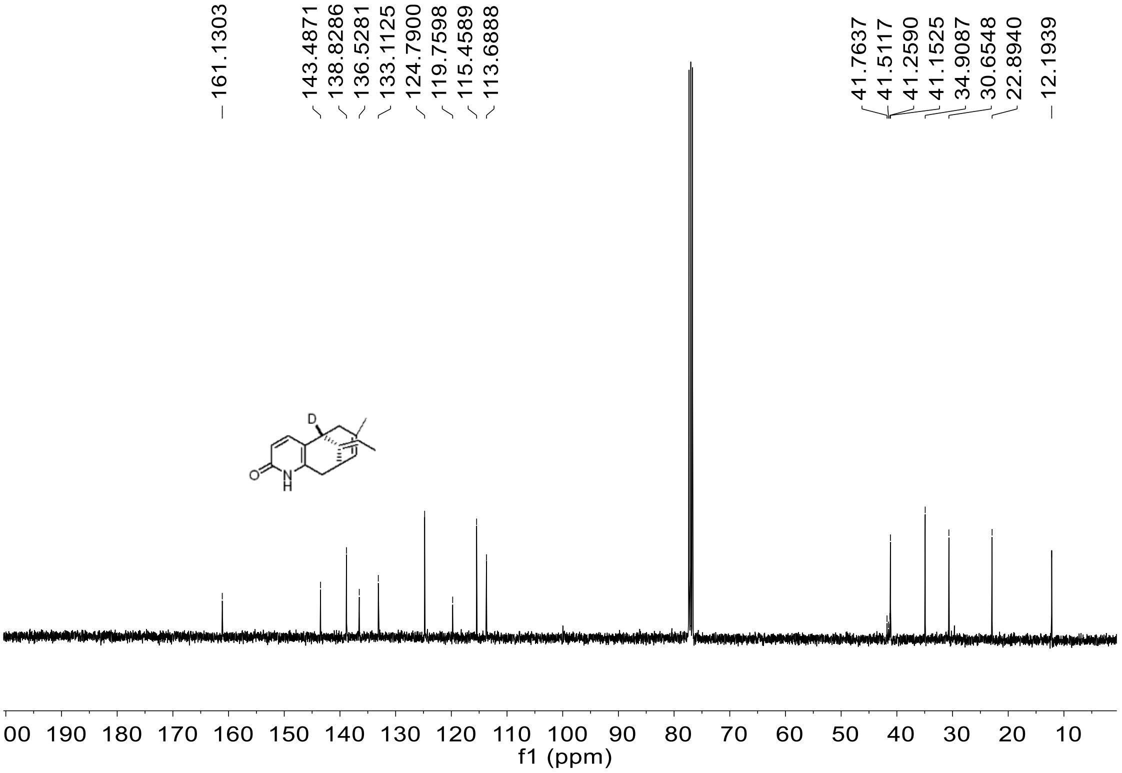

**40b:** $^{1}$H NMR (400 MHz, Chloroform-*d*)

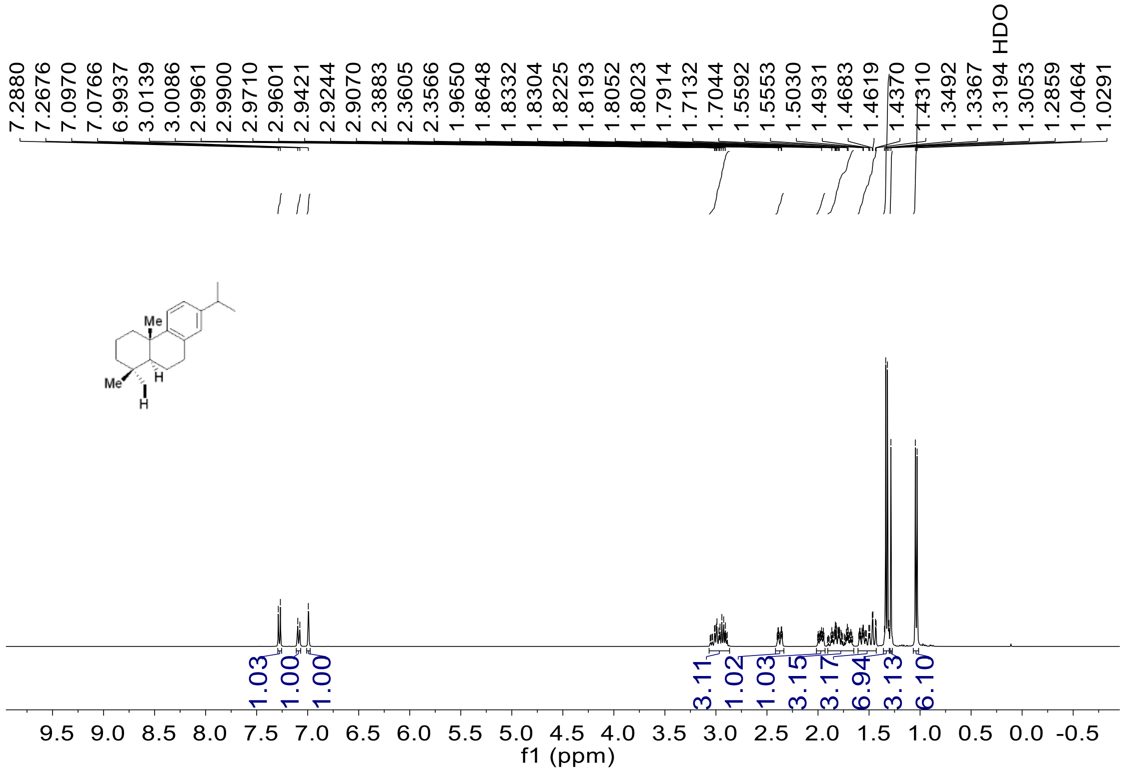


**40b:** $^{13}$C NMR (101 MHz, Chloroform-*d*)

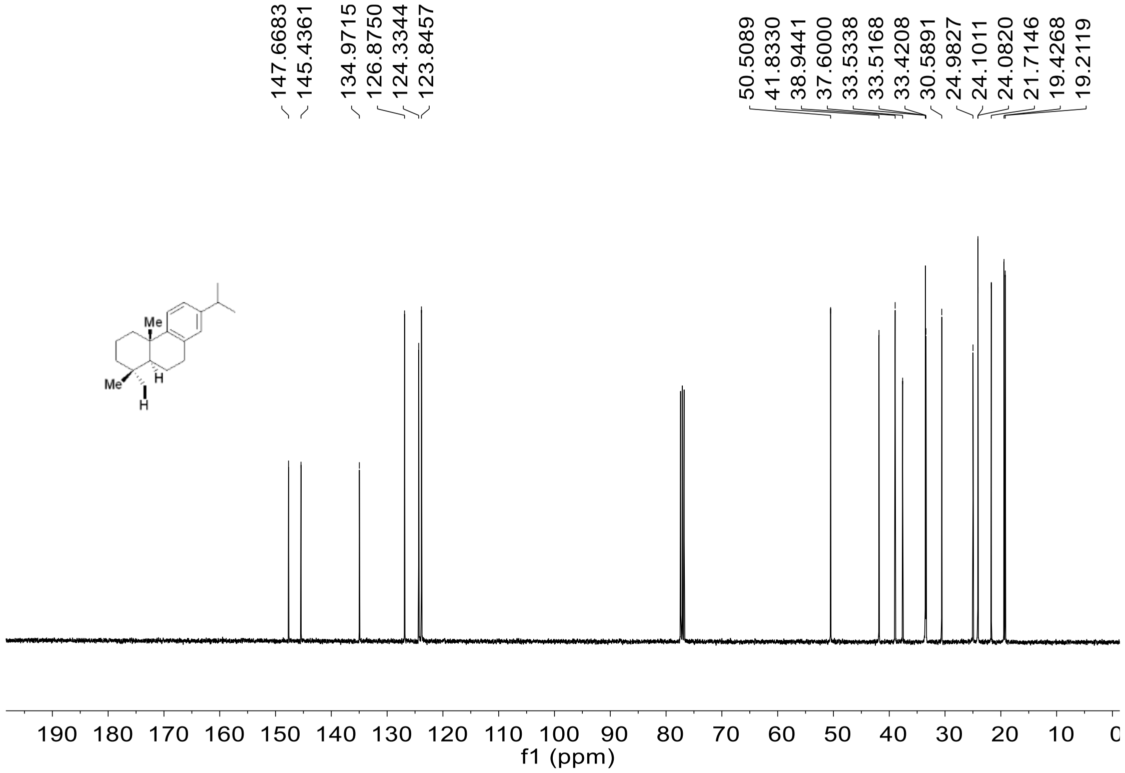

**40c:** $^{1}$H NMR (400 MHz, Chloroform-*d*)

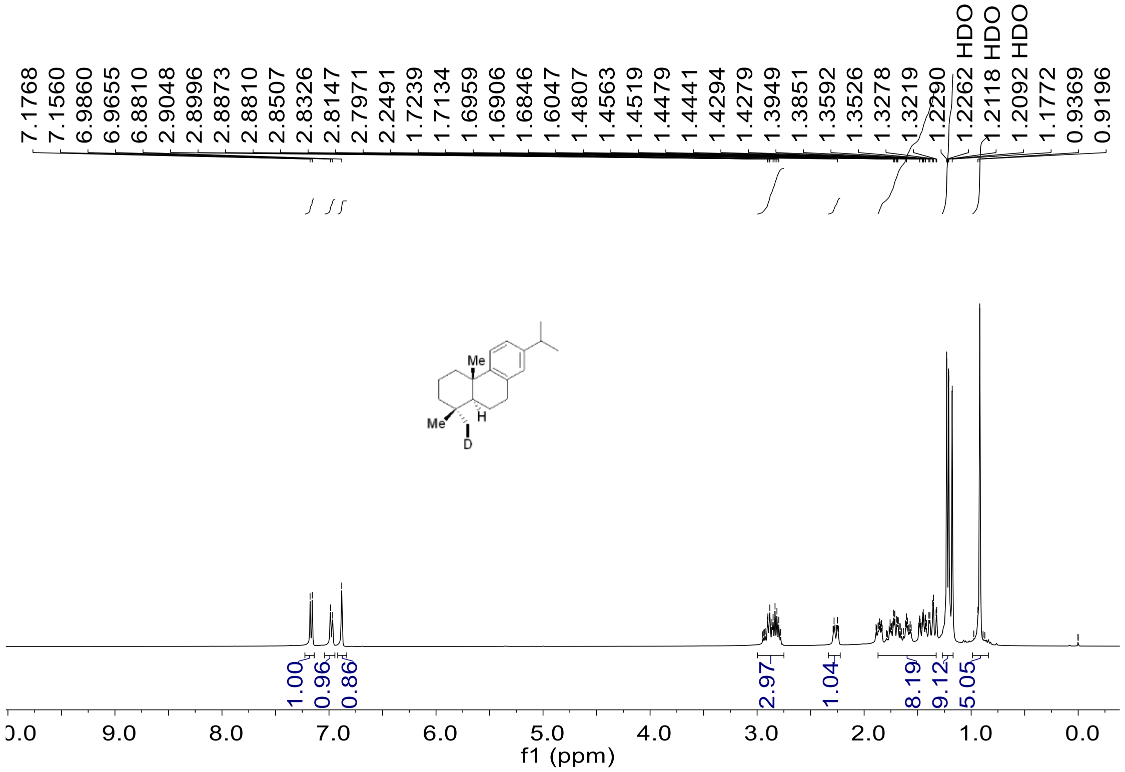


**40c:** $^{13}$C NMR (101 MHz, Chloroform-*d*)

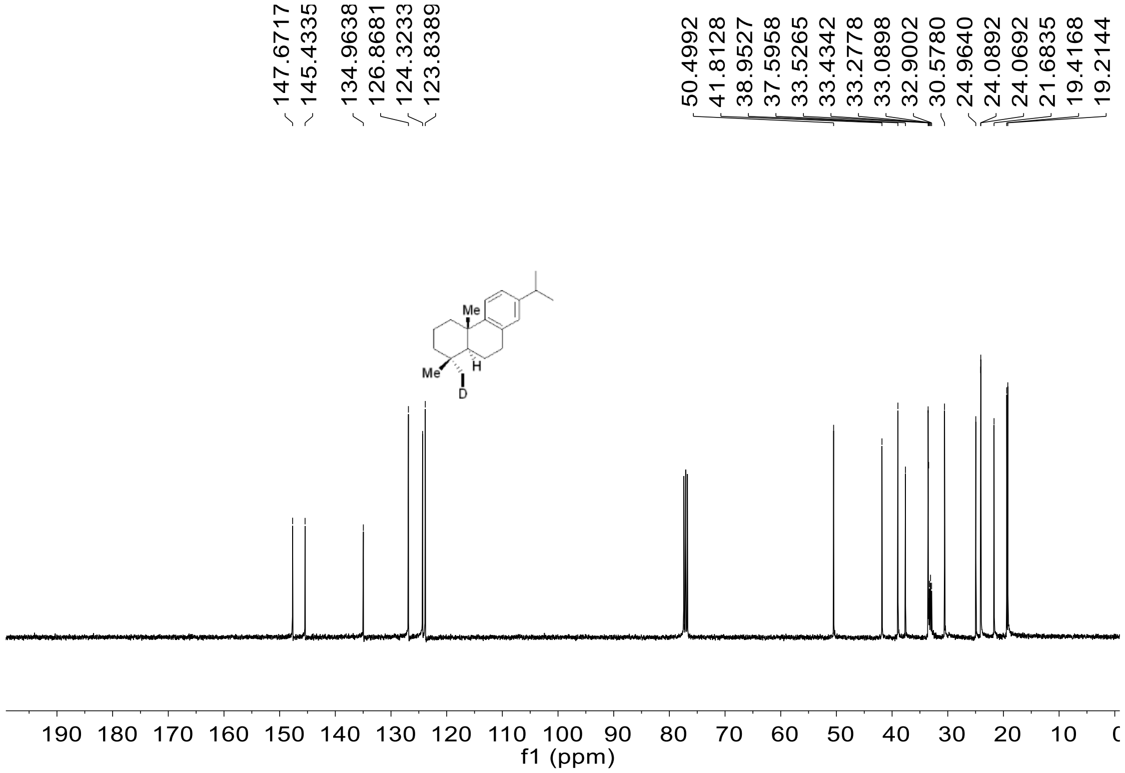

**41e:** $^{1}$H NMR (400 MHz, Chloroform-*d*)

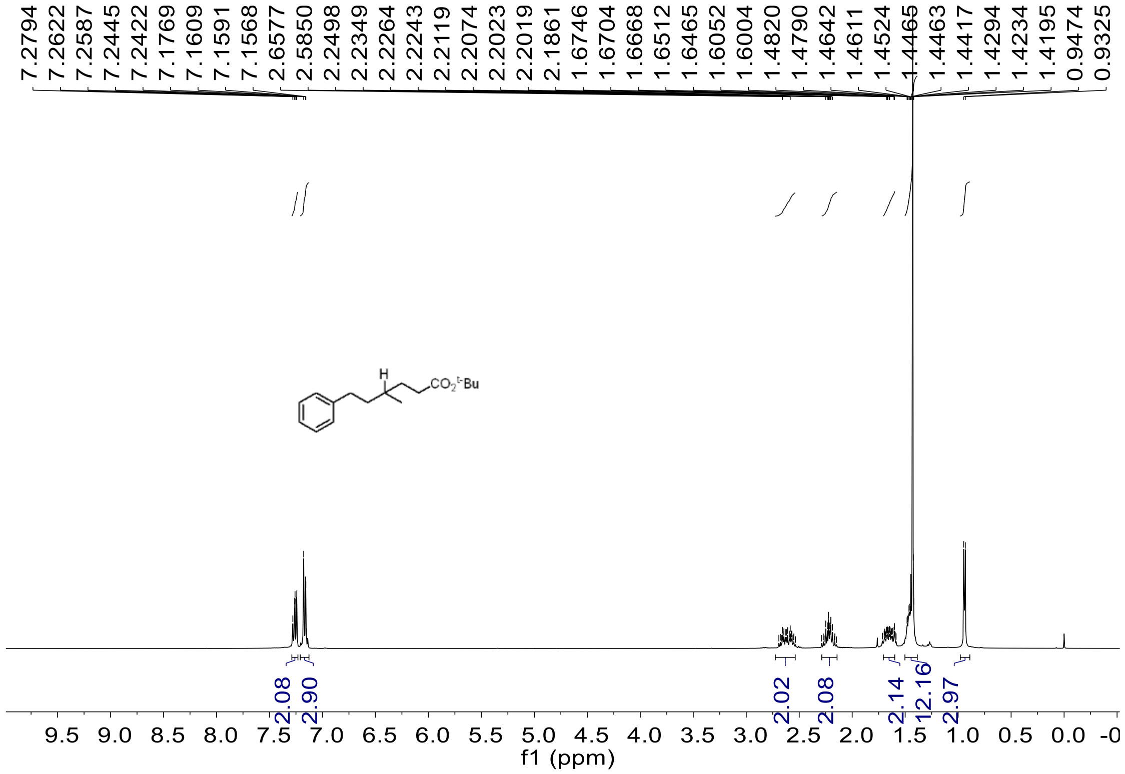


**41e:** $^{13}$C NMR (101 MHz, Chloroform-*d*)

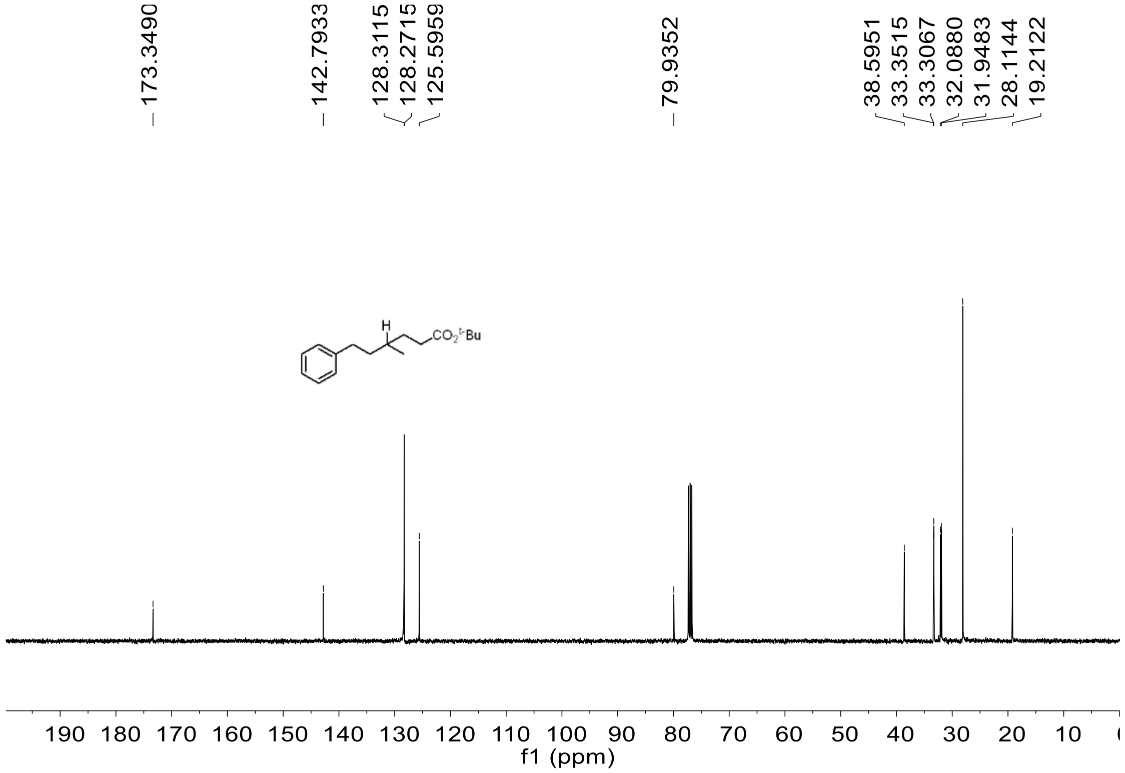

**41f:** $^{1}$H NMR (400 MHz, Chloroform-*d*)

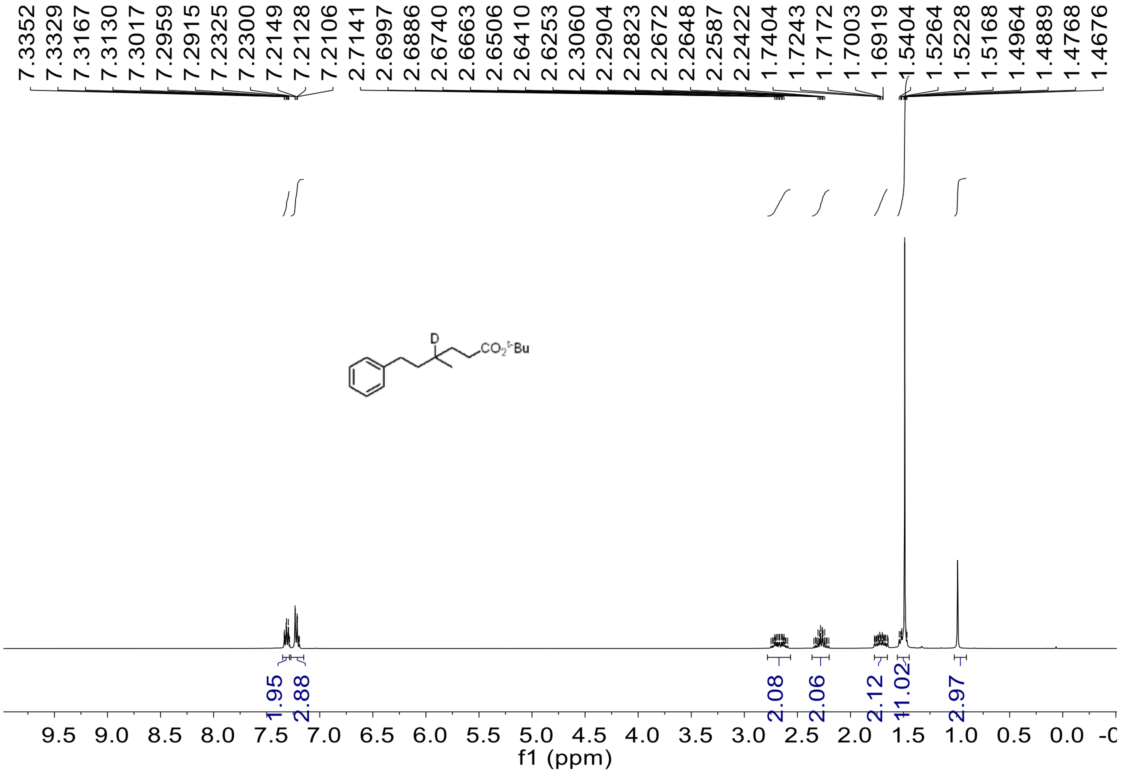


**41f:** $^{13}$C NMR (101 MHz, Chloroform-*d*)

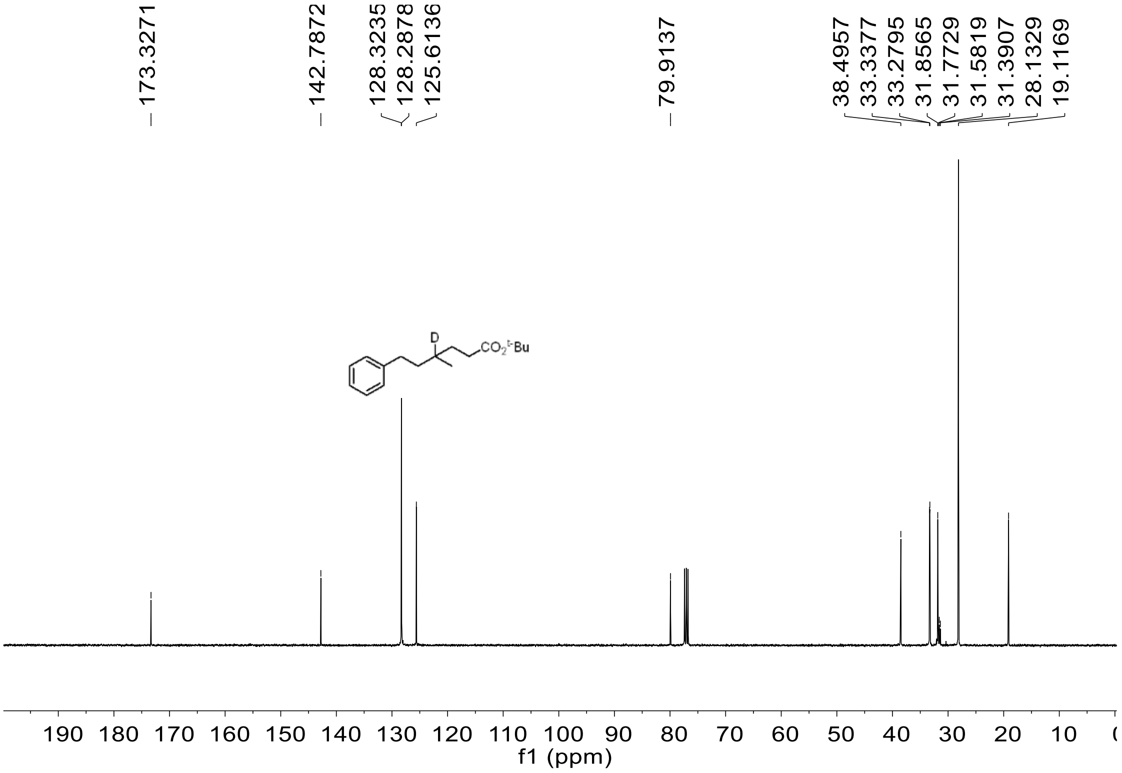

**42e:** $^{1}$H NMR (400 MHz, Chloroform-*d*)

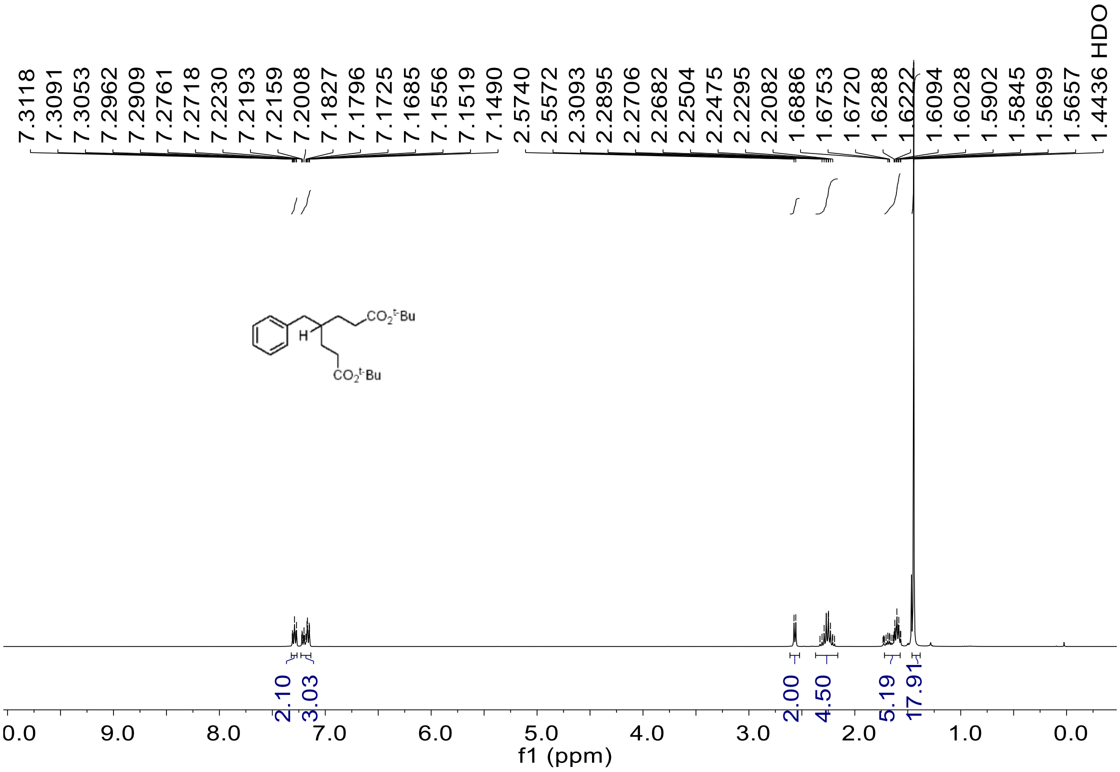


**42e:** $^{13}$C NMR (101 MHz, Chloroform-*d*)

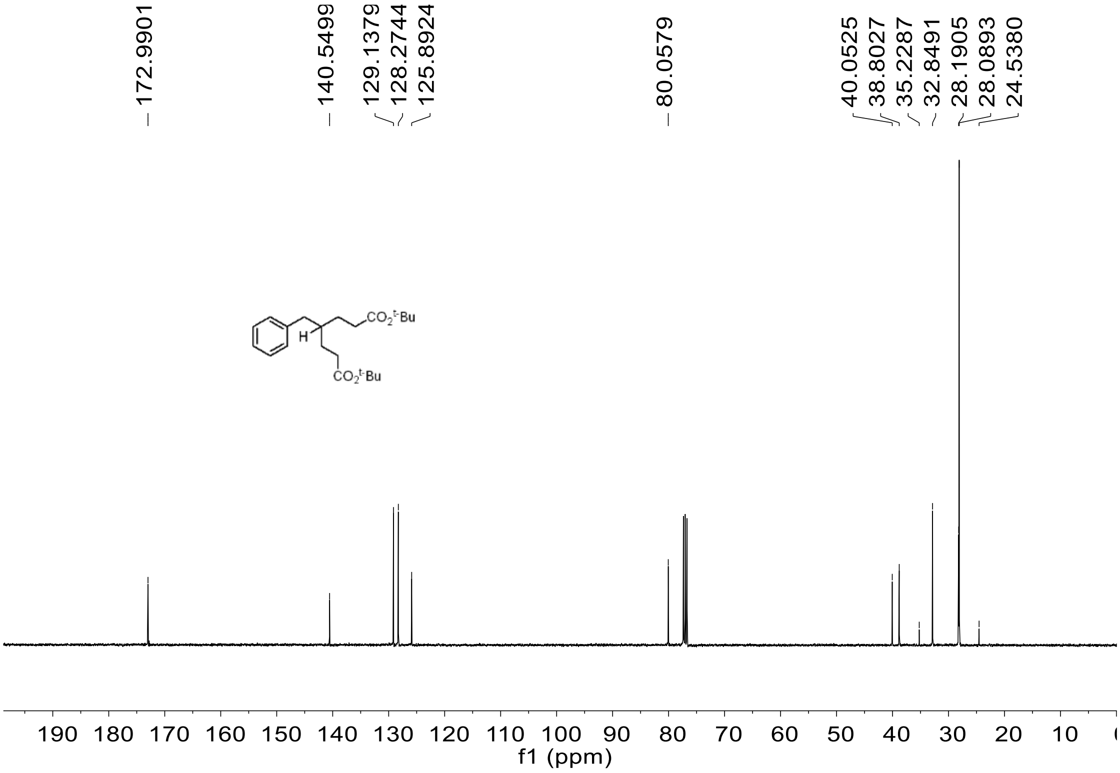

**42f:** $^{1}$H NMR (400 MHz, Chloroform-*d*)

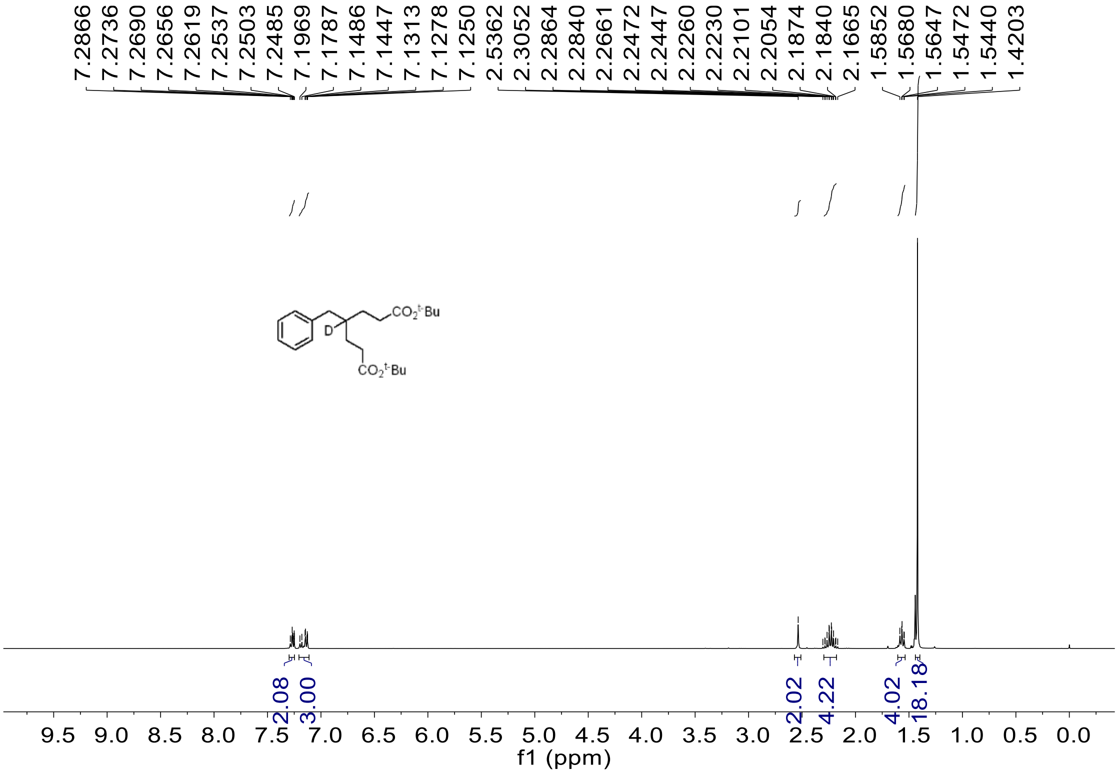


**42f:** $^{13}$C NMR (101 MHz, Chloroform-*d*)

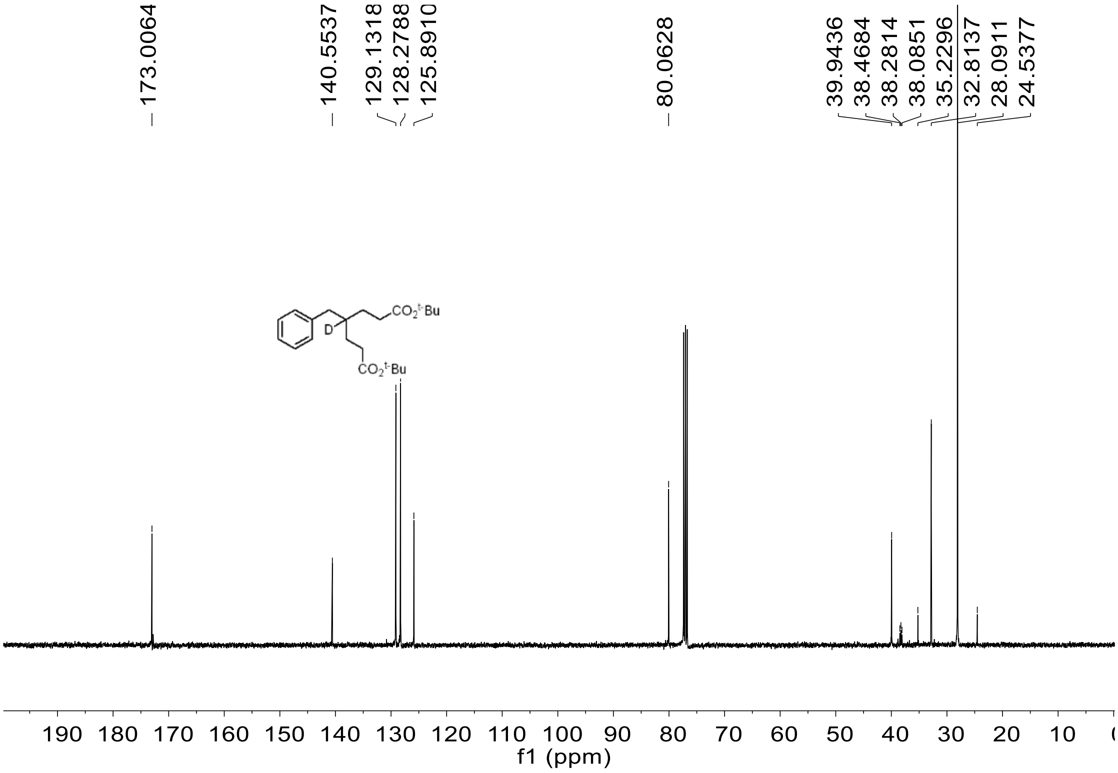

**43e:** $^{1}$H NMR (400 MHz, Chloroform-*d*)

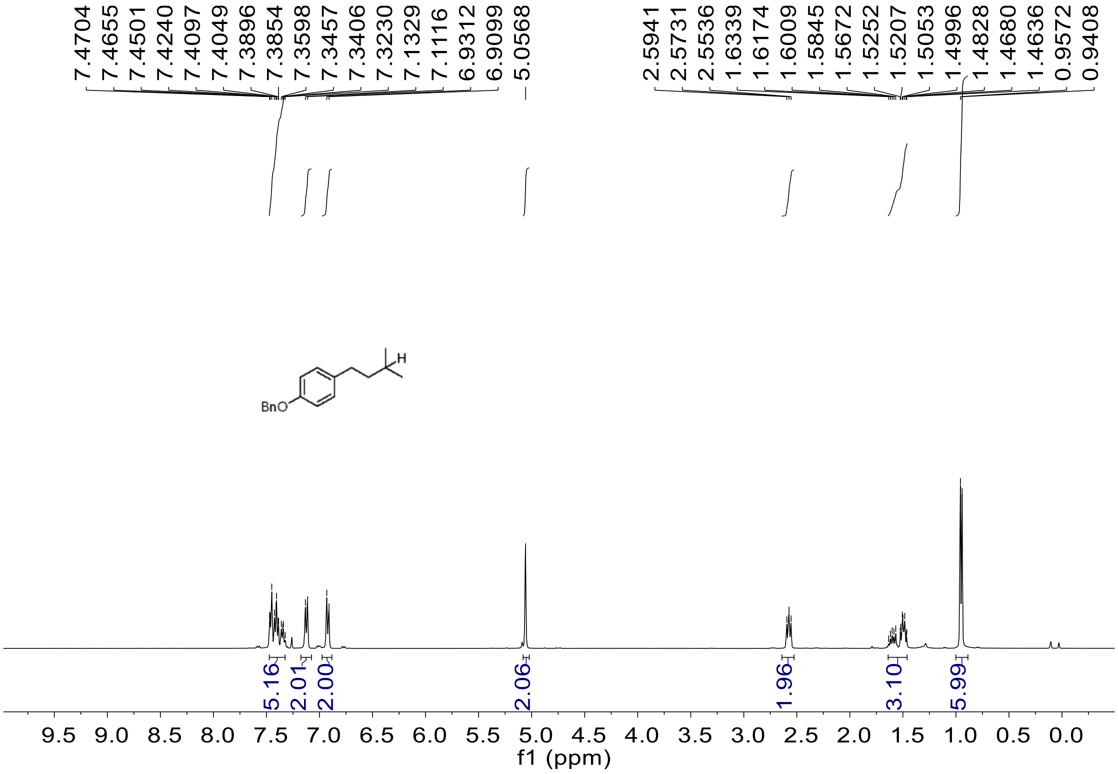

**43e:** $^{13}$C NMR (101 MHz, Chloroform-*d*)

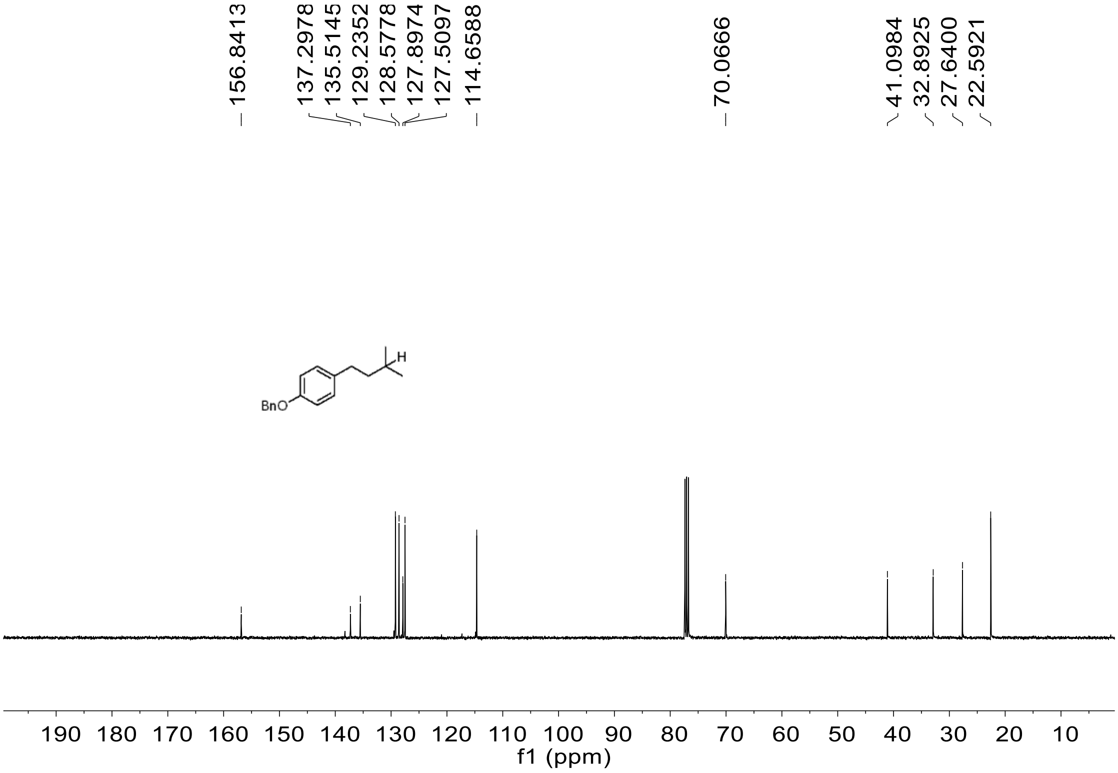

**43f:** $^{1}$H NMR (400 MHz, Chloroform-*d*)

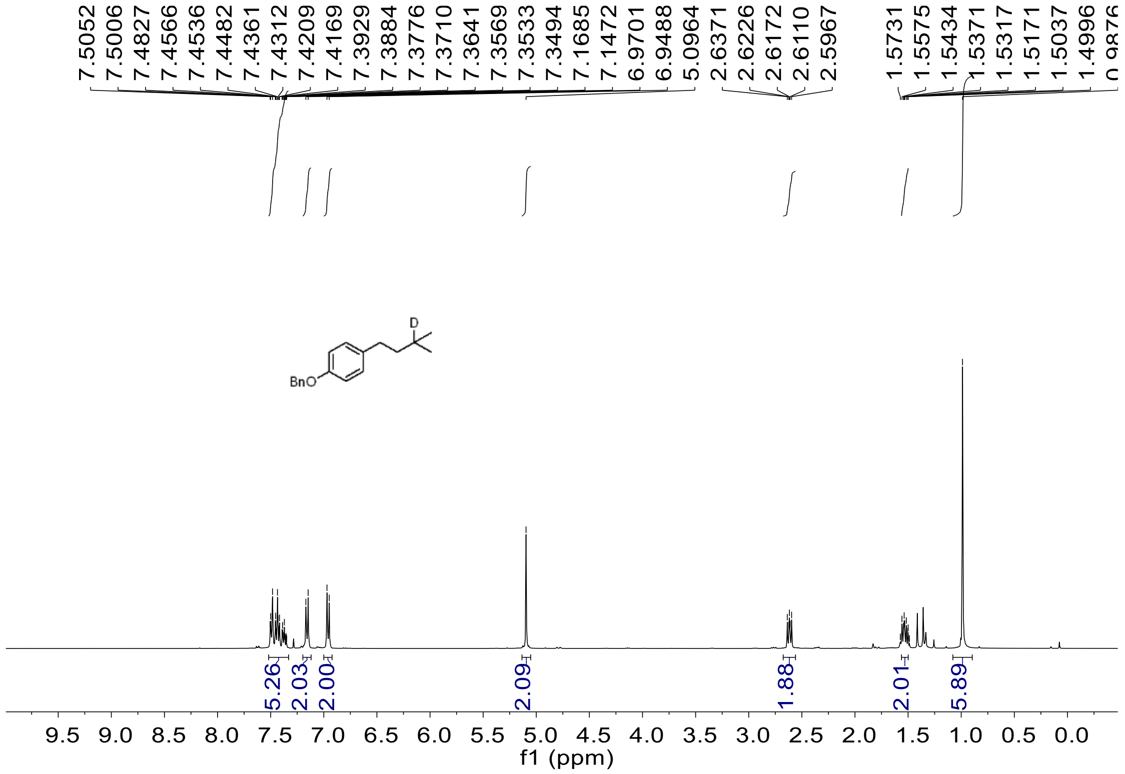

**43f:** $^{13}$C NMR (101 MHz, Chloroform-*d*)

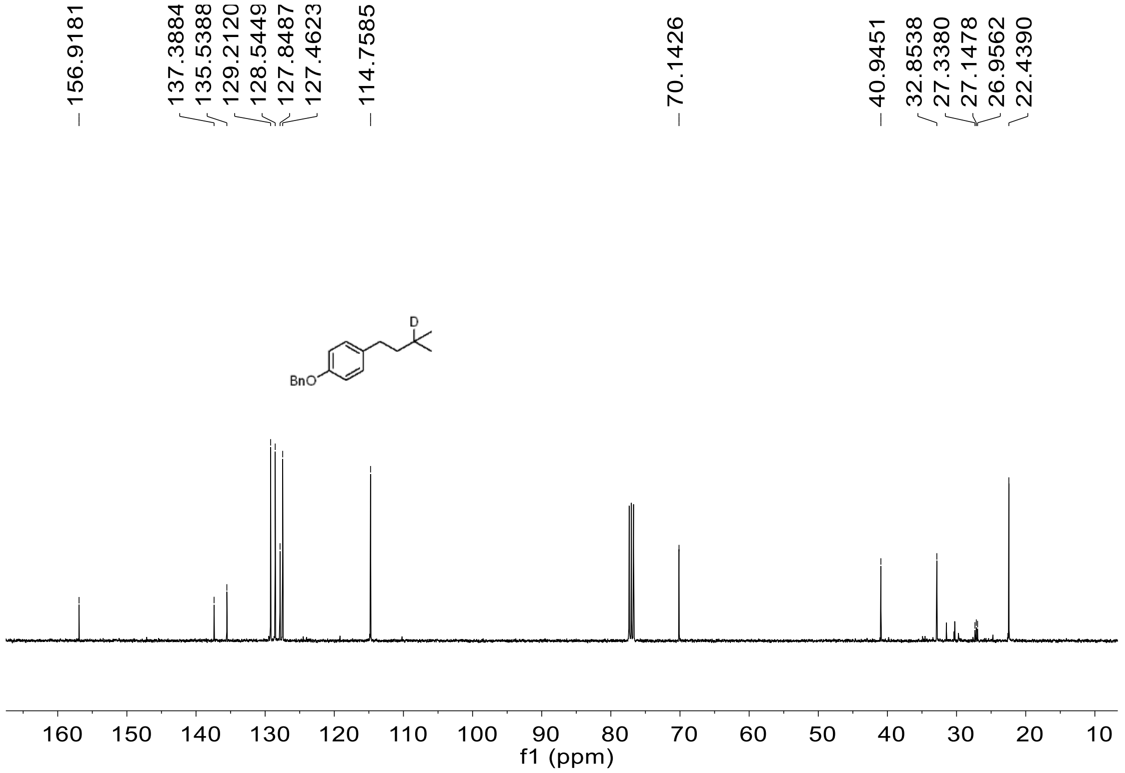

**44c:** $^{1}$H NMR (400 MHz, Chloroform-*d*)

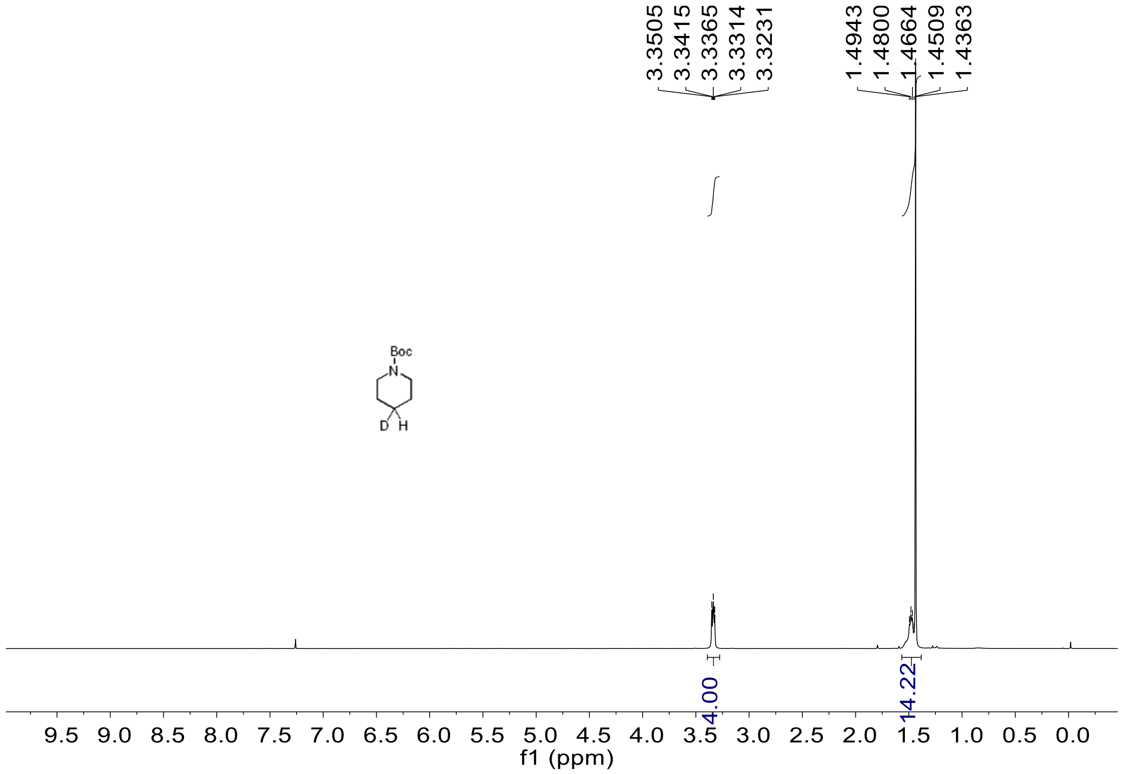


**44c:** $^{13}$C NMR (101 MHz, Chloroform-*d*)

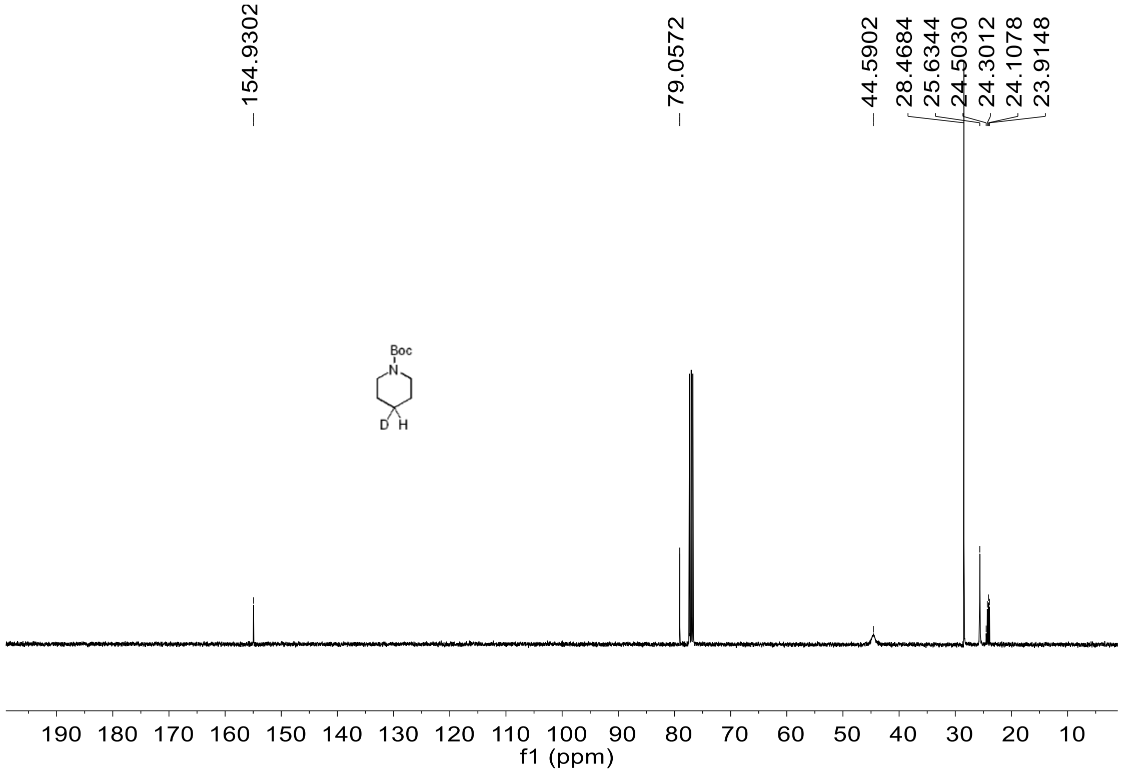

**44c':** $^{1}$H NMR (400 MHz, Chloroform-*d*)

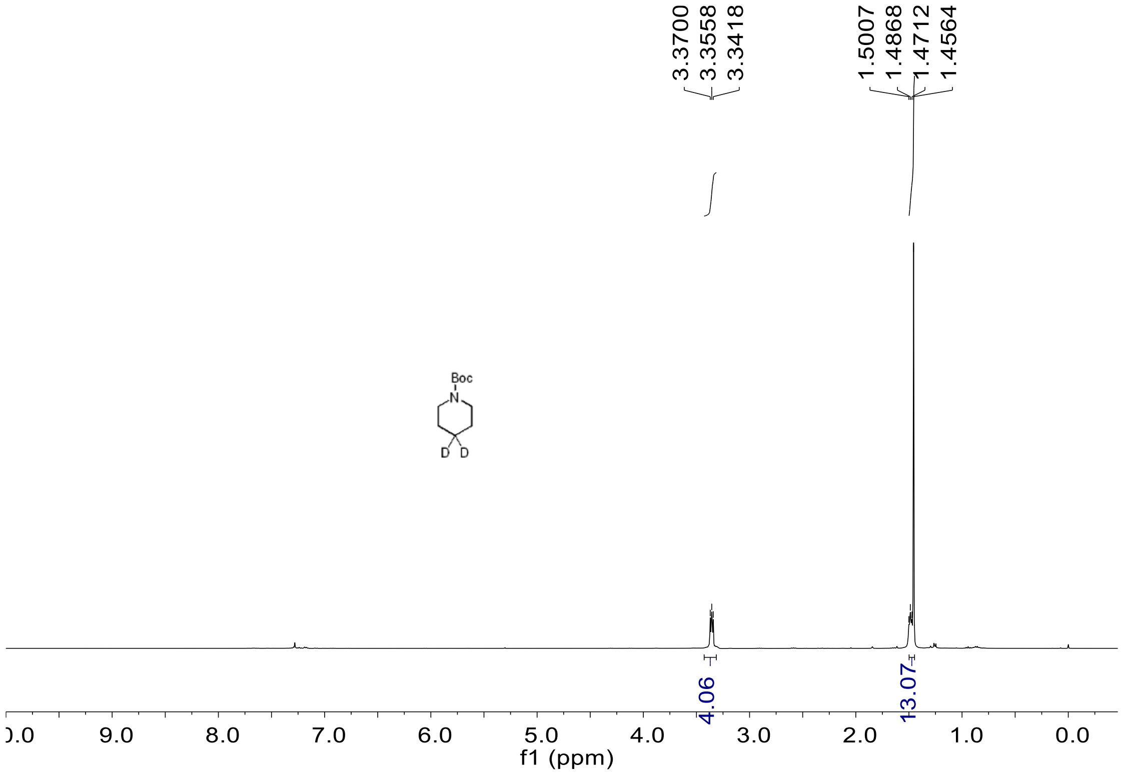


**44c':** $^{13}$C NMR (101 MHz, Chloroform-*d*)

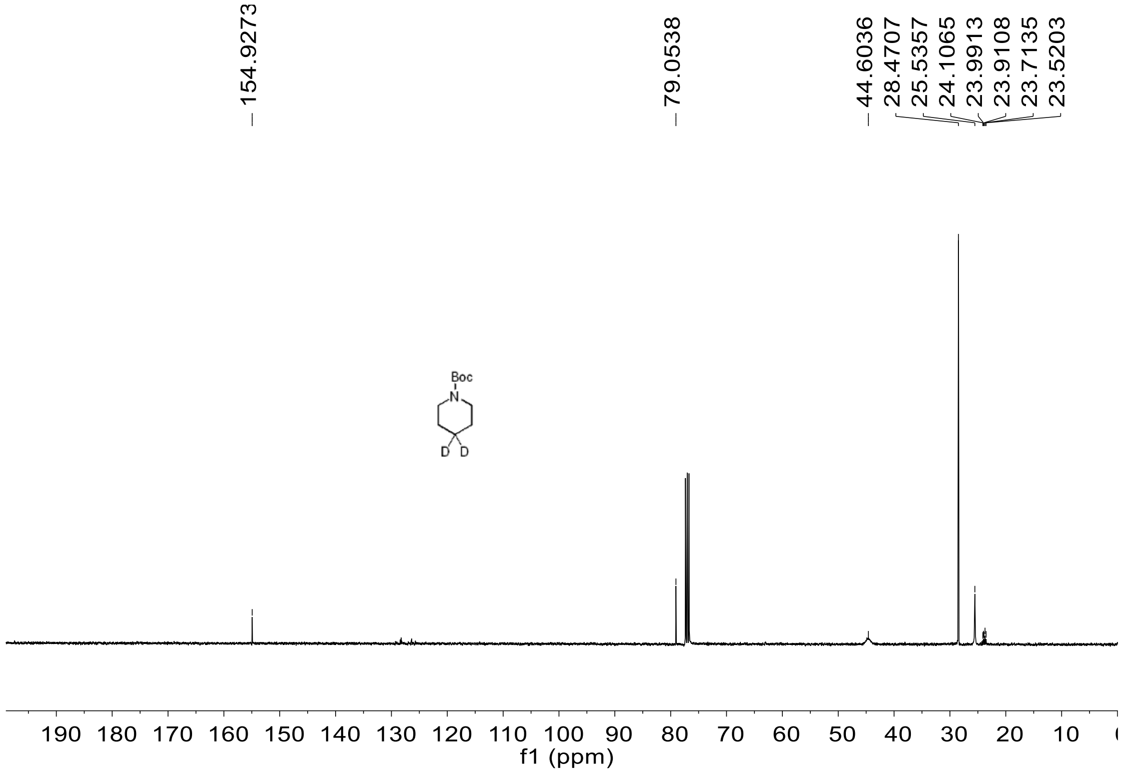

## 9. References


1. H. A. Pal, S. Mohapatra, V. Gupta, S. Ghosh and S. Verma. Self-assembling soft structures for intracellular NO release and promotion of neurite outgrowth. *Chem. Sci.*, **2017**, *8*, 6171–6175.

2. T. Zhang, X. Hu, Z. Wang, T. Yang, H. Sun, G. Li and H. Lu. Carboxylate assisted Iridium-Catalyzed C-H Amination of Arenes with Biologically Relevant Alkyl Azides. *Chem. Eur. J.* **2016**, *22*, 2920–2924.

3. B. Zhao and Z. Shi. Copper-Catalyzed Intermolecular Heck-Like Coupling of Cyclobutanone Oximes Initiated by Selective C-C Bond Cleavage. *Angew. Chem. Int. Ed.* **2017**, *56*, 12727–12731.

4. X. Yu, J. Chen, P. Wang, M. Yang, D. Liang and W. Xiao. Visible-Light-Driven Iminyl Radical-Mediated C-C Single Bond Cleavage/Radical Addition Cascade of Oxime Esters. *Angew. Chem. Int. Ed.* **2018**, *57*, 738 –743.

5. A. H. Dekmezian and M. K. Kaloustian. An Efficient and Unambiguous Synthesis of 2-Hydroxymethyl-1, 3-Propanediol. *Syn. Commun.* **1979**, *9*, 431-435.

6. X. Xu, A. Azuma, A. Kusuda, E. Tokunaga and N. Shibata. Suzuki–Miyaura Cross-Coupling Reactions in a Solkane365/227/Ethanol Blend at Ambient Temperature. *European Journal of Organic Chemistry* **2012**, *8*, 1504-1508.

7. Q. Liu, Y. Liu, H. Song and Q, Wang. Electron Transfer Photoredox Catalysis: Development of aPhotoactivated Reductive Desulfonylation of an Aza-Heteroaromatic Ring. *Advanced Synthesis & Catalysis* **2020**, *362*, 3110-3115.
