# Supplementary material for: Hydro- and deutero-deamination of primary amines using O-diphenylphosphinylhydroxylamine
Source: Nat Commun. 2024 Nov 24;15:10190. doi: 10.1038/s41467-024-54599-y (PMC11586428; doi:10.1038/s41467-024-54599-y)
Supplement: Supplementary file 2 — Transparent Peer Review file [file 41467_2024_54599_MOESM2_ESM.pdf]

nature portfolio

Peer Review File

Hydro- and Deutero-deamination of Primary Amines using O-Diphenylphosphinylhydroxylamine

Corresponding Author: Professor Hongjian Lu

This file contains all reviewer reports in order by version, followed by all author rebuttals in order by version.

Version 0:

Reviewer comments:

Reviewer #1

(Remarks to the Author)
The authors describe a conversion of primary amino groups of aliphatic and aromatic substrates to hydrogen or deuterium atom using O-diphenylphosphinylhydroxylamine (hydrodeamination and deuterodeamination). The reaction proceeds at 50 degree for a short time, and the corresponding hydrogenated and deuterated products are obtained in good to excellent yields from primary, secondary, tertiary alkylamines and aniline derivatives including biologically active molecules. Several hydrodeamination of primary amines have already been reported as described in the introduction section and shown in Figure 1A. However, the hydrogen atom source of this reaction is H2O, deuterium atom can easily be introduced using D2O. This reaction may become a useful method to introduce a deuterium atom into organic compounds. Therefore, this manuscript is suitable for publication in Nat. Commun. after revisions according to the following comments:

1. In Figure 1B, “hypothesize” should be “Hypothesis”.

2. In Figure 1B, the authors hypothesized this reaction (down equation) based on their previous report (upper equation). If so, the hypothesized intermediate in this reaction should be monoalkyltriazanium.

3. In Figure 1C, the meaning of the abbreviations S.C.H and S.C.D should be written in the figure, not only in the main text. This is the same with S.C. in Figure 3A.

4. In Figure 2, the results of deuterodeamination of 32a-34a should also be inserted, even though the yields and %D values were moderate.

5. In Figure 5, several substrates have chiral center at the carbon atom connected with primary amino group. How about the stereochemistry after hydrodeamination? It may be difficult to maintain the stereochemistry after the reaction due to the formation of radical intermediates.

6. Judging from several mechanistic experiments in Figure 4, it is reasonable to consider radical pathway. If so, the formed alkyl or aryl radicals could abstract a hydrogen atom from the alpha-position of THF. If so, the %D value of the products should decrease. How about the reactions in D2O/THF-D8 instead of D2O/THF? (The abstraction of hydrogen atom of isodiazene may be much faster than that of THF.)

Reviewer #2

(Remarks to the Author)
Lu and coworkers describe a practical and scalable hydro- and deuterodeamination of primary amines using O-diphenylphosphinylhydroxylamine (DPPH). The reaction enables the hydro- and deuterodeamination of a large variety of primary amines with different substitution in the alpha-carbon. The reaction conditions show a broad functional group compatibility and several biologically active compounds have been successfully deaminated. Morover, the authors developed conditions to achieve the deuterodeamination. While this protocol is clearly practical, it is very similar to the hydrodeamination reported by Levin in 2021 (J. Am. Am. Soc. 2021, 3, 17366). In my opinion, compared to Levin’s work, it provides a convenient alternative reagent rather than a real conceptual advance. I do not agree with the author’s statement when they define these results as groundbreaking. Moreover, this manuscript is a natural extension of the author's previous

work on N-deletion (Nat. Synth. 2024, 913) which again diminish the novelty of this study. From the mechanistic point of view, there is nothing surprising giving the similarity of this work with Levin's publication. The control experiments and conclusions are almost identical in both publications. Finally, the authors have omitted important recent two-step hydrodeamination protocols in their introduction, which does not reflect the actual state of the art (for example: Angew. Chem. Int. Ed. 2024, e202405779; Angew. Chem. Int. Ed. 2024, e202317683). I think this is a nice contribution but it does note provide the level of novelty and conceptual advance that one would expect from Nature Communications.

Reviewer #3

(Remarks to the Author)
Lu et.al. reported an new method for deutero-deamination of primary amines using the commercially available reagent. O-Diphenylphosphinylhydroxylamine. The is deamination reaction is only known twice by Oestreich 2020 (ref 38) and Levin 2021 (ref 39). This manuscript has several advantages over the reported methods such as the use of commercial reagent, mild conditions and the broad scope of primary, secondary, tertiary and aryl amine.
However, the necessity of the use of super stoichiometric amounts of the deamination reagents, I would suggest acceptance of this work in Nat. Commun. after minor revision.

- It would be useful to report on the tolerance of the secondary amine and ketones, aldehydes functional groups.
-It suggest the citation of the following papers: Org. Biomol. Chem., 2022,20, 1379-1385, Synthesis 2024; 56(01): 87-106, ChemSusChem 2022, 15, e202200402, ACS Catal. 2023, 13, 21, 14205–14212

Version 1:

Reviewer comments:

Reviewer #1

(Remarks to the Author)
This reviewer reviewed the former version of the manuscript, which describes a conversion of primary amino groups of aliphatic and aromatic substrates to hydrogen or deuterium atom using O-diphenylphosphinylhydroxylamine (hydrodeamination and deuterodeamination). The authors revised the manuscript based on the reviewers' comments and provide careful explanations and counterarguments, especially regarding the comments from Reviewer 2. Therefore, this reviewer thinks that this revised manuscript is suitable for publication in Nat. Commun.

Reviewer #3

(Remarks to the Author)
The authors have made the requested revision accordingly. Thus the reviewer suggests acceptance

Open Access This Peer Review File is licensed under a Creative Commons Attribution 4.0 International License, which

permits use, sharing, adaptation, distribution and reproduction in any medium or format, as long as you give appropriate credit to the original author(s) and the source, provide a link to the Creative Commons license, and indicate if changes were made.
In cases where reviewers are anonymous, credit should be given to 'Anonymous Referee' and the source.
The images or other third party material in this Peer Review File are included in the article's Creative Commons license, unless indicated otherwise in a credit line to the material. If material is not included in the article's Creative Commons license and your intended use is not permitted by statutory regulation or exceeds the permitted use, you will need to obtain permission directly from the copyright holder.
To view a copy of this license, visit https://creativecommons.org/licenses/by/4.0/

Reviewer #1 (Remarks to the Author):

The authors describe a conversion of primary amino groups of aliphatic and aromatic substrates to hydrogen or deuterium atom using O-diphenylphosphinylhydroxylamine (hydrodeamination and deuterodeamination). The reaction proceeds at 50 degree for a short time, and the corresponding hydrogenated and deuterated products are obtained in good to excellent yields from primary, secondary, tertiary alkylamines and aniline derivatives including biologically active molecules. Several hydrodeamination of primary amines have already been reported as described in the introduction section and shown in Figure 1A. However, the hydrogen atom source of this reaction is H2O, deuterium atom can easily be introduced using D2O. This reaction may become a useful method to introduce a deuterium atom into organic compounds. Therefore, this manuscript is suitable for publication in Nat. Commun. after revisions according to the following comments:
Response: *Thank you for your thorough review, insightful comments, and the positive assessment of our work. We appreciate your valuable feedback and the opportunity to improve our manuscript. Below, we address each of your comments in detail, and we are confident that the revisions will further enhance the quality of the paper.*

1. In Figure 1B, “hypothesize” should be “Hypothesis”.
Response: *Thank you for your careful review and for pointing out the incorrect use of the term "hypothesize." As suggested, we have corrected “hypothesize” to “Hypothesis” in Figure 1B (now Figure 1b in the revised version).*

2. In Figure 1B, the authors hypothesized this reaction (down equation) based on their previous report (upper equation). If so, the hypothesized intermediate in this reaction should be monoalkyltriazanium.
Response: *Thank you for your thoughtful and insightful comment. In accordance with your suggestion, we have revised the hypothesized intermediate to monoalkyltriazanium. This change has been implemented in both Figure 1B (now Figure 1b in the revised version) and Figure 1F (now Figure 1f in the revised version).*

3. In Figure 1C, the meaning of the abbreviations S.C.H and S.C.D should be written in the figure, not only in the main text. This is the same with S.C. in Figure 3A.
Response: *Thank you for your thoughtful suggestion. We agree that adding the full terms will improve clarity. In Figure 1C (now Figure 1c in the revised manuscript), we have replaced the abbreviations “S.C.H” and “S.C.D” with “hydro-deamination” and “deutero-deamination,” respectively. Similarly, in Figure 3 (and Figure 4), we now use “S.C.H. or S.C.D.” with the meanings of these abbreviations provided in the figure notes, replacing the original “S.C.”.*

4. In Figure 2, the results of deuterodeamination of 32a-34a should also be inserted, even though the yields and %D values were moderate.

***Response:** As requested, the yields and %D values for the deuterodeamination of **32a-34a** (revised **35a-37a**) have been incorporated into the revised Figure 2, despite the moderate outcomes.*

5. In Figure 5, several substrates have chiral center at the carbon atom connected with primary amino group. How about the stereochemistry after hydrodeamination? It may be difficult to maintain the stereochemistry after the reaction due to the formation of radical intermediates.

***Response:** As demonstrated in the reaction of substrate **27a** (revised **39a**), the chiral center at the α-position of the amino group is lost following hydrodeamination. To further confirm this, we conducted an additional reaction using a new substrate, metirosine **29a**, which contains a chiral α-tertiary amino group. This resulted in a racemized product, clearly indicating that stereochemistry is not retained. These findings support the proposed radical mechanism for the reaction.*

6. Judging from several mechanistic experiments in Figure 4, it is reasonable to consider radical pathway. If so, the formed alkyl or aryl radicals could abstract a hydrogen atom from the alpha-position of THF. If so, the %D value of the products should decrease. How about the reactions in D2O/THF-D8 instead of D2O/THF? (The abstraction of hydrogen atom of isodiazene may be much faster than that of THF.)

***Response:** Thank you for your thoughtful and insightful comment. Given that the reactions of alkyl amines in D_2O/THF yielded desired products with high %D values, we conducted the reaction of aromatic amine **34a** in D_2O/d_8-THF, as shown in the new Figure 2 (see the note m). The %D value of the product increased from 59% to 69%. These results agree with your hypothesis that the abstraction of hydrogen atom of isodiazene may be much faster than that of THF.*

Reviewer #2 (Remarks to the Author):

Lu and coworkers describe a practical and scalable hydro- and deuterodeamination of primary amines using O-diphenylphosphinylhydroxylamine (DPPH). The reaction enables the hydro- and deuterodeamination of a large variety of primary amines with different substitution in the alpha-carbon. The reaction conditions show a broad functional group compatibility and several biologically active compounds have been successfully deaminated. Morover, the authors developed conditions to achieve the deuterodeamination.

***Response:** Thank you for reviewing our manuscript and highlighting some key aspects of our work. We appreciate your acknowledgment of the practical and scalable hydro- and deuterodeamination of primary amines. We are pleased that you recognized the broad functional group compatibility and the successful deamination of biologically active compounds.*

While this protocol is clearly practical, it is very similar to the hydrodeamination reported by Levin in 2021 (J. Am. Am. Soc. 2021, 3, 17366). In my opinion, compared to Levin's work, it provides a

convenient alternative reagent rather than a real conceptual advance. I do not agree with the author's statement when they define these results as groundbreaking. Moreover, this manuscript is a natural extension of the author's previous work on N-deletion (Nat. Synth. 2024, 913) which again diminish the novelty of this study. From the mechanistic point of view, there is nothing surprising giving the similarity of this work with Levin's publication. The control experiments and conclusions are almost identical in both publications.

Response: *While I appreciate the reviewer's summary, I would like to clarify several key differences between our work and Levin's 2021 study.* ***Synthetic Scope****: Our method accommodates a broader range of primary amines, including sterically hindered α-tertiary alkylamines, which Levin's approach does not efficiently process. Moreover, our method generally yields higher product purity and efficiency. For example, in the reaction of leelamine* ***37b*** *(Figure 3a), we achieved 97% yield with a simple acidic water wash, while Levin's method achieved only 65% yield and required column chromatography. Additionally, our use of H_2O as a hydrogen atom source facilitates easy deuterium incorporation using D_2O, offering an efficient means of introducing deuterium into organic compounds, as also noted by the first reviewer.* ***Novel Mechanism****: Levin's work involves a HERON rearrangement to form an isodiazene intermediate, followed by a novel rearrangement of that intermediate. In contrast, our study introduces a previously unrecognized monoalkyltriazanium intermediate, which rearranges to form the isodiazene. This distinct intermediate represents a significant mechanistic advance in our understanding of this reaction pathway. With regard to the suggestion that our manuscript is an extension of our previous N-deletion work (Nat. Synth. 2024, 913), we respectfully disagree. Our previous work relied on in-cage biradical coupling facilitated by a benzyl group, whereas the current study does not require benzyl groups, utilizing non-benzyl amines instead. Mechanistically, the N-deletion study involved in-cage biradical coupling, while the current work operates through an out-of-cage hydrogen atom transfer (HAT) process, further distinguishing the two studies. In conclusion, we believe our work offers important advances in both methodology and mechanistic insight.*

Finally, the authors have omitted important recent two-step hydrodeamination protocols in their introduction, which does not reflect the actual state of the art (for example: Angew. Chem. Int. Ed. 2024, e202405779; Angew. Chem. Int. Ed. 2024, e202317683).

Response: *Thank you for your feedback. It appears that the reviewer may have overlooked details in our original submission. In the second paragraph of the introduction, we specifically mentioned that "Consequently, the two-step indirect deamination methods involving the pre-activation of primary amines have been developed, in which the conversion of aliphatic primary amines to reactive isonitriles[34,35] or Katritzky-type pyridinium salts[36,37] represents widely employed strategies." We have cited the relevant recent publications in original references 34 and 35: "34. Quirós, I. et al. "Isonitriles as Alkyl Radical Precursors in Visible Light Mediated Hydro– and Deuterodeamination Reactions." Angew. Chem., Int. Ed., e202317683 (2023). And 35. Jiao, Z. et al. "Unified Approach to Deamination*

and Deoxygenation Through Isonitrile Hydrodecyanation: A Combined Experimental and Computational Investigation." Angew. Chem., Int. Ed., e202405779 (2024).".

I think this is a nice contribution but it does note provide the level of novelty and conceptual advance that one would expect from Nature Communications.
Response: *Thank you for your thoughtful feedback. In response to your comments and those of the other reviewers, we have carefully revised the manuscript to emphasize the novel aspects of our work. The expanded synthetic scope and the discovery of the monoalkyltriazanium intermediate represent significant methodological and mechanistic advancements. Given the positive feedback from the other two reviewers and the substantial improvements made, we hope that the revised manuscript now meets the standards for publication in Nature Communications.*

Reviewer #3 (Remarks to the Author):

Lu et.al. reported an new method for deutero-deamination of primary amines using the commercially available reagent. O-Diphenylphosphinylhydroxylamine. The is deamination reaction is only known twice by Oestreich 2020 (ref 38) and Levin 2021 (ref 39). This manuscript has several advantages over the reported methods such as the use of commercial reagent, mild conditions and the broad scope of primary, secondary, tertiary and aryl amine. However, the necessity of the use of super stoichiometric amounts of the deamination reagents, I would suggest acceptance of this work in Nat. Commun. after minor revision.
Response: *Thank you for your insightful comments and your recommendation for acceptance after minor revisions. We appreciate your recognition of the advantages of our method, including the use of a commercially available reagent, mild reaction conditions, and the broad substrate scope.*

It would be useful to report on the tolerance of the secondary amine and ketones, aldehydes functional groups.

Response: *Thank you for the suggestion. As recommended, we have tested two new substrates **2a** and **13a** containing ketone and aldehyde functional groups, respectively, and the results are presented in Figure 2. Moderate yields of the desired products were observed, likely due to imine formation, which competes with the intended reaction and reduces efficiency. Nevertheless, the method shows reasonable tolerance for these functional groups.*

It suggest the citation of the following papers: Org. Biomol. Chem., 2022,20, 1379-1385, Synthesis 2024; 56(01): 87-106, ChemSusChem 2022, 15, e202200402, ACS Catal. 2023, 13, 21, 14205–14212

Response: *Thank you for the suggestion. We have incorporated the recommended references in the revised manuscript as follows: McFadden et al. (2022) (Org. Biomol. Chem., 20, 1379−1385), Wohlgemuth (2022) (ChemSusChem, 15, e202200402), and Schwartz et al. (2023) (Synthesis, 56, 87−106) introduce methods of deaminative C–C bond formation and are now cited as references 33-35. Wang et al. (2023) (ACS Catal., 13, 14205−14212) discusses biomimetic dehydroamination using Katritzky's salt and is cited as reference 40. To comply with the reference limit of 70, we have removed the following: Ouyang et al. (2015) (original ref. 26, Chem. Rev., 115, 12045−12090) due to overlap with Wang et al. (2016) (original ref. 27, revised ref. 26, Chem. Soc. Rev., 45, 1257−1272). Högenauer et al. (2001) (original ref. 51, Bioorg. Med. Chem. Lett., 11, 2627−2630) was removed due to its similarity with Högenauer et al. (2000) (original ref. 52, revised ref. 53, Tetrahedron Lett., 41, 9229−9232). Marsura & Luu Duc (1982) (original ref. 41, Synthesis, 595.) was deemed unnecessary.*
